# An Immunoinformatic Approach for Identifying and Designing Conserved Multi-Epitope Vaccines for Coronaviruses

**DOI:** 10.3390/biomedicines12112530

**Published:** 2024-11-05

**Authors:** Yu Chuan Ong, Bimo Ario Tejo, Wei Boon Yap

**Affiliations:** 1 Center for Toxicology and Health Risk Studies, Faculty of Health Sciences, Universiti Kebangsaan Malaysia, Kuala Lumpur 50300, Malaysia; p131331@siswa.ukm.edu.my; 2 Department of Chemistry, Faculty of Science, Universiti Putra Malaysia, Serdang 43400, Malaysia; bimo.tejo@upm.edu.my; 3 One Health UKM, Universiti Kebangsaan Malaysia, Bangi 43600, Malaysia

**Keywords:** SARS-CoV-2, coronavirus, universal vaccine, peptide, cell-mediated immunity

## Abstract

Background/Objectives: The COVID-19 pandemic caused by the novel severe acute respiratory syndrome coronavirus 2 (SARS-CoV-2) virus has exposed the vulnerabilities and unpreparedness of the global healthcare system in dealing with emerging zoonoses. In the past two decades, coronaviruses (CoV) have been responsible for three major viral outbreaks, and the likelihood of future outbreaks caused by these viruses is high and nearly inevitable. Therefore, effective prophylactic universal vaccines targeting multiple circulating and emerging coronavirus strains are warranted. Methods: This study utilized an immunoinformatic approach to identify evolutionarily conserved CD4+ (HTL) and CD8+ (CTL) T cells, and B-cell epitopes in the coronaviral spike (S) glycoprotein. Results: A total of 132 epitopes were identified, with the majority of them found to be conserved across the bat CoVs, pangolin CoVs, endemic coronaviruses, SARS-CoV-2, and Middle East respiratory syndrome coronavirus (MERS-CoV). Their peptide sequences were then aligned and assembled to identify the overlapping regions. Eventually, two major peptide assemblies were derived based on their promising immune-stimulating properties. Conclusions: In this light, they can serve as lead candidates for universal coronavirus vaccine development, particularly in the search for pan-coronavirus multi-epitope universal vaccines that can confer protection against current and novel coronaviruses.

## 1. Introduction

Coronaviruses (CoVs) were thought to only cause common colds in humans until 2002–2003, when severe acute respiratory syndrome-coronavirus (SARS-CoV) struck the world health system. The SARS-CoV outbreak caused 8098 infections and 774 deaths globally (~10% mortality) [1]. Nearly 10 years after the SARS outbreak, another coronavirus outbreak took place in the Middle East and the etiological agent was identified as Middle East respiratory syndrome coronavirus (MERS-CoV). The outbreak then spread to South Korea, with the first case reported in 2015 involving a 65-year-old man who had recently travelled to the Middle East [2]. Compared to SARS-CoV, MERS-CoV had the highest mortality rate of ~35% among the three outbreak strains, with a total of 2458 infections and 848 reported deaths. In December 2019, a novel coronavirus strain namely SARS-CoV-2 caused a large-scale viral outbreak in WuHan, China. Since then, it has spread rampantly throughout the world and caused respiratory distress in humans. The disease associated with SARS-CoV-2 was coined COVID-19 and resulted in the breakdown of the healthcare system in many countries. The outbreak was eventually announced as a global pandemic by WHO on 11 March 2020 [3]. As of July 2024, COVID-19 has affected more than 775 million people and caused more than 7 million deaths worldwide [4].

As the coronaviral spike (S) glycoprotein is located outside the viral particle and mediates viral entry into host epithelial cells, it is undoubtedly the main target of neutralizing antibodies (NAbs) upon infection, making it the most important therapeutic target and the main focus in vaccine design. However, the emergence of new SARS-CoV-2 Omicron variants has rendered the vaccines ineffective, with ChAdOx1 nCoV-19 (Vaxzevria, AstraZeneca) conferring almost no protection from 20–24 weeks after the second dose of vaccine [5]. It is also notable that the emergence of new variants of concern (VOC), such as Omicron, has attracted attention globally as the new variants can escape the neutralizing antibodies and have increased transmissibility due to the presence of more than 30 mutations as compared to the parental strain, SARS-CoV-2-Wuhan-Hu-1 [6,7,8,9]. In view of the rising concerns regarding the increased infectivity of the new variants and controversies about the effectiveness of the existing vaccines, there is an urgent need for a pan-coronavirus vaccine that can induce the synthesis of neutralizing antibodies and is more comprehensive in conferring protection against the newly emerging variants as well as future coronavirus outbreaks While many groups have predicted and identified evolutionarily conserved epitopes in silico [10,11,12,13,14,15,16], and some of them were validated in vitro and in vivo [17,18,19,20,21,22,23], this study scrutinized the conserved epitopes further. Many predicted cytotoxic T lymphocyte (CTL), helper T lymphocyte (HTL), and linear B lymphocyte (LBL) epitopes were found in close vicinity to the S glycoprotein. Instead of studying them individually, they were aligned into single and relatively long peptide sequences. This novel strategy of having multi-epitopes is expected to stimulate a stronger and multi-faceted immune response against coronaviruses, addressing the limitations of the current vaccines against the emerging variants.

In this study, the evolutionarily conserved epitopes in both human and animal coronaviruses were identified using unique immunoinformatic approaches. After a stringent scrutiny and selection, 52 CTL epitopes, 11 HTL epitopes, and 68 linear B-lymphocyte (LBL) epitopes were identified from 30 coronavirus sequences derived from human CoVs (hCoVs) responsible for the common cold, SARS-CoV, MERS-CoV and SARS-CoV-2. Subsequently, the predicted epitopes were aligned and assembled into two final composite peptide sequences that were found to be evolutionarily conserved across SARS-CoVs, bats, and pangolin coronaviruses. These two assembled epitopes were not only found to be conserved in many of the coronavirus strains, but they also possessed HTL, CTL, and B-cell antigen binding sites, and they matched a diverse array of HLA class I and II supertypes prevalent in the human population, indicating their potential to activate both T and B cells effectively. Although these epitopes were identified on the basis of being conserved in SARS-CoVs and bat and pangolin coronaviruses, their distinctive compatibility with human HTL and CTL and B cells renders them of high potential in vaccine development. Altogether, these discoveries not only pave the way for the development of a pan-coronavirus multiepitope vaccine to combat existing and novel coronavirus strains but its immunoinformatics are highly applicable in universal vaccine development, especially in identifying immunogenic conserved epitopes in target antigens.

## 2. Materials and Methods

### 2.1. Coronaviral S Gene Sequence Retrieval and Sequence Conservation Analysis

Forty-two coronaviral peptide sequences of the S gene were retrieved from the NCBI GenBank (https://www.ncbi.nlm.nih.gov/genbank/ (accessed on 28 July 2024)) as listed in Table 1, Table 2 and Table 3. A total of 24 sequences encompassing SARS-CoV-2 and its variants were retrieved from the NCBI GenBank (Table 1) with the latest variant being XBB.1.5. Six sequences of the other coronaviruses causing SARS, MERS and common colds in humans are listed in Table 2, and Table 3, on the other hand, tabulates twelve sequences of coronaviruses isolated from bats, pangolins and birds.

In order to identify the conserved regions in the coronaviral S glycoproteins, the amino acid sequence of the S glycoprotein of SARS-CoV-2 Wuhan-Hu-1 strain (Table 1) was used as a reference sequence to perform Clustal Omega multiple sequence alignments in the EMBL-EBI (https://www.ebi.ac.uk/ (accessed on 28 July 2024)). The alignment was based on the Percentage Identity Threshold of 80% in the amino acid sequences using Jalview 2.11.2.6 (https://www.jalview.org/ (accessed on 28 July 2024)). The evolutionarily conserved regions of the S glycoproteins were identified and subjected to antigenicity screening, selection, and assembly.

### 2.2. The Flow of Prediction of Conserved HTL, CTL and Linear B-Lymphocyte (LBL) Epitopes of Coronaviral S Glycoproteins

**Figure 1:**
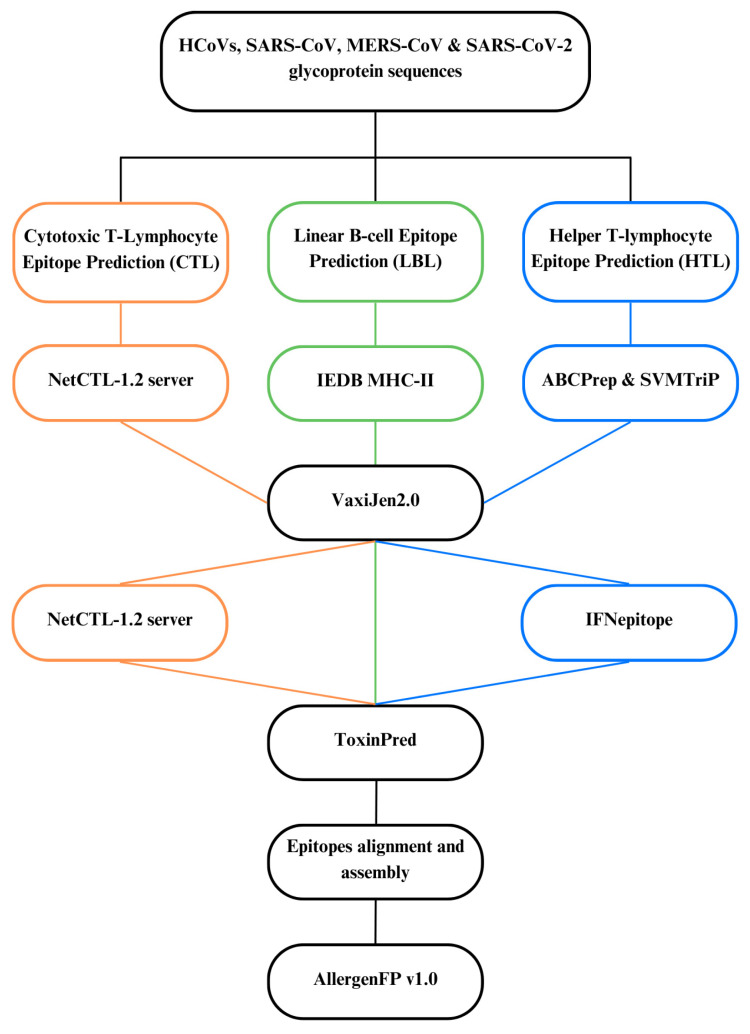
Flow of in silico prediction of conserved epitopes of the coronaviral S proteins. The orange, green and blue-colored lines represent the CTL, LBL and HTL prediction steps, respectively.

The prediction was performed separately for (i) CTL, (ii) HTL, and (iii) LBL epitopes by referring to their respective databases. The flow of prediction of the conversed epitopes is depicted in Figure 1. The conserved epitopes were individually screened and identified, and their antigenicity and toxigenicity were predicted using VaxiJen 2.0 and ToxinPred, respectively.

#### 2.2.1. Prediction of Conserved CTL Epitopes

The conserved CTL epitopes were identified using NetCTL-1.2 (https://services.healthtech.dtu.dk/services/NetCTL-1.2/ (accessed on 1 August 2024)). A total of 30 amino acid sequences of human coronaviral S glycoproteins were uploaded to NetCTL-1.2 by following the default criteria, which entailed 9 amino acids in length with a minimum threshold of 0.75. The available HLA class I supertypes provided by NetCTL-1.2 included A1, A2, A24, A26, B7, B8, B27, B39, B44, B58, and B62. The redundant epitope sequences were filtered and subjected to the subsequent screening. The selected epitopes were then subjected to in silico antigenicity screening using VaxiJen 2.0 (http://www.ddg-pharmfac.net/vaxijen/VaxiJen/VaxiJen.html (accessed on 1 August 2024)). The screening was performed using the default settings and “Virus” was selected as the target organism as part of the prediction criteria. Antigens labeled as “Probable Antigen” were sorted and selected. Subsequently, the selected antigens were screened for their immunogenicity using the IEDB Class I immunogenicity web-based prediction tool (http://tools.iedb.org/immunogenicity/ (accessed on 1 August 2024)). The non-immunogenic epitopes were indicated with negative scores and removed and the remaining epitopes were later examined using ToxinPred (https://webs.iiitd.edu.in/raghava/toxinpred/protein.php (accessed on 1 August 2024)) to eliminate the probable toxic CTL epitopes. Following that, the epitopes were selected based on their high frequencies across different strains and supertypes. Table 4 summarizes the prediction of CTL epitopes.

#### 2.2.2. Prediction of Conserved HTL Epitopes

In the conserved HTL epitope prediction, the amino acid sequences of the coronaviruses were screened using IEDB MHC-II (http://tools.iedb.org/mhcii/ (accessed on 1 August 2024)) with the following prediction conditions: (i) Percentile rank: 25%, 15-mers amino acid; (ii) prediction method: Consensus 2.22; (iii) HLA supertypes: HLA-DR, HLA-DQ, and HLA-DP. Table 5 summarizes the prediction conditions for the conserved HTL epitopes. Epitopes with a percentile rank lower than 20.0 were eliminated as this indicates that those epitopes capture a less than 50% immune response [24].

Similar to the conserved CTL epitope prediction, the antigenicity and toxigenicity of the epitopes were analyzed using the same methods as shown in Table 4. In addition, the HTL epitopes were screened using IFNepitope (https://webs.iiitd.edu.in/raghava/ifnepitope/predict.php (accessed on 1 August 2024)) for their abilities to induce interferon synthesis. The prediction criteria included “Motif and SVM hybrid” as the prediction approach and “IFN-gamma versus Non IFN-gamma” as the prediction model. The “NEGATIVE” HTL epitopes were removed.

#### 2.2.3. Prediction of Conserved LBL Epitopes

ABCPred (https://webs.iiitd.edu.in/raghava/abcpred/ABC_submission.html (accessed on 1 August 2024)) and SVMTriP (http://sysbio.unl.edu/SVMTriP/prediction.php (accessed on 1 August 2024)) were used for predicting the conserved LBL epitopes with criteria such as 16-mers amino acids in length for both tools and the thresholds of 0.51 and 0.50, respectively, as shown in Table 6. All of the predicted epitopes were selected for further antigenicity and toxigenicity prediction. The antigenicity and toxigenicity of the epitopes were screened as described in Table 4.

### 2.3. Alignment of the Predicted Conserved CTL, HTL and LBL Epitopes and Allergenicity Prediction

To identify the locations of the epitopes identified in Section 2.2, SARS-CoV-2-Wuhan-Hu-1 S glycoprotein was used as a reference sequence for multiple sequence alignment. The overlapped regions of the epitopes were aligned. Then, they were assembled into long amino acid sequences containing the conserved CTL, HTL, and LBL epitopes. Subsequently, the assembled peptide sequences were screened for allergenicity using AllergenFP v1.0 (https://www.ddg-pharmfac.net/AllergenFP/ (accessed on 1 August 2024)) to identify the probable allergens in the assembled sequences. The probable allergenic sequences, if any, were eliminated.

### 2.4. Population Coverage Analysis

The assembled epitopes were subjected to population coverage analysis using the IEDB Population Coverage analysis tool (http://tools.iedb.org/population/ (accessed on 5 October 2024)) with the default parameters. The analysis was conducted based on the following parameters: (i) “World” as the selected area/population, and (ii) the calculation was determined for “Class I”, “Class II”, and “Class I and II combined”. The “MHC-restricted allele(s)” were determined based on the predicted HLA Class I and Class II associated with the assembled epitopes.

### 2.5. Structural Visualisation of Assembled Epitopes

The structural information of the closed (PDB: 6VXX) and open states (PBD: 6VYB) of the S glycoprotein were retrieved from the RCSB Protein Data Bank (https://www.rcsb.org/ (accessed on 10 August 2024)). By using the retrieved information, the structures of the assembled conserved epitopes were then visualized using UCSF ChimeraX (https://www.cgl.ucsf.edu/chimerax/ (accessed on 10 August 2024)).

### 2.6. Molecular Docking of the Assembled Epitopes to TLR4 and TLR2 Receptors

The molecular docking was performed using HADDOCK 2.4 (https://www.bonvinlab.org/software/haddock2.4/ (accessed on 15 October 2024)) to determine the interaction of the assembled epitopes with cell receptors involved in the vaccine-induced immune responses. Specifically, both Toll-Like Receptor 2 (TLR2) (PDB ID: 2z7x) and TLR4 dimer (PDB ID: 4g8a) were selected as target receptors due to their localization on the cell surface and their roles in initiating innate immune responses upon binding to vaccine components [25,26]. The binding was analyzed using PDBsum (https://www.ebi.ac.uk/thornton-srv/databases/pdbsum/ (accessed on 19 October 2024)) and the binding affinity in terms of Gibbs free energy (ΔG) and dissociation constants (M) at 25 °C between the docked molecules were calculated using the PRODIGY web server (https://rascar.science.uu.nl/prodigy/ (accessed on 19 October 2024)) [27,28].

### 2.7. Immune Simulation Using C-IMMSIM Server

Immune simulation was performed on the C-IMMSIM server (https://kraken.iac.rm.cnr.it/C-IMMSIM/ (accessed on 19 October 2024)) to characterize the immune response profile and immunogenicity of the assembled peptides [29]. The entire simulation was performed for 300-time steps, equivalent to about 80 days (a time step is about 8 h). Three peptide injections were given four weeks apart at time steps 10, 94, 178 [29].

## 3. Results

### 3.1. Conserved Regions in the S Glycoproteins of Bat and Pangolin CoV, hCoVs, SARS-CoV-2, SARS-CoV, and MERS-CoV

The S glycoprotein of coronaviruses consists of a signal peptide, a receptor binding subunit (S1) and a fusion subunit (S2) with a length ranging from 1105–1351 amino acids [30,31,32,33]. With reference to the SARS-CoV-2-Wuhan-Hu-1, in the S1 subunit, there is an N-terminal domain (NTD, 14–305 residues) and receptor-binding domain (RBD, 319–541 residues), whereas, in the S2 subunit, there is a fusion peptide (FP, 788–806 residues), heptapeptide repeat sequence 1 (HR1) (912–984 residues), HR2 (1163–1213 residues), transmembrane domain (TM, 1213–1237 residues), and cytoplasmic domain (1237–1273 residues) [34]. As the coronavirus S glycoprotein is located outside of the viral particle and mediates the viral entry into the host epithelial cells, it is undoubtedly the main target of neutralizing antibodies (NAbs) upon infection, making it the most important therapeutic target and essential in vaccine design.

Given the importance of coronaviral S glycoprotein in vaccine development, this study aimed to screen and identify the evolutionarily conserved sequences in animal and human coronaviral S glycoproteins. The amino acid sequence of SARS-CoV-2-Wuhan-Hu-1 S glycoprotein served as the reference sequence for all retrieved coronavirus sequences. Upon screening and alignment, the results revealed that bat coronavirus strains, such as Bat CoV RATG13, Bat CoV ZXC21, and Bat CoV YN02, and pangolin coronavirus strains, such as Pangolin CoV GX-P2V, Pangolin CoV GX-P5E, Pangolin CoV GX-P5L, Pangolin CoV GX-P1E, Pangolin CoV GX-P4L, and Pangolin CoV MP789, showed some level of evolutionary divergence compared to that of SARS-CoV-2-Wuhan-Hu-1 with an identity threshold above 80% (Supplementary Figure S1). These results also explain why bats or pangolins are deduced as the most likely reservoirs of SARS-CoV-2. In contrast, three selected avian coronaviral S glycoproteins were distantly related to the reference sequence due to their relatively high evolutionary divergence.

The amino acid sequences of SARS-CoV-2 and hCoVs, causing the common cold, were also aligned with the reference sequence (Supplementary Figure S2). The S1 regions of hCoVs, i.e., H-CoV-HKU1–genotype B, CoV-OC43, CoV-NL63, and CoV-229E, showed insignificant similarities to the reference sequence. Interestingly, their S2 regions were relatively conserved, particularly at residues S815–S874, S897–S934, S944–S1069, and S1207–S1218. The residues S897–S1069 corresponded to the HR1 and HR2 regions of the S2 subunit, whereas the S1207–S1218 region was part of the HR2 and TM domain. The conservation of the HR1 and HR2 regions was documented previously and suggested as the targets for the development of fusion inhibitor agents [35,36]. Furthermore, the alignment of S glycoprotein sequences of SARS-CoV-2 and its variants, MERS-CoV and SARS-CoV, revealed that SARS-CoV, SARS-CoV-2 and its variants were highly similar to each other (Supplementary Figure S3). This finding suggests that the emergence of SARS-CoV and SARS-CoV-2 might be due to the recombination of viral genomes between bat coronaviruses in their natural reservoir (bats) or the intermediate host (pangolin), or both. There were no observable evolutionarily conserved regions in the S glycoprotein sequence of MERS-CoV relative to that of SARS-CoV. Altogether, the alignment of coronaviral S glycoproteins with the reference sequence revealed a high evolutionary relationship between SARS-CoV (Urbani), bat CoVs, and pangolin CoVs. It is suggested that the emergence of the highly contagious and pandemic-causing SARS-CoV-2 is highly attributable to genome recombination or mutations of the coronavirus in animal hosts such as bats [37,38,39]. The high evolutionary relationship among coronaviruses sheds light on the development of universal vaccines using conserved epitopes.

### 3.2. Prediction and Screening of Conserved CTL Epitopes of S Glycoprotein

The conserved CTL epitopes of S glycoprotein were first screened and predicted based on 30 coronaviral S glycoprotein sequences. The NetCTL-1.2 of DTU Health Tech provides high sensitivity and specificity among the publicly available bioinformatics tools [40,41]. This web-based bioinformatics tool utilizes a combination of predictive algorithms including proteasomal cleavage, TAP transport efficiency, and MHC class I affinity to acquire highly probable CTL epitopes in a given sequence. Given the easy accessibility, the epitopes were selected based on the available human leucocyte antigen (HLA) class I supertypes provided by the algorithms, such as A1, A2, A3, A24, A26, B7, B8, B27, B39, B44, B58, and B62 supertypes.

HLA class I is known to be responsible for presenting processed antigens to T-cell receptors. Generally, there are three classical HLA class I encoding genes (HLA-A, HLA-B, and HLA-C) and all of them are extremely polymorphic. The number of identified HLA alleles has grown exponentially over the past decades and is likely to increase with time. To date, there are over 36,000 sequences of highly curated HLA alleles deposited in the IPD-IMGT/HLA Database (https://www.ebi.ac.uk/ipd/imgt/hla/ (accessed on 14 August 2024)). Undoubtedly, the vast number of HLA alleles makes the epitope prediction significantly complex and impractical. Thus, in the mid-1990s, an allele-specific classification called HLA supertype was created, in which the first nine HLA class I supertypes were described [42] and three more HLA class I supertypes were added later. Hence, there are 12 HLA class I supertypes in the latest update [43]. dos Santos Francisco et al. (2015) investigated HLA class I supertype frequencies among 55 human populations and found that HLA supertypes A2, A3, B7, B27, and B44 were evenly distributed and not specific to only certain populations [37]. Half of the populations showed frequencies at 14–29% for A2, 14–32% for A3, 18–31% for B7, and 21–32% for B44. In contrast, HLA supertypes A1, A24, B58, and B62 had greater frequency variations among the studied populations. It is also worth mentioning that the A24 supertype was found at higher frequencies (40% on average) in SEA, PAC, AUS, NEA and AME; meanwhile, the A1 supertype had an average frequency of 21% in Africa, Europe, and Southwest Asia [44].

The prediction of conserved CTL epitopes was based on the HLA class I supertypes to cover as many human populations as possible. The initial screening yielded 1,048,575 potential CTL epitopes that matched the 12 HLA class I supertypes. The large number of epitopes was then streamed down based on their antigenicity, immunogenicity, and toxigenicity. The elimination was performed using VaxiJen 2.0, IEDB MHC Class I immunogenicity and ToxinPred, respectively. The remaining 2114 epitopes were subjected to another round of screening based on their frequency of appearance in 30 coronavirus strains and 12 HLA class I supertypes. After stringent selection and removal of redundant epitopes, 12 epitopes (Table 7) were chosen for further analysis during the epitope’s alignment step.

### 3.3. Prediction and Screening of Conserved HTL Epitopes

The IEDB MHC-II prediction tool was used to predict and identify HTL epitopes because it provides a remarkable performance score owing to the embedded IEDB consensus 2.22 method [45,46]. Thirty coronavirus S glycoprotein sequences were mapped to 27 most widely distributed HLA class II alleles as described by Greenbaum et al. (2011) [47]. A total of 108,767 HTL epitopes were identified and selected based on their being in the top 20% of the consensus percentile rank, corresponding to their abilities to capture 50% of the total immune response [24].

The epitopes were subjected to antigenicity, IFN-inducing, and toxigenicity predictions. The total number of remaining peptides was 1377, which rendered difficulties in epitope selection. Consequently, peptides with 50% or greater matching with the HLA class II alleles and coronavirus strains were chosen. This is to ensure a wider HLA supertype coverage and provide a more comprehensive defense against multiple strains of coronaviruses. There were 52 epitopes retained (Supplementary Table S1), which were subsequently subjected to sequence alignment with that of SARS-CoV-2-Wuhan-Hu-1 to locate their positions. All of them were highly related to SARS-CoV-2 and its variants. Among them, three epitopes including HTL3, HTL6 and HTL26 were also related to SARS-CoV (Urbani).

### 3.4. Prediction and Screening of Conserved LBL Epitopes

**Figure 2:**
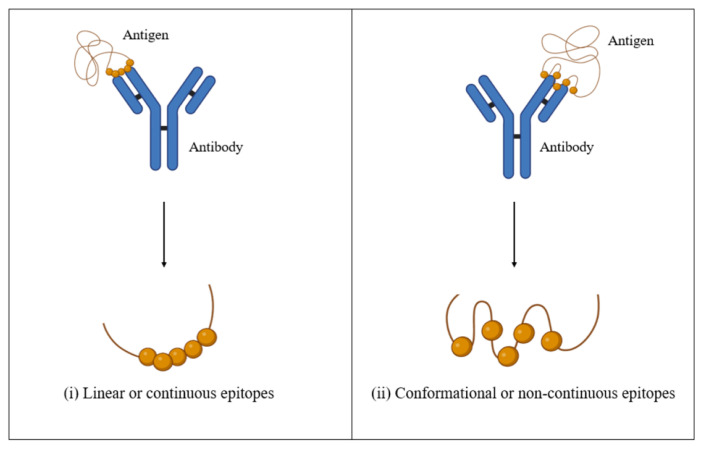
Schematic diagram of linear and conformational B-cell epitopes. Panels (**i**) linear or continuous B-cell epitopes composed of amino acid residues that are sequential to one another; (**ii**) conformational or non-continuous B-cell epitopes composed of amino acids that are non-sequential and scattered along the peptide sequence.

The identified conserved LBL epitopes represented potential antigen candidates for stimulating the humoral immune response. Generally, B-cell epitopes are divided into (i) linear and continuous or (ii) conformational and non-continuous (Figure 2). Although the vast majority of B-cell epitopes are conformational (approximately 90%) [48,49], the prediction of conformational B-cell epitopes is not as established as the LBL epitopes. Thus, the LBL epitope prediction has gained the most attention, especially in epitope-based vaccine development.

In contrast to the conserved HTL and CTL epitope predictions, screening and prediction of LBL are exclusive of HLA class I and II alleles. This is because B cells recognize antigens via B-cell receptors (BCR), known as membrane-bound immunoglobulins (Ig). Immunoglobulins consist of a constant fragment (Fc) region at the stalk and a variable (V) domain at the top. Given its functions in antigen binding, the V domain is responsible for the enormous theoretical diversity (10^13–15^) of the BCR repertoire [50,51]. Despite the high plasticity and diversity of the BCR repertoire, several lines of evidence demonstrated high frequencies of shared BCR clonotypes or elements in human BCR [52,53].

On this account, the conserved LBL epitopes were screened and identified using the ABCPred and SVMTriP prediction tools. These tools are considerably accurate in their predictions [54,55] and the results are analyzable. A total of 4238 peptides with thresholds of 0.5 and greater were obtained after eliminating the duplicated sequences. The antigenicity and toxicity predictions were performed as described earlier to exclude non-antigenic and toxigenic epitopes, leaving only 621 peptide sequences for further analysis. Subsequently, the number of epitopes was narrowed to 68 by retaining the peptides similar to these sequences or found in 50% or greater of the coronavirus strains (Table 8).

### 3.5. Alignment and Assembly of the Identified HTL, CTL T, and LBL Epitopes

In this study, a total of 131 epitopes (12 CTL epitopes, 52 HTL epitopes, and 68 LBL epitopes) were identified. Generally, the selection criteria included (i) within the S1 or S2 region, (ii) conserved regions, and (iii) matching most HLA class I and II supertypes. The identified epitopes were aligned to the SARS-CoV-2-Wuhan-Hu-1 S glycoprotein to identify their positions (Supplementary Figure S4). They were then assembled and combined into two peptide sequences encompassing 39 and 34 amino acid residues, respectively (Table 9). Interestingly, the sequences of both assemblies, i.e., Epi1 and Epi2, corresponded to the epitopes located within the S1 region of the S glycoprotein. Epi1 was located at the N-terminal domain (NTD) (S_256–294_) while Epi2 was found in the RBD (S_492–525_). In addition, they were relatively conserved among the pangolin and bat coronaviral S glycoproteins (Table 3) except for the avian coronavirus strains. Notably, Epi1 was 66.7% (26/39) and 43.6% (17/39) similar to that of SARS-CoV and MERS-CoV, respectively (Supplementary Figure S3), suggesting that the conservation of Epi1 renders it a promising candidate of a broad-spectrum, cross-protective vaccine that potentially offers prophylactic protection against multiple coronavirus strains.

Next, the allergenicity of Epi1 and Epi2 was determined by using AllergenFP v1.0. The results showed that Epi1 was a potential allergen whereas Epi2 was a non-allergen. Epi1 had the highest Tanimoto similarity index to a major allergen Pru av 1 (UniProtKB/Swiss-Prot ID: O24248) that causes birch pollinosis and oral allergy in patients allergic to cherry. Epi1 shared nine amino acids with that of the Pru av 1 peptide, albeit scattered throughout the latter’s peptide sequence (Supplementary Figure S5). Nonetheless, the overlapping residues in the Epi1 and Pru av 1 peptides do not coincide with the known IgE binding regions of the Pru av 1 peptide, i.e., the P-loop region (44LEGDGGPGT52) [56,57], suggesting that the allergenic potential of Epi1 is mostly negligible. Furthermore, due to its relatively smaller molecular size, the 3D structure and physiochemical properties of Epi1 are not definitive, thus it is inconclusive to serve as an allergen after modification for vaccine development.

### 3.6. Population Coverage

Table 10 summarizes the findings of the population coverage of HLA classes of Epi1 and Epi2. The total world population coverage analysis showed that Epi1 and Epi2 matched with 75.53% and 81.06% with HLA class I. In terms of HLA class II, Epi1 showed a coverage of 99.88% whilst it was 99.74% for Epi2. When HLA class I and II were combined, the coverage for both epitopes was notably high, at 99.97% for Epi1 and 99.95% for Epi2. These results suggest that Epi1 and Epi2 are potential lead peptides for vaccine development, targeting broad global coverage and addressing the challenges posed by mutations in coronaviruses.

### 3.7. Identification of the Locations of the Conserved Epitopes in Coronaviral S Glycoprotein

**Figure 3:**
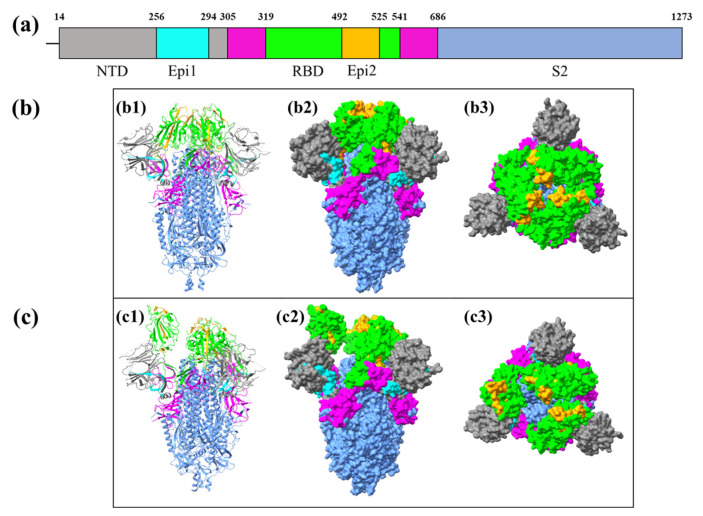
Locations of the Assembled Epitopes in the S Glycoprotein. (**a**) Schematic diagram of SARS-CoV-2 S glycoprotein with different colors representing the S1 subunit (S_14–685_) (magenta) and S2 subunit (S_686–1273_) (cornflower blue). (**b**) Ribbon and 3D structures of the closed state of SARS-CoV-2 S glycoprotein (PDB: 6VXX). (**b1**) The ribbon structure of closed state S glycoprotein. The locations of Epi1 and Epi2 were in cyan and orange colors, respectively. (**b2**) The orthogonal view of the closed state of S glycoprotein with Epi1 (cyan) and Epi2 (orange). (**b3**) The top-down view of the closed state of S glycoprotein displaying Epi1 (cyan) and Epi2 (orange). (**c**) Ribbon and 3D structures of the open state of SARS-CoV-2 S glycoprotein (PBD: 6VYB); (**c1**) The ribbon structure of the open state S glycoprotein and the locations of Epi1 and Epi2 in cyan and orange colors, respectively. (**c2**) The orthogonal view of the open state of S glycoprotein displaying Epi1 (cyan) and Epi2 (orange). (**c3**) The top-down view of the open state of S glycoprotein with Epi1 and Epi2 in cyan and orange colors, respectively.

As mentioned previously, Epi1 and Epi2 are located at the NTD (S_256–294_) and RBD (S_492–525_), respectively (Figure 3a). Figure 3 indicates the closed state and open state positions of Epi1 and Epi2 on the S glycoprotein.

The position of epitopes on an antigen contributes to its antigenicity and immunogenicity. The findings showed that Epi1 (cyan) was slightly embedded within the NTD (gray) and, therefore, was relatively less exposed than Epi2 (orange) located within the RBD domain (S_319–541_). This justifies the conservation of Epi1. Nonetheless, the conservation of epitopes is not solely determined by the exposure to immune cells or antibodies; it also depends on the functional importance of the epitopes. Mutations in conserved epitopes possibly disrupt key processes, such as viral attachment, entry, and immune evasion, thereby compromising the viral infectivity and replication in host cells [58,59,60]. In this light, conserved epitopes are important to ensure the structural and functional integrity of viral particles.

### 3.8. Molecular Docking of the Assembled Epitopes to TLR2 and TLR4 Receptors

To mount a robust immune response, it is of utmost importance for a vaccine to interact with cell receptors. Molecular docking was therefore performed with Toll-like receptors, specifically TLR2 and TLR4, as these receptors are well recognized for their roles in interacting with viral structural proteins, which subsequently leads to inflammatory cytokine production [61,62].

#### 3.8.1. Docking of TLR2 with Epi1 and Epi2

**Figure 4:**
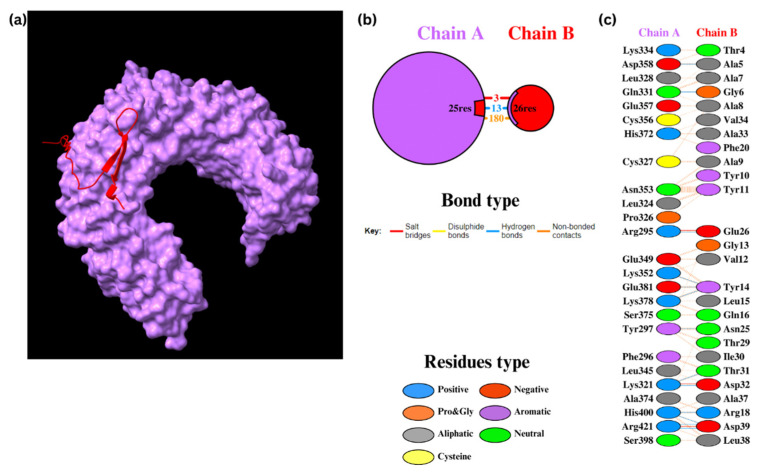
Docking of Epi1 with TLR2. (**a**) Epi1-TLR2 molecules. (**b**) The PDBsum results display the protein–protein interface between chain A (TLR2) and chain B (Epi1). (**c**) The PDBsum results display interacting amino acid residues in the interface.

The molecular docking analysis of Epi1 and Epi2 with TLR2 was performed using the HADDOCK 2.4 web server. For Epi1-TLR2, HADDOCK clustered 141 structures into 15 clusters, which represented 70% of the water-refined models (Figure 4). After refinement, 13 structures were clustered into one cluster resulting in 100% of the water-refined models. Epi1-TLR2 showed a strong binding affinity with a score of −76.6 ± 5.2, in which a negative score demonstrates a good binding between the docked molecules (Table 11). The interaction was visualized using UCSF ChimeraX 1.8 and the bonding was analyzed using PDBsum. The results showed three salt bridges, 13 hydrogen bonds, and 180 non-bonded contacts.

**Figure 5:**
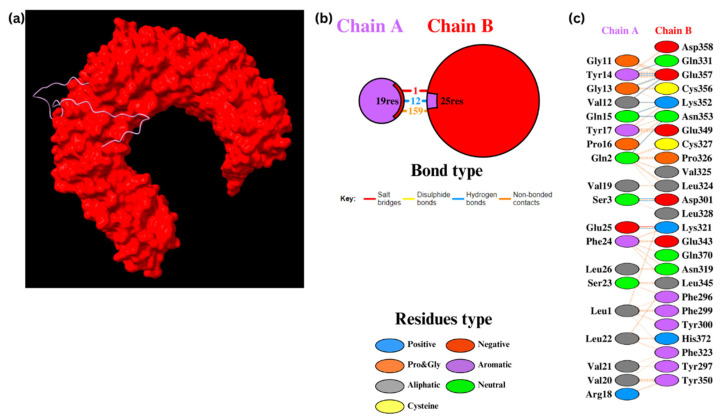
Docking of Epi2 with TLR2. (**a**) Epi2-TLR2 molecules. (**b**) The PDBsum results display the protein–protein interface between chain A (Epi2) and chain B (TLR2). (**c**) The PDBsum results demonstrate interactions between amino acid residues in the interface.

Similarly, for Epi2-TLR2, HADDOCK clustered 145 structures into 12 clusters, which represented 72% of the water-refined models (Figure 5). Upon refinement, 25 structures were clustered into one cluster resulting in 100% of the water-refined models. Epi2-TLR2 showed a good binding affinity with a HADDOCK score of −82.4 ± 6.5 (Table 11). There were one salt bridge, 12 hydrogen bonds, and 159 non-bonded contacts between the molecules.

The binding affinities were further confirmed using the PRODIGY web server, which calculated the Gibbs free energy (ΔG) and the dissociation constants (K_d_) (Supplementary Table S2). Epi1-TLR2 and Epi2-TLR2 contained ΔG of −14.8 kcal/mol and −13.0 kcal/mol, respectively. The K_d_ were 1.50 × 10^−11^ M for Epi1 and 2.80 × 10^−10^ M for Epi2 at 25 °C. The relatively low ΔG and K_d_ indicate strong, thermodynamically stable interactions.

#### 3.8.2. Docking of TLR4 with Epi1 and Epi2

**Figure 6:**
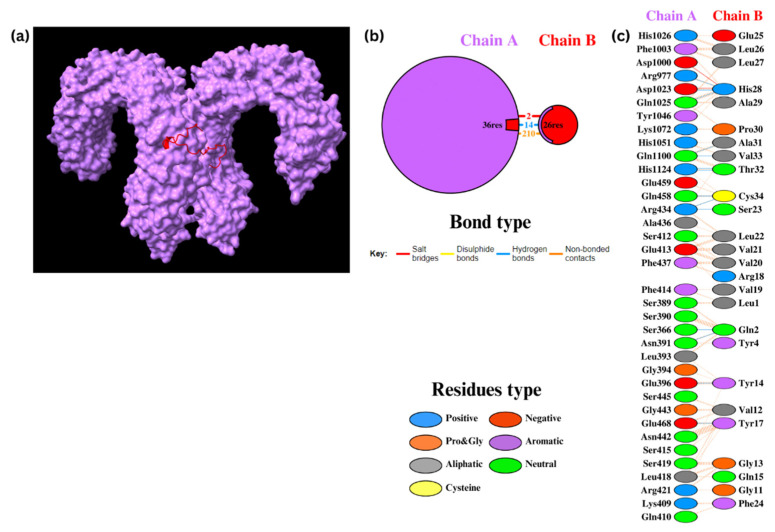
Docking of Epi1 to TLR4. (**a**) Epi1-TLR4 molecules. (**b**) The PDBsum results display the protein–protein interface between chain A (TLR4) and chain B (Epi1). (**c**) The PDBsum results display interactions between amino acid residues in the interface.

Epi1 and Epi2 were also docked to TLR4. For Epi1-TLR4, HADDOCK clustered 101 structures into 15 clusters, which represented 50% of the water-refined models (Figure 6). Subsequently, nine structures were refined and clustered into one cluster resulting in 100% of the water-refined models. The cluster had a HADDOCK score of 24.2 ± 19.4 (Table 12) with 10 hydrogen bonds and 213 non-bonded contacts. The ΔG was −17.1 kcal/mol with a K_d_ of 2.80 × 10^−13^ M (Supplementary Table S2).

**Figure 7:**
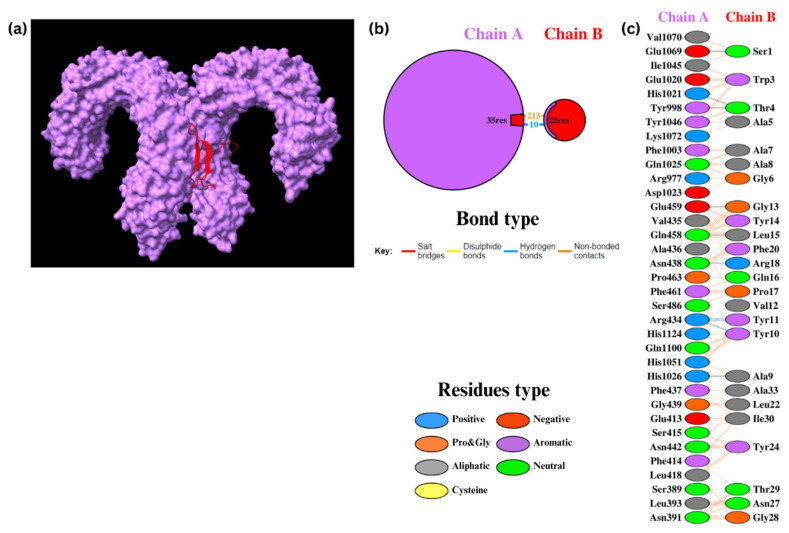
Docking of Epi2 to TLR4. (**a**) Epi2-TLR4 molecules. (**b**) The PDBsum results display the protein–protein interface between chain A (TLR4) and chain B (Epi2). (**c**) The PDBsum results display interactions between amino acid residues in the interface.

For Epi2-TLR4, 113 structures were clustered in 17 clusters, which represents 56% of the water-refined models (Figure 7). After refinement, 20 structures were clustered into one cluster resulting in 100% of the water-refined models. A HADDOCK score of 27.7 ± 4.6 (Table 12) was obtained with two salt bridges, 14 hydrogen bonds, and 210 non-bonded contacts. The ΔG was −18.8 kcal/mol with a K_d_ of 1.70 × 10^−14^ M (Supplementary Table S2), displaying a stronger binding affinity than that of Epi1. These results indicate stable, thermodynamically favorable interactions between the assembled peptides and TLR4.

### 3.9. Immune Simulation

**Figure 8:**
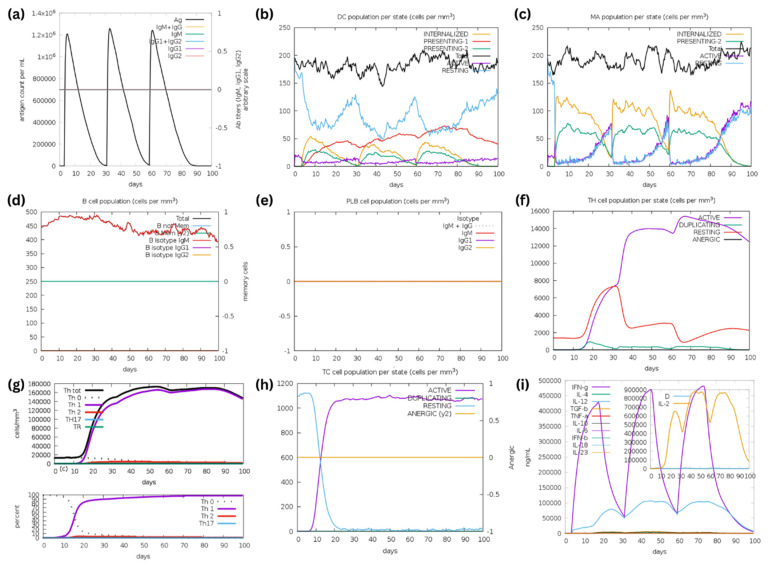
The simulation of immunostimulatory potential of Epi1 (three injections) using the C-IMMSIM server. (**a**) Three doses of Epi1 were injected over a period of 3 months. (**b**) Antigen presentation on MHC class I and II molecules of dendritic cells (DC). The curves indicate various states of DC: active, resting, internalized and antigen presentation. (**c**) States of macrophages: total count, internalized, antigen presentation on MHC class II, active, and resting. (**d**) B lymphocytes: total count, memory cells, and Ig-isotypes, i.e., IgM, IgG1 and IgG2. (**e**) Plasma B lymphocytes: count, Ig-isotype (IgM, IgG1 and IgG2). (**f**) Total CD4+ T-helper lymphocyte count and states, i.e., active, resting, anergic and duplicating. (**g**) Counts of CD4+ T-helper lymphocyte subtypes. (**h**) Total CD8+ T-cytotoxic lymphocyte count and state. (**i**) IFN-γ-, IL-12-, and IL-2-biased cytokine responses upon the peptide injections. D in the inset plot indicates the danger signal.

**Figure 9:**
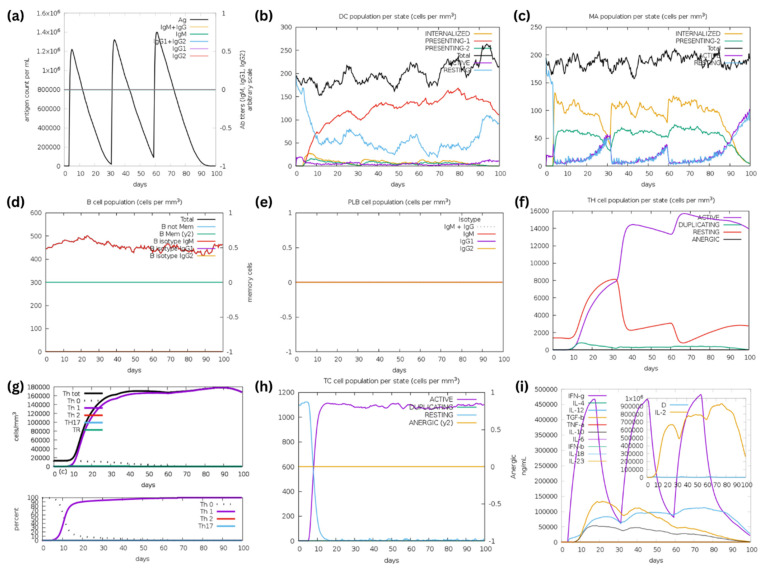
The simulation of immunostimulatory potential of Epi2 (three injections) using the C-IMMSIM server. (**a**) Three doses of Epi2 were injected over a period of 3 months. (**b**) Antigen presen-tation on MHC class I and II molecules of dendritic cells (DC). The curves indicate various states of DC: active, resting, internalized and antigen presentation. (**c**) States of macrophages: total count, internalized, antigen presentation on MHC class II, active, and resting. (**d**) B lymphocytes: total count, memory cells, and Ig-isotypes, i.e., IgM, IgG1 and IgG2. (**e**) Plasma B lymphocytes: count, Ig-isotype (IgM, IgG1 and IgG2). (**f**) Total CD4+ T-helper lymphocyte count and states, i.e., active, resting, anergic and duplicating. (**g**) Counts of CD4+ T-helper lymphocyte subtypes. (**h**) Total CD8+ T-cytotoxic lymphocyte count and state. (**i**) IFN-γ-, IL-12-, and IL-2-biased cytokine responses upon the peptide injections. Some TGF-β and IL-10 responses were also observed. D in the inset plot indicates the danger signal.

An in silico immune simulation was conducted using the C-IMMSIM server [29] to evaluate the immunostimulatory profile of Epi1 and Epi2. The simulation results of Epi1 and Epi2 were remarkably similar. The antibody titers shown in Figure 8a and Figure 9a were zero, indicating lack of B cell responses. This aligns with Figure 8d,e and Figure 9d,e, where immunoglobulin (Ig) isotype switching was absent. Typically, in the humoral response, proliferation of B lymphocytes into plasma cells involves an Ig class-switch recombination, leading to the production of Ig isotypes such as IgG and IgA that are crucial for neutralization of antigens [63]. However, the presence of only IgM-producing B cells implies that Ig isotype-switching did not occur as expected, particularly due to the lack of IL-4 and IL-21 (Figure 8i and Figure 9i) [63]. A strong bias toward a cell-mediated immune response was, on the other hand, observed in the simulation (Figure 8f–h and Figure 9f–h).

The antigen uptake and presentation of dendritic cells (DCs) and macrophages are shown in Figure 8b,c and Figure 9b,c. In terms of Epi1 uptake and presentation on DCs, the antigen was preferably displayed on MHC class II to MHC class I. Interestingly, the presentation of Epi2 on DCs was predominantly mediated by MHC class I. Macrophages presented the epitopes mainly through MHC class II molecules.

The hypothesis of Epi1 and Epi2 preferably stimulating cell-mediated immunity is supported by the findings in Figure 8f,g and Figure 9f,g. The results implied a rapid and robust CD4+ T-helper cell response after the first immunization, with the T-helper cell counts exceeding 15,000 cells/mm^3^ following the third injection. Notably, there was a clear bias toward Th1 differentiation (Figure 8g and Figure 9g). This is in line with the findings of Figure 8h and Figure 9h. Under the influence of IFN-γ, IL-12 and IL-2 secreted by Th1, cytotoxic T (Tc) cells are stimulated and continue to proliferate into active Tc that are responsible for getting rid of infected cells in natural infections.

According to Figure 8i and Figure 9i, the cytokine profile showed elevated IL-2, IFN-γ, and IL-12 synthesis following the Epi1 and Epi2 vaccinations, indicating T-cell proliferation and expansion. Interestingly, the anti-inflammatory cytokines, i.e., TGF-β and IL-10, were also observed at lower levels upon Epi2 immunization (Figure 9i), peaking at around 14,000 ng/mL and 5000 ng/mL, respectively. TGF-β and IL-10 are known to be associated with the Th2 response with relatively lower TGF-β and IL-10 synthesis, supporting the notion that Epi1 and Epi2 preferably mount Th1-biased immunity upon vaccination.

All in all, Epi1 and Epi2 elicited a predominantly cell-mediated immune response, characterized by strong Th1 and Tc cell activation. This phenomenon is seconded by robust production of IL-2, IFN-γ, and IL-12.

## 4. Discussion

The 2019 SARS-CoV-2 pandemic revealed the unpreparedness of global healthcare systems to effectively respond to such a crisis, eventually leading to the breakdown of healthcare systems. The evolution and natural selection of coronaviruses are believed to contribute to the emergence of various VOCs with exceptional abilities to escape vaccine- and infection-induced immunity. The COVID-19 prophylactic vaccines are mainly based on the whole S glycoprotein subunit of SARS-CoV-2-Wuhan-1 due to its high antigenicity and immunogenicity [64,65,66,67,68,69,70]. However, the effectiveness of the COVID-19 vaccines is becoming less pronounced following the development of immune-evading variants due to the perpetual gene mutations [71,72,73,74]. In addition, the immune imprinting induced by immunization and previous infections also reduces the efficaciousness of the vaccines against newly emerged variants [75,76]. In this light, a conserved multi-epitope approach has been adopted to develop pre-emptive vaccines against highly mutable coronaviruses by targeting the critical functional viral antigens. This strategy not only induces broad and long-lasting immune responses but it also prevents the need for a constant review of vaccine formulations due to viral mutations. To achieve this, epitope identification and characterization are entailed to generate epitope maps depicting their antibody specificities in silico prior to rigorous in vitro and in vivo empirical investigations [13,14,15,16,20,21,22,23,77,78,79].

Phylogenetically related zoonotic coronaviruses, including distantly related avian coronaviruses, were included in this study to identify and analyze the conserved regions. A significant genetic divergence was observed between avian and human coronaviruses compared to SARS-CoV-2. This divergence is especially notable when comparing avian coronaviruses, which belong to the *Gammacoronavirus* genus, with human coronaviruses and SARS-CoV-2, which belong to the *Alphacoronavirus* and *Betacoronavirus* genera, respectively. This observation is consistent with the phylogenetic data reported by Gilbert and Tengs (2021) [80]. It is noteworthy that none of the avian coronaviruses have been reported to infect humans to date. In this light, the prediction of conserved epitopes of coronaviruses prioritizes those and their close zoonotic counterparts causing diseases in humans (hCoVs, MERS-CoV, SARS-CoV, and SARS-CoV-2).

Initially, 12 HTL epitopes, 52 CTL epitopes, and 68 LBL epitopes were identified and the majority of them were conserved across the coronavirus strains. The avian coronaviruses, hCoVs, and MERS-CoV were distantly related to SARS-CoV, SARS-CoV-2, bat CoVs, and pangolin CoVs. The evolutionary convergence among those coronaviruses is likely due to the different natural and/or intermediate hosts [81]. In addition, it is noteworthy that the evolutionary convergence also results in the host-cell receptor variations as observed in hCoVs, among which the surface receptors responsible for viral adsorption are mainly surface peptidases and sialic acid-rich glycan-based receptors [82].

Human leukocyte antigen (HLA) alleles are among the most gene-dense and polymorphic regions in the human genome [83]. HLA molecules are responsible for antigen presentation to T-cell receptors (TcR) on CTL and HTL, and therefore can readily affect the vaccine-induced immune response [41,84,85,86,87,88,89,90,91,92]. In this light, it is important to retain antigenic epitopes that can interact and bind to HLA class I and class II molecules in vaccine design and development. Epi1 and Epi2 fulfill the characteristics of immunogenic vaccine candidates, given their multiple T-cell and B-cell epitopes. The total world population coverage analysis revealed a remarkably high class I coverage of above 75% and 81% for Epi1 and Epi2, respectively, whilst the class II coverage was greater than 99% for both Epi1 and Epi2. These results suggest the promising potential of these peptides as vaccine candidates with broad global MHC coverage, addressing the challenges posed by constant genome mutations in coronaviruses. Among the matched HLA class I supertypes, the A*02 supertype is prevalently found in almost all human populations [93]. In regard to the HLA class II supertypes, more than 20 HLA class II supertypes were identified. Together with the LBL epitopes, Epi1 and Epi2 are expected to trigger cellular and humoral immune responses in vivo, thereby providing a more comprehensive protection against coronavirus infection [49,94].

Many of the identified epitope sequences overlapped one another; therefore, they were aligned and assembled into single peptide sequences. Two peptide assemblies, Epi1 and Epi2, consisting of HTL, CTL, and LBL epitopes, represented residues S256–294 (SGWTAGAAAYYVGYLQPRTFLLKYNENGTITDAVDCALD) and S492–525 (LQSYGFQPTNGVGYQPYRVVVLSFELLHAPATVC) of the S glycoprotein, respectively. Epi1 is located in the S1 region, particularly the NTD; Epi2, on the other hand, is found in the RBD region. Essentially, the S glycoprotein of SARS-CoV-2, one of the structural components of the virus, was reported to interact with TLR4 and potentially TLR2, thus leading to inflammatory responses [95,96,97,98,99]. In humans, TLR4 are predominantly expressed in cells of myeloid origin such as macrophages, immature dendritic cells, monocytes, and granulocytes [100]. TLR4 signaling is initiated when the receptor binds to a ligand, leading to homodimerization and recruitment of Toll/interleukin-1 receptor-like (TIR)-domain-containing adapter molecules. This activates two major pathways: (i) the Myeloid differentiation primary response 8-dependent (MyD88-dependent) pathway, responsible for early nuclear factor kappa-β (NF-κβ) activation and pro-inflammatory cytokine release, and (ii) the TIR domain-containing adaptor inducing IFN-β-TRIF-related adaptor molecule pathway (TRIF-TRAM pathway), which induces type-I interferon production and TNF-α secretion for late NF-κβ activation [100]. On the other hand, human TLR2 is mainly expressed on myelomonocytic cell lines and specific blood cells with CD14+ monocytes showing the highest expression, followed by CD15+ granulocytes [101]. TLR2 signaling is initiated through ligand-induced dimerization with other TLRs, such as TLR1, TLR4, TLR6, and TLR10 [102,103,104,105], enabling the recognition of diverse microbial products and viral proteins [106,107,108,109]. This interaction subsequently activates MyD88-dependent signaling, leading to phosphorylation events involving interleukin-1 receptor-associated kinase (IRAK) proteins and receptor-associated factor 6 (TRAF6), which in turn activates NF-κB and mitogen-activated protein kinase (MAPK) pathways [110]. These pathways promote the production of pro-inflammatory cytokines and modulate cell proliferation and survival [111,112]. In this light, Epi1 and Epi2 were docked to TLR2 and TLR4 to estimate their interactions and binding strength. The docking results showed relatively good HADDOCK scores for both Epi1-TLR2 and Epi2-TLR2, indicating the potential of these peptides to activate the TLR-2-mediated innate immune response. Similarly, the HADDOCK scores of Epi1-TLR4 and Epi2-TLR4 were reasonable, implying the activation of TLR4 signaling upon binding to Epi1 and Epi2. These observations were further validated in the PRODIGY and PDBsum analyses, in which stable and favorable interactions were formed between the complexes. All in all, the standard HADDOCK 2.4 protocol effectively combines rigid docking with molecular dynamics simulations.

Epi1 and Epi2 were shown to predominantly elicit a cell-mediated immune response, characterized by strong Th1 and Tc cell activation with elevated levels of IL-2, IFN-γ, and IL-12 after in silico immunization. Notably, antigen presentation by macrophages and dendritic cells on the MHC class II molecules is responsible for stimulating CD4+ T-helper cells. Concomitantly, dendritic cells were also demonstrated to display epitopes of Epi2 on MHC class I molecules, thereby promoting the activation of cytotoxic T cells [113]. The activation of CD4+ T cells also results in the elevation of IL-2, which, in turn, promotes Th1 differentiation [114,115]. In addition, IL-12 secreted by dendritic cells and macrophages, also boosts Th1 differentiation for IFN-γ production [115,116], a key pro-inflammatory cytokine essential for orchestrating cell-mediated immunity [117]. Interestingly, the in silico simulation results coincided with several in vitro and in vivo reports. For instance, Meyer et al. (2023) reported that the S269–277 epitope, which is similar to that of Epi1, could induce a high magnitude of CTL immune response after in vitro stimulation [19]. An in vivo study confirmed that a peptide segment identical to that of Epi1 (S265–279 or YYVGYLQPRTFLLKY) could induce a robust antigen-specific IFN-γ-producing CTL response [17]; meanwhile, another in silico study identified the SGWTAGAAAYYV motif found in Epi1 as the immunodominant site for T-cell and humoral responses [12]. Collectively, Epi1 is a robust candidate for the development of multi-epitope vaccines. Although Epi2 (S492–525) and its immunostimulatory roles have not been reported elsewhere, its PYRVVVLSF motif was hypothesized to induce adaptive immunity [11].

Given the conservation of Epi1 and Epi2, they hold promise as lead antigens in universal multi-epitope vaccine development, particularly in fighting the upcoming mutants. This helps address issues concerning the constant gene mutations and immune evasion seen in coronaviruses. Epi2 consists of most of the important residues required to form tight binding with ACE2 receptors [118,119,120]. It also encompasses well-known mutation sites found in the currently circulating Omicron variant, i.e., N501 and Y505. The N501Y mutation can lower neutralizing antibody binding in vitro [121], while the Y505H mutation reduces viral protein stability, affects viral infectivity, and promotes immune evasion [122,123]. Given the importance of the mutations, including them in a vaccine formulation is likely to add to the relevance of the multi-epitope vaccine with the circulating coronavirus variants, hence greater immune protection.

Incorporating multiple epitopes in a vaccine formulation can offer broader and more durable protection against a wider range of viral variants. The multi-epitope sequences identified in this study shed light on the ongoing development and applications of coronavirus vaccines. The immune simulation analysis indicates that the medium length assembled peptides have limitations in stimulating humoral immune responses, as evidenced by the results (Figure 8 and Figure 9). This could be attributed to the C-IMMSIM web server’s restriction to MHC class II DRB alleles selection, having no alleles like DPA, DPB, DQA, or DQB in the MHC class II selection, which was found to be more prevalent in those assembled peptides. The interaction of MHC class II and peptides are crucial. The binding between peptides and the MHC molecules may promote humoral responses in vivo through both (i) direct mechanisms involving antigen presentation by APCs or (ii) indirect mechanisms where the interaction of the MHC receptor of CD4+ T cells interacts with B-cell MHC class II molecules, and/or (iii) through the release of cytokines by CD4+ T cells [63]. While stimulation of CD4+ and CD8+ T cells is likely to promote humoral responses in vivo, this needs to be validated through further in vivo studies. Additionally, in order to effectively deliver the assembled peptide and further improve its immunogenicity in vivo, the peptide can also be conjugated with virus-like particles (VLPs) or nanoparticle-based delivery systems to further enhance their abilities in inducing humoral and cellular responses [124,125]. Furthermore, the epitopes can be developed into multivalent vaccines consisting of promising flu antigens such as nucleoprotein (NP) of influenza A virus (IAV), which assembles into virus-like particles (VLP) for vaccine delivery [30,126]. To strengthen the multivalency of the vaccine, highly conserved matrix 2 ectodomain protein (M2e) of IAV can be added into the vaccine formulation. The IAV M2e is known for conferring partial protection in animal models against IAV [127,128]. Collectively, the aforementioned prospective applications highlight the versatility and the potential of these multi-epitope peptides in curbing coronavirus infections.

Although these predictive models allow us to screen epitopes quickly, they may not fully capture the complexities of antigen processing, epitope conformations, or interplays among immune cells and signaling pathways. This limited scope means in silico predictions might not fully correlate with the in vitro or in vivo immunogenicity and effectiveness of vaccine candidates. For instance, the C-IMMSIM predictions applied in this study may restrict certain MHC allele selections (e.g., DRB alleles), potentially affecting the overall humoral response prediction. Thus, in vitro and in vivo studies are warranted to further validate the potential of Epi1 and Epi2 as vaccine candidates. All in all, this study revealed evolutionarily conserved regions within SARS-CoV-2, SARS-CoV, and certain animal coronaviruses, while highlighting the genetic divergence observed in MERS-CoV and hCoVs as implied by their minimal sequence similarities in the S1 subunit.

## 5. Conclusions

In conclusion, evolutionarily conserved epitopes are present among animal and human coronaviral S glycoproteins. Overall, 132 candidates representing HTL, CTL and LBL epitopes with relatively low evolutionary divergence were identified. They were screened and filtered into two final peptide assemblies: Epi1 is composed of four HLA class I, five HLA class II, and three LBL epitopes; meanwhile, Epi2 consists of two HLA class I, eight HLA class II, and two LBL epitopes. Both peptides are located within the S1 subunit of the coronaviral S protein and demonstrate high population coverage and conservation. Additionally, they are expected to exhibit robust immunomodulatory roles, particularly cellular immunity via TLR2- and TLR4-mediated immune responses. Notably, Epi1 also contains immunodominant CTL epitopes, which adds to its potential as a vaccine candidate. Collectively, the conserved epitopes provide a robust foundation for universal vaccine development with extraordinary abilities to stimulate broad-spectrum immunity to mitigate the impact of coronaviral infections.

## Figures and Tables

**Table 1:** SARS-CoV-2 and its variants.

Nomenclature	Lineage	Accession Number
SARS-CoV-2-Wuhan-Hu-1 strain		NC_045512.2
Alpha	B.1.1.7	OK340744.1
Beta	B.1.351	OQ341818.1
Delta	B.1.617.2	OQ314763.1
Gamma	P.1	OQ316323.1
Omicron	B.1.1.529	OQ344199.1
Omicron	BA.1	OQ355083.1
Omicron	BA.1.1	OQ352636.1
Omicron	BA.2	OQ341824.1
Omicron	BA.2.12.1	OQ355080.1
Omicron	BA.2.75	OQ215893.1
Omicron	BA.2.75.2	OQ346937.1
Omicron	BA.4	OQ333888.1
Omicron	BA.4.6	OQ349323.1
Omicron	BA.5	OQ343976.1
Omicron	BA.5.2.6	OQ346806.1
Omicron	BF.11	OQ347094.1
Omicron	BF.7	OQ346784.1
Omicron	BN.1	OQ346744.1
Omicron	BQ.1	OQ346454.1
Omicron	BQ.1.1	OQ346605.1
Omicron	CH.1.1	OQ346876.1
Omicron	XBB	OQ347865.1
Omicron	XBB.1.5	XBB.1.5 is a sub-lineage of XBB with an additional spike RBD mutation S486P

**Table 2:** Sequences of hCoVs, SARS and MERS with their accession numbers.

Nomenclature	Accession Number
MERS-CoV	NC_019843
SARS-CoV (Urbani)	AY278741.1
HCoV-HKU1–genotype B	AY884001
HCoV-OC43	KF923903
HCoV-NL63	NC_005831

**Table 3:** Coronaviruses infecting bats, pangolins and birds.

Strain Name	Accession Number
Bat CoV RATG13	MN996532.2
Bat CoV ZXC21	MG772934.1
Bat CoV YN02	MW201982.1
Pangolin CoV GX-P2V	MT072864.1
Pangolin CoV GX-P5E	MT040336.1
Pangolin CoV GX-P5L	MT040335.1
Pangolin CoV GX-P1E	MT040334.1
Pangolin CoV GX-P4L	MT040333.1
Pangolin CoV MP789	MT121216.1
Avian CoV Ind-TN92-03	NC_048213.1
Avian CoV DK/GD/27/2014	NC_048214.1
Avian CoV MG10	NC_010800.1

**Table 4:** CTL prediction tools and their prediction criteria.

CTL Prediction Tools	Prediction Tool’s Criteria
NetCTL-1.2	Threshold: 0.75, 9-mers. Predict with all available supertypes (A1, A2, A24, A26, B7, B8, B27, B39, B44, B58, and B62). Select sequences with a combined score of above 0.75. Remove repetitive epitope(s) after prediction.
VaxiJen 2.0	Target Organism: Virus. Threshold: Default. Exclude “Non-antigenic” epitope(s).
IEDB MHC Class I immunogenicity	Masking position: Default. Exclude non-immunogenic epitopes. Prediction method: SVM (Swiss-Prot) based.
ToxinPred	Quantitative Matrix (QM) method: Blank. E-value cut-off for motif-based method: 10. SVM threshold: 0. Exclude “Toxin” epitope(s)

**Table 5:** Prediction flow and criteria of conserved HTL epitopes.

HTL Prediction Tools	Prediction Tool’s Criteria
IEDB MHC-II	Percentile rank: 20%, 15-mers. Method: Consensus 2.22. HLA Supertype: HLA-DR, HLA-DQ, HLA-DP. i. HLA-DR: DRB1*01:01 DRB1*07:01 DRB1*09:01 DRB3-01:01 DRB4*01:01 ii. HLA-DQ: DQA1*01:01/DQB1*05:01 DQA1*01:02/DQB1*06:02 DQA1*03:01/DQB1*03:02 DQA1*04:01/DQB1*04:02 DQA1*05:01/DQB1*02:01 DQA1*05:01/DQB1*03:01 iii. HLA-DP: DPA1*01/DPB1*04:01 DPA1*01:03/DPB1*02:01 DPA1*02:01/DPB1*01:01 DPA1*02:01/DPB1*05:01 DPA1*03:01/DPB1*04:02 Exclude epitope(s) with percentile rank higher than 20.0
IFNepitope	Prediction approach: Motif and SVM hybrid. Model for prediction: IFN-gamma versus non IFN-gamma. Exclude “NEGATIVE” epitope(s).

**Table 6:** Prediction flow and criteria of conserved LBL epitopes.

**LBL Prediction Tools**	**Prediction Tool’s Condition**
ABCPred	Length of epitope: 16-mers Threshold: 0.51 and above Overlapping filter: ON
SVMTriP	Length of epitope: 16-mers Select epitope(s) with a score of 0.5 and above

**Table 7:** Final selected CTL epitopes.

Epitopes	Number of Coronavirus Strains in Which the Epitope Is Found (Out of 30)	Location in the S Glycoprotein *	Assigned Name
RVVVLSFEL	25	509–517	CTL1
STQDLFLPF	24	50–59	CTL2
WTAGAAAYY	24	258–266	CTL3
YLQPRTFLL	24	269–277	CTL4
QIITTDNTF	24	1113–1121	CTL5
GAAAYYVGY	24	261–269	CTL6
ITDAVDCAL	24	284–293	CTL7
FTISVTTEI	24	718–726	CTL8
FVFLVLLPL	23	2–9	CTL9
QSYGFRPTY	15	493–501	CTL10
SVLYNFAPF	13	366–374	CTL11
YQPYRVVVL	6	505–513	CTL12

* Reference sequence: SARS-CoV-2-Wuhan-Hu-1 sequence.

**Table 8:** Final selected LBL epitopes.

Peptide Sequence	Number of Matched Coronavirus Strains	Location in S Glycoprotein	Assigned Name
CVLGQSKRVDFCGKGY	25	1045–1060	LBL1
DKYFKNHTSPDVDLGD	25	1166–1181	LBL2
DEDDSEPVLKGVKLHY	25	1270–1285	LBL3
AMQMAYFNGIGVTQN	25	899–914	LBL4
AGAALQIPFAMQMAYR	25	903–918	LBL5
FAMQMAYRFNGIGVTQ	25	911–926	LBL6
ASANLAATKMSECVLG	24	1033–1048	LBL7
ATKMSECVLGQSKRVD	24	1039–1054	LBL8
HGVVFLHVTYVPAQEK	24	1071–1086	LBL9
HVTYVPAQEKNFTTAP	24	1077–1092	LBL10
FVSGNCDVVIGIVNNT	24	1134–1149	LBL11
VIGIVNNTVYDPLQPE	24	1142–1157	LBL12
HTSPDVDLGDISGINA	24	1172–1187	LBL13
LGDISGINASVVNIQK	24	1179–1194	LBL14
GTTLDSKTQSLLIVNN	24	120–135	LBL15
ESLIDLQELGKYEQYI	24	1208–1223	LBL16
YVGYLQPRTFLLKYNE	24	279–294	LBL17
NENGTITDAVDCALDP	24	293–308	LBL18
AVDCALDPLSETKCTL	24	301–316	LBL19
DPLSETKCTLKSFTVE	24	307–322	LBL20
TVEKGIYQTSNFRVQP	24	320–335	LBL21
VQPTESIVRFPNITNL	24	333-348	LBL22
NDLCFTNVYADSFVIR	24	388–403	LBL23
PTKLNDLCFTNVYADS	24	397–412	LBL24
VVLSFELLHAPATVCG	24	524–539	LBL25
FRSSVLHSTQDLFLPF	24	56–71	LBL26
TDAVRDPQTLEILDIT	24	586–601	LBL27
EILDITPCSFGGVSVI	24	596–611	LBL28
GVSVITPGTNTSNQVA	24	607–622	LBL29
HSTQDLFLPFFSNVTW	24	62–77	LBL30
YSTGSNVFQTRAGCLI	24	649–664	LBL31
TISVTTEILPVSMTKT	24	732–747	LBL32
TECSNLLLQYGSFCTQ	24	760–775	LBL33
RALTGIAVEQDKNTQE	24	778–793	LBL34
AVEQDKNTQEVFAQVK	24	784–799	LBL35
EMIAQYTSALLAGTIT	24	881–896	LBL36
AGTITSGWTFGAGAAL	24	892–907	LBL37
IGKIQDSLSSTASALG	24	944–959	LBL38
FKCYGVSPTKLNDLCF	24	374–389	LBL39
FVTQRNFYEPQIITTD	23	1116–1131	LBL40
YEQYIKWPWYIWLGFI	23	1219–1234	LBL41
PWYIWLGFIAGLIAIV	23	1226–1241	LBL42
EPLVDLPIGINITRFQ	23	237–252	LBL43
QTLLALHRSYLTPGDS	23	239–254	LBL44
TRFQTLLALHRSYLTP	23	249–264	LBL45
NQVAVLYQGVNCTEVP	23	606–621	LBL46
YQGVNCTEVPVAIHAD	23	612–627	LBL47
NNSIAIPTNFTISVTT	23	722–737	LBL48
RDLICAQKFNGLTVLP	23	860–875	LBL49
VFLVLLPLVSSQCVNL	22	16–31	LBL50
TGTGVLTESNKKFLPF	22	560–575	LBL51
NNSYECDIPIGAGICA	22	670–685	LBL52
SQSIIAYTMSLGAENS	22	702–717	LBL53
YTMSLGAENSVAYSNN	22	708–723	LBL54
GDCLGDIAARDLICAQ	22	851–866	LBL55
DIPIGAGICASYQTQT	21	663–678	LBL56
PFLMDLEGKQGNFKNL	20	187–202	LBL57
GWTAGAAAYYVGYLQP	20	270–285	LBL58
HRSYLTPGDSSSGWTA	19	258–273	LBL59
YGVGHQPYRVVVLSFE	19	501–516	LBL60
SYQTQTKSHRRARSVA	19	673–688	LBL61
TASALGKLQDVVNHNA	19	941–956	LBL62
KQLSSKFGAISSVLND	19	964–979	LBL63
PVLPFNDGVYFASTEK	18	95–110	LBL64
PGQTGNIADYNYKLPD	17	412–427	LBL65
RKSNLKPFERDISTEI	17	470–485	LBL66
GSFCTQLKRALTGIAV	17	757–772	LBL67
LQSYGFRPTYGVGHQP	15	492–507	LBL68

**Table 9:** Final assembled epitopes.

Combination of Peptides	Peptide Sequence	Peptide Location *	Peptide Length	Matched HLA Class I Supertype	Matched HLA Class II Supertype	Assigned Name
CTL3+ CTL4+ CTL6+ CTL7+ HTL50+ HTL42+ HTL30+ HTL31+ HTL43+ LBL59+ LBL58+ LBL17	SGWTAGAAAYYVGYLQPRTFLLKYNENGTITDAVDCALD	256–294 (N-terminal domain)	39	A1, A2, A26, B8, B39, B58, B62	HLA-DPA1*01:03/DPB1*04:01; HLA-DPA1*01:03/DPB1*02:01; HLA-DPA1*02:01/DPB1*01:01; HLA-DPA1*02:01/DPB1*05:01; HLA-DPA1*03:01/DPB1*04:02; HLA-DQA1*01:01/DQB1*05:01; HLA-DQA1*01:02/DQB1*06:02; HLA-DQA1*04:01/DQB1*04:02; HLA-DQA1*05:01/DQB1*02:01; HLA-DQA1*05:01/DQB1*03:01; HLA-DRB1*01:01; HLA-DRB1*07:01; HLA-DRB1*09:01	Epi1
CTL1+ CTL10+ HTL51+ HTL25+ HTL14+ HTL22+ HTL23+ HTL15+ HTL16+ HTL45+ LBL60+ LBL68	LQSYGFQPTNGVGYQPYRVVVLSFELLHAPATVC	492–525 (RBD)	34	A1, A2, A3, B7, B27, B58, B62	HLA-DPA1*01:03/DPB1*04:01; HLA-DPA1*01:03/DPB1*02:01; HLA-DPA1*02:01/DPB1*01:01; HLA-DPA1*02:01/DPB1*05:01; HLA-DPA1*03:01/DPB1*04:02; HLA-DQA1*01:01/DQB1*05:01; HLA-DQA1*03:01/DQB1*03:02; HLA-DQA1*05:01/DQB1*02:01; HLA-DRB1*01:01; HLA-DRB1*07:01; HLA-DRB1*09:01; HLA-DRB4*01:01	Epi2

* Reference sequence: SARS-CoV-2-Wuhan-Hu-1 sequence.

**Table 10:** Analysis of population coverage of HLA classes of Epi1 and Epi2.

Class	Epi1			Epi2		
	Coverage	Average Hit	PC90 *	Coverage	Average Hit	PC90 *
Class I	75.53%	1.09	0.41	81.06%	1.25	0.53
Class II	99.88%	4.27	3.04	99.74%	3.82	2.53
Combined	99.97%	5.36	3.79	99.95%	5.07	3.48

* Minimum number of epitope hits/HLA combinations recognized by 90% of the population.

**Table 11:** HADDOCK 2.4 docking results for Epi1-TLR2 and Epi2-TLR2.

Scores	Epi1-TLR2	Epi2-TLR2
HADDOCK score	−76.6 ± 5.2	−82.4 ± 6.5
Cluster size	13	25
RMSD from the overall lowest-energy structure (Å)	4.9 ± 0.3	0.4 ± 0.3
Van der Waals energy (kcal mol^−1^)	−63 ± 4.5	−69.8 ± 8.8
Electrostatic energy (kcal mol^−1^)	−255.2 ± 32.3	−245.9 ± 51.1
Desolvation energy (kcal mol^−1^)	−21.6 ± 5.8	−37.3 ± 4.5
Restraints violation energy (kcal mol^−1^)	589.9 ± 65.3	738.6 ± 17.6
Buried surface area (Å^2^)	2292.3 ± 89.5	2271.0 ± 76.4
Z-Score	−1.5	−1.5

**Table 12:** HADDOCK 2.4 docking results for Epi1-TLR4 and Epi2-TLR4.

Scores	Epi1-TLR4	Epi2-TLR4
HADDOCK score	24.2 ± 19.4	27.7 ± 4.6
Cluster size	9	20
RMSD from the overall lowest energy structure (Å)	0.5 ± 0.3	8.4 ± 0.0
Van der Waals energy (kcal mol^−1^)	−92.7 ± 13.5	−101.1 ± 6.5
Electrostatic energy (kcal mol^−1^)	−133.2 ± 5.6	−125.3 ± 11.0
Desolvation energy (kcal mol^−1^)	−56.0 ± 4.1	−44.5 ± 1.0
Restraints violation energy (kcal mol^−1^)	1995.3 ± 111.3	1953.8 ± 46.4
Buried surface area (Å^2^)	2719.0 ± 164.8	2844.3 ± 109.4
Z-Score	−2.2	−1.8

## Data Availability

The data presented in this study are available on request from the corresponding author.

## Supplementary Materials

The following supporting information can be downloaded at: https://www.mdpi.com/article/10.3390/biomedicines12112530/s1, Table S1: Final selected HTL epitopes; Table S2: Complete PRODIGY analysis for all of the docked complexes; Figure S1: Animal coronaviruses alignment result with SARS-CoV-2-Wuhan-Hu-1 strain as the reference sequence; Figure S2: Human coronaviruses alignment result with SARS-CoV-2-Wuhan-Hu-1 strain as the reference sequence; Figure S3: Alignment result of SARS-CoV, MERS-CoV, and all SARS-CoV-2 variants with SARS-CoV-2-Wuhan-Hu-1 strain as the reference sequence; Figure S4: Alignment result of assembled epitopes, Sepi1 and Sepi2, to SARS-CoV-2-Wuhan-Hu-1 sequence; Figure S5: Alignment result of Sepi1 to Pru av 1 allergen peptide sequence.
