# Supplementary material for: An Immunoinformatic Approach for Identifying and Designing Conserved Multi-Epitope Vaccines for Coronaviruses
Source: Biomedicines. 2024 Nov 5;12(11):2530. doi: 10.3390/biomedicines12112530 (PMC11592158; doi:10.3390/biomedicines12112530)
Supplement: Supplementary file 1 [file biomedicines-12-02530-s001.zip › Supplementary Figure S1. Animal CoV alignment.pdf]

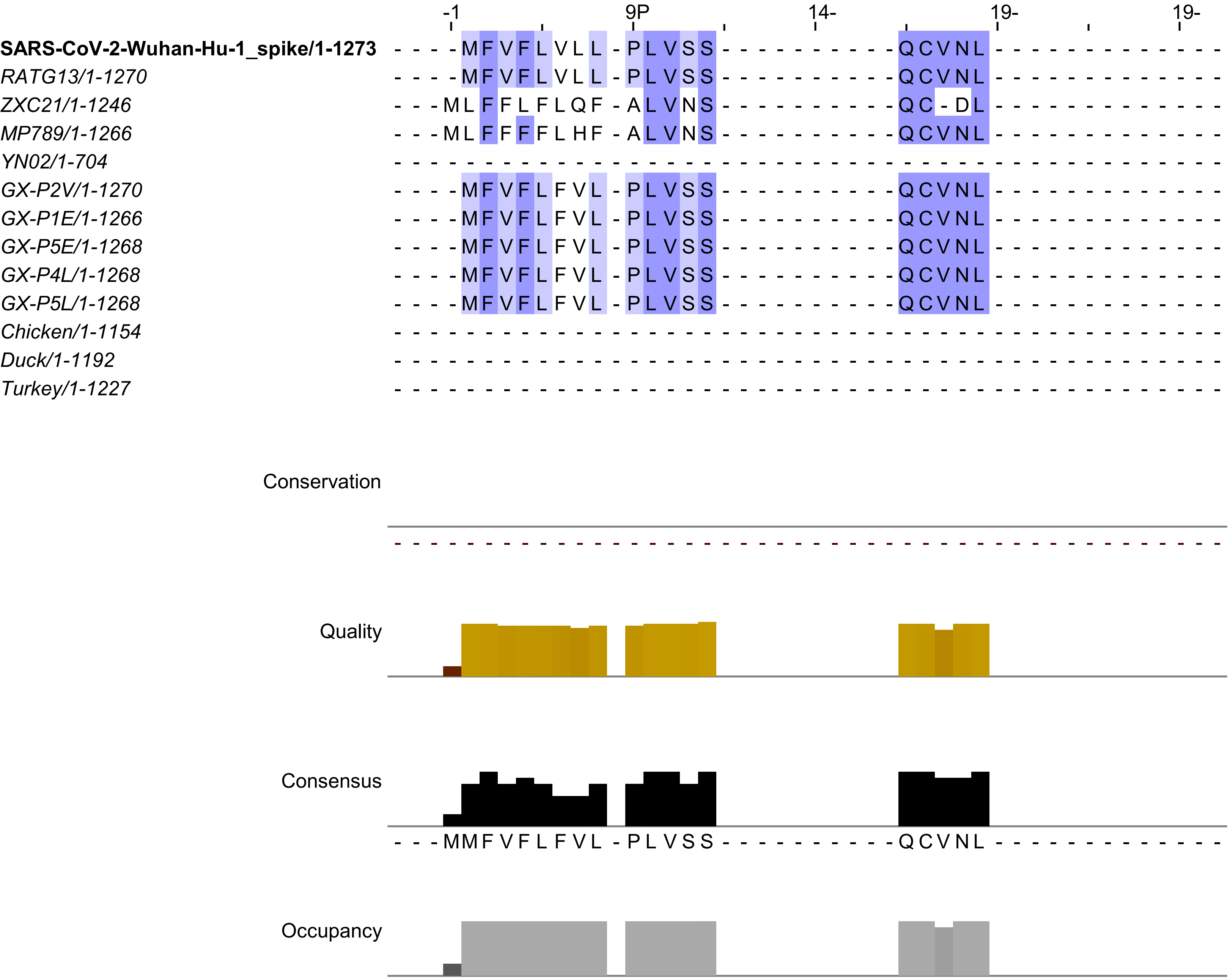

-1 9P 14- 19- 19-
SARS-CoV-2-Wuhan-Hu-1_spike/1-1273 ----MFVFLVLL-PLVSS---------QCVNL-------------
RATG13/1-1270 ----MFVFLVLL-PLVSS---------QCVNL-------------
ZXC21/1-1246 ---MLFFLFLQF-ALVNS---------QC-DL-------------
MP789/1-1266 ---MLFFFFLHF-ALVNS---------QCVNL-------------
YN02/1-704
GX-P2V/1-1270 ----MFVFLFVL-PLVSS---------QCVNL-------------
GX-P1E/1-1266 ----MFVFLFVL-PLVSS---------QCVNL-------------
GX-P5E/1-1268 ----MFVFLFVL-PLVSS---------QCVNL-------------
GX-P4L/1-1268 ----MFVFLFVL-PLVSS---------QCVNL-------------
GX-P5L/1-1268 ----MFVFLFVL-PLVSS---------QCVNL-------------
Chicken/1-1154
Duck/1-1192
Turkey/1-1227
Conservation
Quality
Consensus
---MMFVFLFVL-PLVSS---------QCVNL-------------
Occupancy

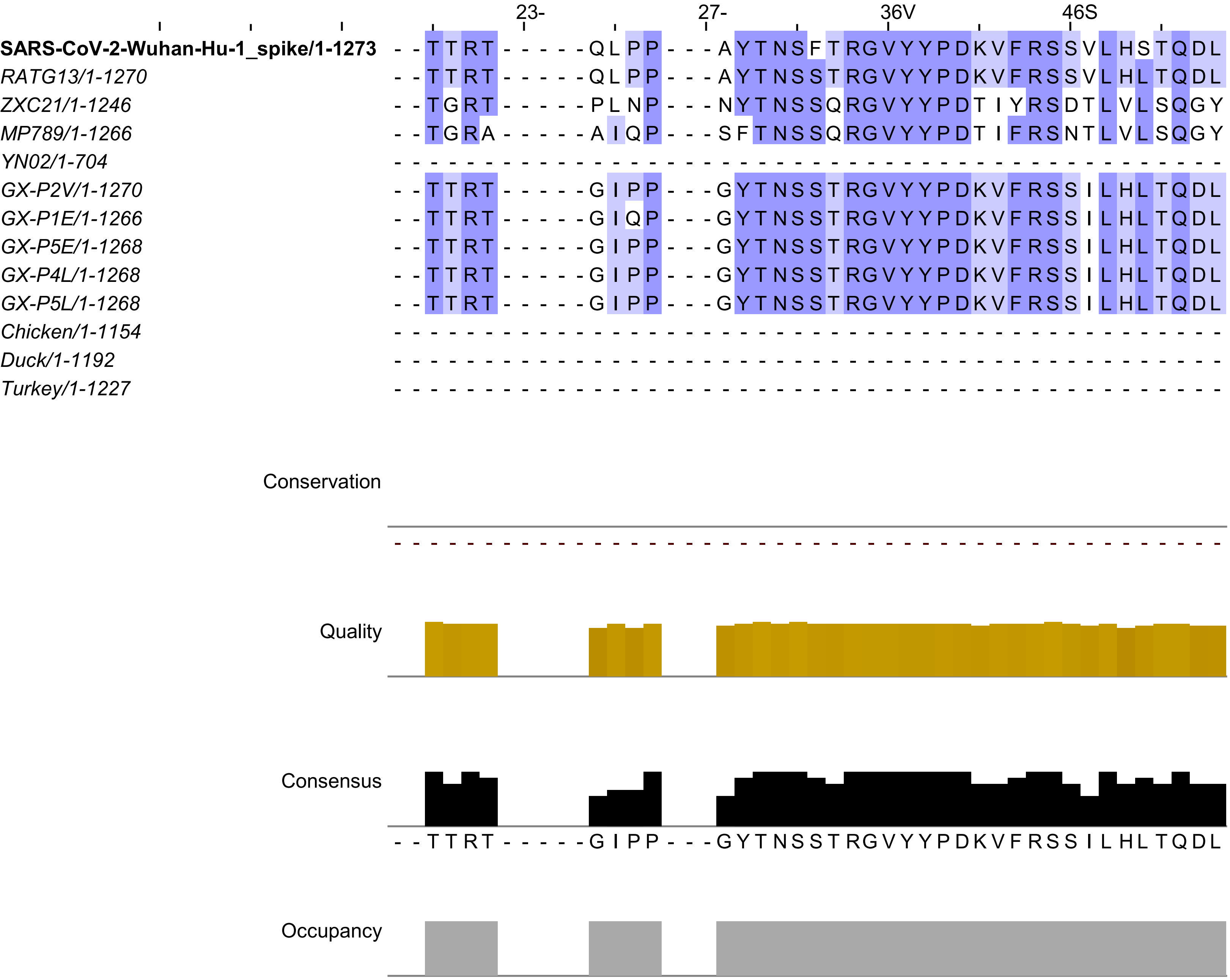
23-
27-
36V
46S
SARS-CoV-2-Wuhan-Hu-1_spike/1-1273 --TTRT-----QLPP---AYTNSFTRGVYYPDKVFRSSVLHSTQDL
RATG13/1-1270 --TTRT-----QLPP---AYTNSSTRGVYYPDKVFRSSVLHLTQDL
ZXC21/1-1246 --TGRT-----PLNP---NYTNSSQRGVYYPDTIYRSDTLVLSQGY
MP789/1-1266 --TGRA-----AIQP---SFTNSSQRGVYYPDTIFRSNTLVLSQGY
YN02/1-704
GX-P2V/1-1270 --TTRT-----GIPP---GYTNSSTRGVYYPDKVFRSSILHLTQDL
GX-P1E/1-1266 --TTRT-----GIQP---GYTNSSTRGVYYPDKVFRSSILHLTQDL
GX-P5E/1-1268 --TTRT-----GIPP---GYTNSSTRGVYYPDKVFRSSILHLTQDL
GX-P4L/1-1268 --TTRT-----GIPP---GYTNSSTRGVYYPDKVFRSSILHLTQDL
GX-P5L/1-1268 --TTRT-----GIPP---GYTNSSTRGVYYPDKVFRSSILHLTQDL
Chicken/1-1154
Duck/1-1192
Turkey/1-1227
Conservation
Quality
Consensus
--TTRT-----GIPP---GYTNSSTRGVYYPDKVFRSSILHLTQDL
Occupancy

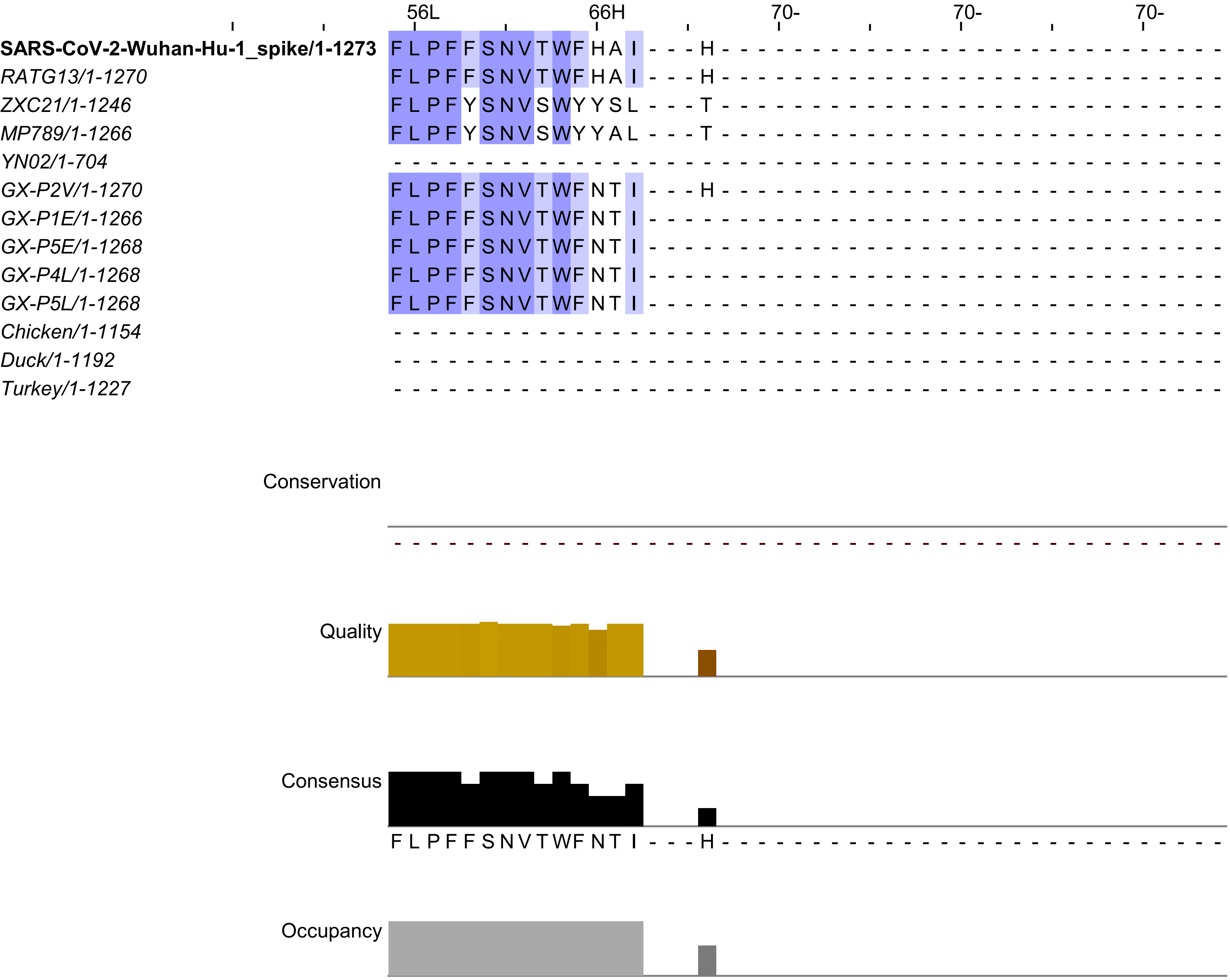
56L
66H
70-
70-
70-
SARS-CoV-2-Wuhan-Hu-1_spike/1-1273 FLPFFSNVTWFHAI---H------------------------
RATG13/1-1270 FLPFFSNVTWFHAI---H------------------------
ZXC21/1-1246 FLPFYSNVSWYYSL---T------------------------
MP789/1-1266 FLPFYSNVSWYYAL---T------------------------
YN02/1-704 ---
GX-P2V/1-1270 FLPFFSNVTWFNTI---H------------------------
GX-P1E/1-1266 FLPFFSNVTWFNTI-----------------------------
GX-P5E/1-1268 FLPFFSNVTWFNTI-----------------------------
GX-P4L/1-1268 FLPFFSNVTWFNTI-----------------------------
GX-P5L/1-1268 FLPFFSNVTWFNTI-----------------------------
Chicken/1-1154 ---
Duck/1-1192 ---
Turkey/1-1227 ---
Conservation
Quality
Consensus
FLPFFSNVTWFNTI---H------------------------
Occupancy

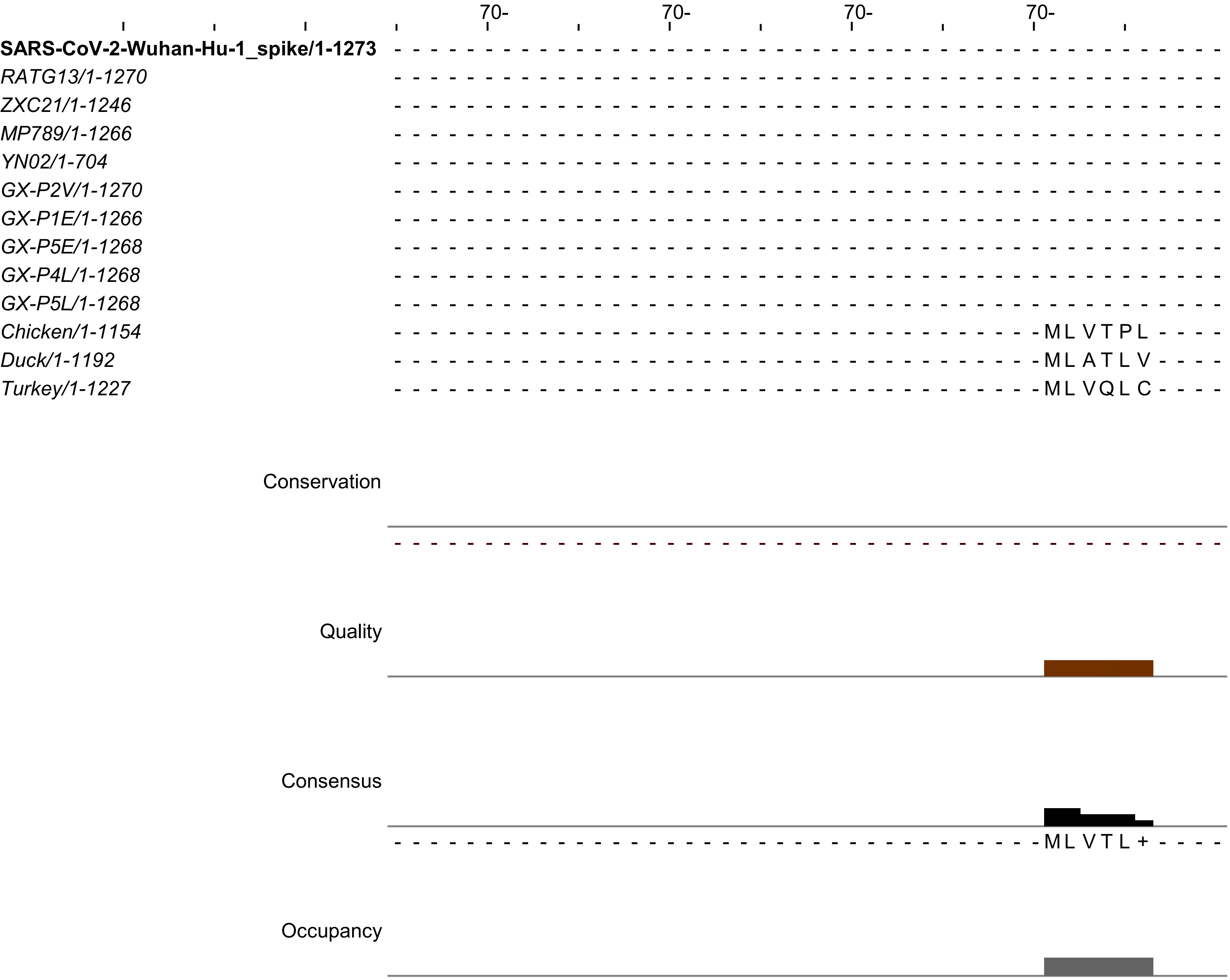
70-
70-
70-
70-
SARS-CoV-2-Wuhan-Hu-1_spike/1-1273
RATG13/1-1270
ZXC21/1-1246
MP789/1-1266
YN02/1-704
GX-P2V/1-1270
GX-P1E/1-1266
GX-P5E/1-1268
GX-P4L/1-1268
GX-P5L/1-1268
Chicken/1-1154 MLVTPL
Duck/1-1192 MLATLV
Turkey/1-1227 MLVQLC
Conservation
Quality
Consensus
MLVTL+
Occupancy

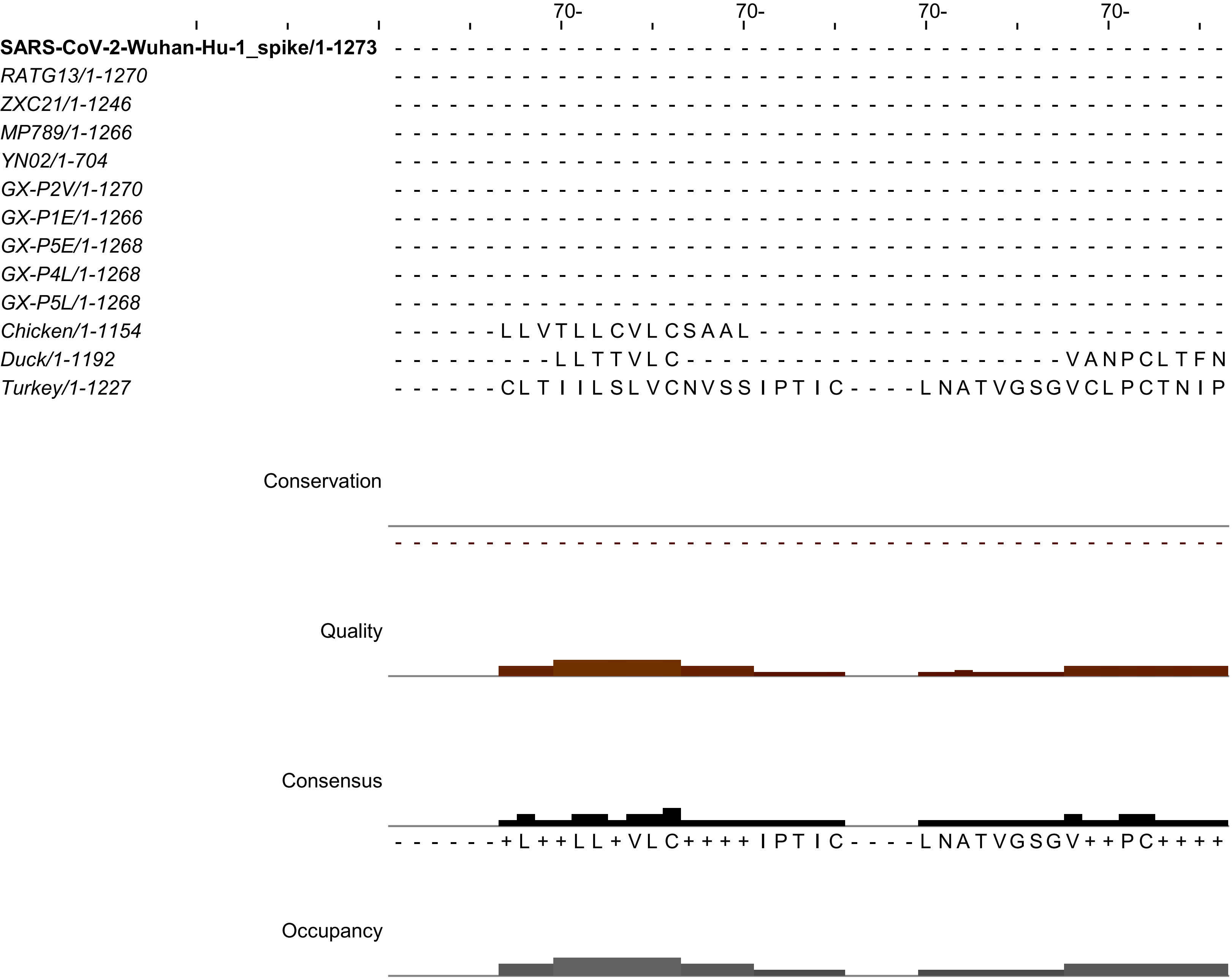
70-
70-
70-
70-
SARS-CoV-2-Wuhan-Hu-1_spike/1-1273
RATG13/1-1270
ZXC21/1-1246
MP789/1-1266
YN02/1-704
GX-P2V/1-1270
GX-P1E/1-1266
GX-P5E/1-1268
GX-P4L/1-1268
GX-P5L/1-1268
Chicken/1-1154 ------LLVTLLCVLCSAAL--------------------------
Duck/1-1192 ---------LLTTVLC------------------VANPCLTFN
Turkey/1-1227 ------CLTIILSLVCNVSSIPTIC----LNATVGSGVCLPCTNIP
Conservation
Quality
Consensus
------+L++LL+VLC++++IPTIC----LNATVGSGV++PC++++
Occupancy

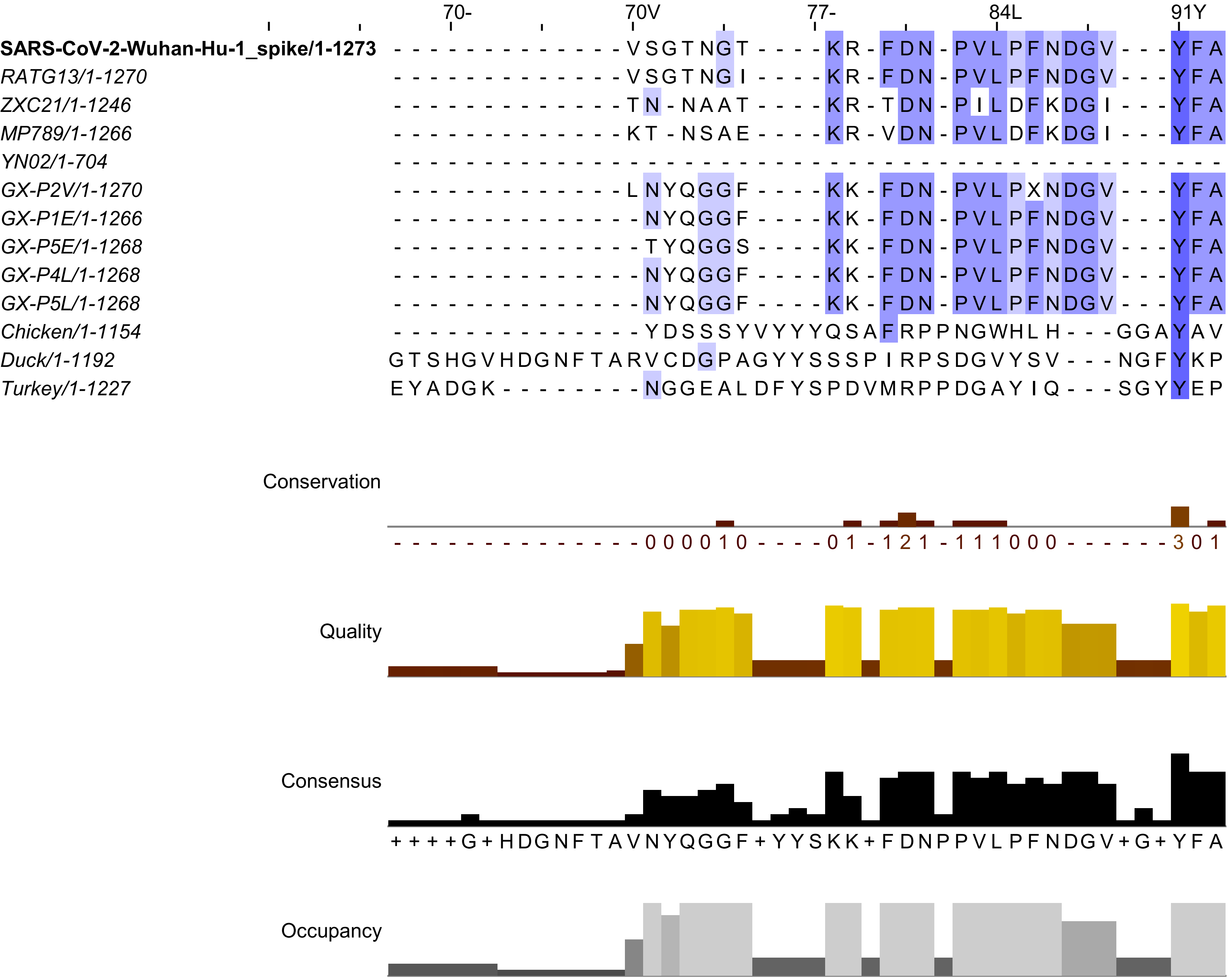

70-
70V
77-
84L
91Y
SARS-CoV-2-Wuhan-Hu-1_spike/1-1273
RATG13/1-1270
ZXC21/1-1246
MP789/1-1266
YN02/1-704
GX-P2V/1-1270
GX-P1E/1-1266
GX-P5E/1-1268
GX-P4L/1-1268
GX-P5L/1-1268
Chicken/1-1154
Duck/1-1192
Turkey/1-1227
Conservation
Quality
Consensus
Occupancy

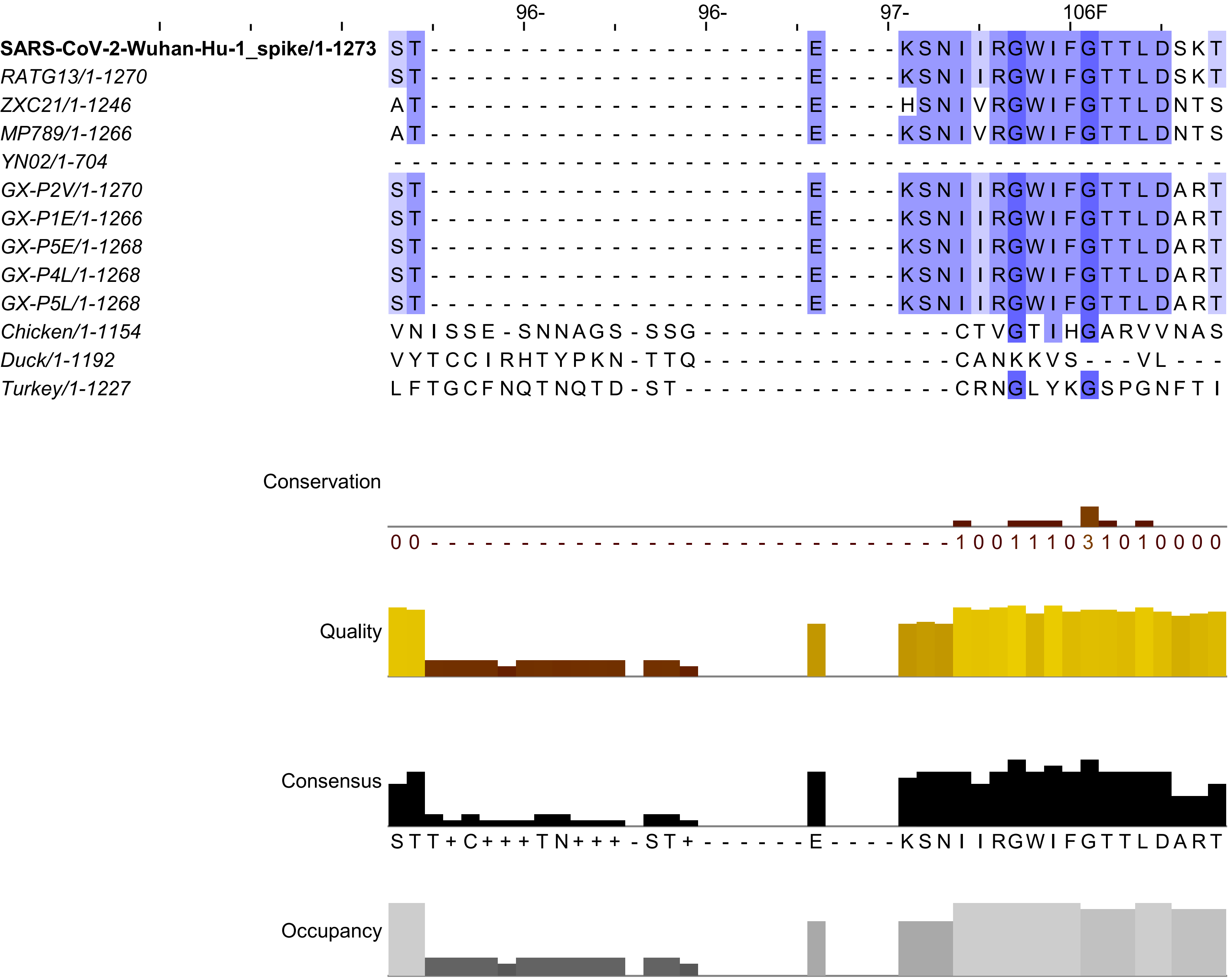

96-
96-
97-
106F
SARS-CoV-2-Wuhan-Hu-1_spike/1-1273
RATG13/1-1270
ZXC21/1-1246
MP789/1-1266
YN02/1-704
GX-P2V/1-1270
GX-P1E/1-1266
GX-P5E/1-1268
GX-P4L/1-1268
GX-P5L/1-1268
Chicken/1-1154
Duck/1-1192
Turkey/1-1227
Conservation
Quality
Consensus
Occupancy

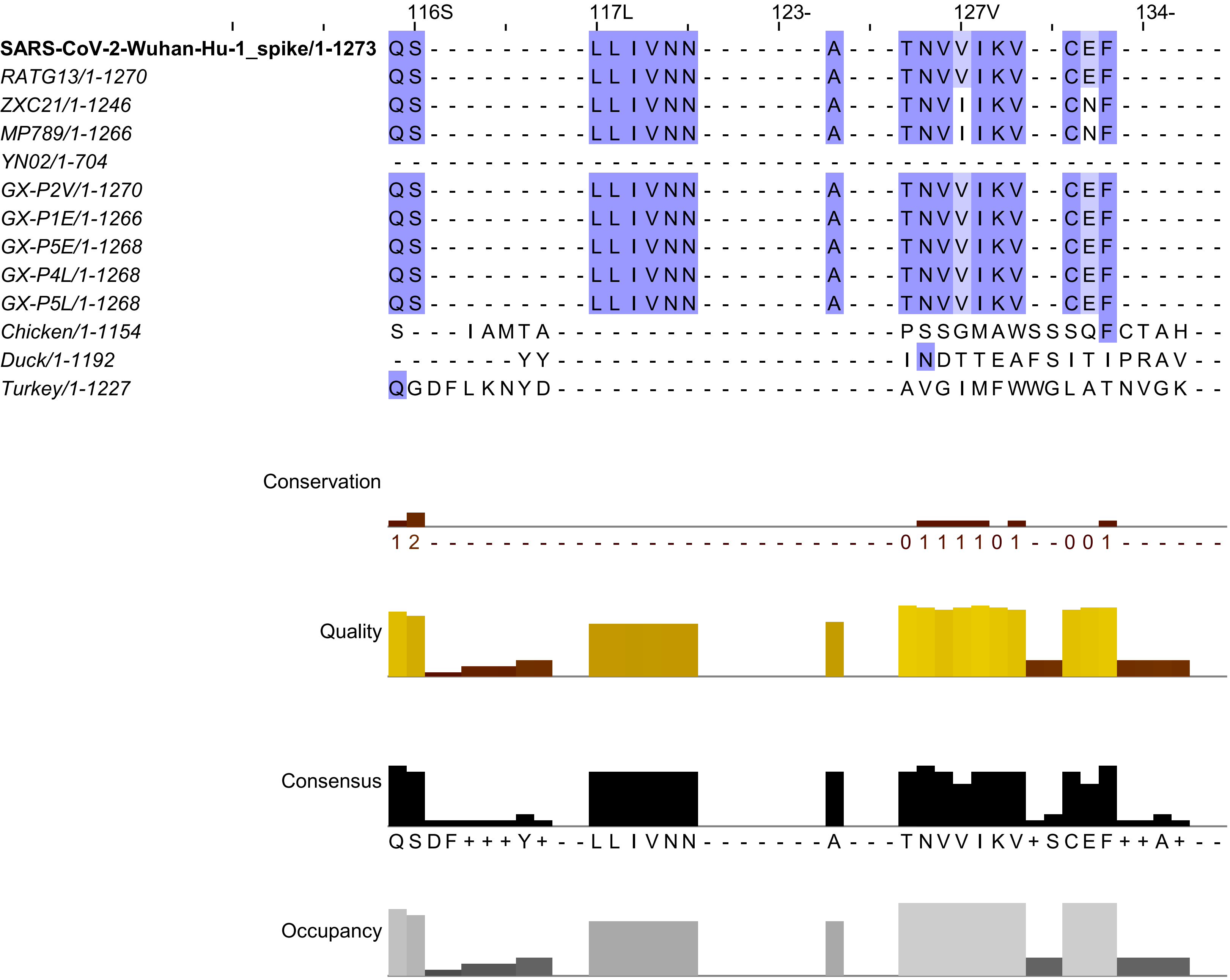
116S
117L
123-
127V
134-
SARS-CoV-2-Wuhan-Hu-1_spike/1-1273 QS---------LLIVNN-------A---TNVVIKV--CEF------
RATG13/1-1270 QS---------LLIVNN-------A---TNVVIKV--CEF------
ZXC21/1-1246 QS---------LLIVNN-------A---TNVIIKV--CNF------
MP789/1-1266 QS---------LLIVNN-------A---TNVIIKV--CNF------
YN02/1-704 --
GX-P2V/1-1270 QS---------LLIVNN-------A---TNVVIKV--CEF------
GX-P1E/1-1266 QS---------LLIVNN-------A---TNVVIKV--CEF------
GX-P5E/1-1268 QS---------LLIVNN-------A---TNVVIKV--CEF------
GX-P4L/1-1268 QS---------LLIVNN-------A---TNVVIKV--CEF------
GX-P5L/1-1268 QS---------LLIVNN-------A---TNVVIKV--CEF------
Chicken/1-1154 S---IAMTA-------------------PSSGMAWSSSQFCTAH--
Duck/1-1192 -------YY-------------------INDTTEAFSITIPRAV--
Turkey/1-1227 QGDFLKNYD-------------------AVGIMFWWGLATNVGK--
Conservation
12--------------------------0111101--001------
Quality
Consensus
QSDF+++Y+--LLIVNN-------A---TNVVIKV+SCEF++A+--
Occupancy

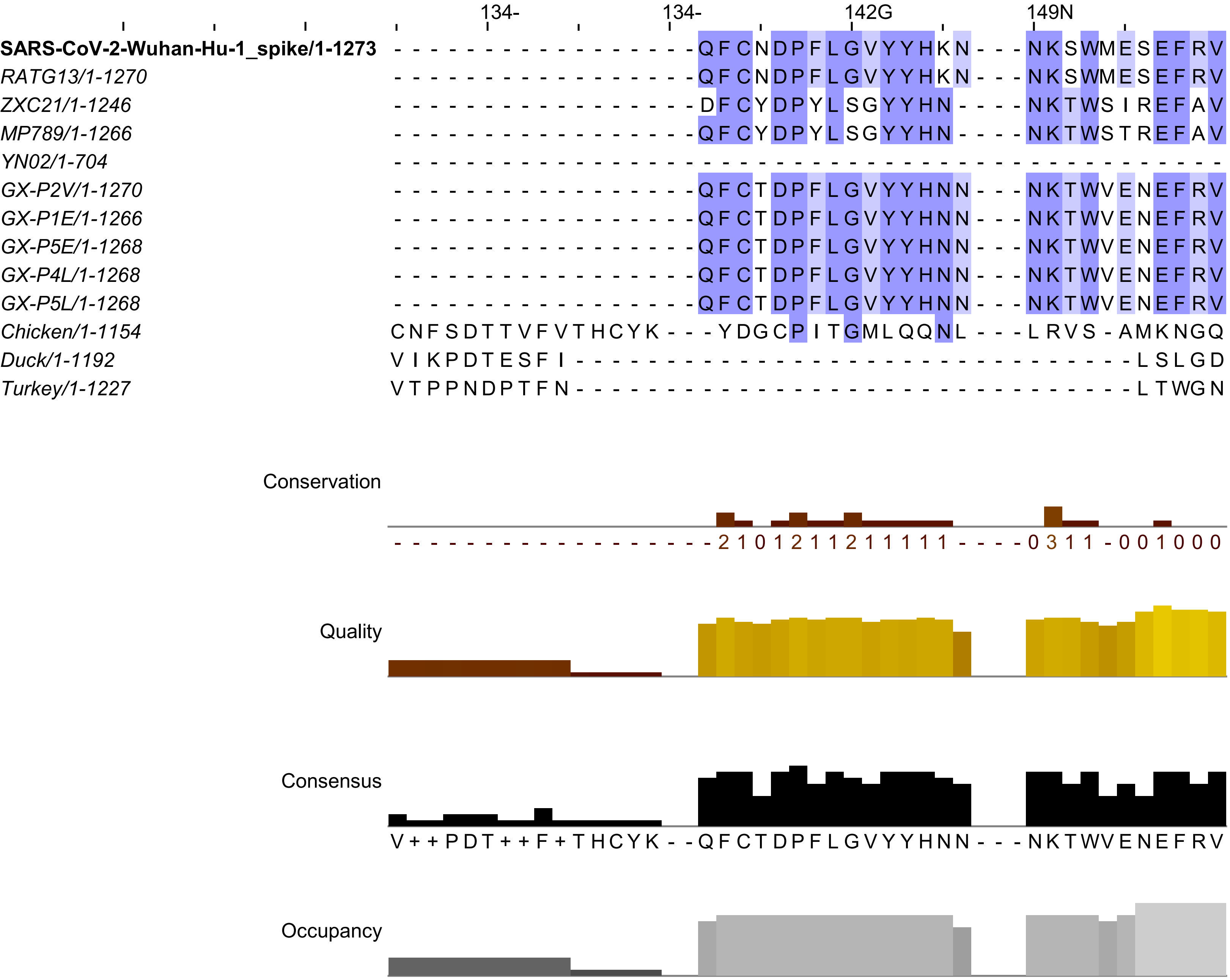
134-
134-
142G
149N
SARS-CoV-2-Wuhan-Hu-1_spike/1-1273
RATG13/1-1270
ZXC21/1-1246
MP789/1-1266
YN02/1-704
GX-P2V/1-1270
GX-P1E/1-1266
GX-P5E/1-1268
GX-P4L/1-1268
GX-P5L/1-1268
Chicken/1-1154
Duck/1-1192
Turkey/1-1227
Conservation
Quality
Consensus
Occupancy

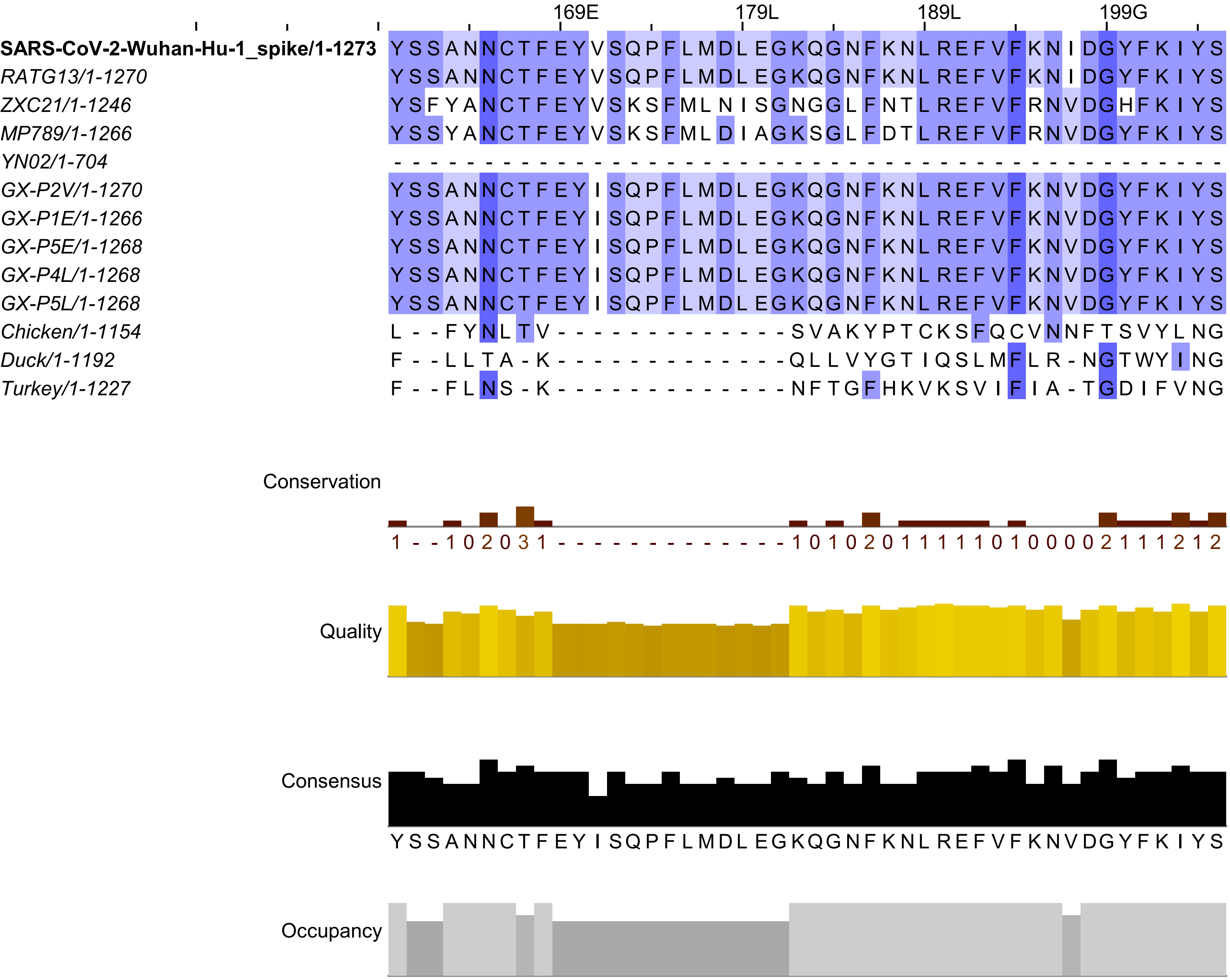
169E
179L
189L
199G
SARS-CoV-2-Wuhan-Hu-1_spike/1-1273 YSSANNCTFEYVSQPFLMDLEGKQGNFKNLREFVFKNIDGYFKIYS
RATG13/1-1270 YSSANNCTFEYVSQPFLMDLEGKQGNFKNLREFVFKNIDGYFKIYS
ZXC21/1-1246 YSFYANCTFEYVSKSFMLNISGNGGLFNTLREFVFRNVDGHFKIYS
MP789/1-1266 YSSYANCTFEYVSKSFMLDIAGKSGLFDTLREFVFRNVDGYFKIYS
YN02/1-704
GX-P2V/1-1270 YSSANNCTFEYISQPFLMDLEGKQGNFKNLREFVFKNVDGYFKIYS
GX-P1E/1-1266 YSSANNCTFEYISQPFLMDLEGKQGNFKNLREFVFKNVDGYFKIYS
GX-P5E/1-1268 YSSANNCTFEYISQPFLMDLEGKQGNFKNLREFVFKNVDGYFKIYS
GX-P4L/1-1268 YSSANNCTFEYISQPFLMDLEGKQGNFKNLREFVFKNVDGYFKIYS
GX-P5L/1-1268 YSSANNCTFEYISQPFLMDLEGKQGNFKNLREFVFKNVDGYFKIYS
Chicken/1-1154 L--FYNLTV-------------SVAKYPTCKSFQCVNNFTSVYLNG
Duck/1-1192 F--LLTA-K-------------QLLVYGTIQSLMFLR-NGTWYING
Turkey/1-1227 F--FLNS-K-------------NFTGFHKVKSVIFIA-TGDIFVNG
Conservation
Quality
Consensus
YSSANNCTFEYISQPFLMDLEGKQGNFKNLREFVFKNVDGYFKIYS
Occupancy

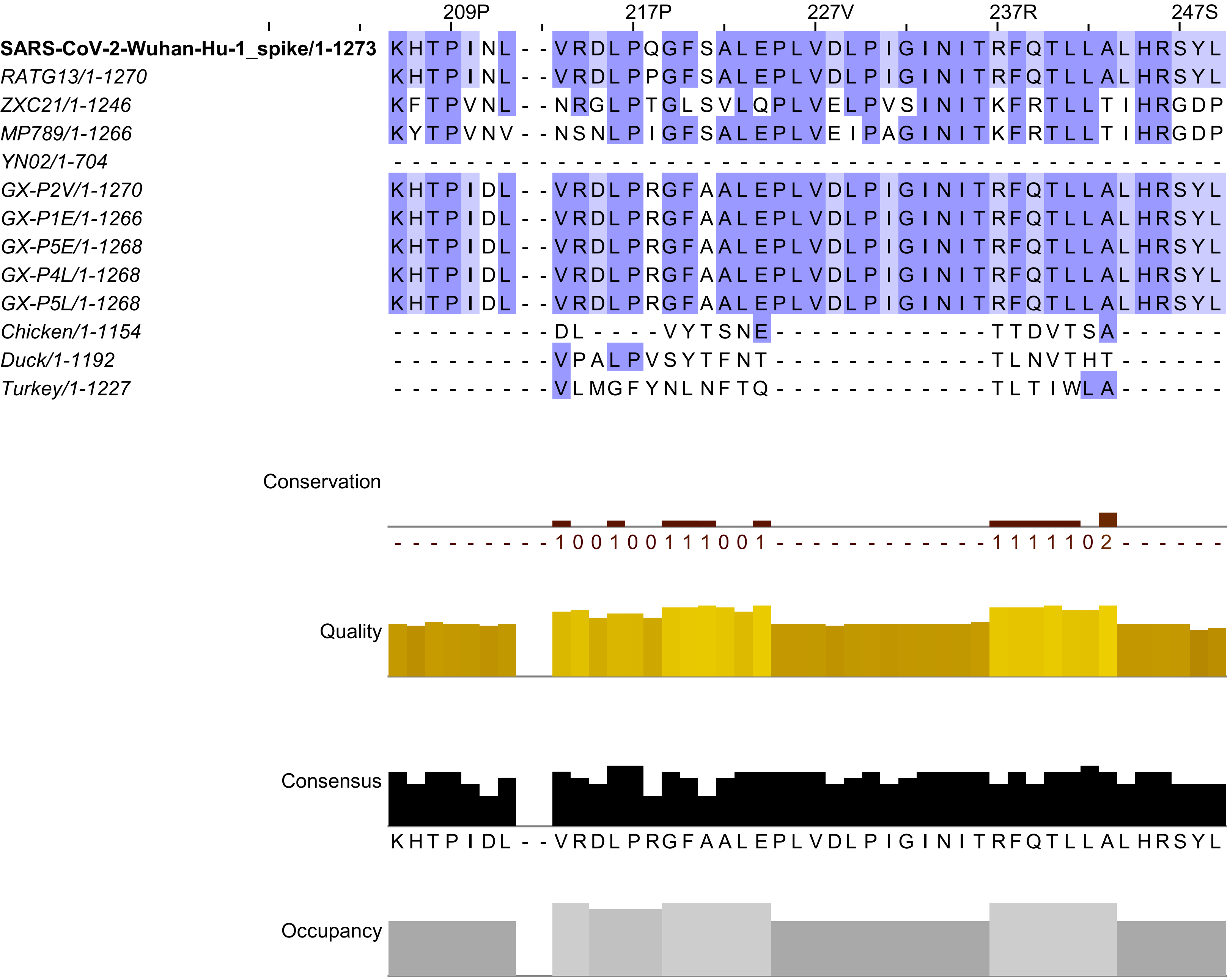

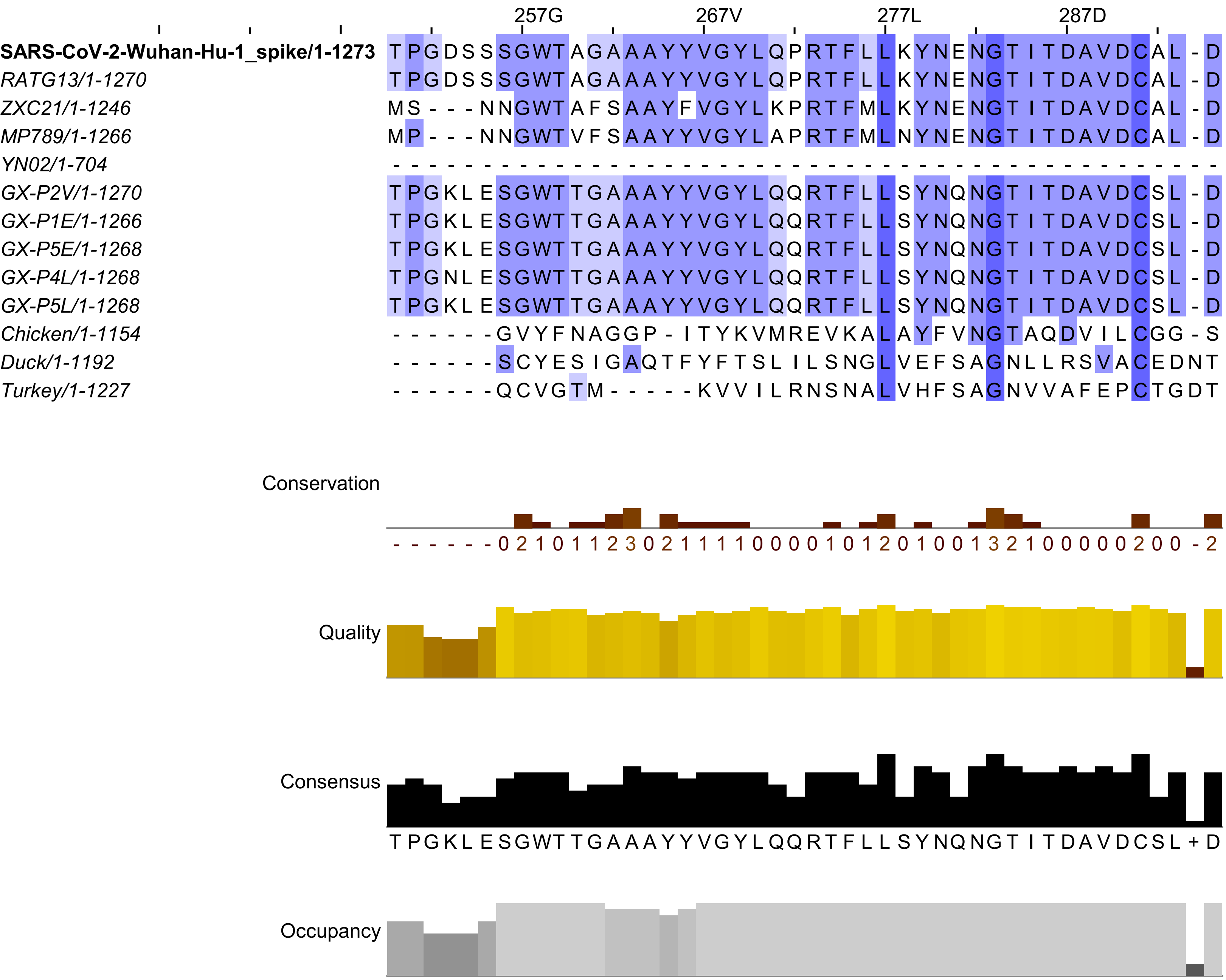
257G
267V
277L
287D
SARS-CoV-2-Wuhan-Hu-1_spike/1-1273 TPGDSSSGWTAGAAAYYVGYLQPRTFLLKYNENGTITDAVDCAL-D
RATG13/1-1270 TPGDSSSGWTAGAAAYYVGYLQPRTFLLKYNENGTITDAVDCAL-D
ZXC21/1-1246 MS---NNGWTAFSAAYFVGYLKPRTFMLKYNENGTITDAVDCAL-D
MP789/1-1266 MP---NNGWTVFSAAYYVGYLAPRTFMLNYNENGTITDAVDCAL-D
YN02/1-704 --
GX-P2V/1-1270 TPGKLESGWTTGAAAYYVGYLQQRTFLLSYNQNGTITDAVDCSL-D
GX-P1E/1-1266 TPGKLESGWTTGAAAYYVGYLQQRTFLLSYNQNGTITDAVDCSL-D
GX-P5E/1-1268 TPGKLESGWTTGAAAYYVGYLQQRTFLLSYNQNGTITDAVDCSL-D
GX-P4L/1-1268 TPGNLESGWTTGAAAYYVGYLQQRTFLLSYNQNGTITDAVDCSL-D
GX-P5L/1-1268 TPGKLESGWTTGAAAYYVGYLQQRTFLLSYNQNGTITDAVDCSL-D
Chicken/1-1154 ------GVYFNAGGP-ITYKVMREVKALAYFVNGTAQDVILCGG-S
Duck/1-1192 ------SCYESIGAQTFYFTSLILSNGLVEFSAGNLLRSVACEDNT
Turkey/1-1227 ------QCVGTM-----KVVILRNSNALVHFSAGNVVAFEPCTGDT
Conservation
------0210112302111100001012010013210000020 0-2
Quality
Consensus
TPGKLESGWTTGAAAYYVGYLQQRTFLLSYNQNGTITDAVDCSL+D
Occupancy

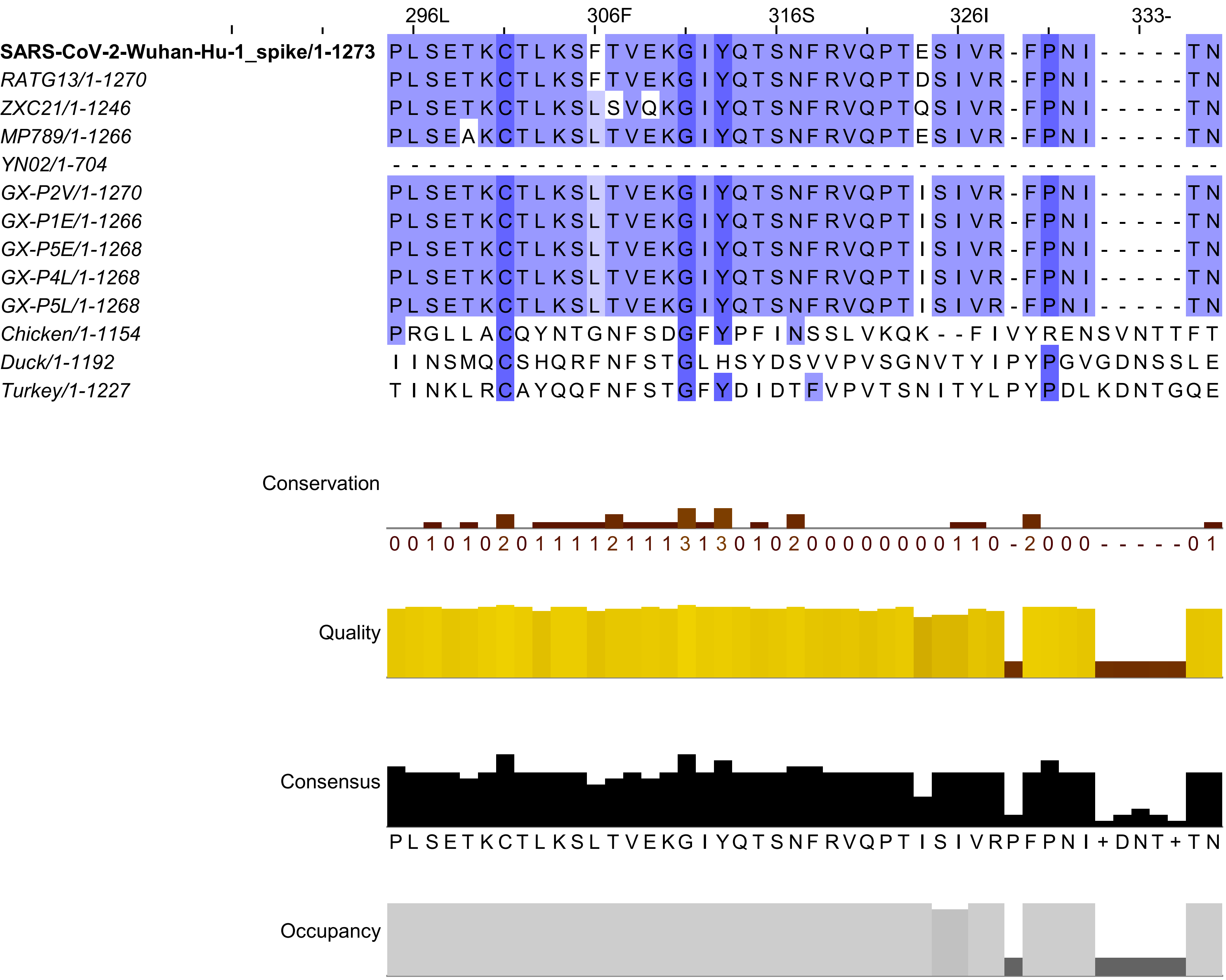
296L
306F
316S
326I
333-
SARS-CoV-2-Wuhan-Hu-1_spike/1-1273 PLSETKCTLKSFTVEKGIYQTSNFRVQPTESIVR-FPNI-----TN
RATG13/1-1270 PLSETKCTLKSFTVEKGIYQTSNFRVQPTDSIVR-FPNI-----TN
ZXC21/1-1246 PLSETKCTLKSLSVQKGIYQTSNFRVQPTQSIVR-FPNI-----TN
MP789/1-1266 PLSEAKCTLKSLTVEKGIYQTSNFRVQPTESIVR-FPNI-----TN
YN02/1-704 --
GX-P2V/1-1270 PLSETKCTLKSLTVEKGIYQTSNFRVQPTISIVR-FPNI-----TN
GX-P1E/1-1266 PLSETKCTLKSLTVEKGIYQTSNFRVQPTISIVR-FPNI-----TN
GX-P5E/1-1268 PLSETKCTLKSLTVEKGIYQTSNFRVQPTISIVR-FPNI-----TN
GX-P4L/1-1268 PLSETKCTLKSLTVEKGIYQTSNFRVQPTISIVR-FPNI-----TN
GX-P5L/1-1268 PLSETKCTLKSLTVEKGIYQTSNFRVQPTISIVR-FPNI-----TN
Chicken/1-1154 PRGLLACQYNTGNFSDGFYPFINSSLVKQK--FIVYRENSVNTTFT
Duck/1-1192 IINSMQCSHQRFNFSTGLHSYDSVVPVSGNVTYIPYPGVGDNSSLE
Turkey/1-1227 TINKLRCAYQQFNFSTGFYDIDTFVPVTSNITYLPYPDLKDNTGQE
Conservation
0 0 1 0 1 0 2 0 1 1 1 1 2 1 1 1 3 1 3 0 1 0 2 0 0 0 0 0 0 0 1 1 0 - 2 0 0 0 - - - - - 0 1
Quality
Consensus
PLSETKCTLKSLTVEKGIYQTSNFRVQPTISIVRPFPNI+DNT+TN
Occupancy

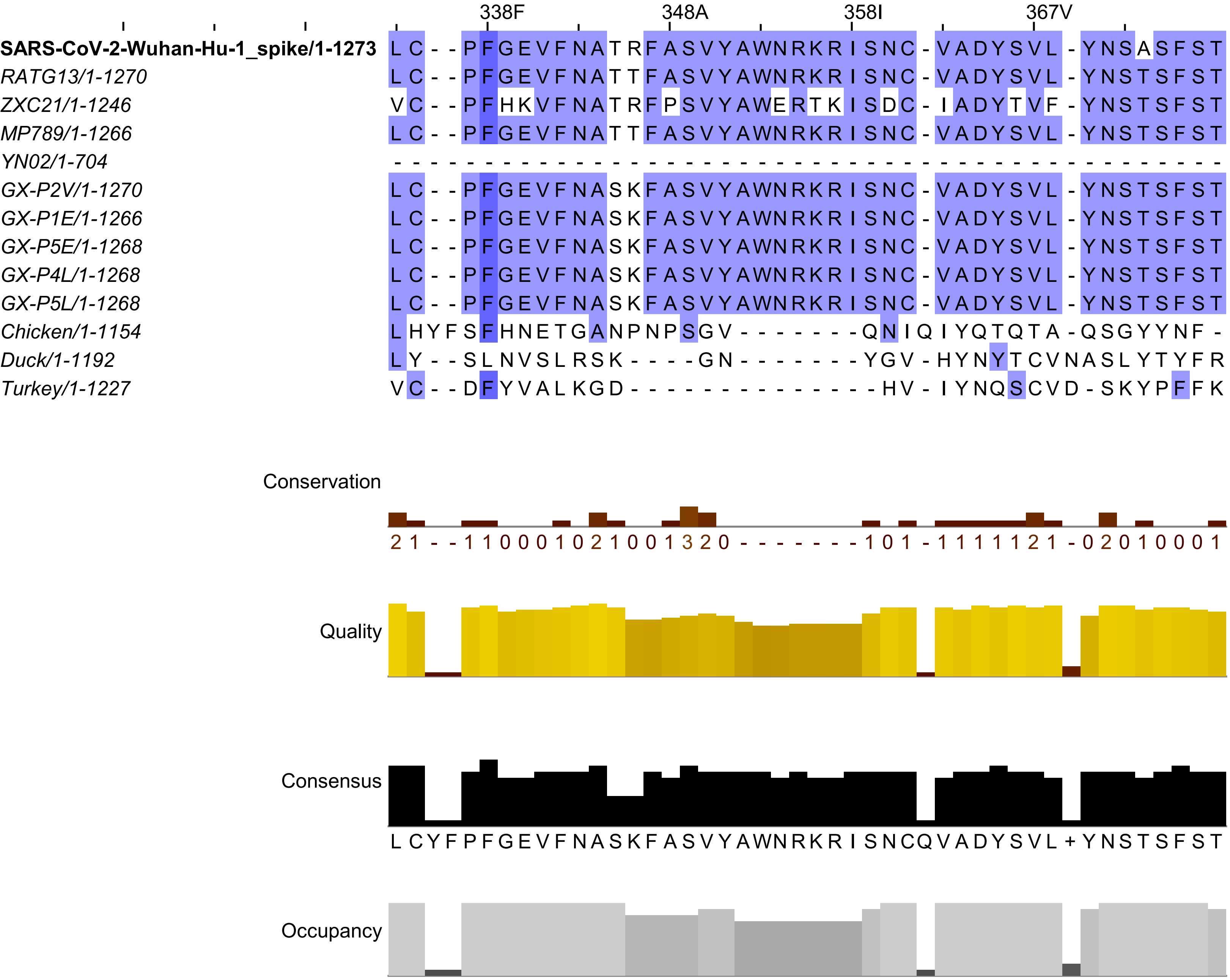
338F
348A
358I
367V
SARS-CoV-2-Wuhan-Hu-1_spike/1-1273 LC--PFGEVFNATRFASVYAWNRKRISNC-VADYSVL-YNSASFST
RATG13/1-1270 LC--PFGEVFNATTFASVYAWNRKRISNC-VADYSVL-YNSTSFST
ZXC21/1-1246 VC--PFHKVFNATRFPSVYAWERTKISDC-IADYTVF-YNSTSFST
MP789/1-1266 LC--PFGEVFNATTFASVYAWNRKRISNC-VADYSVL-YNSTSFST
YN02/1-704 --
GX-P2V/1-1270 LC--PFGEVFNASKFASVYAWNRKRISNC-VADYSVL-YNSTSFST
GX-P1E/1-1266 LC--PFGEVFNASKFASVYAWNRKRISNC-VADYSVL-YNSTSFST
GX-P5E/1-1268 LC--PFGEVFNASKFASVYAWNRKRISNC-VADYSVL-YNSTSFST
GX-P4L/1-1268 LC--PFGEVFNASKFASVYAWNRKRISNC-VADYSVL-YNSTSFST
GX-P5L/1-1268 LC--PFGEVFNASKFASVYAWNRKRISNC-VADYSVL-YNSTSFST
Chicken/1-1154 LHYFSFHNETGANPNPSGV-------QNIQIYQTQTA-QSGYYNF-
Duck/1-1192 LY--SLNVSLRSK----GN--------YGV-HYNYTCVNASLYTYFR
Turkey/1-1227 VC--DFYVALKGD---------------HV-IYNQSCVD-SKYPFFK
Conservation
21--110001021001320--------101-1111121-02010001
Quality
Consensus
LCYFPFGEVFNASKFASVYAWNRKRISNCQVADYSVL+YNSTSFST
Occupancy

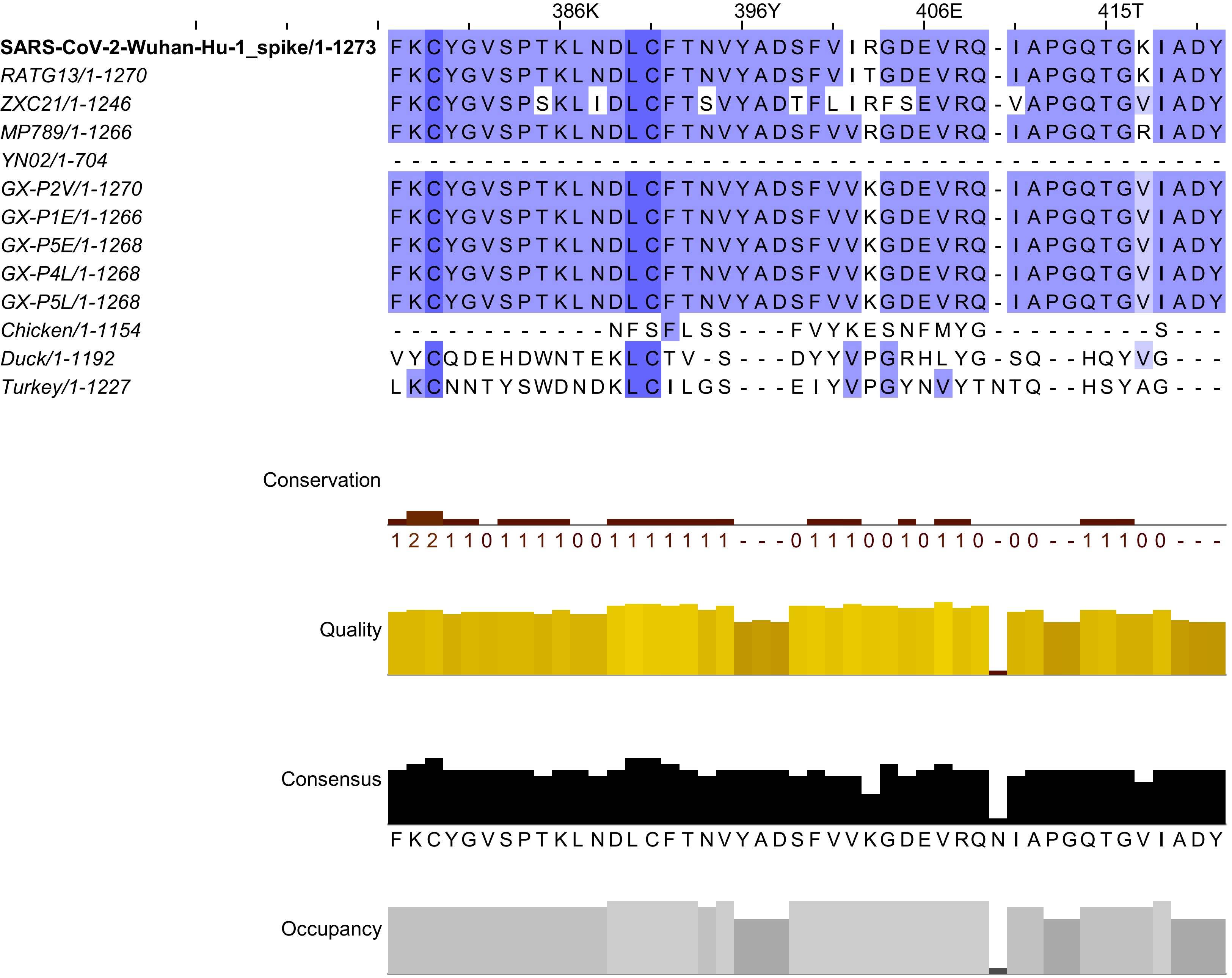

386K
396Y
406E
415T
SARS-CoV-2-Wuhan-Hu-1_spike/1-1273
RATG13/1-1270
ZXC21/1-1246
MP789/1-1266
YN02/1-704
GX-P2V/1-1270
GX-P1E/1-1266
GX-P5E/1-1268
GX-P4L/1-1268
GX-P5L/1-1268
Chicken/1-1154
Duck/1-1192
Turkey/1-1227
Conservation
Quality
Consensus
Occupancy

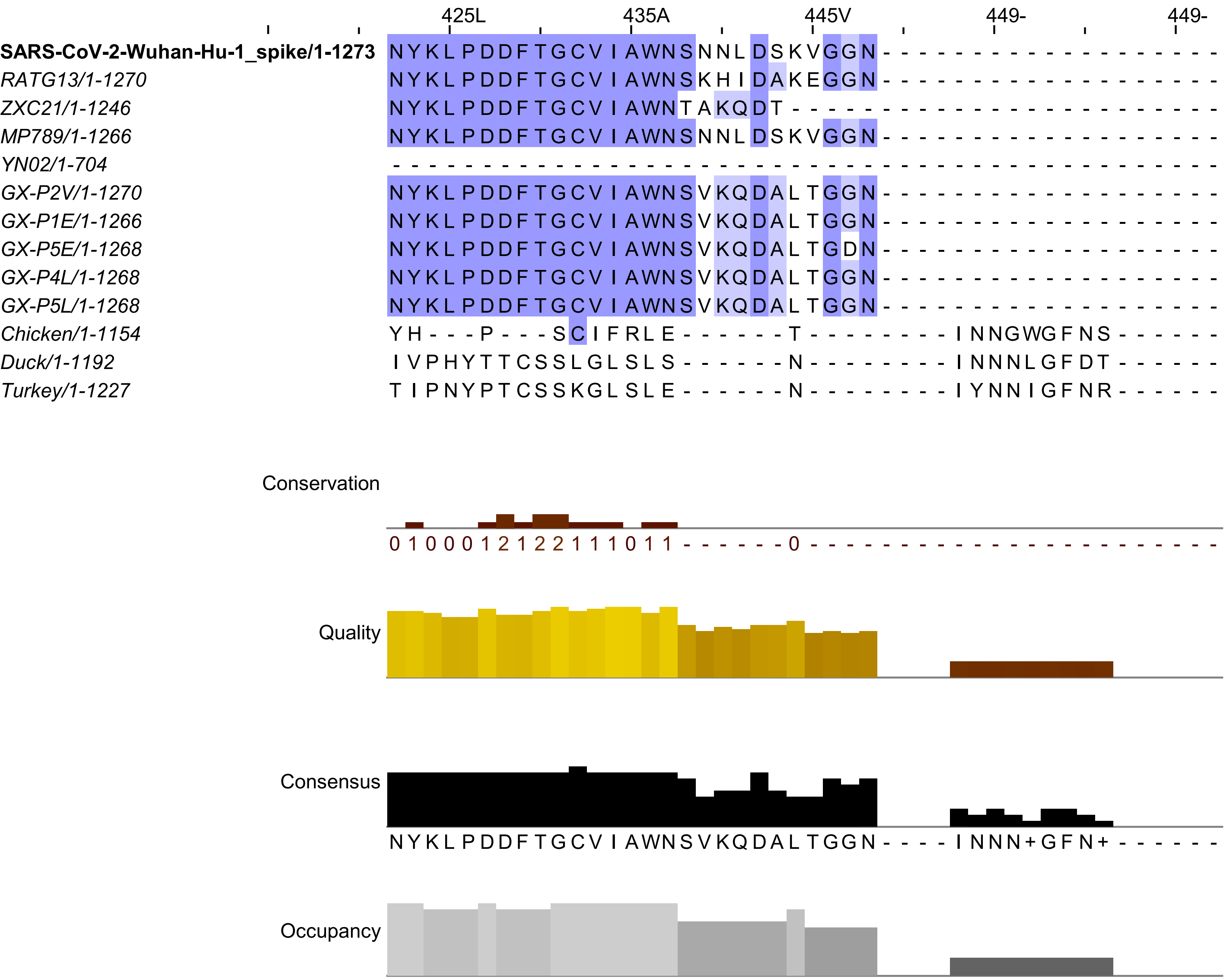
425L
435A
445V
449-
449-
SARS-CoV-2-Wuhan-Hu-1_spike/1-1273 NYKLPDDFTGCVIAWNSNNLDSKVGGN---------------------
RATG13/1-1270 NYKLPDDFTGCVIAWNSKHIDAKEGGN---------------------
ZXC21/1-1246 NYKLPDDFTGCVIAWNTAKQDT--------------------------
MP789/1-1266 NYKLPDDFTGCVIAWNSNNLDSKVGGN---------------------
YN02/1-704 --
GX-P2V/1-1270 NYKLPDDFTGCVIAWNSVKQDALTGGN---------------------
GX-P1E/1-1266 NYKLPDDFTGCVIAWNSVKQDALTGGN---------------------
GX-P5E/1-1268 NYKLPDDFTGCVIAWNSVKQDALTGDN---------------------
GX-P4L/1-1268 NYKLPDDFTGCVIAWNSVKQDALTGGN---------------------
GX-P5L/1-1268 NYKLPDDFTGCVIAWNSVKQDALTGGN---------------------
Chicken/1-1154 YH---P---SCIFRLE------T---------INNGWGFNS------
Duck/1-1192 IVPHYTTCSSLGLSLS------N---------INNNLGFDT------
Turkey/1-1227 TIPNYPTCSSKGLSLE------N---------IYNNIGFNR------
Conservation
0100012122111011------0-------------------------
Quality
Consensus
NYKLPDDFTGCVIAWNSVKQDALTGGN----INNN+GFN+------
Occupancy

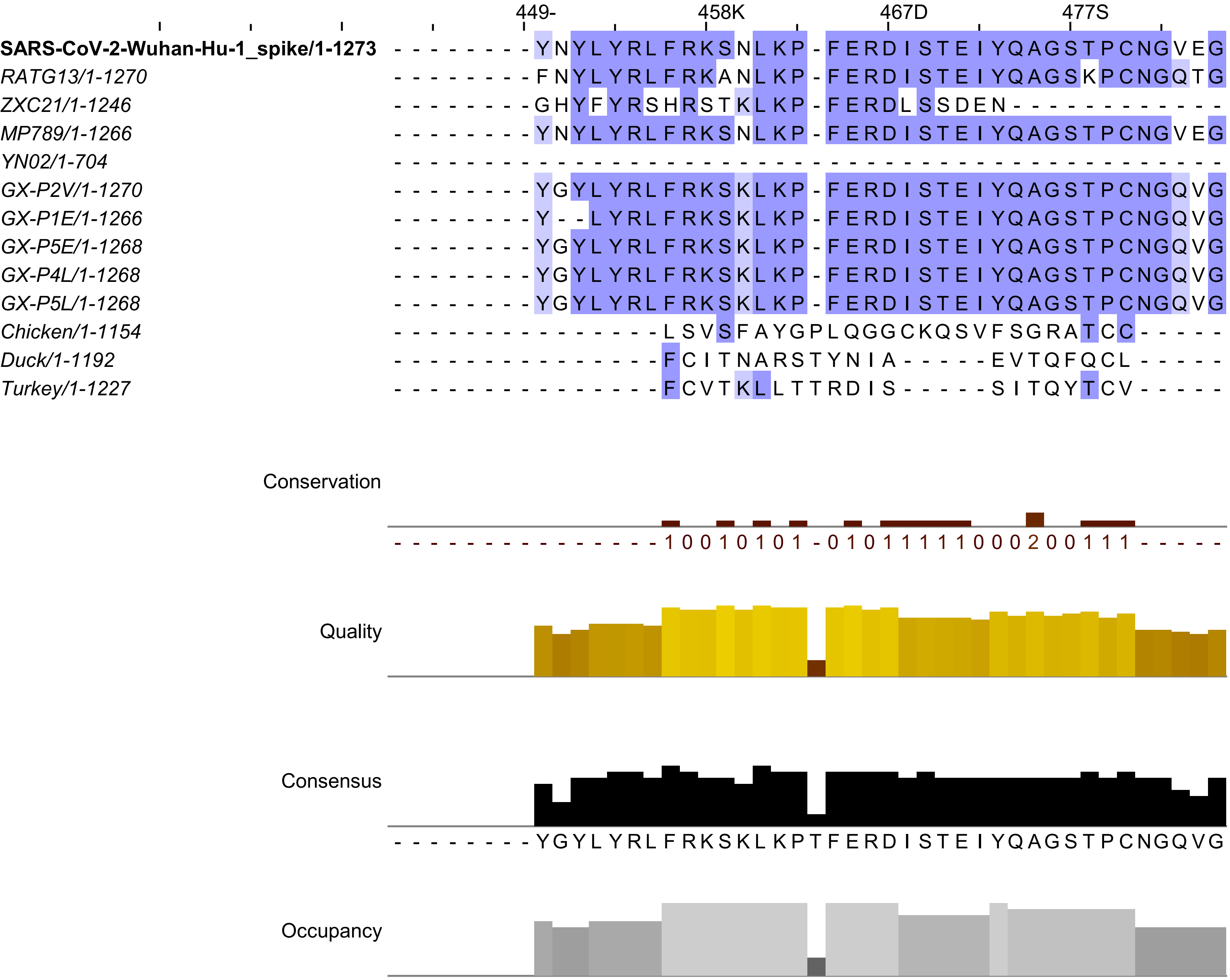
449-
458K
467D
477S
SARS-CoV-2-Wuhan-Hu-1_spike/1-1273 --------YNYLYRLFRKSNLKP-FERDISTEIYQAGSTPCNGVEG
RATG13/1-1270 --------FNYLYRLFRKANLKP-FERDISTEIYQAGSKPCNGQTG
ZXC21/1-1246 --------GHYFYRSHRSTKLKP-FERDLSSDEN------------
MP789/1-1266 --------YNYLYRLFRKSNLKP-FERDISTEIYQAGSTPCNGVEG
YN02/1-704 --
GX-P2V/1-1270 --------YGYLYRLFRKSKLKP-FERDISTEIYQAGSTPCNGQVG
GX-P1E/1-1266 --------Y--LYRLFRKSKLKP-FERDISTEIYQAGSTPCNGQVG
GX-P5E/1-1268 --------YGYLYRLFRKSKLKP-FERDISTEIYQAGSTPCNGQVG
GX-P4L/1-1268 --------YGYLYRLFRKSKLKP-FERDISTEIYQAGSTPCNGQVG
GX-P5L/1-1268 --------YGYLYRLFRKSKLKP-FERDISTEIYQAGSTPCNGQVG
Chicken/1-1154 ---------------LSVSFAYGPLQGGCKQSVFSGRATCC-----
Duck/1-1192 ---------------FCITNARSTYNIA-----EVTQFQCL-----
Turkey/1-1227 ---------------FCVTKLLTTRDIS-----SITQYTCV-----
Conservation
---------------10010101-0101111100020 0111-----
Quality
Consensus
--------YGYLYRLFRKSKLKPTFERDISTEIYQAGSTPCNGQVG
Occupancy

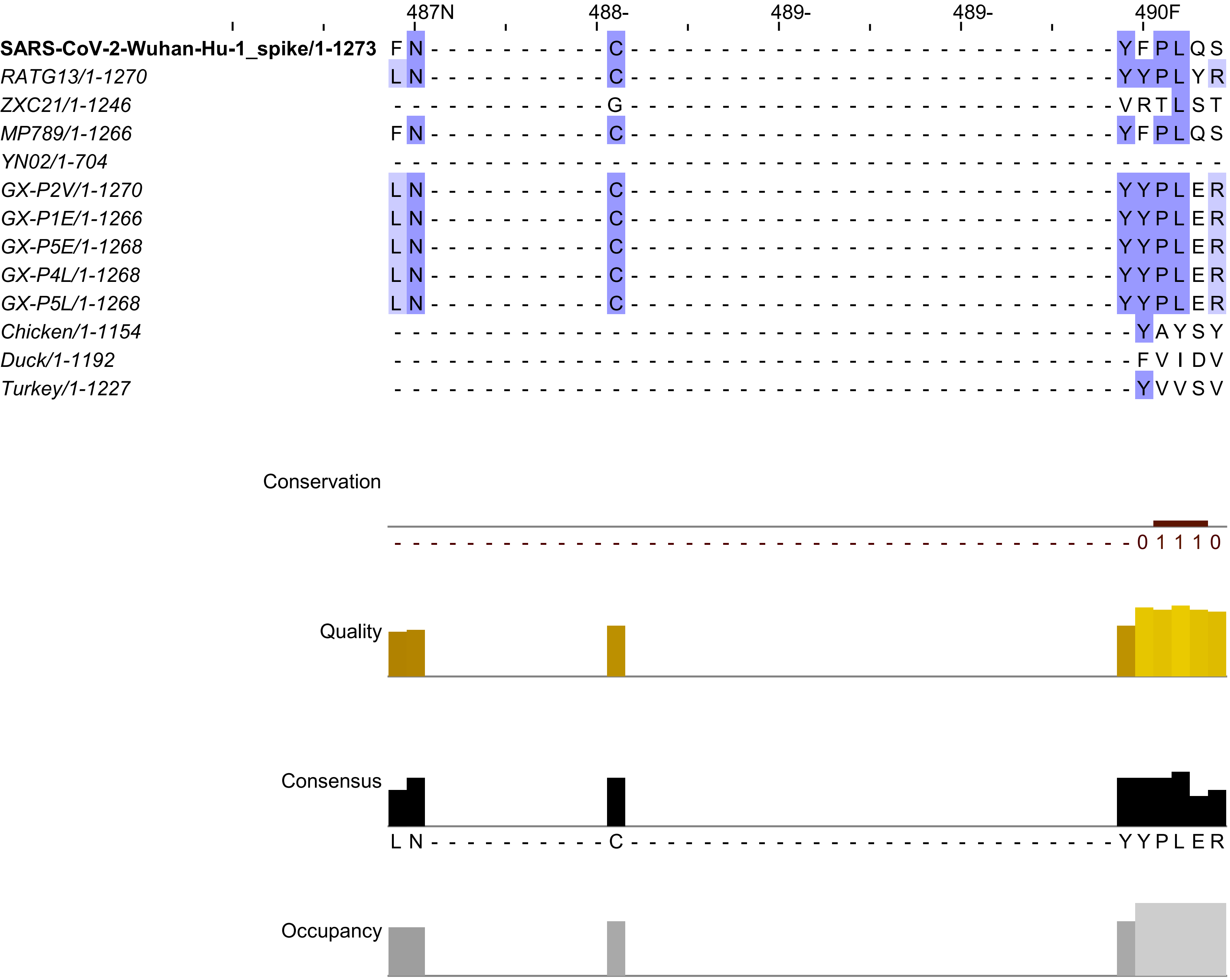
487N
488-
489-
489-
490F
SARS-CoV-2-Wuhan-Hu-1_spike/1-1273 F N - - - - - - - - - - C - Y F P L Q S
RATG13/1-1270 L N - - - - - - - - - - C - Y Y P L Y R
ZXC21/1-1246 - - - - - - - - - - - - G - V R T L S T
MP789/1-1266 F N - - - - - - - - - - C - Y F P L Q S
YN02/1-704
GX-P2V/1-1270 L N - - - - - - - - - - C - Y Y P L E R
GX-P1E/1-1266 L N - - - - - - - - - - C - Y Y P L E R
GX-P5E/1-1268 L N - - - - - - - - - - C - Y Y P L E R
GX-P4L/1-1268 L N - - - - - - - - - - C - Y Y P L E R
GX-P5L/1-1268 L N - - - - - - - - - - C - Y Y P L E R
Chicken/1-1154 - Y A Y S Y
Duck/1-1192 - F V I D V
Turkey/1-1227 - Y V V S V
Conservation
0 1 1 1 0
Quality
Consensus
L N - - - - - - - - - - C - Y Y P L E R
Occupancy

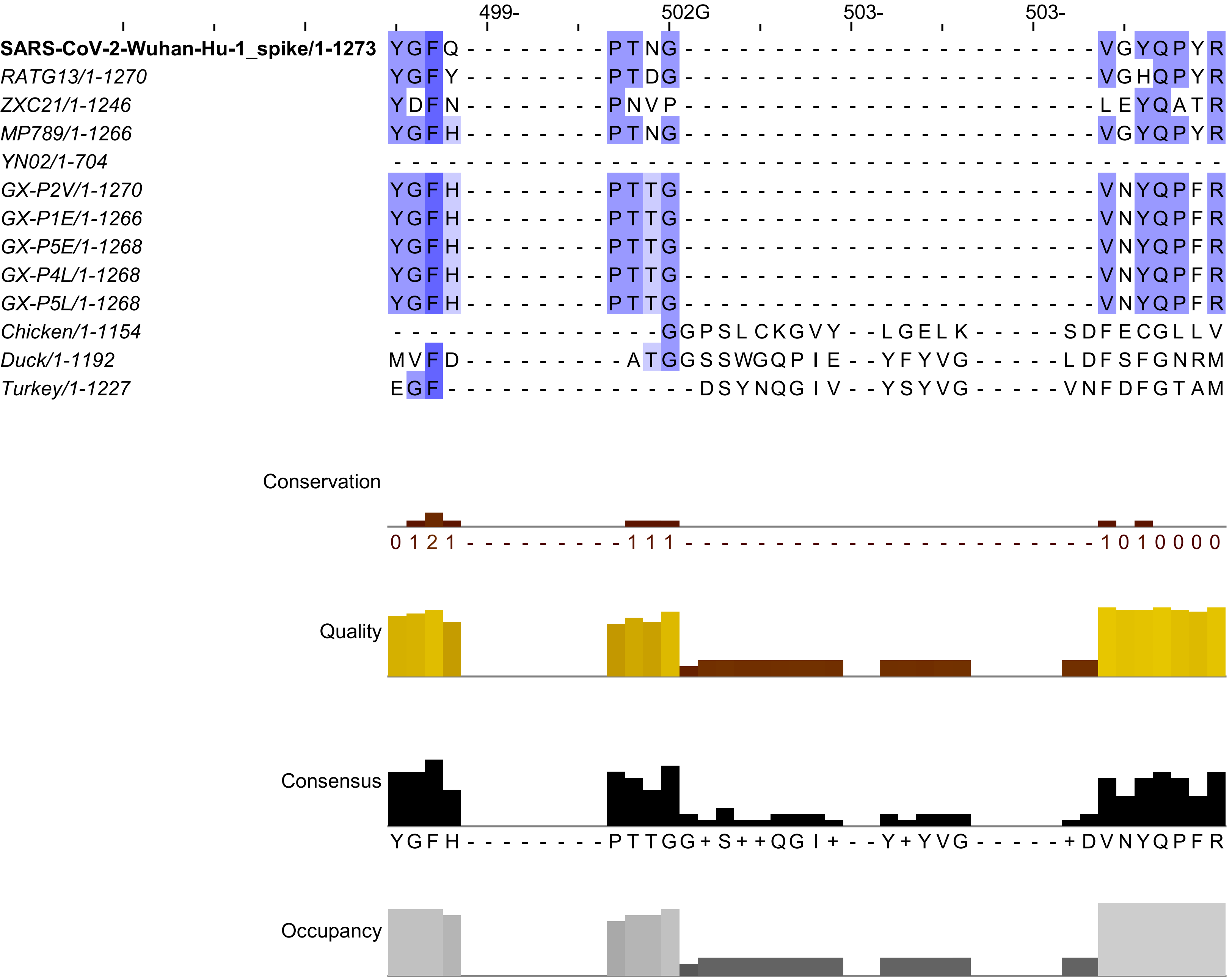

499-
502G
503-
503-
SARS-CoV-2-Wuhan-Hu-1_spike/1-1273
RATG13/1-1270
ZXC21/1-1246
MP789/1-1266
YN02/1-704
GX-P2V/1-1270
GX-P1E/1-1266
GX-P5E/1-1268
GX-P4L/1-1268
GX-P5L/1-1268
Chicken/1-1154
Duck/1-1192
Turkey/1-1227
Conservation
Quality
Consensus
Occupancy

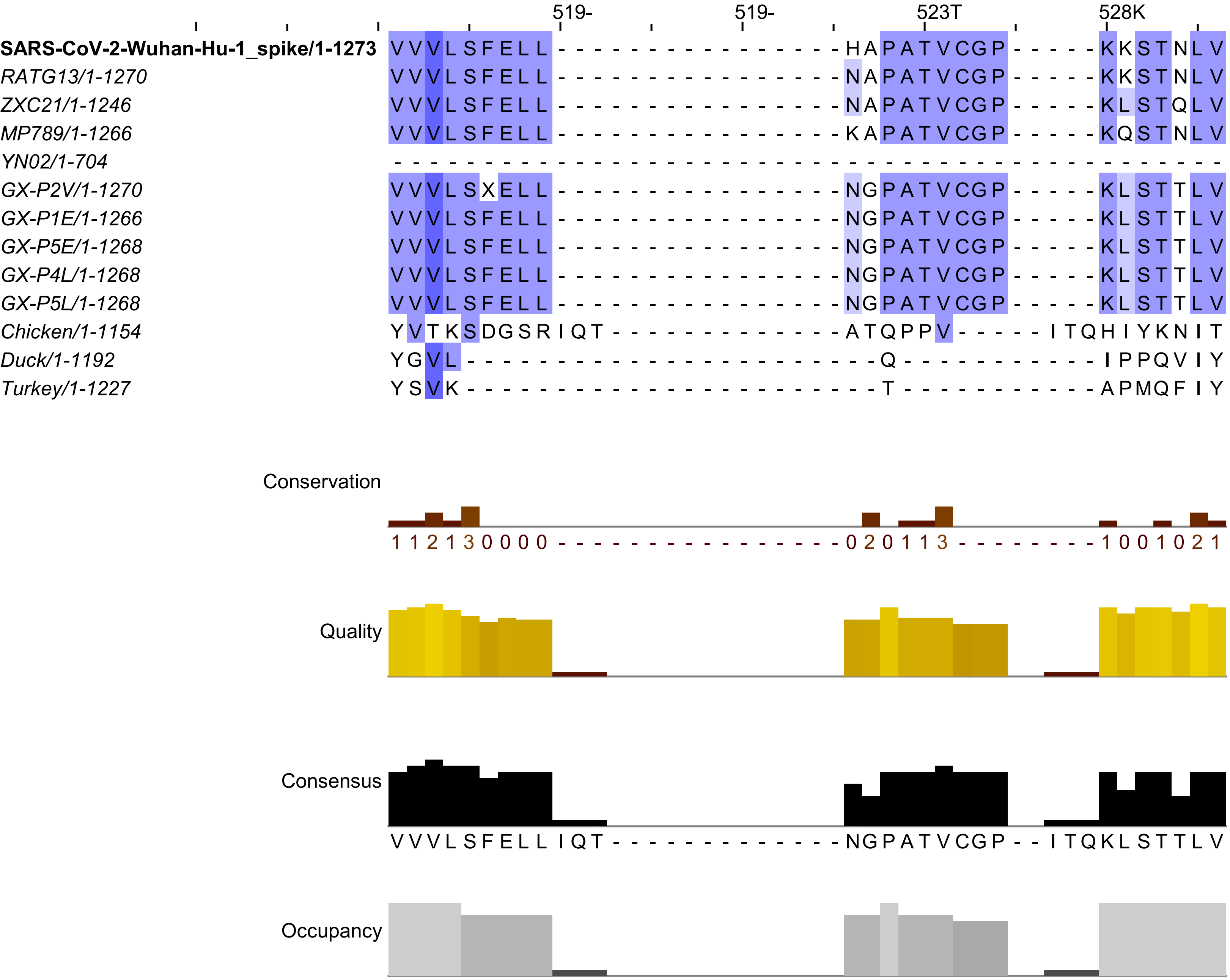
519-
519-
523T
528K
SARS-CoV-2-Wuhan-Hu-1_spike/1-1273 VVVLSFELL----------------HAPATVCGP-----KKSTNLV
RATG13/1-1270 VVVLSFELL----------------NAPATVCGP-----KKSTNLV
ZXC21/1-1246 VVVLSFELL----------------NAPATVCGP-----KLSTQLV
MP789/1-1266 VVVLSFELL----------------KAPATVCGP-----KQSTNLV
YN02/1-704 --
GX-P2V/1-1270 VVVLSXELL----------------NGPATVCGP-----KLSTTLV
GX-P1E/1-1266 VVVLSFELL----------------NGPATVCGP-----KLSTTLV
GX-P5E/1-1268 VVVLSFELL----------------NGPATVCGP-----KLSTTLV
GX-P4L/1-1268 VVVLSFELL----------------NGPATVCGP-----KLSTTLV
GX-P5L/1-1268 VVVLSFELL----------------NGPATVCGP-----KLSTTLV
Chicken/1-1154 YVTKSDGSRIQT-------------ATQPPV-----ITQHIYKNIT
Duck/1-1192 YGVL---------------------Q------------IPPQVIY
Turkey/1-1227 YSVK---------------------T------------APMQFIY
Conservation
112130000----------------020113---------1001021
Quality
Consensus
VVVLSFELLIQT-------------NGPATVCGP--ITQKLSTTLV
Occupancy

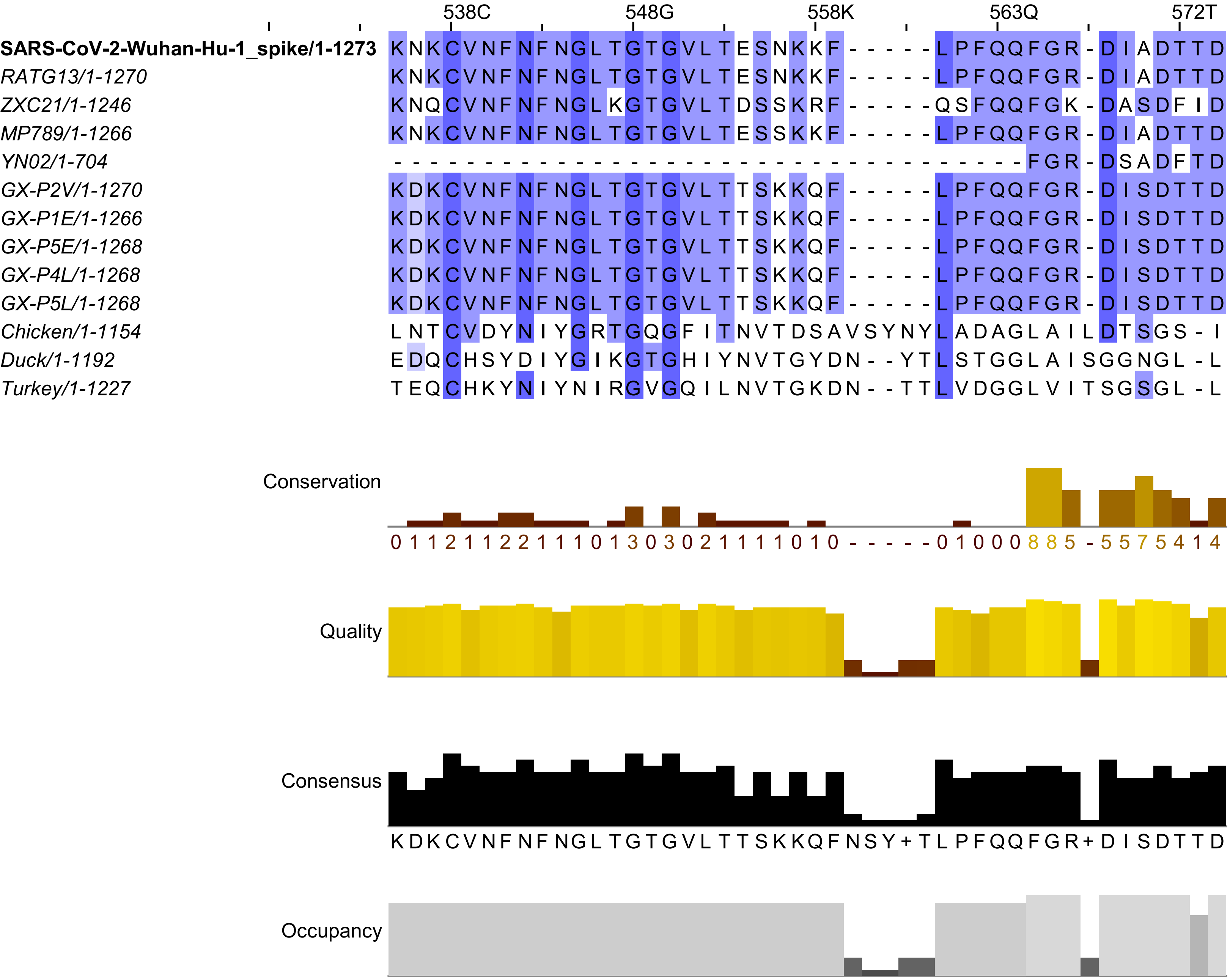
538C
548G
558K
563Q
572T
SARS-CoV-2-Wuhan-Hu-1_spike/1-1273
RATG13/1-1270
ZXC21/1-1246
MP789/1-1266
YN02/1-704
GX-P2V/1-1270
GX-P1E/1-1266
GX-P5E/1-1268
GX-P4L/1-1268
GX-P5L/1-1268
Chicken/1-1154
Duck/1-1192
Turkey/1-1227
Conservation
Quality
Consensus
Occupancy

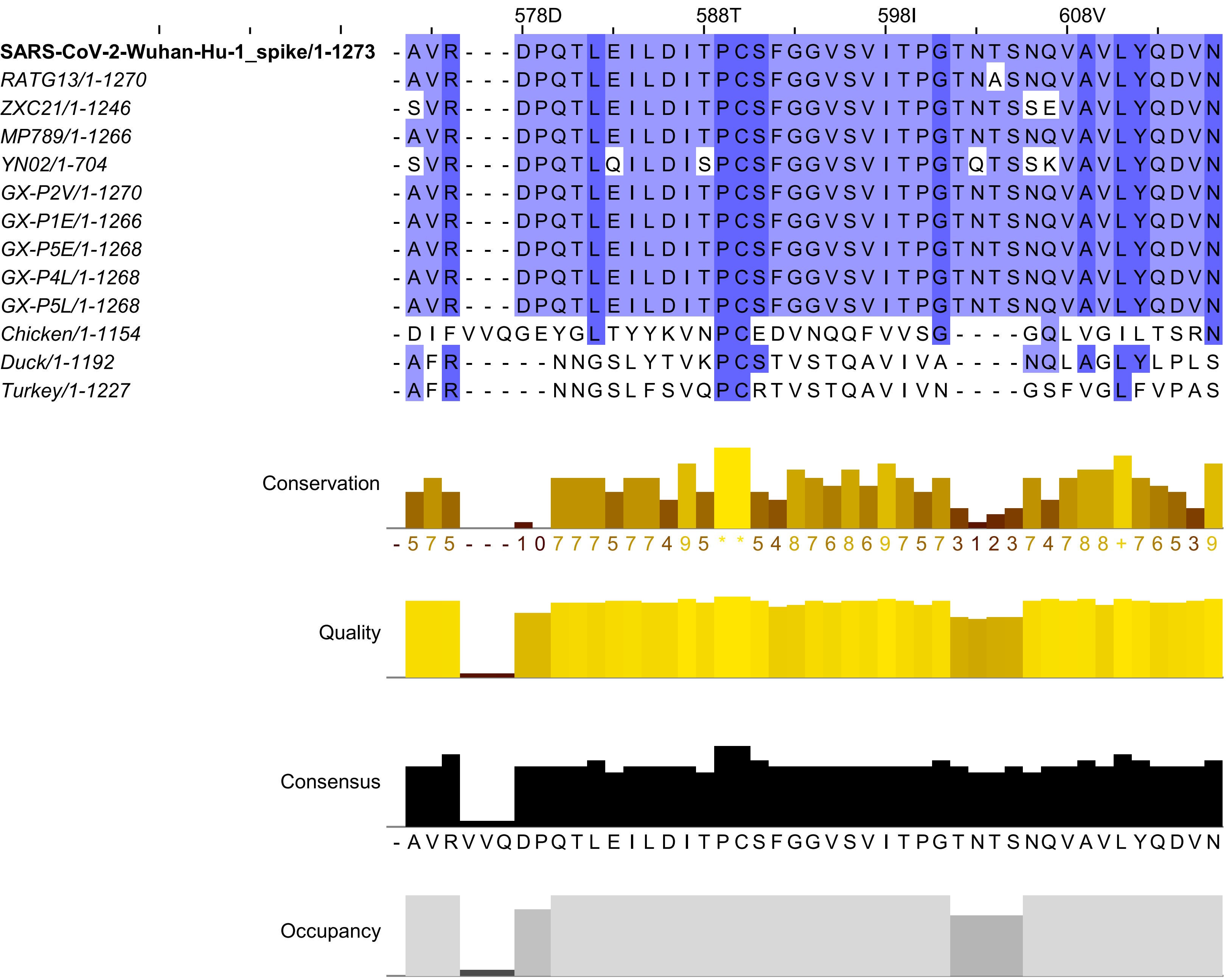

578D
588T
598I
608V
SARS-CoV-2-Wuhan-Hu-1_spike/1-1273
RATG13/1-1270
ZXC21/1-1246
MP789/1-1266
YN02/1-704
GX-P2V/1-1270
GX-P1E/1-1266
GX-P5E/1-1268
GX-P4L/1-1268
GX-P5L/1-1268
Chicken/1-1154
Duck/1-1192
Turkey/1-1227
Conservation
Quality
Consensus
Occupancy

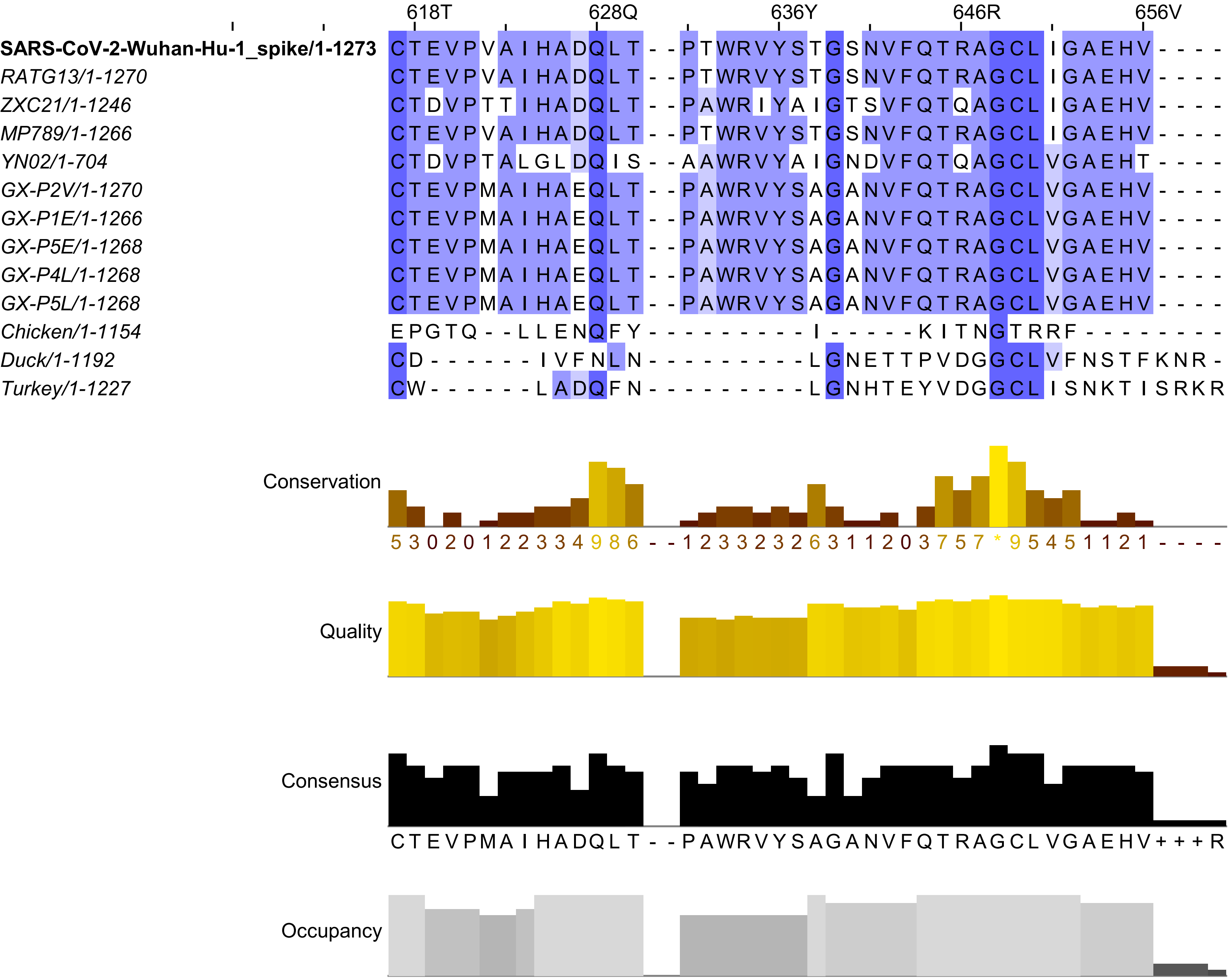

618T
628Q
636Y
646R
656V
SARS-CoV-2-Wuhan-Hu-1_spike/1-1273 CTEVPVAIHADQLT--PTWRVYSTGSNVFQTRAGCLIGAEHV----
RATG13/1-1270 CTEVPVAIHADQLT--PTWRVYSTGSNVFQTRAGCLIGAEHV----
ZXC21/1-1246 CTDVPTTIHADQLT--PAWRIYAIGTSVFQTQAGCLIGAEHV----
MP789/1-1266 CTEVPVAIHADQLT--PTWRVYSTGSNVFQTRAGCLIGAEHV----
YN02/1-704 CTDVPTALGLDQIS--AAWRVYAIGNDVFQTQAGCLVGAEHT----
GX-P2V/1-1270 CTEVPMAIHAEQLT--PAWRVYSAGANVFQTRAGCLVGAEHV----
GX-P1E/1-1266 CTEVPMAIHAEQLT--PAWRVYSAGANVFQTRAGCLVGAEHV----
GX-P5E/1-1268 CTEVPMAIHAEQLT--PAWRVYSAGANVFQTRAGCLVGAEHV----
GX-P4L/1-1268 CTEVPMAIHAEQLT--PAWRVYSAGANVFQTRAGCLVGAEHV----
GX-P5L/1-1268 CTEVPMAIHAEQLT--PAWRVYSAGANVFQTRAGCLVGAEHV----
Chicken/1-1154 EPGTQ--LLENQFY---------I-----KITNGTRRF--------
Duck/1-1192 CD------IVFNLN---------LGNETTPVDGGCLVFNSTFKNR-
Turkey/1-1227 CW------LADQFN---------LGNHTEYVDGGCLISNKTISRKR
Conservation
5 3 0 2 0 1 2 2 3 3 4 9 8 6 - - 1 2 3 3 2 3 2 6 3 1 1 2 0 3 7 5 7 * 9 5 4 5 1 1 2 1 - - - -
Quality
Consensus
CTEVPMAIHADQLT--PAWRVYSAGANVFQTRAGCLVGAEHV+++R
Occupancy

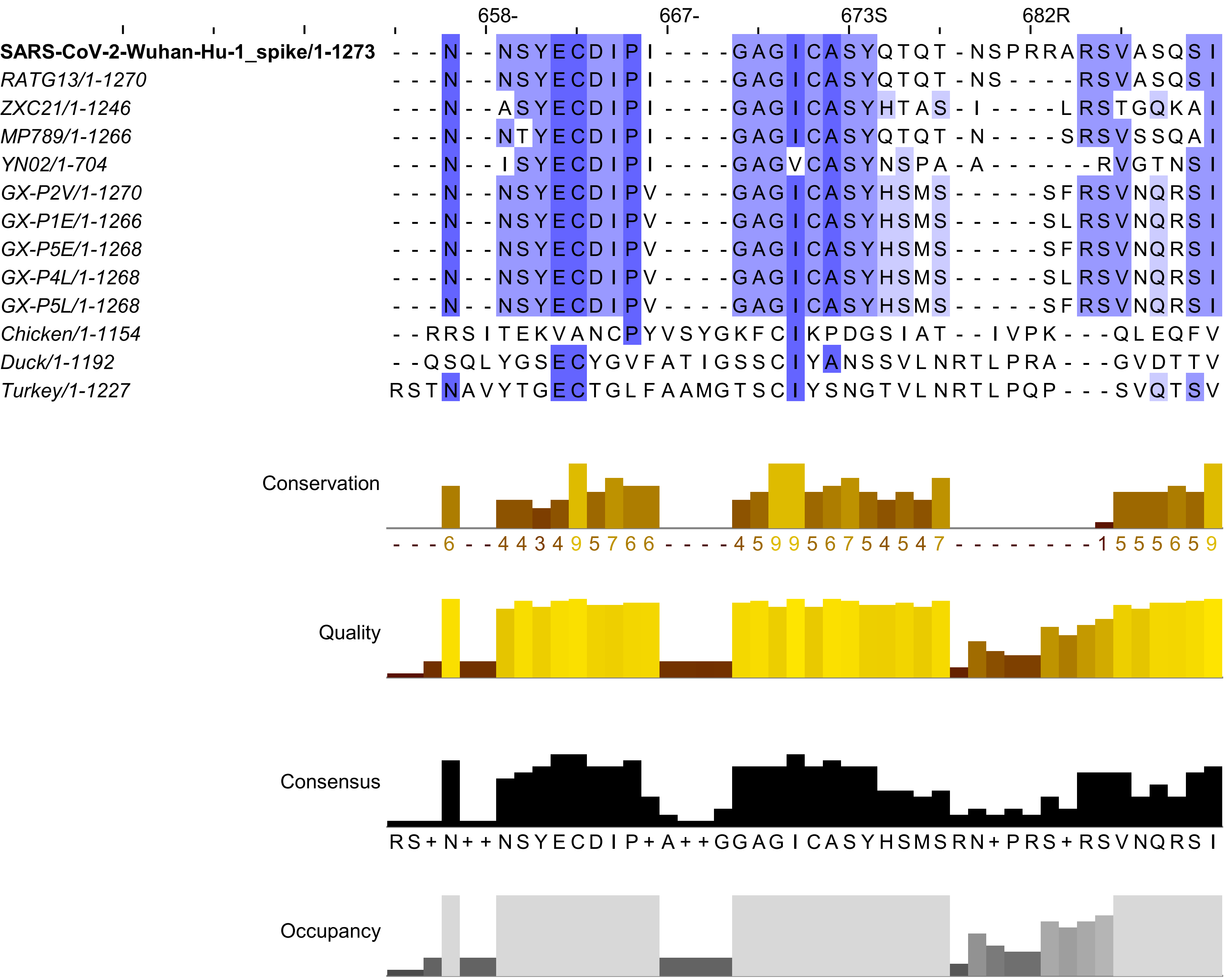
658-
667-
673S
682R
SARS-CoV-2-Wuhan-Hu-1_spike/1-1273 ---N--NSYECDIPI----GAGICASYQTQT-NSPRRARSVASQSI
RATG13/1-1270 ---N--NSYECDIPI----GAGICASYQTQT-NS----RSVASQSI
ZXC21/1-1246 ---N--ASYECDIPI----GAGICASYHTAS-I----LRSTGQKAI
MP789/1-1266 ---N--NTYECDIPI----GAGICASYQTQT-N----SRSVSSQAI
YN02/1-704 ---N--ISYECDIPI----GAGVCASYNSPA-A------RVGTNSI
GX-P2V/1-1270 ---N--NSYECDIPV----GAGICASYHSMS-----SFRSVNQRSI
GX-P1E/1-1266 ---N--NSYECDIPV----GAGICASYHSMS-----SLRSVNQRSI
GX-P5E/1-1268 ---N--NSYECDIPV----GAGICASYHSMS-----SFRSVNQRSI
GX-P4L/1-1268 ---N--NSYECDIPV----GAGICASYHSMS-----SLRSVNQRSI
GX-P5L/1-1268 ---N--NSYECDIPV----GAGICASYHSMS-----SFRSVNQRSI
Chicken/1-1154 --RRSITEKVANCPYVSYGKFCIKPDGSIAT--IVPK---QLEQFV
Duck/1-1192 --QSQLYGSECYGVFATIGSSCIYANSSVLNRTLPRA---GVDTTV
Turkey/1-1227 RSTNAVYTGECTGLFAAMGTSCIYSNGTVLNRTLPQP---SVQTSV
Conservation
---6--44349576 6----459956754547--------1555659
Quality
Consensus
RS+N++NSYECDIP+A++GGAGICASYHSMSRN+PRS+RSVNQRSI
Occupancy

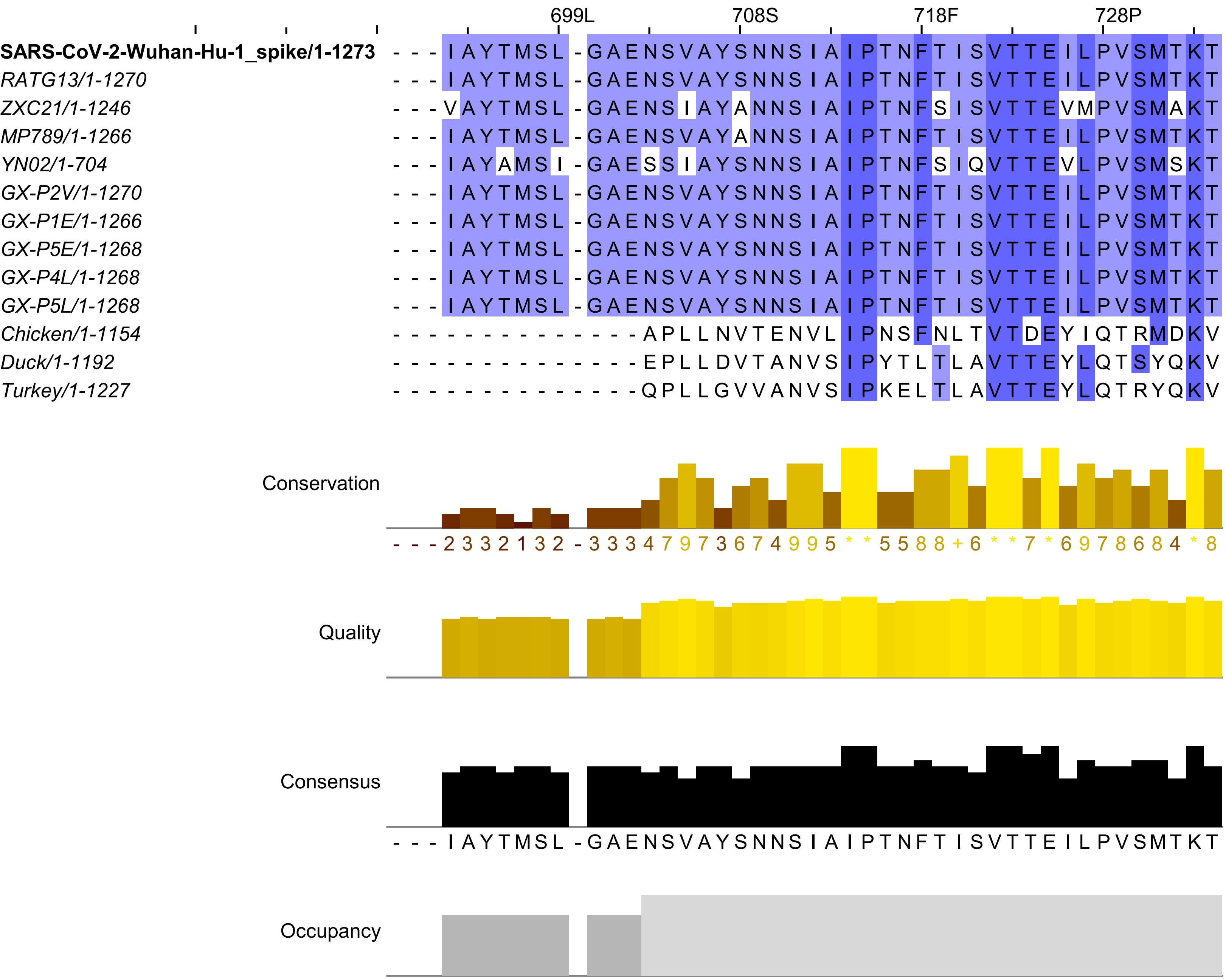
699L
708S
718F
728P
SARS-CoV-2-Wuhan-Hu-1_spike/1-1273 ---IAYTMSL-GAENSVAYSNNSIAIPTNFTISVTTEILPVSMTKT
RATG13/1-1270 ---IAYTMSL-GAENSVAYSNNSIAIPTNFTISVTTEILPVSMTKT
ZXC21/1-1246 ---VAYTMSL-GAENSIAYANNSIAIPTNFSISVTTEVMPVSMAKT
MP789/1-1266 ---IAYTMSL-GAENSVAYANNSIAIPTNFTISVTTEILPVSMTKT
YN02/1-704 ---IAYAMSI-GAESSIAYSNNSIAIPTNFSIQVTTEVLPVSMSKT
GX-P2V/1-1270 ---IAYTMSL-GAENSVAYSNNSIAIPTNFTISVTTEILPVSMTKT
GX-P1E/1-1266 ---IAYTMSL-GAENSVAYSNNSIAIPTNFTISVTTEILPVSMTKT
GX-P5E/1-1268 ---IAYTMSL-GAENSVAYSNNSIAIPTNFTISVTTEILPVSMTKT
GX-P4L/1-1268 ---IAYTMSL-GAENSVAYSNNSIAIPTNFTISVTTEILPVSMTKT
GX-P5L/1-1268 ---IAYTMSL-GAENSVAYSNNSIAIPTNFTISVTTEILPVSMTKT
Chicken/1-1154 --------------APLLNVTENVLIPNSFNLTVTDEYIQTRMDKV
Duck/1-1192 --------------EPLLDVTANVSIPYTLTLAVTTEYLQTSYQKV
Turkey/1-1227 --------------QPLLGVVANVSIPKELTLAVTTEYLQTRYQKV
Conservation
---2332132-3334797367499 5**5588+6**7*697868 4*8
Quality
Consensus
---IAYTMSL-GAENSVAYSNNSIAIPTNFTISVTTEILPVSMTKT
Occupancy

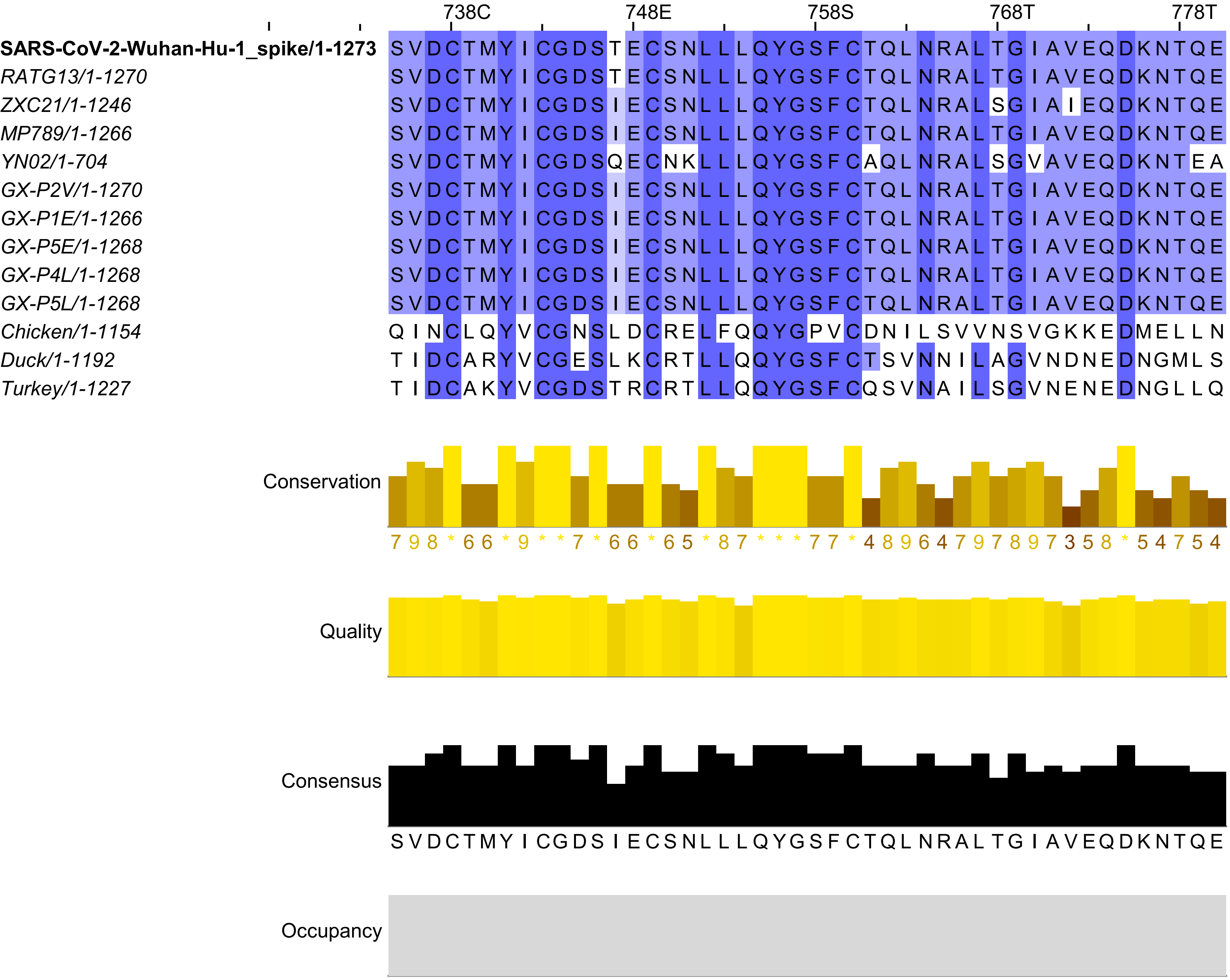
738C
748E
758S
768T
778T
SARS-CoV-2-Wuhan-Hu-1_spike/1-1273 SVDCTMYICGDSTECSNLLLQYGSFCTQLNRALTGIAVEQDKNTQE
RATG13/1-1270 SVDCTMYICGDSTECSNLLLQYGSFCTQLNRALTGIAVEQDKNTQE
ZXC21/1-1246 SVDCTMYICGDSIECSNLLLQYGSFCTQLNRALSGIAIEQDKNTQE
MP789/1-1266 SVDCTMYICGDSIECSNLLLQYGSFCTQLNRALTGIAVEQDKNTQE
YN02/1-704 SVDCTMYICGDSQECNKLLLQYGSFCAQLNRALSGVAVEQDKNTEA
GX-P2V/1-1270 SVDCTMYICGDSIECSNLLLQYGSFCTQLNRALTGIAVEQDKNTQE
GX-P1E/1-1266 SVDCTMYICGDSIECSNLLLQYGSFCTQLNRALTGIAVEQDKNTQE
GX-P5E/1-1268 SVDCTMYICGDSIECSNLLLQYGSFCTQLNRALTGIAVEQDKNTQE
GX-P4L/1-1268 SVDCTMYICGDSIECSNLLLQYGSFCTQLNRALTGIAVEQDKNTQE
GX-P5L/1-1268 SVDCTMYICGDSIECSNLLLQYGSFCTQLNRALTGIAVEQDKNTQE
Chicken/1-1154 QINCLQYVCGNSLDCRELFQQYGPVCDNILSVVNSVGKKEDMELLN
Duck/1-1192 TIDCARYVCGESLKCRTLLQQYGSFCTSVNNILAGVNDNEDNGMLS
Turkey/1-1227 TIDCAKYVCGDSTRCRTLLQQYGSFCQSVNAILSGVNENEDNGLLQ
Conservation
7 9 8 * 6 6 * 9 * * 7 * 6 6 * 6 5 * 8 7 * * * 7 7 * 4 8 9 6 4 7 9 7 8 9 7 3 5 8 * 5 4 7 5 4
Quality
Consensus
SVDCTMYICGDSIECSNLLLQYGSFCTQLNRALTGIAVEQDKNTQE
Occupancy

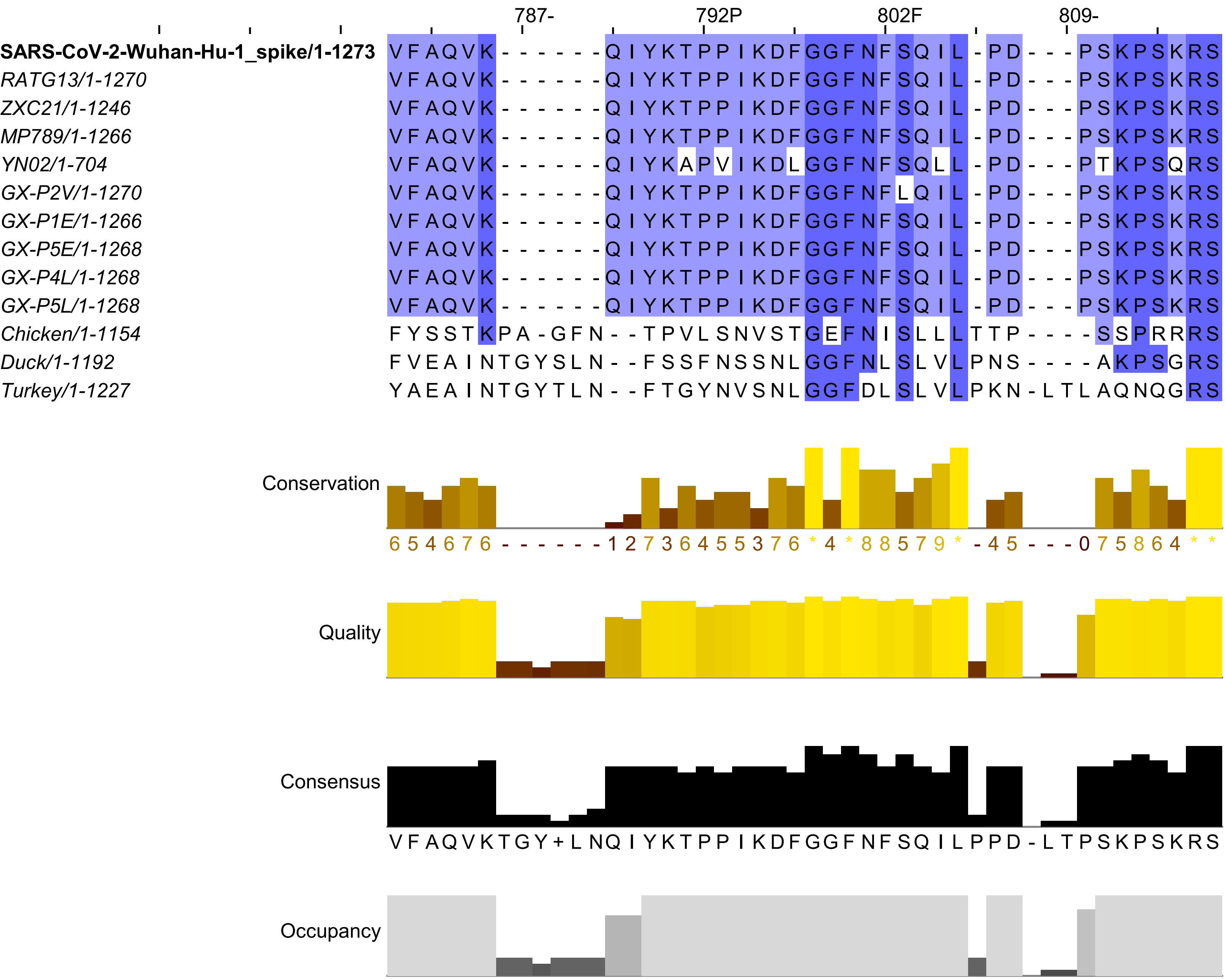
787-
792P
802F
809-
SARS-CoV-2-Wuhan-Hu-1_spike/1-1273 VFAQVK------QIYKTPPIKDFGGFNFSQIL-PD---PSKPSKRS
RATG13/1-1270 VFAQVK------QIYKTPPIKDFGGFNFSQIL-PD---PSKPSKRS
ZXC21/1-1246 VFAQVK------QIYKTPPIKDFGGFNFSQIL-PD---PSKPSKRS
MP789/1-1266 VFAQVK------QIYKTPPIKDFGGFNFSQIL-PD---PSKPSKRS
YN02/1-704 VFAQVK------QIYKAPVIKDLGGFNFSQLL-PD---PTKPSQRS
GX-P2V/1-1270 VFAQVK------QIYKTPPIKDFGGFNFLQIL-PD---PSKPSKRS
GX-P1E/1-1266 VFAQVK------QIYKTPPIKDFGGFNFSQIL-PD---PSKPSKRS
GX-P5E/1-1268 VFAQVK------QIYKTPPIKDFGGFNFSQIL-PD---PSKPSKRS
GX-P4L/1-1268 VFAQVK------QIYKTPPIKDFGGFNFSQIL-PD---PSKPSKRS
GX-P5L/1-1268 VFAQVK------QIYKTPPIKDFGGFNFSQIL-PD---PSKPSKRS
Chicken/1-1154 FYSSTKPA-GFN--TPVLSNVSTGEFNISLLLTTP----SSPRRRS
Duck/1-1192 FVEAINTGYSLN--FSSFNSSNLGGFNLSLVLPNS----AKPSGRS
Turkey/1-1227 YAEAINTGYTLN--FTGYNVSNLGGFDLSLVLPKN-LTLAQNQGRS
Conservation
654676------12736455376*4*88579*-45---075864**
Quality
Consensus
VFAQVKTGY+LNQIYKTPPIKDFGGFNFSQILPPD-LTPSKPSKRS
Occupancy

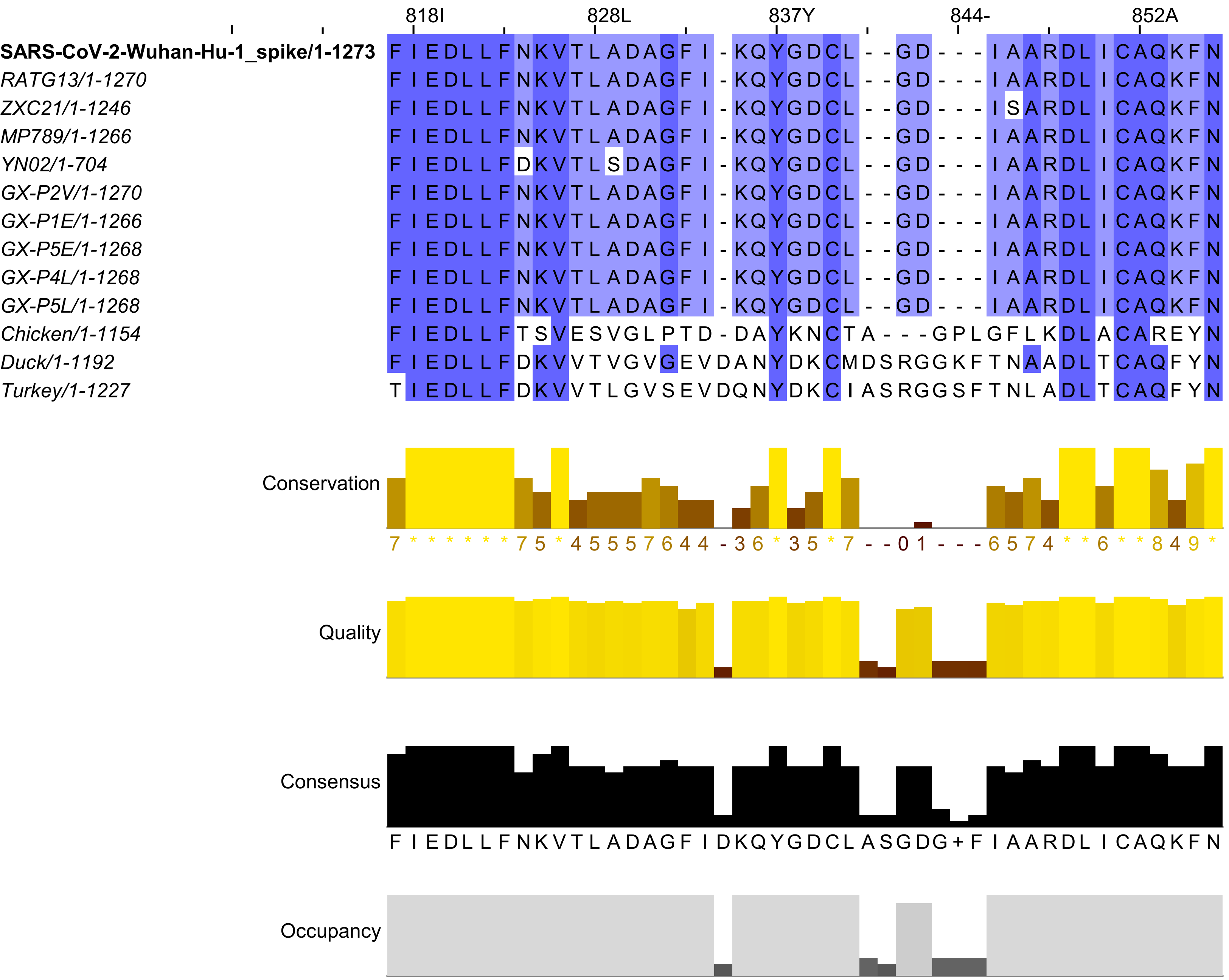
818I
828L
837Y
844-
852A
SARS-CoV-2-Wuhan-Hu-1_spike/1-1273 F I E D L L F N K V T L A D A G F I - K Q Y G D C L - - G D - - - I A A R D L I C A Q K F N
RATG13/1-1270 F I E D L L F N K V T L A D A G F I - K Q Y G D C L - - G D - - - I A A R D L I C A Q K F N
ZXC21/1-1246 F I E D L L F N K V T L A D A G F I - K Q Y G D C L - - G D - - - I S A R D L I C A Q K F N
MP789/1-1266 F I E D L L F N K V T L A D A G F I - K Q Y G D C L - - G D - - - I A A R D L I C A Q K F N
YN02/1-704 F I E D L L F D K V T L S D A G F I - K Q Y G D C L - - G D - - - I A A R D L I C A Q K F N
GX-P2V/1-1270 F I E D L L F N K V T L A D A G F I - K Q Y G D C L - - G D - - - I A A R D L I C A Q K F N
GX-P1E/1-1266 F I E D L L F N K V T L A D A G F I - K Q Y G D C L - - G D - - - I A A R D L I C A Q K F N
GX-P5E/1-1268 F I E D L L F N K V T L A D A G F I - K Q Y G D C L - - G D - - - I A A R D L I C A Q K F N
GX-P4L/1-1268 F I E D L L F N K V T L A D A G F I - K Q Y G D C L - - G D - - - I A A R D L I C A Q K F N
GX-P5L/1-1268 F I E D L L F N K V T L A D A G F I - K Q Y G D C L - - G D - - - I A A R D L I C A Q K F N
Chicken/1-1154 F I E D L L F T S V E S V G L P T D - D A Y K N C T A - - - G P L G F L K D L A C A R E Y N
Duck/1-1192 F I E D L L F D K V V T V G V G E V D A N Y D K C M D S R G G K F T N A A D L T C A Q F Y N
Turkey/1-1227 T I E D L L F D K V V T L G V S E V D Q N Y D K C I A S R G G S F T N L A D L T C A Q F Y N
Conservation
7 * * * * * * 7 5 * 4 5 5 5 7 6 4 4 - 3 6 * 3 5 * 7 - - 0 1 - - - 6 5 7 4 * * 6 * * 8 4 9 *
Quality
Consensus
F I E D L L F N K V T L A D A G F I D K Q Y G D C L A S G D G + F I A A R D L I C A Q K F N
Occupancy

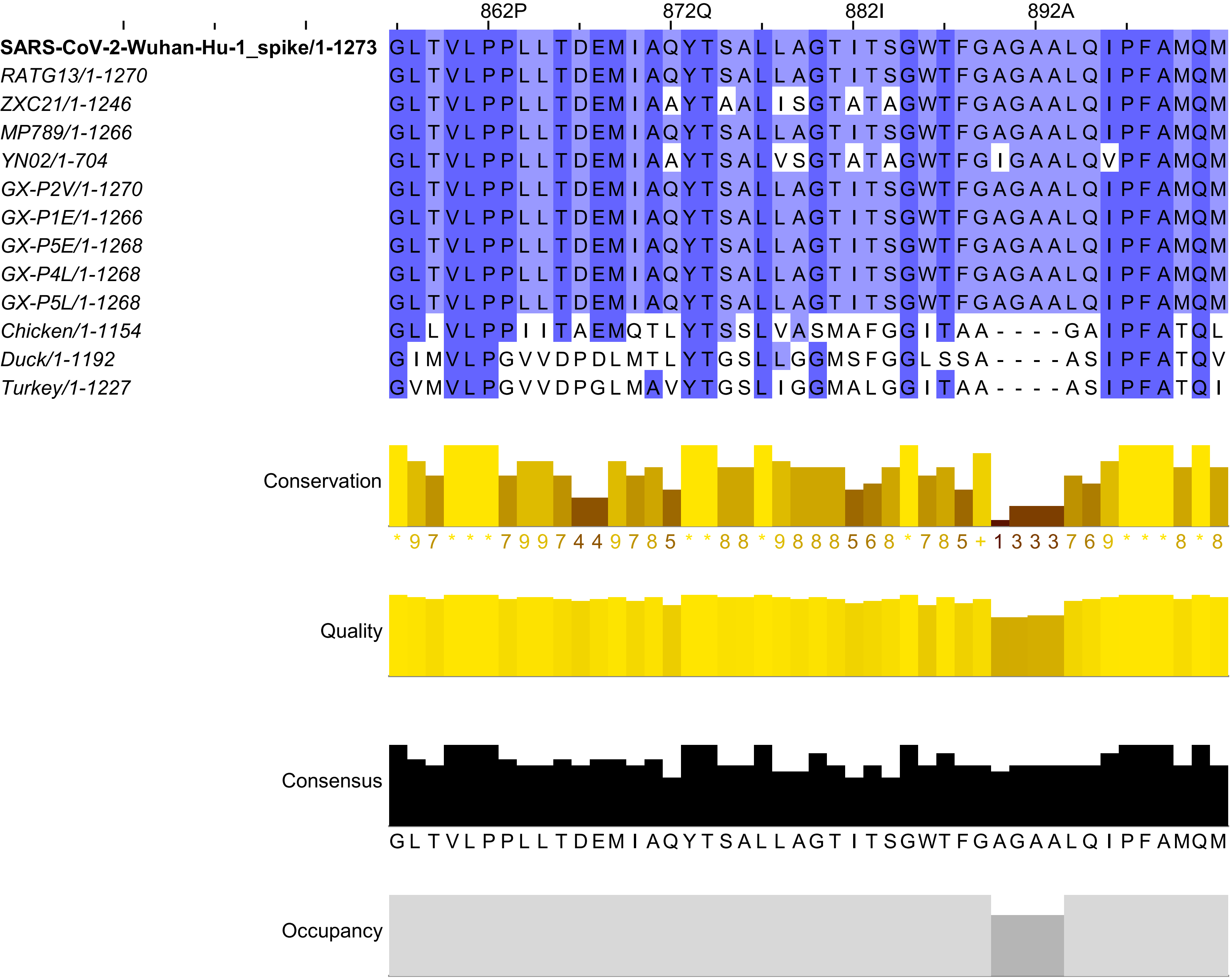
862P
872Q
882I
892A
SARS-CoV-2-Wuhan-Hu-1_spike/1-1273 GLTVLPPLLTDEMIAQYTSALLAGTITSGWTFGAGAALQIPFAMQM
RATG13/1-1270 GLTVLPPLLTDEMIAQYTSALLAGTITSGWTFGAGAALQIPFAMQM
ZXC21/1-1246 GLTVLPPLLTDEMIAAYTAALISGTATAGWTFGAGAALQIPFAMQM
MP789/1-1266 GLTVLPPLLTDEMIAQYTSALLAGTITSGWTFGAGAALQIPFAMQM
YN02/1-704 GLTVLPPLLTDEMIAAYTSALVSGTATAGWTFGIGAALQVPFAMQM
GX-P2V/1-1270 GLTVLPPLLTDEMIAQYTSALLAGTITSGWTFGAGAALQIPFAMQM
GX-P1E/1-1266 GLTVLPPLLTDEMIAQYTSALLAGTITSGWTFGAGAALQIPFAMQM
GX-P5E/1-1268 GLTVLPPLLTDEMIAQYTSALLAGTITSGWTFGAGAALQIPFAMQM
GX-P4L/1-1268 GLTVLPPLLTDEMIAQYTSALLAGTITSGWTFGAGAALQIPFAMQM
GX-P5L/1-1268 GLTVLPPLLTDEMIAQYTSALLAGTITSGWTFGAGAALQIPFAMQM
Chicken/1-1154 GLLVLPPIITAEMQTLYTSSLVASMAFGGITAA----GAIPFATQL
Duck/1-1192 GIMVLPGVVDPDLMTLYTGSLLGGMSFGGLSSA----ASIPFATQV
Turkey/1-1227 GVMVLPGVVDPGLMAVYTGSLIGGMALGGITAA----ASIPFATQI
Conservation
*97***7997449785**88*98885 68*785+1333769***8*8
Quality
Consensus
GLTVLPPLLTDEMIAQYTSALLAGTITSGWTFGAGAALQIPFAMQM
Occupancy

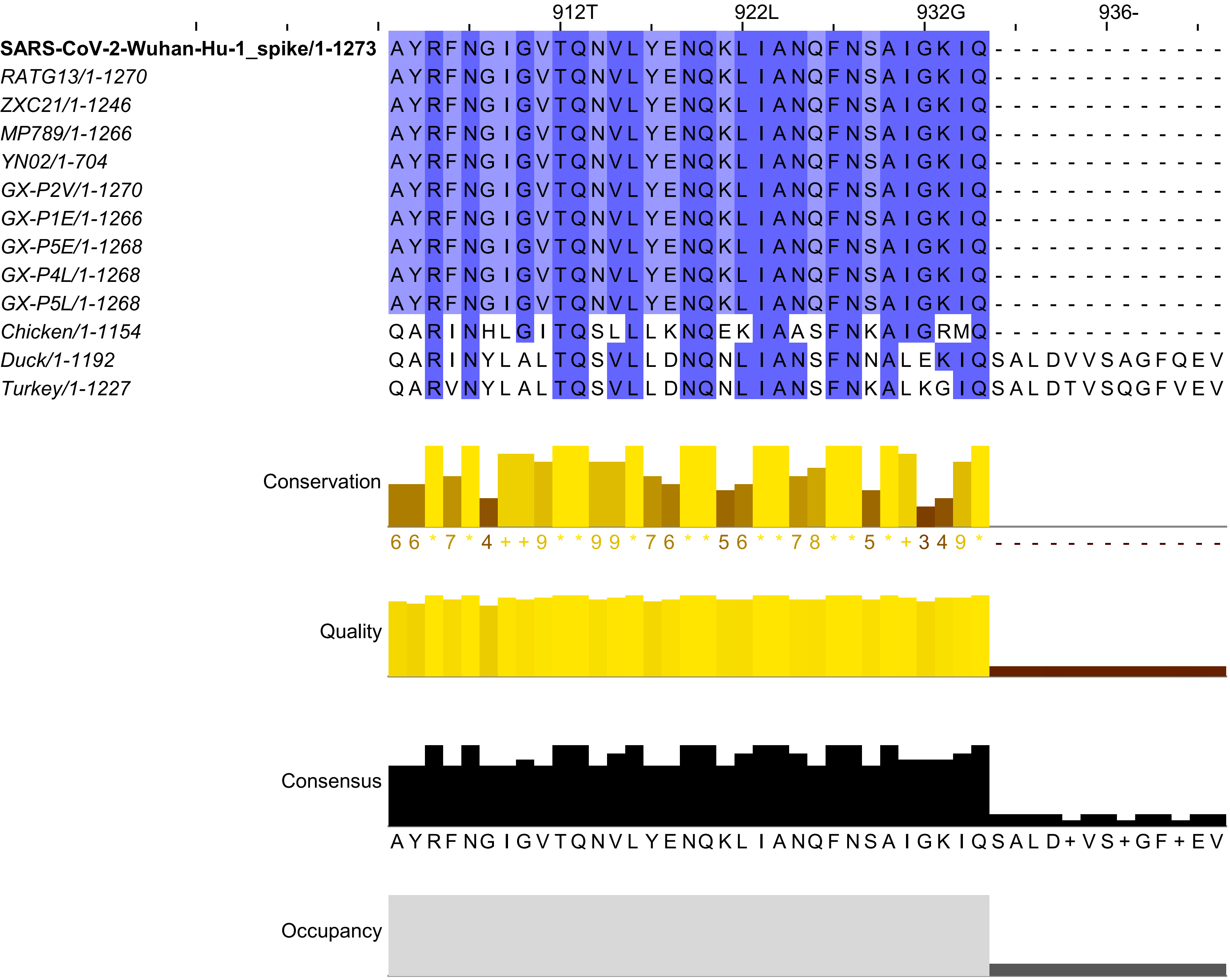
912T
922L
932G
936-
SARS-CoV-2-Wuhan-Hu-1_spike/1-1273 AYRFNGIGVTQNVLYENQKLIANQFNSAIGKIQ-------------
RATG13/1-1270 AYRFNGIGVTQNVLYENQKLIANQFNSAIGKIQ-------------
ZXC21/1-1246 AYRFNGIGVTQNVLYENQKLIANQFNSAIGKIQ-------------
MP789/1-1266 AYRFNGIGVTQNVLYENQKLIANQFNSAIGKIQ-------------
YN02/1-704 AYRFNGIGVTQNVLYENQKLIANQFNSAIGKIQ-------------
GX-P2V/1-1270 AYRFNGIGVTQNVLYENQKLIANQFNSAIGKIQ-------------
GX-P1E/1-1266 AYRFNGIGVTQNVLYENQKLIANQFNSAIGKIQ-------------
GX-P5E/1-1268 AYRFNGIGVTQNVLYENQKLIANQFNSAIGKIQ-------------
GX-P4L/1-1268 AYRFNGIGVTQNVLYENQKLIANQFNSAIGKIQ-------------
GX-P5L/1-1268 AYRFNGIGVTQNVLYENQKLIANQFNSAIGKIQ-------------
Chicken/1-1154 QARINHLGITQSLLLKNQEKIAASFNKAIGRMQ-------------
Duck/1-1192 QARINYLALTQSVLLDNQNLIANSFNNALEKIQSALDVVSAGFQEV
Turkey/1-1227 QARVNYLALTQSVLLDNQNLIANSFNKALKGIQSALDTVSQGFVEV
Conservation
66*7*4++9**99*76**56**78**5*+349*-------------
Quality
Consensus
AYRFNGIGVTQNVLYENQKLIANQFNSAIGKIQSALD+VS+GF+EV
Occupancy

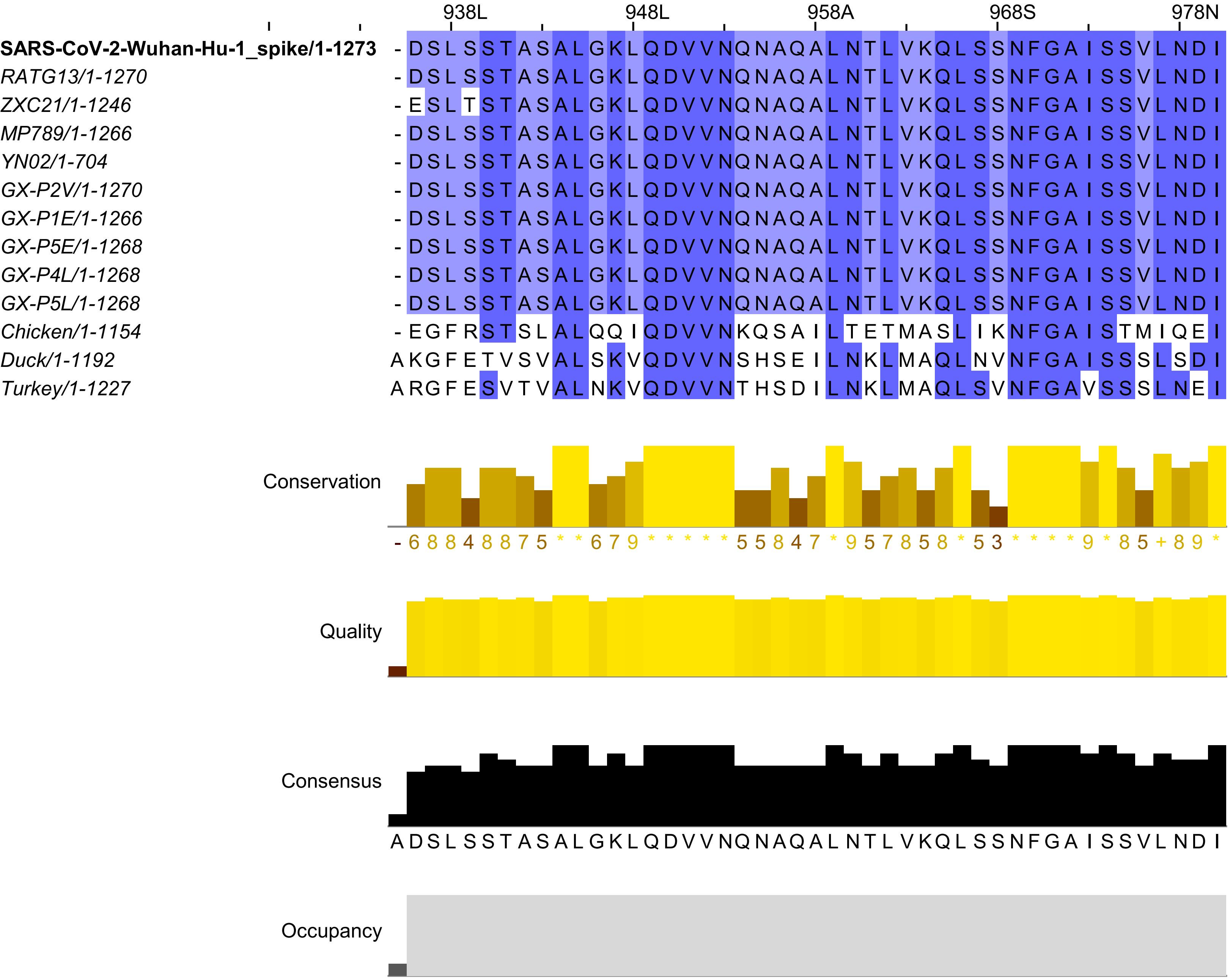
938L 948L 958A 968S 978N
SARS-CoV-2-Wuhan-Hu-1_spike/1-1273 -DSLSSTASALGKLQDVVNQNAQALNTLVKQLSSNFGAISSVLNDI
RATG13/1-1270 -DSLSSTASALGKLQDVVNQNAQALNTLVKQLSSNFGAISSVLNDI
ZXC21/1-1246 -ESLTSTASALGKLQDVVNQNAQALNTLVKQLSSNFGAISSVLNDI
MP789/1-1266 -DSLSSTASALGKLQDVVNQNAQALNTLVKQLSSNFGAISSVLNDI
YN02/1-704 -DSLSSTASALGKLQDVVNQNAQALNTLVKQLSSNFGAISSVLNDI
GX-P2V/1-1270 -DSLSSTASALGKLQDVVNQNAQALNTLVKQLSSNFGAISSVLNDI
GX-P1E/1-1266 -DSLSSTASALGKLQDVVNQNAQALNTLVKQLSSNFGAISSVLNDI
GX-P5E/1-1268 -DSLSSTASALGKLQDVVNQNAQALNTLVKQLSSNFGAISSVLNDI
GX-P4L/1-1268 -DSLSSTASALGKLQDVVNQNAQALNTLVKQLSSNFGAISSVLNDI
GX-P5L/1-1268 -DSLSSTASALGKLQDVVNQNAQALNTLVKQLSSNFGAISSVLNDI
Chicken/1-1154 -EGFRSTSLALQQIQDVVNKQSAILTETMASLIKNFGAISTMIQEI
Duck/1-1192 AKGFETVSVALSKVQDVVNSHSEILNKLMAQLNVNFGAISSSLSDI
Turkey/1-1227 ARGFESVTVALNKVQDVVNTHSDILNKLMAQLSVNFGAVSSSLNEI
Conservation
- 6 8 8 4 8 8 7 5 * * 6 7 9 * * * * * 5 5 8 4 7 * 9 5 7 8 5 8 * 5 3 * * * * 9 * 8 5 + 8 9 *
Quality
Consensus
ADSLSSTASALGKLQDVVNQNAQALNTLVKQLSSNFGAISSVLNDI
Occupancy

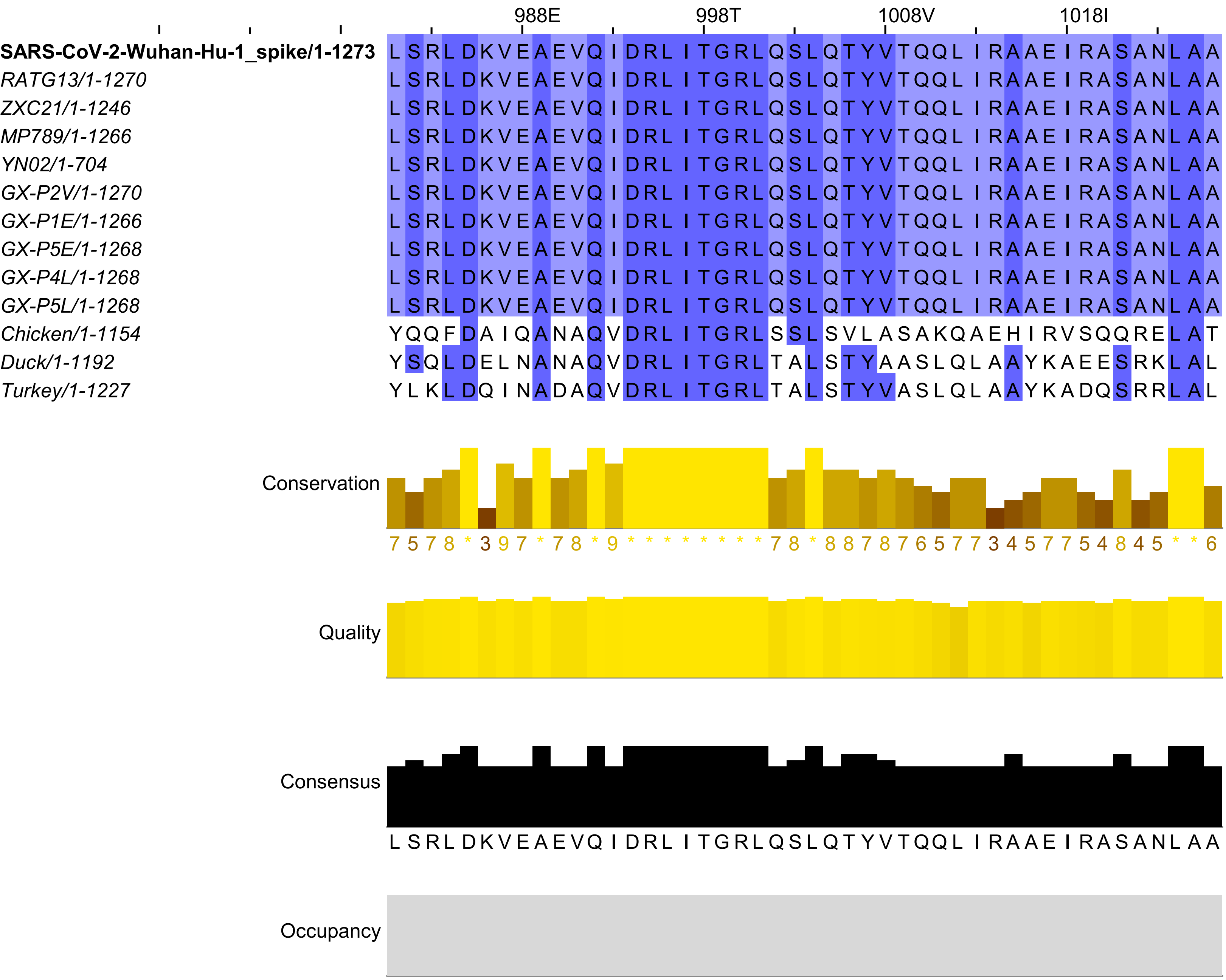

988E
998T
1008V
1018I
SARS-CoV-2-Wuhan-Hu-1_spike/1-1273 LSRLDKVEAEVQIDRLITGRLQSLQTYVTQQLIRAAEIRASANLAA
RATG13/1-1270 LSRLDKVEAEVQIDRLITGRLQSLQTYVTQQLIRAAEIRASANLAA
ZXC21/1-1246 LSRLDKVEAEVQIDRLITGRLQSLQTYVTQQLIRAAEIRASANLAA
MP789/1-1266 LSRLDKVEAEVQIDRLITGRLQSLQTYVTQQLIRAAEIRASANLAA
YN02/1-704 LSRLDKVEAEVQIDRLITGRLQSLQTYVTQQLIRAAEIRASANLAA
GX-P2V/1-1270 LSRLDKVEAEVQIDRLITGRLQSLQTYVTQQLIRAAEIRASANLAA
GX-P1E/1-1266 LSRLDKVEAEVQIDRLITGRLQSLQTYVTQQLIRAAEIRASANLAA
GX-P5E/1-1268 LSRLDKVEAEVQIDRLITGRLQSLQTYVTQQLIRAAEIRASANLAA
GX-P4L/1-1268 LSRLDKVEAEVQIDRLITGRLQSLQTYVTQQLIRAAEIRASANLAA
GX-P5L/1-1268 LSRLDKVEAEVQIDRLITGRLQSLQTYVTQQLIRAAEIRASANLAA
Chicken/1-1154 YQQFDAIQANAQVDRLITGRLSSLSVLASAKQAEHIRVSQQRELAT
Duck/1-1192 YSQLDELNANAQVDRLITGRLTALSTYAASLQLAAYKAEESRKLAL
Turkey/1-1227 YLKLDQINADAQVDRLITGRLTALSTYVASLQLAAYKADQSRRLAL
Conservation
7578*397*78*9********78*887876577345775484 5**6
Quality
Consensus
LSRLDKVEAEVQIDRLITGRLQSLQTYVTQQLIRAAEIRASANLAA
Occupancy

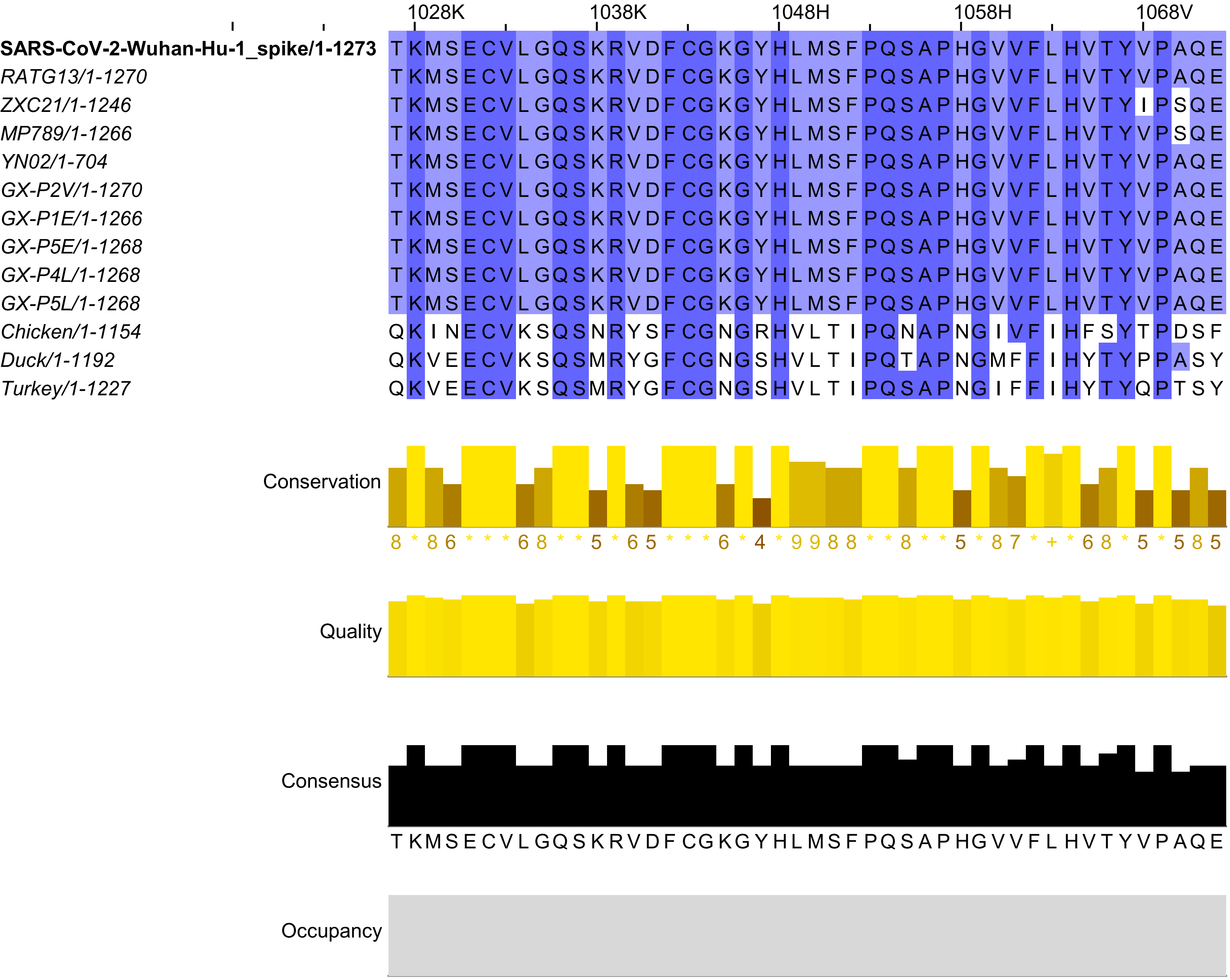
1028K 1038K 1048H 1058H 1068V
SARS-CoV-2-Wuhan-Hu-1_spike/1-1273 TKMSECVLGQSKRVDFCGKGYHLMSFPQSAPHGVVFLHVTYVPAQE
RATG13/1-1270 TKMSECVLGQSKRVDFCGKGYHLMSFPQSAPHGVVFLHVTYVPAQE
ZXC21/1-1246 TKMSECVLGQSKRVDFCGKGYHLMSFPQSAPHGVVFLHVTYIPSQE
MP789/1-1266 TKMSECVLGQSKRVDFCGKGYHLMSFPQSAPHGVVFLHVTYVPSQE
YN02/1-704 TKMSECVLGQSKRVDFCGKGYHLMSFPQSAPHGVVFLHVTYVPAQE
GX-P2V/1-1270 TKMSECVLGQSKRVDFCGKGYHLMSFPQSAPHGVVFLHVTYVPAQE
GX-P1E/1-1266 TKMSECVLGQSKRVDFCGKGYHLMSFPQSAPHGVVFLHVTYVPAQE
GX-P5E/1-1268 TKMSECVLGQSKRVDFCGKGYHLMSFPQSAPHGVVFLHVTYVPAQE
GX-P4L/1-1268 TKMSECVLGQSKRVDFCGKGYHLMSFPQSAPHGVVFLHVTYVPAQE
GX-P5L/1-1268 TKMSECVLGQSKRVDFCGKGYHLMSFPQSAPHGVVFLHVTYVPAQE
Chicken/1-1154 QKINECVKSQSNRYSFCGNGRHVLTIPQNAPNGIVFIHFSYTPDSF
Duck/1-1192 QKVEECVKSQSMRYGFCGNGSHVLTIPQTAPNGMFFIHYTYPPASY
Turkey/1-1227 QKVEECVKSQSMRYGFCGNGSHVLTIPQSAPNGIFFIHYTYQPTSY
Conservation
8 * 8 6 * * * 6 8 * * 5 * 6 5 * * * 6 * 4 * 9 9 8 8 * * 8 * * 5 * 8 7 * + * 6 8 * 5 * 5 8 5
Quality
Consensus
TKMSECVLGQSKRVDFCGKGYHLMSFPQSAPHGVVFLHVTYVPAQE
Occupancy

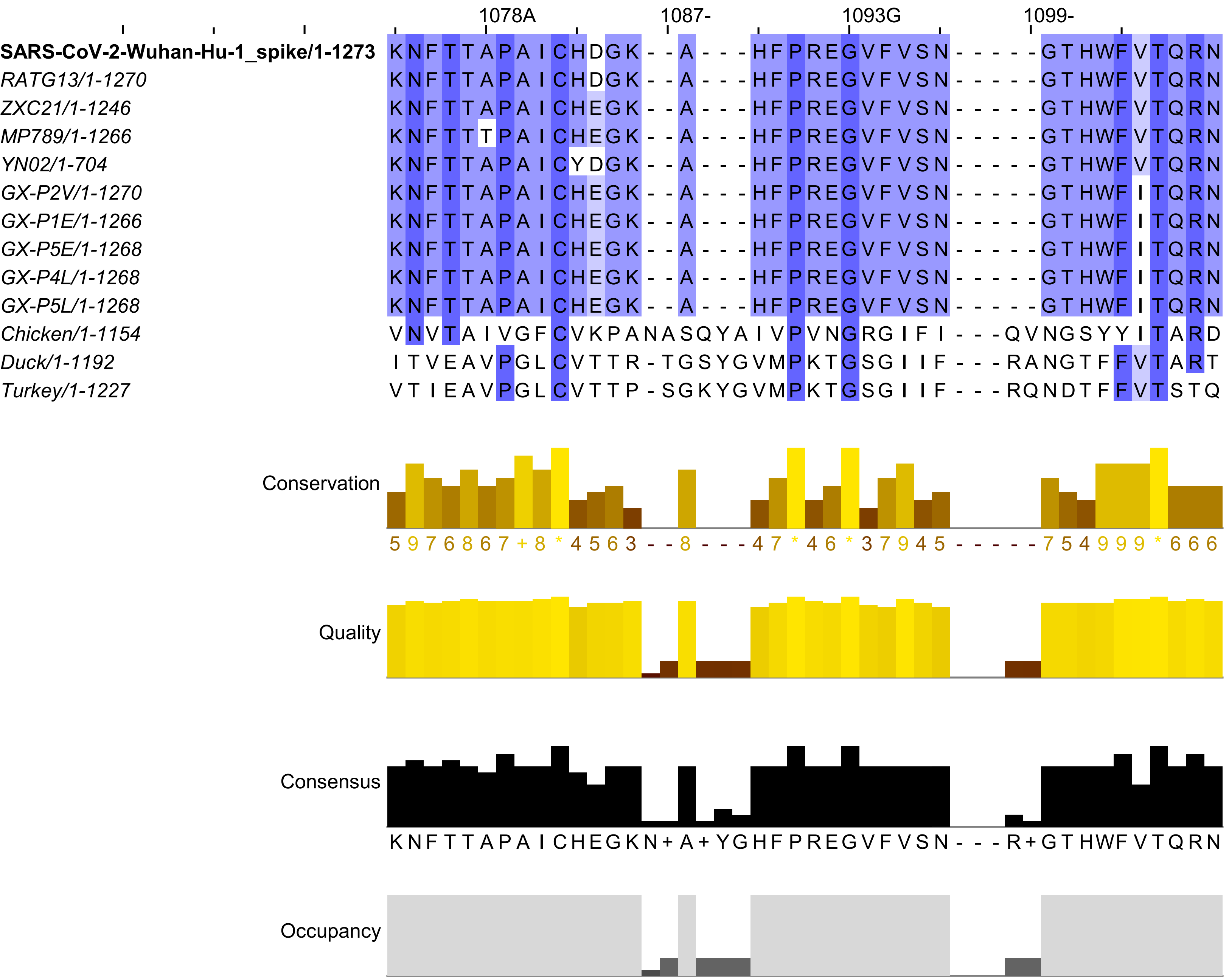

1078A
1087-
1093G
1099-
SARS-CoV-2-Wuhan-Hu-1_spike/1-1273
RATG13/1-1270
ZXC21/1-1246
MP789/1-1266
YN02/1-704
GX-P2V/1-1270
GX-P1E/1-1266
GX-P5E/1-1268
GX-P4L/1-1268
GX-P5L/1-1268
Chicken/1-1154
Duck/1-1192
Turkey/1-1227
Conservation
Quality
Consensus
Occupancy

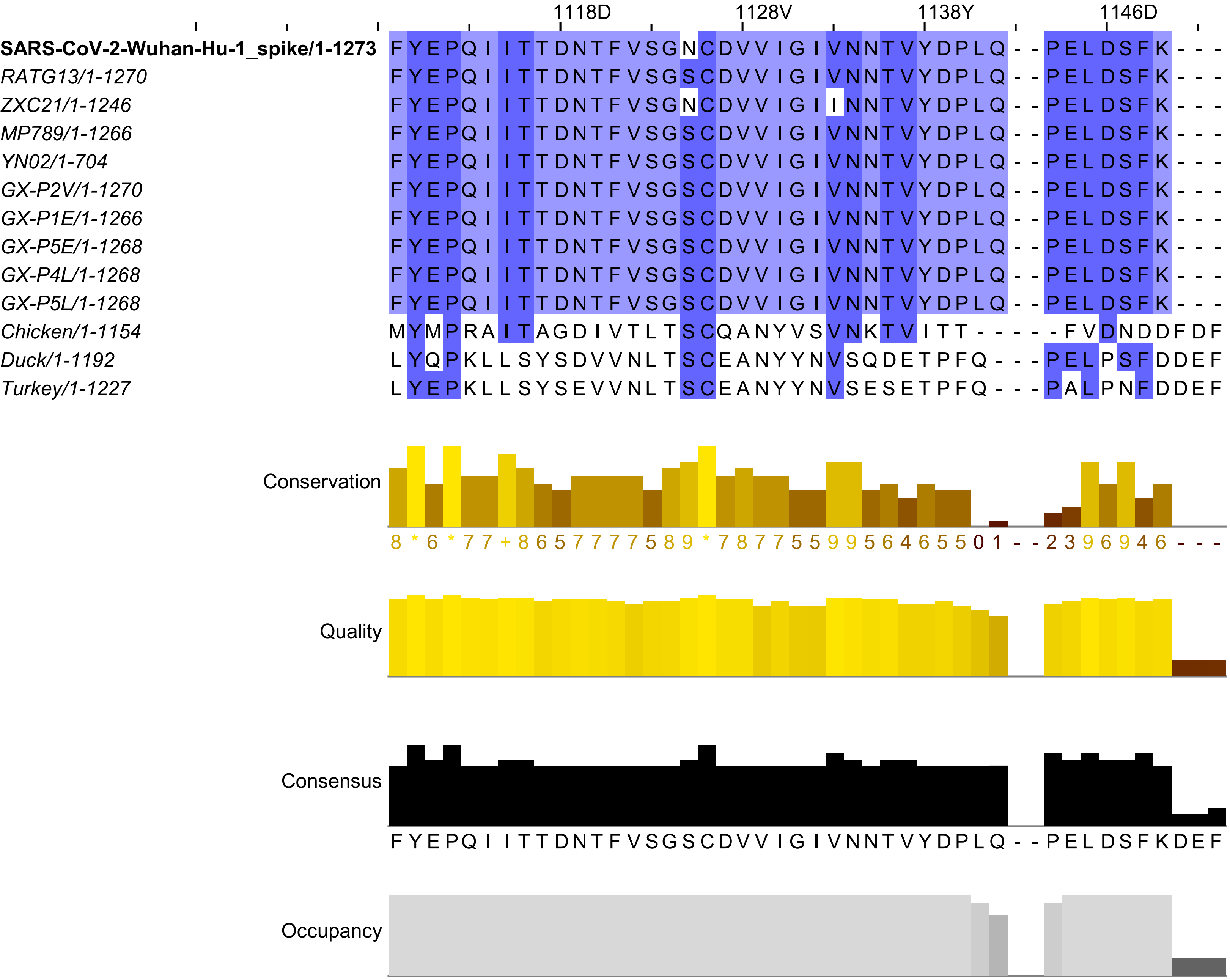

1118D
1128V
1138Y
1146D
SARS-CoV-2-Wuhan-Hu-1_spike/1-1273 FYEPQIITTDNTFVSGNCDVVIGIVNNTVYDPLQ--PELDSFK---
RATG13/1-1270 FYEPQIITTDNTFVSGSCDVVIGIVNNTVYDPLQ--PELDSFK---
ZXC21/1-1246 FYEPQIITTDNTFVSGNCDVVIGIINNTVYDPLQ--PELDSFK---
MP789/1-1266 FYEPQIITTDNTFVSGSCDVVIGIVNNTVYDPLQ--PELDSFK---
YN02/1-704 FYEPQIITTDNTFVSGSCDVVIGIVNNTVYDPLQ--PELDSFK---
GX-P2V/1-1270 FYEPQIITTDNTFVSGSCDVVIGIVNNTVYDPLQ--PELDSFK---
GX-P1E/1-1266 FYEPQIITTDNTFVSGSCDVVIGIVNNTVYDPLQ--PELDSFK---
GX-P5E/1-1268 FYEPQIITTDNTFVSGSCDVVIGIVNNTVYDPLQ--PELDSFK---
GX-P4L/1-1268 FYEPQIITTDNTFVSGSCDVVIGIVNNTVYDPLQ--PELDSFK---
GX-P5L/1-1268 FYEPQIITTDNTFVSGSCDVVIGIVNNTVYDPLQ--PELDSFK---
Chicken/1-1154 MYMPRAITAGDIVTLTSCQANYVSVNKTVITT-----FVDNDDFDF
Duck/1-1192 LYQPKLLSYSDVVNLTSCEANYYNVSQDETPFQ---PELPSFDDEF
Turkey/1-1227 LYEPKLLSYSEVVNLTSCEANYYNVSESETPFQ---PALPNFDDEF
Conservation
8*6*77+8657777589*787755995646550 1--2396946---
Quality
Consensus
FYEPQIITTDNTFVSGSCDVVIGIVNNTVYDPLQ--PELDSFKDEF
Occupancy

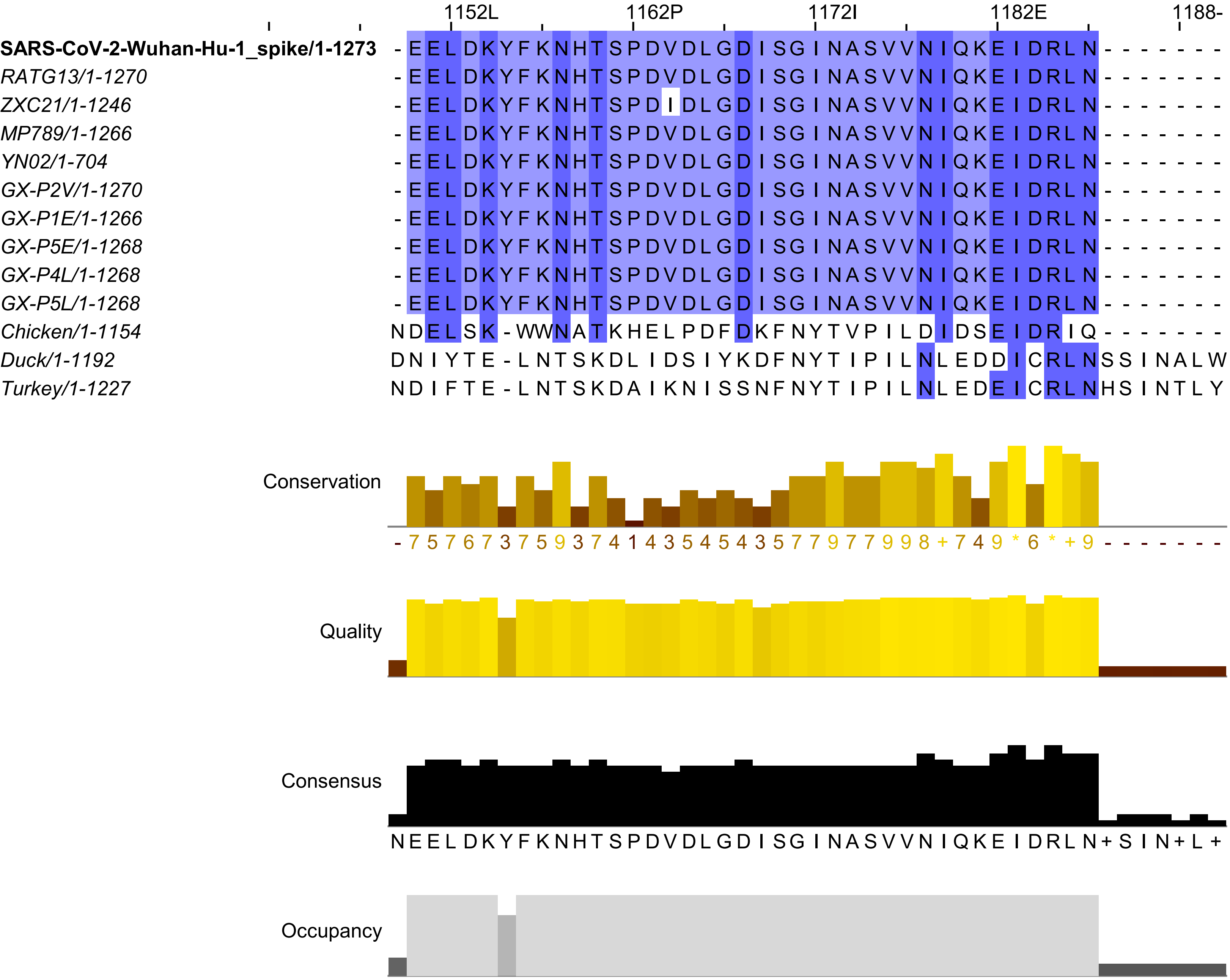

1152L
1162P
1172I
1182E
1188-
SARS-CoV-2-Wuhan-Hu-1_spike/1-1273 -EELDKYFKNHTSPDVDLGDISGINASVVNIQKEIDRLN--------
RATG13/1-1270 -EELDKYFKNHTSPDVDLGDISGINASVVNIQKEIDRLN--------
ZXC21/1-1246 -EELDKYFKNHTSPDIDLGDISGINASVVNIQKEIDRLN--------
MP789/1-1266 -EELDKYFKNHTSPDVDLGDISGINASVVNIQKEIDRLN--------
YN02/1-704 -EELDKYFKNHTSPDVDLGDISGINASVVNIQKEIDRLN--------
GX-P2V/1-1270 -EELDKYFKNHTSPDVDLGDISGINASVVNIQKEIDRLN--------
GX-P1E/1-1266 -EELDKYFKNHTSPDVDLGDISGINASVVNIQKEIDRLN--------
GX-P5E/1-1268 -EELDKYFKNHTSPDVDLGDISGINASVVNIQKEIDRLN--------
GX-P4L/1-1268 -EELDKYFKNHTSPDVDLGDISGINASVVNIQKEIDRLN--------
GX-P5L/1-1268 -EELDKYFKNHTSPDVDLGDISGINASVVNIQKEIDRLN--------
Chicken/1-1154 NDELSK-WWNATKHELPDFDKFNYTVPILDIDSEIDRIQ--------
Duck/1-1192 DNIYTE-LNTSKDLIDSIYKDFNYTIPILNLEDDICRLNSSINALW
Turkey/1-1227 NDIFTE-LNTSKDAIKNISSNFNYTIPILNLEDEICRLNHSINTLY
Conservation
-757673759374143545435779779 98+749*6*+9--------
Quality
Consensus
NEELDKYFKNHTSPDVDLGDISGINASVVNIQKEIDRLN+SIN+L+
Occupancy

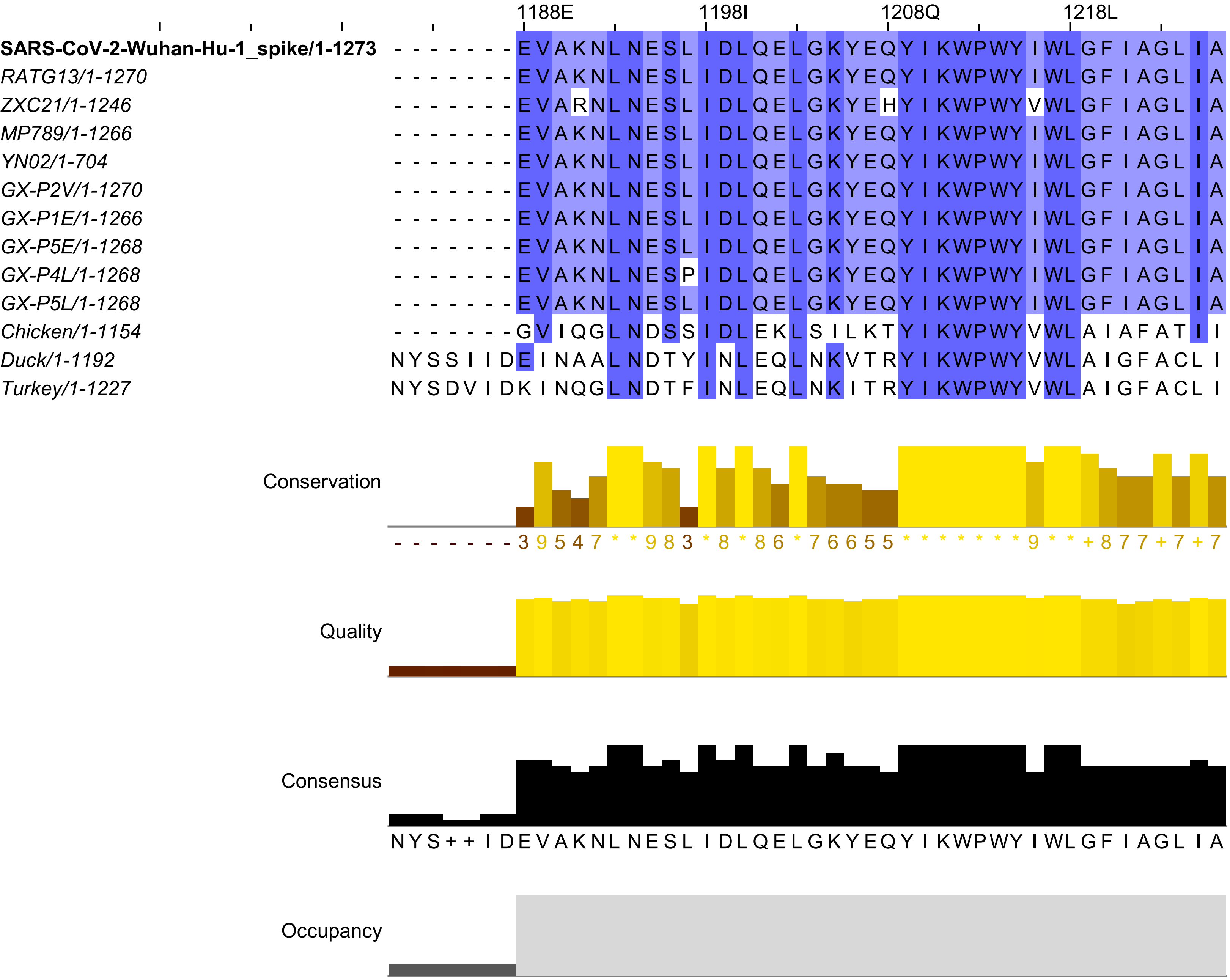
1188E
1198I
1208Q
1218L
SARS-CoV-2-Wuhan-Hu-1_spike/1-1273 -------EVAKNLNESLIDLQELGKYEQYIKWPWYIWLGFIAGLIA
RATG13/1-1270 -------EVAKNLNESLIDLQELGKYEQYIKWPWYIWLGFIAGLIA
ZXC21/1-1246 -------EVARNLNESLIDLQELGKYEHYIKWPWYVWLGFIAGLIA
MP789/1-1266 -------EVAKNLNESLIDLQELGKYEQYIKWPWYIWLGFIAGLIA
YN02/1-704 -------EVAKNLNESLIDLQELGKYEQYIKWPWYIWLGFIAGLIA
GX-P2V/1-1270 -------EVAKNLNESLIDLQELGKYEQYIKWPWYIWLGFIAGLIA
GX-P1E/1-1266 -------EVAKNLNESLIDLQELGKYEQYIKWPWYIWLGFIAGLIA
GX-P5E/1-1268 -------EVAKNLNESLIDLQELGKYEQYIKWPWYIWLGFIAGLIA
GX-P4L/1-1268 -------EVAKNLNESPIDLQELGKYEQYIKWPWYIWLGFIAGLIA
GX-P5L/1-1268 -------EVAKNLNESLIDLQELGKYEQYIKWPWYIWLGFIAGLIA
Chicken/1-1154 -------GVIQGLNDSSIDLEKLSILKTYIKWPWYVWLAIAFATII
Duck/1-1192 NYSSIIDEINAALNDTYINLEQLNKVTRYIKWPWYVWLAIGFACLI
Turkey/1-1227 NYSDVIDKINQGLNDTFINLEQLNKITRYIKWPWYVWLAIGFACLI
Conservation
-------3954 7**983*8*86*76655*******9**+877+7+7
Quality
Consensus
NYS++IDEVAKNLNESLIDLQELGKYEQYIKWPWYIWLGFIAGLIA
Occupancy

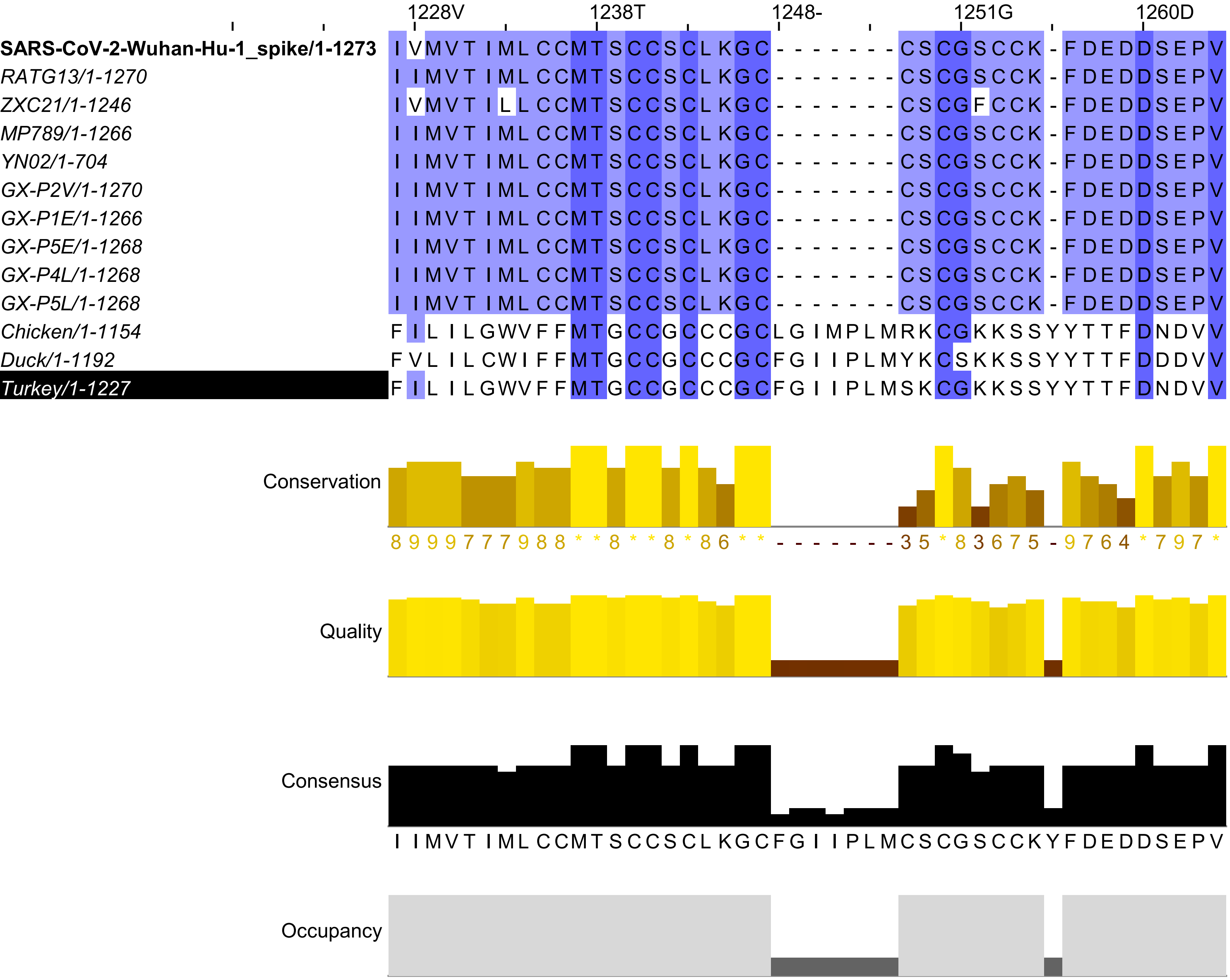

1228V
1238T
1248-
1251G
1260D
SARS-CoV-2-Wuhan-Hu-1_spike/1-1273 IVMVTIMLCCMTSCCSCLKGC-------CSCGSCCK-FDEDDSEPV
RATG13/1-1270 IIMVTIMLCCMTSCCSCLKGC-------CSCGSCCK-FDEDDSEPV
ZXC21/1-1246 IVMVTILLCCMTSCCSCLKGC-------CSCGFCCK-FDEDDSEPV
MP789/1-1266 IIMVTIMLCCMTSCCSCLKGC-------CSCGSCCK-FDEDDSEPV
YN02/1-704 IIMVTIMLCCMTSCCSCLKGC-------CSCGSCCK-FDEDDSEPV
GX-P2V/1-1270 IIMVTIMLCCMTSCCSCLKGC-------CSCGSCCK-FDEDDSEPV
GX-P1E/1-1266 IIMVTIMLCCMTSCCSCLKGC-------CSCGSCCK-FDEDDSEPV
GX-P5E/1-1268 IIMVTIMLCCMTSCCSCLKGC-------CSCGSCCK-FDEDDSEPV
GX-P4L/1-1268 IIMVTIMLCCMTSCCSCLKGC-------CSCGSCCK-FDEDDSEPV
GX-P5L/1-1268 IIMVTIMLCCMTSCCSCLKGC-------CSCGSCCK-FDEDDSEPV
Chicken/1-1154 FILILGWVFFMTGCCGCCCGCLGIMPLMRKCGKKSSYYTTFDNDVV
Duck/1-1192 FVLILCWIFFMTGCCGCCCGCFGIIPLMYKCSKKSSYYTTFDDDVV
Turkey/1-1227 FILILGWVFFMTGCCGCCCGCFGIIPLMSKCGKKSSYYTTFDNDVV
Conservation
8999777988**8**8*86**-------35*83675-9764*797*
Quality
Consensus
IIMVTIMLCCMTSCCSCLKGCFGIIPLMCSCGSCCKYFDEDDSEPV
Occupancy

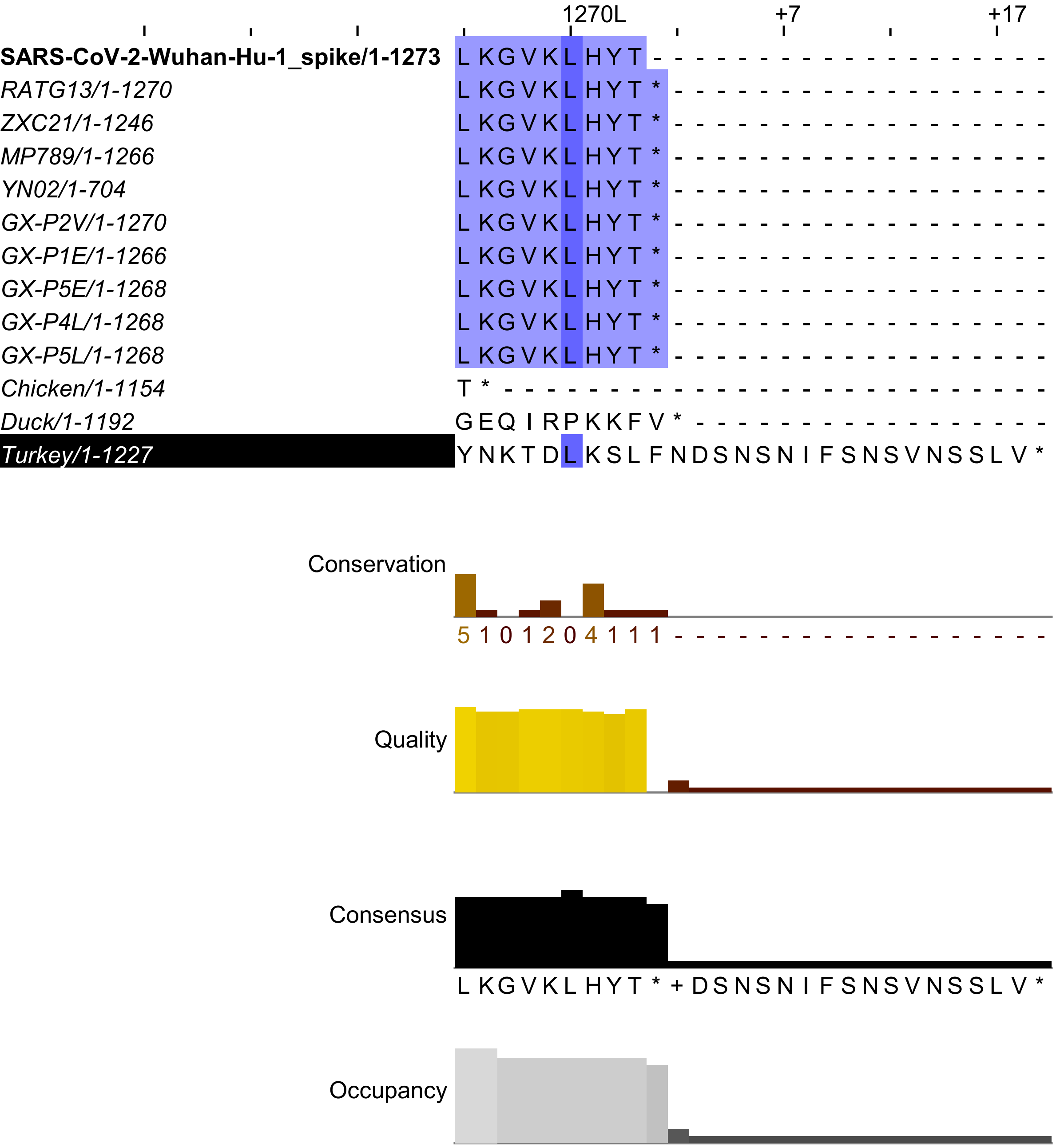
1270L
+7
+17
SARS-CoV-2-Wuhan-Hu-1_spike/1-1273 L K G V K L H Y T -
RATG13/1-1270 L K G V K L H Y T *
ZXC21/1-1246 L K G V K L H Y T *
MP789/1-1266 L K G V K L H Y T *
YN02/1-704 L K G V K L H Y T *
GX-P2V/1-1270 L K G V K L H Y T *
GX-P1E/1-1266 L K G V K L H Y T *
GX-P5E/1-1268 L K G V K L H Y T *
GX-P4L/1-1268 L K G V K L H Y T *
GX-P5L/1-1268 L K G V K L H Y T *
Chicken/1-1154 T *
Duck/1-1192 G E Q I R P K K F V *
Turkey/1-1227 Y N K T D L K S L F N D S N S N I F S N S V N S S L V *
Conservation
5 1 0 1 2 0 4 1 1 1
Quality
Consensus
L K G V K L H Y T * + D S N S N I F S N S V N S S L V *
Occupancy
