# Supplementary material for: An Immunoinformatic Approach for Identifying and Designing Conserved Multi-Epitope Vaccines for Coronaviruses
Source: Biomedicines. 2024 Nov 5;12(11):2530. doi: 10.3390/biomedicines12112530 (PMC11592158; doi:10.3390/biomedicines12112530)
Supplement: Supplementary file 1 [file biomedicines-12-02530-s001.zip › Supplementary Figure S2. hCoVs alignment.pdf]

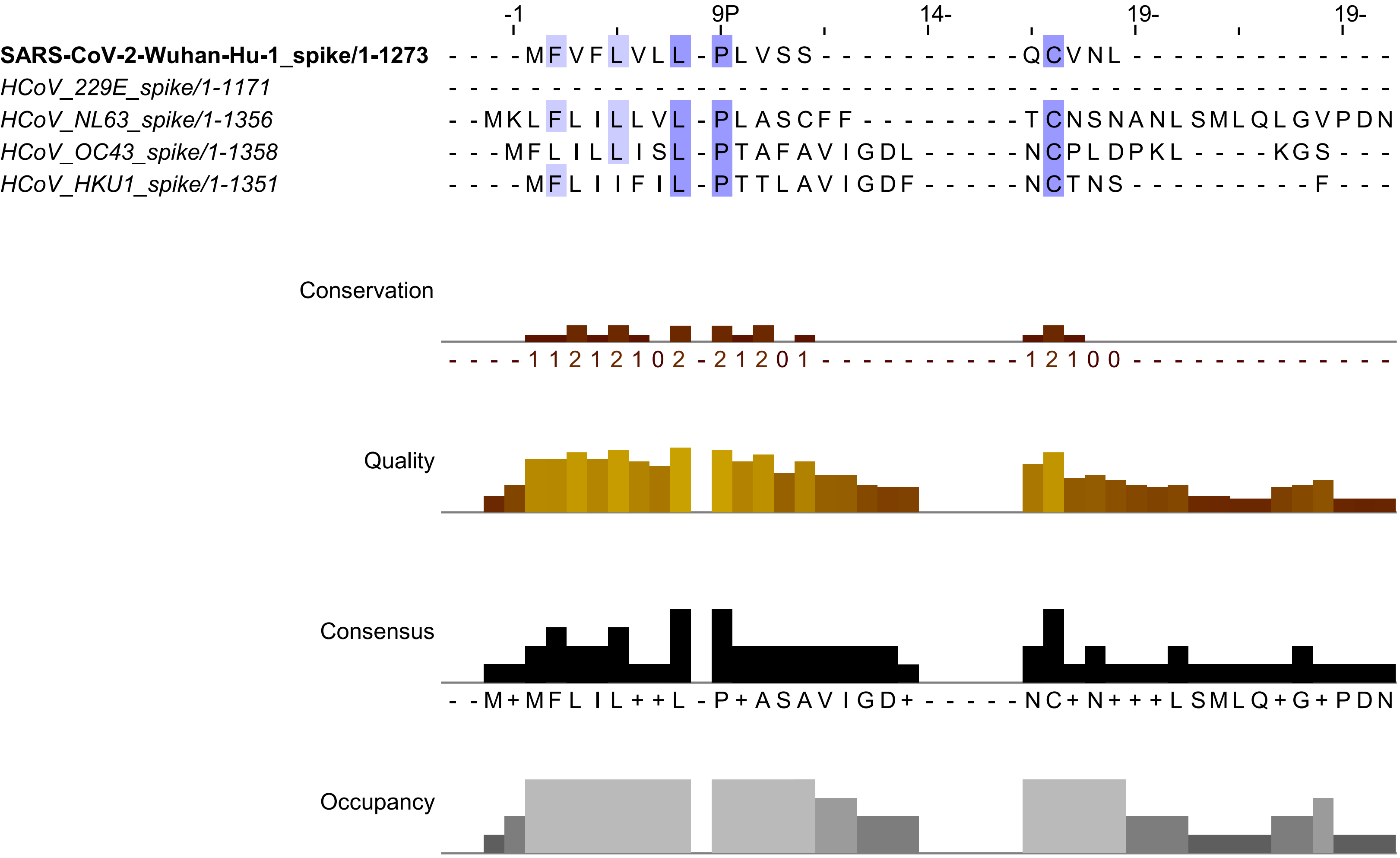

-1
9P
14-
19-
19-
SARS-CoV-2-Wuhan-Hu-1_spike/1-1273
HCoV_229E_spike/1-1171
HCoV_NL63_spike/1-1356
HCoV_OC43_spike/1-1358
HCoV_HKU1_spike/1-1351
Conservation
Quality
Consensus
Occupancy

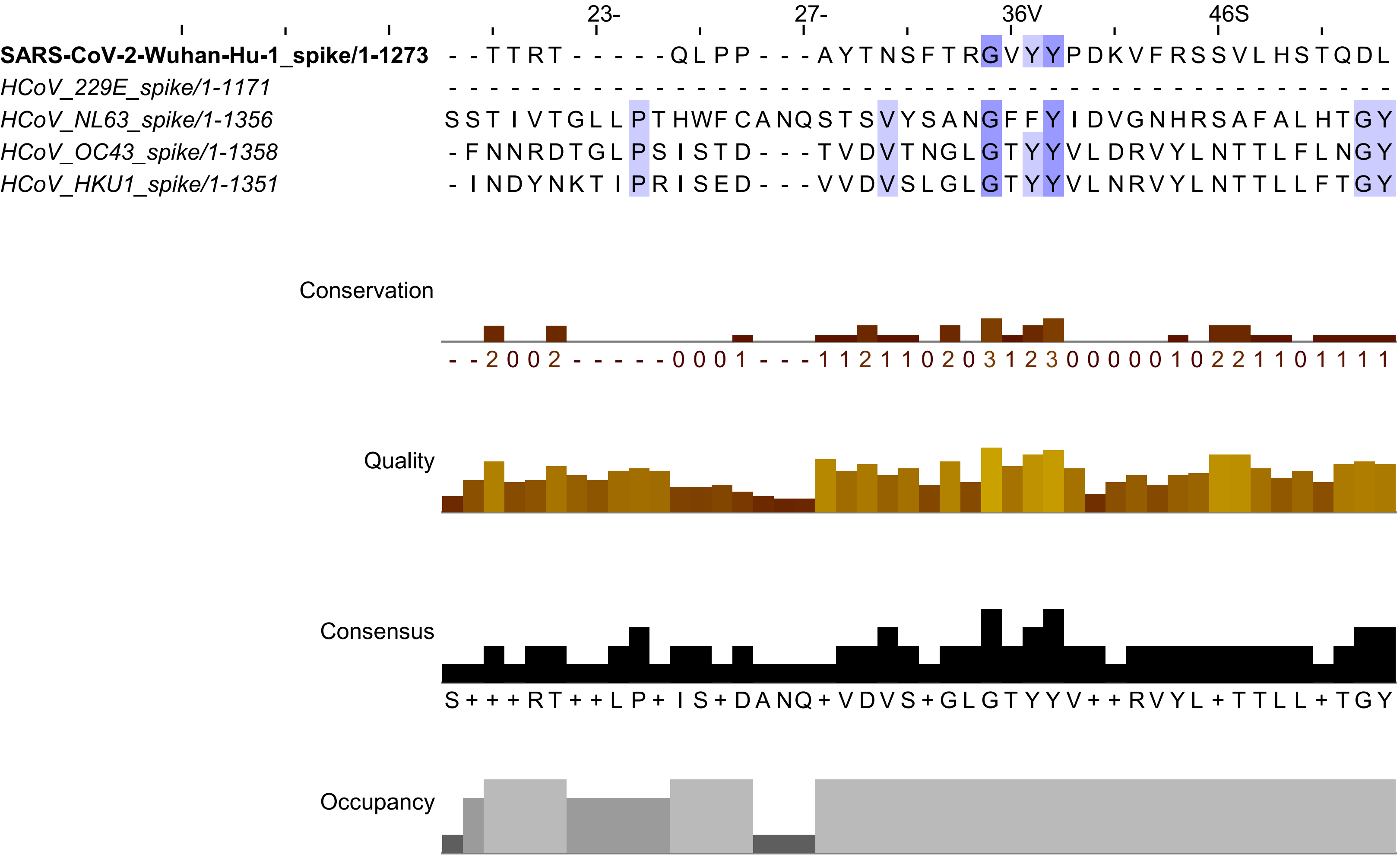
23-
27-
36V
46S
SARS-CoV-2-Wuhan-Hu-1_spike/1-1273
--TTRT-----QLPP---AYTNSFTRGVYYPDKVFRSSVLHSTQDL
HCoV_229E_spike/1-1171
HCoV_NL63_spike/1-1356
SSTIVTGLLPTHWFCANQSTSVYSANGFFYIDVGNHRSAFALHTGY
HCoV_OC43_spike/1-1358
-FNNRDTGLPSISTD---TVDVTNGLGTYYVLDRVYLNTTLFLNGY
HCoV_HKU1_spike/1-1351
-INDYNKTIPRISED---VVDVSLGLGTYYVLNRVYLNTTLLFTGY
Conservation
--2002-----0001---1121102031230000010221101111
Quality
Consensus
S+++RT++LP+IS+DANQ+VDVS+GLGTYYV++RVYL+TTLL+TGY
Occupancy

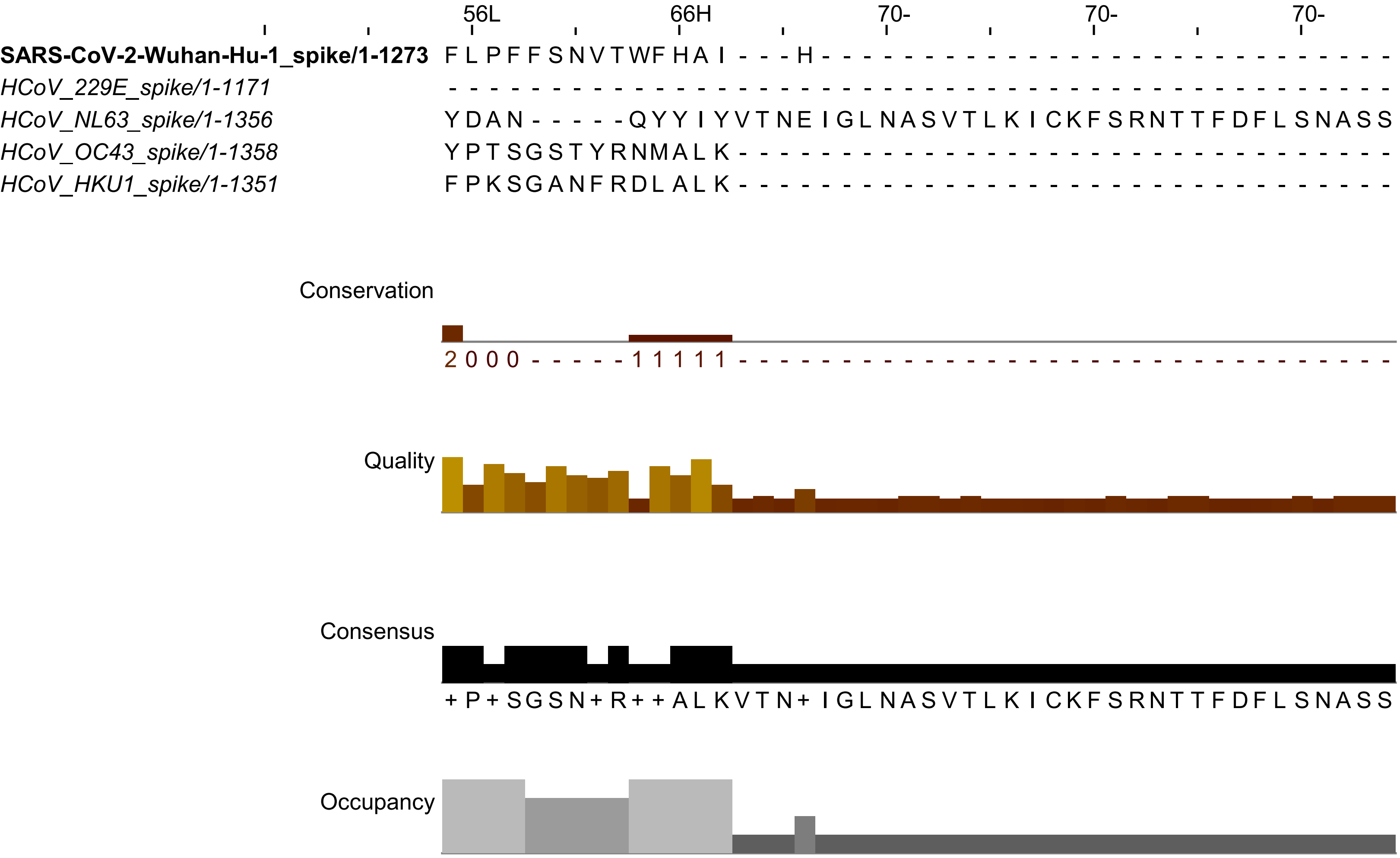
56L
66H
70-
70-
70-
SARS-CoV-2-Wuhan-Hu-1_spike/1-1273 F L P F F S N V T W F H A I - - - H -
HCoV_229E_spike/1-1171 -
HCoV_NL63_spike/1-1356 Y D A N - - - - - Q Y Y I Y V T N E I G L N A S V T L K I C K F S R N T T F D F L S N A S S
HCoV_OC43_spike/1-1358 Y P T S G S T Y R N M A L K -
HCoV_HKU1_spike/1-1351 F P K S G A N F R D L A L K -
Conservation
2 0 0 0 - - - - - 1 1 1 1 1 -
Quality
Consensus
+ P + S G S N + R + + A L K V T N + I G L N A S V T L K I C K F S R N T T F D F L S N A S S
Occupancy

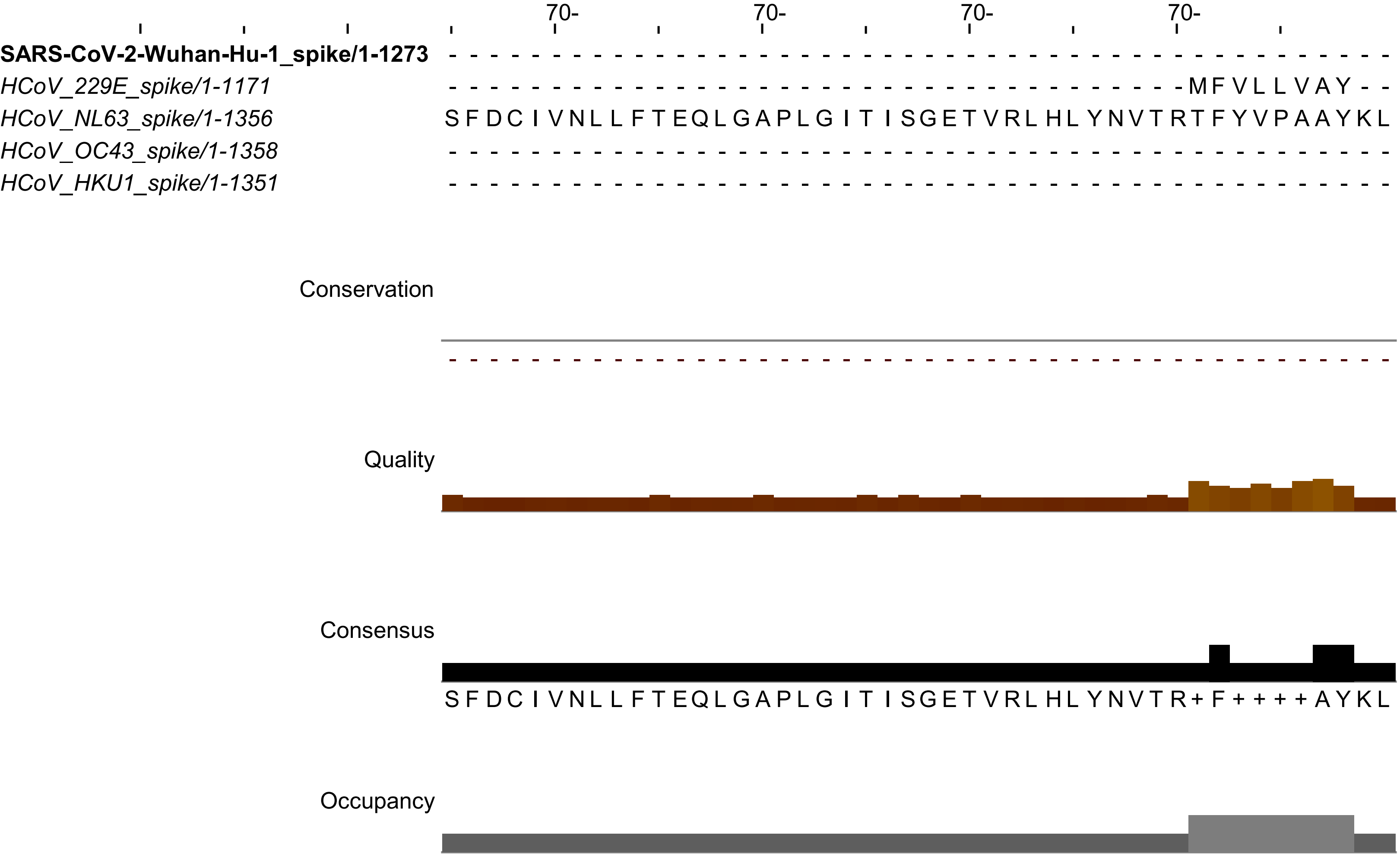

70- 70- 70- 70-
SARS-CoV-2-Wuhan-Hu-1_spike/1-1273 -
HCoV_229E_spike/1-1171 - MFVLLVAY - -
HCoV_NL63_spike/1-1356 SFDCIVNLLFTEQLGAPLGITISGETVRLHLYNVTRTFYVPAAYKL
HCoV_OC43_spike/1-1358 -
HCoV_HKU1_spike/1-1351 -
Conservation
Quality
Consensus
SFDCIVNLLFTEQLGAPLGITISGETVRLHLYNVTR+F++++AYKL
Occupancy

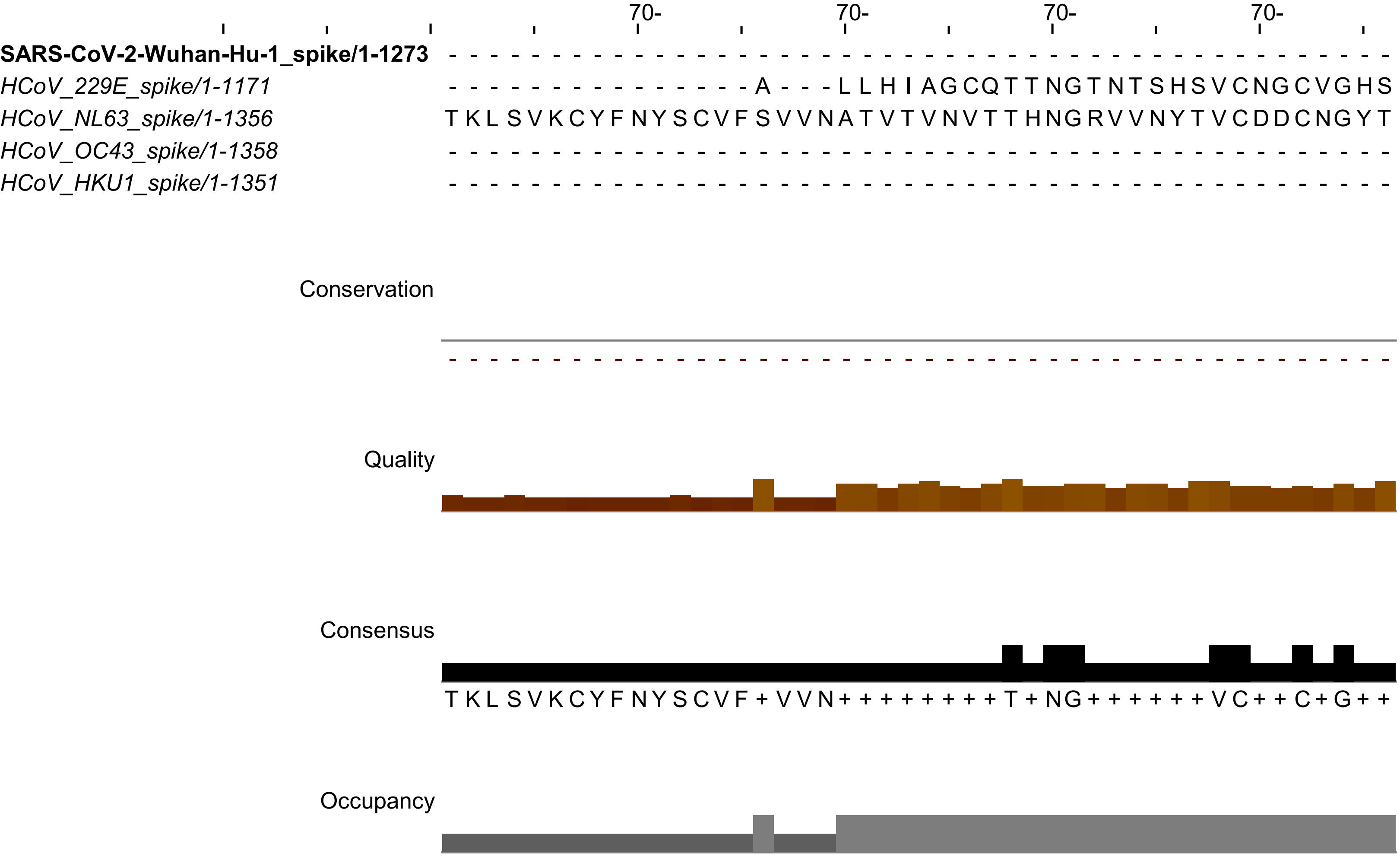
70-
70-
70-
70-
SARS-CoV-2-Wuhan-Hu-1_spike/1-1273
HCoV_229E_spike/1-1171
HCoV_NL63_spike/1-1356
HCoV_OC43_spike/1-1358
HCoV_HKU1_spike/1-1351
- - - - - - - - - - - - - - - A - - - L L H I A G C Q T T N G T N T S H S V C N G C V G H S
T K L S V K C Y F N Y S C V F S V V N A T V T V N V T T H N G R V V N Y T V C D D C N G Y T
Conservation
Quality
Consensus
T K L S V K C Y F N Y S C V F + V V N + + + + + + + + + T + N G + + + + + + V C + + C + G + +
Occupancy

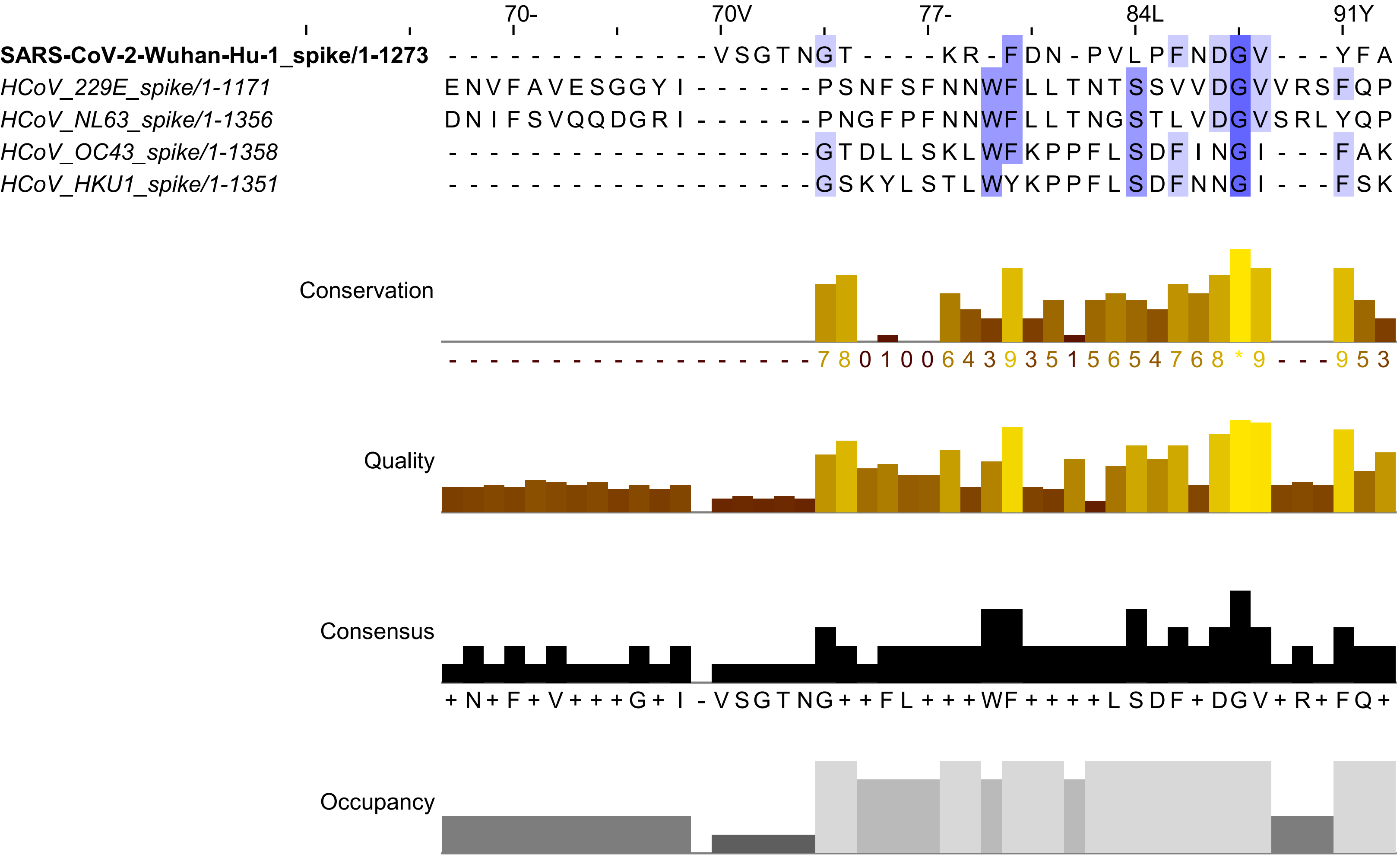

70-
70V
77-
84L
91Y
SARS-CoV-2-Wuhan-Hu-1_spike/1-1273
HCoV_229E_spike/1-1171
HCoV_NL63_spike/1-1356
HCoV_OC43_spike/1-1358
HCoV_HKU1_spike/1-1351
Conservation
Quality
Consensus
Occupancy

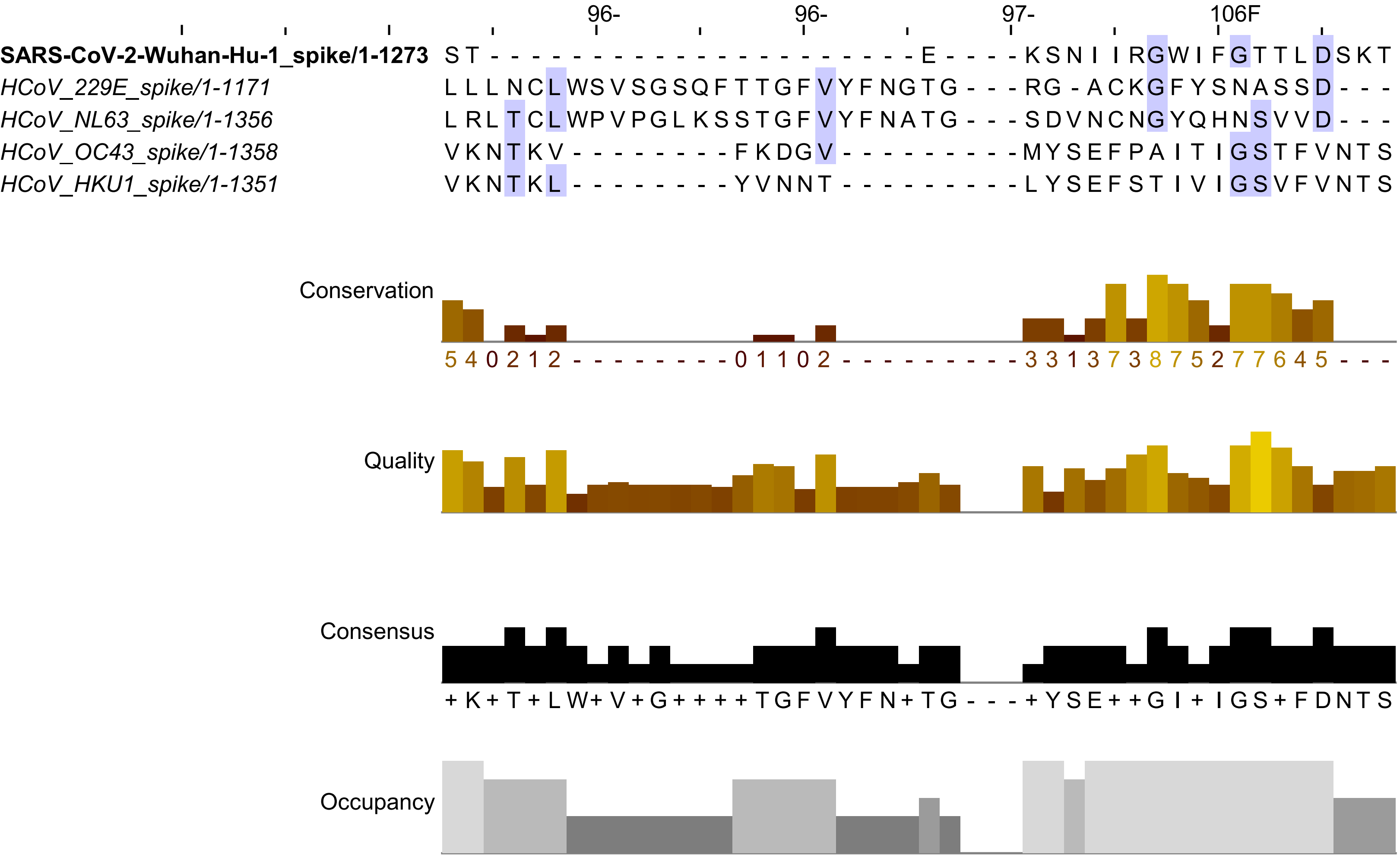

96-
96-
97-
106F
SARS-CoV-2-Wuhan-Hu-1_spike/1-1273
HCoV_229E_spike/1-1171
HCoV_NL63_spike/1-1356
HCoV_OC43_spike/1-1358
HCoV_HKU1_spike/1-1351
Conservation
Quality
Consensus
Occupancy

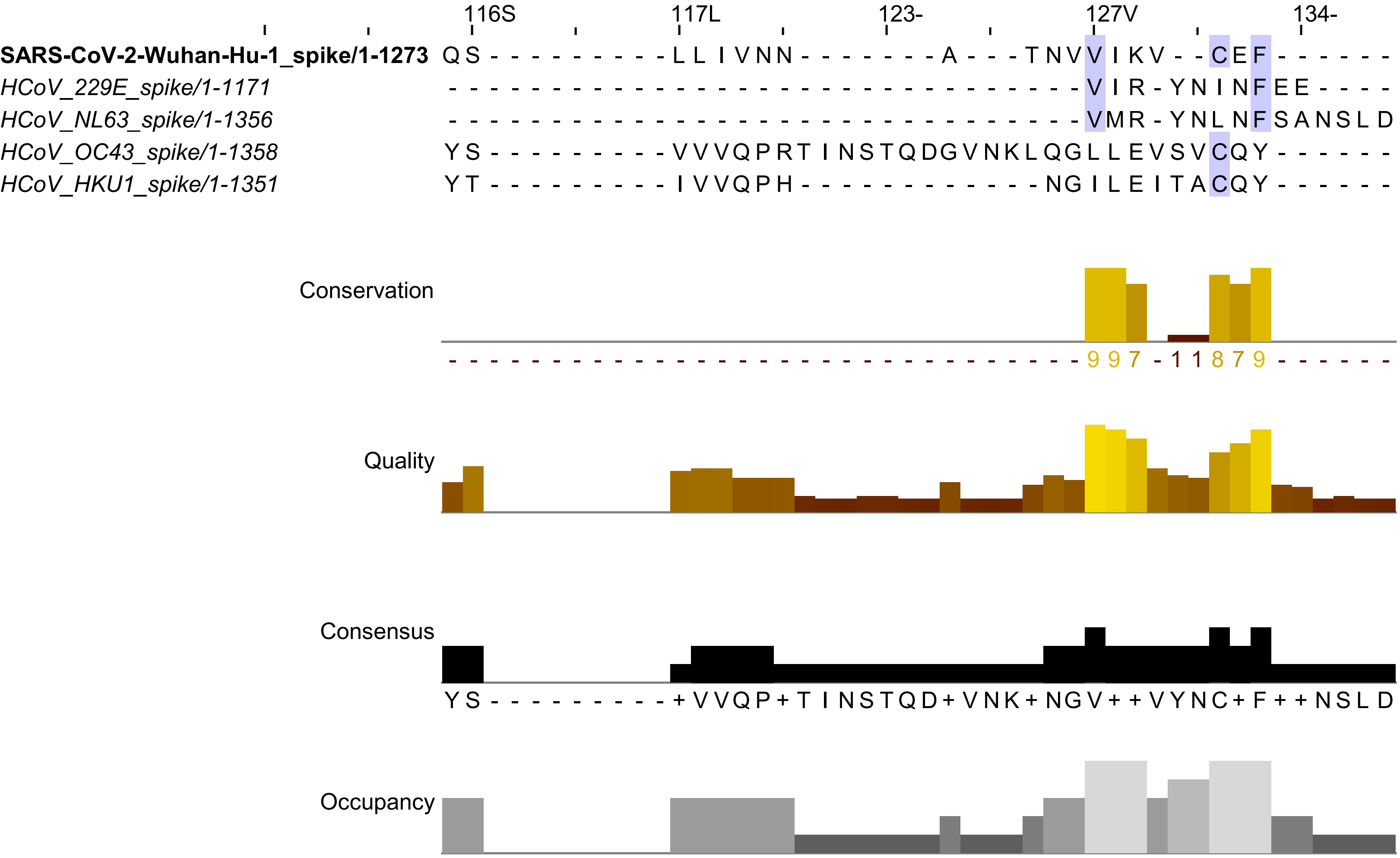
116S
117L
123-
127V
134-
SARS-CoV-2-Wuhan-Hu-1_spike/1-1273 QS---------LLIVNN-------A---TNVVIKV--CEF------
HCoV_229E_spike/1-1171 -----------------------------VIR-YNINFEE----
HCoV_NL63_spike/1-1356 -----------------------------VMR-YNLNFSANSLD
HCoV_OC43_spike/1-1358 YS---------VVVQPRTINSTQDGVNKLQGLLEVSVCQY------
HCoV_HKU1_spike/1-1351 YT---------IVVQPH-------------NGILEITACQY------
Conservation
-----------------------------997-11879------
Quality
Consensus
YS---------+VVQP+TINSTQD+VNK+NGV++VYNC+F++NSLD
Occupancy

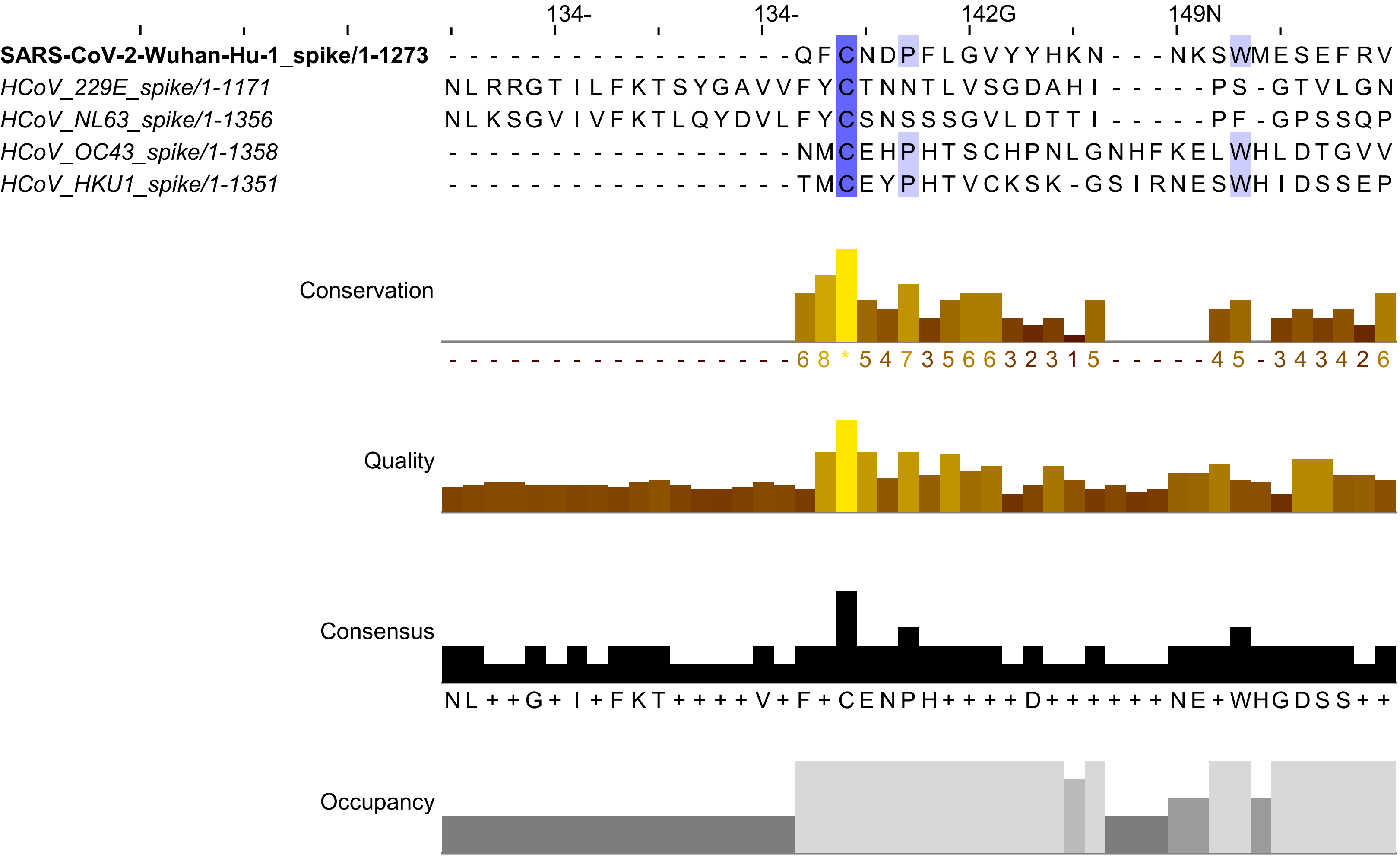

134-
134-
142G
149N
SARS-CoV-2-Wuhan-Hu-1_spike/1-1273
HCoV_229E_spike/1-1171
HCoV_NL63_spike/1-1356
HCoV_OC43_spike/1-1358
HCoV_HKU1_spike/1-1351
- - - - - - - - - - - - - - - - - QFCNDPFLGVYYHKN- - - NKSWMESEFRV
NLRRGTILFKTSYGAVVFYCTNNTLVSGDAHI- - - - - PS-GTVLGN
NLKSGVIVFKTLQYDVLFYCSNSSSGVLDTTI- - - - - PF-GPSSQP
- - - - - - - - - - - - - - - - - NMCEHPHTSCHPNLGNHFKELWHLDTGVV
- - - - - - - - - - - - - - - - - TMCEYPHTVCKSK-GSIRNESWHIDSSEP
Conservation
- - - - - - - - - - - - - - - - - 68*5473566323 15- - - - - 45-343426
Quality
Consensus
NL++G+I+FKT++++V+F+CENPH++++D++++++NE+WHGDSS++
Occupancy

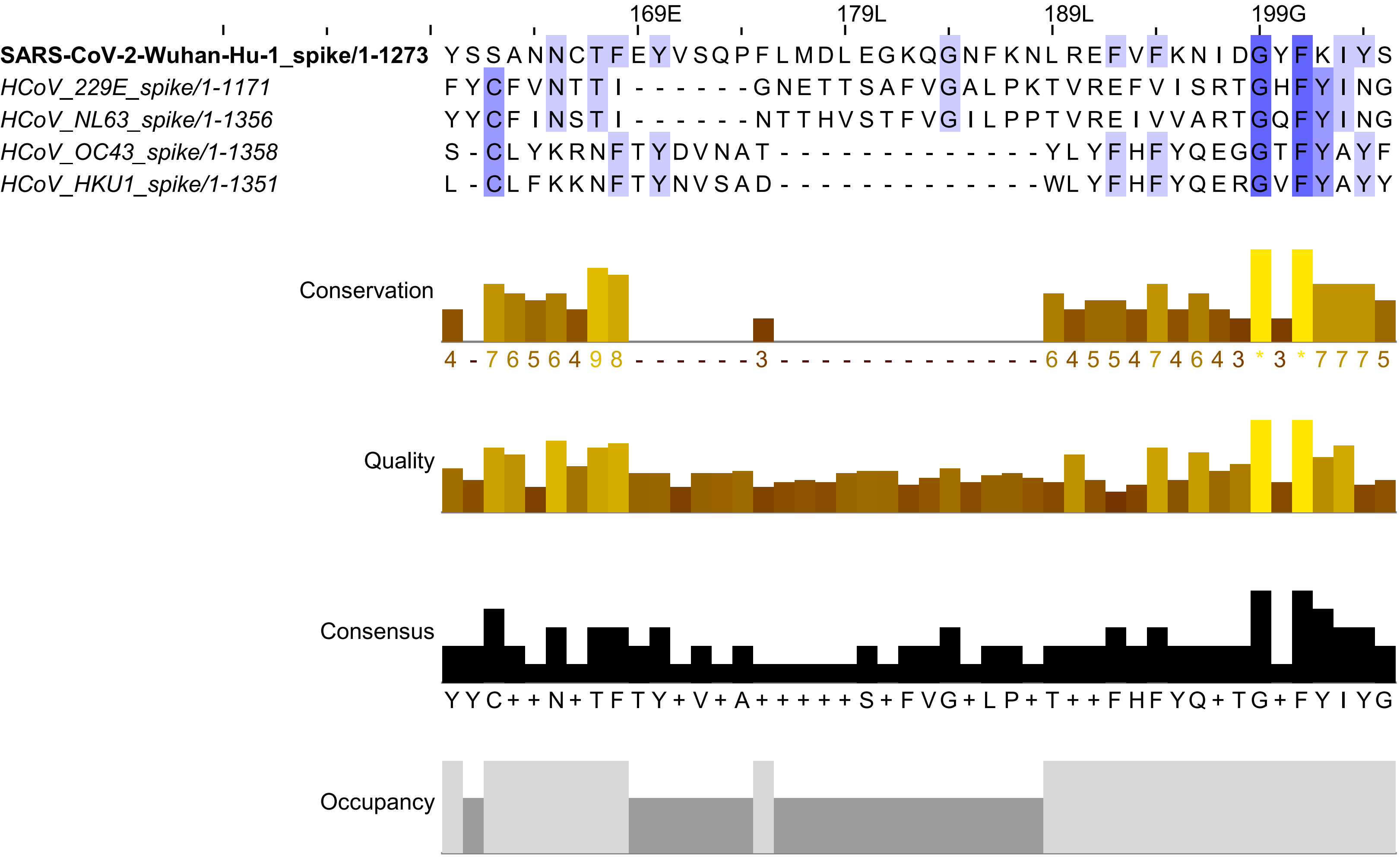
169E
179L
189L
199G
SARS-CoV-2-Wuhan-Hu-1_spike/1-1273
HCoV_229E_spike/1-1171
HCoV_NL63_spike/1-1356
HCoV_OC43_spike/1-1358
HCoV_HKU1_spike/1-1351
Conservation
Quality
Consensus
Occupancy

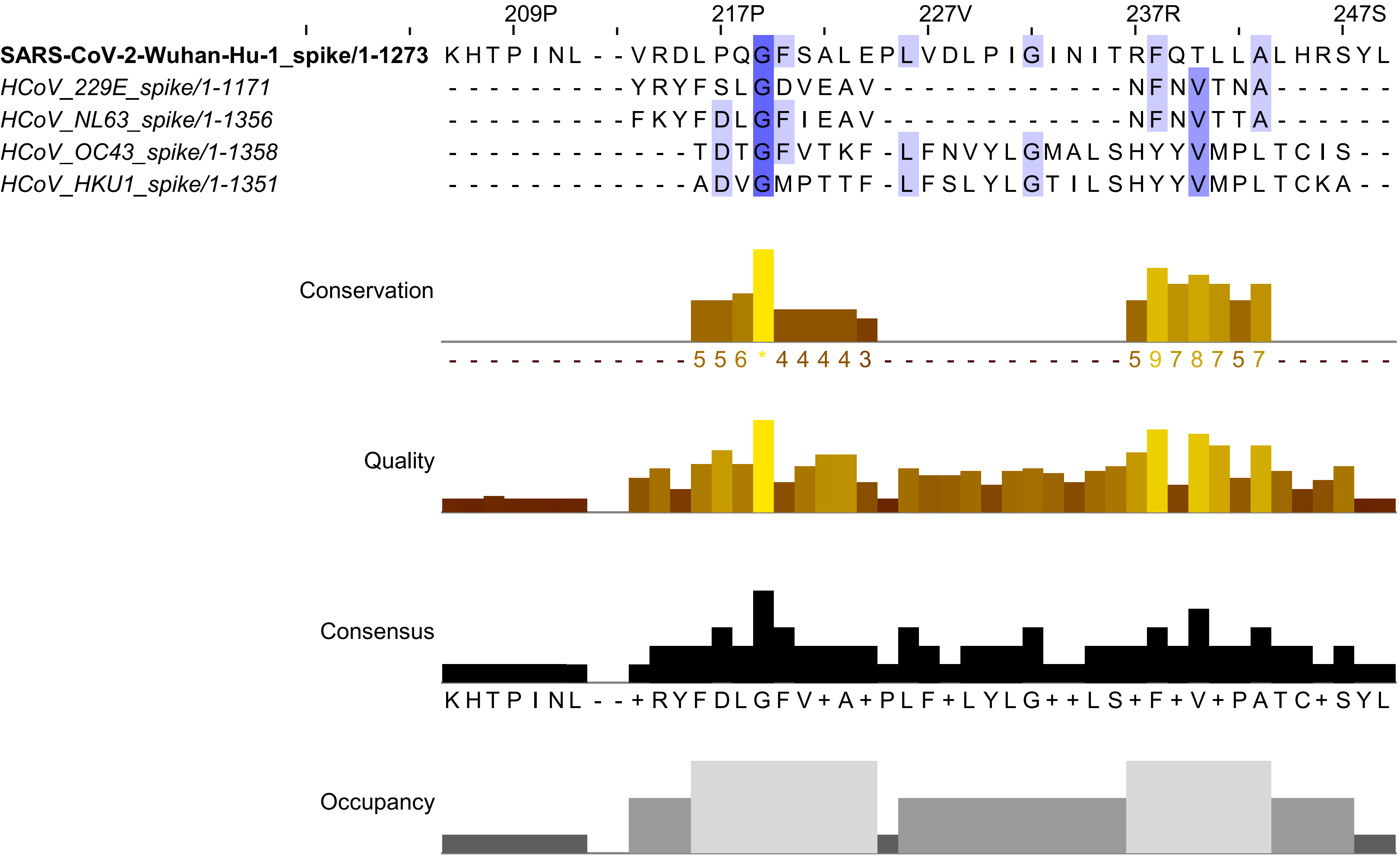

209P 217P 227V 237R 247S
SARS-CoV-2-Wuhan-Hu-1_spike/1-1273 KHTPINL--VRDLPQGFSALEPLVDLPIGINITRFQTLLALHRSYL
HCoV_229E_spike/1-1171 ---------YRYFSLGDVEAV------------NFNVTNA------
HCoV_NL63_spike/1-1356 ---------FKYFDLGFIEAV------------NFNVTTA------
HCoV_OC43_spike/1-1358 ------------TDTGFVTKF-LFNVYLGMALSHYYVMPLTCIS--
HCoV_HKU1_spike/1-1351 ------------ADVGMPTTF-LFSLYLGTILSHYYVMPLTCKA--
Conservation
------------556*44443------------5978757------
Quality
Consensus
KHTPINL--+RYFDLGFV+A+PLF+LYLG++LS+F+V+PATC+SYL
Occupancy

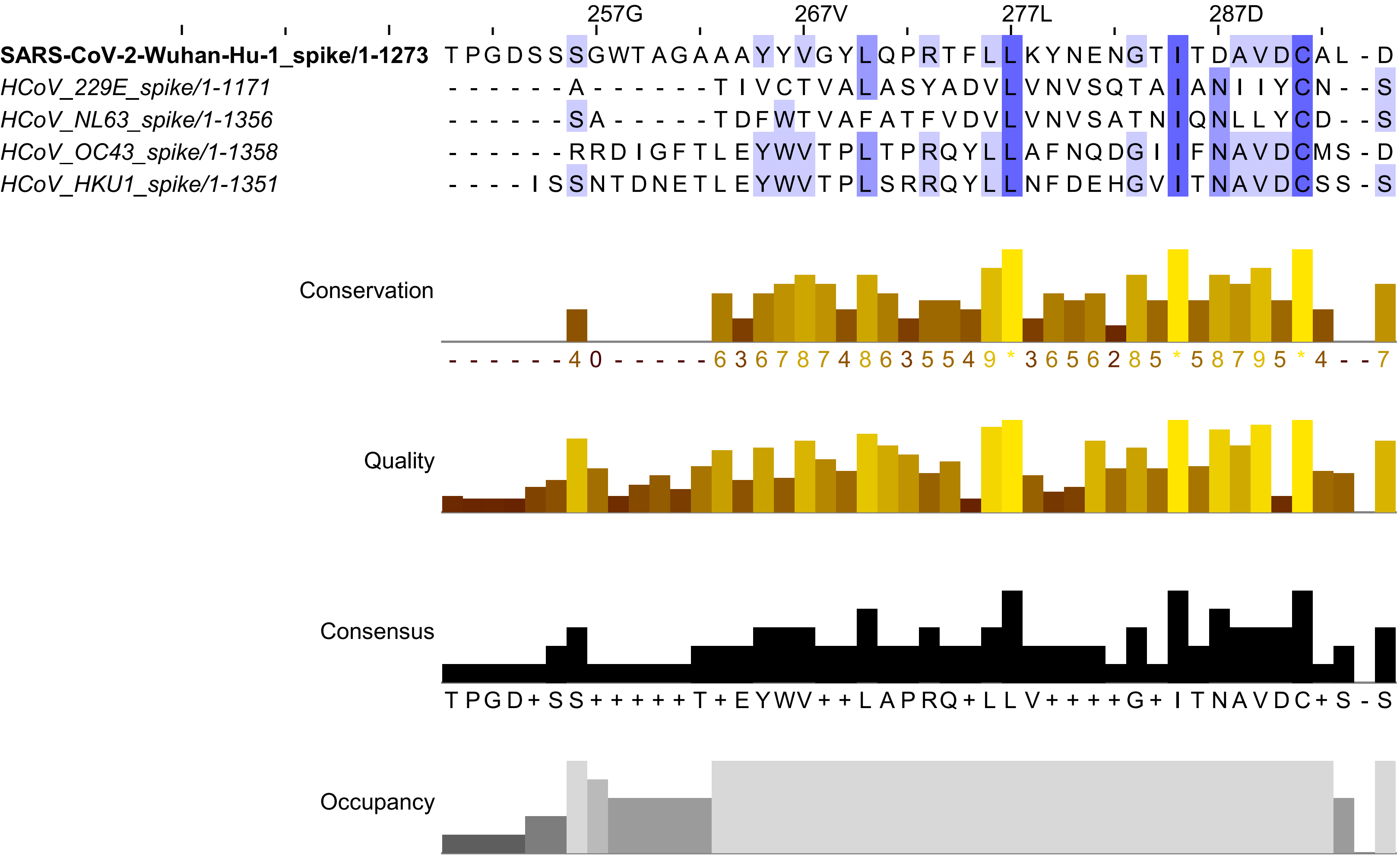

257G
267V
277L
287D
SARS-CoV-2-Wuhan-Hu-1_spike/1-1273 TPGDSSSGWTAGAAAYYVGYLQPRTFLLKYNENGTITDAVDCAL-D
HCoV_229E_spike/1-1171 ------A------TIVCTVALASYADVLVNVSQTAIANIIYCN--S
HCoV_NL63_spike/1-1356 ------SA-----TDFWTVAFATFVDVLVNVSATNIQNLLYCD--S
HCoV_OC43_spike/1-1358 ------RRDIGFTLEYWVTPLTPRQYLLAFNQDGIIFNAVDCMS-D
HCoV_HKU1_spike/1-1351 ----ISSNTDNETLEYWVTPLSRRQYLLNFDEHGVITNAVDCSS-S
Conservation
------40-----636787486355 49*3656285*58795*4--7
Quality
Consensus
TPGD+SS+++++T+EYWV++LAPRQ+LLV++++G+ITNAVDC+S-S
Occupancy

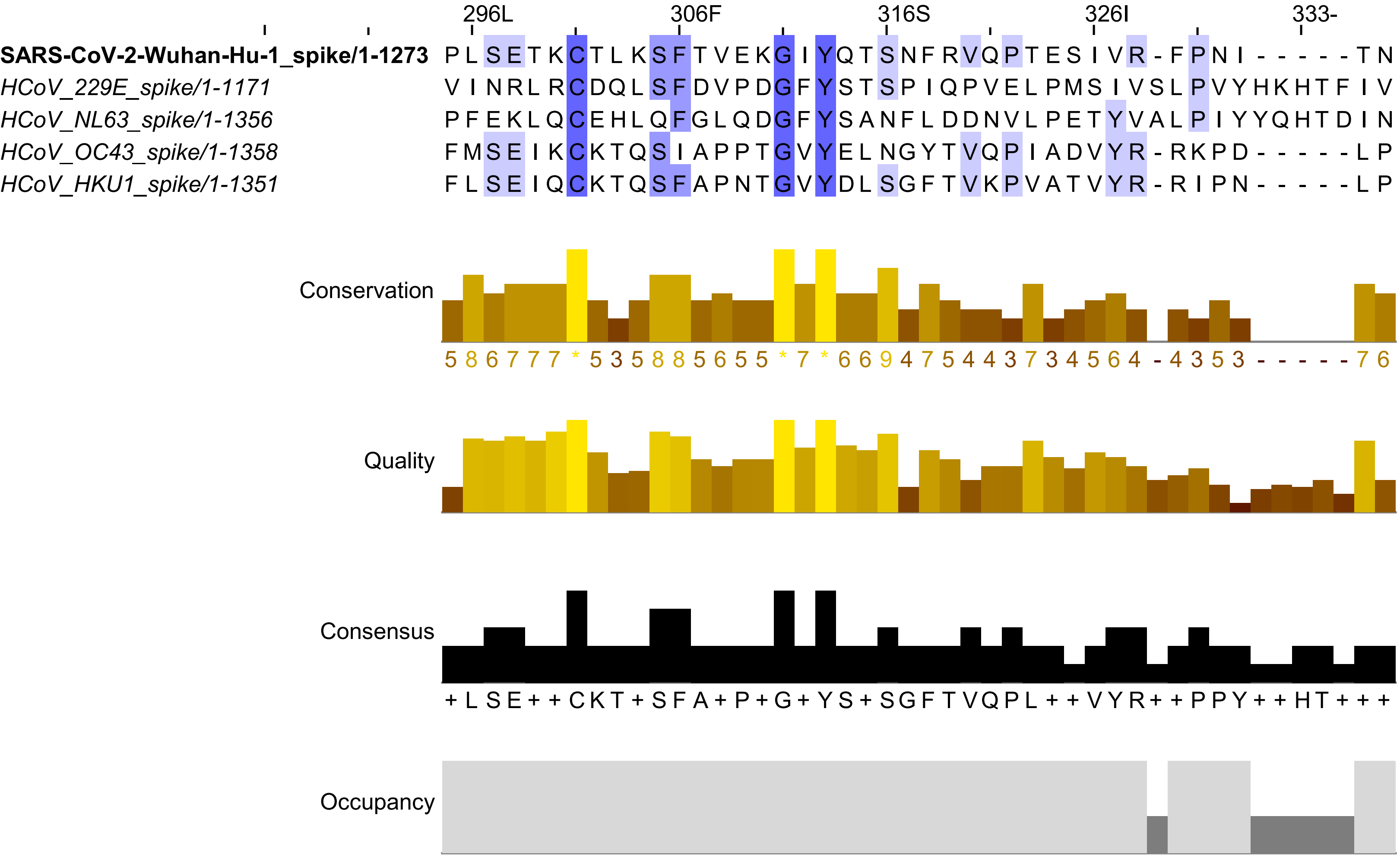
296L
306F
316S
326I
333-
SARS-CoV-2-Wuhan-Hu-1_spike/1-1273 PLSETKCTLKSFTVEKGIYQTSNFRVQPTESIVR-FPNI------TN
HCoV_229E_spike/1-1171 VINRLRCDQLSFDVPDGFYSTSPIQPVELPMSIVSLPVYHKHTFIV
HCoV_NL63_spike/1-1356 PFEKLQCEHLQFGLQDGFYSANFLDDNVLPETYVALPIYYQHTDIN
HCoV_OC43_spike/1-1358 FMSEIKCKTQSIAPPTGVYELNGYTVQPIADVYR-RKPD-----LP
HCoV_HKU1_spike/1-1351 FLSEIQCKTQSFAPNTGVYDLSGFTVKPVATVYR-RIPN-----LP
Conservation
586777*53588565 5*7*669475443734564-4353-----76
Quality
Consensus
+LSE++CKT+SFA+P+G+YS+SGFTVQPL++VYR++PPY++HT+++
Occupancy

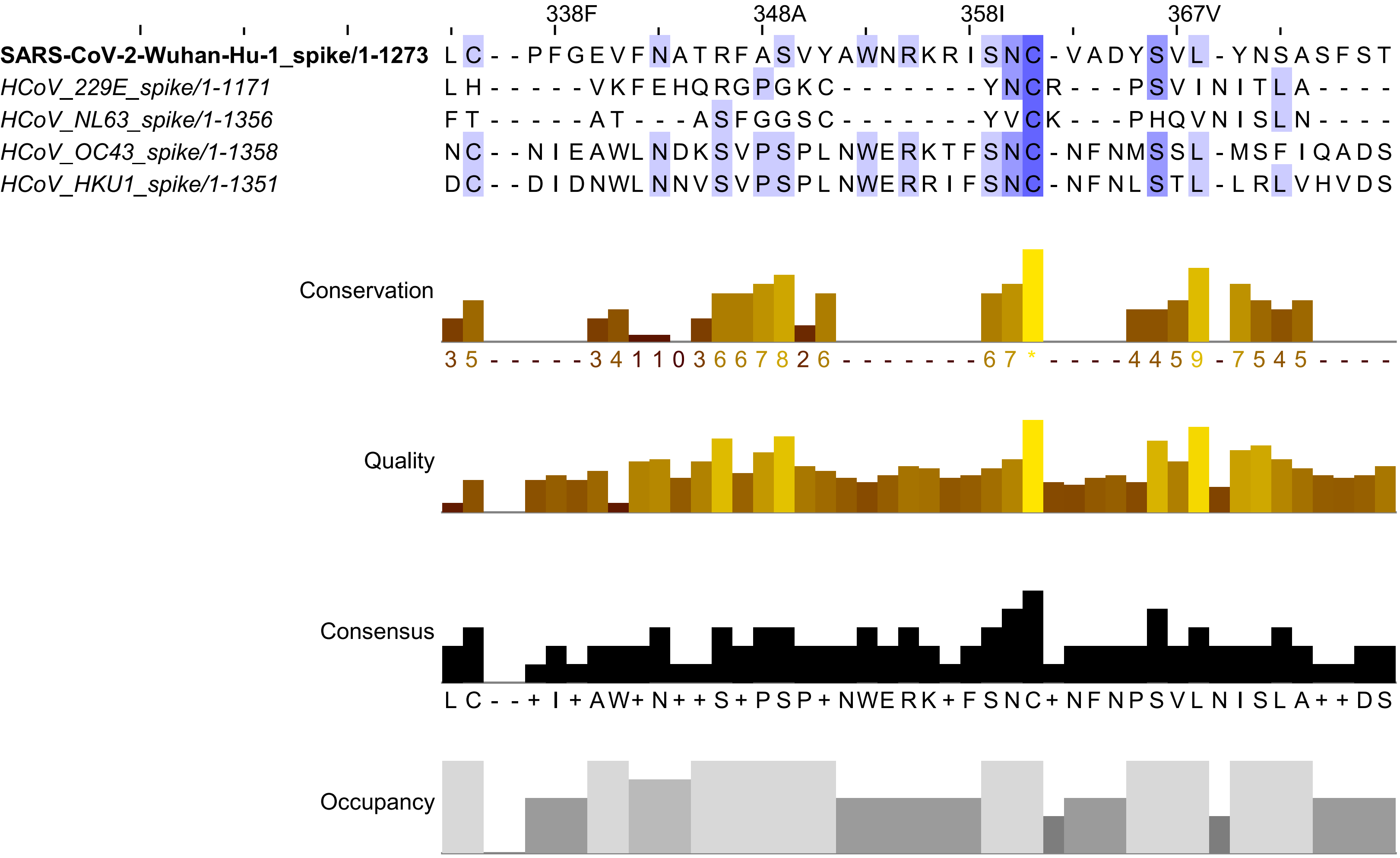

338F
348A
358I
367V
SARS-CoV-2-Wuhan-Hu-1_spike/1-1273 L C - - P F G E V F N A T R F A S V Y A W N R K R I S N C - V A D Y S V L - Y N S A S F S T
HCoV_229E_spike/1-1171 L H - - - - - V K F E H Q R G P G K C - - - - - - - Y N C R - - - P S V I N I T L A - - - -
HCoV_NL63_spike/1-1356 F T - - - - - A T - - - A S F G G S C - - - - - - - Y V C K - - - P H Q V N I S L N - - - -
HCoV_OC43_spike/1-1358 N C - - N I E A W L N D K S V P S P L N W E R K T F S N C - N F N M S S L - M S F I Q A D S
HCoV_HKU1_spike/1-1351 D C - - D I D N W L N N V S V P S P L N W E R R I F S N C - N F N L S T L - L R L V H V D S
Conservation
3 5 - - - - - 3 4 1 1 0 3 6 6 7 8 2 6 - - - - - - - 6 7 * - - - - 4 4 5 9 - 7 5 4 5 - - - -
Quality
Consensus
L C - - + I + A W + N + + S + P S P + N W E R K + F S N C + N F N P S V L N I S L A + + D S
Occupancy

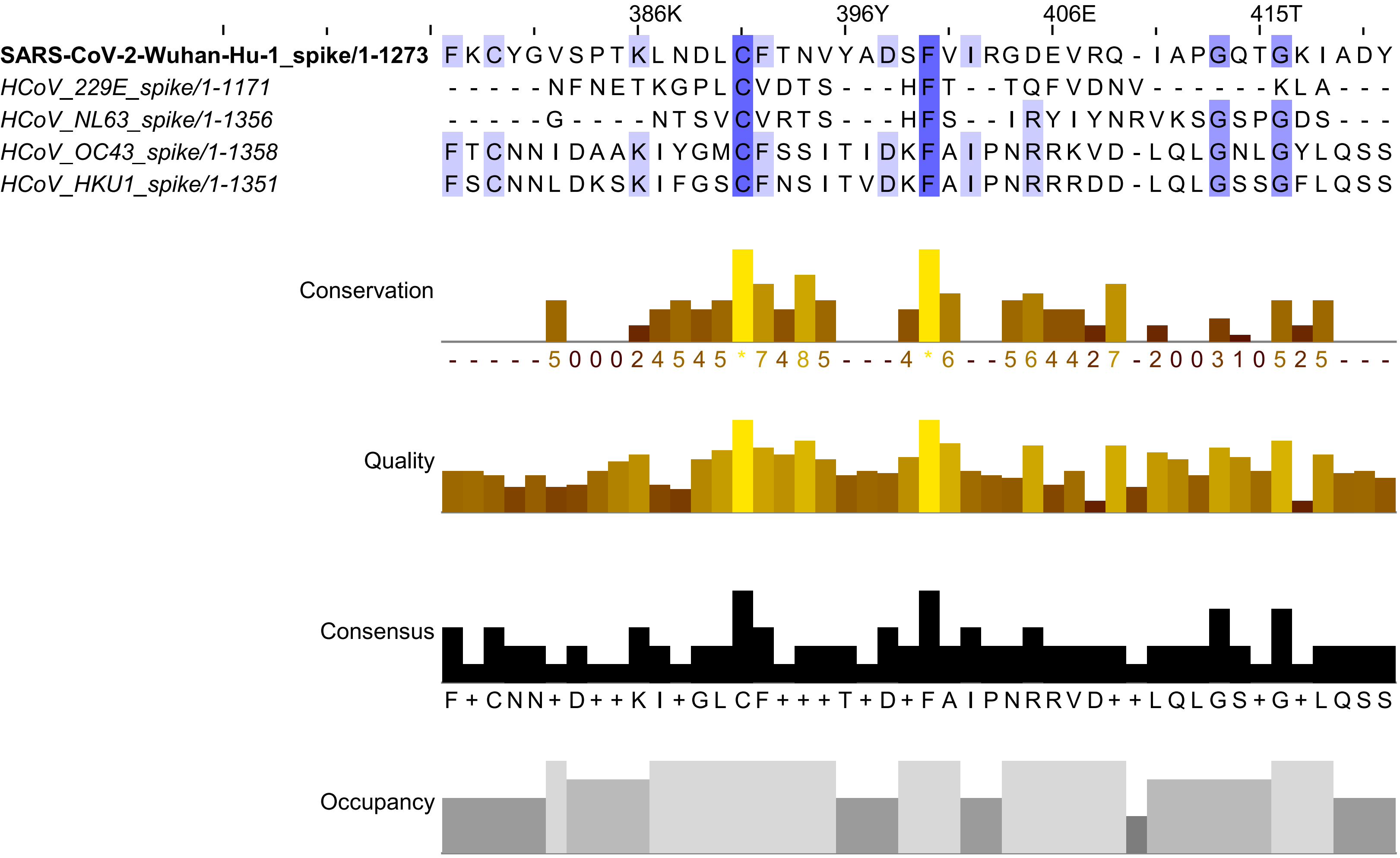
386K
396Y
406E
415T
SARS-CoV-2-Wuhan-Hu-1_spike/1-1273 FKCYGVSPTKLNDLCFTNVYADSFVIRGDEVRQ-IAPGQTGKIADY
HCoV_229E_spike/1-1171 -----NFNETKGPLCVDTS---HFT--TQFVDNV------KLA---
HCoV_NL63_spike/1-1356 -----G----NTSVCVRTS---HFS--IRYIYNRVKSGSPGDS---
HCoV_OC43_spike/1-1358 FTCNNIDAAKIYGMCFSSITIDKFAIPNRRKVD-LQLGNLGYLQSS
HCoV_HKU1_spike/1-1351 FSCNNLDKSKIFGSCFNSITVDKFAIPNRRRDD-LQLGSSGFLQSS
Conservation
-----50002454 5*7485---4*6--564427-2003105 25---
Quality
Consensus
F+CNN+D++KI+GLCF+++T+D+FAIPNRRVD++LQLGS+G+LQSS
Occupancy

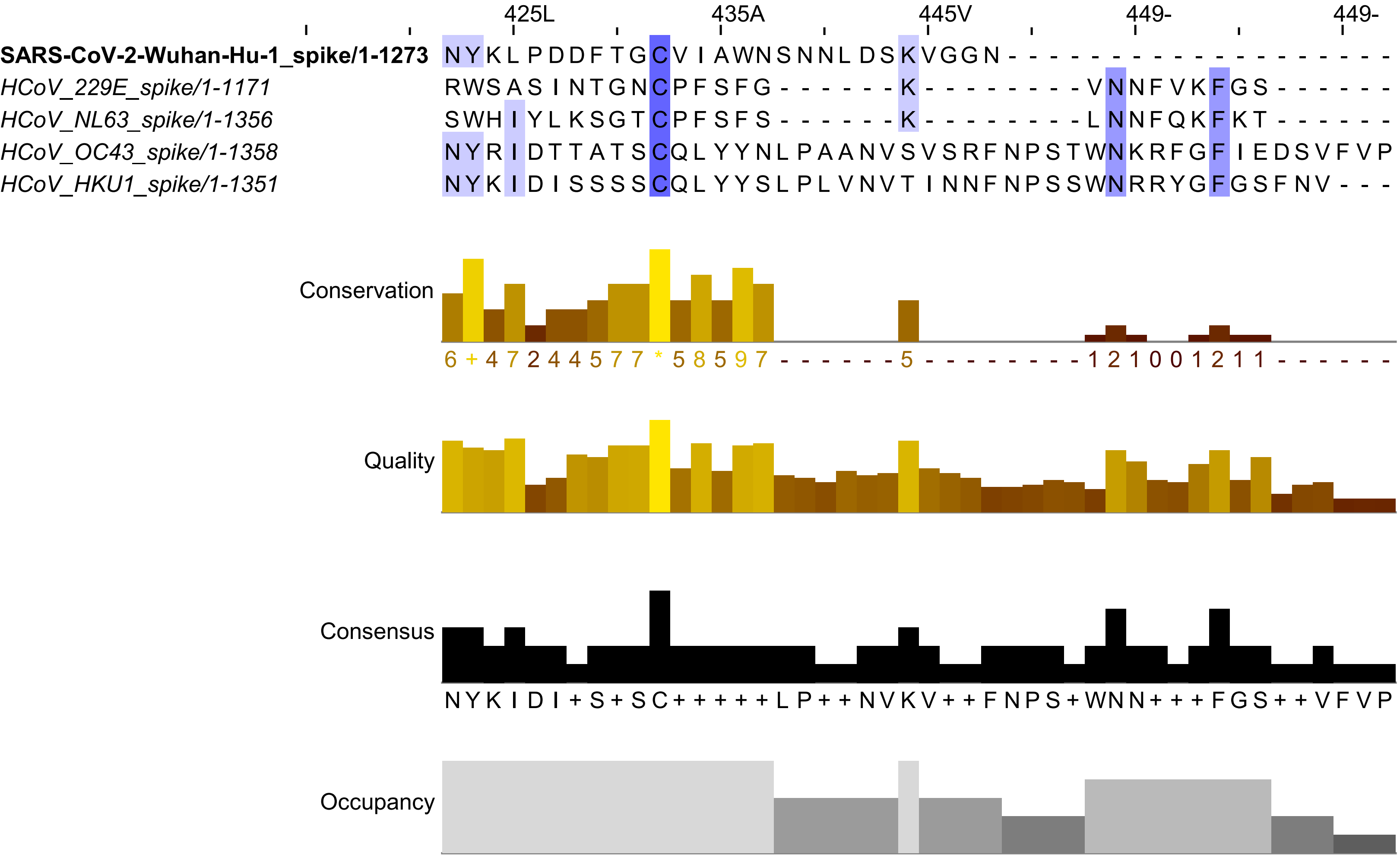

425L
435A
445V
449-
449-
SARS-CoV-2-Wuhan-Hu-1_spike/1-1273
HCoV_229E_spike/1-1171
HCoV_NL63_spike/1-1356
HCoV_OC43_spike/1-1358
HCoV_HKU1_spike/1-1351
NYKLPDDFTGCVIAWNSNNLDSKVGGN------------------
RWSASINTGNCPFSFG------K---------VNNFVKFGS------
SWHIYLKSGTCPFSFS------K---------LNNFQKFKT------
NYRIDTTATSCQLYYNLPAANVSVSRFNPSTWNKRFGFIEDSVFVP
NYKIDISSSSCQLYYSLPLVNVTINNFNPSSWNRRYGFGSFNV---
Conservation
6+47244577*58597------5---------121001211------
Quality
Consensus
NYKIDI+S+SC+++++LP++NVKV++FNPS+WNN+++FGS++VFVP
Occupancy

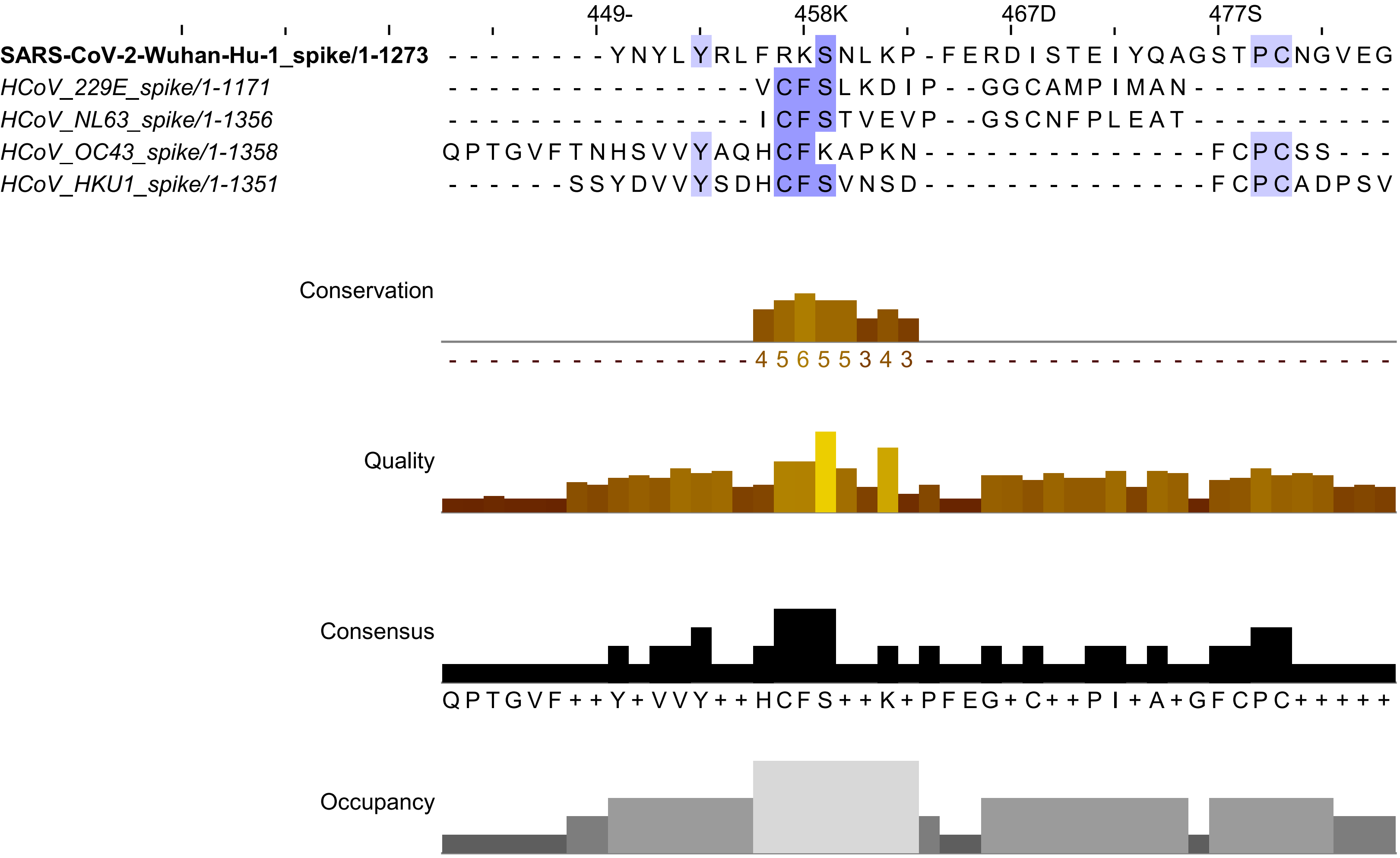
449-
458K
467D
477S
SARS-CoV-2-Wuhan-Hu-1_spike/1-1273 --------YNYLYRLFRKSNLKP-FERDISTEIYQAGSTPCNGVEG
HCoV_229E_spike/1-1171 ----------------VCFSLKDIP--GGCAMPIMAN----------
HCoV_NL63_spike/1-1356 ---------------ICFSTVEVP--GSCNFPLEAT----------
HCoV_OC43_spike/1-1358 QPTGVFTNHSVVYAQHCFKAPKN--------------FCPCSS---
HCoV_HKU1_spike/1-1351 ------SSYDVVYSDHCFSVNSD---------------FCPCADPSV
Conservation
4 5 6 5 5 3 4 3
Quality
Consensus
QPTGVF++Y+VVY++HCFS++K+PFEG+C++PI+A+GFCPC+++++
Occupancy

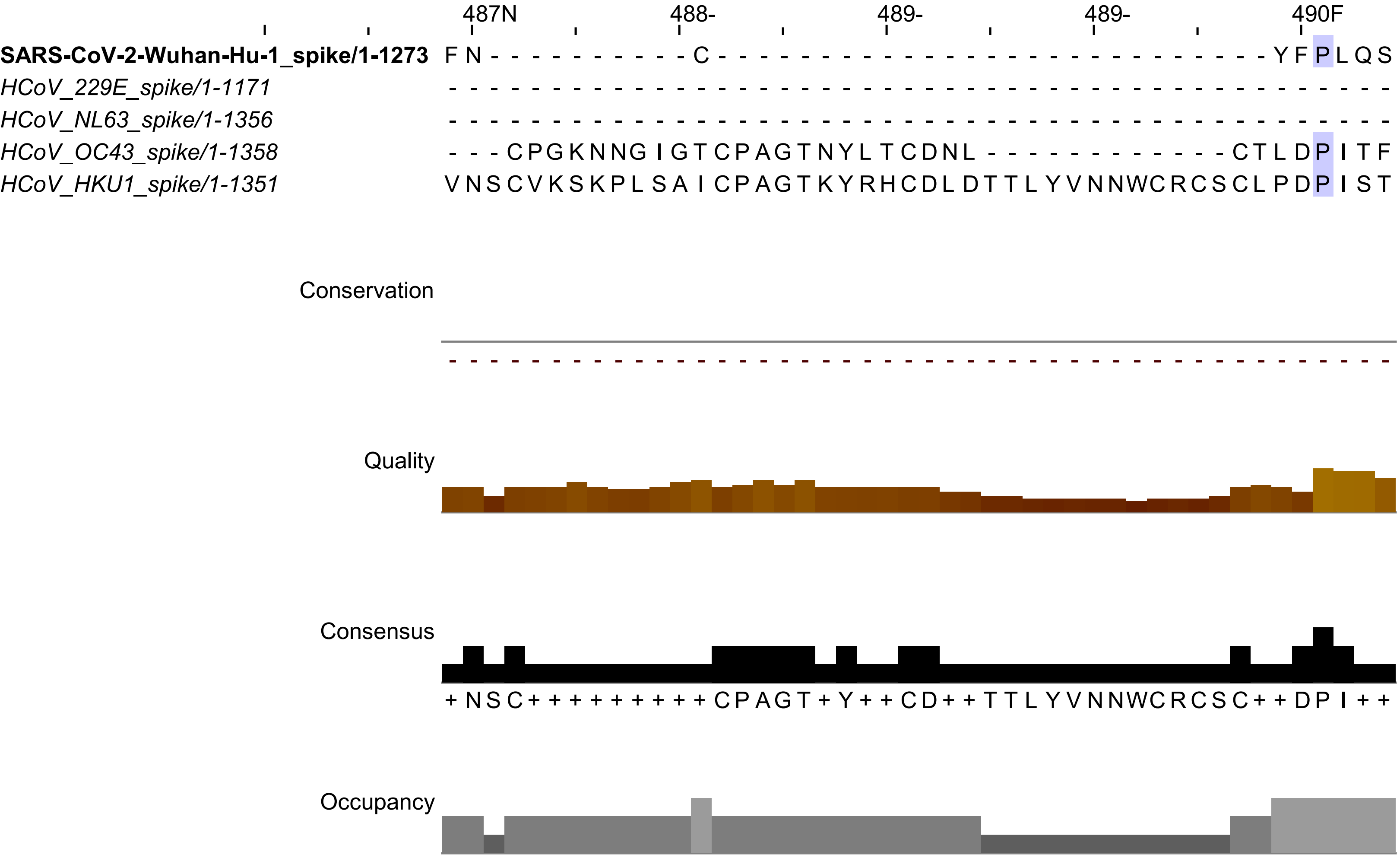
487N
488-
489-
489-
490F
SARS-CoV-2-Wuhan-Hu-1_spike/1-1273 F N - - - - - - - - - - C - Y F P L Q S
HCoV_229E_spike/1-1171
HCoV_NL63_spike/1-1356
HCoV_OC43_spike/1-1358 - - - C P G K N N G I G T C P A G T N Y L T C D N L - - - - - - - - - - - - C T L D P I T F
HCoV_HKU1_spike/1-1351 V N S C V K S K P L S A I C P A G T K Y R H C D L D T T L Y V N N W C R C S C L P D P I S T
Conservation
Quality
Consensus
+ N S C + + + + + + + + + + C P A G T + Y + + C D + + T T L Y V N N W C R C S C + + D P I + +
Occupancy

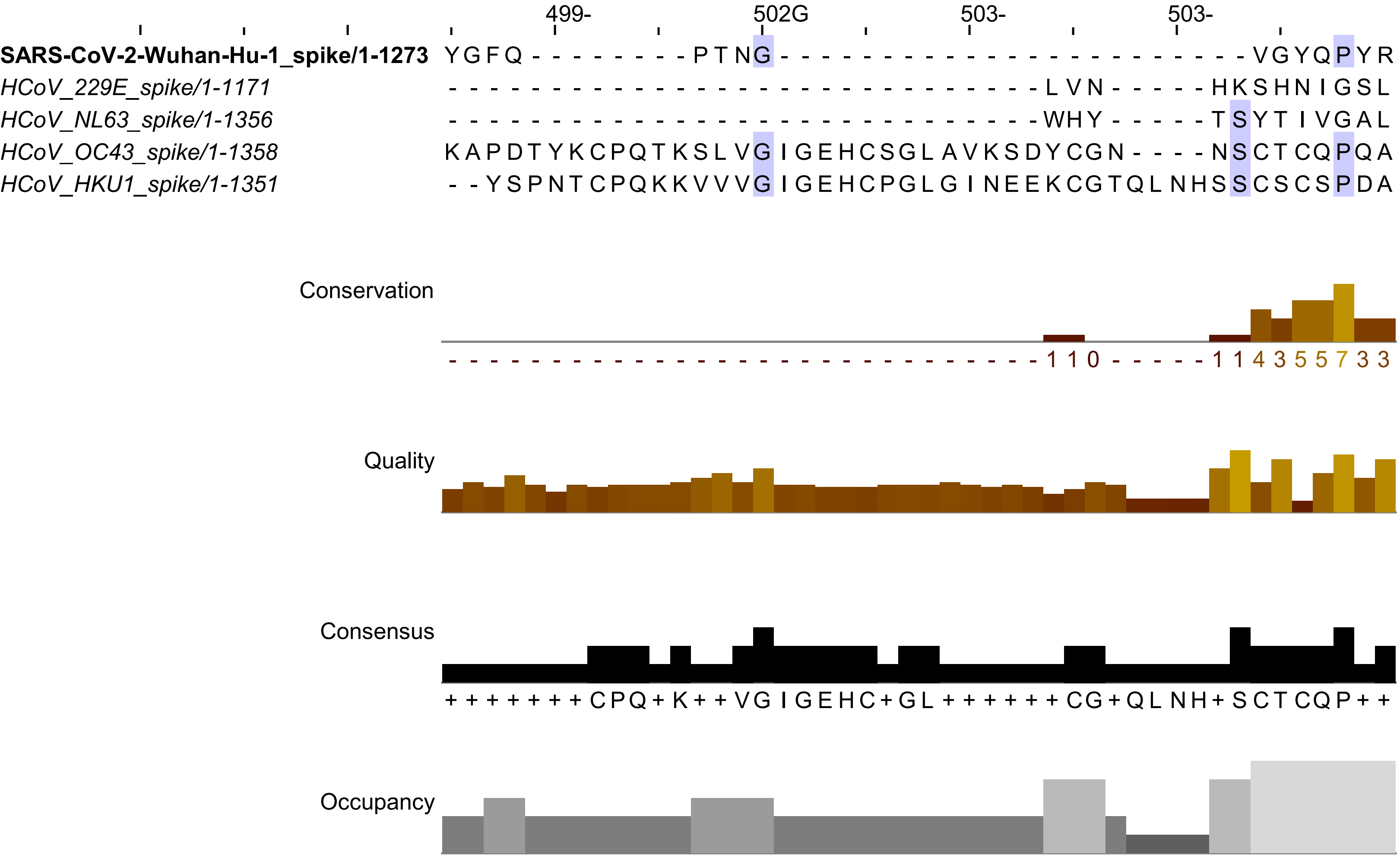

499-
502G
503-
503-
SARS-CoV-2-Wuhan-Hu-1_spike/1-1273 YGFQ--------PTNG---------------------VGYQPYR
HCoV_229E_spike/1-1171 -----------------------------LVN-----HKSHNIGSL
HCoV_NL63_spike/1-1356 -----------------------------WHY-----TSYTIVGAL
HCoV_OC43_spike/1-1358 KAPDTYKCPQTKSLVGIGEHCSGLAVKSDYCGN----NSCTCQPQA
HCoV_HKU1_spike/1-1351 --YSPNTCPQKKVVVGIGEHCPGLGINEEKCGTQLNHSSCSCSPDA
Conservation
-----------------------------110-----11435573 3
Quality
Consensus
+++++++CPQ+K++VGIGEHC+GL+++++++CG+QLNH+SCTCQP++
Occupancy

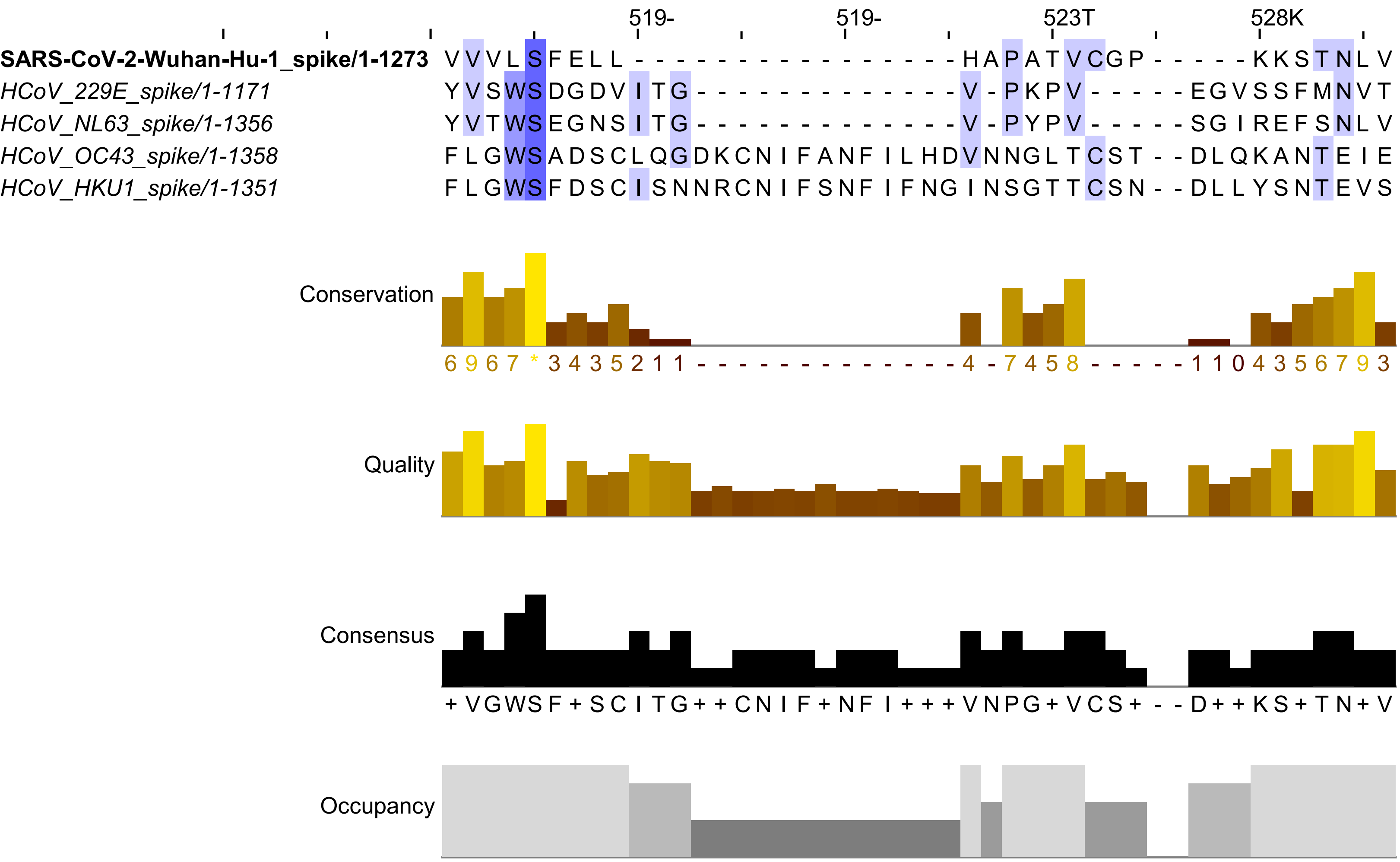
519-
519-
523T
528K
SARS-CoV-2-Wuhan-Hu-1_spike/1-1273
HCoV_229E_spike/1-1171
HCoV_NL63_spike/1-1356
HCoV_OC43_spike/1-1358
HCoV_HKU1_spike/1-1351
Conservation
Quality
Consensus
Occupancy

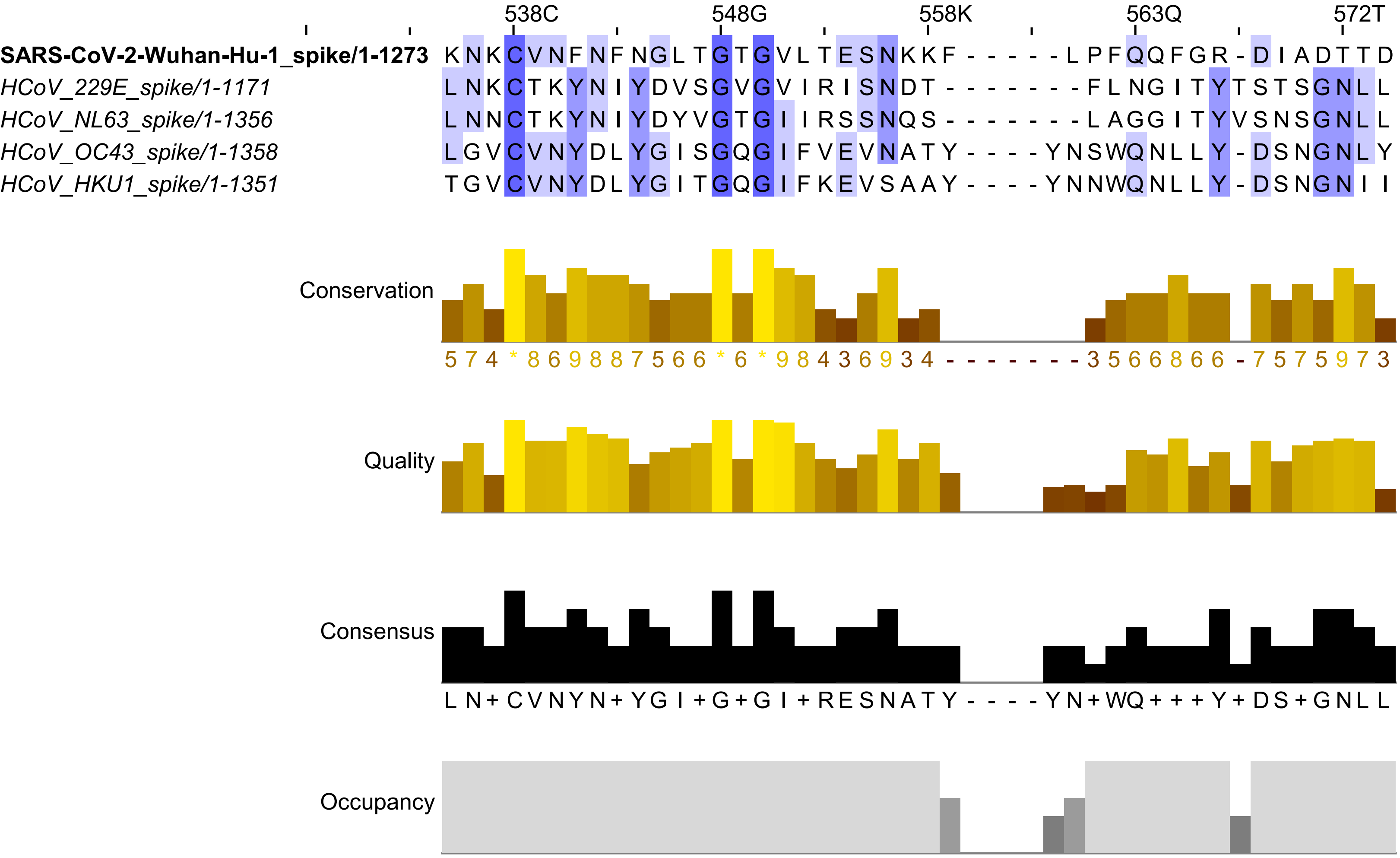
538C
548G
558K
563Q
572T
SARS-CoV-2-Wuhan-Hu-1_spike/1-1273 KNKCVNFNFNGLTGTGVLTESNKKF-----LPFQQFGR-DIADTTD
HCoV_229E_spike/1-1171 LNKCTKYNIYDVSGVGVIRISNDT--------FLNGITYTSTSGNLL
HCoV_NL63_spike/1-1356 LNNCTKYNIYDYVGTGIIRSSNQS-------LAGGITYVSNSGNLL
HCoV_OC43_spike/1-1358 LGVCVNYDLYGISGQGIFVEVNATY----YNSWQNLLY-DSNGNLY
HCoV_HKU1_spike/1-1351 TGVCVNYDLYGITGQGIFKEVSAAY----YNNWQNLLY-DSNGNII
Conservation
574*869887566*6*9843693 4-------3566866-757597 3
Quality
Consensus
LN+CVNYN+YGI+G+GI+RESNATY----YN+WQ+++Y+DS+GNLL
Occupancy

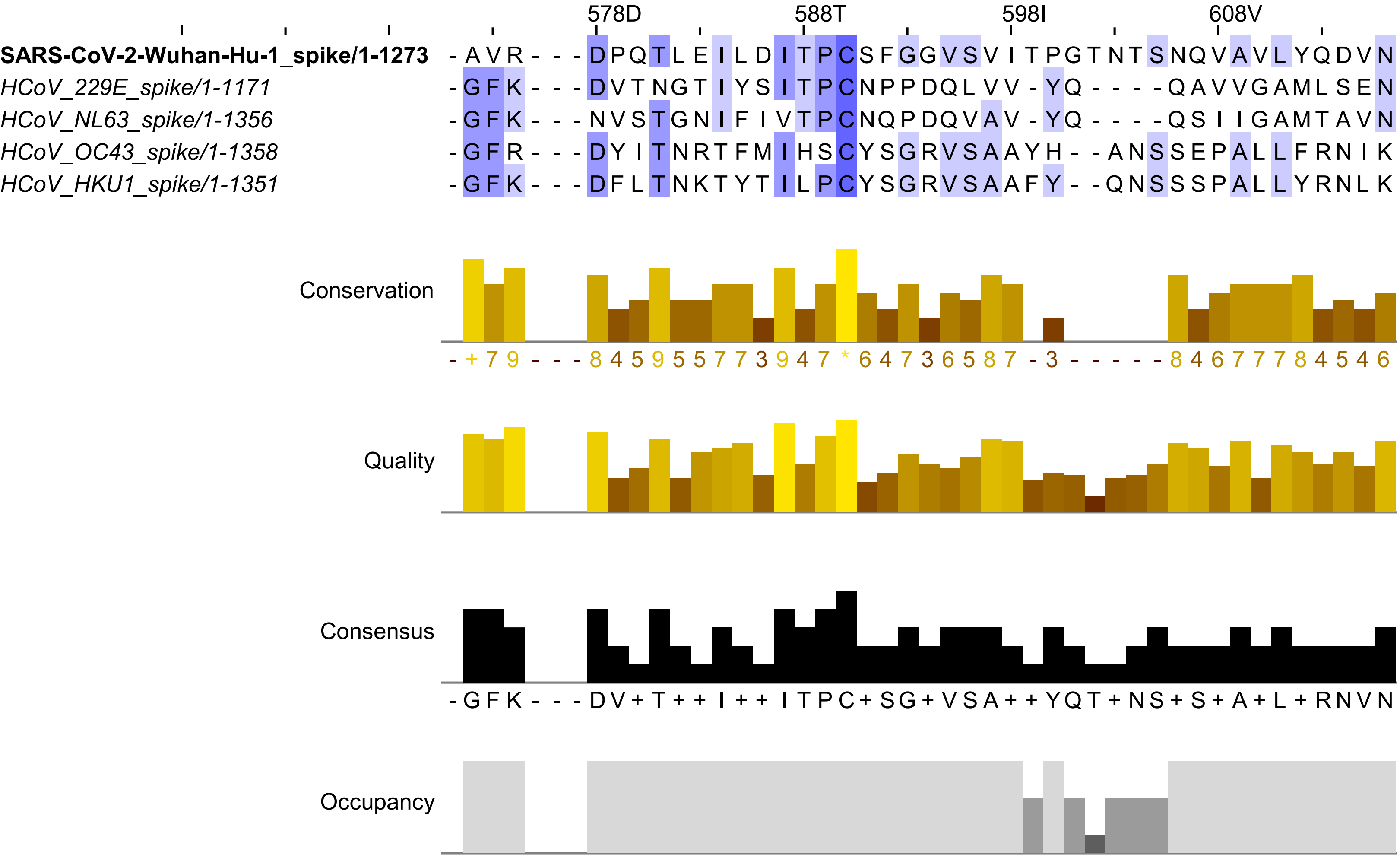
578D
588T
598I
608V
SARS-CoV-2-Wuhan-Hu-1_spike/1-1273 -AVR---DPQTLEILDITPCSFGGVSVITPGTNTSNQVAVLYQDVN
HCoV_229E_spike/1-1171 -GFK---DVTNGTIYSITPCNPPDQLVV-YQ----QAVVGAMLSEN
HCoV_NL63_spike/1-1356 -GFK---NVSTGNIFIVTPCNQPDQVAV-YQ----QSIIGAMTAVN
HCoV_OC43_spike/1-1358 -GFR---DYITNRTFMIHSCYSGRVSAAYH--ANSSEPALLFRNIK
HCoV_HKU1_spike/1-1351 -GFK---DFLTNKTYTILPCYSGRVSAAFY--QNSSSPALLYRNLK
Conservation
-+79---84595577394 7*64736587-3-----84677784546
Quality
Consensus
-GFK---DV+T++I++ITPC+SG+VSA++YQT+NS+S+A+L+RNVN
Occupancy

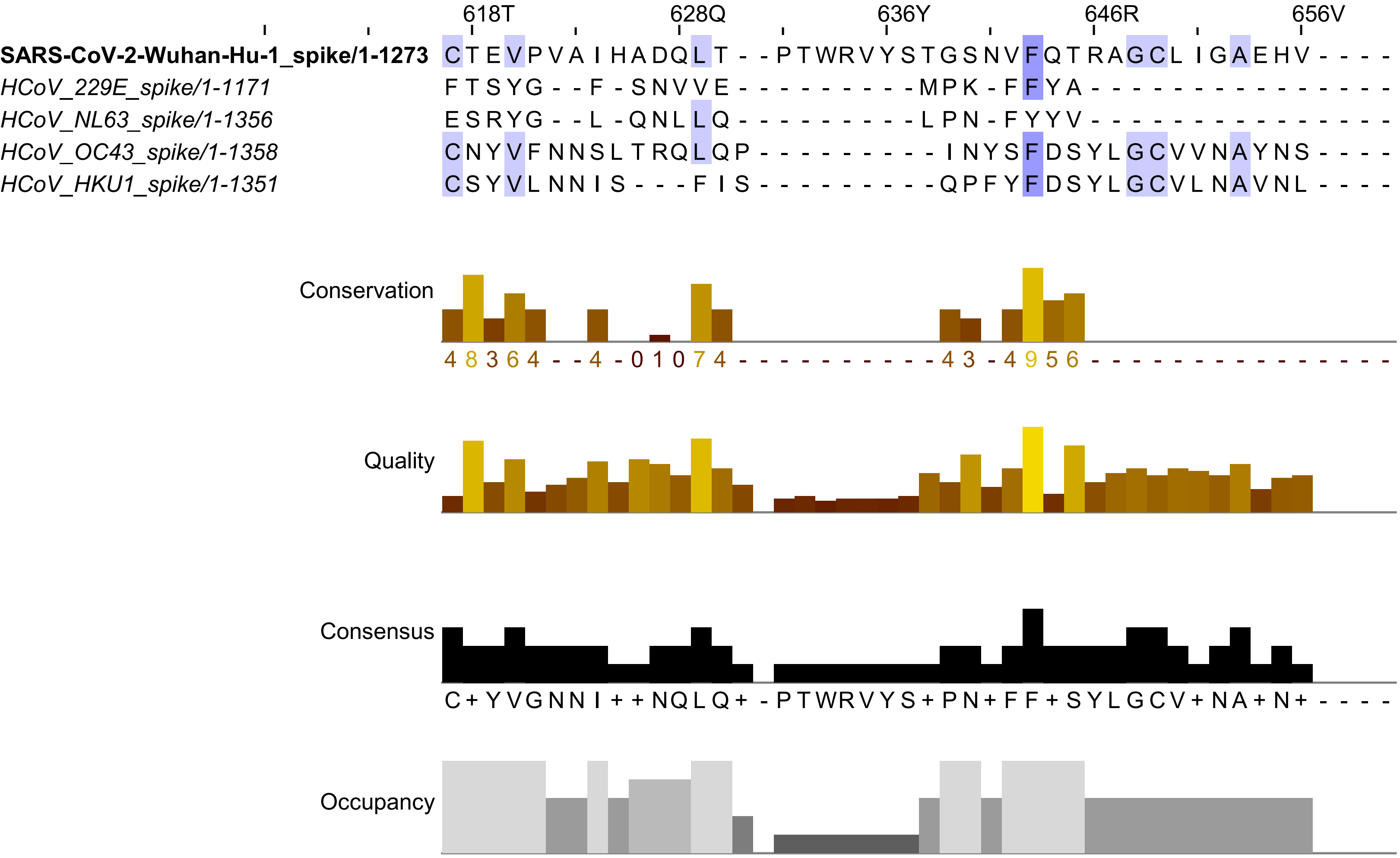
618T
628Q
636Y
646R
656V
SARS-CoV-2-Wuhan-Hu-1_spike/1-1273
HCoV_229E_spike/1-1171
HCoV_NL63_spike/1-1356
HCoV_OC43_spike/1-1358
HCoV_HKU1_spike/1-1351
CTEVPVAIHADQLT--PTWRVYSTGSNVFQTRAGCLIGAEHV----
FTSYG--F-SNVVE--------MPK-FFYA----------------
ESRYG--L-QNLLQ---------LPN-FYYV---------------
CNYVFNNSLTRQLQP---------INYSFDSYLGCVVNAYNS----
CSYVLNNIS---FIS---------QPFYFDSYLGCVLNAVNL----
Conservation
48364--4-01074----------43-4956---------------
Quality
Consensus
C+YVGNNI++NQLQ+-PTWRVYS+PN+FF+SYLGCV+NA+N+----
Occupancy

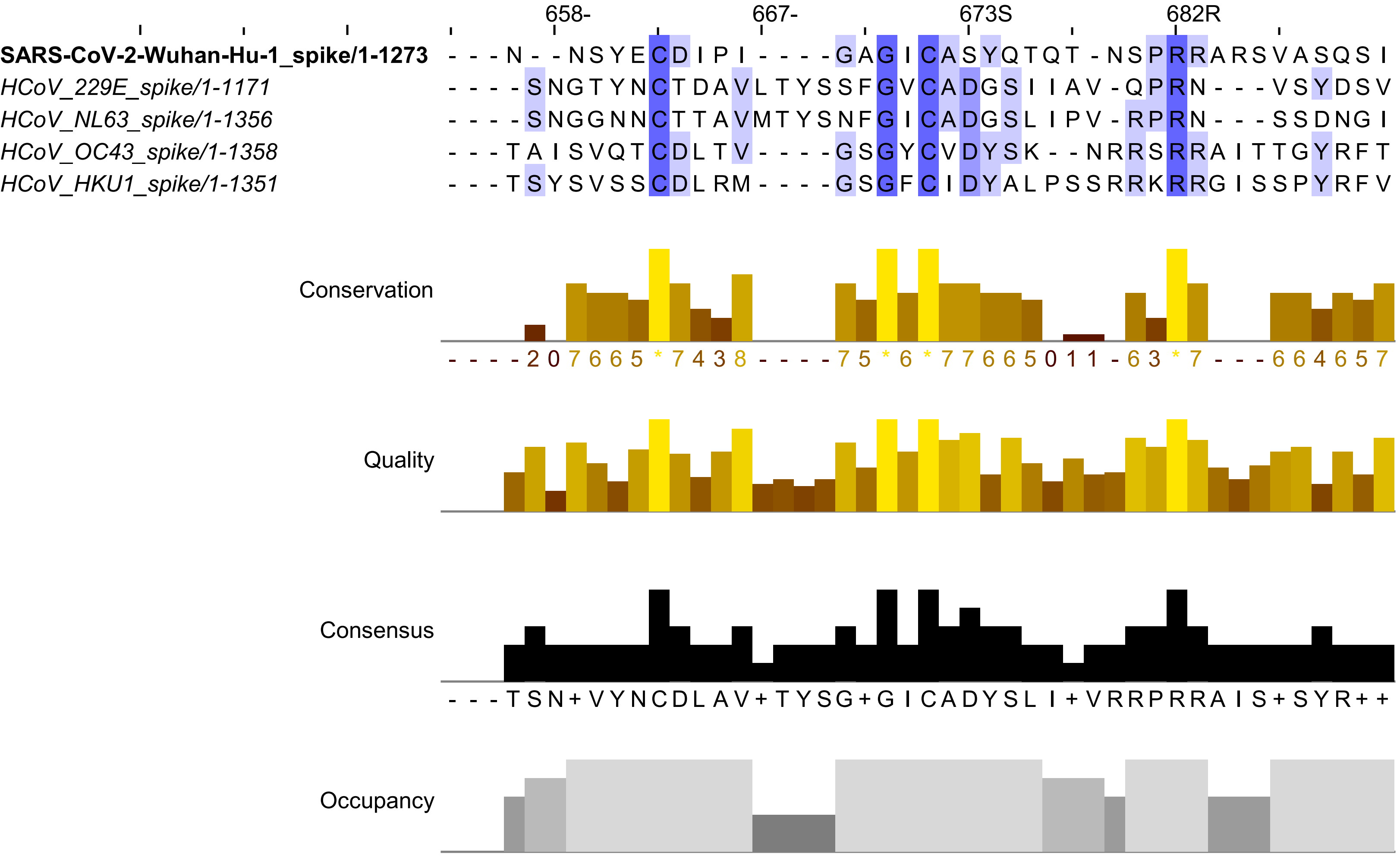

658-
667-
673S
682R
SARS-CoV-2-Wuhan-Hu-1_spike/1-1273 ---N--NSYECDIPI----GAGICASYQTQT-NSPRRARSVASQSI
HCoV_229E_spike/1-1171 ----SNGTYNCTDAVLTYSSFGVCADGSIIAV-QPRN---VSYDSV
HCoV_NL63_spike/1-1356 ----SNGGNNCTTAVMTYSNFGICADGSLIPV-RPRN---SSDNGI
HCoV_OC43_spike/1-1358 ---TAISVQTCDLTV----GSGYCVDYSK--NRRSRRAITTGYRFT
HCoV_HKU1_spike/1-1351 ---TSYSVSSCDLRM----GSGFCIDYALPSSRRKRRGISSPYRFV
Conservation
----207665*7438----75*6*776650011-63*7---664657
Quality
Consensus
---TSN+VYNCDLAV+TYSG+GICADYSLI+VRRPRRAIS+SYR++
Occupancy

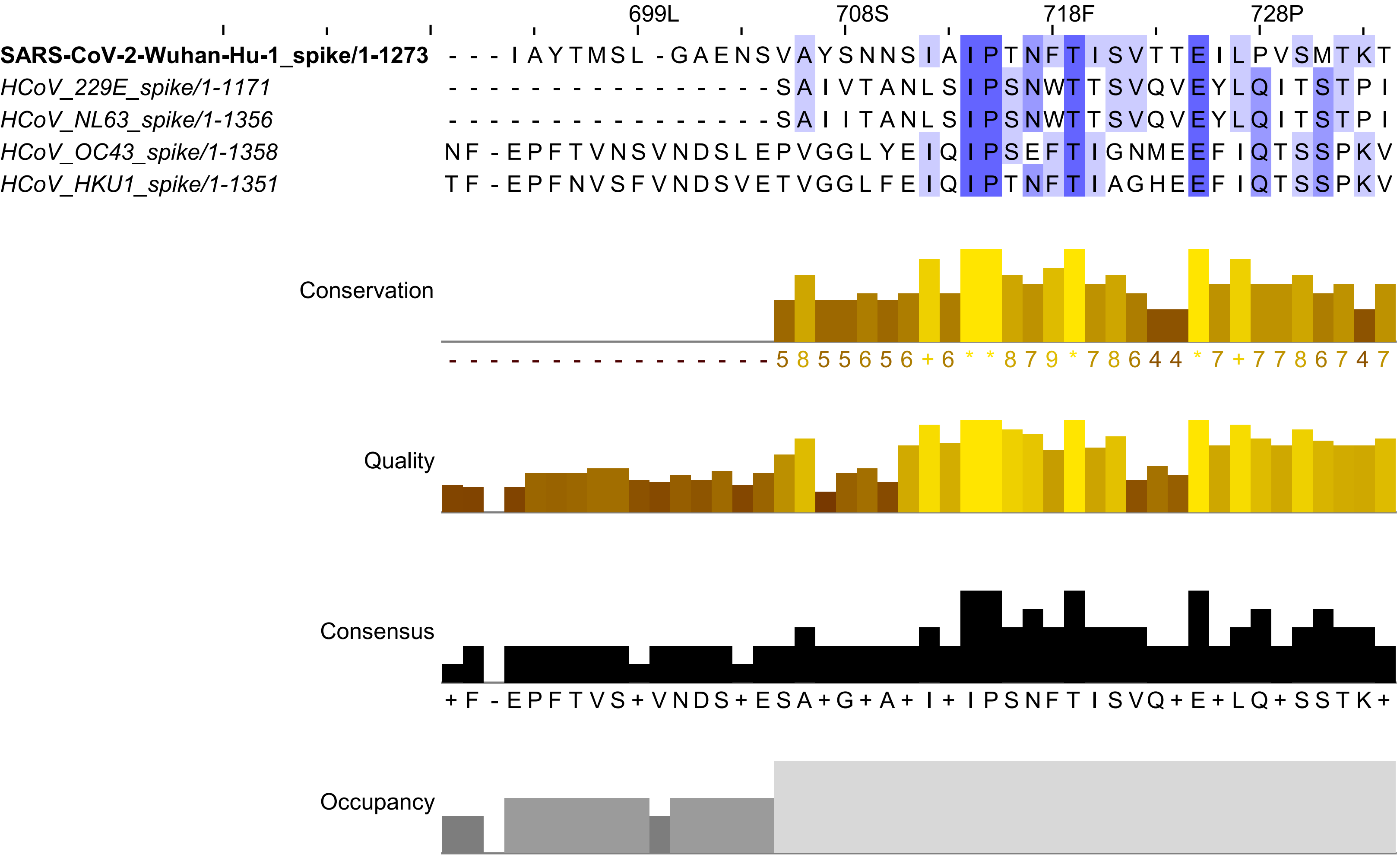
699L
708S
718F
728P
SARS-CoV-2-Wuhan-Hu-1_spike/1-1273 ---IAYTMSL-GAENSVAYSNNSIAIPTNFTISVTTEILPVSMTKT
HCoV_229E_spike/1-1171 -----------------SAIVTANLSIPSNWTTSVQVEYLQITSTPI
HCoV_NL63_spike/1-1356 -----------------SAIITANLSIPSNWTTSVQVEYLQITSTPI
HCoV_OC43_spike/1-1358 NF-EPFTVNSVNDSLEPVGGLYEIQIPSEFTIGNMEEFIQTSSPKV
HCoV_HKU1_spike/1-1351 TF-EPFNVSFVNDSVETVGGLFEIQIPTNFTIAGHEEFIQTSSPKV
Conservation
-----------------5855656+6**879*78644*7+778674 7
Quality
Consensus
+F-EPFTVS+VNDS+ESA+G+A+I+IPSNFTISVQ+E+LQ+SSTK+
Occupancy

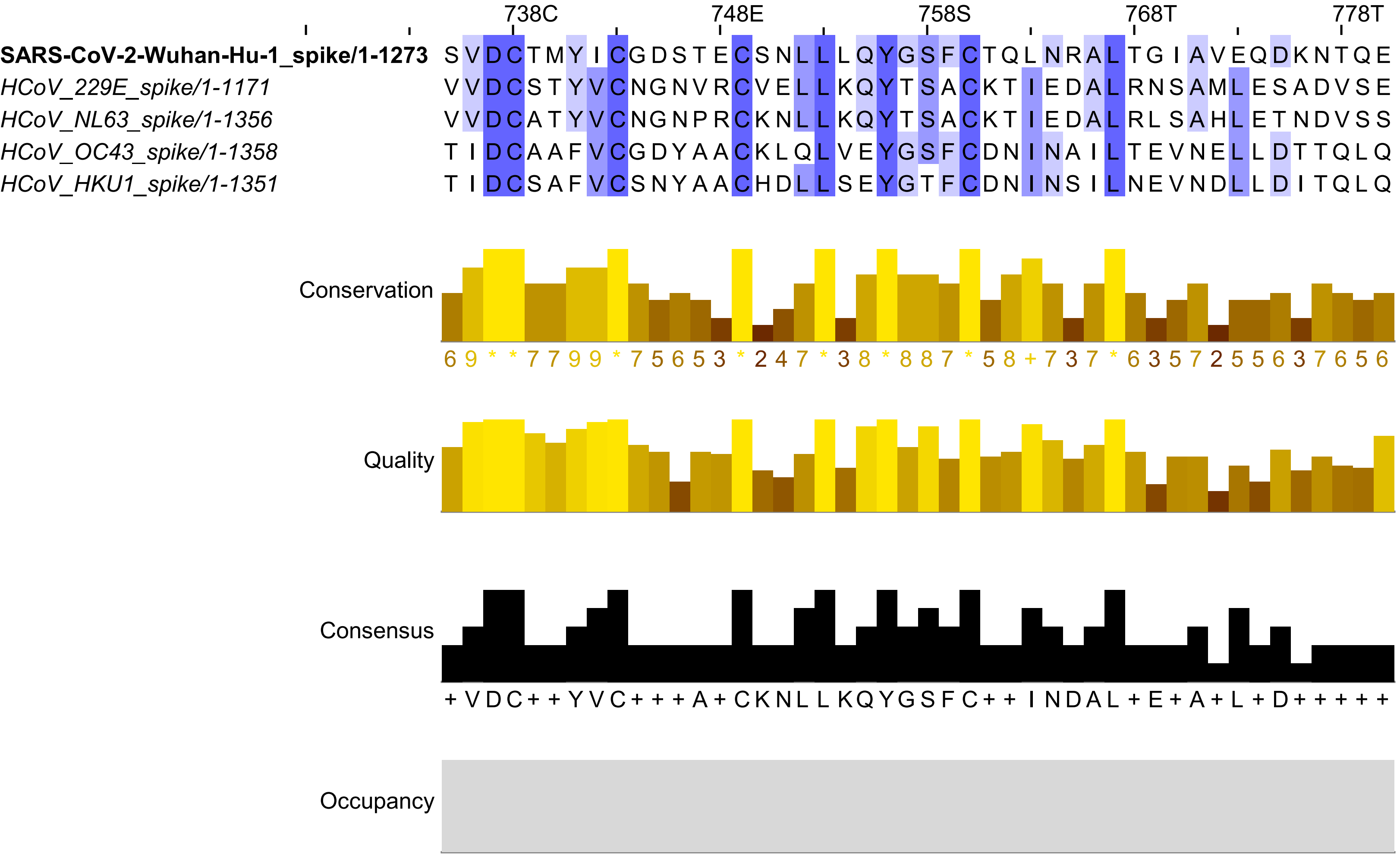
738C
748E
758S
768T
778T
SARS-CoV-2-Wuhan-Hu-1_spike/1-1273 SVDCTMYICGDSTECSNLLLQYGSFCTQLNRALTGIAVEQDKNTQE
HCoV_229E_spike/1-1171 VVDCSTYVCNGNVRCVELLKQYTSACKTIEDALRNSAMLESADVSE
HCoV_NL63_spike/1-1356 VVDCATYVCNGNPRCKNLLKQYTSACKTIEDALRLSAHLETNDVSS
HCoV_OC43_spike/1-1358 TIDCAAFVCGDYAACKLQLVEYGSFCDNINAILTEVNELLDTTQLQ
HCoV_HKU1_spike/1-1351 TIDCSAFVCSNYAACHDLLSEYGTFCDNINSILNEVNDLLDITQLQ
Conservation
69**7799*75653*247*38*887*58+737*6357255637656
Quality
Consensus
+VDC++YVC+++A+CKNLLKQYGSFC++INDAL+E+A+L+D+++++
Occupancy

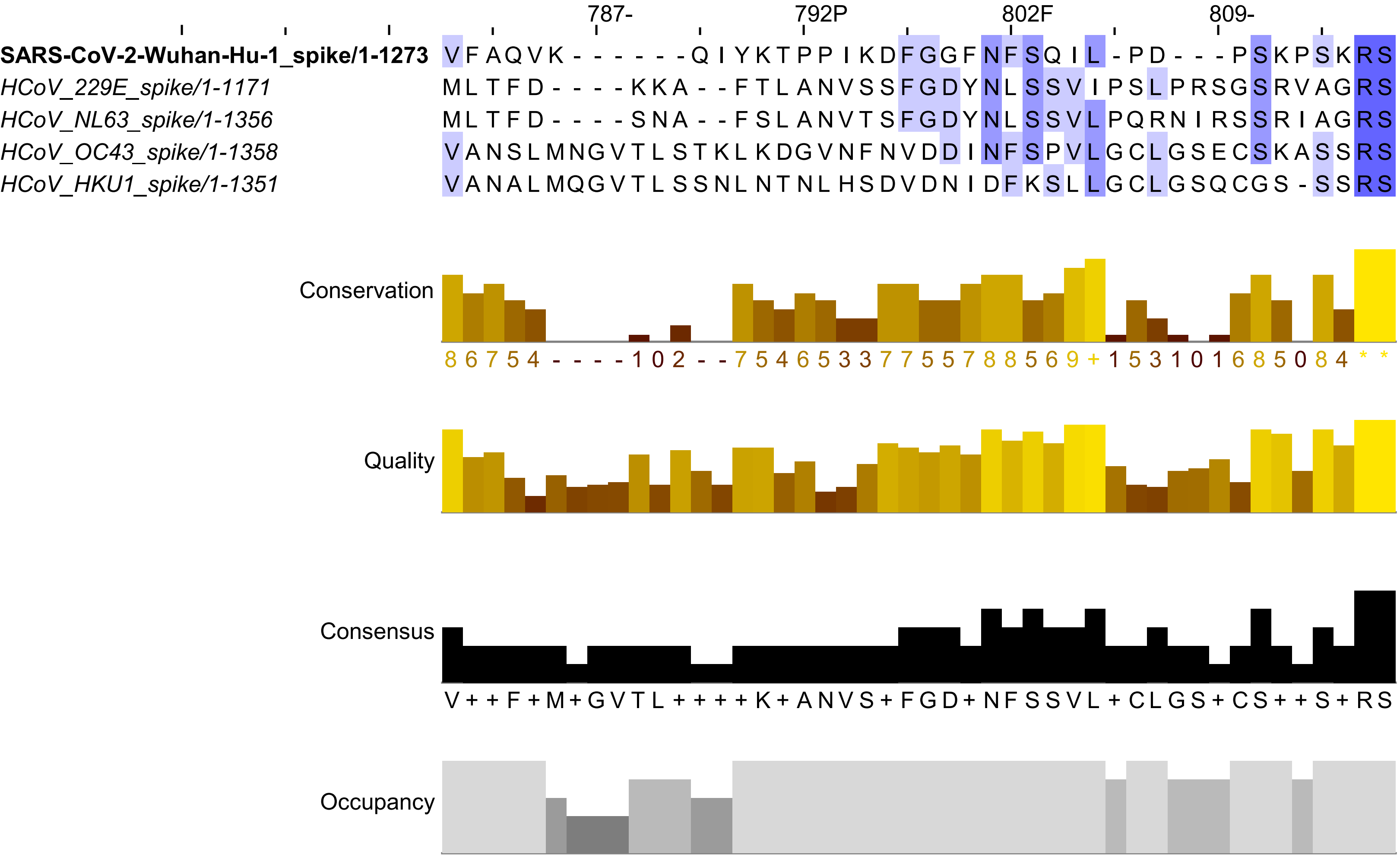

787-
792P
802F
809-
SARS-CoV-2-Wuhan-Hu-1_spike/1-1273
HCoV_229E_spike/1-1171
HCoV_NL63_spike/1-1356
HCoV_OC43_spike/1-1358
HCoV_HKU1_spike/1-1351
Conservation
Quality
Consensus
Occupancy

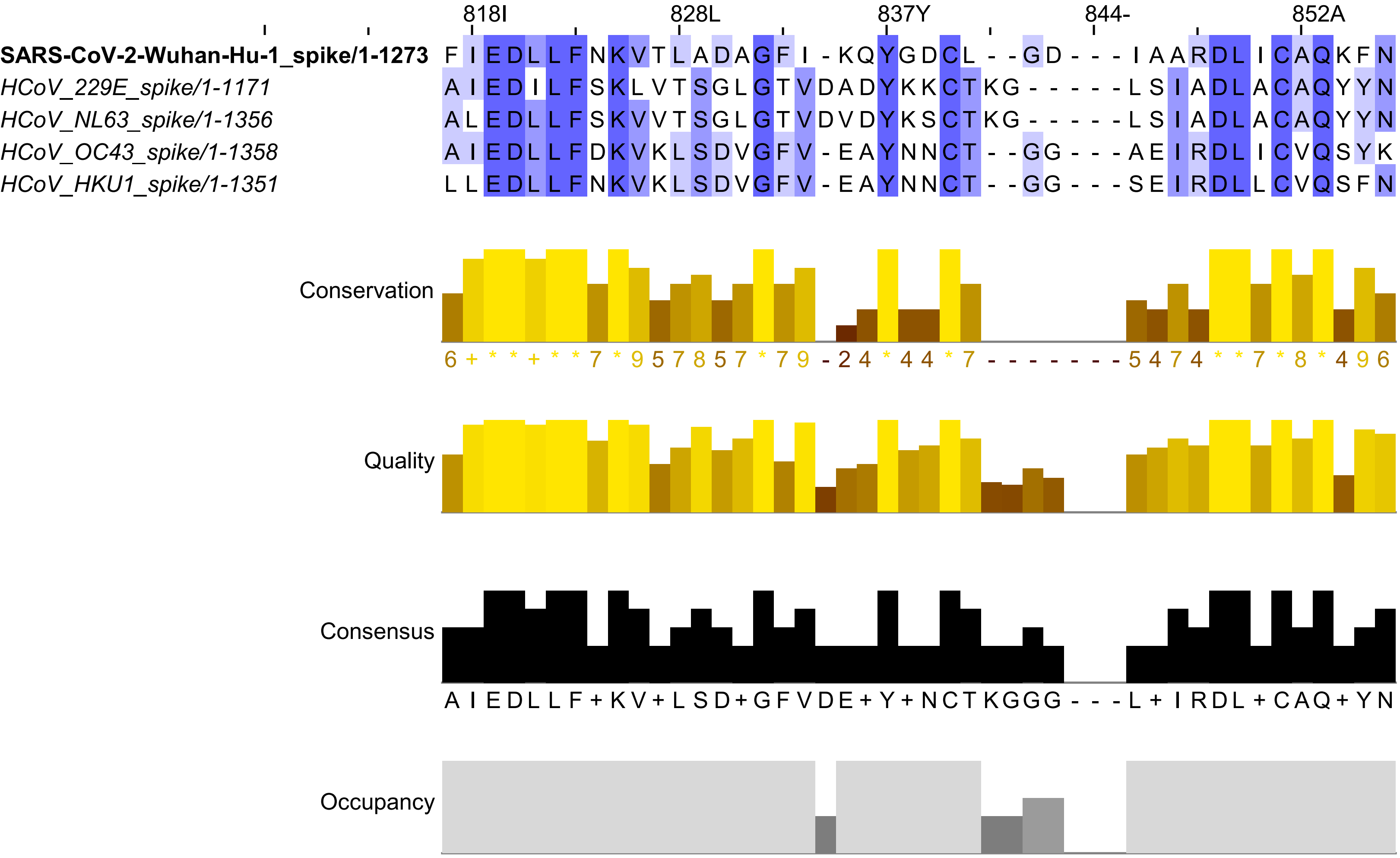

818I
828L
837Y
844-
852A
SARS-CoV-2-Wuhan-Hu-1_spike/1-1273
HCoV_229E_spike/1-1171
HCoV_NL63_spike/1-1356
HCoV_OC43_spike/1-1358
HCoV_HKU1_spike/1-1351
Conservation
Quality
Consensus
Occupancy

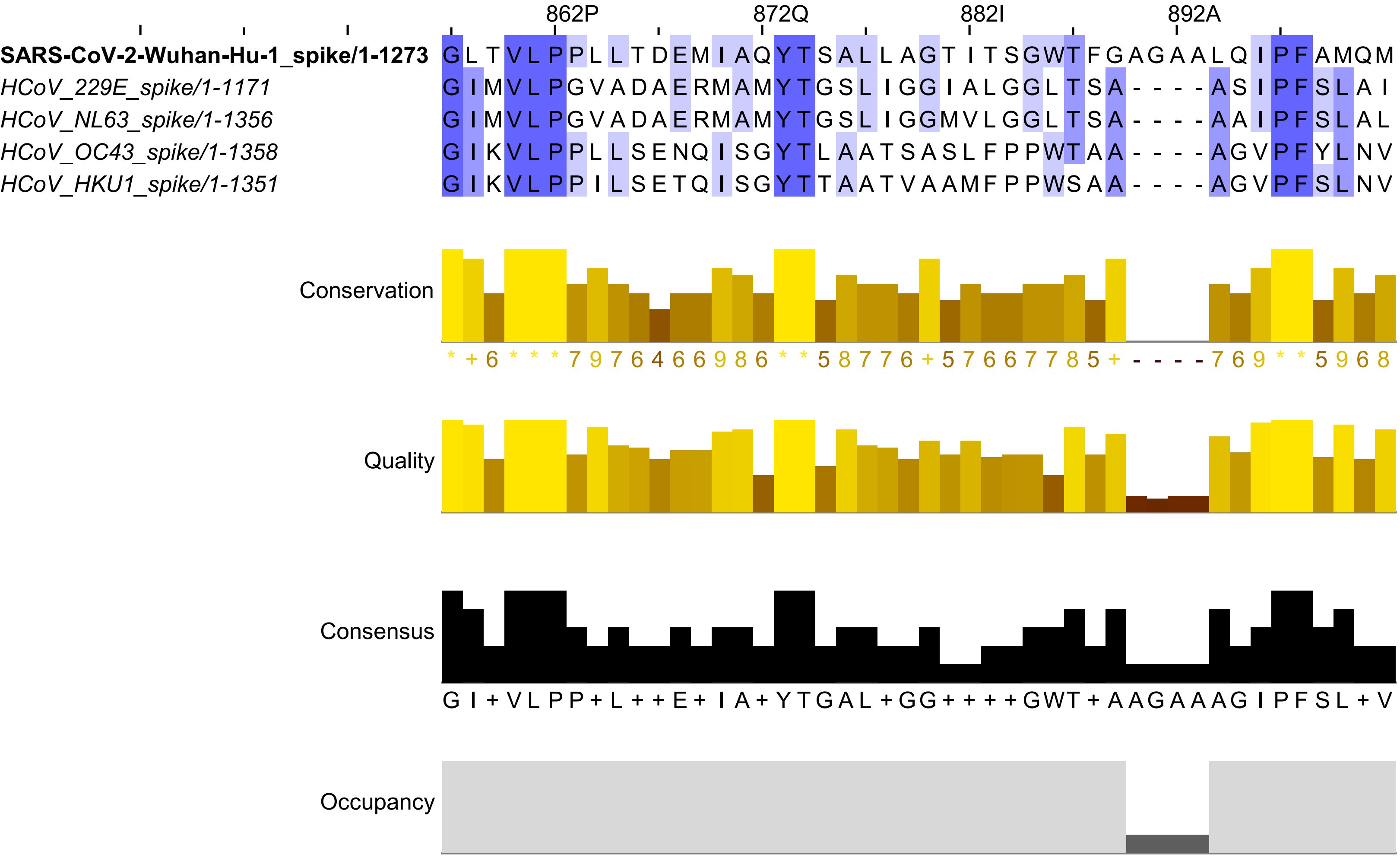

862P
872Q
882I
892A
SARS-CoV-2-Wuhan-Hu-1_spike/1-1273
HCoV_229E_spike/1-1171
HCoV_NL63_spike/1-1356
HCoV_OC43_spike/1-1358
HCoV_HKU1_spike/1-1351
GLTVLPPLLTDEMIAQYTSALLAGTITSGWTFGAGAALQIPFAMQM
GIMVLPGVADAERMAMYTGSLIGGIALGGLTSA----ASIPFSLAI
GIMVLPGVADAERMAMYTGSLIGGMVLGGLTSA----AAIPFSLAL
GIKVLPPLLSENQISGYTLAATSASLFPPWTAA----AGVPFYLNV
GIKVLPPILSETQISGYTTAATVAAMFPPWSAA----AGVPFSLNV
Conservation
*+6***7976466986**58776+57667785+----769**5968
Quality
Consensus
GI+VLPP+L++E+IA+YTGAL+GG++++GWT+AAGAAAGIPFSL+V
Occupancy

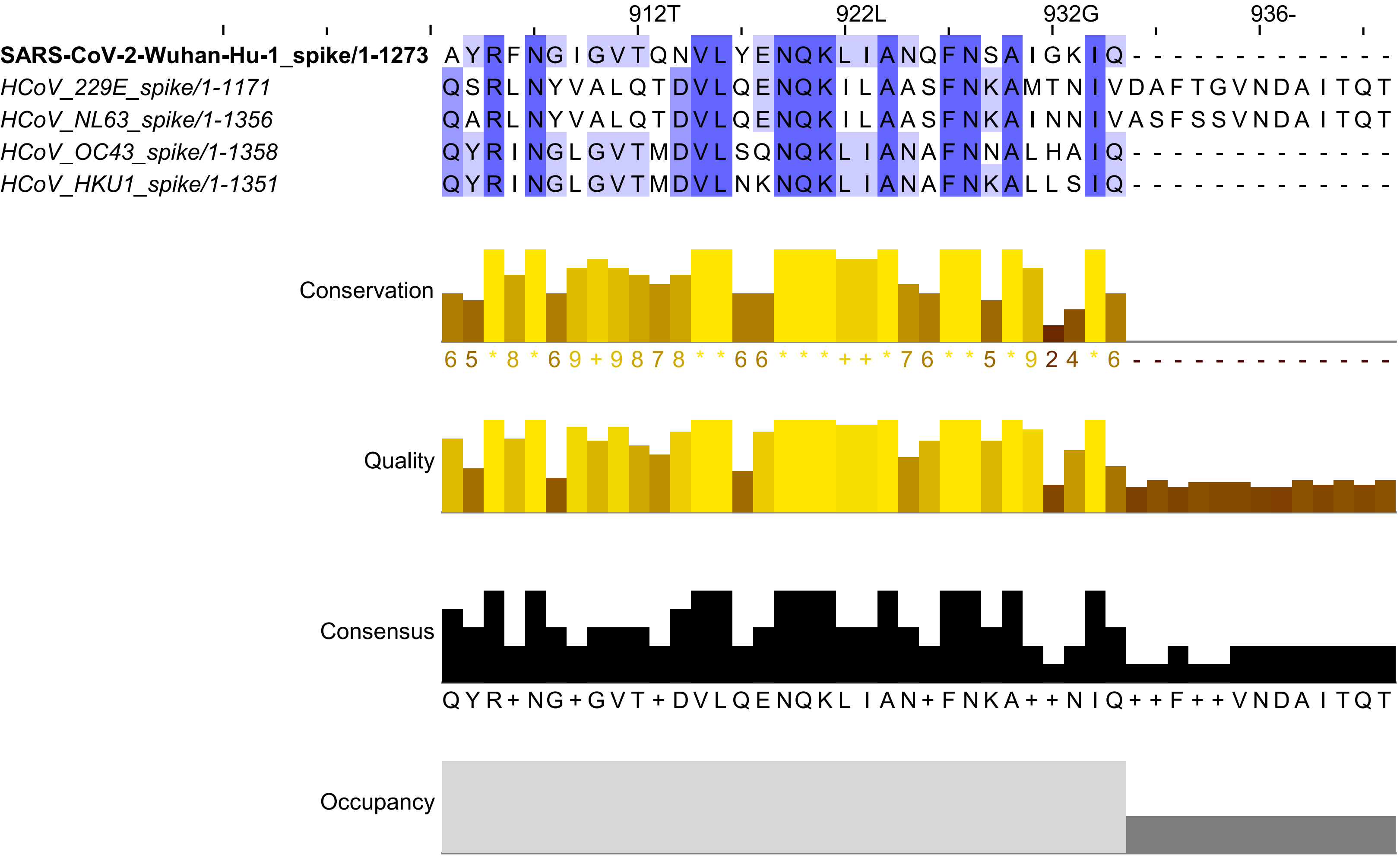
912T
922L
932G
936-
SARS-CoV-2-Wuhan-Hu-1_spike/1-1273 AYRFNGIGVTQNVLYENQKLIANQFNSAIGKIQ-------------
HCoV_229E_spike/1-1171 QSRLNYVALQTDVLQENQKILAASFNKAMTNIVDAFTGVNDAITQT
HCoV_NL63_spike/1-1356 QARLNYVALQTDVLQENQKILAASFNKAINNIVASFSSVNDAITQT
HCoV_OC43_spike/1-1358 QYRINGLGVTMDVLSQNQKLIANAFNNALHAIQ-------------
HCoV_HKU1_spike/1-1351 QYRINGLGVTMDVLNKNQKLIANAFNKALLSIQ-------------
Conservation
65*8*69+9878**66***++*76**5*924*6-------------
Quality
Consensus
QYR+NG+GVT+DVLQENQKLIAN+FNKA++NIQ++F++VNDAITQT
Occupancy

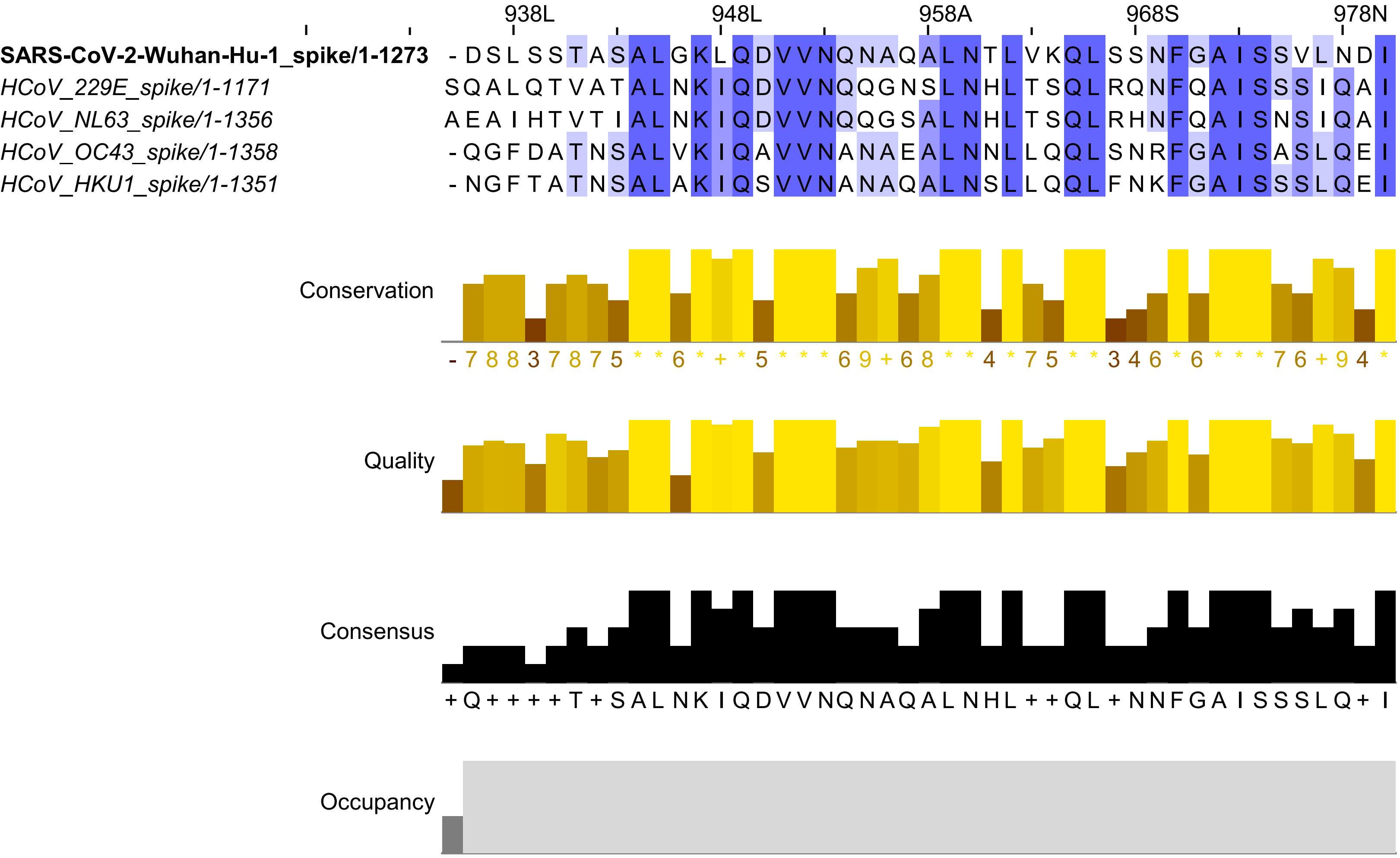

938L
948L
958A
968S
978N
SARS-CoV-2-Wuhan-Hu-1_spike/1-1273 -DSLSSTASALGKLQDVVNQNAQALNTLVKQLSSNFGAISSVLNDI
HCoV_229E_spike/1-1171 SQALQTVATALNKIQDVVNQQGNSLNHLTSQLRQNFQAISSSIQAI
HCoV_NL63_spike/1-1356 AEAIHTVTIALNKIQDVVNQQGSALNHLTSQLRHNFQAISNSIQAI
HCoV_OC43_spike/1-1358 -QGFDATNSALVKIQAVVNANAEALNNLLQQLSNRFGAISASLQEI
HCoV_HKU1_spike/1-1351 -NGFTATNSALAKIQSVVNANAQALNSLLQQLFNKFGAISSSLQEI
Conservation
-78837875**6*+*5***69+68**4*75**346*6***76+94*
Quality
Consensus
+Q+++++T+SALNKIQDVVNQNAQALNHL++QL+NNFGAISSSLQ+I
Occupancy

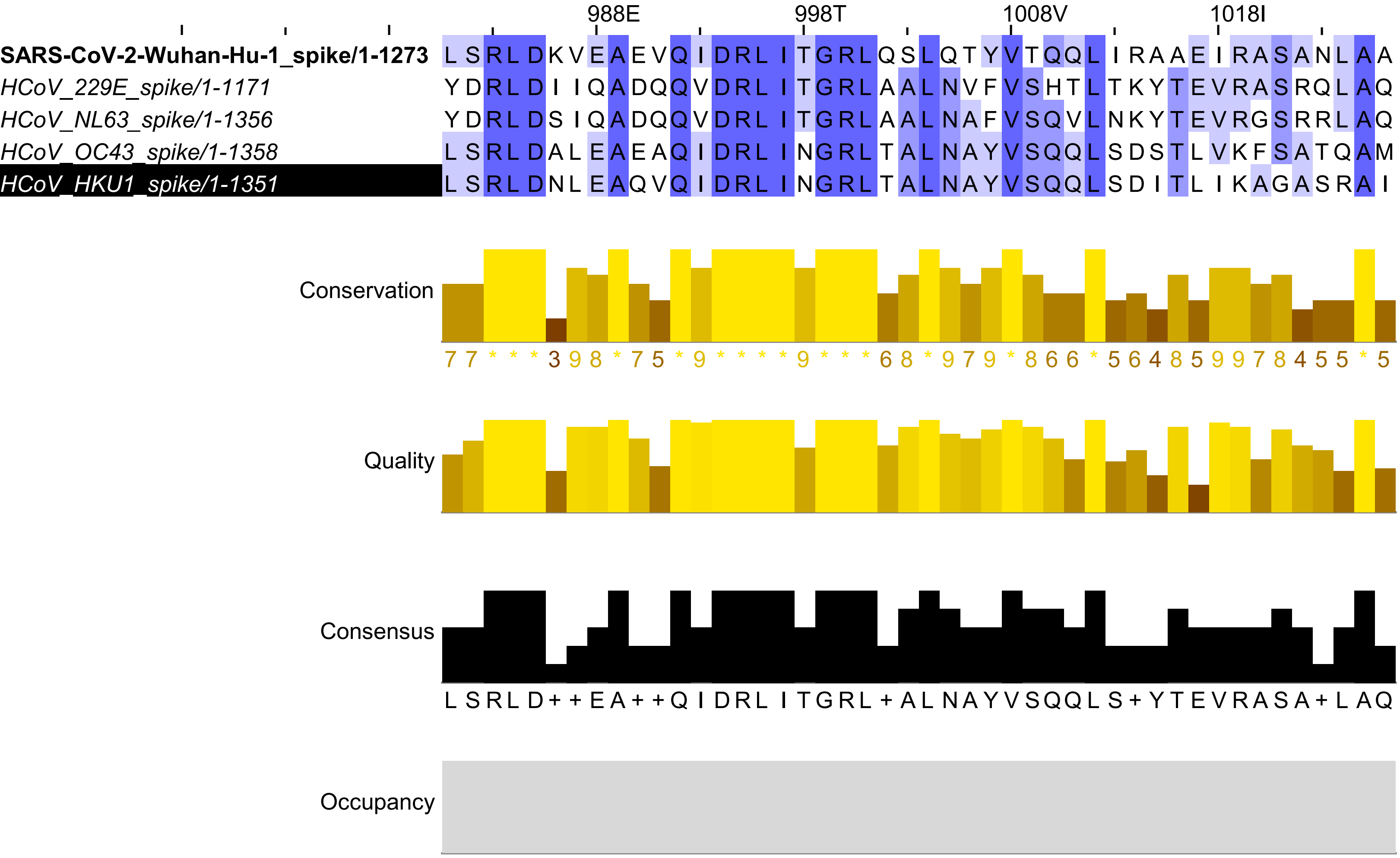
988E
998T
1008V
1018I
SARS-CoV-2-Wuhan-Hu-1_spike/1-1273 LSRLDKVEAEVQIDRLITGRLQSLQTYVTQQLIRAAEIRASANLAA
HCoV_229E_spike/1-1171 YDRLDIIQADQQVDRLITGRLAALNVFVSHTLTKYTEVRASRQLAQ
HCoV_NL63_spike/1-1356 YDRLDSIQADQQVDRLITGRLAALNAFVSQVLNKYTEVRGSRRLAQ
HCoV_OC43_spike/1-1358 LSRLDALEAEAQIDRLINGRLTALNAYVSQQLSDSTLVKFSATQAM
HCoV_HKU1_spike/1-1351 LSRLDNLEAQVQIDRLINGRLTALNAYVSQQLSDITLIKAGASRAI
Conservation
77***398*75*9****9***68*979*866*5648599784 55*5
Quality
Consensus
LSRLD++EA++QIDRLITGRL+ALNAYVSQQLS+YTEVRASA+LAQ
Occupancy

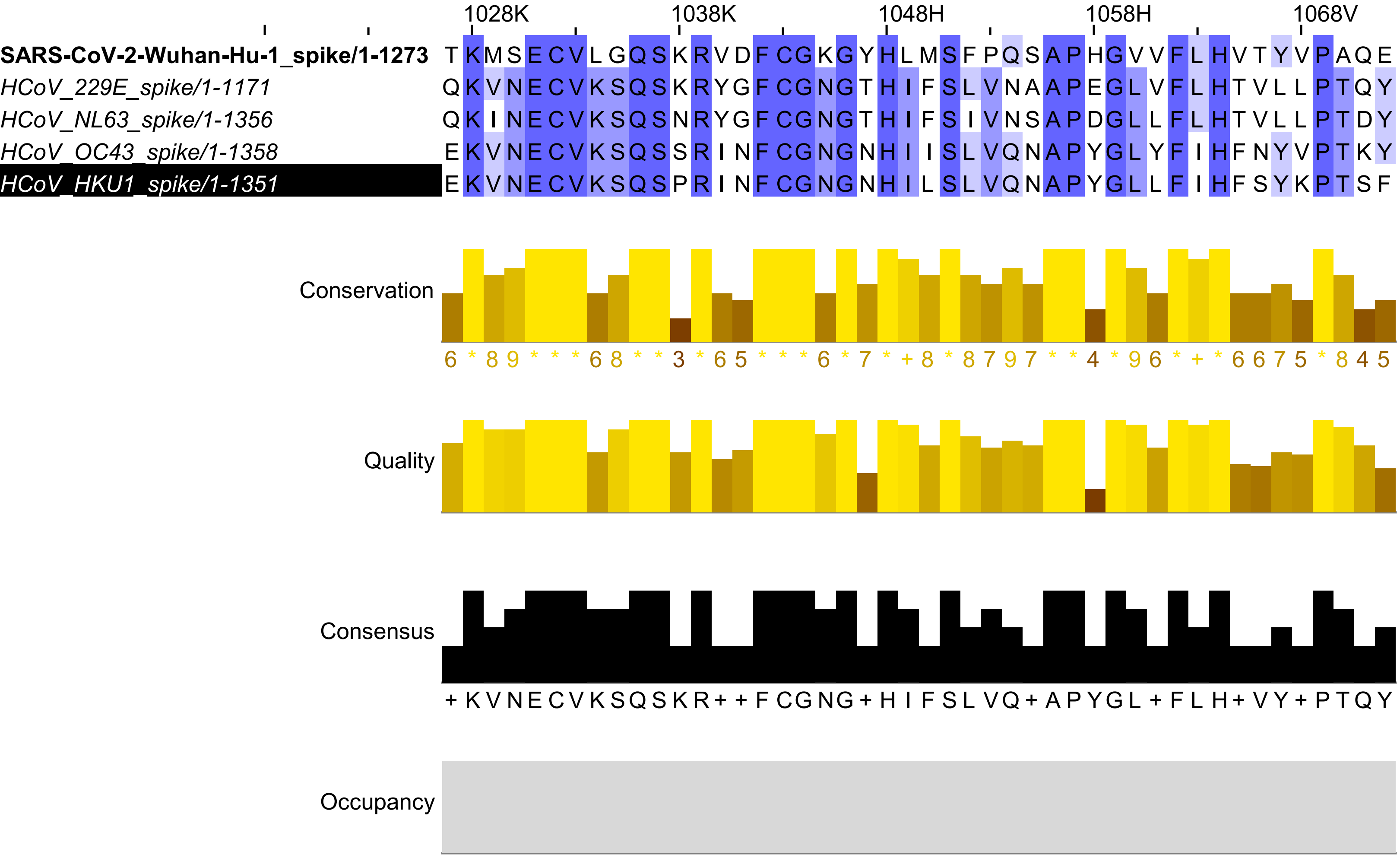
1028K
1038K
1048H
1058H
1068V
SARS-CoV-2-Wuhan-Hu-1_spike/1-1273 TKMSECVLGQSKRVDFCGKGYHLMSFPQSAPHGVVFLHVTYVPAQE
HCoV_229E_spike/1-1171 QKVNECVKSQSKRYGFCGNGTHIFSLVNAAPEGLVFLHTVLLPTQY
HCoV_NL63_spike/1-1356 QKINECVKSQSNRYGFCGNGTHIFSIVNSAPDGLLFLHTVLLPTDY
HCoV_OC43_spike/1-1358 EKVNECVKSQSSRINFCGNGNHIISLVQNAPYGLYFIHFNYVPTKY
HCoV_HKU1_spike/1-1351 EKVNECVKSQSPRINFCGNGNHILSLVQNAPYGLLFIHFSYKPTSF
Conservation
6*89***68**3*65***6*7*+8*8797**4*96*+*6675*845
Quality
Consensus
+KVNECVKSQSKR++FCGNG+HIFSLVQ+APYGL+FLH+VY+PTQY
Occupancy

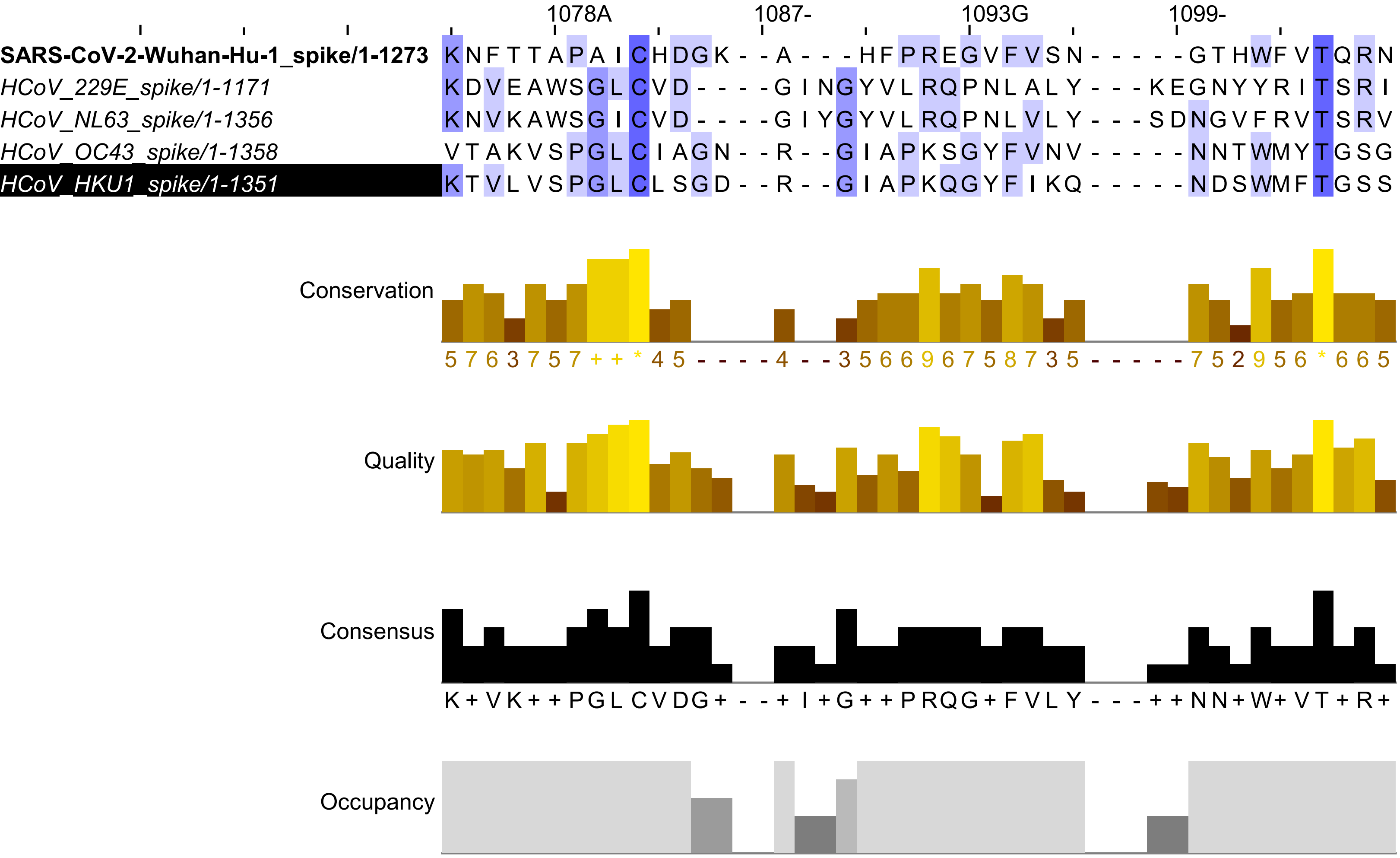
1078A
1087-
1093G
1099-
SARS-CoV-2-Wuhan-Hu-1_spike/1-1273 KNFTTAPAICHDGK--A---HFPREGVFVSN-----GTHWFVTQRN
HCoV_229E_spike/1-1171 KDVEAWSGLCVD----GINGYVLRQPNLALY---KEGNYYRITSRI
HCoV_NL63_spike/1-1356 KNVKAWSGICVD----GIYGYVLRQPNLVLY---SDNGVFRVTSRV
HCoV_OC43_spike/1-1358 VTAKVSPGLCIAGN--R--GIAPKSGYFVNV-----NNTWMYTGSG
HCoV_HKU1_spike/1-1351 KTVLVSPGLCLSGD--R--GIAPKQGYFIKQ-----NDSWMFTGSS
Conservation
5763757++*45-----4---35669675873 5-----752956*665
Quality
Consensus
K+VK++PGLCVDG+--+I+G++PRQG+FVLY---++NN+W+VT+R+
Occupancy

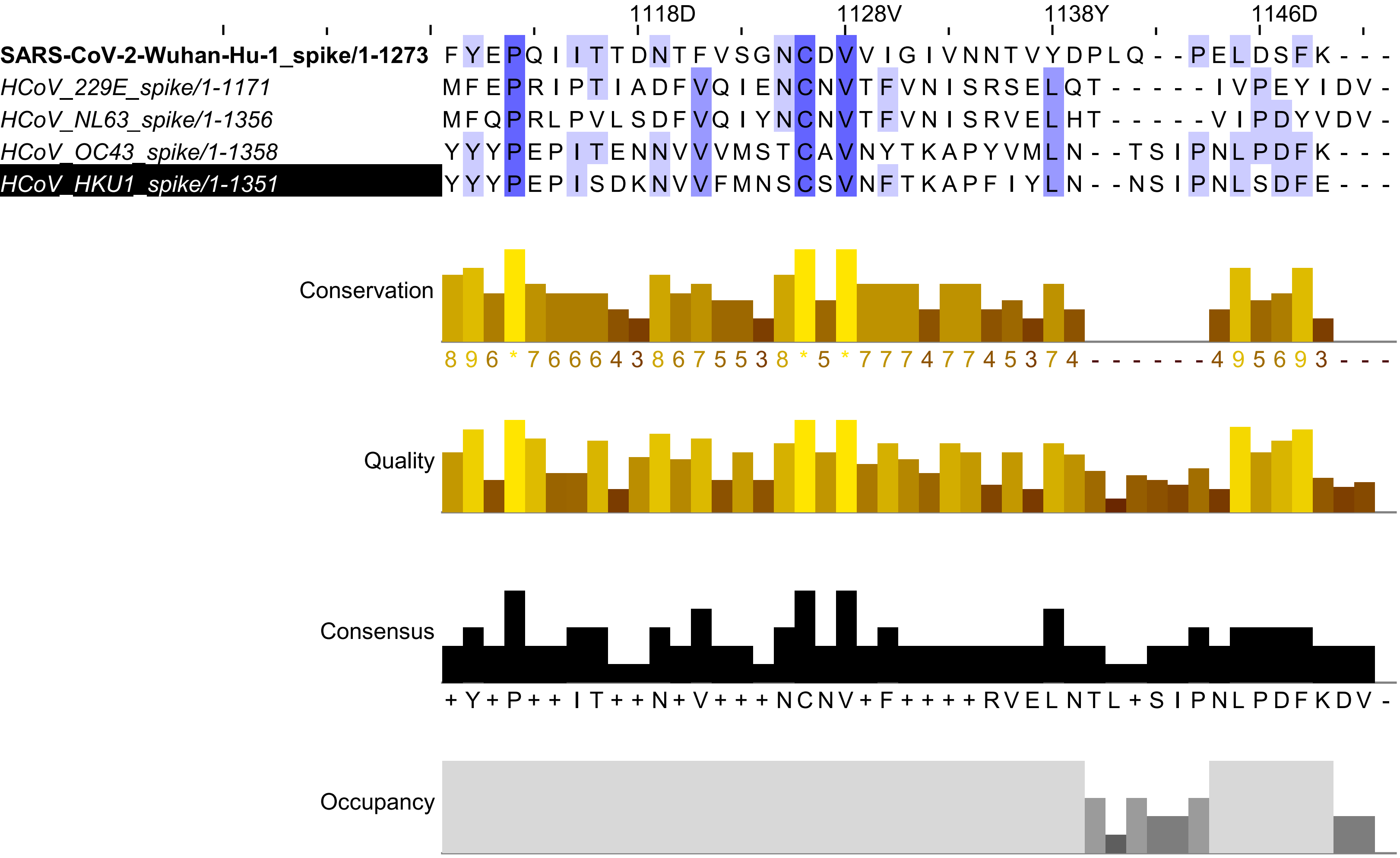
1118D
1128V
1138Y
1146D
SARS-CoV-2-Wuhan-Hu-1_spike/1-1273 FYEPQIITTDNTFVSGNCDVVIGIVNNTVYDPLQ--PELDSFK---
HCoV_229E_spike/1-1171 MFEPRIPTIADFVQIENCNVTFVNISRSELQT-----IVPEYIDV-
HCoV_NL63_spike/1-1356 MFQPRLPVLSDFVQIYNCNVTFVNISRVELHT-----VIPDYVDV-
HCoV_OC43_spike/1-1358 YYYPEPITENNVVVMSTCAVNYTKAPYVMLN--TSIPNLPDFK---
HCoV_HKU1_spike/1-1351 YYYPEPISDKNVVFMNSCSVNFTKAPFIYLN--NSIPNLSDFE---
Conservation
896*7666438675538*5*77747745374------49569 3---
Quality
Consensus
+Y+P++IT++N+V+++NCNV+F++++RVELNTL+SIPNLPDFKDV-
Occupancy

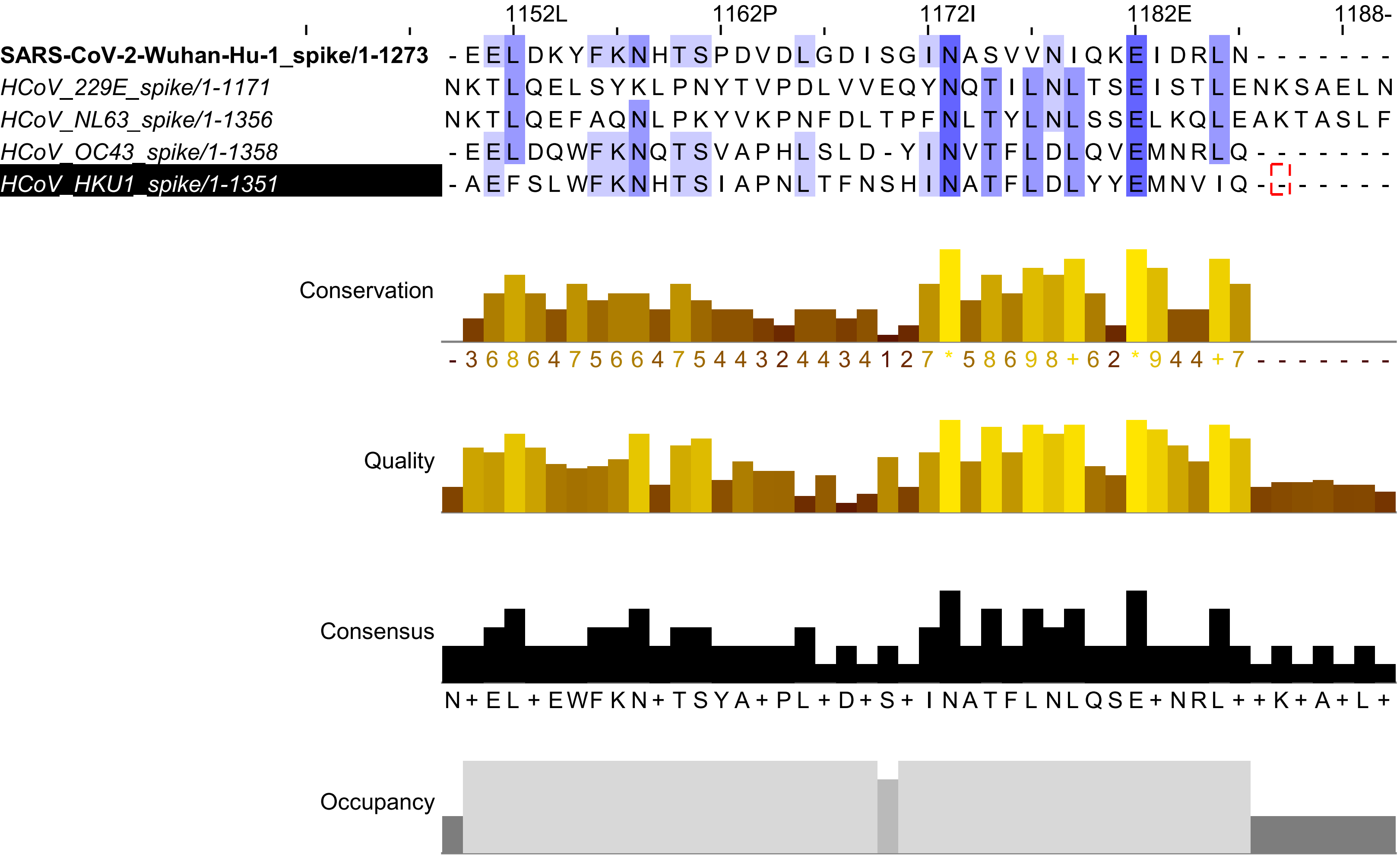
1152L
1162P
1172I
1182E
1188-
SARS-CoV-2-Wuhan-Hu-1_spike/1-1273
HCoV_229E_spike/1-1171
HCoV_NL63_spike/1-1356
HCoV_OC43_spike/1-1358
HCoV_HKU1_spike/1-1351
- E E L D K Y F K N H T S P D V D L G D I S G I N A S V V N I Q K E I D R L N - - - - - - - -
N K T L Q E L S Y K L P N Y T V P D L V V E Q Y N Q T I L N L T S E I S T L E N K S A E L N
N K T L Q E F A Q N L P K Y V K P N F D L T P F N L T Y L N L S S E L K Q L E A K T A S L F
- E E L D Q W F K N Q T S V A P H L S L D - Y I N V T F L D L Q V E M N R L Q - - - - - - - -
- A E F S L W F K N H T S I A P N L T F N S H I N A T F L D L Y Y E M N V I Q - - - - - - - -
Conservation
- 3 6 8 6 4 7 5 6 6 4 7 5 4 4 3 2 4 4 3 4 1 2 7 * 5 8 6 9 8 + 6 2 * 9 4 4 + 7 - - - - - - - -
Quality
Consensus
N + E L + E W F K N + T S Y A + P L + D + S + I N A T F L N L Q S E + N R L + + K + A + L +
Occupancy

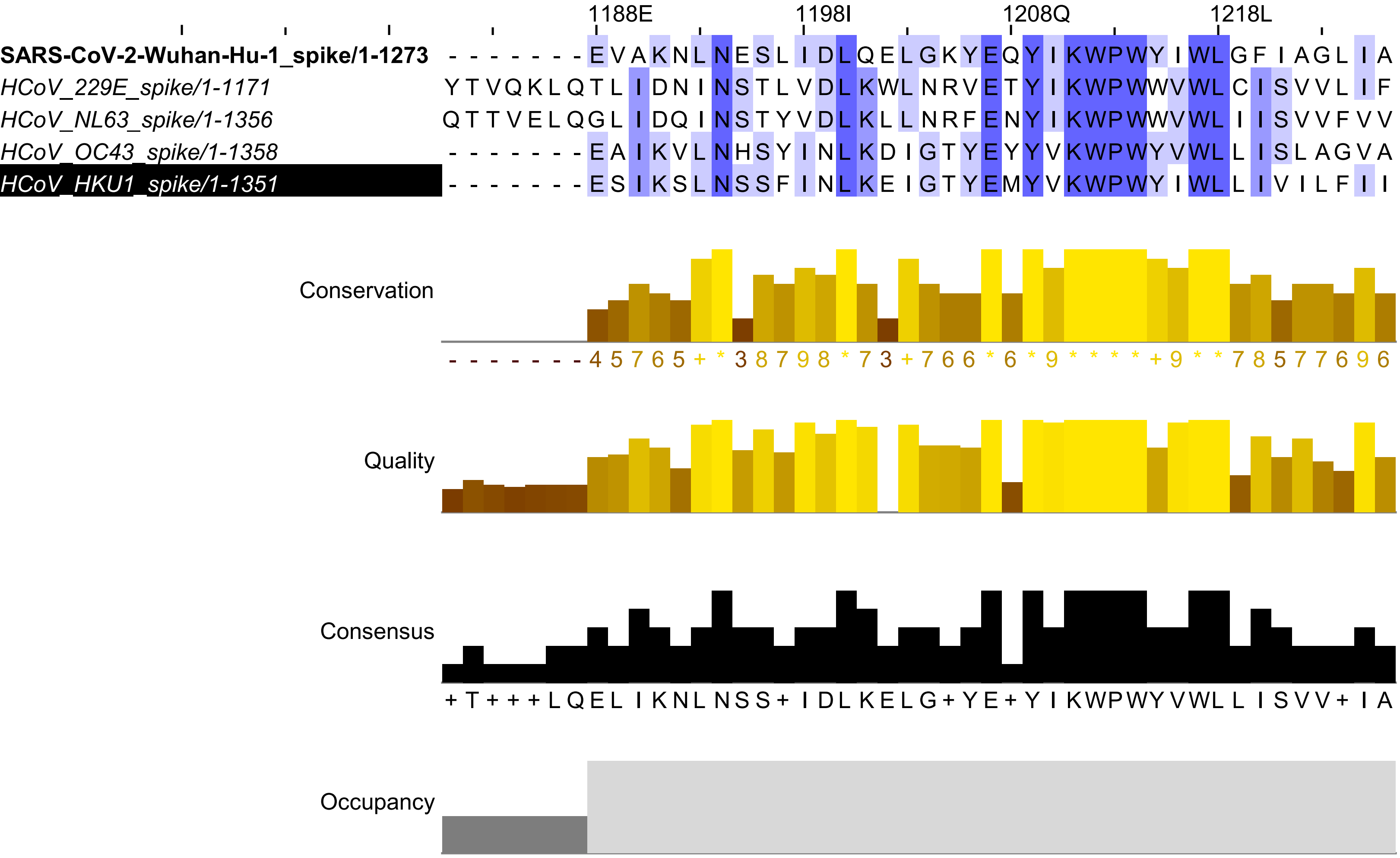
1188E
1198I
1208Q
1218L
SARS-CoV-2-Wuhan-Hu-1_spike/1-1273 -------EVAKNLNESLIDLQELGKYEQYIKWPWYIWLGFIAGLIA
HCoV_229E_spike/1-1171 YTVQKLQTLIDNINSTLVDLKWLNRVETYIKWPWWVWLCISVVLIF
HCoV_NL63_spike/1-1356 QTTVELQGLIDQINSTYVDLKLLNRFENYIKWPWWVWLIISVVFVV
HCoV_OC43_spike/1-1358 -------EAIKVLNHSYINLKDIGTYEYYVKWPWYVWLLISLAGVA
HCoV_HKU1_spike/1-1351 -------ESIKSLNSSFINLKEIGTYEMYVKWPWYIWLLIVILFII
Conservation
-------45765+*38798*73+766*6*9****+9**78577696
Quality
Consensus
+T+++LQELIKNLNSS+IDLKELG+YE+YIKWPWYVWLLISVV+IA
Occupancy

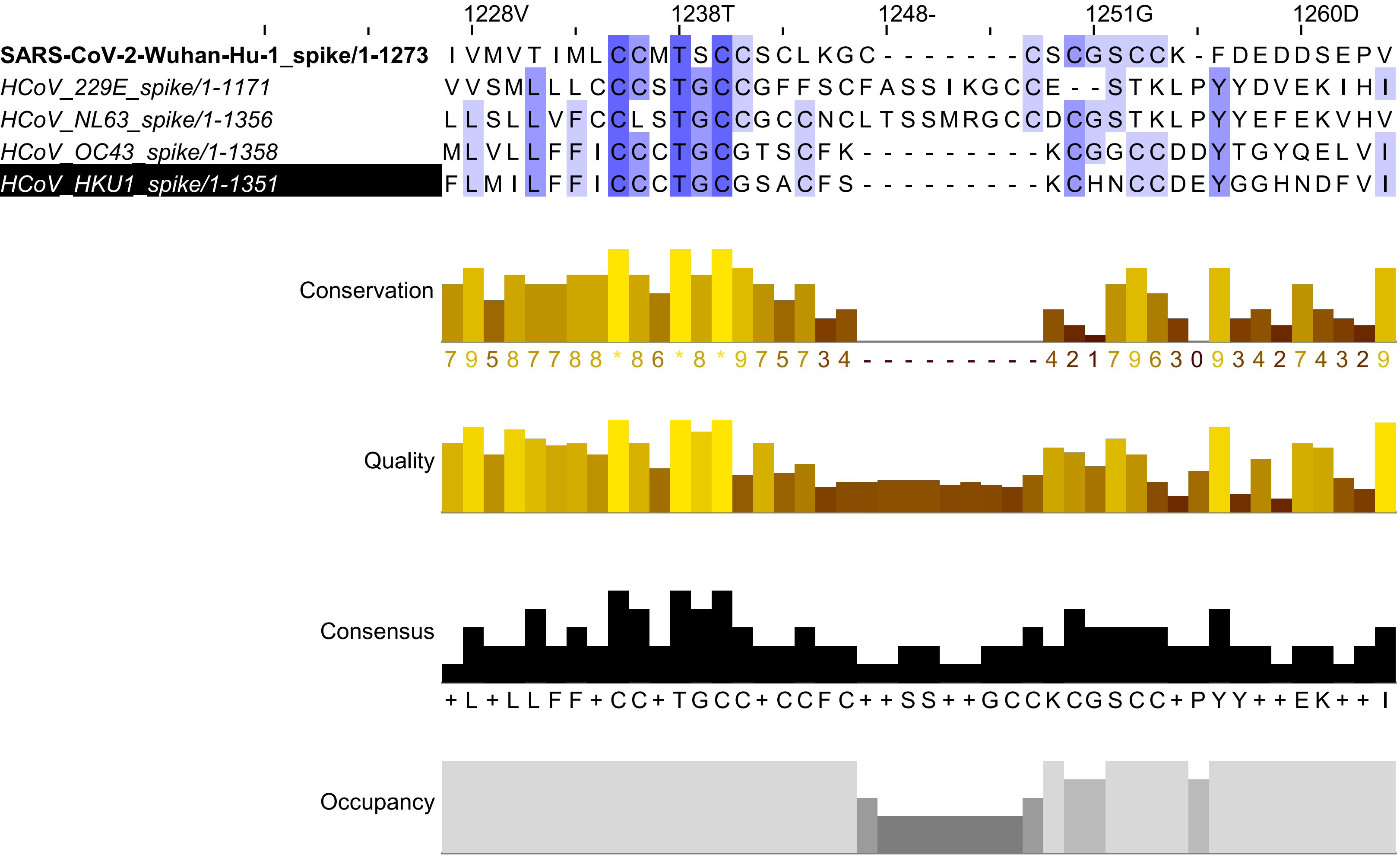
1228V
1238T
1248-
1251G
1260D
SARS-CoV-2-Wuhan-Hu-1_spike/1-1273 IVMVTIMLCCMTSCCSCLKGC--------CSCGSCCK-FDEDDSEPV
HCoV_229E_spike/1-1171 VVSMLLLCCCSTGCCGFFSCFASSIKGCCE--STKLPYYDVEKIHI
HCoV_NL63_spike/1-1356 LLSLLVFCCLSTGCCGCCNCLTSSMRGCCDCGSTKLPYYEFEKVHV
HCoV_OC43_spike/1-1358 MLVLLFFICCCTGCGTSCFK----------KCGGCCDDYTGYQELVI
HCoV_HKU1_spike/1-1351 FLMILFFICCCTGCGSACFS----------KCHNCCDEYGGHNDFVI
Conservation
7958778 8*86*8*97573 4---------4217963093427432 9
Quality
Consensus
+L+LLFF+CC+TGCC+CCFC++SS++GCCKCGSCC+PYY++EK++I
Occupancy

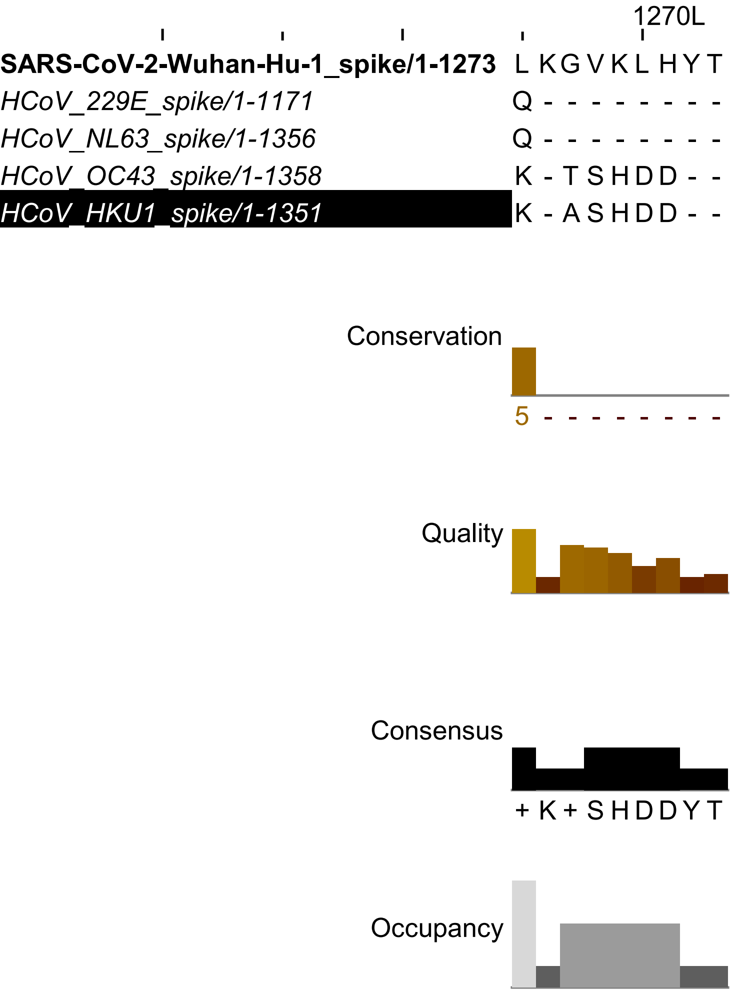
1270L
SARS-CoV-2-Wuhan-Hu-1_spike/1-1273 L K G V K L H Y T
HCoV_229E_spike/1-1171 Q - - - - - - - -
HCoV_NL63_spike/1-1356 Q - - - - - - - -
HCoV_OC43_spike/1-1358 K - T S H D D - -
HCoV_HKU1_spike/1-1351 K - A S H D D - -
Conservation
5 - - - - - - - -
Quality
Consensus
+ K + S H D D Y T
Occupancy
