# Supplementary material for: An Immunoinformatic Approach for Identifying and Designing Conserved Multi-Epitope Vaccines for Coronaviruses
Source: Biomedicines. 2024 Nov 5;12(11):2530. doi: 10.3390/biomedicines12112530 (PMC11592158; doi:10.3390/biomedicines12112530)
Supplement: Supplementary file 1 [file biomedicines-12-02530-s001.zip › Supplementary Figure S3. SARS and MERS CoV alignment.pdf]

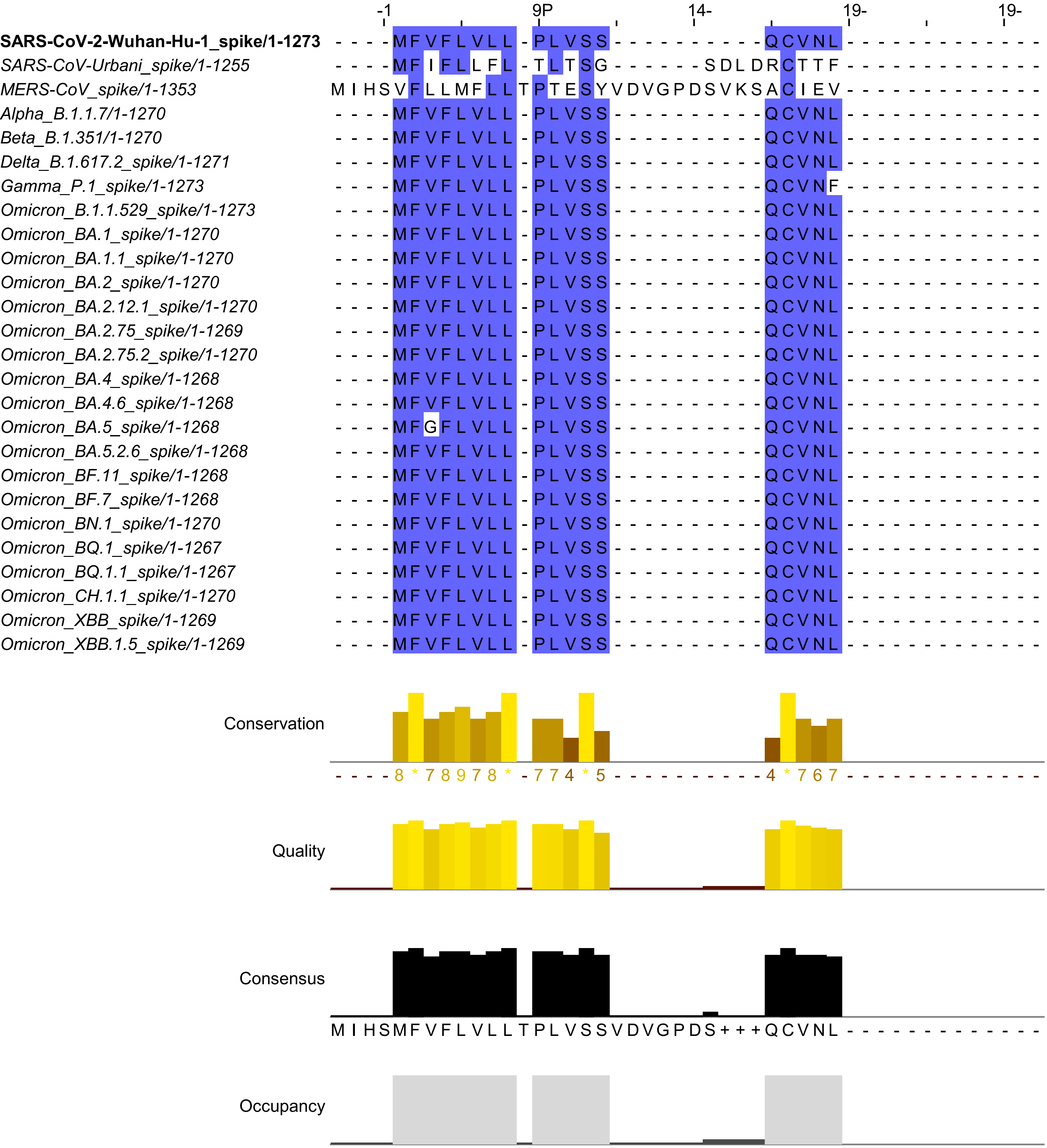

-1
9P
14-
19-
19-
SARS-CoV-2-Wuhan-Hu-1_spike/1-1273 ----MFVFLVLL-PLVSS----------QCVNL-------------
SARS-CoV-Urbani_spike/1-1255 ----MFIFLLFL-TLTSG------SDLDRCTTF-------------
MERS-CoV_spike/1-1353 MIHSVFLLMFLLTPTESYVDVGPDSVKSACIEV-------------
Alpha_B.1.1.7/1-1270 ----MFVFLVLL-PLVSS----------QCVNL-------------
Beta_B.1.351/1-1270 ----MFVFLVLL-PLVSS----------QCVNL-------------
Delta_B.1.617.2_spike/1-1271 ----MFVFLVLL-PLVSS----------QCVNL-------------
Gamma_P.1_spike/1-1273 ----MFVFLVLL-PLVSS----------QCVNF-------------
Omicron_B.1.1.529_spike/1-1273 ----MFVFLVLL-PLVSS----------QCVNL-------------
Omicron_BA.1_spike/1-1270 ----MFVFLVLL-PLVSS----------QCVNL-------------
Omicron_BA.1.1_spike/1-1270 ----MFVFLVLL-PLVSS----------QCVNL-------------
Omicron_BA.2_spike/1-1270 ----MFVFLVLL-PLVSS----------QCVNL-------------
Omicron_BA.2.12.1_spike/1-1270 ----MFVFLVLL-PLVSS----------QCVNL-------------
Omicron_BA.2.75_spike/1-1269 ----MFVFLVLL-PLVSS----------QCVNL-------------
Omicron_BA.2.75.2_spike/1-1270 ----MFVFLVLL-PLVSS----------QCVNL-------------
Omicron_BA.4_spike/1-1268 ----MFVFLVLL-PLVSS----------QCVNL-------------
Omicron_BA.4.6_spike/1-1268 ----MFVFLVLL-PLVSS----------QCVNL-------------
Omicron_BA.5_spike/1-1268 ----MFGFLVLL-PLVSS----------QCVNL-------------
Omicron_BA.5.2.6_spike/1-1268 ----MFVFLVLL-PLVSS----------QCVNL-------------
Omicron_BF.11_spike/1-1268 ----MFVFLVLL-PLVSS----------QCVNL-------------
Omicron_BF.7_spike/1-1268 ----MFVFLVLL-PLVSS----------QCVNL-------------
Omicron_BN.1_spike/1-1270 ----MFVFLVLL-PLVSS----------QCVNL-------------
Omicron_BQ.1_spike/1-1267 ----MFVFLVLL-PLVSS----------QCVNL-------------
Omicron_BQ.1.1_spike/1-1267 ----MFVFLVLL-PLVSS----------QCVNL-------------
Omicron_CH.1.1_spike/1-1270 ----MFVFLVLL-PLVSS----------QCVNL-------------
Omicron_XBB_spike/1-1269 ----MFVFLVLL-PLVSS----------QCVNL-------------
Omicron_XBB.1.5_spike/1-1269 ----MFVFLVLL-PLVSS----------QCVNL-------------
Conservation
----8*78978*-774*5----------4*767-------------
Quality
Consensus
MIHSMFVFLVLLTPLVSSVDVGPDS+++QCVNL-------------
Occupancy

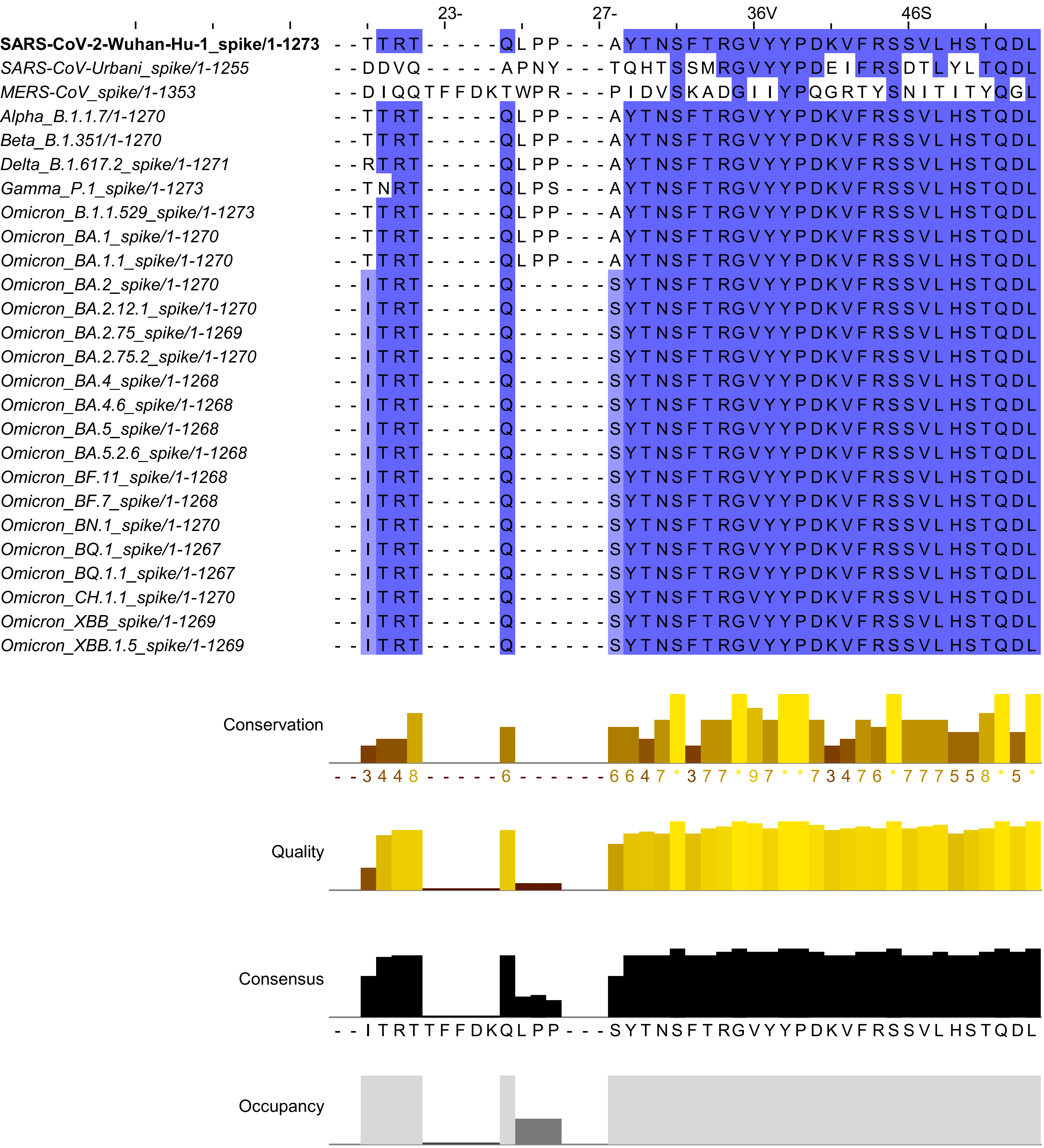
23-
27-
36V
46S
SARS-CoV-2-Wuhan-Hu-1_spike/1-1273 --TTRT-----QLPP---AYTNSFTRGVYYPDKVFRSSVLHSTQDL
SARS-CoV-Urbani_spike/1-1255 --DDVQ-----APNY---TQHTSSMRGVYYPDEIFRSDTLYLTQDL
MERS-CoV_spike/1-1353 --DIQQTFFDKTWPR---PIDVSKADGIIYPQGRTYSNITITYQGL
Alpha_B.1.1.7/1-1270 --TTRT-----QLPP---AYTNSFTRGVYYPDKVFRSSVLHSTQDL
Beta_B.1.351/1-1270 --TTRT-----QLPP---AYTNSFTRGVYYPDKVFRSSVLHSTQDL
Delta_B.1.617.2_spike/1-1271 --RTRT-----QLPP---AYTNSFTRGVYYPDKVFRSSVLHSTQDL
Gamma_P.1_spike/1-1273 --TNRT-----QLPS---AYTNSFTRGVYYPDKVFRSSVLHSTQDL
Omicron_B.1.1.529_spike/1-1273 --TTRT-----QLPP---AYTNSFTRGVYYPDKVFRSSVLHSTQDL
Omicron_BA.1_spike/1-1270 --TTRT-----QLPP---AYTNSFTRGVYYPDKVFRSSVLHSTQDL
Omicron_BA.1.1_spike/1-1270 --TTRT-----QLPP---AYTNSFTRGVYYPDKVFRSSVLHSTQDL
Omicron_BA.2_spike/1-1270 --ITRT-----Q------SYTNSFTRGVYYPDKVFRSSVLHSTQDL
Omicron_BA.2.12.1_spike/1-1270 --ITRT-----Q------SYTNSFTRGVYYPDKVFRSSVLHSTQDL
Omicron_BA.2.75_spike/1-1269 --ITRT-----Q------SYTNSFTRGVYYPDKVFRSSVLHSTQDL
Omicron_BA.2.75.2_spike/1-1270 --ITRT-----Q------SYTNSFTRGVYYPDKVFRSSVLHSTQDL
Omicron_BA.4_spike/1-1268 --ITRT-----Q------SYTNSFTRGVYYPDKVFRSSVLHSTQDL
Omicron_BA.4.6_spike/1-1268 --ITRT-----Q------SYTNSFTRGVYYPDKVFRSSVLHSTQDL
Omicron_BA.5_spike/1-1268 --ITRT-----Q------SYTNSFTRGVYYPDKVFRSSVLHSTQDL
Omicron_BA.5.2.6_spike/1-1268 --ITRT-----Q------SYTNSFTRGVYYPDKVFRSSVLHSTQDL
Omicron_BF.11_spike/1-1268 --ITRT-----Q------SYTNSFTRGVYYPDKVFRSSVLHSTQDL
Omicron_BF.7_spike/1-1268 --ITRT-----Q------SYTNSFTRGVYYPDKVFRSSVLHSTQDL
Omicron_BN.1_spike/1-1270 --ITRT-----Q------SYTNSFTRGVYYPDKVFRSSVLHSTQDL
Omicron_BQ.1_spike/1-1267 --ITRT-----Q------SYTNSFTRGVYYPDKVFRSSVLHSTQDL
Omicron_BQ.1.1_spike/1-1267 --ITRT-----Q------SYTNSFTRGVYYPDKVFRSSVLHSTQDL
Omicron_CH.1.1_spike/1-1270 --ITRT-----Q------SYTNSFTRGVYYPDKVFRSSVLHSTQDL
Omicron_XBB_spike/1-1269 --ITRT-----Q------SYTNSFTRGVYYPDKVFRSSVLHSTQDL
Omicron_XBB.1.5_spike/1-1269 --ITRT-----Q------SYTNSFTRGVYYPDKVFRSSVLHSTQDL
Conservation
--3448-----6------6647*377*97**73476*777558*5*
Quality
Consensus
--ITRTTFFDKQLPP---SYTNSFTRGVYYPDKVFRSSVLHSTQDL
Occupancy

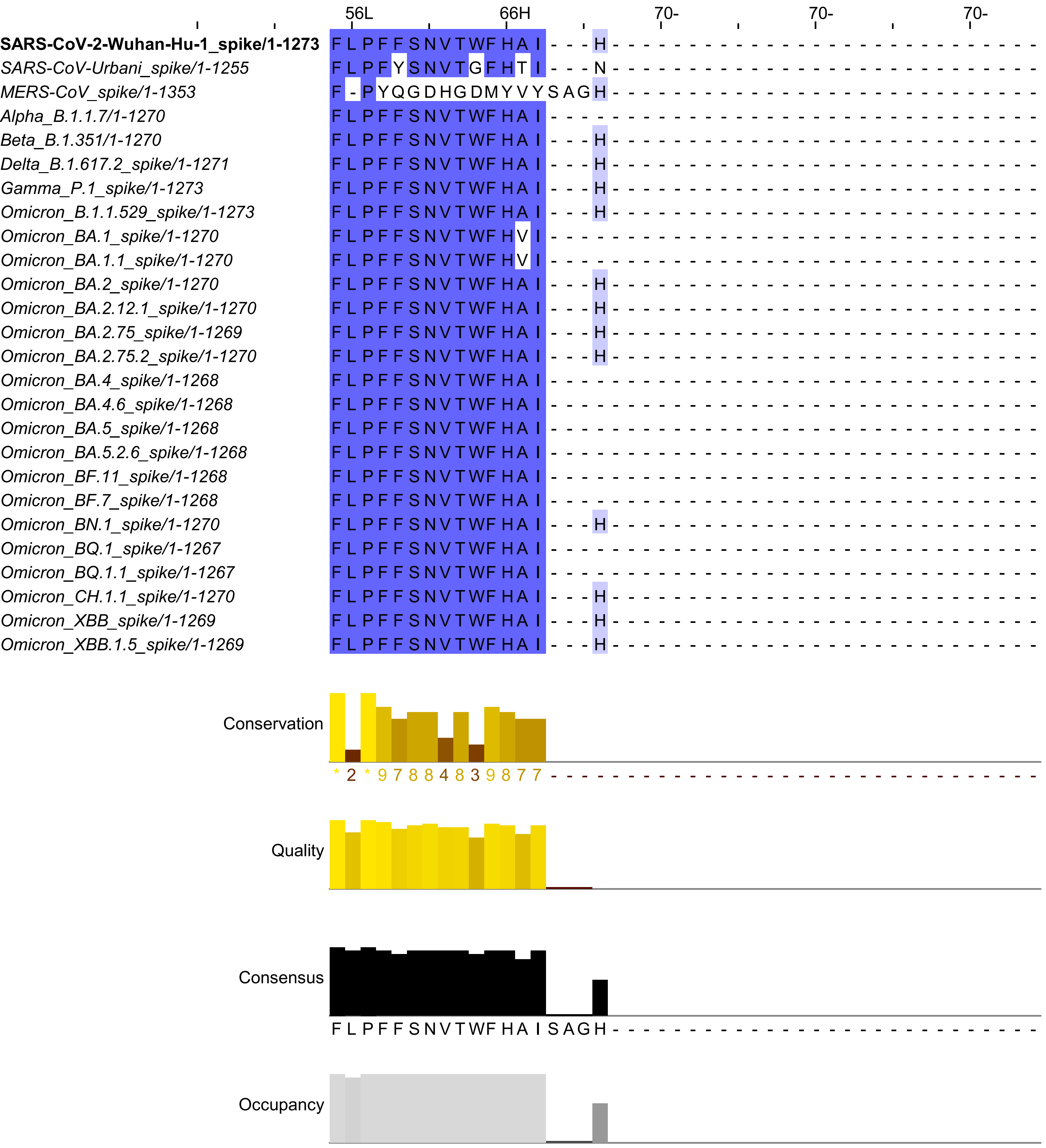
56L
66H
70-
70-
70-
SARS-CoV-2-Wuhan-Hu-1_spike/1-1273 FLPFFSNVTWFHAI---H
SARS-CoV-Urbani_spike/1-1255 FLPFYSNVTGFHTI---N
MERS-CoV_spike/1-1353 F-PYQGDHGDMYVYSAGH
Alpha_B.1.1.7/1-1270 FLPFFSNVTWFHAI
Beta_B.1.351/1-1270 FLPFFSNVTWFHAI---H
Delta_B.1.617.2_spike/1-1271 FLPFFSNVTWFHAI---H
Gamma_P.1_spike/1-1273 FLPFFSNVTWFHAI---H
Omicron_B.1.1.529_spike/1-1273 FLPFFSNVTWFHAI---H
Omicron_BA.1_spike/1-1270 FLPFFSNVTWFHVI
Omicron_BA.1.1_spike/1-1270 FLPFFSNVTWFHVI
Omicron_BA.2_spike/1-1270 FLPFFSNVTWFHAI---H
Omicron_BA.2.12.1_spike/1-1270 FLPFFSNVTWFHAI---H
Omicron_BA.2.75_spike/1-1269 FLPFFSNVTWFHAI---H
Omicron_BA.2.75.2_spike/1-1270 FLPFFSNVTWFHAI---H
Omicron_BA.4_spike/1-1268 FLPFFSNVTWFHAI
Omicron_BA.4.6_spike/1-1268 FLPFFSNVTWFHAI
Omicron_BA.5_spike/1-1268 FLPFFSNVTWFHAI
Omicron_BA.5.2.6_spike/1-1268 FLPFFSNVTWFHAI
Omicron_BF.11_spike/1-1268 FLPFFSNVTWFHAI
Omicron_BF.7_spike/1-1268 FLPFFSNVTWFHAI
Omicron_BN.1_spike/1-1270 FLPFFSNVTWFHAI---H
Omicron_BQ.1_spike/1-1267 FLPFFSNVTWFHAI
Omicron_BQ.1.1_spike/1-1267 FLPFFSNVTWFHAI
Omicron_CH.1.1_spike/1-1270 FLPFFSNVTWFHAI---H
Omicron_XBB_spike/1-1269 FLPFFSNVTWFHAI---H
Omicron_XBB.1.5_spike/1-1269 FLPFFSNVTWFHAI---H
Conservation
* 2 * 9 7 8 8 4 8 3 9 8 7 7
Quality
Consensus
FLPFFSNVTWFHAISAGH
Occupancy

```
                                          70-       70-       70-       70-
SARS-CoV-2-Wuhan-Hu-1_spike/1-1273  ----------------------------------------------
SARS-CoV-Urbani_spike/1-1255        ----------------------------------------------
MERS-CoV_spike/1-1353               ----------------------------------------------
Alpha_B.1.1.7/1-1270                ----------------------------------------------
Beta_B.1.351/1-1270                 ----------------------------------------------
Delta_B.1.617.2_spike/1-1271        ----------------------------------------------
Gamma_P.1_spike/1-1273              ----------------------------------------------
Omicron_B.1.1.529_spike/1-1273      ----------------------------------------------
Omicron_BA.1_spike/1-1270           ----------------------------------------------
Omicron_BA.1.1_spike/1-1270         ----------------------------------------------
Omicron_BA.2_spike/1-1270           ----------------------------------------------
Omicron_BA.2.12.1_spike/1-1270      ----------------------------------------------
Omicron_BA.2.75_spike/1-1269        ----------------------------------------------
Omicron_BA.2.75.2_spike/1-1270      ----------------------------------------------
Omicron_BA.4_spike/1-1268           ----------------------------------------------
Omicron_BA.4.6_spike/1-1268         ----------------------------------------------
Omicron_BA.5_spike/1-1268           ----------------------------------------------
Omicron_BA.5.2.6_spike/1-1268       ----------------------------------------------
Omicron_BF.11_spike/1-1268          ----------------------------------------------
Omicron_BF.7_spike/1-1268           ----------------------------------------------
Omicron_BN.1_spike/1-1270           ----------------------------------------------
Omicron_BQ.1_spike/1-1267           ----------------------------------------------
Omicron_BQ.1.1_spike/1-1267         ----------------------------------------------
Omicron_CH.1.1_spike/1-1270         ----------------------------------------------
Omicron_XBB_spike/1-1269            ----------------------------------------------
Omicron_XBB.1.5_spike/1-1269        ----------------------------------------------

                      Conservation
                                    ----------------------------------------------

                           Quality

                         Consensus
                                    ----------------------------------------------

                         Occupancy
```

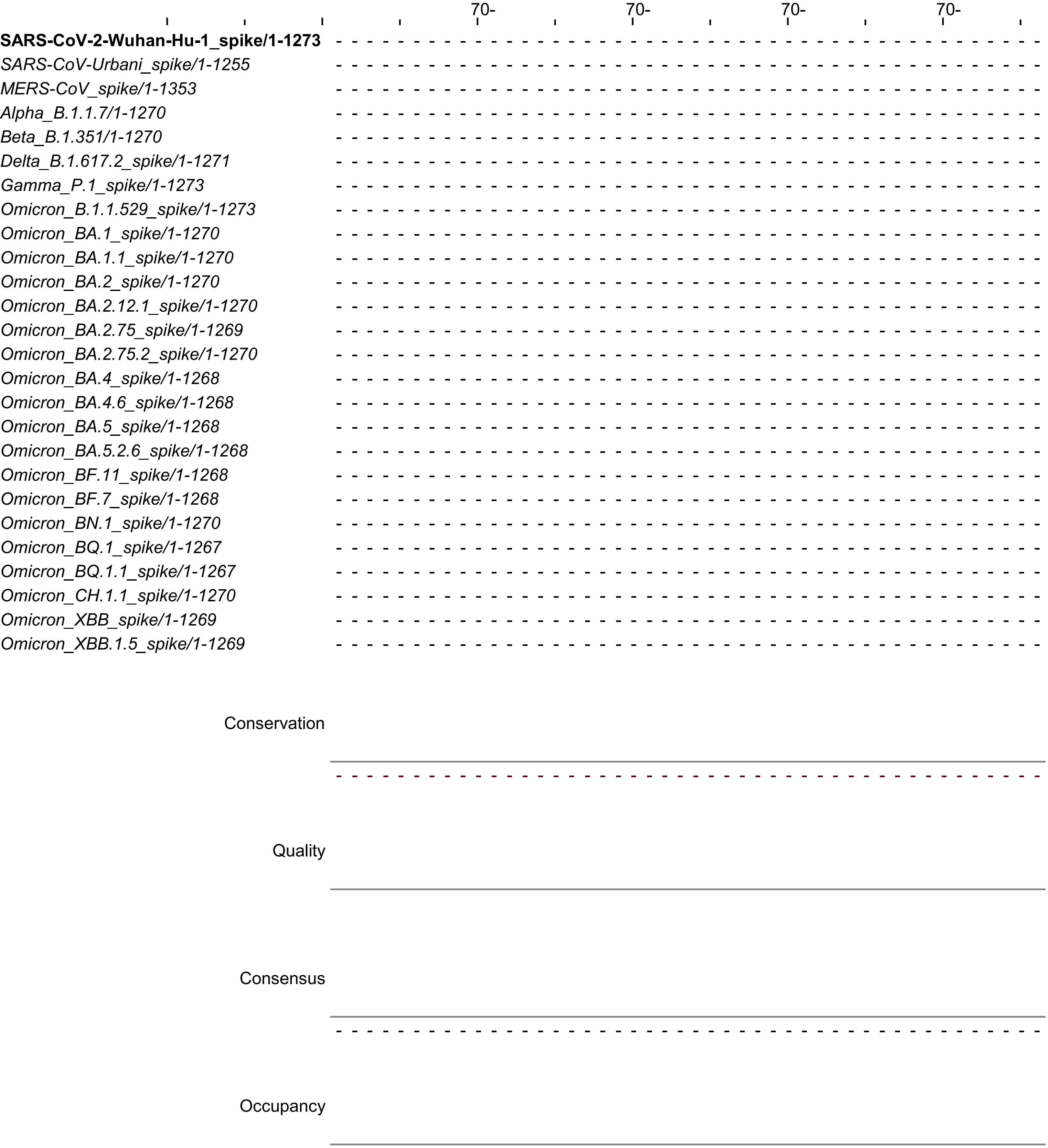
70-
70-
70-
70-
SARS-CoV-2-Wuhan-Hu-1_spike/1-1273
SARS-CoV-Urbani_spike/1-1255
MERS-CoV_spike/1-1353
Alpha_B.1.1.7/1-1270
Beta_B.1.351/1-1270
Delta_B.1.617.2_spike/1-1271
Gamma_P.1_spike/1-1273
Omicron_B.1.1.529_spike/1-1273
Omicron_BA.1_spike/1-1270
Omicron_BA.1.1_spike/1-1270
Omicron_BA.2_spike/1-1270
Omicron_BA.2.12.1_spike/1-1270
Omicron_BA.2.75_spike/1-1269
Omicron_BA.2.75.2_spike/1-1270
Omicron_BA.4_spike/1-1268
Omicron_BA.4.6_spike/1-1268
Omicron_BA.5_spike/1-1268
Omicron_BA.5.2.6_spike/1-1268
Omicron_BF.11_spike/1-1268
Omicron_BF.7_spike/1-1268
Omicron_BN.1_spike/1-1270
Omicron_BQ.1_spike/1-1267
Omicron_BQ.1.1_spike/1-1267
Omicron_CH.1.1_spike/1-1270
Omicron_XBB_spike/1-1269
Omicron_XBB.1.5_spike/1-1269
Conservation
Quality
Consensus
Occupancy

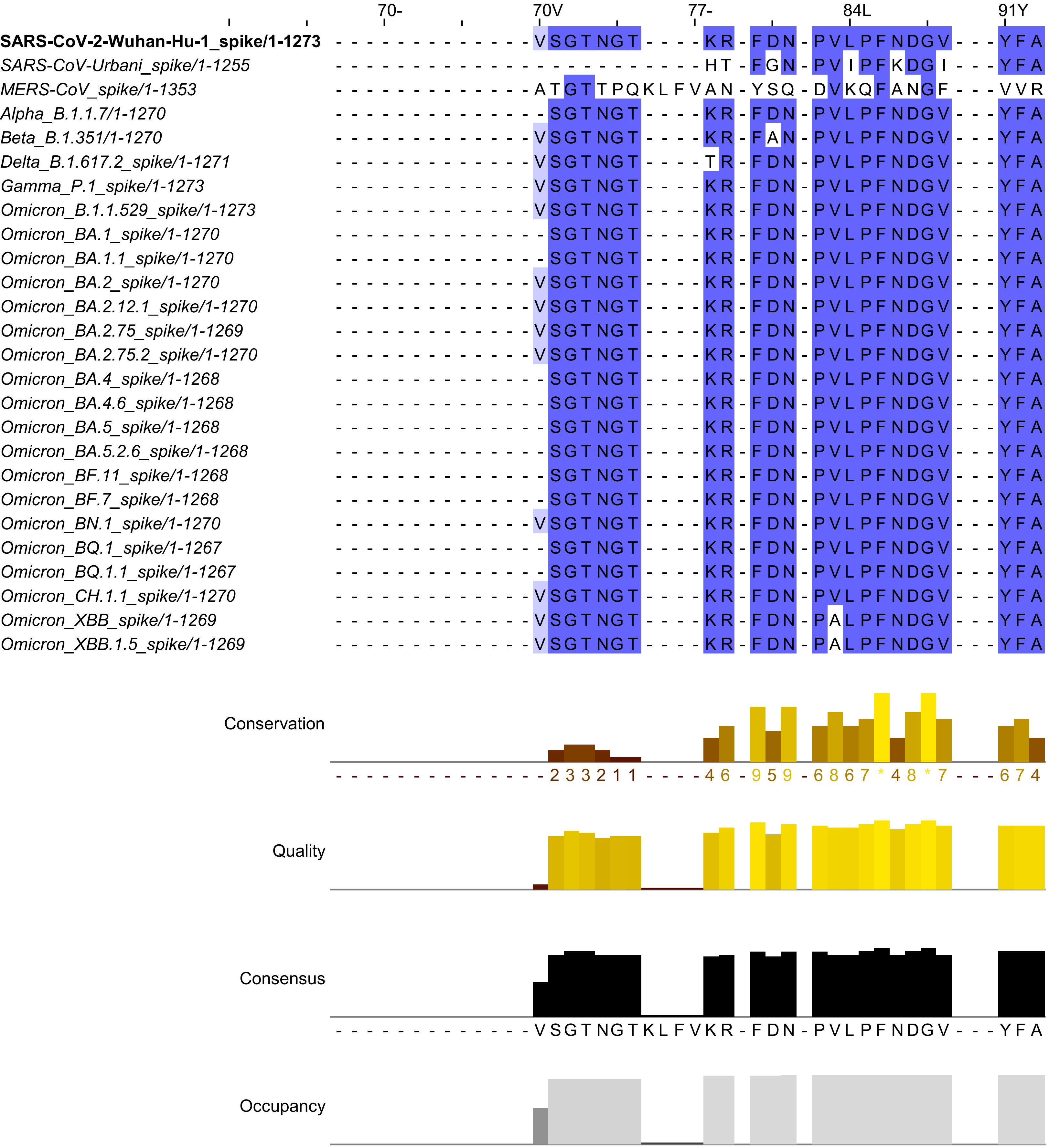
70-
70V
77-
84L
91Y
SARS-CoV-2-Wuhan-Hu-1_spike/1-1273
SARS-CoV-Urbani_spike/1-1255
MERS-CoV_spike/1-1353
Alpha_B.1.1.7/1-1270
Beta_B.1.351/1-1270
Delta_B.1.617.2_spike/1-1271
Gamma_P.1_spike/1-1273
Omicron_B.1.1.529_spike/1-1273
Omicron_BA.1_spike/1-1270
Omicron_BA.1.1_spike/1-1270
Omicron_BA.2_spike/1-1270
Omicron_BA.2.12.1_spike/1-1270
Omicron_BA.2.75_spike/1-1269
Omicron_BA.2.75.2_spike/1-1270
Omicron_BA.4_spike/1-1268
Omicron_BA.4.6_spike/1-1268
Omicron_BA.5_spike/1-1268
Omicron_BA.5.2.6_spike/1-1268
Omicron_BF.11_spike/1-1268
Omicron_BF.7_spike/1-1268
Omicron_BN.1_spike/1-1270
Omicron_BQ.1_spike/1-1267
Omicron_BQ.1.1_spike/1-1267
Omicron_CH.1.1_spike/1-1270
Omicron_XBB_spike/1-1269
Omicron_XBB.1.5_spike/1-1269
Conservation
Quality
Consensus
Occupancy

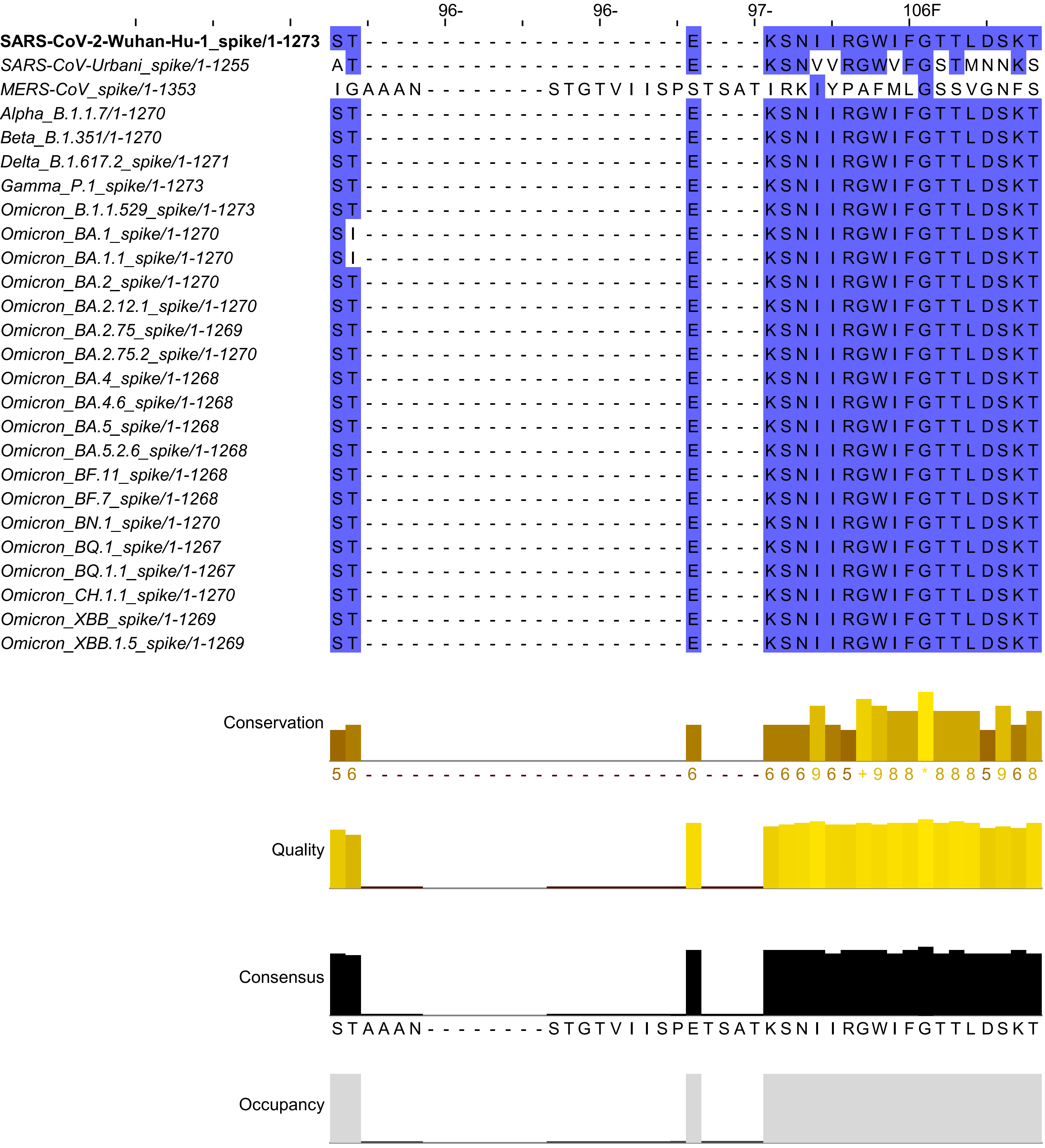
96-
96-
97-
106F
SARS-CoV-2-Wuhan-Hu-1_spike/1-1273
SARS-CoV-Urbani_spike/1-1255
MERS-CoV_spike/1-1353
Alpha_B.1.1.7/1-1270
Beta_B.1.351/1-1270
Delta_B.1.617.2_spike/1-1271
Gamma_P.1_spike/1-1273
Omicron_B.1.1.529_spike/1-1273
Omicron_BA.1_spike/1-1270
Omicron_BA.1.1_spike/1-1270
Omicron_BA.2_spike/1-1270
Omicron_BA.2.12.1_spike/1-1270
Omicron_BA.2.75_spike/1-1269
Omicron_BA.2.75.2_spike/1-1270
Omicron_BA.4_spike/1-1268
Omicron_BA.4.6_spike/1-1268
Omicron_BA.5_spike/1-1268
Omicron_BA.5.2.6_spike/1-1268
Omicron_BF.11_spike/1-1268
Omicron_BF.7_spike/1-1268
Omicron_BN.1_spike/1-1270
Omicron_BQ.1_spike/1-1267
Omicron_BQ.1.1_spike/1-1267
Omicron_CH.1.1_spike/1-1270
Omicron_XBB_spike/1-1269
Omicron_XBB.1.5_spike/1-1269
Conservation
Quality
Consensus
STAAAN--------STGTVIISPETSATKSNIIRGWIFGTTLDSKT
Occupancy

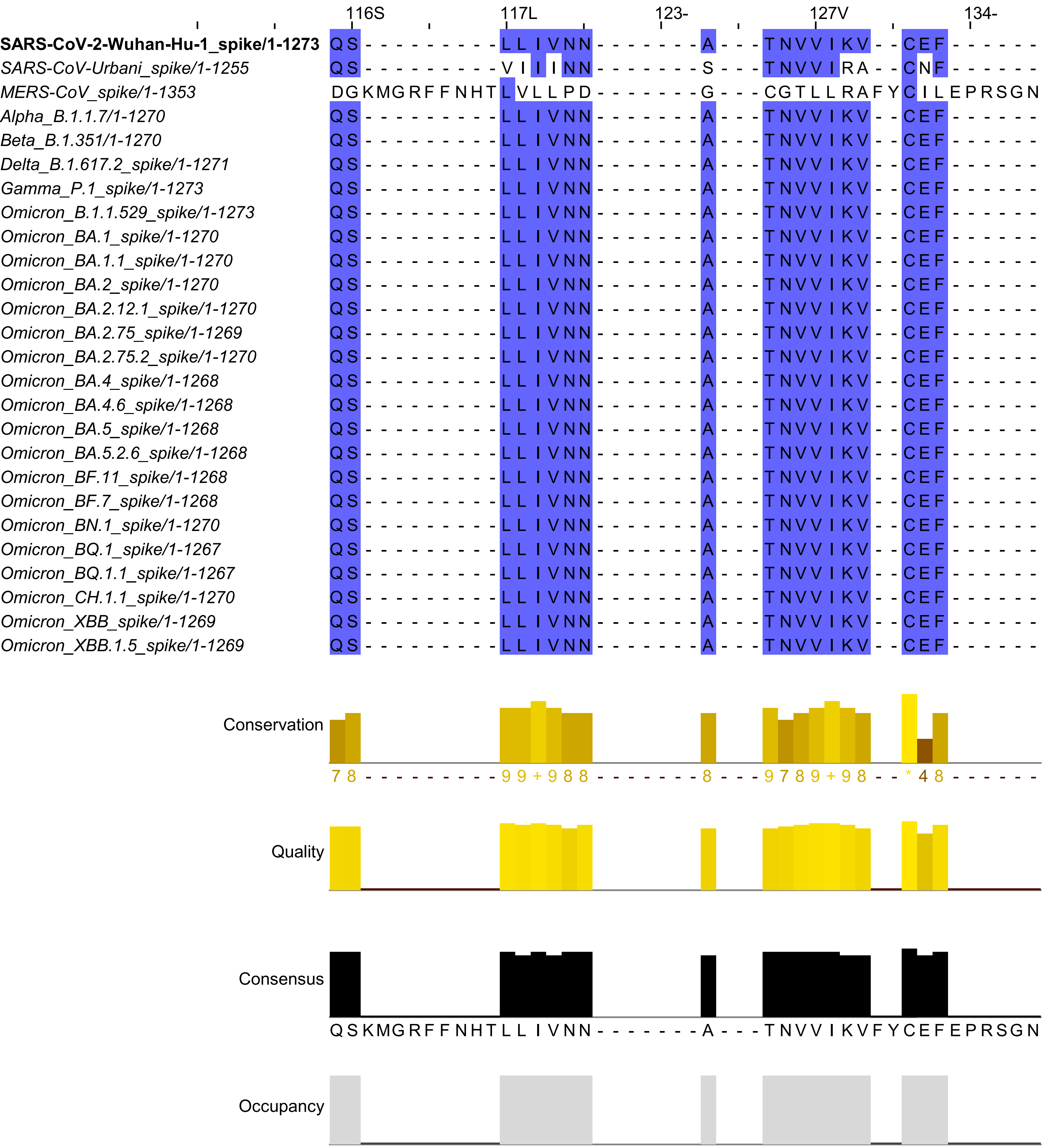
116S
117L
123-
127V
134-
SARS-CoV-2-Wuhan-Hu-1_spike/1-1273 QS---------LLIVNN-------A---TNVVIKV--CEF------
SARS-CoV-Urbani_spike/1-1255 QS---------VIIINN-------S---TNVVIRA--CNF------
MERS-CoV_spike/1-1353 DGKMGRFFNHTLVLLPD-------G---CGTLLRAFYCILEPRSGN
Alpha_B.1.1.7/1-1270 QS---------LLIVNN-------A---TNVVIKV--CEF------
Beta_B.1.351/1-1270 QS---------LLIVNN-------A---TNVVIKV--CEF------
Delta_B.1.617.2_spike/1-1271 QS---------LLIVNN-------A---TNVVIKV--CEF------
Gamma_P.1_spike/1-1273 QS---------LLIVNN-------A---TNVVIKV--CEF------
Omicron_B.1.1.529_spike/1-1273 QS---------LLIVNN-------A---TNVVIKV--CEF------
Omicron_BA.1_spike/1-1270 QS---------LLIVNN-------A---TNVVIKV--CEF------
Omicron_BA.1.1_spike/1-1270 QS---------LLIVNN-------A---TNVVIKV--CEF------
Omicron_BA.2_spike/1-1270 QS---------LLIVNN-------A---TNVVIKV--CEF------
Omicron_BA.2.12.1_spike/1-1270 QS---------LLIVNN-------A---TNVVIKV--CEF------
Omicron_BA.2.75_spike/1-1269 QS---------LLIVNN-------A---TNVVIKV--CEF------
Omicron_BA.2.75.2_spike/1-1270 QS---------LLIVNN-------A---TNVVIKV--CEF------
Omicron_BA.4_spike/1-1268 QS---------LLIVNN-------A---TNVVIKV--CEF------
Omicron_BA.4.6_spike/1-1268 QS---------LLIVNN-------A---TNVVIKV--CEF------
Omicron_BA.5_spike/1-1268 QS---------LLIVNN-------A---TNVVIKV--CEF------
Omicron_BA.5.2.6_spike/1-1268 QS---------LLIVNN-------A---TNVVIKV--CEF------
Omicron_BF.11_spike/1-1268 QS---------LLIVNN-------A---TNVVIKV--CEF------
Omicron_BF.7_spike/1-1268 QS---------LLIVNN-------A---TNVVIKV--CEF------
Omicron_BN.1_spike/1-1270 QS---------LLIVNN-------A---TNVVIKV--CEF------
Omicron_BQ.1_spike/1-1267 QS---------LLIVNN-------A---TNVVIKV--CEF------
Omicron_BQ.1.1_spike/1-1267 QS---------LLIVNN-------A---TNVVIKV--CEF------
Omicron_CH.1.1_spike/1-1270 QS---------LLIVNN-------A---TNVVIKV--CEF------
Omicron_XBB_spike/1-1269 QS---------LLIVNN-------A---TNVVIKV--CEF------
Omicron_XBB.1.5_spike/1-1269 QS---------LLIVNN-------A---TNVVIKV--CEF------
Conservation
78---------99+988-------8---9789+98--*48------
Quality
Consensus
QSKMGRFFNHTLLIVNN-------A---TNVVIKVFYCEFEPRSGN
Occupancy

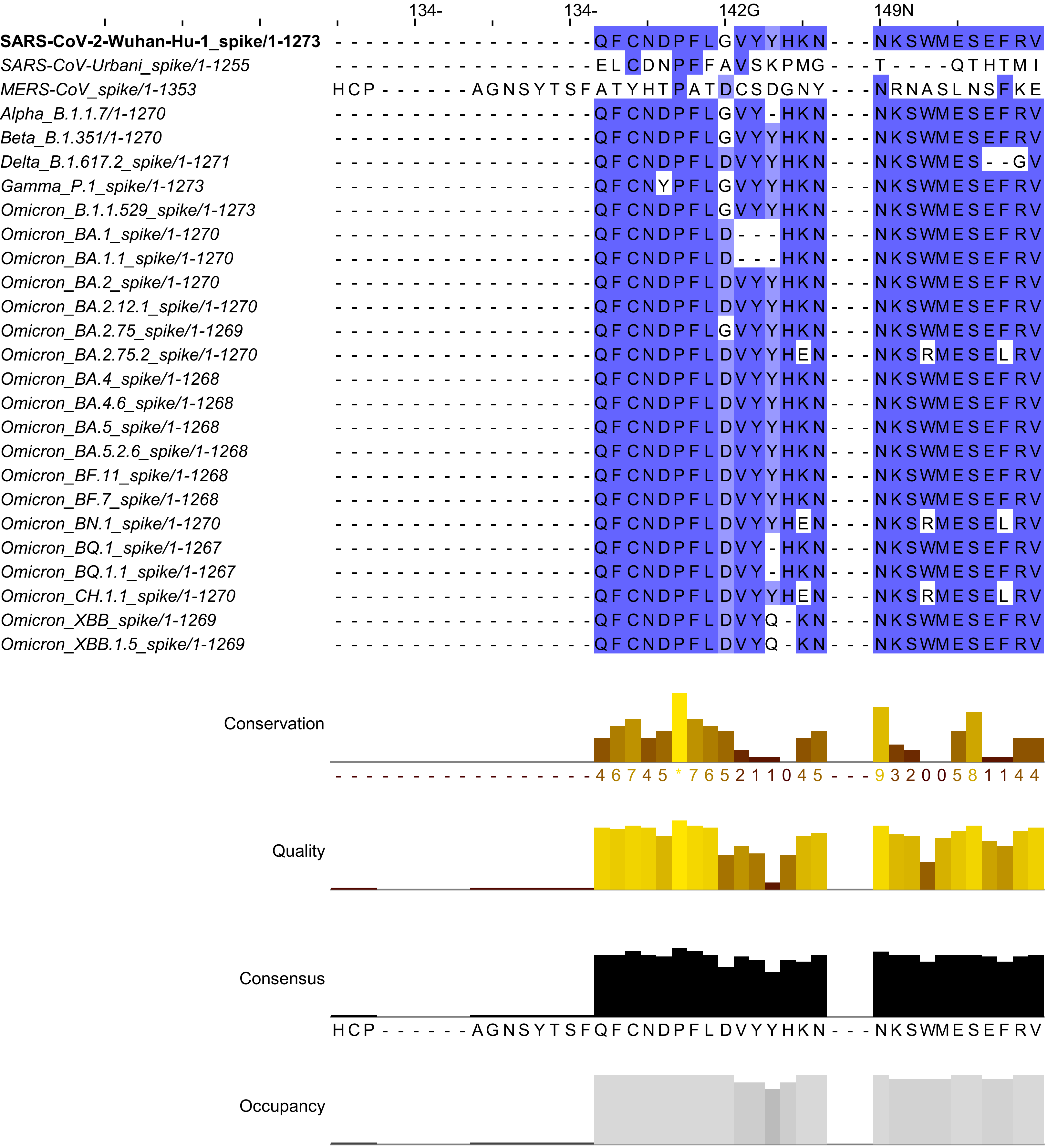
134-
134-
142G
149N
SARS-CoV-2-Wuhan-Hu-1_spike/1-1273 -----------------QFCNDPFLGVYYHKN---NKSWMESEFRV
SARS-CoV-Urbani_spike/1-1255 -----------------ELCDNPFFAVSKPMG---T----QTHTMI
MERS-CoV_spike/1-1353 HCP------AGNSYTSFATYHTPATDCSDGNY---NRNASLNSFKE
Alpha_B.1.1.7/1-1270 -----------------QFCNDPFLGVY-HKN---NKSWMESEFRV
Beta_B.1.351/1-1270 -----------------QFCNDPFLGVYYHKN---NKSWMESEFRV
Delta_B.1.617.2_spike/1-1271 -----------------QFCNDPFLDVYYHKN---NKSWMES--GV
Gamma_P.1_spike/1-1273 -----------------QFCNYPFLGVYYHKN---NKSWMESEFRV
Omicron_B.1.1.529_spike/1-1273 -----------------QFCNDPFLGVYYHKN---NKSWMESEFRV
Omicron_BA.1_spike/1-1270 -----------------QFCNDPFLD---HKN---NKSWMESEFRV
Omicron_BA.1.1_spike/1-1270 -----------------QFCNDPFLD---HKN---NKSWMESEFRV
Omicron_BA.2_spike/1-1270 -----------------QFCNDPFLDVYYHKN---NKSWMESEFRV
Omicron_BA.2.12.1_spike/1-1270 -----------------QFCNDPFLDVYYHKN---NKSWMESEFRV
Omicron_BA.2.75_spike/1-1269 -----------------QFCNDPFLGVYYHKN---NKSWMESEFRV
Omicron_BA.2.75.2_spike/1-1270 -----------------QFCNDPFLDVYYHEN---NKSRMESELRV
Omicron_BA.4_spike/1-1268 -----------------QFCNDPFLDVYYHKN---NKSWMESEFRV
Omicron_BA.4.6_spike/1-1268 -----------------QFCNDPFLDVYYHKN---NKSWMESEFRV
Omicron_BA.5_spike/1-1268 -----------------QFCNDPFLDVYYHKN---NKSWMESEFRV
Omicron_BA.5.2.6_spike/1-1268 -----------------QFCNDPFLDVYYHKN---NKSWMESEFRV
Omicron_BF.11_spike/1-1268 -----------------QFCNDPFLDVYYHKN---NKSWMESEFRV
Omicron_BF.7_spike/1-1268 -----------------QFCNDPFLDVYYHKN---NKSWMESEFRV
Omicron_BN.1_spike/1-1270 -----------------QFCNDPFLDVYYHEN---NKSRMESELRV
Omicron_BQ.1_spike/1-1267 -----------------QFCNDPFLDVY-HKN---NKSWMESEFRV
Omicron_BQ.1.1_spike/1-1267 -----------------QFCNDPFLDVY-HKN---NKSWMESEFRV
Omicron_CH.1.1_spike/1-1270 -----------------QFCNDPFLDVYYHEN---NKSRMESELRV
Omicron_XBB_spike/1-1269 -----------------QFCNDPFLDVYQ-KN---NKSWMESEFRV
Omicron_XBB.1.5_spike/1-1269 -----------------QFCNDPFLDVYQ-KN---NKSWMESEFRV
Conservation
-----------------46745*7652110 45---93200581144
Quality
Consensus
HCP------AGNSYTSFQFCNDPFLDVYYHKN---NKSWMESEFRV
Occupancy

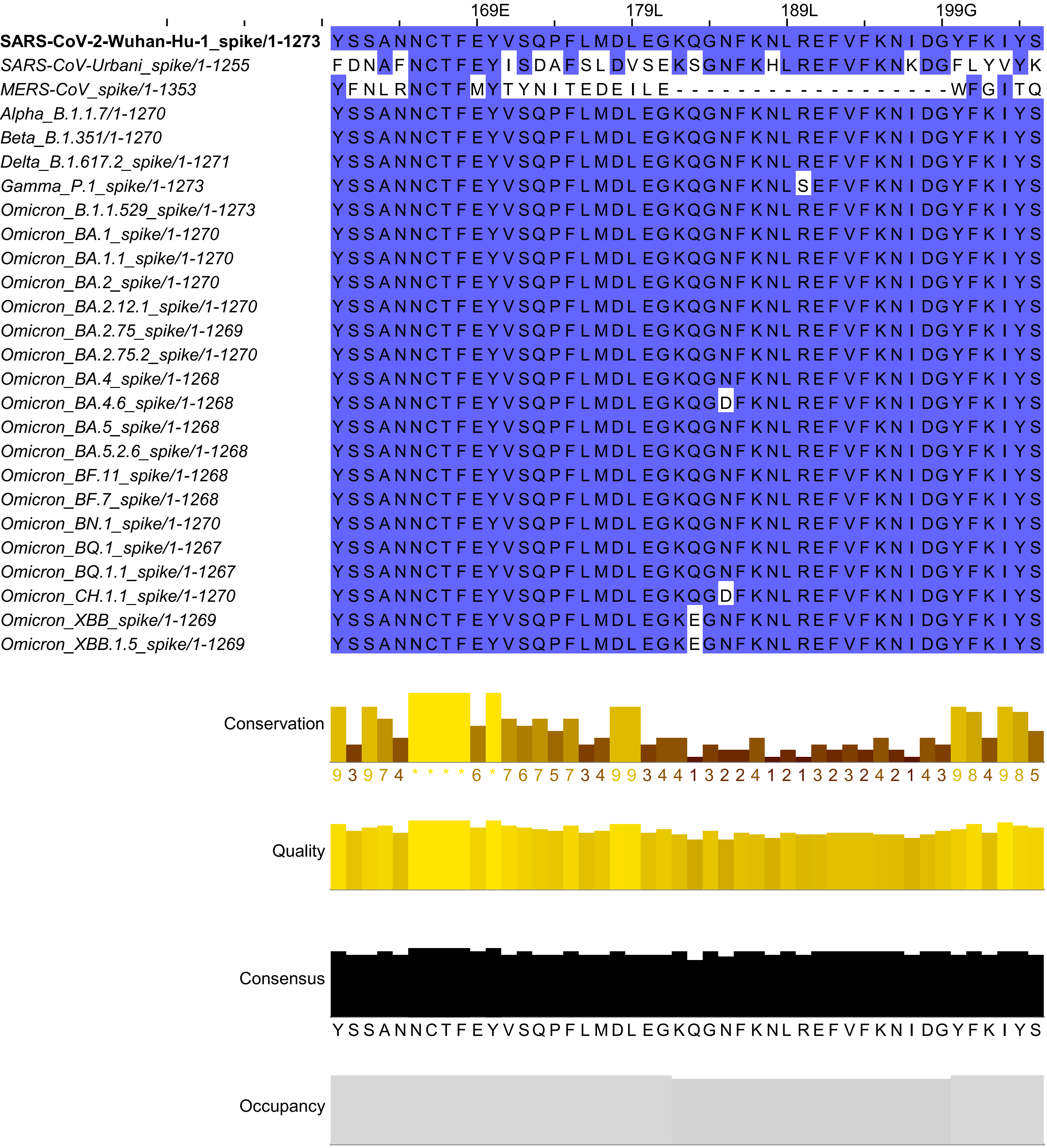

169E
179L
189L
199G
SARS-CoV-2-Wuhan-Hu-1_spike/1-1273 YSSANNCTFEYVSQPFLMDLEGKQGNFKNLREFVFKNIDGYFKIYS
SARS-CoV-Urbani_spike/1-1255 FDNAFNCTFEYISDAFSLDVSEKSGNFKHLREFVFKNKDGFLYVYK
MERS-CoV_spike/1-1353 YFNLRNCTFMYTYNITEDEILE------------------WFGITQ
Alpha_B.1.1.7/1-1270 YSSANNCTFEYVSQPFLMDLEGKQGNFKNLREFVFKNIDGYFKIYS
Beta_B.1.351/1-1270 YSSANNCTFEYVSQPFLMDLEGKQGNFKNLREFVFKNIDGYFKIYS
Delta_B.1.617.2_spike/1-1271 YSSANNCTFEYVSQPFLMDLEGKQGNFKNLREFVFKNIDGYFKIYS
Gamma_P.1_spike/1-1273 YSSANNCTFEYVSQPFLMDLEGKQGNFKNLSEFVFKNIDGYFKIYS
Omicron_B.1.1.529_spike/1-1273 YSSANNCTFEYVSQPFLMDLEGKQGNFKNLREFVFKNIDGYFKIYS
Omicron_BA.1_spike/1-1270 YSSANNCTFEYVSQPFLMDLEGKQGNFKNLREFVFKNIDGYFKIYS
Omicron_BA.1.1_spike/1-1270 YSSANNCTFEYVSQPFLMDLEGKQGNFKNLREFVFKNIDGYFKIYS
Omicron_BA.2_spike/1-1270 YSSANNCTFEYVSQPFLMDLEGKQGNFKNLREFVFKNIDGYFKIYS
Omicron_BA.2.12.1_spike/1-1270 YSSANNCTFEYVSQPFLMDLEGKQGNFKNLREFVFKNIDGYFKIYS
Omicron_BA.2.75_spike/1-1269 YSSANNCTFEYVSQPFLMDLEGKQGNFKNLREFVFKNIDGYFKIYS
Omicron_BA.2.75.2_spike/1-1270 YSSANNCTFEYVSQPFLMDLEGKQGNFKNLREFVFKNIDGYFKIYS
Omicron_BA.4_spike/1-1268 YSSANNCTFEYVSQPFLMDLEGKQGNFKNLREFVFKNIDGYFKIYS
Omicron_BA.4.6_spike/1-1268 YSSANNCTFEYVSQPFLMDLEGKQGDFKNLREFVFKNIDGYFKIYS
Omicron_BA.5_spike/1-1268 YSSANNCTFEYVSQPFLMDLEGKQGNFKNLREFVFKNIDGYFKIYS
Omicron_BA.5.2.6_spike/1-1268 YSSANNCTFEYVSQPFLMDLEGKQGNFKNLREFVFKNIDGYFKIYS
Omicron_BF.11_spike/1-1268 YSSANNCTFEYVSQPFLMDLEGKQGNFKNLREFVFKNIDGYFKIYS
Omicron_BF.7_spike/1-1268 YSSANNCTFEYVSQPFLMDLEGKQGNFKNLREFVFKNIDGYFKIYS
Omicron_BN.1_spike/1-1270 YSSANNCTFEYVSQPFLMDLEGKQGNFKNLREFVFKNIDGYFKIYS
Omicron_BQ.1_spike/1-1267 YSSANNCTFEYVSQPFLMDLEGKQGNFKNLREFVFKNIDGYFKIYS
Omicron_BQ.1.1_spike/1-1267 YSSANNCTFEYVSQPFLMDLEGKQGNFKNLREFVFKNIDGYFKIYS
Omicron_CH.1.1_spike/1-1270 YSSANNCTFEYVSQPFLMDLEGKQGDFKNLREFVFKNIDGYFKIYS
Omicron_XBB_spike/1-1269 YSSANNCTFEYVSQPFLMDLEGKEGNFKNLREFVFKNIDGYFKIYS
Omicron_XBB.1.5_spike/1-1269 YSSANNCTFEYVSQPFLMDLEGKEGNFKNLREFVFKNIDGYFKIYS
Conservation
9 3 9 7 4 * * * * 6 * 7 6 7 5 7 3 4 9 9 3 4 4 1 3 2 2 4 1 2 1 3 2 3 2 4 2 1 4 3 9 8 4 9 8 5
Quality
Consensus
YSSANNCTFEYVSQPFLMDLEGKQGNFKNLREFVFKNIDGYFKIYS
Occupancy

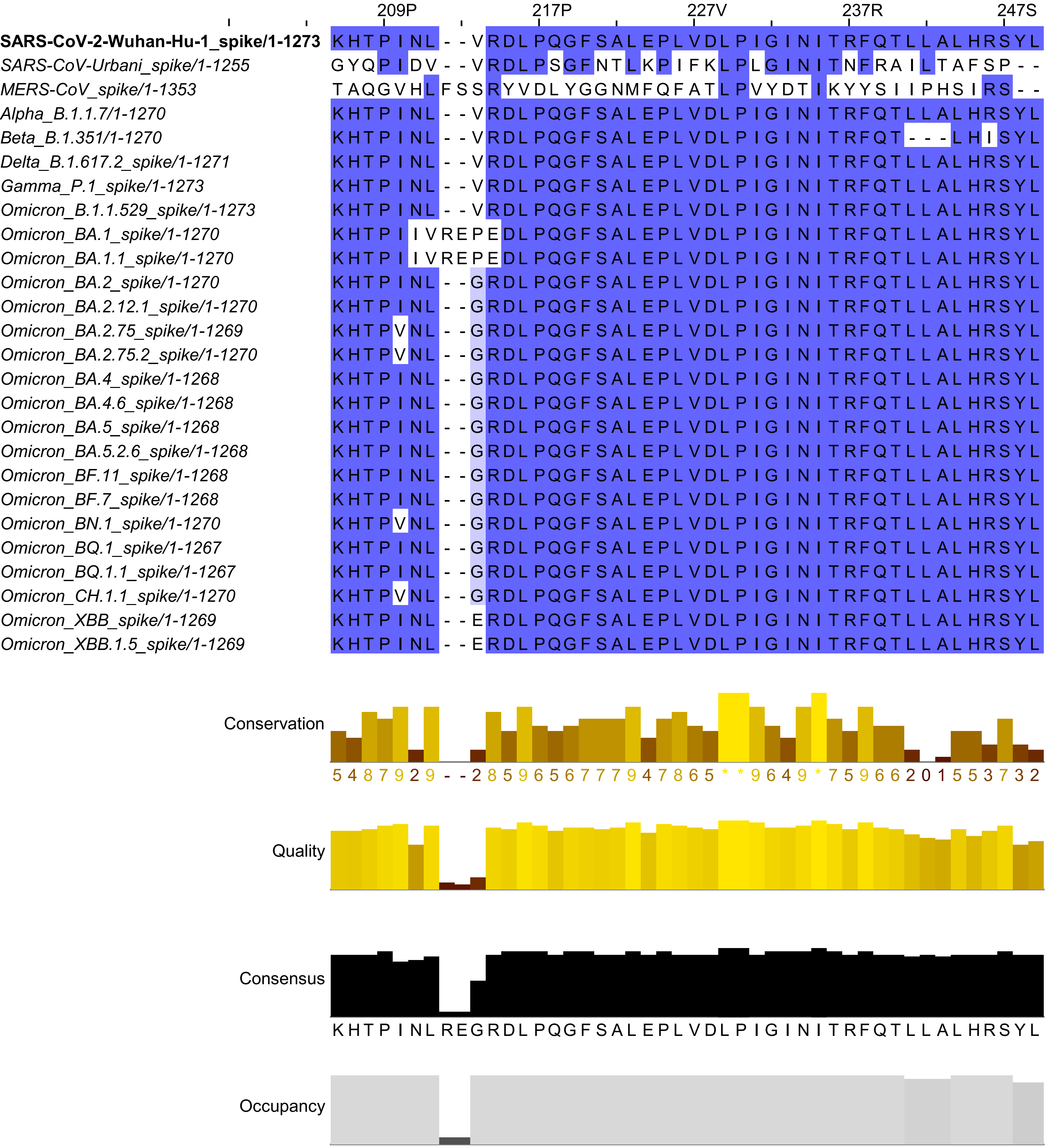
209P
217P
227V
237R
247S
SARS-CoV-2-Wuhan-Hu-1_spike/1-1273 KHTPINL--VRDLPQGFSALEPLVDLPIGINITRFQTLLALHRSYL
SARS-CoV-Urbani_spike/1-1255 GYQPIDV--VRDLPSGFNTLKPIFKLPLGINITNFRAILTAFSP--
MERS-CoV_spike/1-1353 TAQGVHLFSSRYVDLYGGNMFQFATLPVYDTIKYYSIIPHSIRS--
Alpha_B.1.1.7/1-1270 KHTPINL--VRDLPQGFSALEPLVDLPIGINITRFQTLLALHRSYL
Beta_B.1.351/1-1270 KHTPINL--VRDLPQGFSALEPLVDLPIGINITRFQT---LHISYL
Delta_B.1.617.2_spike/1-1271 KHTPINL--VRDLPQGFSALEPLVDLPIGINITRFQTLLALHRSYL
Gamma_P.1_spike/1-1273 KHTPINL--VRDLPQGFSALEPLVDLPIGINITRFQTLLALHRSYL
Omicron_B.1.1.529_spike/1-1273 KHTPINL--VRDLPQGFSALEPLVDLPIGINITRFQTLLALHRSYL
Omicron_BA.1_spike/1-1270 KHTPIIVREPEDLPQGFSALEPLVDLPIGINITRFQTLLALHRSYL
Omicron_BA.1.1_spike/1-1270 KHTPIIVREPEDLPQGFSALEPLVDLPIGINITRFQTLLALHRSYL
Omicron_BA.2_spike/1-1270 KHTPINL--GRDLPQGFSALEPLVDLPIGINITRFQTLLALHRSYL
Omicron_BA.2.12.1_spike/1-1270 KHTPINL--GRDLPQGFSALEPLVDLPIGINITRFQTLLALHRSYL
Omicron_BA.2.75_spike/1-1269 KHTPVNL--GRDLPQGFSALEPLVDLPIGINITRFQTLLALHRSYL
Omicron_BA.2.75.2_spike/1-1270 KHTPVNL--GRDLPQGFSALEPLVDLPIGINITRFQTLLALHRSYL
Omicron_BA.4_spike/1-1268 KHTPINL--GRDLPQGFSALEPLVDLPIGINITRFQTLLALHRSYL
Omicron_BA.4.6_spike/1-1268 KHTPINL--GRDLPQGFSALEPLVDLPIGINITRFQTLLALHRSYL
Omicron_BA.5_spike/1-1268 KHTPINL--GRDLPQGFSALEPLVDLPIGINITRFQTLLALHRSYL
Omicron_BA.5.2.6_spike/1-1268 KHTPINL--GRDLPQGFSALEPLVDLPIGINITRFQTLLALHRSYL
Omicron_BF.11_spike/1-1268 KHTPINL--GRDLPQGFSALEPLVDLPIGINITRFQTLLALHRSYL
Omicron_BF.7_spike/1-1268 KHTPINL--GRDLPQGFSALEPLVDLPIGINITRFQTLLALHRSYL
Omicron_BN.1_spike/1-1270 KHTPVNL--GRDLPQGFSALEPLVDLPIGINITRFQTLLALHRSYL
Omicron_BQ.1_spike/1-1267 KHTPINL--GRDLPQGFSALEPLVDLPIGINITRFQTLLALHRSYL
Omicron_BQ.1.1_spike/1-1267 KHTPINL--GRDLPQGFSALEPLVDLPIGINITRFQTLLALHRSYL
Omicron_CH.1.1_spike/1-1270 KHTPVNL--GRDLPQGFSALEPLVDLPIGINITRFQTLLALHRSYL
Omicron_XBB_spike/1-1269 KHTPINL--ERDLPQGFSALEPLVDLPIGINITRFQTLLALHRSYL
Omicron_XBB.1.5_spike/1-1269 KHTPINL--ERDLPQGFSALEPLVDLPIGINITRFQTLLALHRSYL
Conservation
5487929--285965677779478 65**9649*7596620155373 2
Quality
Consensus
KHTPINLREGRDLPQGFSALEPLVDLPIGINITRFQTLLALHRSYL
Occupancy

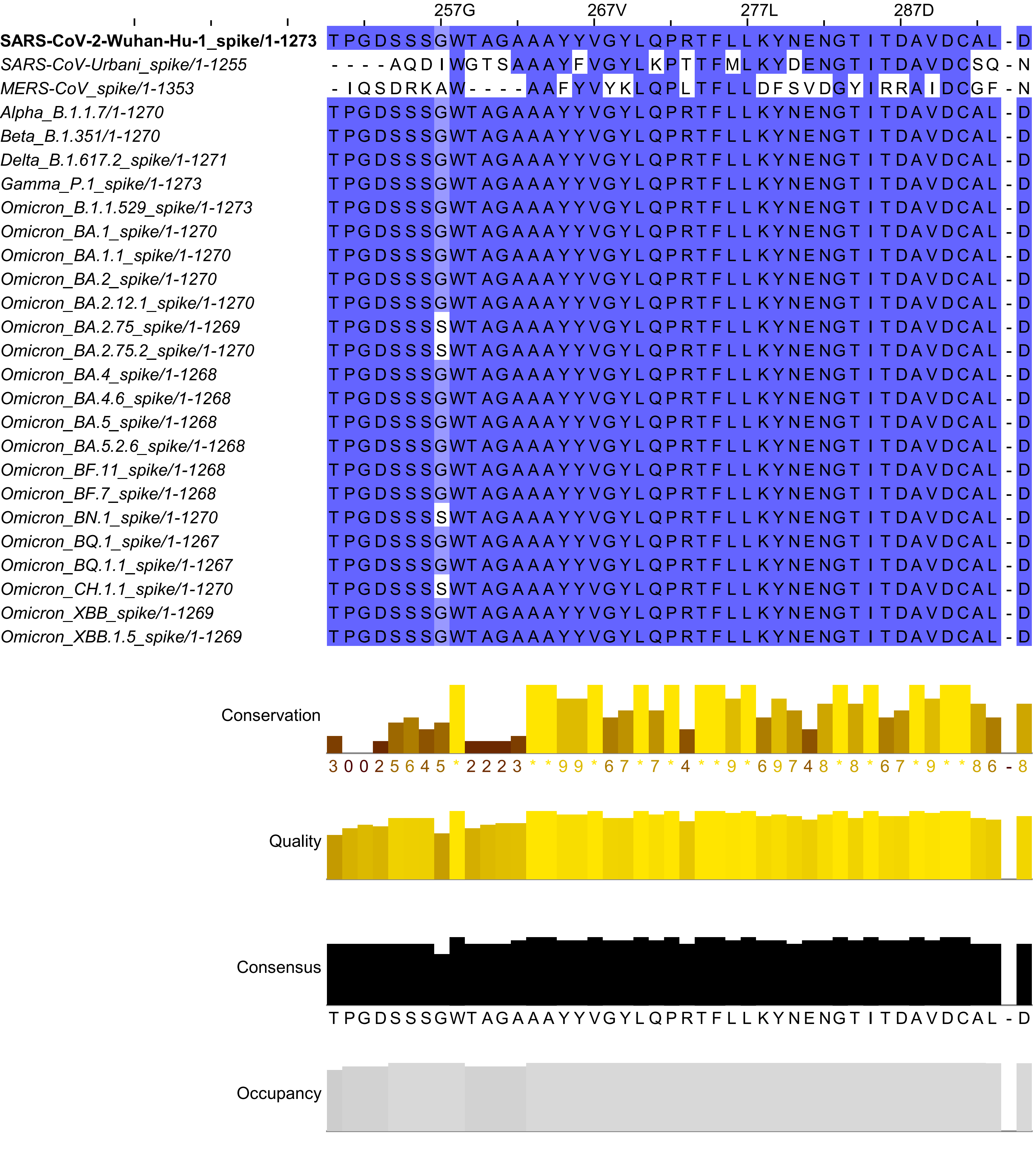

257G
267V
277L
287D
SARS-CoV-2-Wuhan-Hu-1_spike/1-1273 TPGDSSSGWTAGAAAYYVGYLQPRTFLLKYNENGTITDAVDCAL-D
SARS-CoV-Urbani_spike/1-1255 ----AQDIWGTSAAAYFVGYLKPTTFMLKYDENGTITDAVDCSQ-N
MERS-CoV_spike/1-1353 -IQSDRKAW----AAFYVYKLQPLTFLLDFSVDGYIRRAIDCGF-N
Alpha_B.1.1.7/1-1270 TPGDSSSGWTAGAAAYYVGYLQPRTFLLKYNENGTITDAVDCAL-D
Beta_B.1.351/1-1270 TPGDSSSGWTAGAAAYYVGYLQPRTFLLKYNENGTITDAVDCAL-D
Delta_B.1.617.2_spike/1-1271 TPGDSSSGWTAGAAAYYVGYLQPRTFLLKYNENGTITDAVDCAL-D
Gamma_P.1_spike/1-1273 TPGDSSSGWTAGAAAYYVGYLQPRTFLLKYNENGTITDAVDCAL-D
Omicron_B.1.1.529_spike/1-1273 TPGDSSSGWTAGAAAYYVGYLQPRTFLLKYNENGTITDAVDCAL-D
Omicron_BA.1_spike/1-1270 TPGDSSSGWTAGAAAYYVGYLQPRTFLLKYNENGTITDAVDCAL-D
Omicron_BA.1.1_spike/1-1270 TPGDSSSGWTAGAAAYYVGYLQPRTFLLKYNENGTITDAVDCAL-D
Omicron_BA.2_spike/1-1270 TPGDSSSGWTAGAAAYYVGYLQPRTFLLKYNENGTITDAVDCAL-D
Omicron_BA.2.12.1_spike/1-1270 TPGDSSSGWTAGAAAYYVGYLQPRTFLLKYNENGTITDAVDCAL-D
Omicron_BA.2.75_spike/1-1269 TPGDSSSSWTAGAAAYYVGYLQPRTFLLKYNENGTITDAVDCAL-D
Omicron_BA.2.75.2_spike/1-1270 TPGDSSSSWTAGAAAYYVGYLQPRTFLLKYNENGTITDAVDCAL-D
Omicron_BA.4_spike/1-1268 TPGDSSSGWTAGAAAYYVGYLQPRTFLLKYNENGTITDAVDCAL-D
Omicron_BA.4.6_spike/1-1268 TPGDSSSGWTAGAAAYYVGYLQPRTFLLKYNENGTITDAVDCAL-D
Omicron_BA.5_spike/1-1268 TPGDSSSGWTAGAAAYYVGYLQPRTFLLKYNENGTITDAVDCAL-D
Omicron_BA.5.2.6_spike/1-1268 TPGDSSSGWTAGAAAYYVGYLQPRTFLLKYNENGTITDAVDCAL-D
Omicron_BF.11_spike/1-1268 TPGDSSSGWTAGAAAYYVGYLQPRTFLLKYNENGTITDAVDCAL-D
Omicron_BF.7_spike/1-1268 TPGDSSSGWTAGAAAYYVGYLQPRTFLLKYNENGTITDAVDCAL-D
Omicron_BN.1_spike/1-1270 TPGDSSSSWTAGAAAYYVGYLQPRTFLLKYNENGTITDAVDCAL-D
Omicron_BQ.1_spike/1-1267 TPGDSSSGWTAGAAAYYVGYLQPRTFLLKYNENGTITDAVDCAL-D
Omicron_BQ.1.1_spike/1-1267 TPGDSSSGWTAGAAAYYVGYLQPRTFLLKYNENGTITDAVDCAL-D
Omicron_CH.1.1_spike/1-1270 TPGDSSSSWTAGAAAYYVGYLQPRTFLLKYNENGTITDAVDCAL-D
Omicron_XBB_spike/1-1269 TPGDSSSGWTAGAAAYYVGYLQPRTFLLKYNENGTITDAVDCAL-D
Omicron_XBB.1.5_spike/1-1269 TPGDSSSGWTAGAAAYYVGYLQPRTFLLKYNENGTITDAVDCAL-D
Conservation
30025645*2223**99*67*7*4**9*69748*8*67*9**86-8
Quality
Consensus
TPGDSSSGWTAGAAAYYVGYLQPRTFLLKYNENGTITDAVDCAL-D
Occupancy

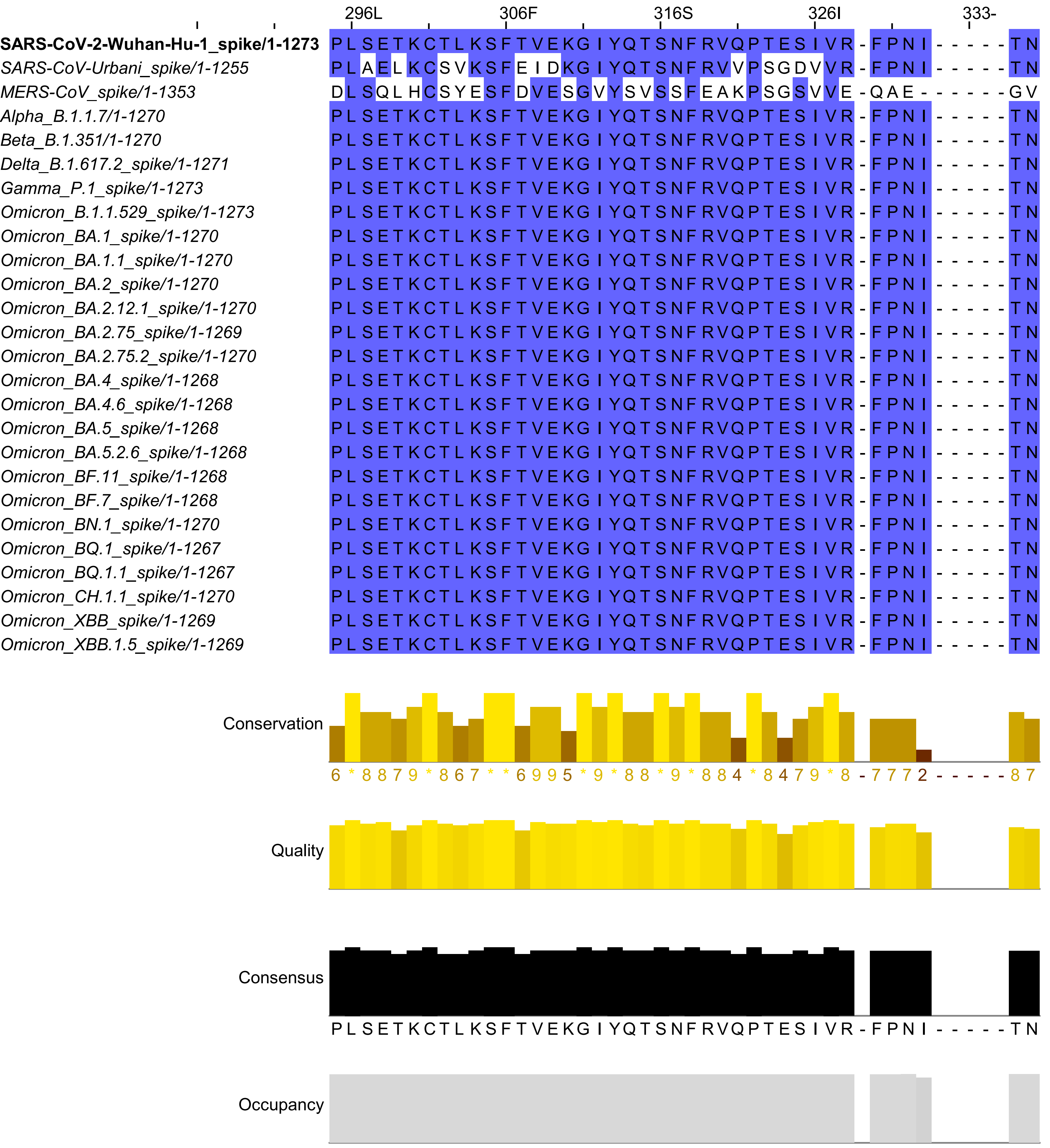

296L
306F
316S
326I
333-
SARS-CoV-2-Wuhan-Hu-1_spike/1-1273 PLSETKCTLKSFTVEKGIYQTSNFRVQPTESIVR-FPNI-----TN
SARS-CoV-Urbani_spike/1-1255 PLAELKCSVKSFEIDKGIYQTSNFRVVPSGDVVR-FPNI-----TN
MERS-CoV_spike/1-1353 DLSQLHCSYESFDVESGVYSVSSFEAKPSGSVVE-QAE------GV
Alpha_B.1.1.7/1-1270 PLSETKCTLKSFTVEKGIYQTSNFRVQPTESIVR-FPNI-----TN
Beta_B.1.351/1-1270 PLSETKCTLKSFTVEKGIYQTSNFRVQPTESIVR-FPNI-----TN
Delta_B.1.617.2_spike/1-1271 PLSETKCTLKSFTVEKGIYQTSNFRVQPTESIVR-FPNI-----TN
Gamma_P.1_spike/1-1273 PLSETKCTLKSFTVEKGIYQTSNFRVQPTESIVR-FPNI-----TN
Omicron_B.1.1.529_spike/1-1273 PLSETKCTLKSFTVEKGIYQTSNFRVQPTESIVR-FPNI-----TN
Omicron_BA.1_spike/1-1270 PLSETKCTLKSFTVEKGIYQTSNFRVQPTESIVR-FPNI-----TN
Omicron_BA.1.1_spike/1-1270 PLSETKCTLKSFTVEKGIYQTSNFRVQPTESIVR-FPNI-----TN
Omicron_BA.2_spike/1-1270 PLSETKCTLKSFTVEKGIYQTSNFRVQPTESIVR-FPNI-----TN
Omicron_BA.2.12.1_spike/1-1270 PLSETKCTLKSFTVEKGIYQTSNFRVQPTESIVR-FPNI-----TN
Omicron_BA.2.75_spike/1-1269 PLSETKCTLKSFTVEKGIYQTSNFRVQPTESIVR-FPNI-----TN
Omicron_BA.2.75.2_spike/1-1270 PLSETKCTLKSFTVEKGIYQTSNFRVQPTESIVR-FPNI-----TN
Omicron_BA.4_spike/1-1268 PLSETKCTLKSFTVEKGIYQTSNFRVQPTESIVR-FPNI-----TN
Omicron_BA.4.6_spike/1-1268 PLSETKCTLKSFTVEKGIYQTSNFRVQPTESIVR-FPNI-----TN
Omicron_BA.5_spike/1-1268 PLSETKCTLKSFTVEKGIYQTSNFRVQPTESIVR-FPNI-----TN
Omicron_BA.5.2.6_spike/1-1268 PLSETKCTLKSFTVEKGIYQTSNFRVQPTESIVR-FPNI-----TN
Omicron_BF.11_spike/1-1268 PLSETKCTLKSFTVEKGIYQTSNFRVQPTESIVR-FPNI-----TN
Omicron_BF.7_spike/1-1268 PLSETKCTLKSFTVEKGIYQTSNFRVQPTESIVR-FPNI-----TN
Omicron_BN.1_spike/1-1270 PLSETKCTLKSFTVEKGIYQTSNFRVQPTESIVR-FPNI-----TN
Omicron_BQ.1_spike/1-1267 PLSETKCTLKSFTVEKGIYQTSNFRVQPTESIVR-FPNI-----TN
Omicron_BQ.1.1_spike/1-1267 PLSETKCTLKSFTVEKGIYQTSNFRVQPTESIVR-FPNI-----TN
Omicron_CH.1.1_spike/1-1270 PLSETKCTLKSFTVEKGIYQTSNFRVQPTESIVR-FPNI-----TN
Omicron_XBB_spike/1-1269 PLSETKCTLKSFTVEKGIYQTSNFRVQPTESIVR-FPNI-----TN
Omicron_XBB.1.5_spike/1-1269 PLSETKCTLKSFTVEKGIYQTSNFRVQPTESIVR-FPNI-----TN
Conservation
6*8879*867**6995*9*88*9*884*8479*8-7772-----87
Quality
Consensus
PLSETKCTLKSFTVEKGIYQTSNFRVQPTESIVR-FPNI-----TN
Occupancy

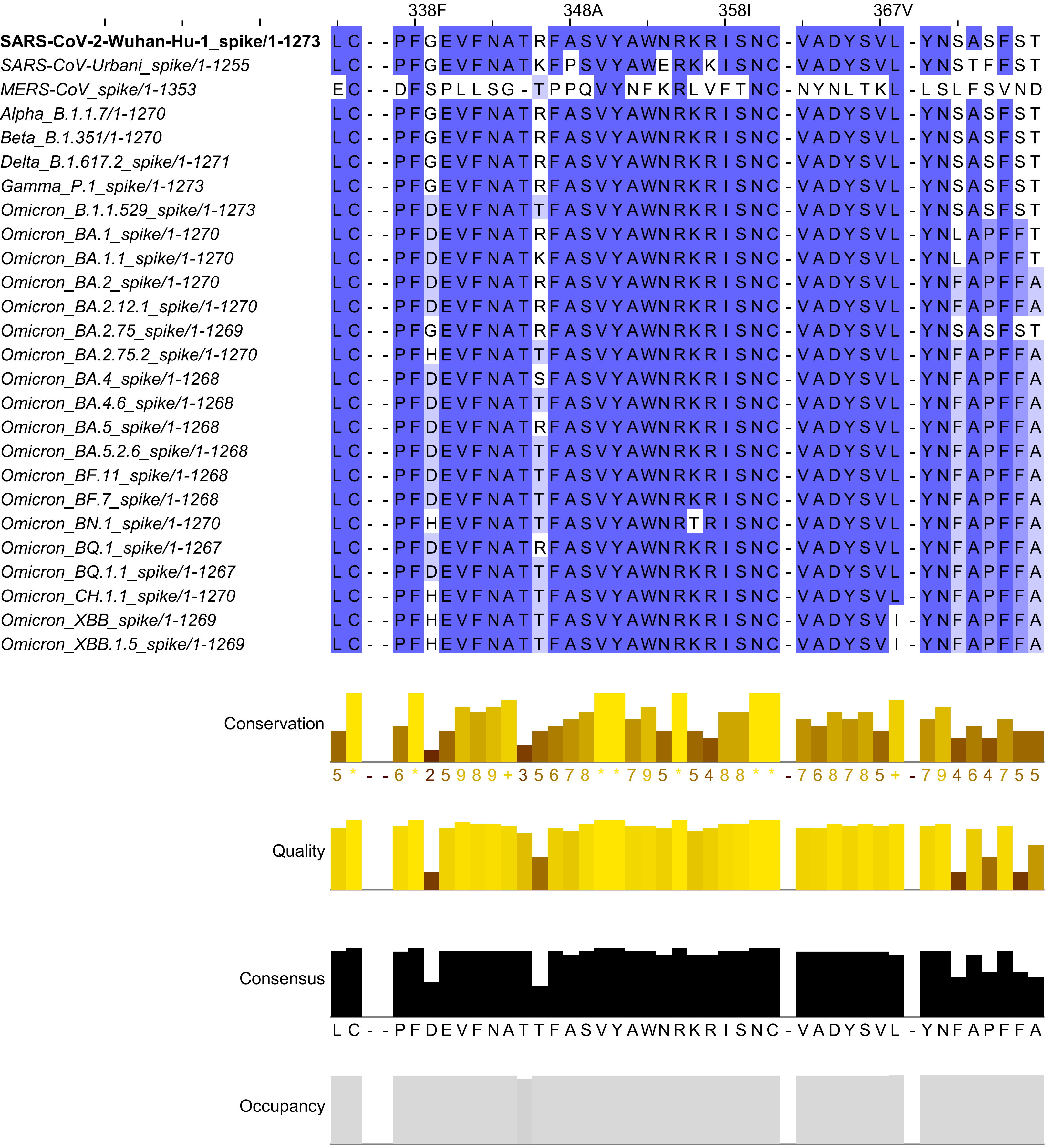
338F
348A
358I
367V
SARS-CoV-2-Wuhan-Hu-1_spike/1-1273
SARS-CoV-Urbani_spike/1-1255
MERS-CoV_spike/1-1353
Alpha_B.1.1.7/1-1270
Beta_B.1.351/1-1270
Delta_B.1.617.2_spike/1-1271
Gamma_P.1_spike/1-1273
Omicron_B.1.1.529_spike/1-1273
Omicron_BA.1_spike/1-1270
Omicron_BA.1.1_spike/1-1270
Omicron_BA.2_spike/1-1270
Omicron_BA.2.12.1_spike/1-1270
Omicron_BA.2.75_spike/1-1269
Omicron_BA.2.75.2_spike/1-1270
Omicron_BA.4_spike/1-1268
Omicron_BA.4.6_spike/1-1268
Omicron_BA.5_spike/1-1268
Omicron_BA.5.2.6_spike/1-1268
Omicron_BF.11_spike/1-1268
Omicron_BF.7_spike/1-1268
Omicron_BN.1_spike/1-1270
Omicron_BQ.1_spike/1-1267
Omicron_BQ.1.1_spike/1-1267
Omicron_CH.1.1_spike/1-1270
Omicron_XBB_spike/1-1269
Omicron_XBB.1.5_spike/1-1269
Conservation
Quality
Consensus
LC--PFDEVFNATTFASVYAWNRKRISNC-VADYSVL-YNFAPFFA
Occupancy

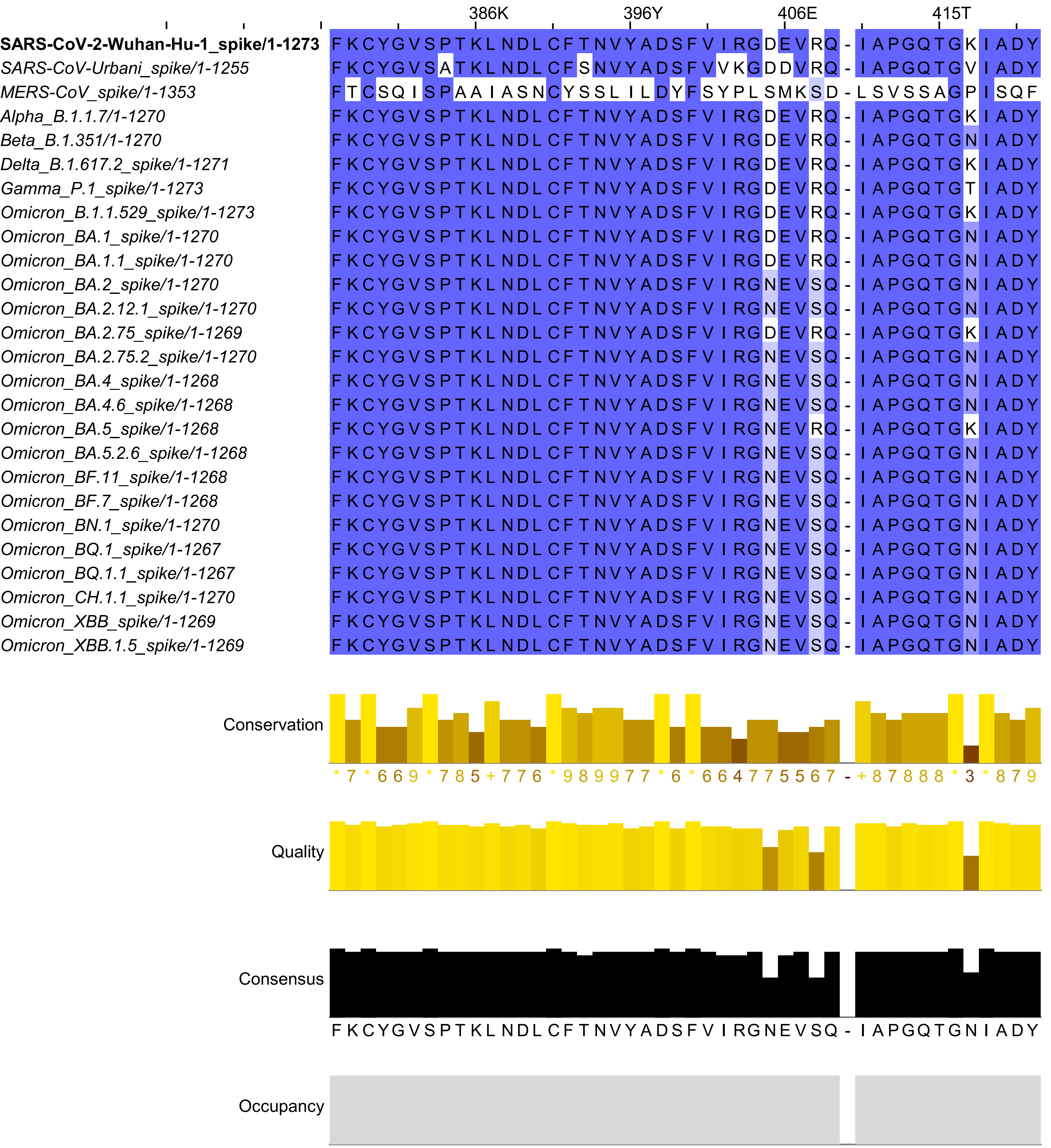
386K
396Y
406E
415T
SARS-CoV-2-Wuhan-Hu-1_spike/1-1273
SARS-CoV-Urbani_spike/1-1255
MERS-CoV_spike/1-1353
Alpha_B.1.1.7/1-1270
Beta_B.1.351/1-1270
Delta_B.1.617.2_spike/1-1271
Gamma_P.1_spike/1-1273
Omicron_B.1.1.529_spike/1-1273
Omicron_BA.1_spike/1-1270
Omicron_BA.1.1_spike/1-1270
Omicron_BA.2_spike/1-1270
Omicron_BA.2.12.1_spike/1-1270
Omicron_BA.2.75_spike/1-1269
Omicron_BA.2.75.2_spike/1-1270
Omicron_BA.4_spike/1-1268
Omicron_BA.4.6_spike/1-1268
Omicron_BA.5_spike/1-1268
Omicron_BA.5.2.6_spike/1-1268
Omicron_BF.11_spike/1-1268
Omicron_BF.7_spike/1-1268
Omicron_BN.1_spike/1-1270
Omicron_BQ.1_spike/1-1267
Omicron_BQ.1.1_spike/1-1267
Omicron_CH.1.1_spike/1-1270
Omicron_XBB_spike/1-1269
Omicron_XBB.1.5_spike/1-1269
Conservation
Quality
Consensus
FKCYGVSPTKLNDLCFTNVYADSFVIRGNEVSQ-IAPGQTGNIADY
Occupancy

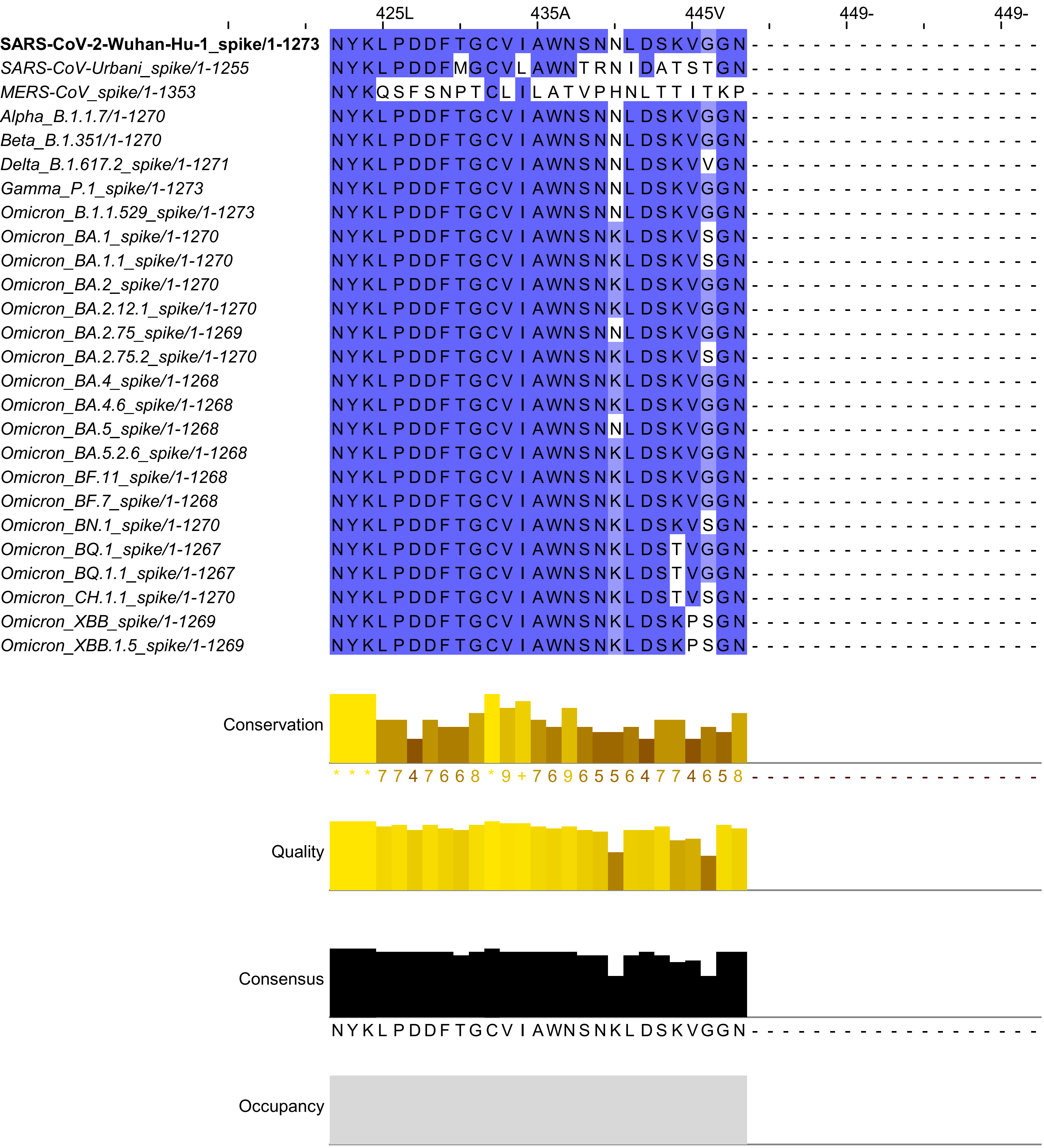
425L
435A
445V
449-
449-
SARS-CoV-2-Wuhan-Hu-1_spike/1-1273 NYKLPDDFTGCVIAWNSNNLDSKVGGN
SARS-CoV-Urbani_spike/1-1255 NYKLPDDFMGCVLAWNTRNIDATSTGN
MERS-CoV_spike/1-1353 NYKQSFSNPTCLILATVPHNLTTITKP
Alpha_B.1.1.7/1-1270 NYKLPDDFTGCVIAWNSNNLDSKVGGN
Beta_B.1.351/1-1270 NYKLPDDFTGCVIAWNSNNLDSKVGGN
Delta_B.1.617.2_spike/1-1271 NYKLPDDFTGCVIAWNSNNLDSKVVGN
Gamma_P.1_spike/1-1273 NYKLPDDFTGCVIAWNSNNLDSKVGGN
Omicron_B.1.1.529_spike/1-1273 NYKLPDDFTGCVIAWNSNNLDSKVGGN
Omicron_BA.1_spike/1-1270 NYKLPDDFTGCVIAWNSNKLDSKVSGN
Omicron_BA.1.1_spike/1-1270 NYKLPDDFTGCVIAWNSNKLDSKVSGN
Omicron_BA.2_spike/1-1270 NYKLPDDFTGCVIAWNSNKLDSKVGGN
Omicron_BA.2.12.1_spike/1-1270 NYKLPDDFTGCVIAWNSNKLDSKVGGN
Omicron_BA.2.75_spike/1-1269 NYKLPDDFTGCVIAWNSNNLDSKVGGN
Omicron_BA.2.75.2_spike/1-1270 NYKLPDDFTGCVIAWNSNKLDSKVSGN
Omicron_BA.4_spike/1-1268 NYKLPDDFTGCVIAWNSNKLDSKVGGN
Omicron_BA.4.6_spike/1-1268 NYKLPDDFTGCVIAWNSNKLDSKVGGN
Omicron_BA.5_spike/1-1268 NYKLPDDFTGCVIAWNSNNLDSKVGGN
Omicron_BA.5.2.6_spike/1-1268 NYKLPDDFTGCVIAWNSNKLDSKVGGN
Omicron_BF.11_spike/1-1268 NYKLPDDFTGCVIAWNSNKLDSKVGGN
Omicron_BF.7_spike/1-1268 NYKLPDDFTGCVIAWNSNKLDSKVGGN
Omicron_BN.1_spike/1-1270 NYKLPDDFTGCVIAWNSNKLDSKVSGN
Omicron_BQ.1_spike/1-1267 NYKLPDDFTGCVIAWNSNKLDSTVGGN
Omicron_BQ.1.1_spike/1-1267 NYKLPDDFTGCVIAWNSNKLDSTVGGN
Omicron_CH.1.1_spike/1-1270 NYKLPDDFTGCVIAWNSNKLDSTVSGN
Omicron_XBB_spike/1-1269 NYKLPDDFTGCVIAWNSNKLDSKPSGN
Omicron_XBB.1.5_spike/1-1269 NYKLPDDFTGCVIAWNSNKLDSKPSGN
Conservation
* * * 7 7 4 7 6 6 8 * 9 + 7 6 9 6 5 5 6 4 7 7 4 6 5 8
Quality
Consensus
NYKLPDDFTGCVIAWNSNKLDSKVGGN
Occupancy

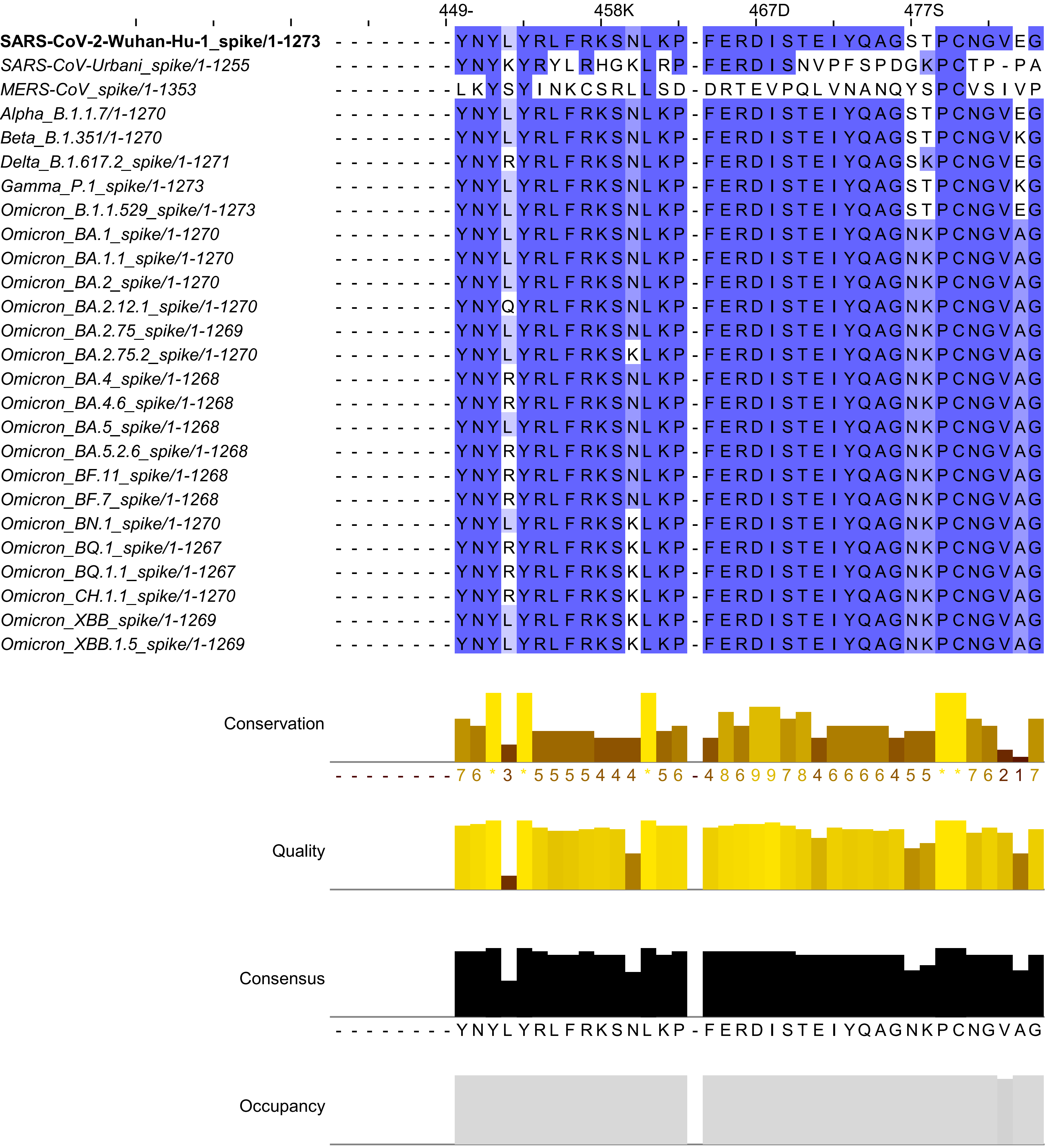
449-
458K
467D
477S
SARS-CoV-2-Wuhan-Hu-1_spike/1-1273 --------YNYLYRLFRKSNLKP-FERDISTEIYQAGSTPCNGVEG
SARS-CoV-Urbani_spike/1-1255 --------YNYKYRYLRHGKLRP-FERDISNVPFSPDGKPCTP-PA
MERS-CoV_spike/1-1353 --------LKYSYINKCSRLLSD-DRTEVPQLVNANQYSPCVSIVP
Alpha_B.1.1.7/1-1270 --------YNYLYRLFRKSNLKP-FERDISTEIYQAGSTPCNGVEG
Beta_B.1.351/1-1270 --------YNYLYRLFRKSNLKP-FERDISTEIYQAGSTPCNGVKG
Delta_B.1.617.2_spike/1-1271 --------YNYRYRLFRKSNLKP-FERDISTEIYQAGSKPCNGVEG
Gamma_P.1_spike/1-1273 --------YNYLYRLFRKSNLKP-FERDISTEIYQAGSTPCNGVKG
Omicron_B.1.1.529_spike/1-1273 --------YNYLYRLFRKSNLKP-FERDISTEIYQAGSTPCNGVEG
Omicron_BA.1_spike/1-1270 --------YNYLYRLFRKSNLKP-FERDISTEIYQAGNKPCNGVAG
Omicron_BA.1.1_spike/1-1270 --------YNYLYRLFRKSNLKP-FERDISTEIYQAGNKPCNGVAG
Omicron_BA.2_spike/1-1270 --------YNYLYRLFRKSNLKP-FERDISTEIYQAGNKPCNGVAG
Omicron_BA.2.12.1_spike/1-1270 --------YNYQYRLFRKSNLKP-FERDISTEIYQAGNKPCNGVAG
Omicron_BA.2.75_spike/1-1269 --------YNYLYRLFRKSNLKP-FERDISTEIYQAGNKPCNGVAG
Omicron_BA.2.75.2_spike/1-1270 --------YNYLYRLFRKSKLKP-FERDISTEIYQAGNKPCNGVAG
Omicron_BA.4_spike/1-1268 --------YNYRYRLFRKSNLKP-FERDISTEIYQAGNKPCNGVAG
Omicron_BA.4.6_spike/1-1268 --------YNYRYRLFRKSNLKP-FERDISTEIYQAGNKPCNGVAG
Omicron_BA.5_spike/1-1268 --------YNYLYRLFRKSNLKP-FERDISTEIYQAGNKPCNGVAG
Omicron_BA.5.2.6_spike/1-1268 --------YNYRYRLFRKSNLKP-FERDISTEIYQAGNKPCNGVAG
Omicron_BF.11_spike/1-1268 --------YNYRYRLFRKSNLKP-FERDISTEIYQAGNKPCNGVAG
Omicron_BF.7_spike/1-1268 --------YNYRYRLFRKSNLKP-FERDISTEIYQAGNKPCNGVAG
Omicron_BN.1_spike/1-1270 --------YNYLYRLFRKSKLKP-FERDISTEIYQAGNKPCNGVAG
Omicron_BQ.1_spike/1-1267 --------YNYRYRLFRKSKLKP-FERDISTEIYQAGNKPCNGVAG
Omicron_BQ.1.1_spike/1-1267 --------YNYRYRLFRKSKLKP-FERDISTEIYQAGNKPCNGVAG
Omicron_CH.1.1_spike/1-1270 --------YNYRYRLFRKSKLKP-FERDISTEIYQAGNKPCNGVAG
Omicron_XBB_spike/1-1269 --------YNYLYRLFRKSKLKP-FERDISTEIYQAGNKPCNGVAG
Omicron_XBB.1.5_spike/1-1269 --------YNYLYRLFRKSKLKP-FERDISTEIYQAGNKPCNGVAG
Conservation
--------76*3*5555444*56-4869978466664 55**76217
Quality
Consensus
--------YNYLYRLFRKSNLKP-FERDISTEIYQAGNKPCNGVAG
Occupancy

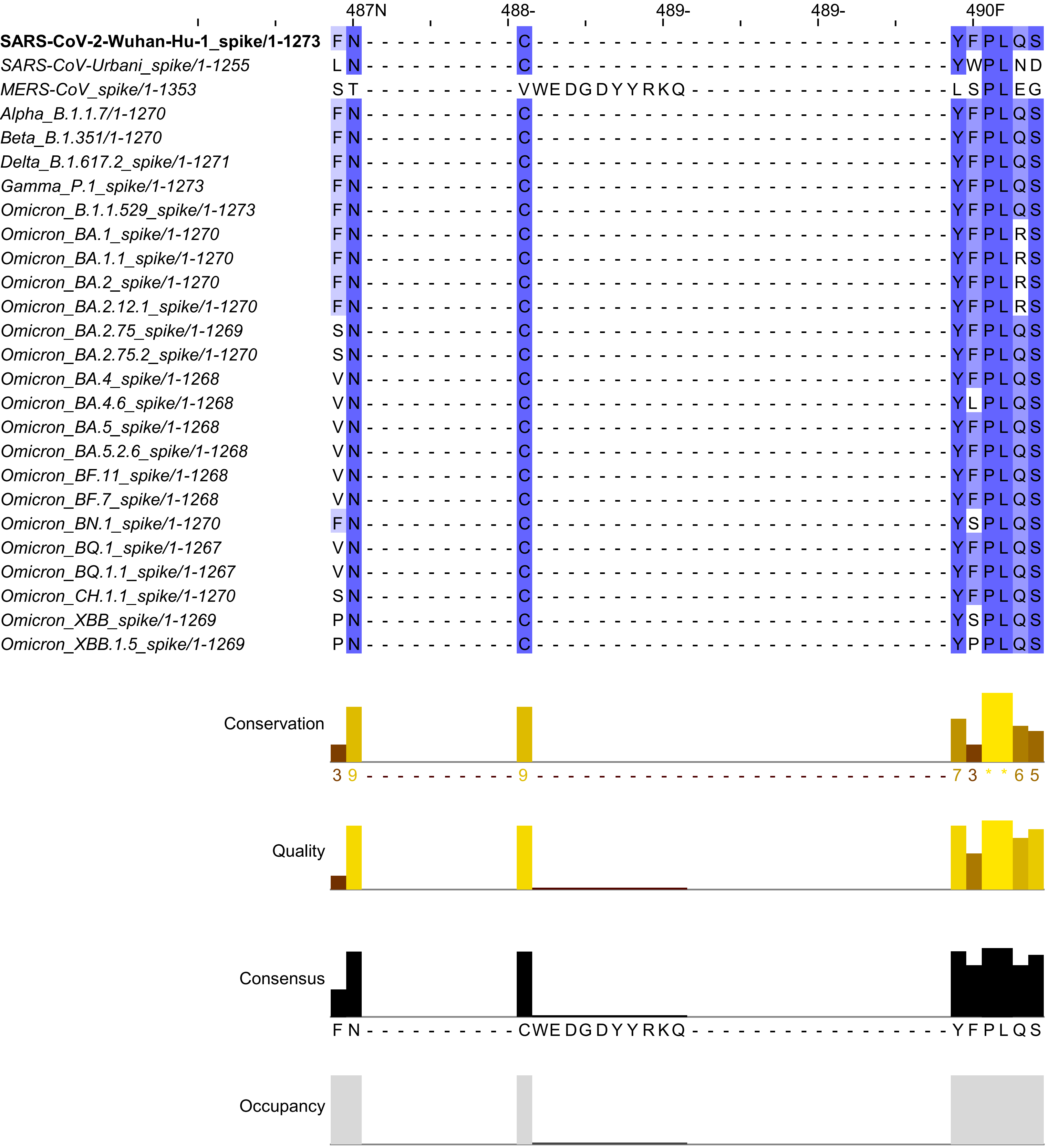

487N
488-
489-
489-
490F
SARS-CoV-2-Wuhan-Hu-1_spike/1-1273 F N - - - - - - - - - - C - Y F P L Q S
SARS-CoV-Urbani_spike/1-1255 L N - - - - - - - - - - C - Y W P L N D
MERS-CoV_spike/1-1353 S T - - - - - - - - - - V W E D G D Y Y R K Q - - - - - - - - - - - - - - - - L S P L E G
Alpha_B.1.1.7/1-1270 F N - - - - - - - - - - C - Y F P L Q S
Beta_B.1.351/1-1270 F N - - - - - - - - - - C - Y F P L Q S
Delta_B.1.617.2_spike/1-1271 F N - - - - - - - - - - C - Y F P L Q S
Gamma_P.1_spike/1-1273 F N - - - - - - - - - - C - Y F P L Q S
Omicron_B.1.1.529_spike/1-1273 F N - - - - - - - - - - C - Y F P L Q S
Omicron_BA.1_spike/1-1270 F N - - - - - - - - - - C - Y F P L R S
Omicron_BA.1.1_spike/1-1270 F N - - - - - - - - - - C - Y F P L R S
Omicron_BA.2_spike/1-1270 F N - - - - - - - - - - C - Y F P L R S
Omicron_BA.2.12.1_spike/1-1270 F N - - - - - - - - - - C - Y F P L R S
Omicron_BA.2.75_spike/1-1269 S N - - - - - - - - - - C - Y F P L Q S
Omicron_BA.2.75.2_spike/1-1270 S N - - - - - - - - - - C - Y F P L Q S
Omicron_BA.4_spike/1-1268 V N - - - - - - - - - - C - Y F P L Q S
Omicron_BA.4.6_spike/1-1268 V N - - - - - - - - - - C - Y L P L Q S
Omicron_BA.5_spike/1-1268 V N - - - - - - - - - - C - Y F P L Q S
Omicron_BA.5.2.6_spike/1-1268 V N - - - - - - - - - - C - Y F P L Q S
Omicron_BF.11_spike/1-1268 V N - - - - - - - - - - C - Y F P L Q S
Omicron_BF.7_spike/1-1268 V N - - - - - - - - - - C - Y F P L Q S
Omicron_BN.1_spike/1-1270 F N - - - - - - - - - - C - Y S P L Q S
Omicron_BQ.1_spike/1-1267 V N - - - - - - - - - - C - Y F P L Q S
Omicron_BQ.1.1_spike/1-1267 V N - - - - - - - - - - C - Y F P L Q S
Omicron_CH.1.1_spike/1-1270 S N - - - - - - - - - - C - Y F P L Q S
Omicron_XBB_spike/1-1269 P N - - - - - - - - - - C - Y S P L Q S
Omicron_XBB.1.5_spike/1-1269 P N - - - - - - - - - - C - Y P P L Q S
Conservation
3 9 - - - - - - - - - - 9 - 7 3 * * 6 5
Quality
Consensus
F N - - - - - - - - - - C W E D G D Y Y R K Q - - - - - - - - - - - - - - - - Y F P L Q S
Occupancy

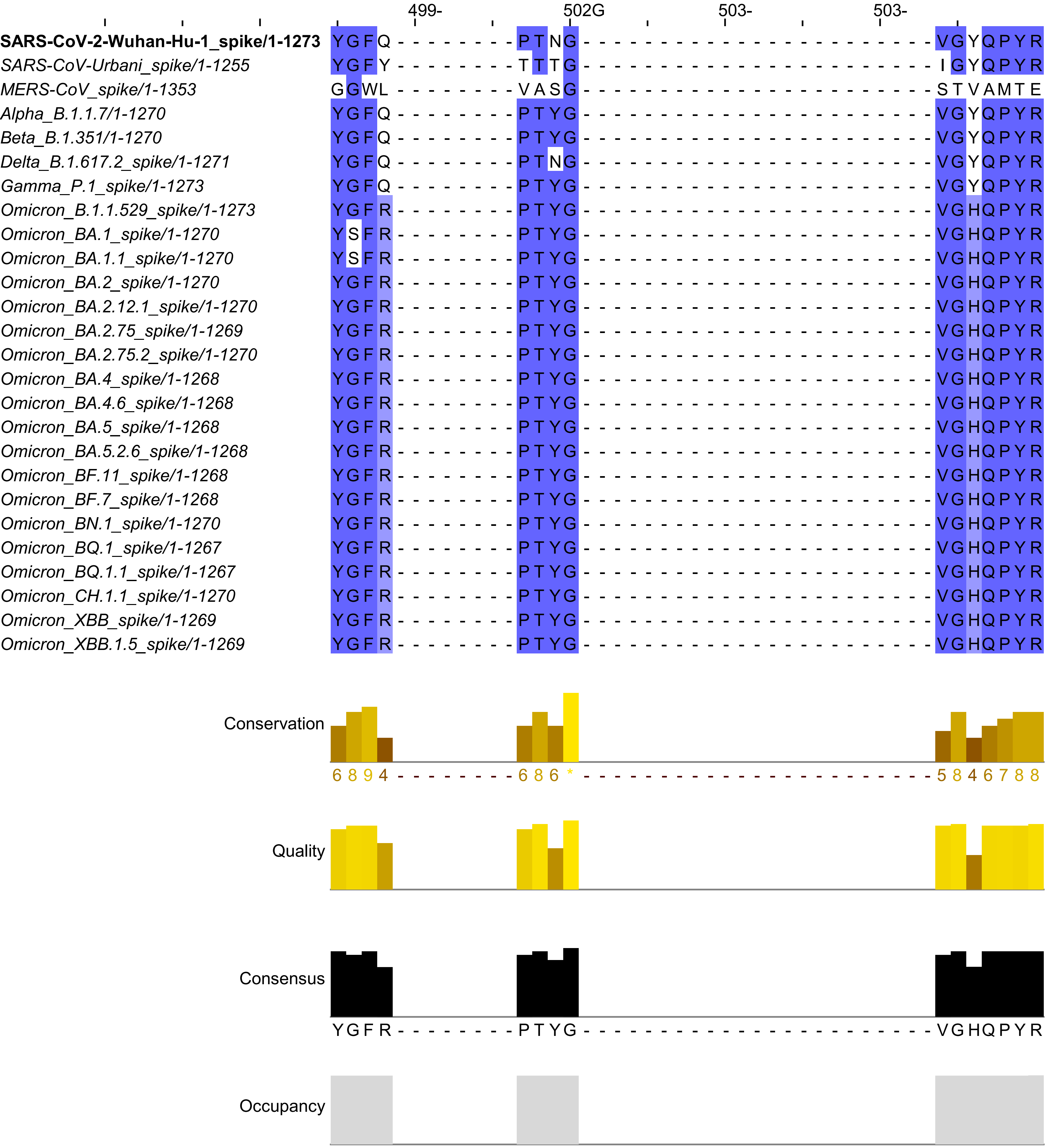
499-
502G
503-
503-
SARS-CoV-2-Wuhan-Hu-1_spike/1-1273 YGFQ--------PTNG-----------------VGYQPYR
SARS-CoV-Urbani_spike/1-1255 YGFY--------TTTG-----------------IGYQPYR
MERS-CoV_spike/1-1353 GGWL--------VASG-----------------STVAMTE
Alpha_B.1.1.7/1-1270 YGFQ--------PTYG-----------------VGYQPYR
Beta_B.1.351/1-1270 YGFQ--------PTYG-----------------VGYQPYR
Delta_B.1.617.2_spike/1-1271 YGFQ--------PTNG-----------------VGYQPYR
Gamma_P.1_spike/1-1273 YGFQ--------PTYG-----------------VGYQPYR
Omicron_B.1.1.529_spike/1-1273 YGFR--------PTYG-----------------VGHQPYR
Omicron_BA.1_spike/1-1270 YSFR--------PTYG-----------------VGHQPYR
Omicron_BA.1.1_spike/1-1270 YSFR--------PTYG-----------------VGHQPYR
Omicron_BA.2_spike/1-1270 YGFR--------PTYG-----------------VGHQPYR
Omicron_BA.2.12.1_spike/1-1270 YGFR--------PTYG-----------------VGHQPYR
Omicron_BA.2.75_spike/1-1269 YGFR--------PTYG-----------------VGHQPYR
Omicron_BA.2.75.2_spike/1-1270 YGFR--------PTYG-----------------VGHQPYR
Omicron_BA.4_spike/1-1268 YGFR--------PTYG-----------------VGHQPYR
Omicron_BA.4.6_spike/1-1268 YGFR--------PTYG-----------------VGHQPYR
Omicron_BA.5_spike/1-1268 YGFR--------PTYG-----------------VGHQPYR
Omicron_BA.5.2.6_spike/1-1268 YGFR--------PTYG-----------------VGHQPYR
Omicron_BF.11_spike/1-1268 YGFR--------PTYG-----------------VGHQPYR
Omicron_BF.7_spike/1-1268 YGFR--------PTYG-----------------VGHQPYR
Omicron_BN.1_spike/1-1270 YGFR--------PTYG-----------------VGHQPYR
Omicron_BQ.1_spike/1-1267 YGFR--------PTYG-----------------VGHQPYR
Omicron_BQ.1.1_spike/1-1267 YGFR--------PTYG-----------------VGHQPYR
Omicron_CH.1.1_spike/1-1270 YGFR--------PTYG-----------------VGHQPYR
Omicron_XBB_spike/1-1269 YGFR--------PTYG-----------------VGHQPYR
Omicron_XBB.1.5_spike/1-1269 YGFR--------PTYG-----------------VGHQPYR
Conservation
6894--------686*-----------------5846788
Quality
Consensus
YGFR--------PTYG-----------------VGHQPYR
Occupancy

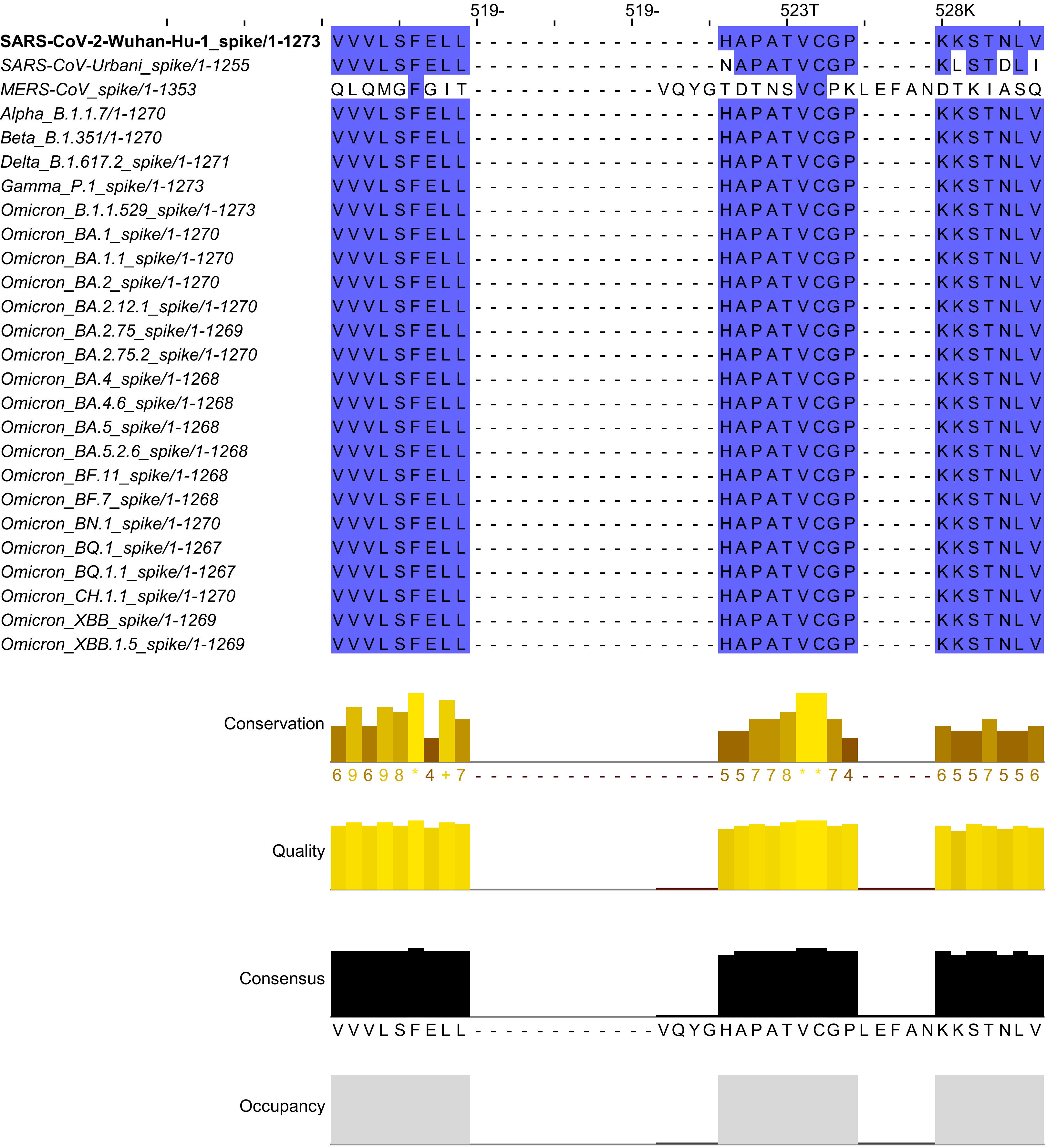
519-
519-
523T
528K
SARS-CoV-2-Wuhan-Hu-1_spike/1-1273 VVVLSFELL----------------HAPATVCGP-----KKSTNLV
SARS-CoV-Urbani_spike/1-1255 VVVLSFELL----------------NAPATVCGP-----KLSTDLI
MERS-CoV_spike/1-1353 QLQMGFGIT------------VQYGTDTNSVCPKLEFANDTKIASQ
Alpha_B.1.1.7/1-1270 VVVLSFELL----------------HAPATVCGP-----KKSTNLV
Beta_B.1.351/1-1270 VVVLSFELL----------------HAPATVCGP-----KKSTNLV
Delta_B.1.617.2_spike/1-1271 VVVLSFELL----------------HAPATVCGP-----KKSTNLV
Gamma_P.1_spike/1-1273 VVVLSFELL----------------HAPATVCGP-----KKSTNLV
Omicron_B.1.1.529_spike/1-1273 VVVLSFELL----------------HAPATVCGP-----KKSTNLV
Omicron_BA.1_spike/1-1270 VVVLSFELL----------------HAPATVCGP-----KKSTNLV
Omicron_BA.1.1_spike/1-1270 VVVLSFELL----------------HAPATVCGP-----KKSTNLV
Omicron_BA.2_spike/1-1270 VVVLSFELL----------------HAPATVCGP-----KKSTNLV
Omicron_BA.2.12.1_spike/1-1270 VVVLSFELL----------------HAPATVCGP-----KKSTNLV
Omicron_BA.2.75_spike/1-1269 VVVLSFELL----------------HAPATVCGP-----KKSTNLV
Omicron_BA.2.75.2_spike/1-1270 VVVLSFELL----------------HAPATVCGP-----KKSTNLV
Omicron_BA.4_spike/1-1268 VVVLSFELL----------------HAPATVCGP-----KKSTNLV
Omicron_BA.4.6_spike/1-1268 VVVLSFELL----------------HAPATVCGP-----KKSTNLV
Omicron_BA.5_spike/1-1268 VVVLSFELL----------------HAPATVCGP-----KKSTNLV
Omicron_BA.5.2.6_spike/1-1268 VVVLSFELL----------------HAPATVCGP-----KKSTNLV
Omicron_BF.11_spike/1-1268 VVVLSFELL----------------HAPATVCGP-----KKSTNLV
Omicron_BF.7_spike/1-1268 VVVLSFELL----------------HAPATVCGP-----KKSTNLV
Omicron_BN.1_spike/1-1270 VVVLSFELL----------------HAPATVCGP-----KKSTNLV
Omicron_BQ.1_spike/1-1267 VVVLSFELL----------------HAPATVCGP-----KKSTNLV
Omicron_BQ.1.1_spike/1-1267 VVVLSFELL----------------HAPATVCGP-----KKSTNLV
Omicron_CH.1.1_spike/1-1270 VVVLSFELL----------------HAPATVCGP-----KKSTNLV
Omicron_XBB_spike/1-1269 VVVLSFELL----------------HAPATVCGP-----KKSTNLV
Omicron_XBB.1.5_spike/1-1269 VVVLSFELL----------------HAPATVCGP-----KKSTNLV
Conservation
69698*4+7----------------55778**74-----6557556
Quality
Consensus
VVVLSFELL------------VQYGHAPATVCGPLEFANKKSTNLV
Occupancy

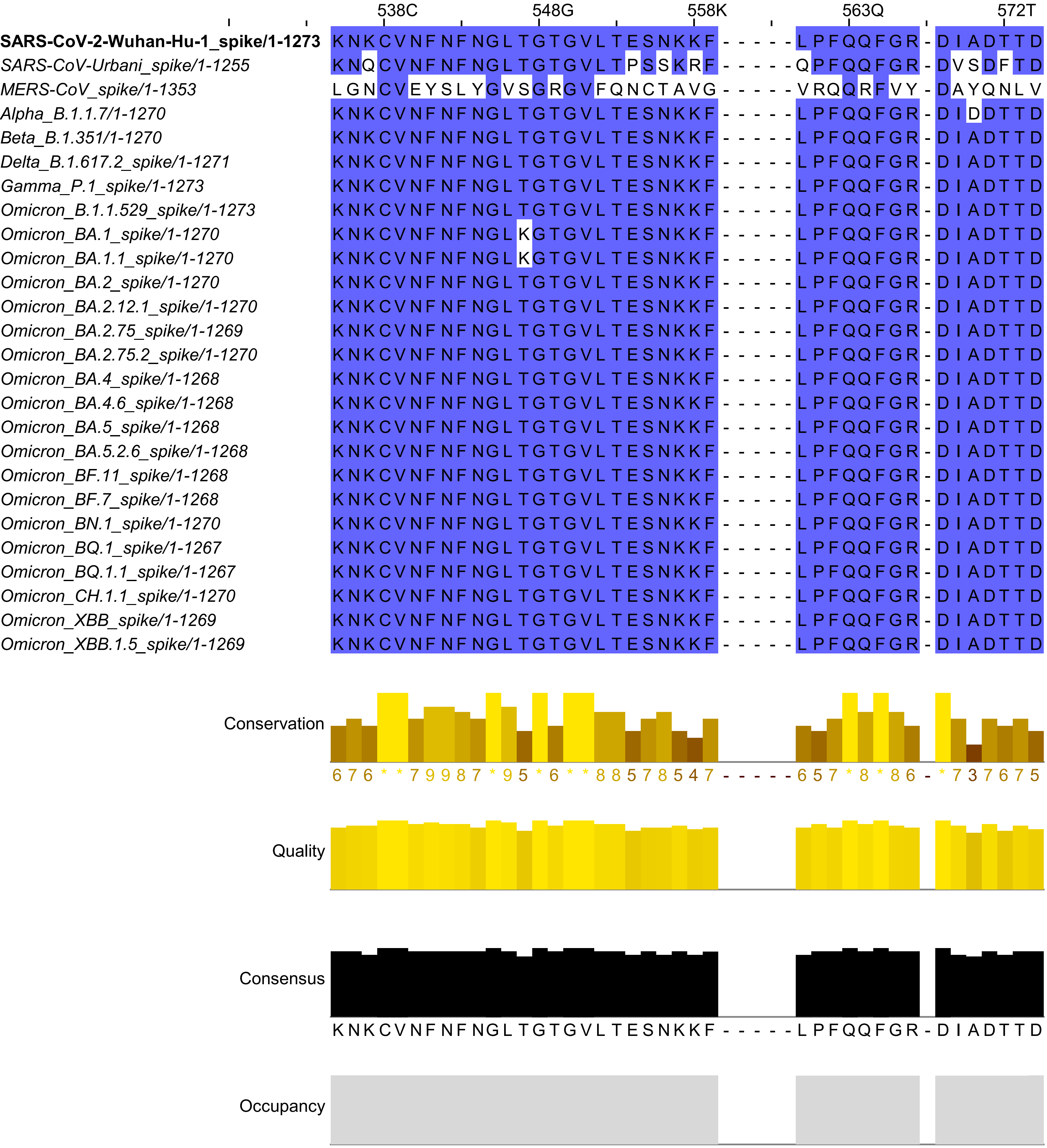
538C
548G
558K
563Q
572T
SARS-CoV-2-Wuhan-Hu-1_spike/1-1273
SARS-CoV-Urbani_spike/1-1255
MERS-CoV_spike/1-1353
Alpha_B.1.1.7/1-1270
Beta_B.1.351/1-1270
Delta_B.1.617.2_spike/1-1271
Gamma_P.1_spike/1-1273
Omicron_B.1.1.529_spike/1-1273
Omicron_BA.1_spike/1-1270
Omicron_BA.1.1_spike/1-1270
Omicron_BA.2_spike/1-1270
Omicron_BA.2.12.1_spike/1-1270
Omicron_BA.2.75_spike/1-1269
Omicron_BA.2.75.2_spike/1-1270
Omicron_BA.4_spike/1-1268
Omicron_BA.4.6_spike/1-1268
Omicron_BA.5_spike/1-1268
Omicron_BA.5.2.6_spike/1-1268
Omicron_BF.11_spike/1-1268
Omicron_BF.7_spike/1-1268
Omicron_BN.1_spike/1-1270
Omicron_BQ.1_spike/1-1267
Omicron_BQ.1.1_spike/1-1267
Omicron_CH.1.1_spike/1-1270
Omicron_XBB_spike/1-1269
Omicron_XBB.1.5_spike/1-1269
Conservation
Quality
Consensus
Occupancy

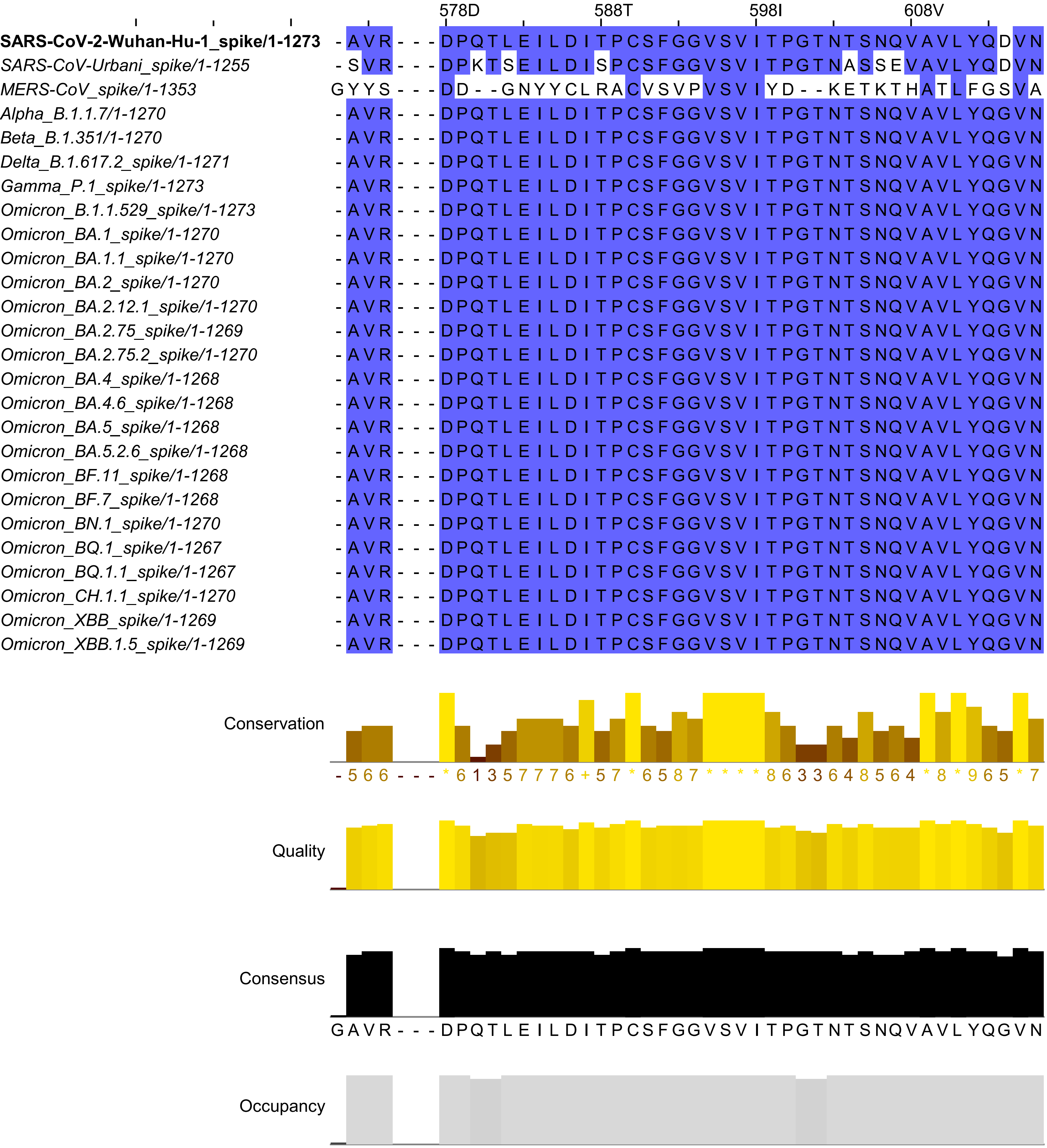
578D 588T 598I 608V
SARS-CoV-2-Wuhan-Hu-1_spike/1-1273 -AVR---DPQTLEILDITPCSFGGVSVITPGTNTSNQVAVLYQDVN
SARS-CoV-Urbani_spike/1-1255 -SVR---DPKTSEILDISPCSFGGVSVITPGTNASSEVAVLYQDVN
MERS-CoV_spike/1-1353 GYYS---DD--GNYYCLRACVSVPVSVIYD--KETKTHATLFGSVA
Alpha_B.1.1.7/1-1270 -AVR---DPQTLEILDITPCSFGGVSVITPGTNTSNQVAVLYQGVN
Beta_B.1.351/1-1270 -AVR---DPQTLEILDITPCSFGGVSVITPGTNTSNQVAVLYQGVN
Delta_B.1.617.2_spike/1-1271 -AVR---DPQTLEILDITPCSFGGVSVITPGTNTSNQVAVLYQGVN
Gamma_P.1_spike/1-1273 -AVR---DPQTLEILDITPCSFGGVSVITPGTNTSNQVAVLYQGVN
Omicron_B.1.1.529_spike/1-1273 -AVR---DPQTLEILDITPCSFGGVSVITPGTNTSNQVAVLYQGVN
Omicron_BA.1_spike/1-1270 -AVR---DPQTLEILDITPCSFGGVSVITPGTNTSNQVAVLYQGVN
Omicron_BA.1.1_spike/1-1270 -AVR---DPQTLEILDITPCSFGGVSVITPGTNTSNQVAVLYQGVN
Omicron_BA.2_spike/1-1270 -AVR---DPQTLEILDITPCSFGGVSVITPGTNTSNQVAVLYQGVN
Omicron_BA.2.12.1_spike/1-1270 -AVR---DPQTLEILDITPCSFGGVSVITPGTNTSNQVAVLYQGVN
Omicron_BA.2.75_spike/1-1269 -AVR---DPQTLEILDITPCSFGGVSVITPGTNTSNQVAVLYQGVN
Omicron_BA.2.75.2_spike/1-1270 -AVR---DPQTLEILDITPCSFGGVSVITPGTNTSNQVAVLYQGVN
Omicron_BA.4_spike/1-1268 -AVR---DPQTLEILDITPCSFGGVSVITPGTNTSNQVAVLYQGVN
Omicron_BA.4.6_spike/1-1268 -AVR---DPQTLEILDITPCSFGGVSVITPGTNTSNQVAVLYQGVN
Omicron_BA.5_spike/1-1268 -AVR---DPQTLEILDITPCSFGGVSVITPGTNTSNQVAVLYQGVN
Omicron_BA.5.2.6_spike/1-1268 -AVR---DPQTLEILDITPCSFGGVSVITPGTNTSNQVAVLYQGVN
Omicron_BF.11_spike/1-1268 -AVR---DPQTLEILDITPCSFGGVSVITPGTNTSNQVAVLYQGVN
Omicron_BF.7_spike/1-1268 -AVR---DPQTLEILDITPCSFGGVSVITPGTNTSNQVAVLYQGVN
Omicron_BN.1_spike/1-1270 -AVR---DPQTLEILDITPCSFGGVSVITPGTNTSNQVAVLYQGVN
Omicron_BQ.1_spike/1-1267 -AVR---DPQTLEILDITPCSFGGVSVITPGTNTSNQVAVLYQGVN
Omicron_BQ.1.1_spike/1-1267 -AVR---DPQTLEILDITPCSFGGVSVITPGTNTSNQVAVLYQGVN
Omicron_CH.1.1_spike/1-1270 -AVR---DPQTLEILDITPCSFGGVSVITPGTNTSNQVAVLYQGVN
Omicron_XBB_spike/1-1269 -AVR---DPQTLEILDITPCSFGGVSVITPGTNTSNQVAVLYQGVN
Omicron_XBB.1.5_spike/1-1269 -AVR---DPQTLEILDITPCSFGGVSVITPGTNTSNQVAVLYQGVN
Conservation
-566---*6135777 6+57*6587****8633648564*8*965*7
Quality
Consensus
GAVR---DPQTLEILDITPCSFGGVSVITPGTNTSNQVAVLYQGVN
Occupancy

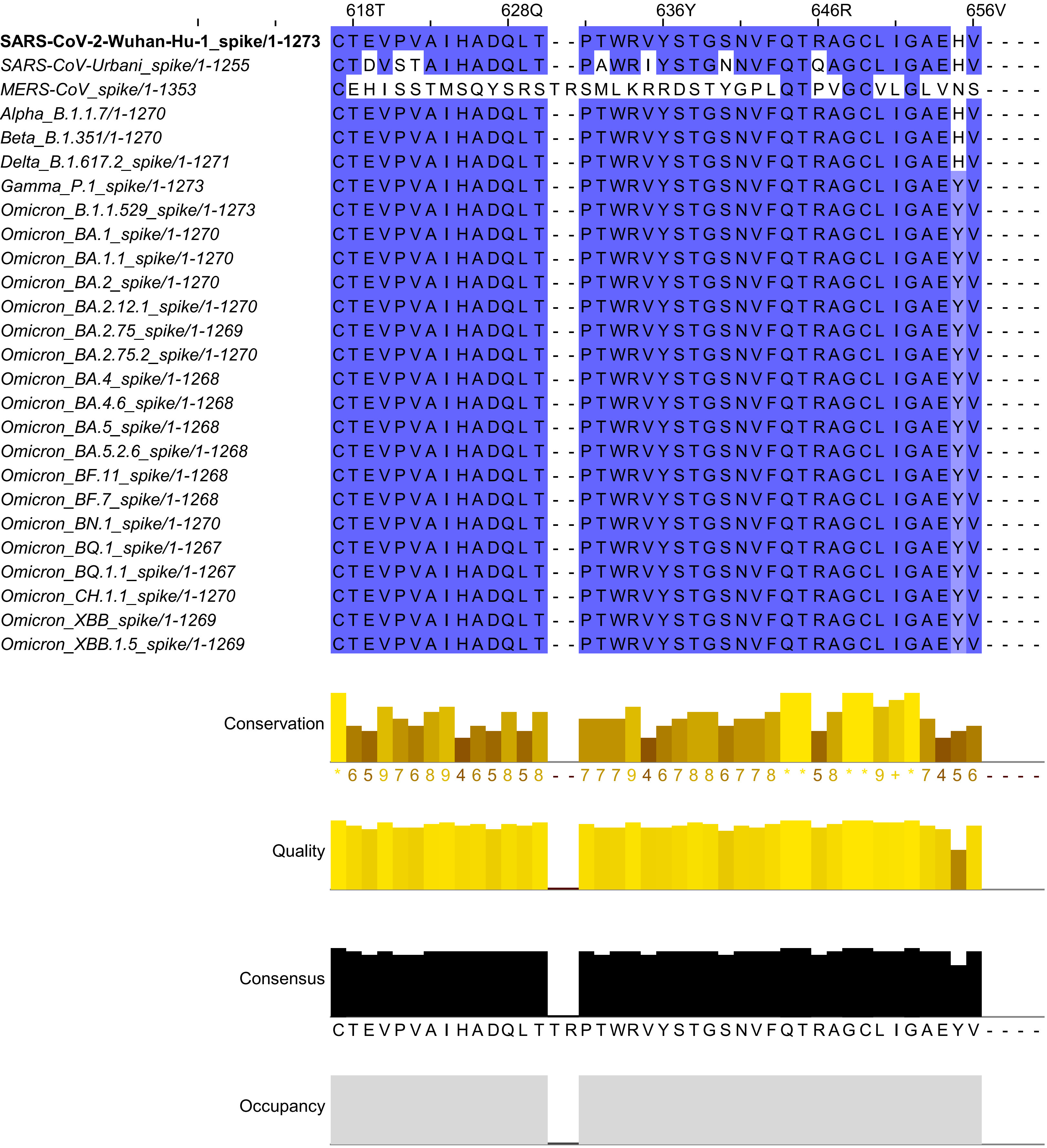
618T
628Q
636Y
646R
656V
SARS-CoV-2-Wuhan-Hu-1_spike/1-1273
SARS-CoV-Urbani_spike/1-1255
MERS-CoV_spike/1-1353
Alpha_B.1.1.7/1-1270
Beta_B.1.351/1-1270
Delta_B.1.617.2_spike/1-1271
Gamma_P.1_spike/1-1273
Omicron_B.1.1.529_spike/1-1273
Omicron_BA.1_spike/1-1270
Omicron_BA.1.1_spike/1-1270
Omicron_BA.2_spike/1-1270
Omicron_BA.2.12.1_spike/1-1270
Omicron_BA.2.75_spike/1-1269
Omicron_BA.2.75.2_spike/1-1270
Omicron_BA.4_spike/1-1268
Omicron_BA.4.6_spike/1-1268
Omicron_BA.5_spike/1-1268
Omicron_BA.5.2.6_spike/1-1268
Omicron_BF.11_spike/1-1268
Omicron_BF.7_spike/1-1268
Omicron_BN.1_spike/1-1270
Omicron_BQ.1_spike/1-1267
Omicron_BQ.1.1_spike/1-1267
Omicron_CH.1.1_spike/1-1270
Omicron_XBB_spike/1-1269
Omicron_XBB.1.5_spike/1-1269
Conservation
Quality
Consensus
CTEVPVAIHADQLTTRPTWRVYSTGSNVFQTRAGCLIGAEYV----
Occupancy

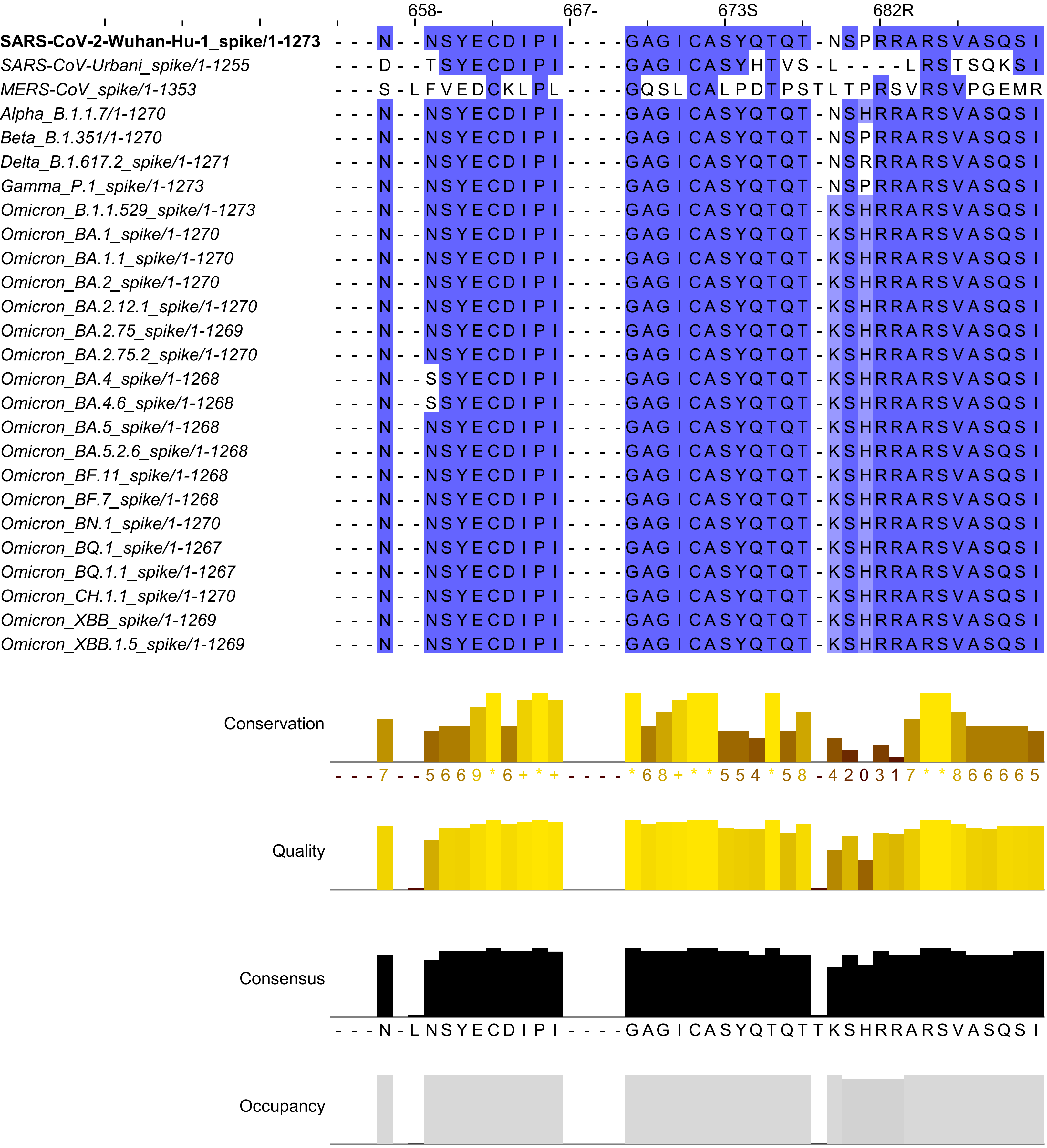
658-
667-
673S
682R
SARS-CoV-2-Wuhan-Hu-1_spike/1-1273
SARS-CoV-Urbani_spike/1-1255
MERS-CoV_spike/1-1353
Alpha_B.1.1.7/1-1270
Beta_B.1.351/1-1270
Delta_B.1.617.2_spike/1-1271
Gamma_P.1_spike/1-1273
Omicron_B.1.1.529_spike/1-1273
Omicron_BA.1_spike/1-1270
Omicron_BA.1.1_spike/1-1270
Omicron_BA.2_spike/1-1270
Omicron_BA.2.12.1_spike/1-1270
Omicron_BA.2.75_spike/1-1269
Omicron_BA.2.75.2_spike/1-1270
Omicron_BA.4_spike/1-1268
Omicron_BA.4.6_spike/1-1268
Omicron_BA.5_spike/1-1268
Omicron_BA.5.2.6_spike/1-1268
Omicron_BF.11_spike/1-1268
Omicron_BF.7_spike/1-1268
Omicron_BN.1_spike/1-1270
Omicron_BQ.1_spike/1-1267
Omicron_BQ.1.1_spike/1-1267
Omicron_CH.1.1_spike/1-1270
Omicron_XBB_spike/1-1269
Omicron_XBB.1.5_spike/1-1269
Conservation
Quality
Consensus
---N-LNSYECDIPI----GAGICASYQTQTTKSHRRARSVASQSI
Occupancy

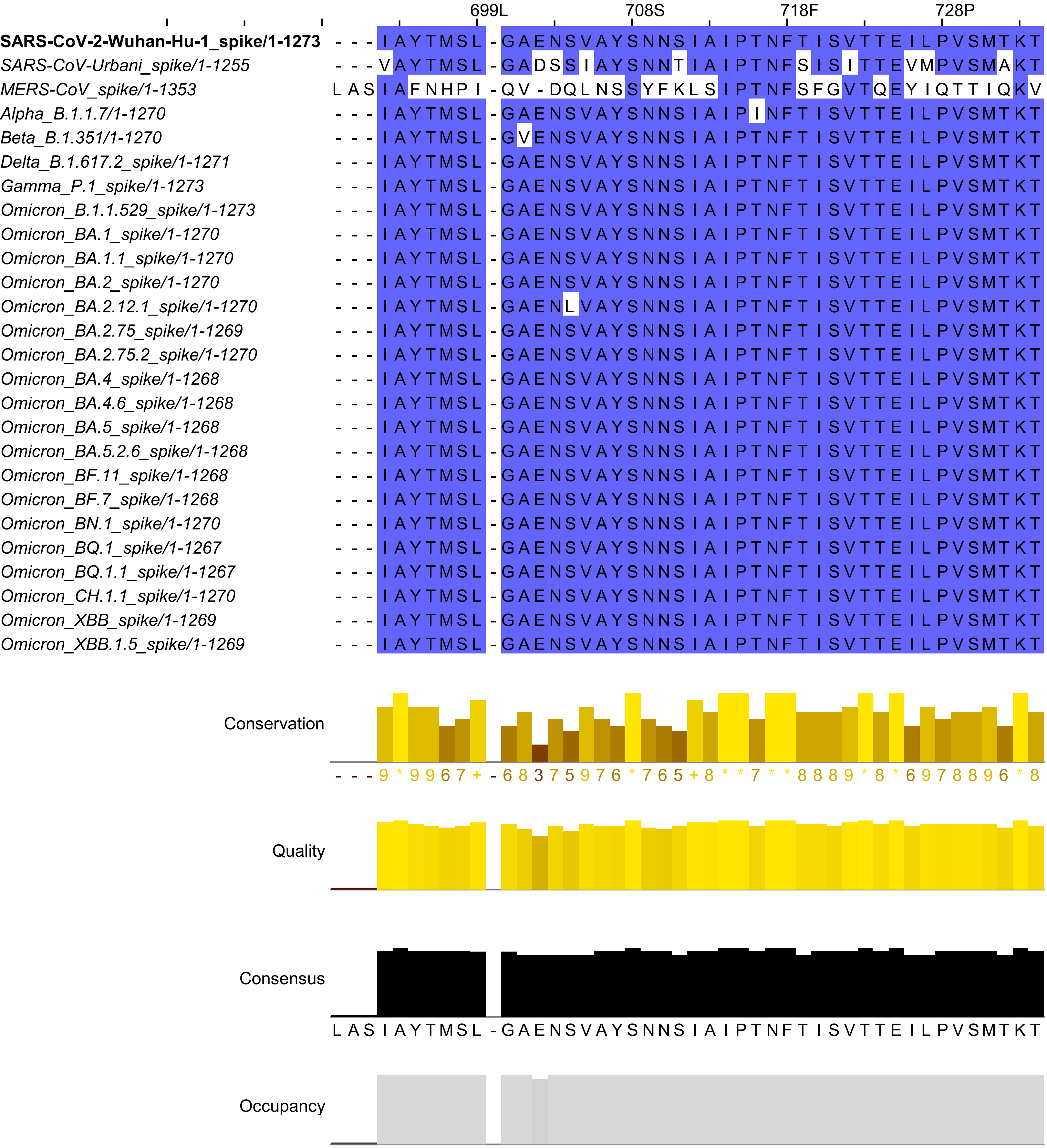
699L
708S
718F
728P
SARS-CoV-2-Wuhan-Hu-1_spike/1-1273 ---IAYTMSL-GAENSVAYSNNSIAIPTNFTISVTTEILPVSMTKT
SARS-CoV-Urbani_spike/1-1255 ---VAYTMSL-GADSSIAYSNNTIAIPTNFSISITTEVMPVSMAKT
MERS-CoV_spike/1-1353 LASIAFNHPI-QV-DQLNSSYFKLSIPTNFSFGVTQEYIQTTIQKV
Alpha_B.1.1.7/1-1270 ---IAYTMSL-GAENSVAYSNNSIAIPINFTISVTTEILPVSMTKT
Beta_B.1.351/1-1270 ---IAYTMSL-GVENSVAYSNNSIAIPTNFTISVTTEILPVSMTKT
Delta_B.1.617.2_spike/1-1271 ---IAYTMSL-GAENSVAYSNNSIAIPTNFTISVTTEILPVSMTKT
Gamma_P.1_spike/1-1273 ---IAYTMSL-GAENSVAYSNNSIAIPTNFTISVTTEILPVSMTKT
Omicron_B.1.1.529_spike/1-1273 ---IAYTMSL-GAENSVAYSNNSIAIPTNFTISVTTEILPVSMTKT
Omicron_BA.1_spike/1-1270 ---IAYTMSL-GAENSVAYSNNSIAIPTNFTISVTTEILPVSMTKT
Omicron_BA.1.1_spike/1-1270 ---IAYTMSL-GAENSVAYSNNSIAIPTNFTISVTTEILPVSMTKT
Omicron_BA.2_spike/1-1270 ---IAYTMSL-GAENSVAYSNNSIAIPTNFTISVTTEILPVSMTKT
Omicron_BA.2.12.1_spike/1-1270 ---IAYTMSL-GAENLVAYSNNSIAIPTNFTISVTTEILPVSMTKT
Omicron_BA.2.75_spike/1-1269 ---IAYTMSL-GAENSVAYSNNSIAIPTNFTISVTTEILPVSMTKT
Omicron_BA.2.75.2_spike/1-1270 ---IAYTMSL-GAENSVAYSNNSIAIPTNFTISVTTEILPVSMTKT
Omicron_BA.4_spike/1-1268 ---IAYTMSL-GAENSVAYSNNSIAIPTNFTISVTTEILPVSMTKT
Omicron_BA.4.6_spike/1-1268 ---IAYTMSL-GAENSVAYSNNSIAIPTNFTISVTTEILPVSMTKT
Omicron_BA.5_spike/1-1268 ---IAYTMSL-GAENSVAYSNNSIAIPTNFTISVTTEILPVSMTKT
Omicron_BA.5.2.6_spike/1-1268 ---IAYTMSL-GAENSVAYSNNSIAIPTNFTISVTTEILPVSMTKT
Omicron_BF.11_spike/1-1268 ---IAYTMSL-GAENSVAYSNNSIAIPTNFTISVTTEILPVSMTKT
Omicron_BF.7_spike/1-1268 ---IAYTMSL-GAENSVAYSNNSIAIPTNFTISVTTEILPVSMTKT
Omicron_BN.1_spike/1-1270 ---IAYTMSL-GAENSVAYSNNSIAIPTNFTISVTTEILPVSMTKT
Omicron_BQ.1_spike/1-1267 ---IAYTMSL-GAENSVAYSNNSIAIPTNFTISVTTEILPVSMTKT
Omicron_BQ.1.1_spike/1-1267 ---IAYTMSL-GAENSVAYSNNSIAIPTNFTISVTTEILPVSMTKT
Omicron_CH.1.1_spike/1-1270 ---IAYTMSL-GAENSVAYSNNSIAIPTNFTISVTTEILPVSMTKT
Omicron_XBB_spike/1-1269 ---IAYTMSL-GAENSVAYSNNSIAIPTNFTISVTTEILPVSMTKT
Omicron_XBB.1.5_spike/1-1269 ---IAYTMSL-GAENSVAYSNNSIAIPTNFTISVTTEILPVSMTKT
Conservation
---9*9967+-68375976*765+8**7**8889*8*697889 6*8
Quality
Consensus
LASIAYTMSL-GAENSVAYSNNSIAIPTNFTISVTTEILPVSMTKT
Occupancy

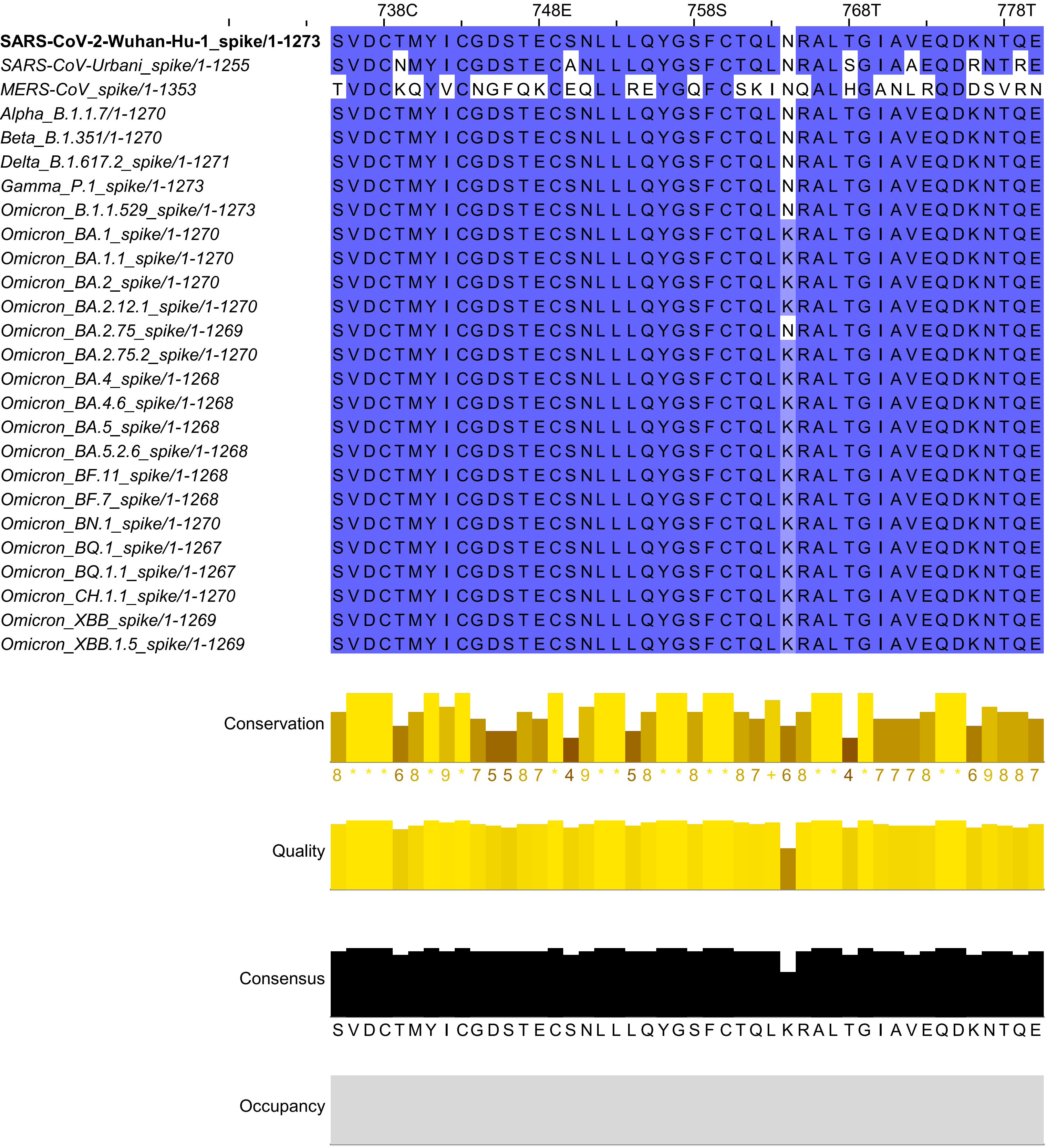
738C
748E
758S
768T
778T
SARS-CoV-2-Wuhan-Hu-1_spike/1-1273 SVDCTMYICGDSTECSNLLLQYGSFCTQLNRALTGIAVEQDKNTQE
SARS-CoV-Urbani_spike/1-1255 SVDCNMYICGDSTECANLLLQYGSFCTQLNRALSGIAAEQDRNTRE
MERS-CoV_spike/1-1353 TVDCKQYVCNGFQKCEQLLREYGQFCSKINQALHGANLRQDDSVRN
Alpha_B.1.1.7/1-1270 SVDCTMYICGDSTECSNLLLQYGSFCTQLNRALTGIAVEQDKNTQE
Beta_B.1.351/1-1270 SVDCTMYICGDSTECSNLLLQYGSFCTQLNRALTGIAVEQDKNTQE
Delta_B.1.617.2_spike/1-1271 SVDCTMYICGDSTECSNLLLQYGSFCTQLNRALTGIAVEQDKNTQE
Gamma_P.1_spike/1-1273 SVDCTMYICGDSTECSNLLLQYGSFCTQLNRALTGIAVEQDKNTQE
Omicron_B.1.1.529_spike/1-1273 SVDCTMYICGDSTECSNLLLQYGSFCTQLNRALTGIAVEQDKNTQE
Omicron_BA.1_spike/1-1270 SVDCTMYICGDSTECSNLLLQYGSFCTQLKRALTGIAVEQDKNTQE
Omicron_BA.1.1_spike/1-1270 SVDCTMYICGDSTECSNLLLQYGSFCTQLKRALTGIAVEQDKNTQE
Omicron_BA.2_spike/1-1270 SVDCTMYICGDSTECSNLLLQYGSFCTQLKRALTGIAVEQDKNTQE
Omicron_BA.2.12.1_spike/1-1270 SVDCTMYICGDSTECSNLLLQYGSFCTQLKRALTGIAVEQDKNTQE
Omicron_BA.2.75_spike/1-1269 SVDCTMYICGDSTECSNLLLQYGSFCTQLNRALTGIAVEQDKNTQE
Omicron_BA.2.75.2_spike/1-1270 SVDCTMYICGDSTECSNLLLQYGSFCTQLKRALTGIAVEQDKNTQE
Omicron_BA.4_spike/1-1268 SVDCTMYICGDSTECSNLLLQYGSFCTQLKRALTGIAVEQDKNTQE
Omicron_BA.4.6_spike/1-1268 SVDCTMYICGDSTECSNLLLQYGSFCTQLKRALTGIAVEQDKNTQE
Omicron_BA.5_spike/1-1268 SVDCTMYICGDSTECSNLLLQYGSFCTQLKRALTGIAVEQDKNTQE
Omicron_BA.5.2.6_spike/1-1268 SVDCTMYICGDSTECSNLLLQYGSFCTQLKRALTGIAVEQDKNTQE
Omicron_BF.11_spike/1-1268 SVDCTMYICGDSTECSNLLLQYGSFCTQLKRALTGIAVEQDKNTQE
Omicron_BF.7_spike/1-1268 SVDCTMYICGDSTECSNLLLQYGSFCTQLKRALTGIAVEQDKNTQE
Omicron_BN.1_spike/1-1270 SVDCTMYICGDSTECSNLLLQYGSFCTQLKRALTGIAVEQDKNTQE
Omicron_BQ.1_spike/1-1267 SVDCTMYICGDSTECSNLLLQYGSFCTQLKRALTGIAVEQDKNTQE
Omicron_BQ.1.1_spike/1-1267 SVDCTMYICGDSTECSNLLLQYGSFCTQLKRALTGIAVEQDKNTQE
Omicron_CH.1.1_spike/1-1270 SVDCTMYICGDSTECSNLLLQYGSFCTQLKRALTGIAVEQDKNTQE
Omicron_XBB_spike/1-1269 SVDCTMYICGDSTECSNLLLQYGSFCTQLKRALTGIAVEQDKNTQE
Omicron_XBB.1.5_spike/1-1269 SVDCTMYICGDSTECSNLLLQYGSFCTQLKRALTGIAVEQDKNTQE
Conservation
8***68*9*75587*49**58**8**87+68**4*7778**69887
Quality
Consensus
SVDCTMYICGDSTECSNLLLQYGSFCTQLKRALTGIAVEQDKNTQE
Occupancy

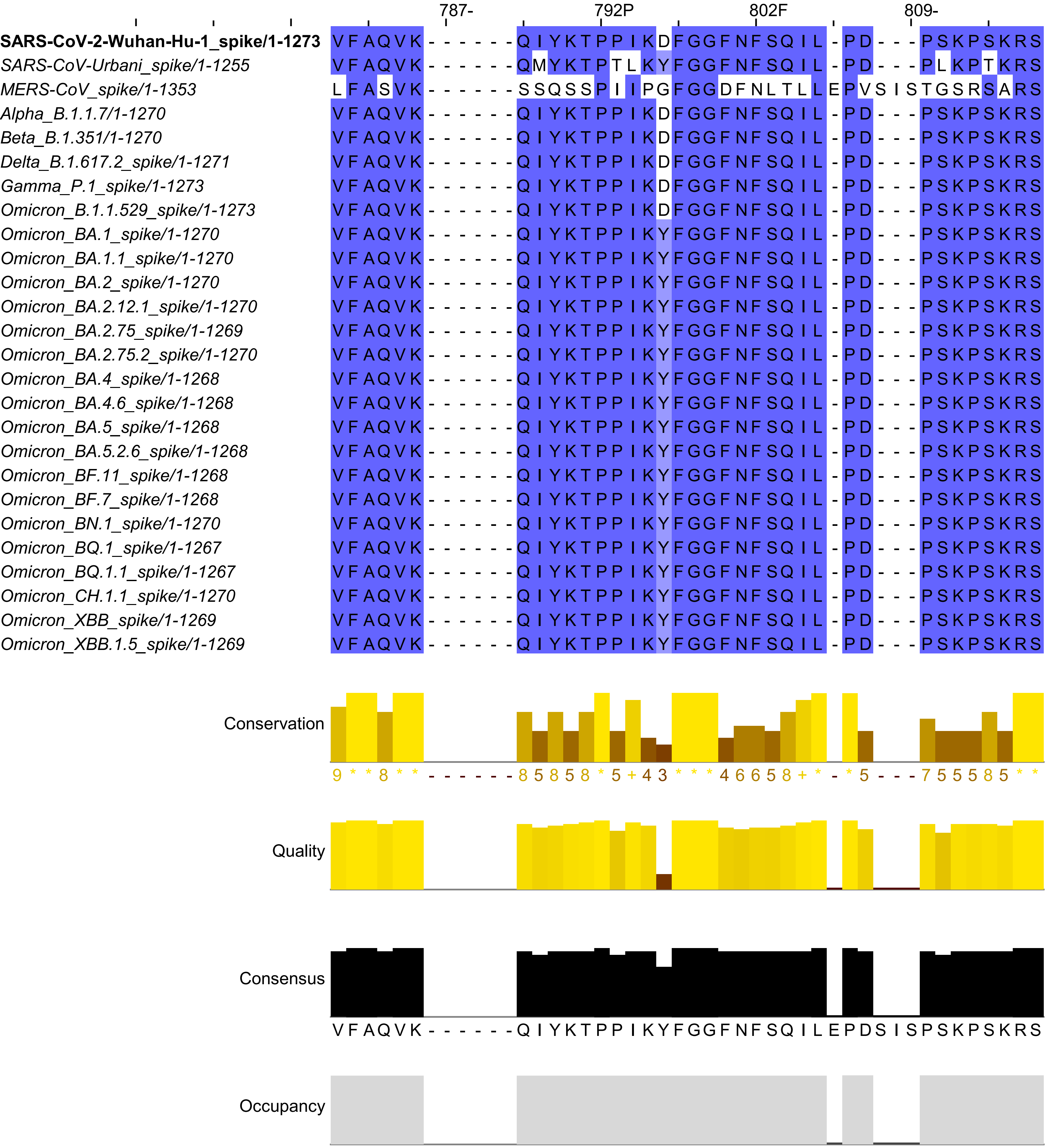
787-
792P
802F
809-
SARS-CoV-2-Wuhan-Hu-1_spike/1-1273 VFAQVK------QIYKTPPIKDFGGFNFSQIL-PD---PSKPSKRS
SARS-CoV-Urbani_spike/1-1255 VFAQVK------QMYKTPTLKYFGGFNFSQIL-PD---PLKPTKRS
MERS-CoV_spike/1-1353 LFASVK------SSQSSPIIPGFGGDFNLTLLEPVSISTGSRSARS
Alpha_B.1.1.7/1-1270 VFAQVK------QIYKTPPIKDFGGFNFSQIL-PD---PSKPSKRS
Beta_B.1.351/1-1270 VFAQVK------QIYKTPPIKDFGGFNFSQIL-PD---PSKPSKRS
Delta_B.1.617.2_spike/1-1271 VFAQVK------QIYKTPPIKDFGGFNFSQIL-PD---PSKPSKRS
Gamma_P.1_spike/1-1273 VFAQVK------QIYKTPPIKDFGGFNFSQIL-PD---PSKPSKRS
Omicron_B.1.1.529_spike/1-1273 VFAQVK------QIYKTPPIKDFGGFNFSQIL-PD---PSKPSKRS
Omicron_BA.1_spike/1-1270 VFAQVK------QIYKTPPIKYFGGFNFSQIL-PD---PSKPSKRS
Omicron_BA.1.1_spike/1-1270 VFAQVK------QIYKTPPIKYFGGFNFSQIL-PD---PSKPSKRS
Omicron_BA.2_spike/1-1270 VFAQVK------QIYKTPPIKYFGGFNFSQIL-PD---PSKPSKRS
Omicron_BA.2.12.1_spike/1-1270 VFAQVK------QIYKTPPIKYFGGFNFSQIL-PD---PSKPSKRS
Omicron_BA.2.75_spike/1-1269 VFAQVK------QIYKTPPIKYFGGFNFSQIL-PD---PSKPSKRS
Omicron_BA.2.75.2_spike/1-1270 VFAQVK------QIYKTPPIKYFGGFNFSQIL-PD---PSKPSKRS
Omicron_BA.4_spike/1-1268 VFAQVK------QIYKTPPIKYFGGFNFSQIL-PD---PSKPSKRS
Omicron_BA.4.6_spike/1-1268 VFAQVK------QIYKTPPIKYFGGFNFSQIL-PD---PSKPSKRS
Omicron_BA.5_spike/1-1268 VFAQVK------QIYKTPPIKYFGGFNFSQIL-PD---PSKPSKRS
Omicron_BA.5.2.6_spike/1-1268 VFAQVK------QIYKTPPIKYFGGFNFSQIL-PD---PSKPSKRS
Omicron_BF.11_spike/1-1268 VFAQVK------QIYKTPPIKYFGGFNFSQIL-PD---PSKPSKRS
Omicron_BF.7_spike/1-1268 VFAQVK------QIYKTPPIKYFGGFNFSQIL-PD---PSKPSKRS
Omicron_BN.1_spike/1-1270 VFAQVK------QIYKTPPIKYFGGFNFSQIL-PD---PSKPSKRS
Omicron_BQ.1_spike/1-1267 VFAQVK------QIYKTPPIKYFGGFNFSQIL-PD---PSKPSKRS
Omicron_BQ.1.1_spike/1-1267 VFAQVK------QIYKTPPIKYFGGFNFSQIL-PD---PSKPSKRS
Omicron_CH.1.1_spike/1-1270 VFAQVK------QIYKTPPIKYFGGFNFSQIL-PD---PSKPSKRS
Omicron_XBB_spike/1-1269 VFAQVK------QIYKTPPIKYFGGFNFSQIL-PD---PSKPSKRS
Omicron_XBB.1.5_spike/1-1269 VFAQVK------QIYKTPPIKYFGGFNFSQIL-PD---PSKPSKRS
Conservation
9**8**------85858*5+43***46658+*-*5---755585**
Quality
Consensus
VFAQVK------QIYKTPPIKYFGGFNFSQILEPDSISPSKPSKRS
Occupancy

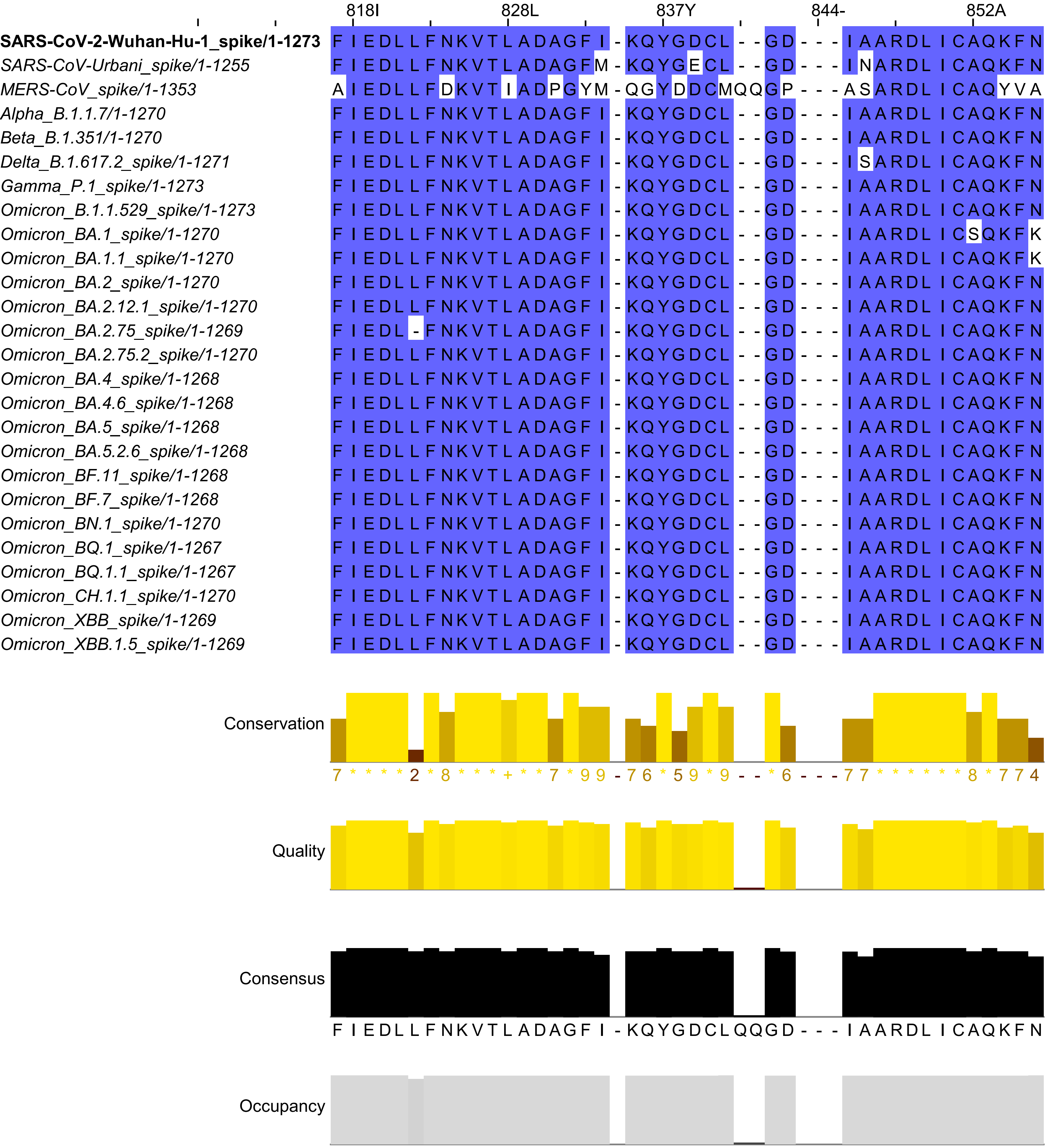

818I
828L
837Y
844-
852A
SARS-CoV-2-Wuhan-Hu-1_spike/1-1273 F I E D L L F N K V T L A D A G F I - K Q Y G D C L - - G D - - - I A A R D L I C A Q K F N
SARS-CoV-Urbani_spike/1-1255 F I E D L L F N K V T L A D A G F M - K Q Y G E C L - - G D - - - I N A R D L I C A Q K F N
MERS-CoV_spike/1-1353 A I E D L L F D K V T I A D P G Y M - Q G Y D D C M Q Q G P - - - A S A R D L I C A Q Y V A
Alpha_B.1.1.7/1-1270 F I E D L L F N K V T L A D A G F I - K Q Y G D C L - - G D - - - I A A R D L I C A Q K F N
Beta_B.1.351/1-1270 F I E D L L F N K V T L A D A G F I - K Q Y G D C L - - G D - - - I A A R D L I C A Q K F N
Delta_B.1.617.2_spike/1-1271 F I E D L L F N K V T L A D A G F I - K Q Y G D C L - - G D - - - I S A R D L I C A Q K F N
Gamma_P.1_spike/1-1273 F I E D L L F N K V T L A D A G F I - K Q Y G D C L - - G D - - - I A A R D L I C A Q K F N
Omicron_B.1.1.529_spike/1-1273 F I E D L L F N K V T L A D A G F I - K Q Y G D C L - - G D - - - I A A R D L I C A Q K F N
Omicron_BA.1_spike/1-1270 F I E D L L F N K V T L A D A G F I - K Q Y G D C L - - G D - - - I A A R D L I C S Q K F K
Omicron_BA.1.1_spike/1-1270 F I E D L L F N K V T L A D A G F I - K Q Y G D C L - - G D - - - I A A R D L I C A Q K F K
Omicron_BA.2_spike/1-1270 F I E D L L F N K V T L A D A G F I - K Q Y G D C L - - G D - - - I A A R D L I C A Q K F N
Omicron_BA.2.12.1_spike/1-1270 F I E D L L F N K V T L A D A G F I - K Q Y G D C L - - G D - - - I A A R D L I C A Q K F N
Omicron_BA.2.75_spike/1-1269 F I E D L - F N K V T L A D A G F I - K Q Y G D C L - - G D - - - I A A R D L I C A Q K F N
Omicron_BA.2.75.2_spike/1-1270 F I E D L L F N K V T L A D A G F I - K Q Y G D C L - - G D - - - I A A R D L I C A Q K F N
Omicron_BA.4_spike/1-1268 F I E D L L F N K V T L A D A G F I - K Q Y G D C L - - G D - - - I A A R D L I C A Q K F N
Omicron_BA.4.6_spike/1-1268 F I E D L L F N K V T L A D A G F I - K Q Y G D C L - - G D - - - I A A R D L I C A Q K F N
Omicron_BA.5_spike/1-1268 F I E D L L F N K V T L A D A G F I - K Q Y G D C L - - G D - - - I A A R D L I C A Q K F N
Omicron_BA.5.2.6_spike/1-1268 F I E D L L F N K V T L A D A G F I - K Q Y G D C L - - G D - - - I A A R D L I C A Q K F N
Omicron_BF.11_spike/1-1268 F I E D L L F N K V T L A D A G F I - K Q Y G D C L - - G D - - - I A A R D L I C A Q K F N
Omicron_BF.7_spike/1-1268 F I E D L L F N K V T L A D A G F I - K Q Y G D C L - - G D - - - I A A R D L I C A Q K F N
Omicron_BN.1_spike/1-1270 F I E D L L F N K V T L A D A G F I - K Q Y G D C L - - G D - - - I A A R D L I C A Q K F N
Omicron_BQ.1_spike/1-1267 F I E D L L F N K V T L A D A G F I - K Q Y G D C L - - G D - - - I A A R D L I C A Q K F N
Omicron_BQ.1.1_spike/1-1267 F I E D L L F N K V T L A D A G F I - K Q Y G D C L - - G D - - - I A A R D L I C A Q K F N
Omicron_CH.1.1_spike/1-1270 F I E D L L F N K V T L A D A G F I - K Q Y G D C L - - G D - - - I A A R D L I C A Q K F N
Omicron_XBB_spike/1-1269 F I E D L L F N K V T L A D A G F I - K Q Y G D C L - - G D - - - I A A R D L I C A Q K F N
Omicron_XBB.1.5_spike/1-1269 F I E D L L F N K V T L A D A G F I - K Q Y G D C L - - G D - - - I A A R D L I C A Q K F N
Conservation
7 * * * * 2 * 8 * * * + * * 7 * 9 9 - 7 6 * 5 9 * 9 - - * 6 - - - 7 7 * * * * * * 8 * 7 7 4
Quality
Consensus
F I E D L L F N K V T L A D A G F I - K Q Y G D C L Q Q G D - - - I A A R D L I C A Q K F N
Occupancy

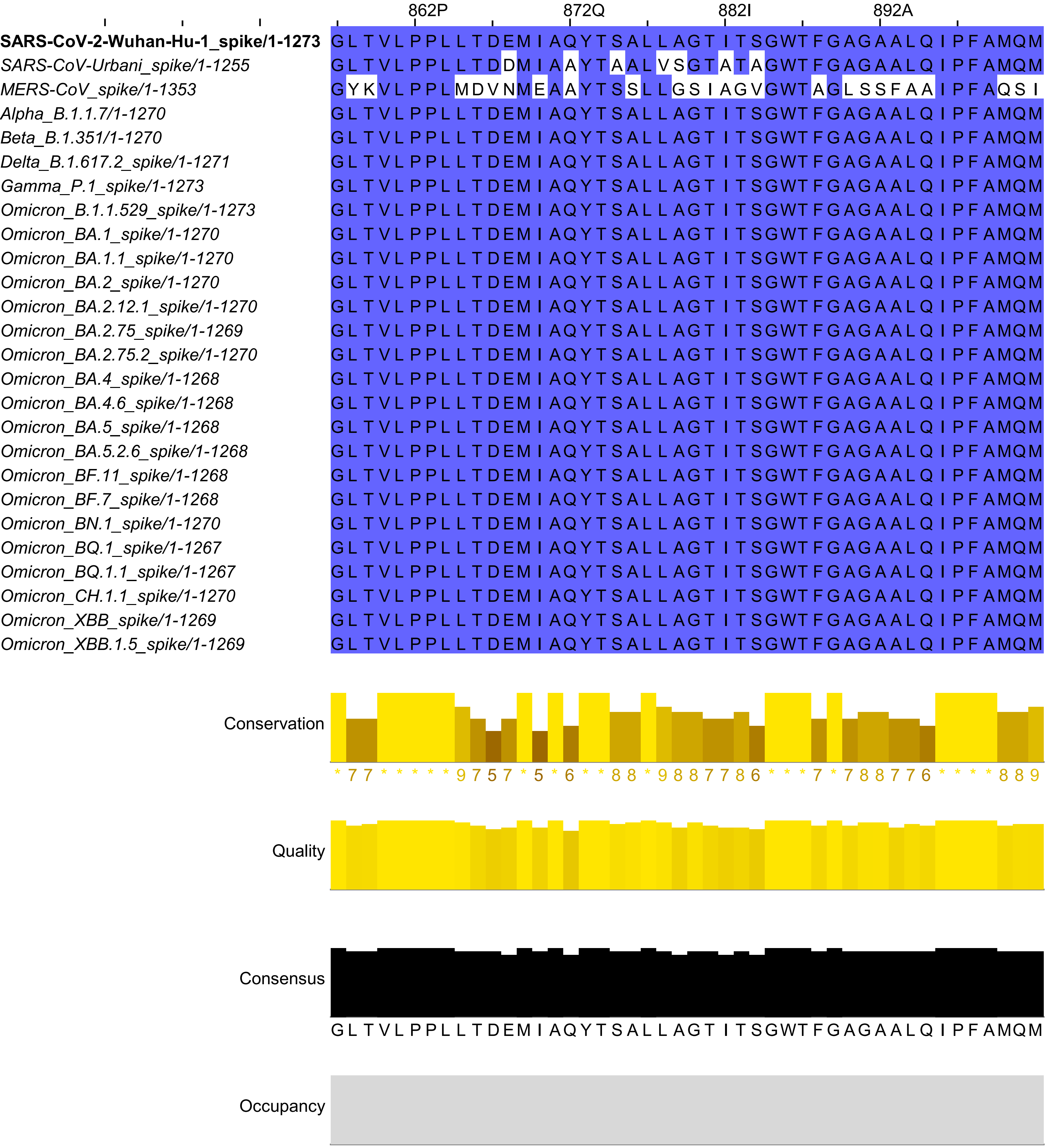

862P
872Q
882I
892A
SARS-CoV-2-Wuhan-Hu-1_spike/1-1273 GLTVLPPLLTDEMIAQYTSALLAGTITSGWTFGAGAALQIPFAMQM
SARS-CoV-Urbani_spike/1-1255 GLTVLPPLLTDDMIAAYTAALVSGTATAGWTFGAGAALQIPFAMQM
MERS-CoV_spike/1-1353 GYKVLPPLMDVNMEAAYTSSLLGSIAGVGWTAGLSSFAAIPFAQSI
Alpha_B.1.1.7/1-1270 GLTVLPPLLTDEMIAQYTSALLAGTITSGWTFGAGAALQIPFAMQM
Beta_B.1.351/1-1270 GLTVLPPLLTDEMIAQYTSALLAGTITSGWTFGAGAALQIPFAMQM
Delta_B.1.617.2_spike/1-1271 GLTVLPPLLTDEMIAQYTSALLAGTITSGWTFGAGAALQIPFAMQM
Gamma_P.1_spike/1-1273 GLTVLPPLLTDEMIAQYTSALLAGTITSGWTFGAGAALQIPFAMQM
Omicron_B.1.1.529_spike/1-1273 GLTVLPPLLTDEMIAQYTSALLAGTITSGWTFGAGAALQIPFAMQM
Omicron_BA.1_spike/1-1270 GLTVLPPLLTDEMIAQYTSALLAGTITSGWTFGAGAALQIPFAMQM
Omicron_BA.1.1_spike/1-1270 GLTVLPPLLTDEMIAQYTSALLAGTITSGWTFGAGAALQIPFAMQM
Omicron_BA.2_spike/1-1270 GLTVLPPLLTDEMIAQYTSALLAGTITSGWTFGAGAALQIPFAMQM
Omicron_BA.2.12.1_spike/1-1270 GLTVLPPLLTDEMIAQYTSALLAGTITSGWTFGAGAALQIPFAMQM
Omicron_BA.2.75_spike/1-1269 GLTVLPPLLTDEMIAQYTSALLAGTITSGWTFGAGAALQIPFAMQM
Omicron_BA.2.75.2_spike/1-1270 GLTVLPPLLTDEMIAQYTSALLAGTITSGWTFGAGAALQIPFAMQM
Omicron_BA.4_spike/1-1268 GLTVLPPLLTDEMIAQYTSALLAGTITSGWTFGAGAALQIPFAMQM
Omicron_BA.4.6_spike/1-1268 GLTVLPPLLTDEMIAQYTSALLAGTITSGWTFGAGAALQIPFAMQM
Omicron_BA.5_spike/1-1268 GLTVLPPLLTDEMIAQYTSALLAGTITSGWTFGAGAALQIPFAMQM
Omicron_BA.5.2.6_spike/1-1268 GLTVLPPLLTDEMIAQYTSALLAGTITSGWTFGAGAALQIPFAMQM
Omicron_BF.11_spike/1-1268 GLTVLPPLLTDEMIAQYTSALLAGTITSGWTFGAGAALQIPFAMQM
Omicron_BF.7_spike/1-1268 GLTVLPPLLTDEMIAQYTSALLAGTITSGWTFGAGAALQIPFAMQM
Omicron_BN.1_spike/1-1270 GLTVLPPLLTDEMIAQYTSALLAGTITSGWTFGAGAALQIPFAMQM
Omicron_BQ.1_spike/1-1267 GLTVLPPLLTDEMIAQYTSALLAGTITSGWTFGAGAALQIPFAMQM
Omicron_BQ.1.1_spike/1-1267 GLTVLPPLLTDEMIAQYTSALLAGTITSGWTFGAGAALQIPFAMQM
Omicron_CH.1.1_spike/1-1270 GLTVLPPLLTDEMIAQYTSALLAGTITSGWTFGAGAALQIPFAMQM
Omicron_XBB_spike/1-1269 GLTVLPPLLTDEMIAQYTSALLAGTITSGWTFGAGAALQIPFAMQM
Omicron_XBB.1.5_spike/1-1269 GLTVLPPLLTDEMIAQYTSALLAGTITSGWTFGAGAALQIPFAMQM
Conservation
*77*****9757*5*6**88*9887786***7*788776****889
Quality
Consensus
GLTVLPPLLTDEMIAQYTSALLAGTITSGWTFGAGAALQIPFAMQM
Occupancy

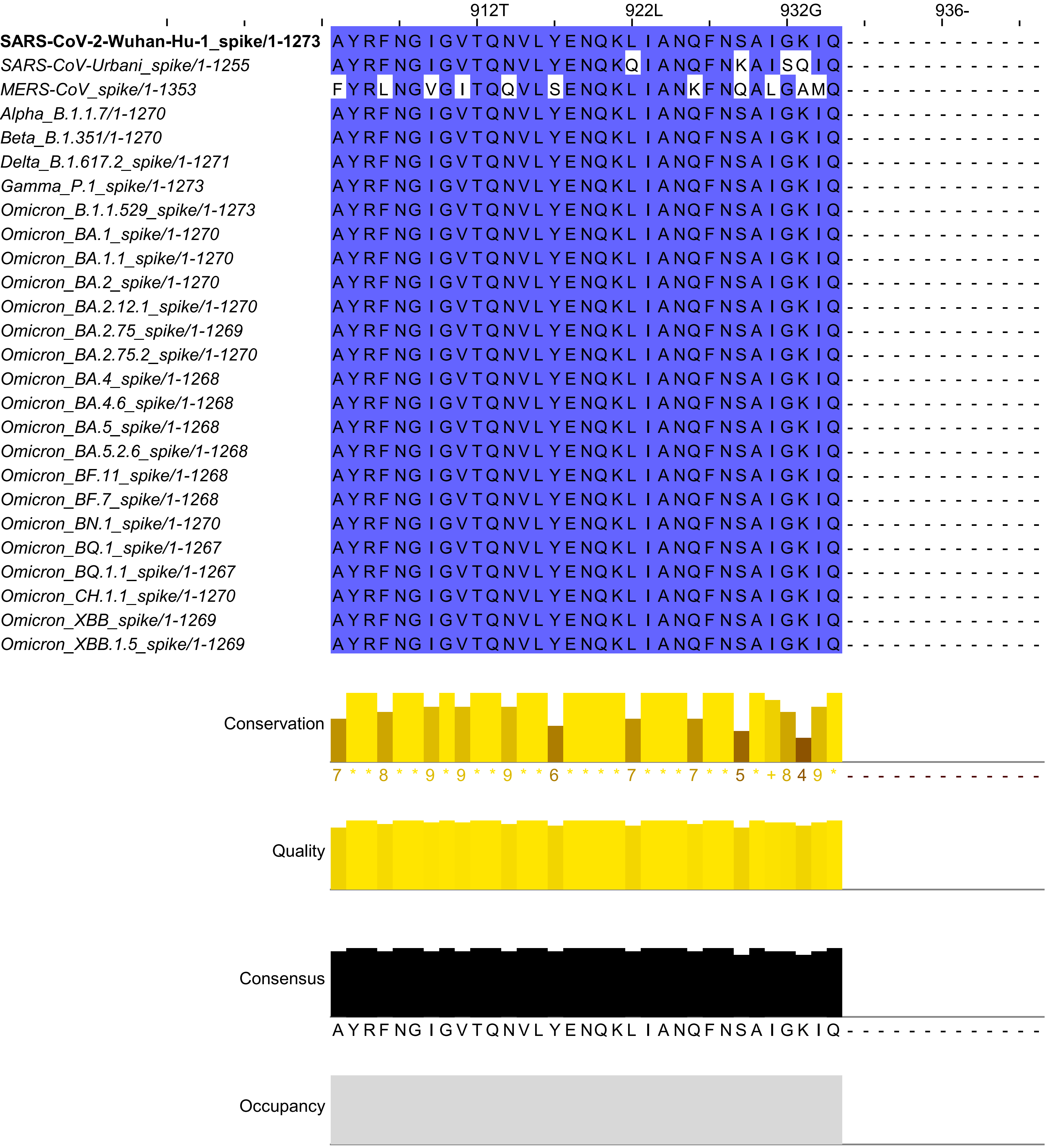
912T
922L
932G
936-
SARS-CoV-2-Wuhan-Hu-1_spike/1-1273 AYRFNGIGVTQNVLYENQKLIANQFNSAIGKIQ
SARS-CoV-Urbani_spike/1-1255 AYRFNGIGVTQNVLYENQKQIANQFNKAISQIQ
MERS-CoV_spike/1-1353 FYRLNGVGITQQVLSENQKLIANKFNQALGAMQ
Alpha_B.1.1.7/1-1270 AYRFNGIGVTQNVLYENQKLIANQFNSAIGKIQ
Beta_B.1.351/1-1270 AYRFNGIGVTQNVLYENQKLIANQFNSAIGKIQ
Delta_B.1.617.2_spike/1-1271 AYRFNGIGVTQNVLYENQKLIANQFNSAIGKIQ
Gamma_P.1_spike/1-1273 AYRFNGIGVTQNVLYENQKLIANQFNSAIGKIQ
Omicron_B.1.1.529_spike/1-1273 AYRFNGIGVTQNVLYENQKLIANQFNSAIGKIQ
Omicron_BA.1_spike/1-1270 AYRFNGIGVTQNVLYENQKLIANQFNSAIGKIQ
Omicron_BA.1.1_spike/1-1270 AYRFNGIGVTQNVLYENQKLIANQFNSAIGKIQ
Omicron_BA.2_spike/1-1270 AYRFNGIGVTQNVLYENQKLIANQFNSAIGKIQ
Omicron_BA.2.12.1_spike/1-1270 AYRFNGIGVTQNVLYENQKLIANQFNSAIGKIQ
Omicron_BA.2.75_spike/1-1269 AYRFNGIGVTQNVLYENQKLIANQFNSAIGKIQ
Omicron_BA.2.75.2_spike/1-1270 AYRFNGIGVTQNVLYENQKLIANQFNSAIGKIQ
Omicron_BA.4_spike/1-1268 AYRFNGIGVTQNVLYENQKLIANQFNSAIGKIQ
Omicron_BA.4.6_spike/1-1268 AYRFNGIGVTQNVLYENQKLIANQFNSAIGKIQ
Omicron_BA.5_spike/1-1268 AYRFNGIGVTQNVLYENQKLIANQFNSAIGKIQ
Omicron_BA.5.2.6_spike/1-1268 AYRFNGIGVTQNVLYENQKLIANQFNSAIGKIQ
Omicron_BF.11_spike/1-1268 AYRFNGIGVTQNVLYENQKLIANQFNSAIGKIQ
Omicron_BF.7_spike/1-1268 AYRFNGIGVTQNVLYENQKLIANQFNSAIGKIQ
Omicron_BN.1_spike/1-1270 AYRFNGIGVTQNVLYENQKLIANQFNSAIGKIQ
Omicron_BQ.1_spike/1-1267 AYRFNGIGVTQNVLYENQKLIANQFNSAIGKIQ
Omicron_BQ.1.1_spike/1-1267 AYRFNGIGVTQNVLYENQKLIANQFNSAIGKIQ
Omicron_CH.1.1_spike/1-1270 AYRFNGIGVTQNVLYENQKLIANQFNSAIGKIQ
Omicron_XBB_spike/1-1269 AYRFNGIGVTQNVLYENQKLIANQFNSAIGKIQ
Omicron_XBB.1.5_spike/1-1269 AYRFNGIGVTQNVLYENQKLIANQFNSAIGKIQ
Conservation
7 * * 8 * * 9 * 9 * * 9 * * 6 * * * * 7 * * * 7 * * 5 * + 8 4 9 *
Quality
Consensus
AYRFNGIGVTQNVLYENQKLIANQFNSAIGKIQ
Occupancy

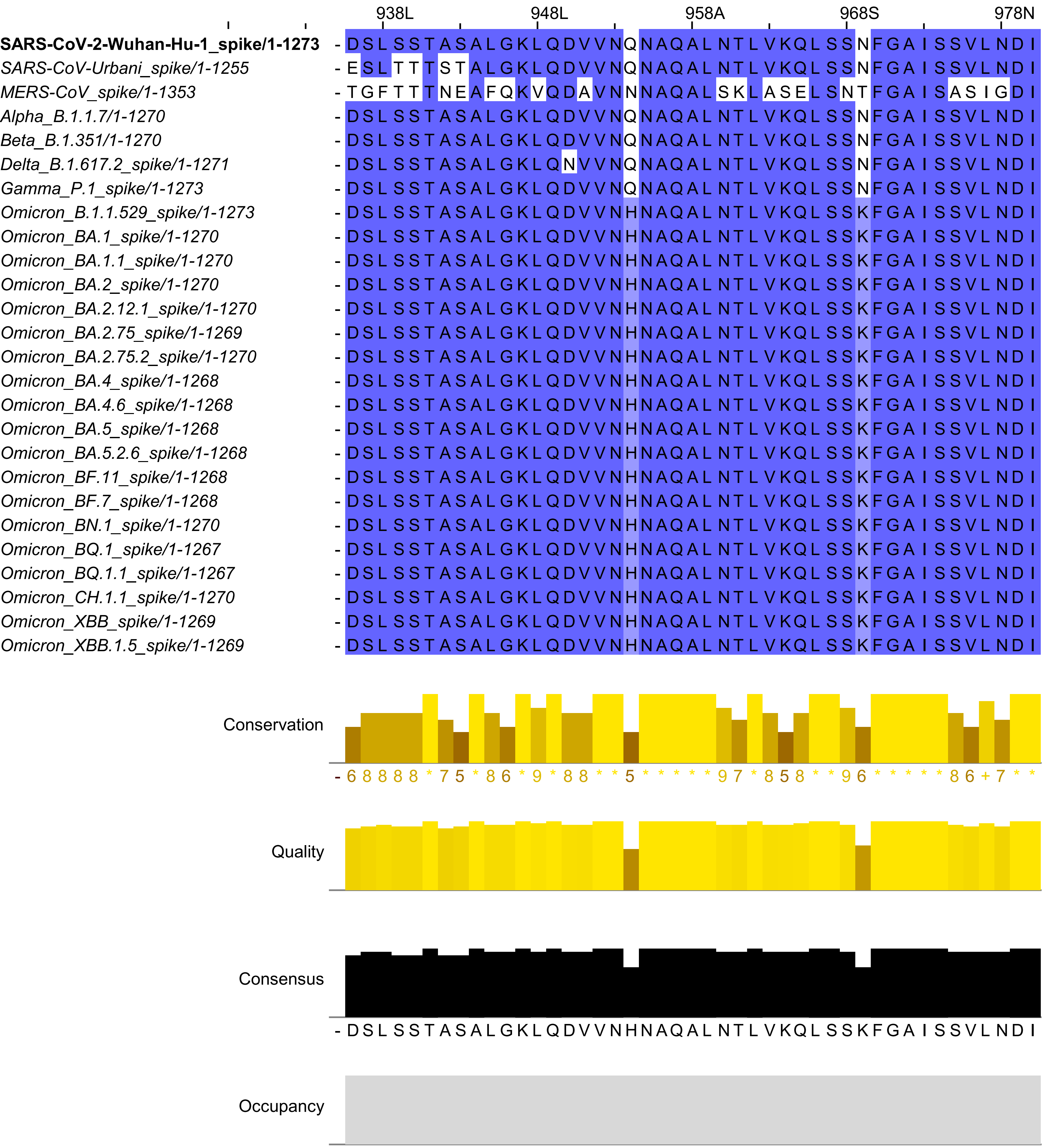
938L
948L
958A
968S
978N
SARS-CoV-2-Wuhan-Hu-1_spike/1-1273 - DSLSSTASALGKLQDVVNQNAQALNTLVKQLSSNFGAISSVLNDI
SARS-CoV-Urbani_spike/1-1255 - ESLTTTSTALGKLQDVVNQNAQALNTLVKQLSSNFGAISSVLNDI
MERS-CoV_spike/1-1353 - TGFTTTNEAFQKVQDAVNNNAQALSKLASELSNTFGAISASIGDI
Alpha_B.1.1.7/1-1270 - DSLSSTASALGKLQDVVNQNAQALNTLVKQLSSNFGAISSVLNDI
Beta_B.1.351/1-1270 - DSLSSTASALGKLQDVVNQNAQALNTLVKQLSSNFGAISSVLNDI
Delta_B.1.617.2_spike/1-1271 - DSLSSTASALGKLQNVVNQNAQALNTLVKQLSSNFGAISSVLNDI
Gamma_P.1_spike/1-1273 - DSLSSTASALGKLQDVVNQNAQALNTLVKQLSSNFGAISSVLNDI
Omicron_B.1.1.529_spike/1-1273 - DSLSSTASALGKLQDVVNHNAQALNTLVKQLSSKFGAISSVLNDI
Omicron_BA.1_spike/1-1270 - DSLSSTASALGKLQDVVNHNAQALNTLVKQLSSKFGAISSVLNDI
Omicron_BA.1.1_spike/1-1270 - DSLSSTASALGKLQDVVNHNAQALNTLVKQLSSKFGAISSVLNDI
Omicron_BA.2_spike/1-1270 - DSLSSTASALGKLQDVVNHNAQALNTLVKQLSSKFGAISSVLNDI
Omicron_BA.2.12.1_spike/1-1270 - DSLSSTASALGKLQDVVNHNAQALNTLVKQLSSKFGAISSVLNDI
Omicron_BA.2.75_spike/1-1269 - DSLSSTASALGKLQDVVNHNAQALNTLVKQLSSKFGAISSVLNDI
Omicron_BA.2.75.2_spike/1-1270 - DSLSSTASALGKLQDVVNHNAQALNTLVKQLSSKFGAISSVLNDI
Omicron_BA.4_spike/1-1268 - DSLSSTASALGKLQDVVNHNAQALNTLVKQLSSKFGAISSVLNDI
Omicron_BA.4.6_spike/1-1268 - DSLSSTASALGKLQDVVNHNAQALNTLVKQLSSKFGAISSVLNDI
Omicron_BA.5_spike/1-1268 - DSLSSTASALGKLQDVVNHNAQALNTLVKQLSSKFGAISSVLNDI
Omicron_BA.5.2.6_spike/1-1268 - DSLSSTASALGKLQDVVNHNAQALNTLVKQLSSKFGAISSVLNDI
Omicron_BF.11_spike/1-1268 - DSLSSTASALGKLQDVVNHNAQALNTLVKQLSSKFGAISSVLNDI
Omicron_BF.7_spike/1-1268 - DSLSSTASALGKLQDVVNHNAQALNTLVKQLSSKFGAISSVLNDI
Omicron_BN.1_spike/1-1270 - DSLSSTASALGKLQDVVNHNAQALNTLVKQLSSKFGAISSVLNDI
Omicron_BQ.1_spike/1-1267 - DSLSSTASALGKLQDVVNHNAQALNTLVKQLSSKFGAISSVLNDI
Omicron_BQ.1.1_spike/1-1267 - DSLSSTASALGKLQDVVNHNAQALNTLVKQLSSKFGAISSVLNDI
Omicron_CH.1.1_spike/1-1270 - DSLSSTASALGKLQDVVNHNAQALNTLVKQLSSKFGAISSVLNDI
Omicron_XBB_spike/1-1269 - DSLSSTASALGKLQDVVNHNAQALNTLVKQLSSKFGAISSVLNDI
Omicron_XBB.1.5_spike/1-1269 - DSLSSTASALGKLQDVVNHNAQALNTLVKQLSSKFGAISSVLNDI
Conservation
- 6 8 8 8 8 * 7 5 * 8 6 * 9 * 8 8 * * 5 * * * * * 9 7 * 8 5 8 * * 9 6 * * * * * 8 6 + 7 * *
Quality
Consensus
- DSLSSTASALGKLQDVVNHNAQALNTLVKQLSSKFGAISSVLNDI
Occupancy

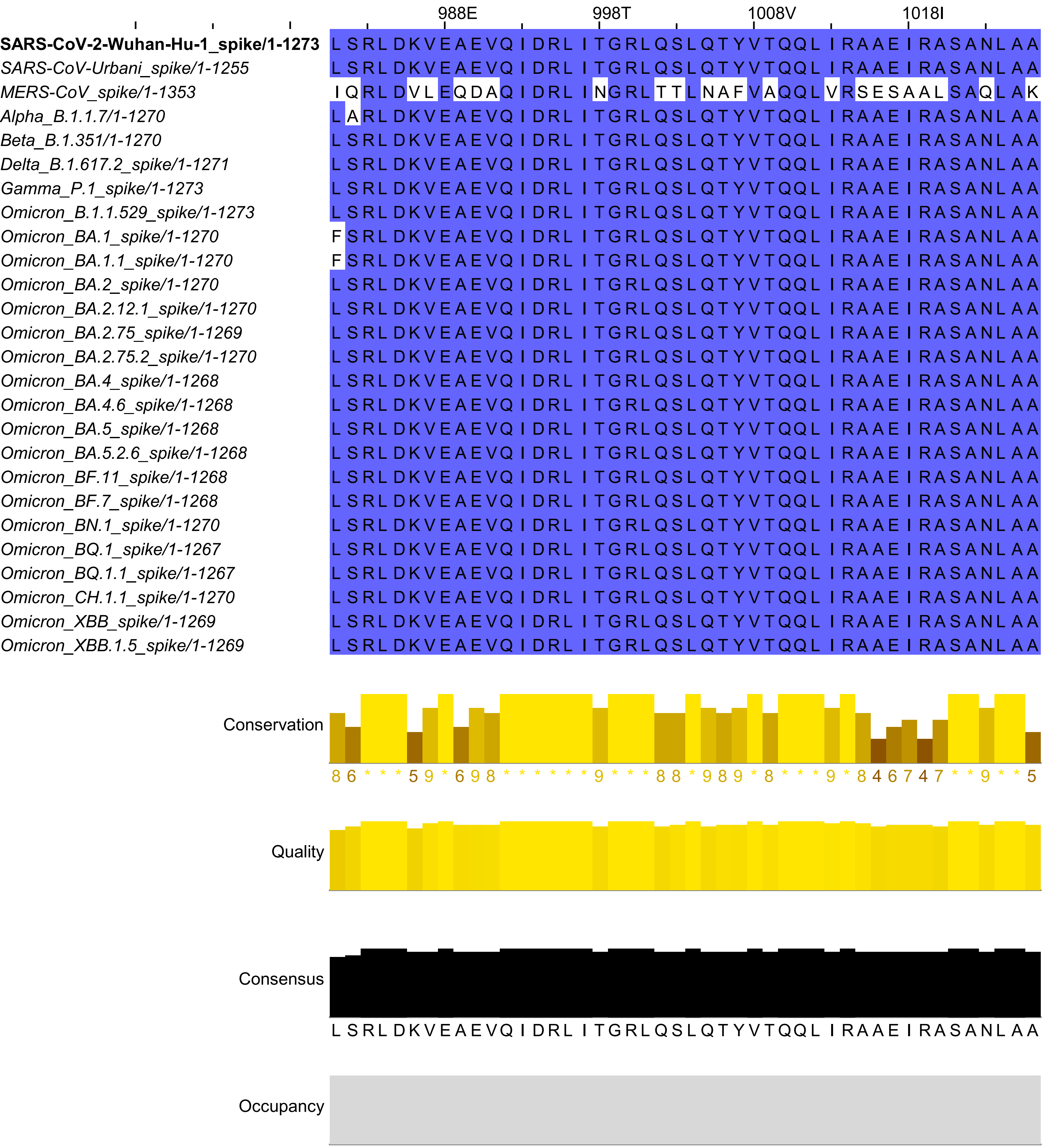
988E
998T
1008V
1018I
SARS-CoV-2-Wuhan-Hu-1_spike/1-1273 LSRLDKVEAEVQIDRLITGRLQSLQTYVTQQLIRAAEIRASANLAA
SARS-CoV-Urbani_spike/1-1255 LSRLDKVEAEVQIDRLITGRLQSLQTYVTQQLIRAAEIRASANLAA
MERS-CoV_spike/1-1353 IQRLDVLEQDAQIDRLINGRLTTLNAFVAQQLVRSESAALSAQLAK
Alpha_B.1.1.7/1-1270 LARLDKVEAEVQIDRLITGRLQSLQTYVTQQLIRAAEIRASANLAA
Beta_B.1.351/1-1270 LSRLDKVEAEVQIDRLITGRLQSLQTYVTQQLIRAAEIRASANLAA
Delta_B.1.617.2_spike/1-1271 LSRLDKVEAEVQIDRLITGRLQSLQTYVTQQLIRAAEIRASANLAA
Gamma_P.1_spike/1-1273 LSRLDKVEAEVQIDRLITGRLQSLQTYVTQQLIRAAEIRASANLAA
Omicron_B.1.1.529_spike/1-1273 LSRLDKVEAEVQIDRLITGRLQSLQTYVTQQLIRAAEIRASANLAA
Omicron_BA.1_spike/1-1270 FSRLDKVEAEVQIDRLITGRLQSLQTYVTQQLIRAAEIRASANLAA
Omicron_BA.1.1_spike/1-1270 FSRLDKVEAEVQIDRLITGRLQSLQTYVTQQLIRAAEIRASANLAA
Omicron_BA.2_spike/1-1270 LSRLDKVEAEVQIDRLITGRLQSLQTYVTQQLIRAAEIRASANLAA
Omicron_BA.2.12.1_spike/1-1270 LSRLDKVEAEVQIDRLITGRLQSLQTYVTQQLIRAAEIRASANLAA
Omicron_BA.2.75_spike/1-1269 LSRLDKVEAEVQIDRLITGRLQSLQTYVTQQLIRAAEIRASANLAA
Omicron_BA.2.75.2_spike/1-1270 LSRLDKVEAEVQIDRLITGRLQSLQTYVTQQLIRAAEIRASANLAA
Omicron_BA.4_spike/1-1268 LSRLDKVEAEVQIDRLITGRLQSLQTYVTQQLIRAAEIRASANLAA
Omicron_BA.4.6_spike/1-1268 LSRLDKVEAEVQIDRLITGRLQSLQTYVTQQLIRAAEIRASANLAA
Omicron_BA.5_spike/1-1268 LSRLDKVEAEVQIDRLITGRLQSLQTYVTQQLIRAAEIRASANLAA
Omicron_BA.5.2.6_spike/1-1268 LSRLDKVEAEVQIDRLITGRLQSLQTYVTQQLIRAAEIRASANLAA
Omicron_BF.11_spike/1-1268 LSRLDKVEAEVQIDRLITGRLQSLQTYVTQQLIRAAEIRASANLAA
Omicron_BF.7_spike/1-1268 LSRLDKVEAEVQIDRLITGRLQSLQTYVTQQLIRAAEIRASANLAA
Omicron_BN.1_spike/1-1270 LSRLDKVEAEVQIDRLITGRLQSLQTYVTQQLIRAAEIRASANLAA
Omicron_BQ.1_spike/1-1267 LSRLDKVEAEVQIDRLITGRLQSLQTYVTQQLIRAAEIRASANLAA
Omicron_BQ.1.1_spike/1-1267 LSRLDKVEAEVQIDRLITGRLQSLQTYVTQQLIRAAEIRASANLAA
Omicron_CH.1.1_spike/1-1270 LSRLDKVEAEVQIDRLITGRLQSLQTYVTQQLIRAAEIRASANLAA
Omicron_XBB_spike/1-1269 LSRLDKVEAEVQIDRLITGRLQSLQTYVTQQLIRAAEIRASANLAA
Omicron_XBB.1.5_spike/1-1269 LSRLDKVEAEVQIDRLITGRLQSLQTYVTQQLIRAAEIRASANLAA
Conservation
86***59*698******9***88*989*8***9*846747**9**5
Quality
Consensus
LSRLDKVEAEVQIDRLITGRLQSLQTYVTQQLIRAAEIRASANLAA
Occupancy

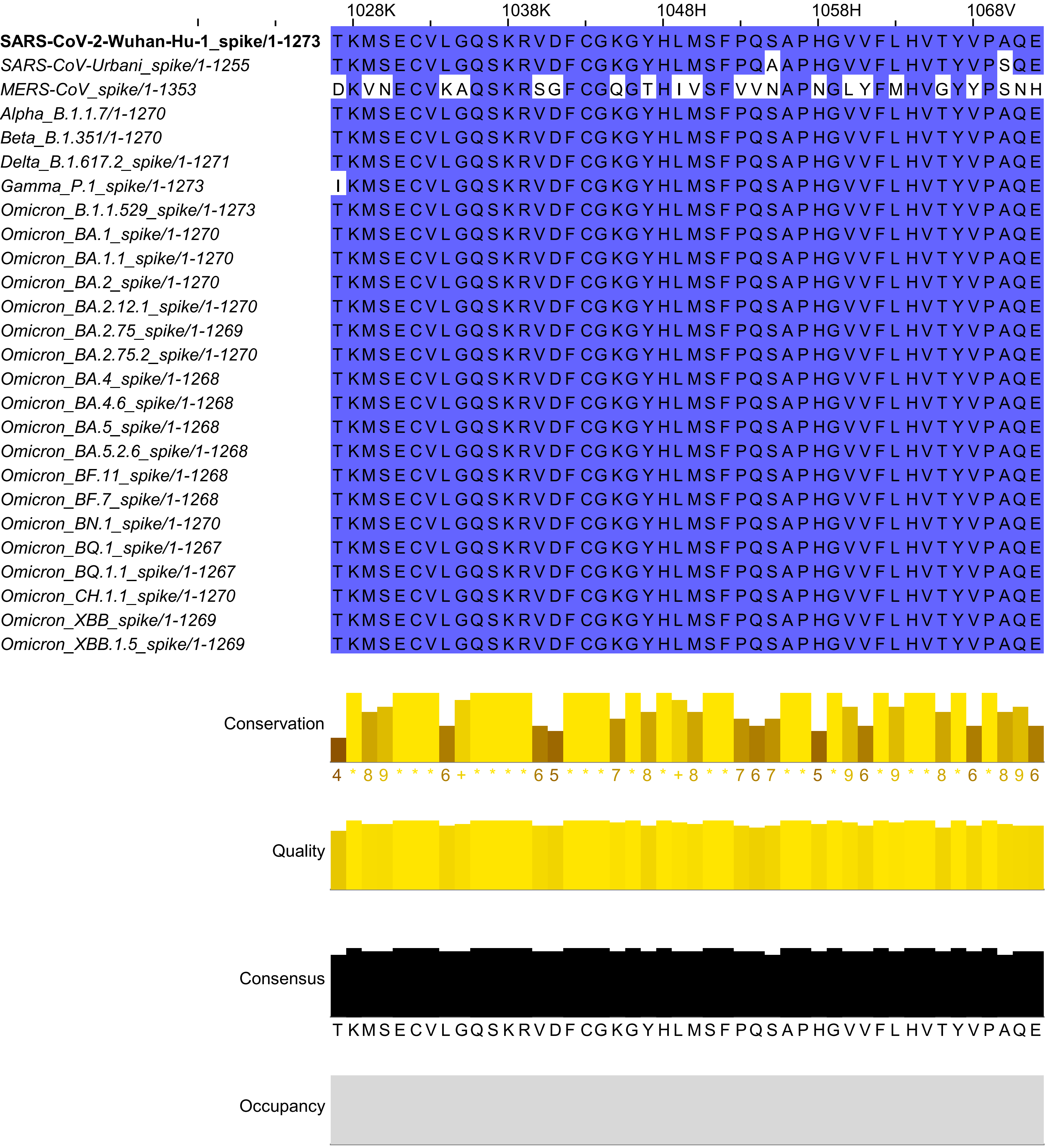

1028K 1038K 1048H 1058H 1068V
SARS-CoV-2-Wuhan-Hu-1_spike/1-1273 TKMSECVLGQSKRVDFCGKGYHLMSFPQSAPHGVVFLHVTYVPAQE
SARS-CoV-Urbani_spike/1-1255 TKMSECVLGQSKRVDFCGKGYHLMSFPQAAPHGVVFLHVTYVPSQE
MERS-CoV_spike/1-1353 DKVNECVKAQSKRSGFCGQGTHIVSFVVNAPNGLYFMHVGYYPSNH
Alpha_B.1.1.7/1-1270 TKMSECVLGQSKRVDFCGKGYHLMSFPQSAPHGVVFLHVTYVPAQE
Beta_B.1.351/1-1270 TKMSECVLGQSKRVDFCGKGYHLMSFPQSAPHGVVFLHVTYVPAQE
Delta_B.1.617.2_spike/1-1271 TKMSECVLGQSKRVDFCGKGYHLMSFPQSAPHGVVFLHVTYVPAQE
Gamma_P.1_spike/1-1273 IKMSECVLGQSKRVDFCGKGYHLMSFPQSAPHGVVFLHVTYVPAQE
Omicron_B.1.1.529_spike/1-1273 TKMSECVLGQSKRVDFCGKGYHLMSFPQSAPHGVVFLHVTYVPAQE
Omicron_BA.1_spike/1-1270 TKMSECVLGQSKRVDFCGKGYHLMSFPQSAPHGVVFLHVTYVPAQE
Omicron_BA.1.1_spike/1-1270 TKMSECVLGQSKRVDFCGKGYHLMSFPQSAPHGVVFLHVTYVPAQE
Omicron_BA.2_spike/1-1270 TKMSECVLGQSKRVDFCGKGYHLMSFPQSAPHGVVFLHVTYVPAQE
Omicron_BA.2.12.1_spike/1-1270 TKMSECVLGQSKRVDFCGKGYHLMSFPQSAPHGVVFLHVTYVPAQE
Omicron_BA.2.75_spike/1-1269 TKMSECVLGQSKRVDFCGKGYHLMSFPQSAPHGVVFLHVTYVPAQE
Omicron_BA.2.75.2_spike/1-1270 TKMSECVLGQSKRVDFCGKGYHLMSFPQSAPHGVVFLHVTYVPAQE
Omicron_BA.4_spike/1-1268 TKMSECVLGQSKRVDFCGKGYHLMSFPQSAPHGVVFLHVTYVPAQE
Omicron_BA.4.6_spike/1-1268 TKMSECVLGQSKRVDFCGKGYHLMSFPQSAPHGVVFLHVTYVPAQE
Omicron_BA.5_spike/1-1268 TKMSECVLGQSKRVDFCGKGYHLMSFPQSAPHGVVFLHVTYVPAQE
Omicron_BA.5.2.6_spike/1-1268 TKMSECVLGQSKRVDFCGKGYHLMSFPQSAPHGVVFLHVTYVPAQE
Omicron_BF.11_spike/1-1268 TKMSECVLGQSKRVDFCGKGYHLMSFPQSAPHGVVFLHVTYVPAQE
Omicron_BF.7_spike/1-1268 TKMSECVLGQSKRVDFCGKGYHLMSFPQSAPHGVVFLHVTYVPAQE
Omicron_BN.1_spike/1-1270 TKMSECVLGQSKRVDFCGKGYHLMSFPQSAPHGVVFLHVTYVPAQE
Omicron_BQ.1_spike/1-1267 TKMSECVLGQSKRVDFCGKGYHLMSFPQSAPHGVVFLHVTYVPAQE
Omicron_BQ.1.1_spike/1-1267 TKMSECVLGQSKRVDFCGKGYHLMSFPQSAPHGVVFLHVTYVPAQE
Omicron_CH.1.1_spike/1-1270 TKMSECVLGQSKRVDFCGKGYHLMSFPQSAPHGVVFLHVTYVPAQE
Omicron_XBB_spike/1-1269 TKMSECVLGQSKRVDFCGKGYHLMSFPQSAPHGVVFLHVTYVPAQE
Omicron_XBB.1.5_spike/1-1269 TKMSECVLGQSKRVDFCGKGYHLMSFPQSAPHGVVFLHVTYVPAQE
Conservation
4*89***6+****65***7*8*+8**767**5*96*9**8*6*896
Quality
Consensus
TKMSECVLGQSKRVDFCGKGYHLMSFPQSAPHGVVFLHVTYVPAQE
Occupancy

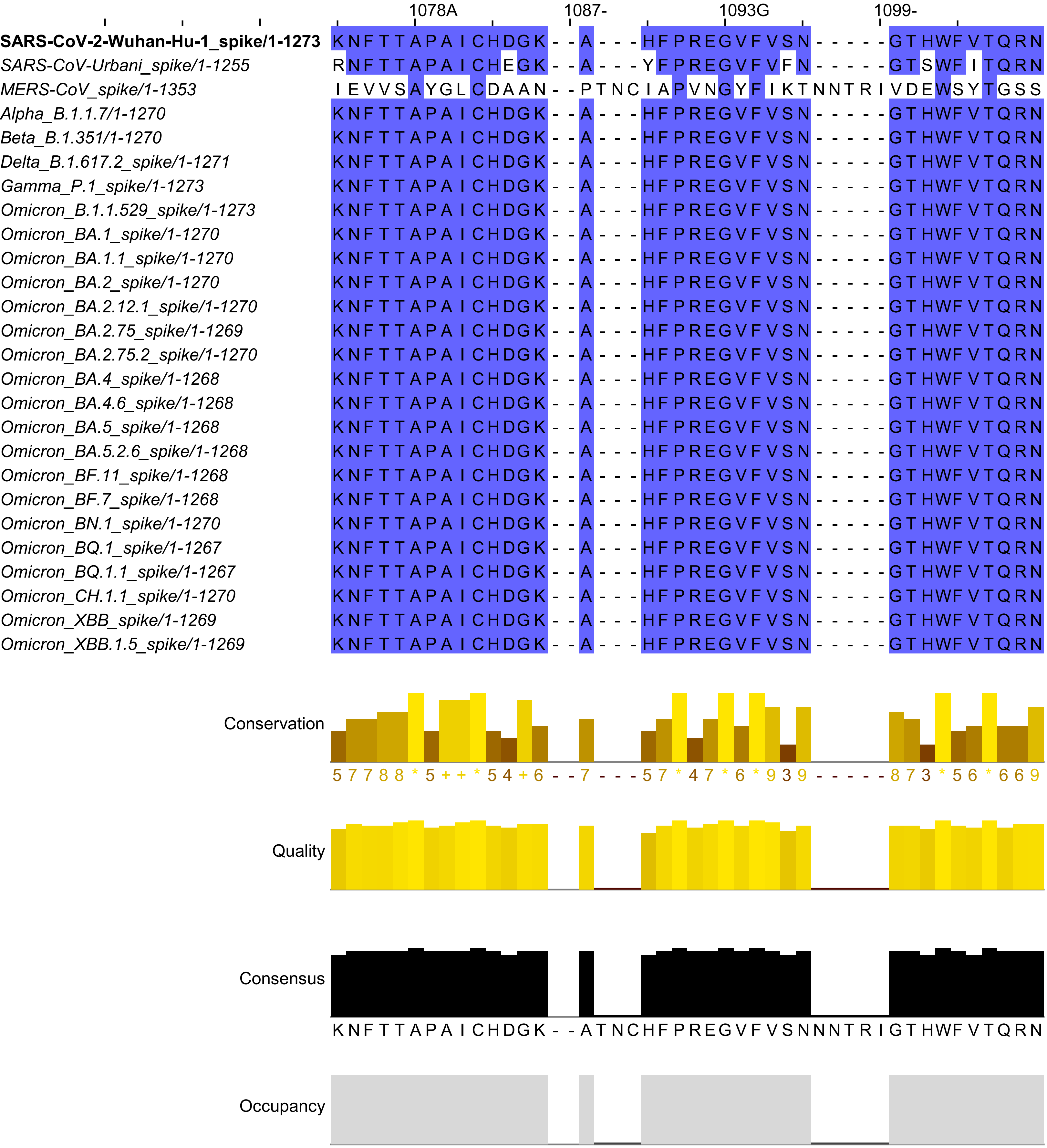
1078A
1087-
1093G
1099-
SARS-CoV-2-Wuhan-Hu-1_spike/1-1273 KNFTTAPAICHDGK--A---HFPREGVFVSN-----GTHWFVTQRN
SARS-CoV-Urbani_spike/1-1255 RNFTTAPAICHEGK--A---YFPREGVFVFN-----GTSWFITQRN
MERS-CoV_spike/1-1353 IEVVSAYGLCDAAN--PTNCIAPVNGYFIKTNNTRIVDEWSYTGSS
Alpha_B.1.1.7/1-1270 KNFTTAPAICHDGK--A---HFPREGVFVSN-----GTHWFVTQRN
Beta_B.1.351/1-1270 KNFTTAPAICHDGK--A---HFPREGVFVSN-----GTHWFVTQRN
Delta_B.1.617.2_spike/1-1271 KNFTTAPAICHDGK--A---HFPREGVFVSN-----GTHWFVTQRN
Gamma_P.1_spike/1-1273 KNFTTAPAICHDGK--A---HFPREGVFVSN-----GTHWFVTQRN
Omicron_B.1.1.529_spike/1-1273 KNFTTAPAICHDGK--A---HFPREGVFVSN-----GTHWFVTQRN
Omicron_BA.1_spike/1-1270 KNFTTAPAICHDGK--A---HFPREGVFVSN-----GTHWFVTQRN
Omicron_BA.1.1_spike/1-1270 KNFTTAPAICHDGK--A---HFPREGVFVSN-----GTHWFVTQRN
Omicron_BA.2_spike/1-1270 KNFTTAPAICHDGK--A---HFPREGVFVSN-----GTHWFVTQRN
Omicron_BA.2.12.1_spike/1-1270 KNFTTAPAICHDGK--A---HFPREGVFVSN-----GTHWFVTQRN
Omicron_BA.2.75_spike/1-1269 KNFTTAPAICHDGK--A---HFPREGVFVSN-----GTHWFVTQRN
Omicron_BA.2.75.2_spike/1-1270 KNFTTAPAICHDGK--A---HFPREGVFVSN-----GTHWFVTQRN
Omicron_BA.4_spike/1-1268 KNFTTAPAICHDGK--A---HFPREGVFVSN-----GTHWFVTQRN
Omicron_BA.4.6_spike/1-1268 KNFTTAPAICHDGK--A---HFPREGVFVSN-----GTHWFVTQRN
Omicron_BA.5_spike/1-1268 KNFTTAPAICHDGK--A---HFPREGVFVSN-----GTHWFVTQRN
Omicron_BA.5.2.6_spike/1-1268 KNFTTAPAICHDGK--A---HFPREGVFVSN-----GTHWFVTQRN
Omicron_BF.11_spike/1-1268 KNFTTAPAICHDGK--A---HFPREGVFVSN-----GTHWFVTQRN
Omicron_BF.7_spike/1-1268 KNFTTAPAICHDGK--A---HFPREGVFVSN-----GTHWFVTQRN
Omicron_BN.1_spike/1-1270 KNFTTAPAICHDGK--A---HFPREGVFVSN-----GTHWFVTQRN
Omicron_BQ.1_spike/1-1267 KNFTTAPAICHDGK--A---HFPREGVFVSN-----GTHWFVTQRN
Omicron_BQ.1.1_spike/1-1267 KNFTTAPAICHDGK--A---HFPREGVFVSN-----GTHWFVTQRN
Omicron_CH.1.1_spike/1-1270 KNFTTAPAICHDGK--A---HFPREGVFVSN-----GTHWFVTQRN
Omicron_XBB_spike/1-1269 KNFTTAPAICHDGK--A---HFPREGVFVSN-----GTHWFVTQRN
Omicron_XBB.1.5_spike/1-1269 KNFTTAPAICHDGK--A---HFPREGVFVSN-----GTHWFVTQRN
Conservation
57788*5++*54+6--7---57*47*6*939-----873*56*669
Quality
Consensus
KNFTTAPAICHDGK--ATNCHFPREGVFVSNNNTRIGTHWFVTQRN
Occupancy

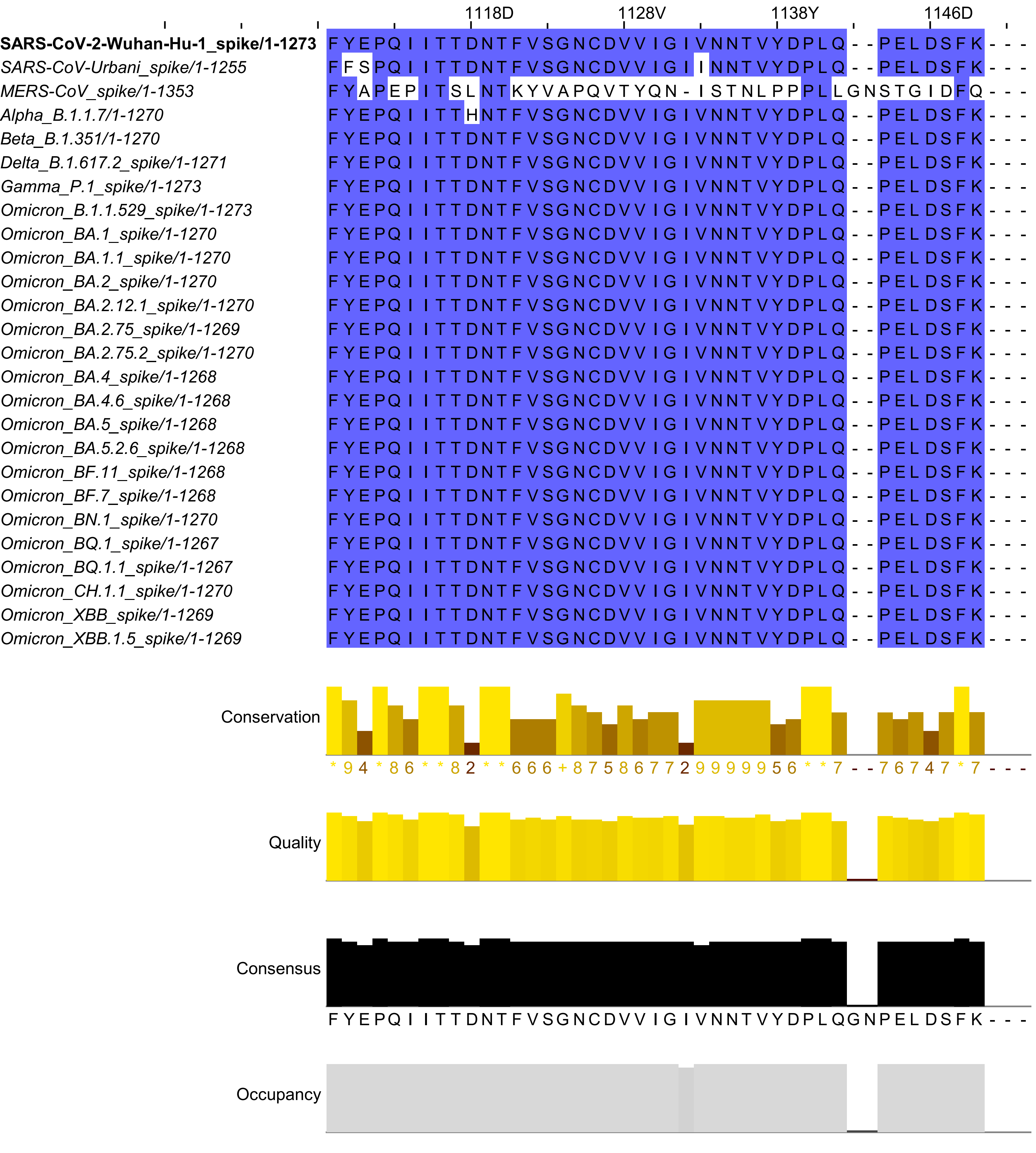

1118D
1128V
1138Y
1146D
SARS-CoV-2-Wuhan-Hu-1_spike/1-1273 FYEPQIITTDNTFVSGNCDVVIGIVNNTVYDPLQ--PELDSFK---
SARS-CoV-Urbani_spike/1-1255 FFSPQIITTDNTFVSGNCDVVIGIINNTVYDPLQ--PELDSFK---
MERS-CoV_spike/1-1353 FYAPEPITSLNTKYVAPQVTYQN-ISTNLPPPLLGNSTGIDFQ---
Alpha_B.1.1.7/1-1270 FYEPQIITTHNTFVSGNCDVVIGIVNNTVYDPLQ--PELDSFK---
Beta_B.1.351/1-1270 FYEPQIITTDNTFVSGNCDVVIGIVNNTVYDPLQ--PELDSFK---
Delta_B.1.617.2_spike/1-1271 FYEPQIITTDNTFVSGNCDVVIGIVNNTVYDPLQ--PELDSFK---
Gamma_P.1_spike/1-1273 FYEPQIITTDNTFVSGNCDVVIGIVNNTVYDPLQ--PELDSFK---
Omicron_B.1.1.529_spike/1-1273 FYEPQIITTDNTFVSGNCDVVIGIVNNTVYDPLQ--PELDSFK---
Omicron_BA.1_spike/1-1270 FYEPQIITTDNTFVSGNCDVVIGIVNNTVYDPLQ--PELDSFK---
Omicron_BA.1.1_spike/1-1270 FYEPQIITTDNTFVSGNCDVVIGIVNNTVYDPLQ--PELDSFK---
Omicron_BA.2_spike/1-1270 FYEPQIITTDNTFVSGNCDVVIGIVNNTVYDPLQ--PELDSFK---
Omicron_BA.2.12.1_spike/1-1270 FYEPQIITTDNTFVSGNCDVVIGIVNNTVYDPLQ--PELDSFK---
Omicron_BA.2.75_spike/1-1269 FYEPQIITTDNTFVSGNCDVVIGIVNNTVYDPLQ--PELDSFK---
Omicron_BA.2.75.2_spike/1-1270 FYEPQIITTDNTFVSGNCDVVIGIVNNTVYDPLQ--PELDSFK---
Omicron_BA.4_spike/1-1268 FYEPQIITTDNTFVSGNCDVVIGIVNNTVYDPLQ--PELDSFK---
Omicron_BA.4.6_spike/1-1268 FYEPQIITTDNTFVSGNCDVVIGIVNNTVYDPLQ--PELDSFK---
Omicron_BA.5_spike/1-1268 FYEPQIITTDNTFVSGNCDVVIGIVNNTVYDPLQ--PELDSFK---
Omicron_BA.5.2.6_spike/1-1268 FYEPQIITTDNTFVSGNCDVVIGIVNNTVYDPLQ--PELDSFK---
Omicron_BF.11_spike/1-1268 FYEPQIITTDNTFVSGNCDVVIGIVNNTVYDPLQ--PELDSFK---
Omicron_BF.7_spike/1-1268 FYEPQIITTDNTFVSGNCDVVIGIVNNTVYDPLQ--PELDSFK---
Omicron_BN.1_spike/1-1270 FYEPQIITTDNTFVSGNCDVVIGIVNNTVYDPLQ--PELDSFK---
Omicron_BQ.1_spike/1-1267 FYEPQIITTDNTFVSGNCDVVIGIVNNTVYDPLQ--PELDSFK---
Omicron_BQ.1.1_spike/1-1267 FYEPQIITTDNTFVSGNCDVVIGIVNNTVYDPLQ--PELDSFK---
Omicron_CH.1.1_spike/1-1270 FYEPQIITTDNTFVSGNCDVVIGIVNNTVYDPLQ--PELDSFK---
Omicron_XBB_spike/1-1269 FYEPQIITTDNTFVSGNCDVVIGIVNNTVYDPLQ--PELDSFK---
Omicron_XBB.1.5_spike/1-1269 FYEPQIITTDNTFVSGNCDVVIGIVNNTVYDPLQ--PELDSFK---
Conservation
*94*86**82**666+87586772999995 6**7--76747*7---
Quality
Consensus
FYEPQIITTDNTFVSGNCDVVIGIVNNTVYDPLQGNPELDSFK---
Occupancy

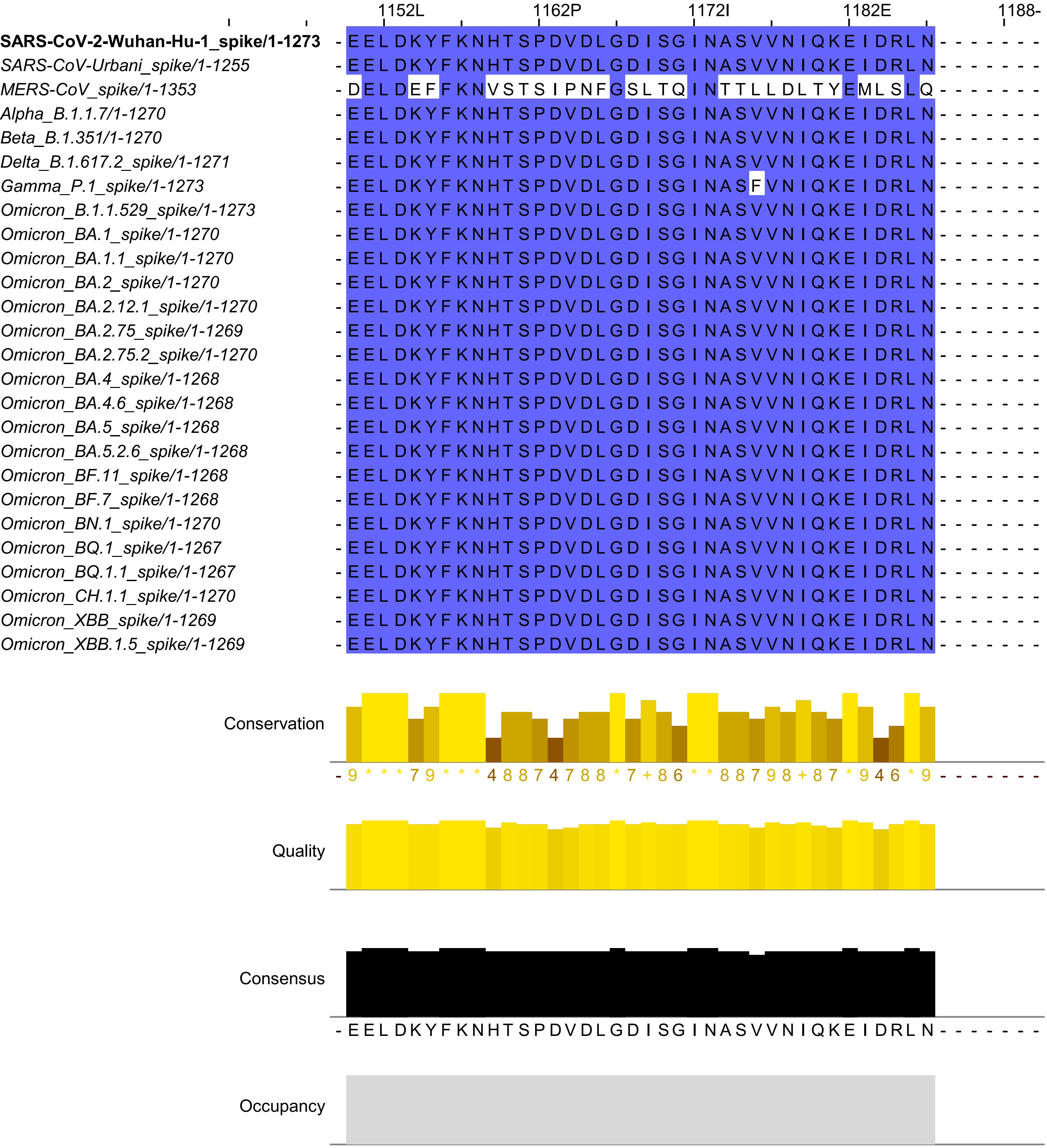

1152L
1162P
1172I
1182E
1188-
SARS-CoV-2-Wuhan-Hu-1_spike/1-1273
SARS-CoV-Urbani_spike/1-1255
MERS-CoV_spike/1-1353
Alpha_B.1.1.7/1-1270
Beta_B.1.351/1-1270
Delta_B.1.617.2_spike/1-1271
Gamma_P.1_spike/1-1273
Omicron_B.1.1.529_spike/1-1273
Omicron_BA.1_spike/1-1270
Omicron_BA.1.1_spike/1-1270
Omicron_BA.2_spike/1-1270
Omicron_BA.2.12.1_spike/1-1270
Omicron_BA.2.75_spike/1-1269
Omicron_BA.2.75.2_spike/1-1270
Omicron_BA.4_spike/1-1268
Omicron_BA.4.6_spike/1-1268
Omicron_BA.5_spike/1-1268
Omicron_BA.5.2.6_spike/1-1268
Omicron_BF.11_spike/1-1268
Omicron_BF.7_spike/1-1268
Omicron_BN.1_spike/1-1270
Omicron_BQ.1_spike/1-1267
Omicron_BQ.1.1_spike/1-1267
Omicron_CH.1.1_spike/1-1270
Omicron_XBB_spike/1-1269
Omicron_XBB.1.5_spike/1-1269
Conservation
Quality
Consensus
Occupancy

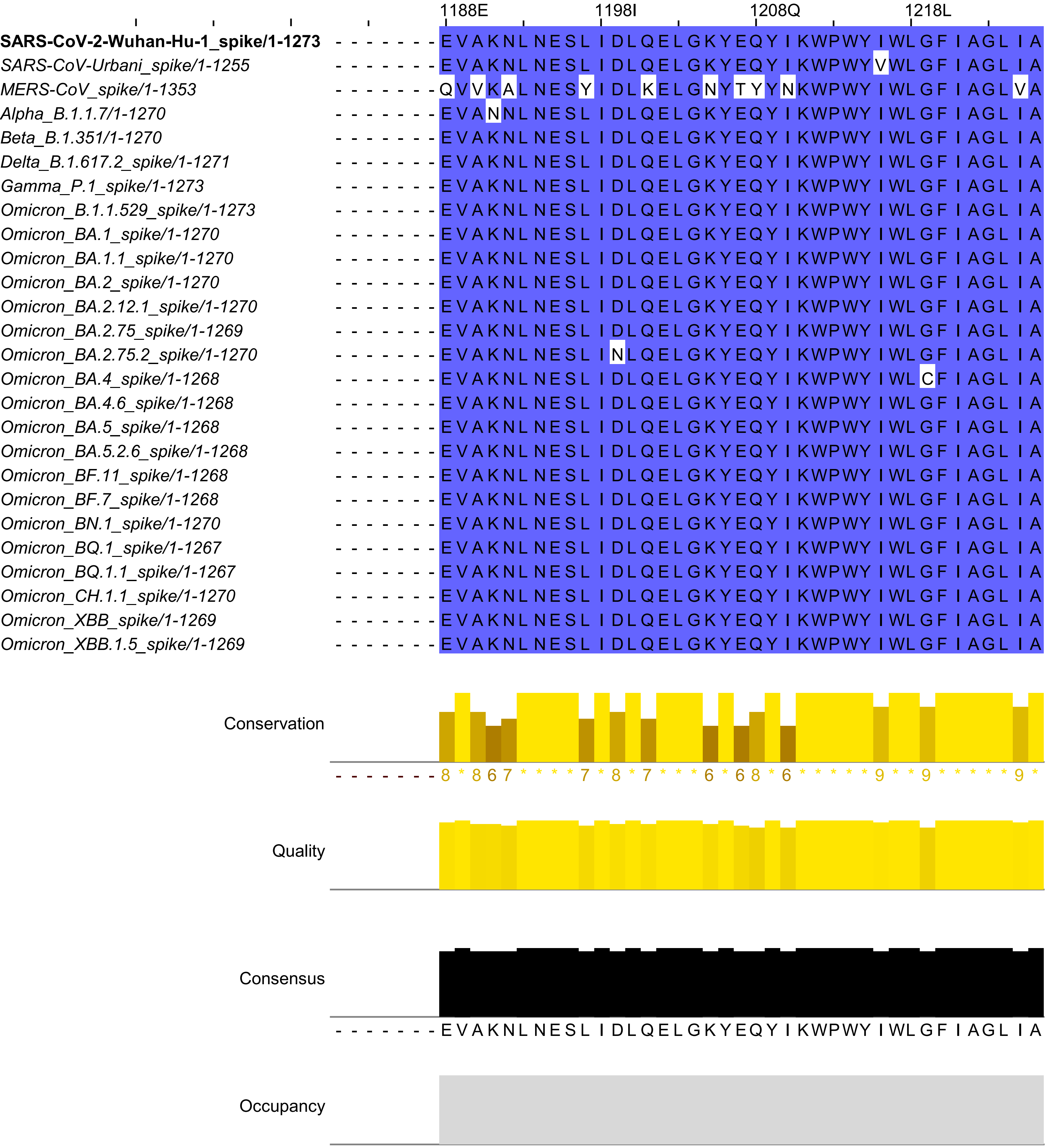
1188E
1198I
1208Q
1218L
SARS-CoV-2-Wuhan-Hu-1_spike/1-1273 -------EVAKNLNESLIDLQELGKYEQYIKWPWYIWLGFIAGLIA
SARS-CoV-Urbani_spike/1-1255 -------EVAKNLNESLIDLQELGKYEQYIKWPWYVWLGFIAGLIA
MERS-CoV_spike/1-1353 -------QVVKALNESYIDLKELGNYTYNKWPWYIWLGFIAGLVA
Alpha_B.1.1.7/1-1270 -------EVANNLNESLIDLQELGKYEQYIKWPWYIWLGFIAGLIA
Beta_B.1.351/1-1270 -------EVAKNLNESLIDLQELGKYEQYIKWPWYIWLGFIAGLIA
Delta_B.1.617.2_spike/1-1271 -------EVAKNLNESLIDLQELGKYEQYIKWPWYIWLGFIAGLIA
Gamma_P.1_spike/1-1273 -------EVAKNLNESLIDLQELGKYEQYIKWPWYIWLGFIAGLIA
Omicron_B.1.1.529_spike/1-1273 -------EVAKNLNESLIDLQELGKYEQYIKWPWYIWLGFIAGLIA
Omicron_BA.1_spike/1-1270 -------EVAKNLNESLIDLQELGKYEQYIKWPWYIWLGFIAGLIA
Omicron_BA.1.1_spike/1-1270 -------EVAKNLNESLIDLQELGKYEQYIKWPWYIWLGFIAGLIA
Omicron_BA.2_spike/1-1270 -------EVAKNLNESLIDLQELGKYEQYIKWPWYIWLGFIAGLIA
Omicron_BA.2.12.1_spike/1-1270 -------EVAKNLNESLIDLQELGKYEQYIKWPWYIWLGFIAGLIA
Omicron_BA.2.75_spike/1-1269 -------EVAKNLNESLIDLQELGKYEQYIKWPWYIWLGFIAGLIA
Omicron_BA.2.75.2_spike/1-1270 -------EVAKNLNESLINLQELGKYEQYIKWPWYIWLGFIAGLIA
Omicron_BA.4_spike/1-1268 -------EVAKNLNESLIDLQELGKYEQYIKWPWYIWLCFIAGLIA
Omicron_BA.4.6_spike/1-1268 -------EVAKNLNESLIDLQELGKYEQYIKWPWYIWLGFIAGLIA
Omicron_BA.5_spike/1-1268 -------EVAKNLNESLIDLQELGKYEQYIKWPWYIWLGFIAGLIA
Omicron_BA.5.2.6_spike/1-1268 -------EVAKNLNESLIDLQELGKYEQYIKWPWYIWLGFIAGLIA
Omicron_BF.11_spike/1-1268 -------EVAKNLNESLIDLQELGKYEQYIKWPWYIWLGFIAGLIA
Omicron_BF.7_spike/1-1268 -------EVAKNLNESLIDLQELGKYEQYIKWPWYIWLGFIAGLIA
Omicron_BN.1_spike/1-1270 -------EVAKNLNESLIDLQELGKYEQYIKWPWYIWLGFIAGLIA
Omicron_BQ.1_spike/1-1267 -------EVAKNLNESLIDLQELGKYEQYIKWPWYIWLGFIAGLIA
Omicron_BQ.1.1_spike/1-1267 -------EVAKNLNESLIDLQELGKYEQYIKWPWYIWLGFIAGLIA
Omicron_CH.1.1_spike/1-1270 -------EVAKNLNESLIDLQELGKYEQYIKWPWYIWLGFIAGLIA
Omicron_XBB_spike/1-1269 -------EVAKNLNESLIDLQELGKYEQYIKWPWYIWLGFIAGLIA
Omicron_XBB.1.5_spike/1-1269 -------EVAKNLNESLIDLQELGKYEQYIKWPWYIWLGFIAGLIA
Conservation
-------8*867****7*8*7***6*68*6*****9**9*****9*
Quality
Consensus
-------EVAKNLNESLIDLQELGKYEQYIKWPWYIWLGFIAGLIA
Occupancy

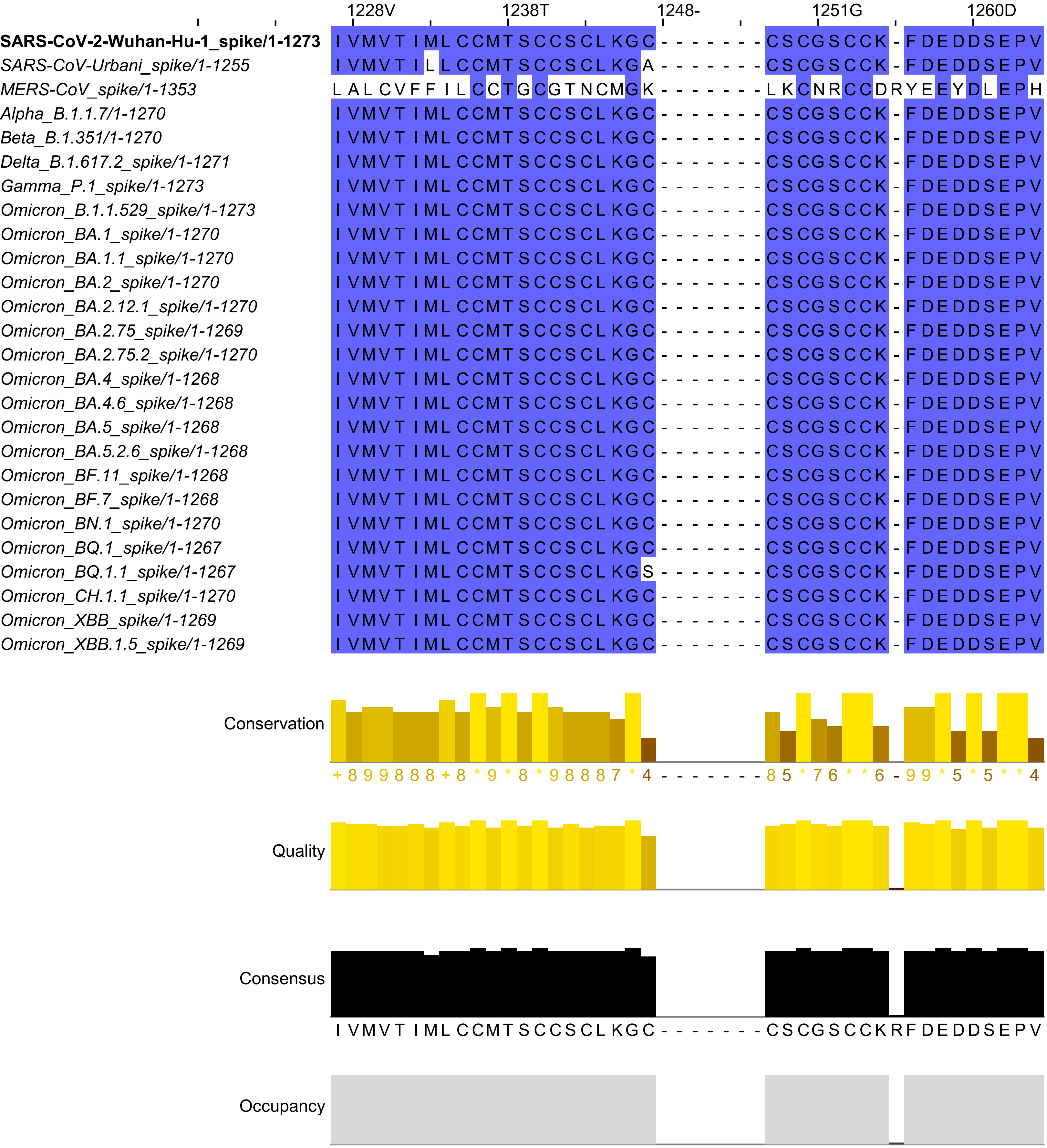
1228V
1238T
1248-
1251G
1260D
SARS-CoV-2-Wuhan-Hu-1_spike/1-1273 IVMVTIMLCCMTSCCSCLKGC-------CSCGSCCK-FDEDDSEPV
SARS-CoV-Urbani_spike/1-1255 IVMVTILLCCMTSCCSCLKGA-------CSCGSCCK-FDEDDSEPV
MERS-CoV_spike/1-1353 LALCVFFILCCTGCGTNCMGK-------LKCNRCCDRYEEYDLEPH
Alpha_B.1.1.7/1-1270 IVMVTIMLCCMTSCCSCLKGC-------CSCGSCCK-FDEDDSEPV
Beta_B.1.351/1-1270 IVMVTIMLCCMTSCCSCLKGC-------CSCGSCCK-FDEDDSEPV
Delta_B.1.617.2_spike/1-1271 IVMVTIMLCCMTSCCSCLKGC-------CSCGSCCK-FDEDDSEPV
Gamma_P.1_spike/1-1273 IVMVTIMLCCMTSCCSCLKGC-------CSCGSCCK-FDEDDSEPV
Omicron_B.1.1.529_spike/1-1273 IVMVTIMLCCMTSCCSCLKGC-------CSCGSCCK-FDEDDSEPV
Omicron_BA.1_spike/1-1270 IVMVTIMLCCMTSCCSCLKGC-------CSCGSCCK-FDEDDSEPV
Omicron_BA.1.1_spike/1-1270 IVMVTIMLCCMTSCCSCLKGC-------CSCGSCCK-FDEDDSEPV
Omicron_BA.2_spike/1-1270 IVMVTIMLCCMTSCCSCLKGC-------CSCGSCCK-FDEDDSEPV
Omicron_BA.2.12.1_spike/1-1270 IVMVTIMLCCMTSCCSCLKGC-------CSCGSCCK-FDEDDSEPV
Omicron_BA.2.75_spike/1-1269 IVMVTIMLCCMTSCCSCLKGC-------CSCGSCCK-FDEDDSEPV
Omicron_BA.2.75.2_spike/1-1270 IVMVTIMLCCMTSCCSCLKGC-------CSCGSCCK-FDEDDSEPV
Omicron_BA.4_spike/1-1268 IVMVTIMLCCMTSCCSCLKGC-------CSCGSCCK-FDEDDSEPV
Omicron_BA.4.6_spike/1-1268 IVMVTIMLCCMTSCCSCLKGC-------CSCGSCCK-FDEDDSEPV
Omicron_BA.5_spike/1-1268 IVMVTIMLCCMTSCCSCLKGC-------CSCGSCCK-FDEDDSEPV
Omicron_BA.5.2.6_spike/1-1268 IVMVTIMLCCMTSCCSCLKGC-------CSCGSCCK-FDEDDSEPV
Omicron_BF.11_spike/1-1268 IVMVTIMLCCMTSCCSCLKGC-------CSCGSCCK-FDEDDSEPV
Omicron_BF.7_spike/1-1268 IVMVTIMLCCMTSCCSCLKGC-------CSCGSCCK-FDEDDSEPV
Omicron_BN.1_spike/1-1270 IVMVTIMLCCMTSCCSCLKGC-------CSCGSCCK-FDEDDSEPV
Omicron_BQ.1_spike/1-1267 IVMVTIMLCCMTSCCSCLKGC-------CSCGSCCK-FDEDDSEPV
Omicron_BQ.1.1_spike/1-1267 IVMVTIMLCCMTSCCSCLKGS-------CSCGSCCK-FDEDDSEPV
Omicron_CH.1.1_spike/1-1270 IVMVTIMLCCMTSCCSCLKGC-------CSCGSCCK-FDEDDSEPV
Omicron_XBB_spike/1-1269 IVMVTIMLCCMTSCCSCLKGC-------CSCGSCCK-FDEDDSEPV
Omicron_XBB.1.5_spike/1-1269 IVMVTIMLCCMTSCCSCLKGC-------CSCGSCCK-FDEDDSEPV
Conservation
+899888+8*9*8*98887*4-------85*76**6-99*5*5**4
Quality
Consensus
IVMVTIMLCCMTSCCSCLKGC-------CSCGSCCKRFDEDDSEPV
Occupancy

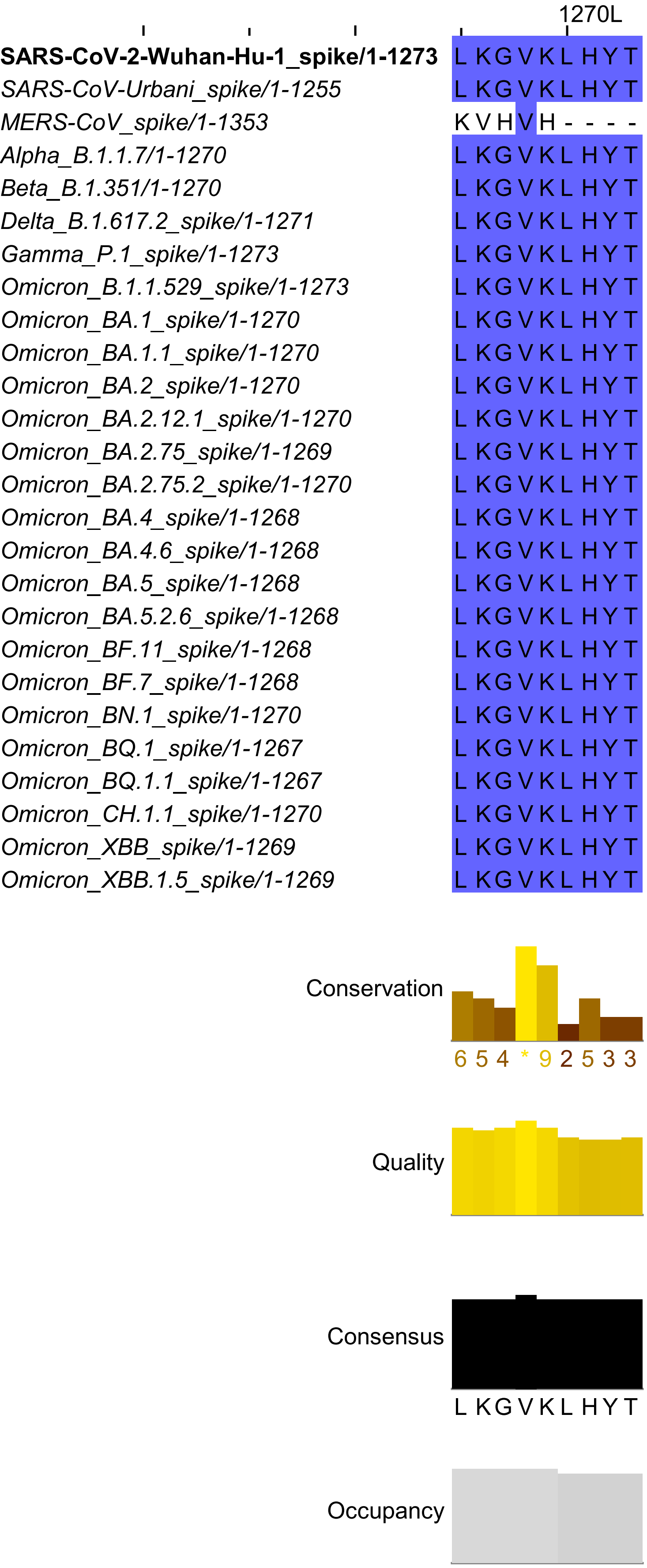
1270L
SARS-CoV-2-Wuhan-Hu-1_spike/1-1273 LKGVKLHYT
SARS-CoV-Urbani_spike/1-1255 LKGVKLHYT
MERS-CoV_spike/1-1353 KVHVH----
Alpha_B.1.1.7/1-1270 LKGVKLHYT
Beta_B.1.351/1-1270 LKGVKLHYT
Delta_B.1.617.2_spike/1-1271 LKGVKLHYT
Gamma_P.1_spike/1-1273 LKGVKLHYT
Omicron_B.1.1.529_spike/1-1273 LKGVKLHYT
Omicron_BA.1_spike/1-1270 LKGVKLHYT
Omicron_BA.1.1_spike/1-1270 LKGVKLHYT
Omicron_BA.2_spike/1-1270 LKGVKLHYT
Omicron_BA.2.12.1_spike/1-1270 LKGVKLHYT
Omicron_BA.2.75_spike/1-1269 LKGVKLHYT
Omicron_BA.2.75.2_spike/1-1270 LKGVKLHYT
Omicron_BA.4_spike/1-1268 LKGVKLHYT
Omicron_BA.4.6_spike/1-1268 LKGVKLHYT
Omicron_BA.5_spike/1-1268 LKGVKLHYT
Omicron_BA.5.2.6_spike/1-1268 LKGVKLHYT
Omicron_BF.11_spike/1-1268 LKGVKLHYT
Omicron_BF.7_spike/1-1268 LKGVKLHYT
Omicron_BN.1_spike/1-1270 LKGVKLHYT
Omicron_BQ.1_spike/1-1267 LKGVKLHYT
Omicron_BQ.1.1_spike/1-1267 LKGVKLHYT
Omicron_CH.1.1_spike/1-1270 LKGVKLHYT
Omicron_XBB_spike/1-1269 LKGVKLHYT
Omicron_XBB.1.5_spike/1-1269 LKGVKLHYT
Conservation
6 5 4 * 9 2 5 3 3
Quality
Consensus
LKGVKLHYT
Occupancy
