# Supplementary material for: An Immunoinformatic Approach for Identifying and Designing Conserved Multi-Epitope Vaccines for Coronaviruses
Source: Biomedicines. 2024 Nov 5;12(11):2530. doi: 10.3390/biomedicines12112530 (PMC11592158; doi:10.3390/biomedicines12112530)
Supplement: Supplementary file 1 [file biomedicines-12-02530-s001.zip › Supplementary Figure S4. Epitopes alignment.pdf]

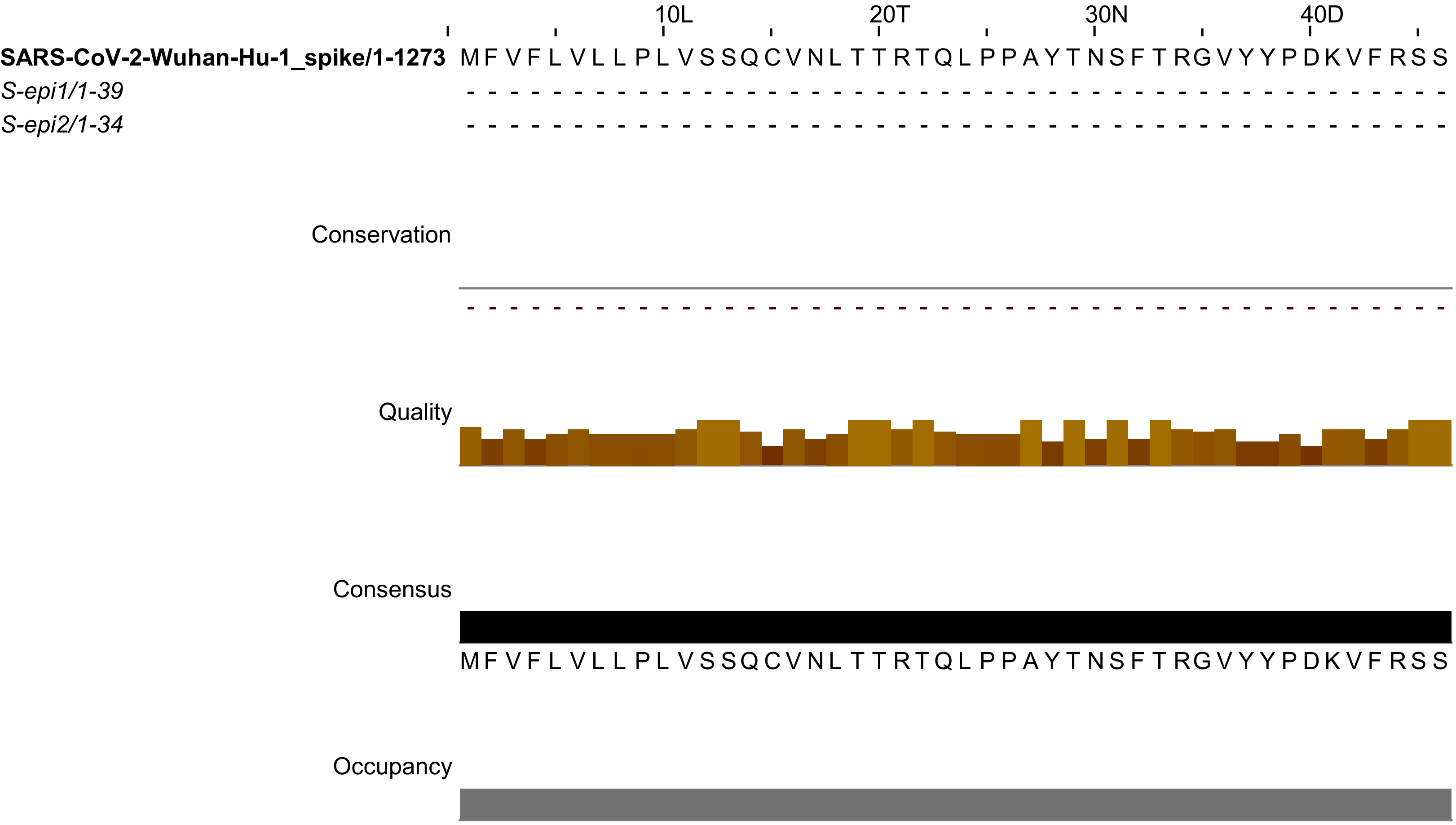
10L 20T 30N 40D
SARS-CoV-2-Wuhan-Hu-1_spike/1-1273 MFVFLVLLPLVSSQCVNLTTRTQLPPAYTNSFTRGVYYPDKVFRSS
S-epi1/1-39
S-epi2/1-34
Conservation
Quality
Consensus
MFVFLVLLPLVSSQCVNLTTRTQLPPAYTNSFTRGVYYPDKVFRSS
Occupancy

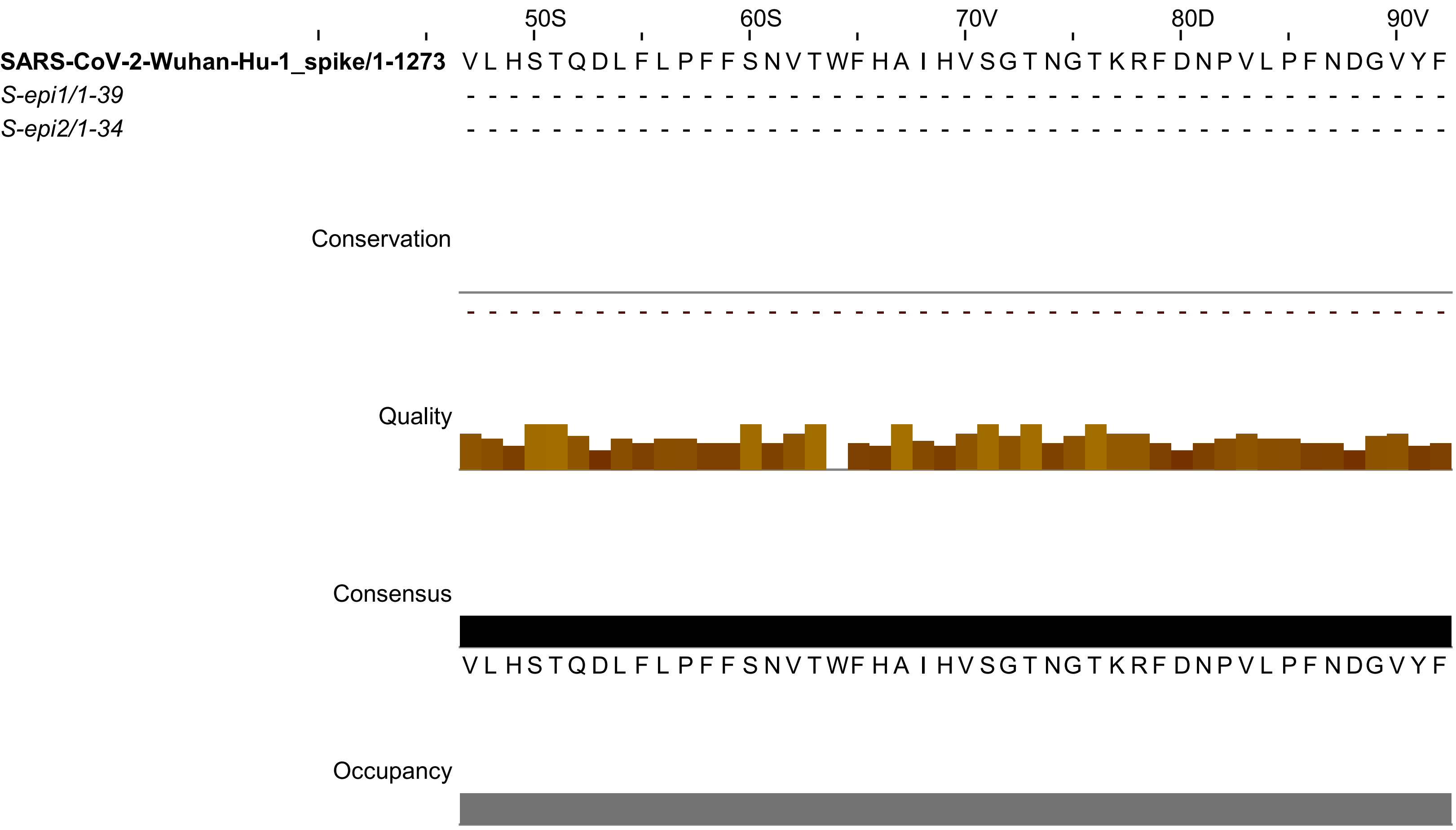

50S 60S 70V 80D 90V
SARS-CoV-2-Wuhan-Hu-1_spike/1-1273 VLHSTQDLFLPFFSNVTWFHAIHVSGTNGTKRFDNPVLPFNDGVYF
S-epi1/1-39
S-epi2/1-34
Conservation
Quality
Consensus
VLHSTQDLFLPFFSNVTWFHAIHVSGTNGTKRFDNPVLPFNDGVYF
Occupancy

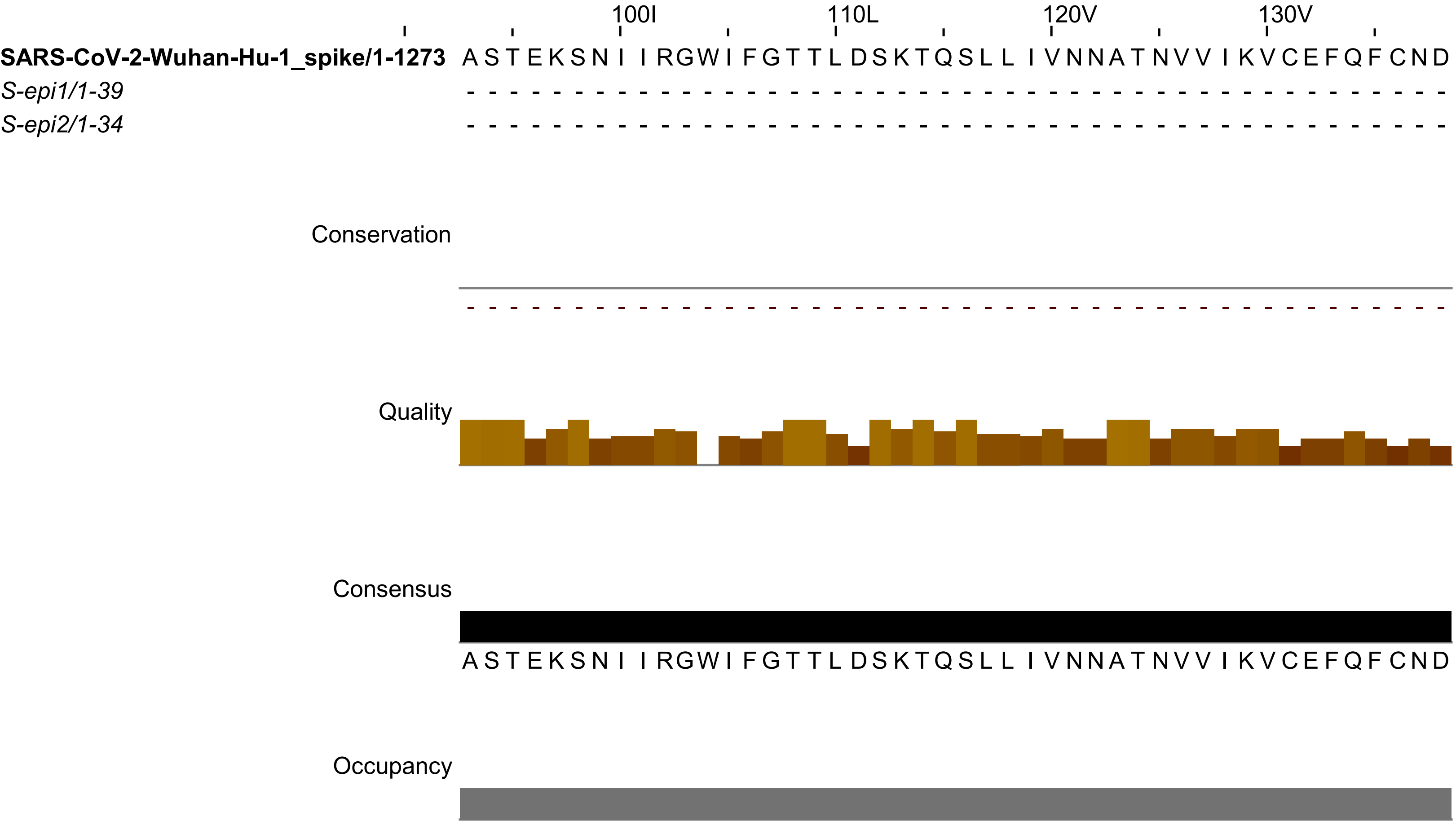
100I
110L
120V
130V
SARS-CoV-2-Wuhan-Hu-1_spike/1-1273
A S T E K S N I I R G W I F G T T L D S K T Q S L L I V N N A T N V V I K V C E F Q F C N D
S-epi1/1-39
S-epi2/1-34
Conservation
Quality
Consensus
A S T E K S N I I R G W I F G T T L D S K T Q S L L I V N N A T N V V I K V C E F Q F C N D
Occupancy

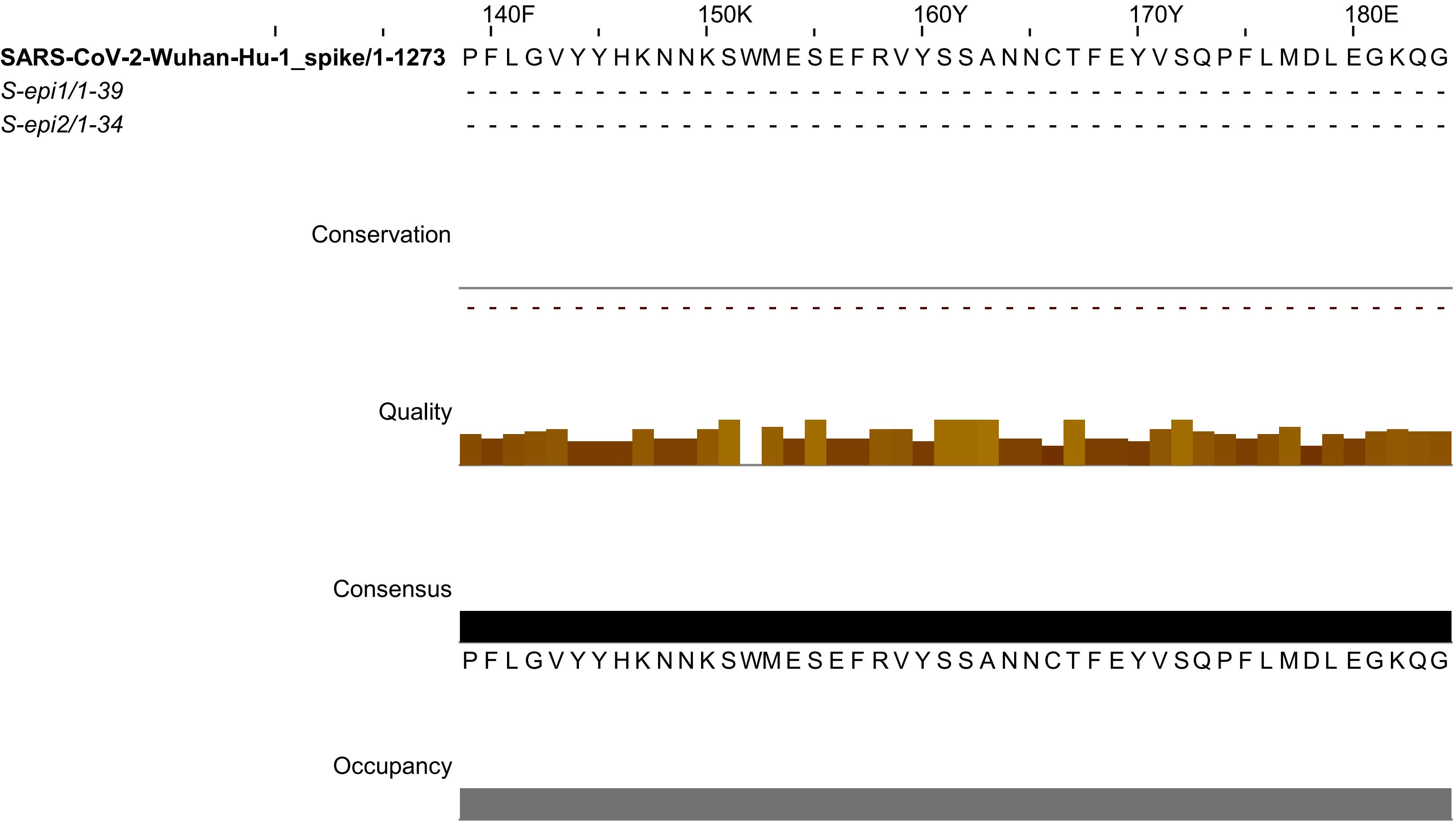
140F
150K
160Y
170Y
180E
SARS-CoV-2-Wuhan-Hu-1_spike/1-1273 PFLGVYYHKNNKSWMESEFRVYSSANNCTFEYVSQPFLMDLEGKQG
S-epi1/1-39
S-epi2/1-34
Conservation
Quality
Consensus
PFLGVYYHKNNKSWMESEFRVYSSANNCTFEYVSQPFLMDLEGKQG
Occupancy

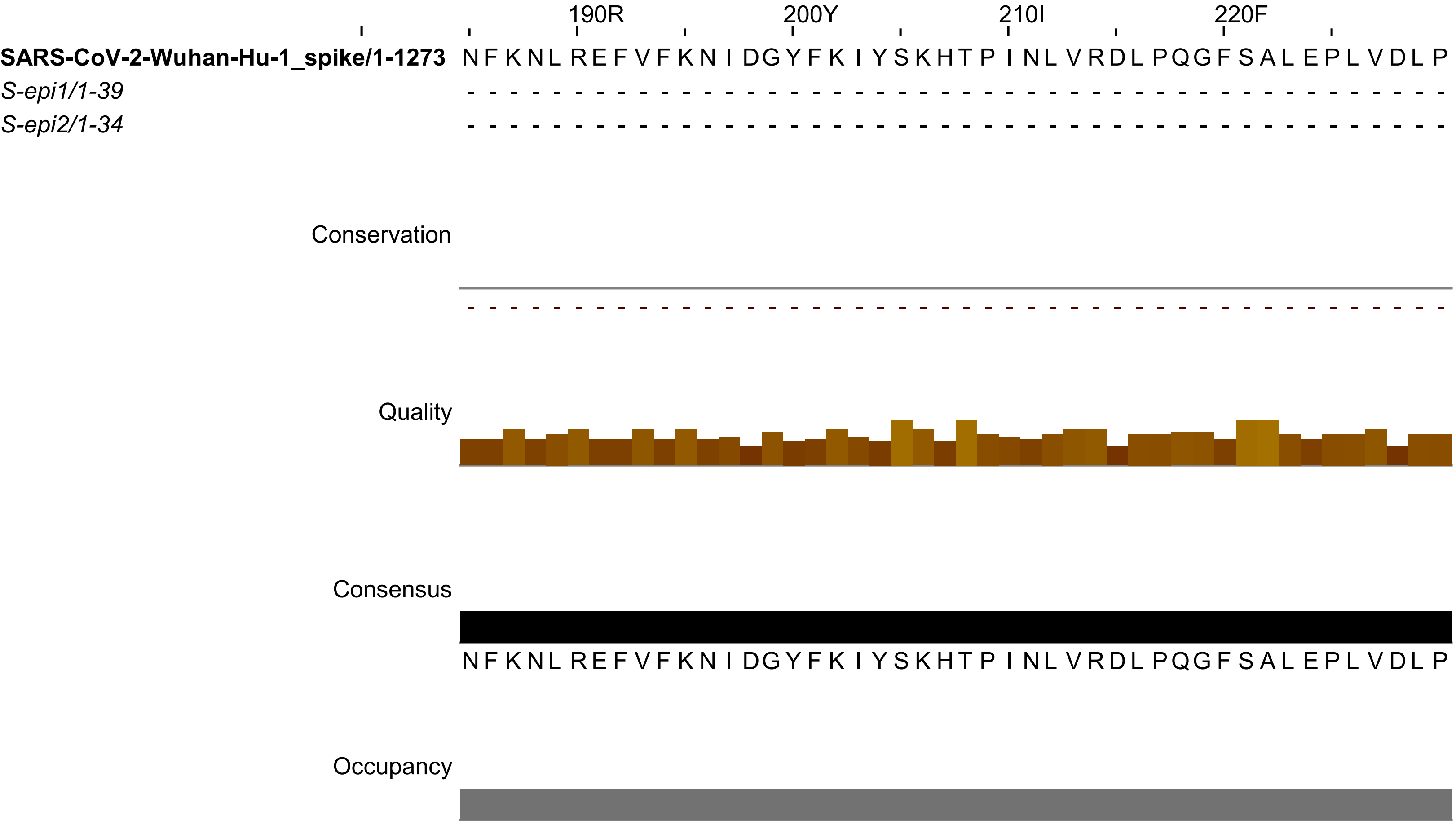
190R
200Y
210I
220F
SARS-CoV-2-Wuhan-Hu-1_spike/1-1273 NFKNLREFVFKNIDGYFKIYSKHTPINLVRDLPQGFSALEPLVDLP
S-epi1/1-39
S-epi2/1-34
Conservation
Quality
Consensus
NFKNLREFVFKNIDGYFKIYSKHTPINLVRDLPQGFSALEPLVDLP
Occupancy

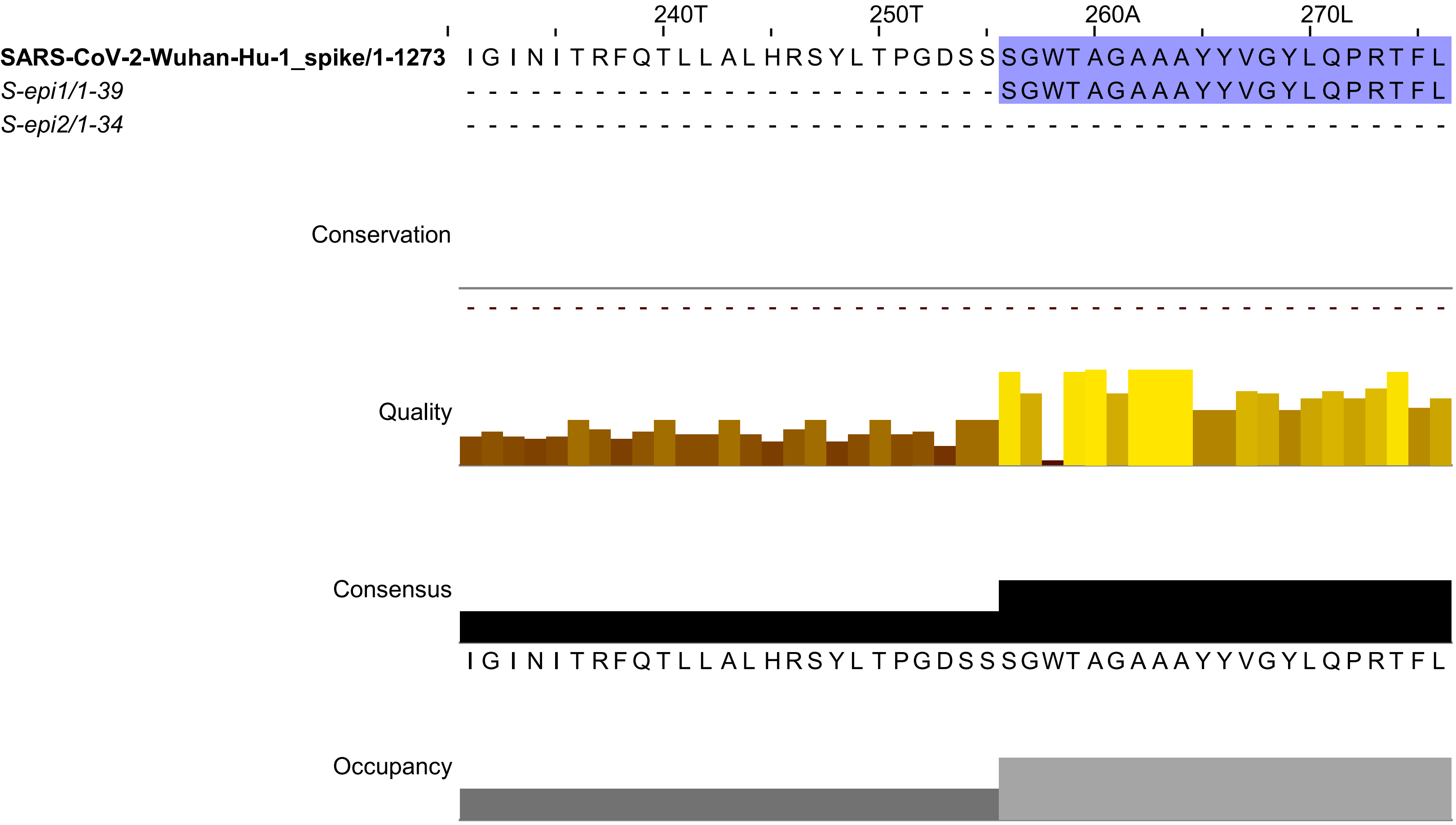
240T
250T
260A
270L
SARS-CoV-2-Wuhan-Hu-1_spike/1-1273 IGINITRFQTLLALHRSYLTPGDSSSGWTAGAAAYYVGYLQPRTFL
S-epi1/1-39 -------------------------SGWTAGAAAYYVGYLQPRTFL
S-epi2/1-34 --
Conservation
Quality
Consensus
IGINITRFQTLLALHRSYLTPGDSSSGWTAGAAAYYVGYLQPRTFL
Occupancy

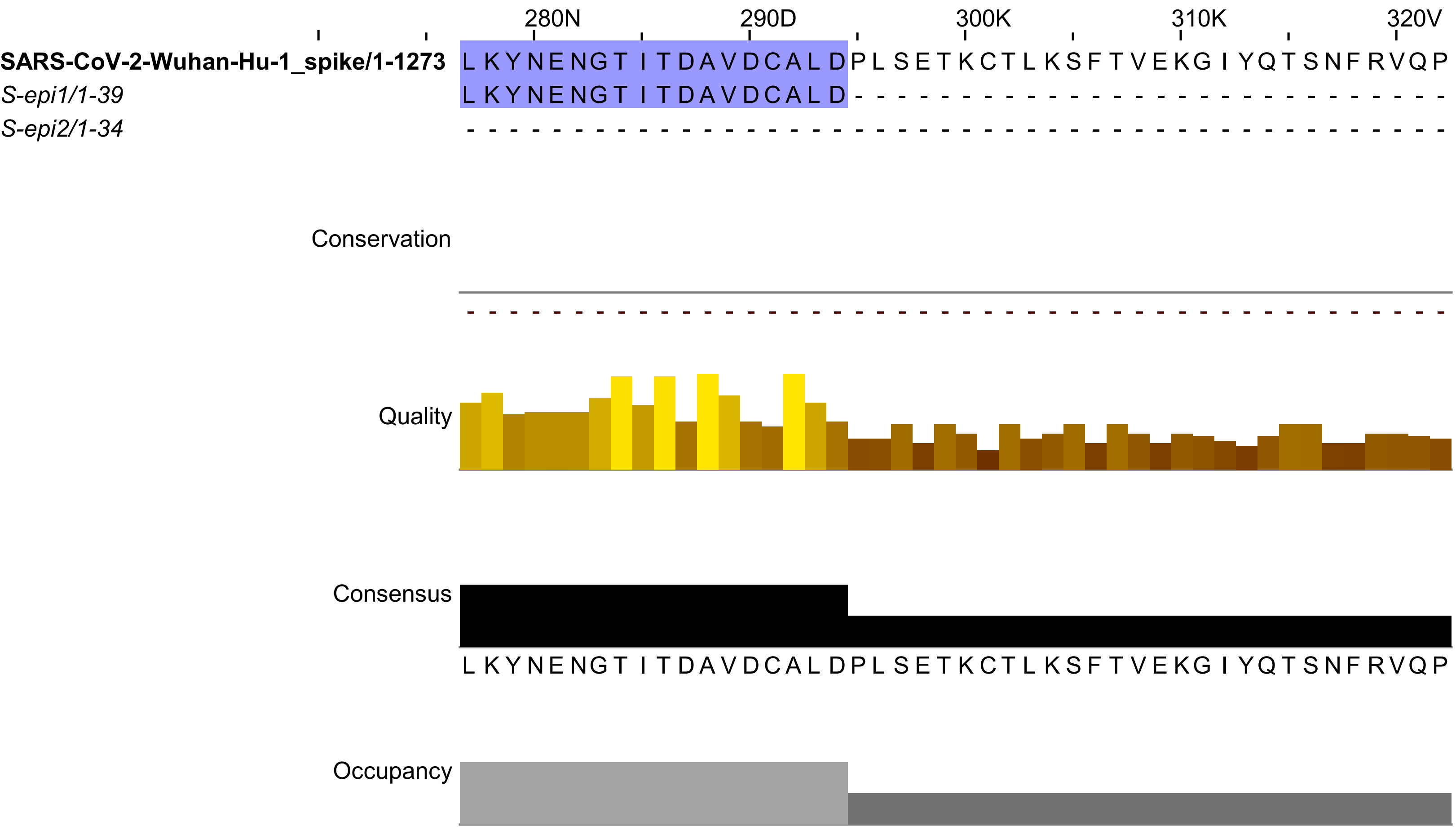
280N
290D
300K
310K
320V
SARS-CoV-2-Wuhan-Hu-1_spike/1-1273
LKYNENGTITDAVDCALDPLSETKCTLKSFTVEKGIYQTSNFRVQP
S-epi1/1-39
LKYNENGTITDAVDCALD
S-epi2/1-34
Conservation
Quality
Consensus
LKYNENGTITDAVDCALDPLSETKCTLKSFTVEKGIYQTSNFRVQP
Occupancy

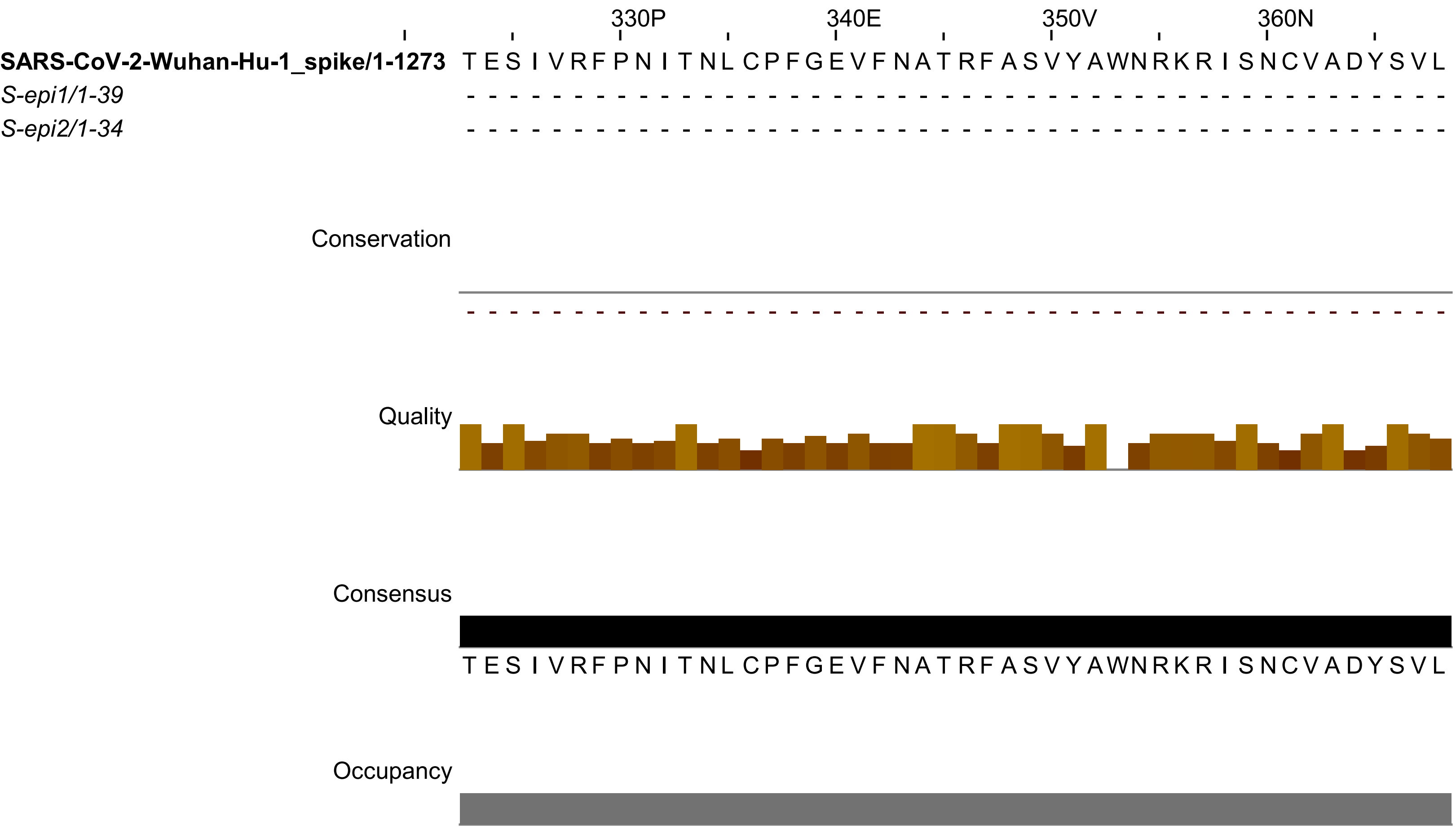
330P
340E
350V
360N
SARS-CoV-2-Wuhan-Hu-1_spike/1-1273 TESIVRFPNITNLCPFGEVFNATRFASVYAWNRKRISNCVADYSVL
S-epi1/1-39
S-epi2/1-34
Conservation
Quality
Consensus
TESIVRFPNITNLCPFGEVFNATRFASVYAWNRKRISNCVADYSVL
Occupancy

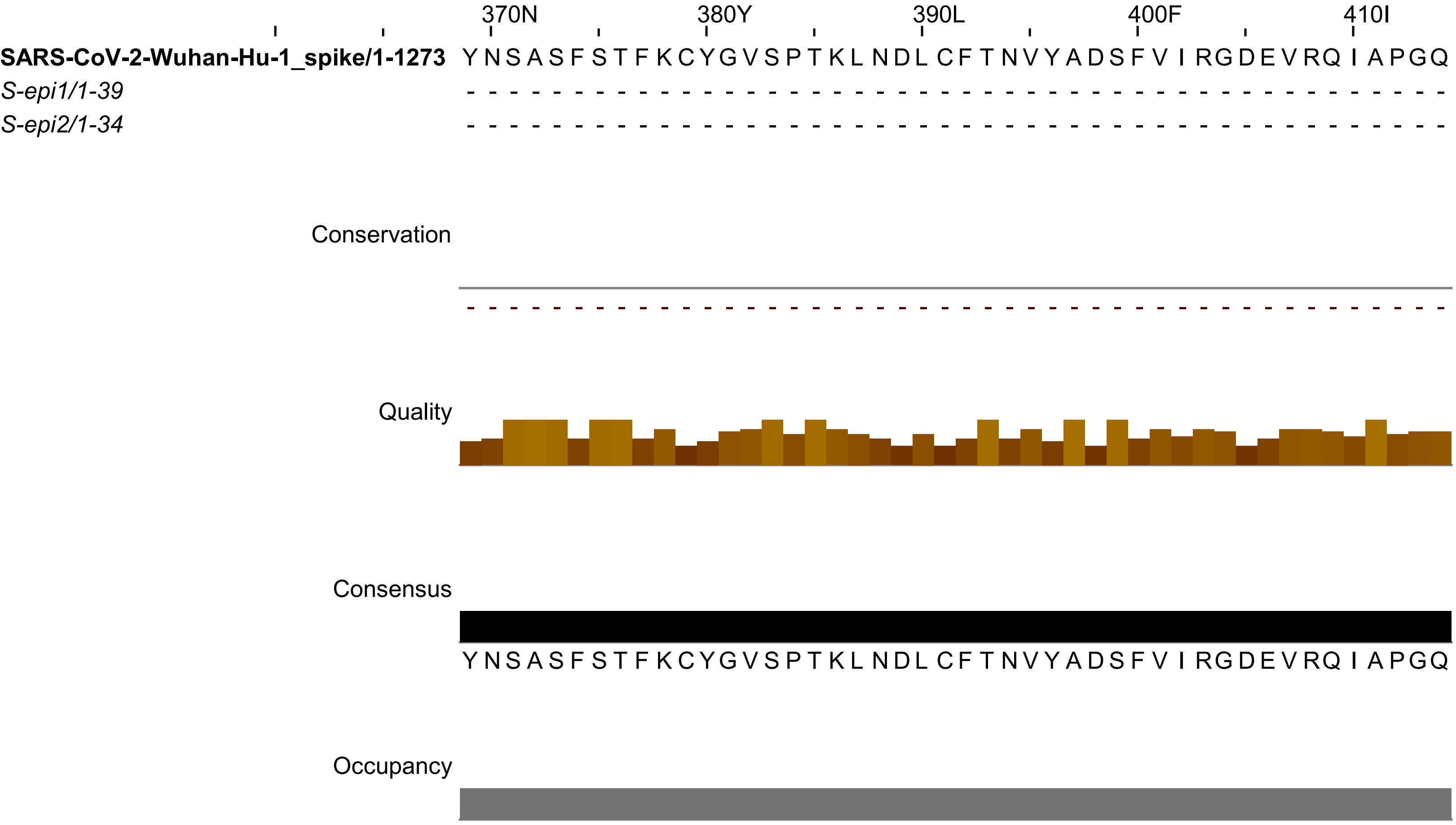
370N
380Y
390L
400F
410I
SARS-CoV-2-Wuhan-Hu-1_spike/1-1273 Y N S A S F S T F K C Y G V S P T K L N D L C F T N V Y A D S F V I R G D E V R Q I A P G Q
S-epi1/1-39
S-epi2/1-34
Conservation
Quality
Consensus
Y N S A S F S T F K C Y G V S P T K L N D L C F T N V Y A D S F V I R G D E V R Q I A P G Q
Occupancy

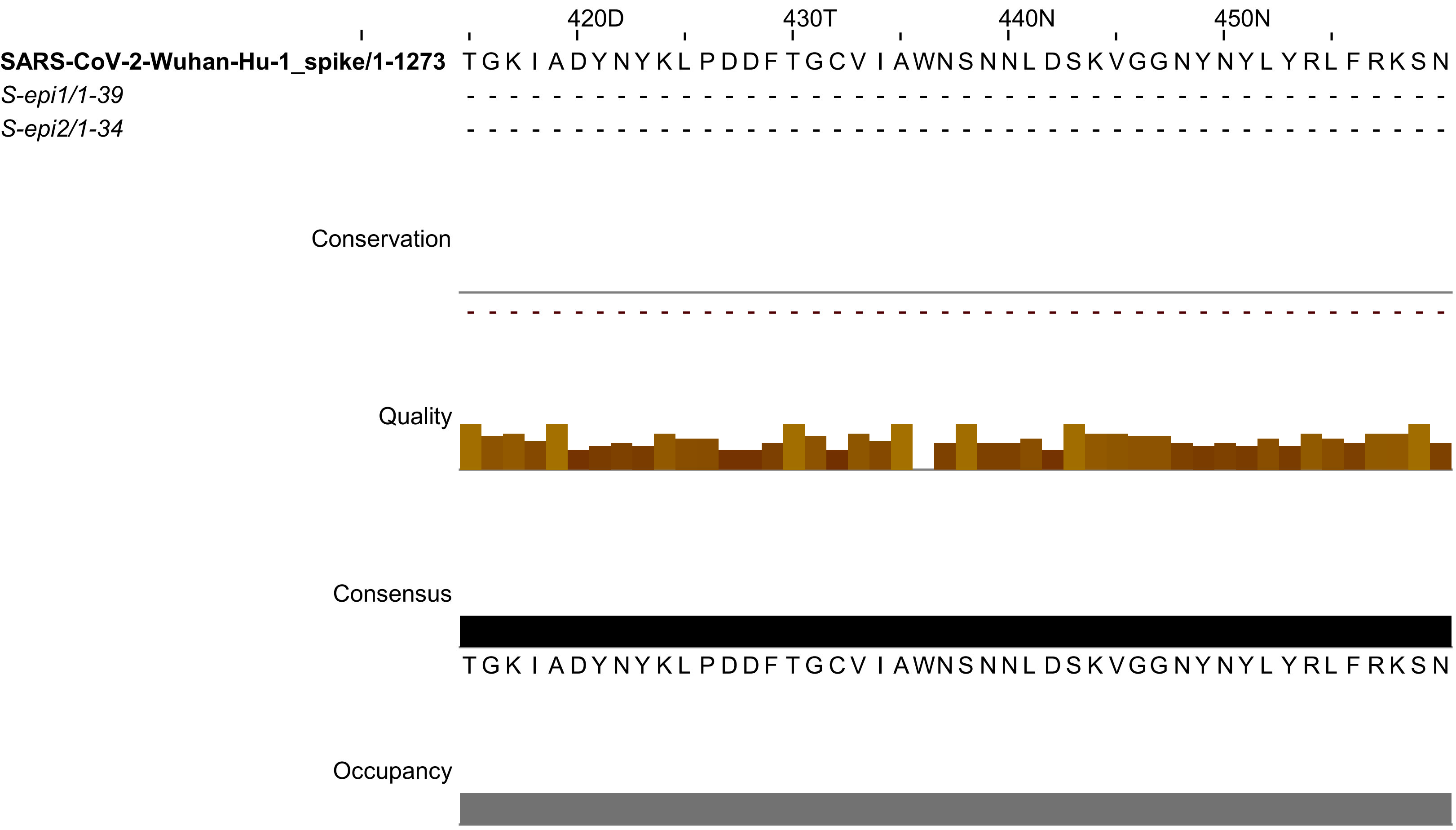

420D
430T
440N
450N
SARS-CoV-2-Wuhan-Hu-1_spike/1-1273
TGKIADYNYKLPDDFTGCVIAWNSNNLDSKVGGNYNYLYRLFRKSN
S-epi1/1-39
S-epi2/1-34
Conservation
Quality
Consensus
TGKIADYNYKLPDDFTGCVIAWNSNNLDSKVGGNYNYLYRLFRKSN
Occupancy

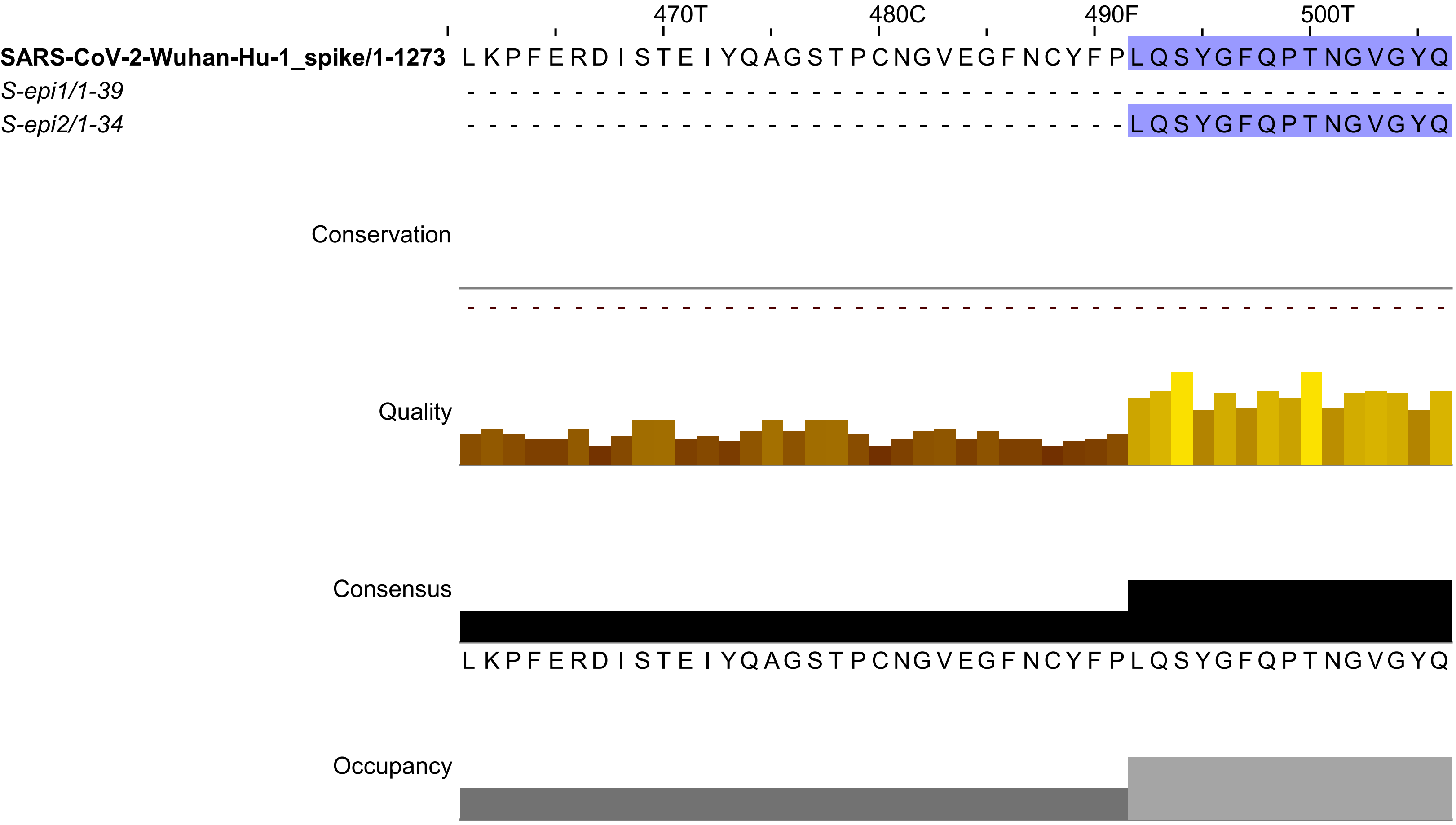
470T
480C
490F
500T
SARS-CoV-2-Wuhan-Hu-1_spike/1-1273 LKPFERDISTEIYQAGSTPCNGVEGFNCYFPLQSYGFQPTNGVGYQ
S-epi1/1-39
S-epi2/1-34 LQSYGFQPTNGVGYQ
Conservation
Quality
Consensus
LKPFERDISTEIYQAGSTPCNGVEGFNCYFPLQSYGFQPTNGVGYQ
Occupancy

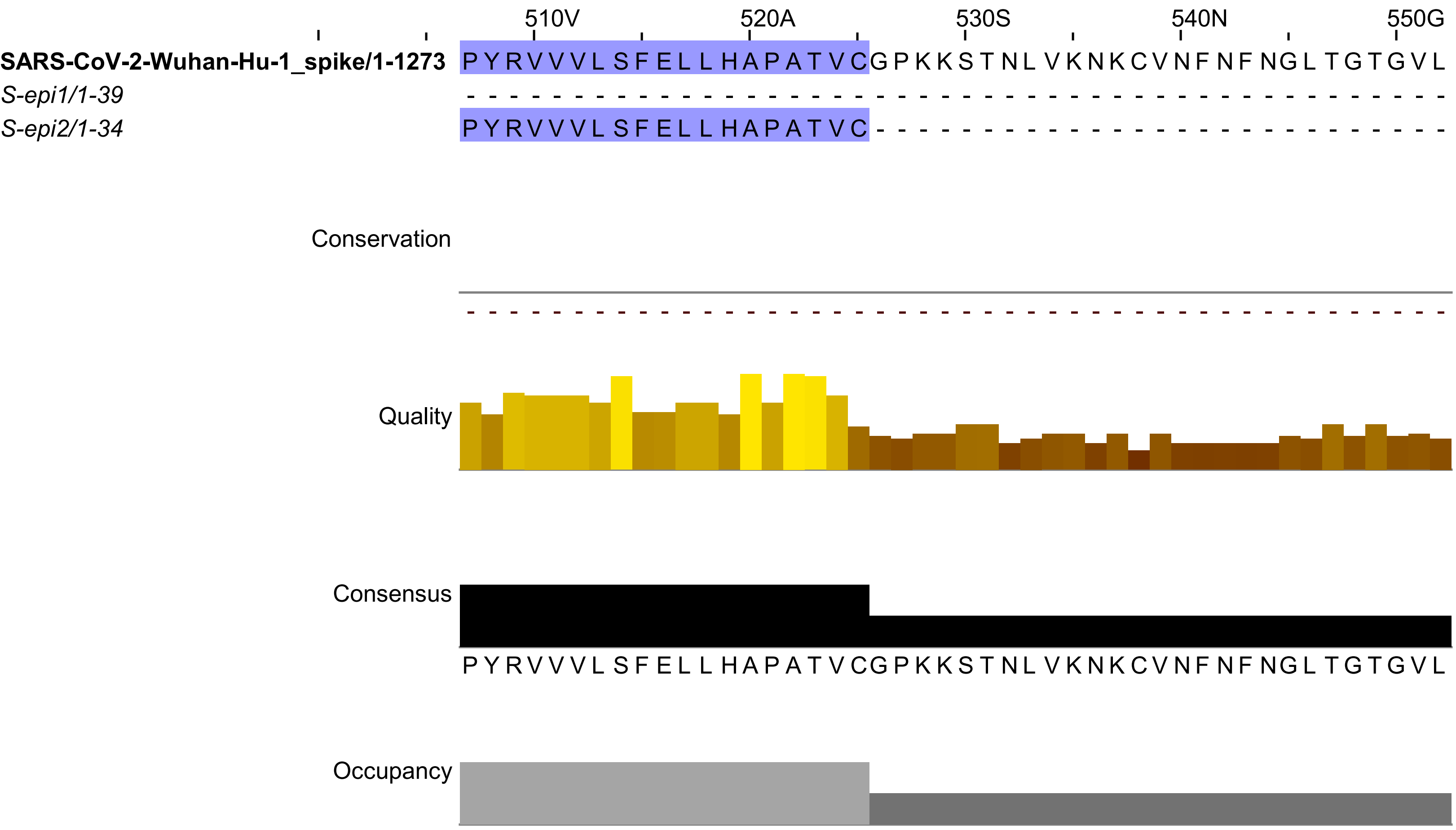
510V
520A
530S
540N
550G
SARS-CoV-2-Wuhan-Hu-1_spike/1-1273
PYRVVVLSFELLHAPATVCGPKKSTNLVKNKCVNFNFNGLTGTGVL
S-epi1/1-39
S-epi2/1-34
PYRVVVLSFELLHAPATVC
Conservation
Quality
Consensus
PYRVVVLSFELLHAPATVCGPKKSTNLVKNKCVNFNFNGLTGTGVL
Occupancy

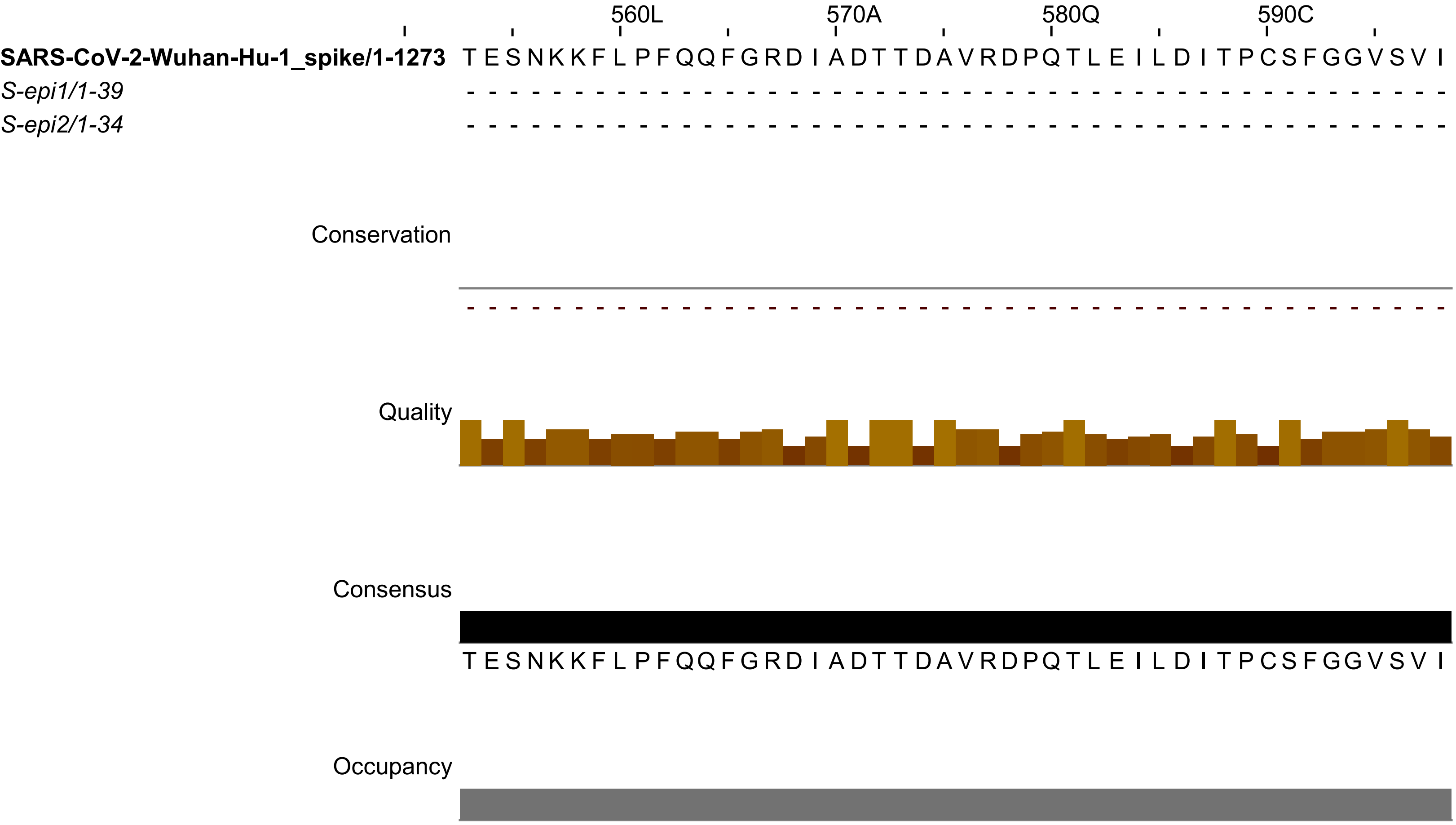
560L
570A
580Q
590C
SARS-CoV-2-Wuhan-Hu-1_spike/1-1273 T E S N K K F L P F Q Q F G R D I A D T T D A V R D P Q T L E I L D I T P C S F G G V S V I
S-epi1/1-39
S-epi2/1-34
Conservation
Quality
Consensus
T E S N K K F L P F Q Q F G R D I A D T T D A V R D P Q T L E I L D I T P C S F G G V S V I
Occupancy

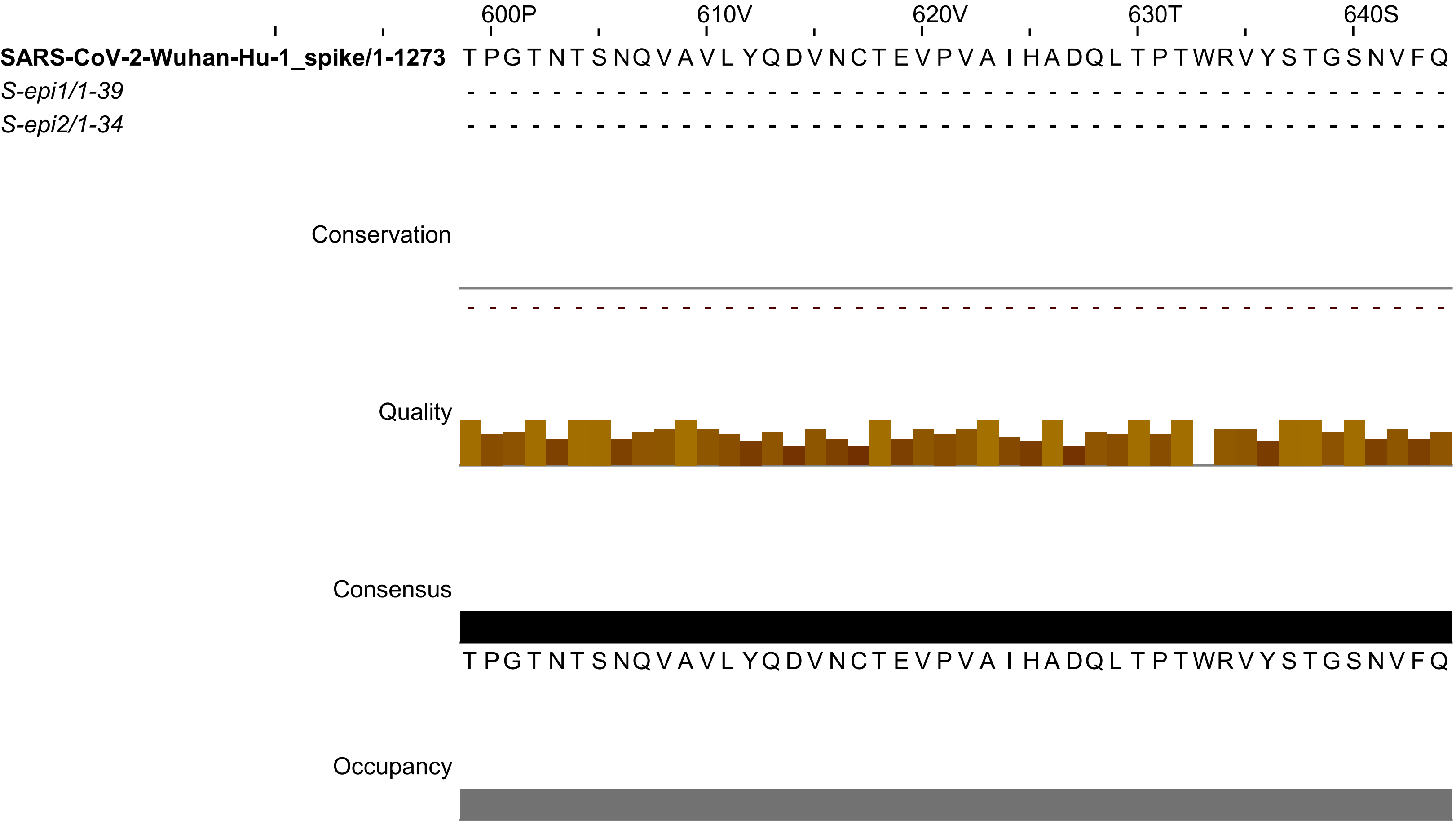
600P
610V
620V
630T
640S
SARS-CoV-2-Wuhan-Hu-1_spike/1-1273 TPGTNTSNQVAVLYQDVNCTEVPVAIHADQLTPTWRVYSTGSNVFQ
S-epi1/1-39
S-epi2/1-34
Conservation
Quality
Consensus
TPGTNTSNQVAVLYQDVNCTEVPVAIHADQLTPTWRVYSTGSNVFQ
Occupancy

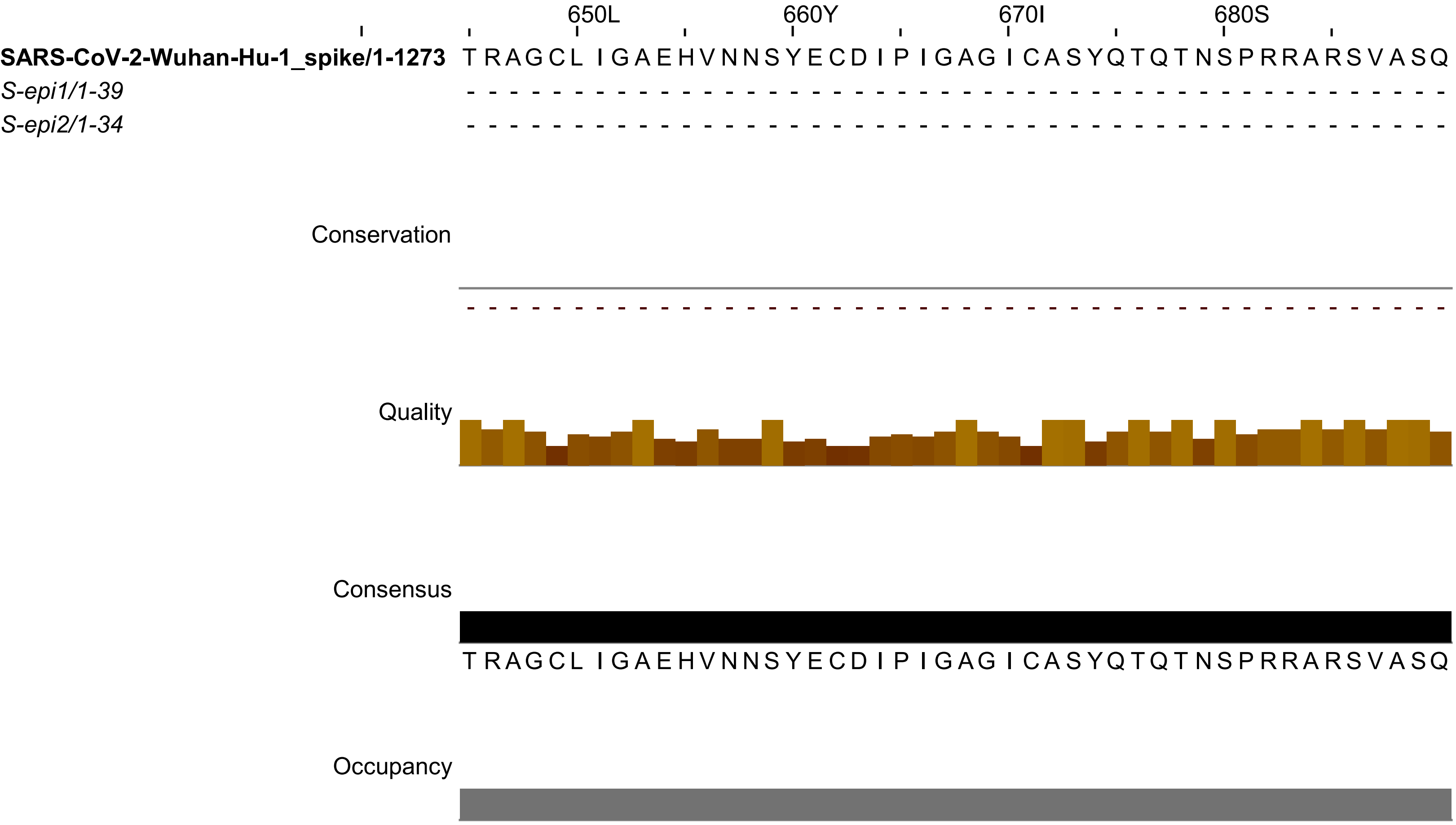
650L
660Y
670I
680S
SARS-CoV-2-Wuhan-Hu-1_spike/1-1273
TRAGCLIGAEHVNNSYECDIPIGAGICASYQTQTNSPRRARSVASQ
S-epi1/1-39
S-epi2/1-34
Conservation
Quality
Consensus
TRAGCLIGAEHVNNSYECDIPIGAGICASYQTQTNSPRRARSVASQ
Occupancy

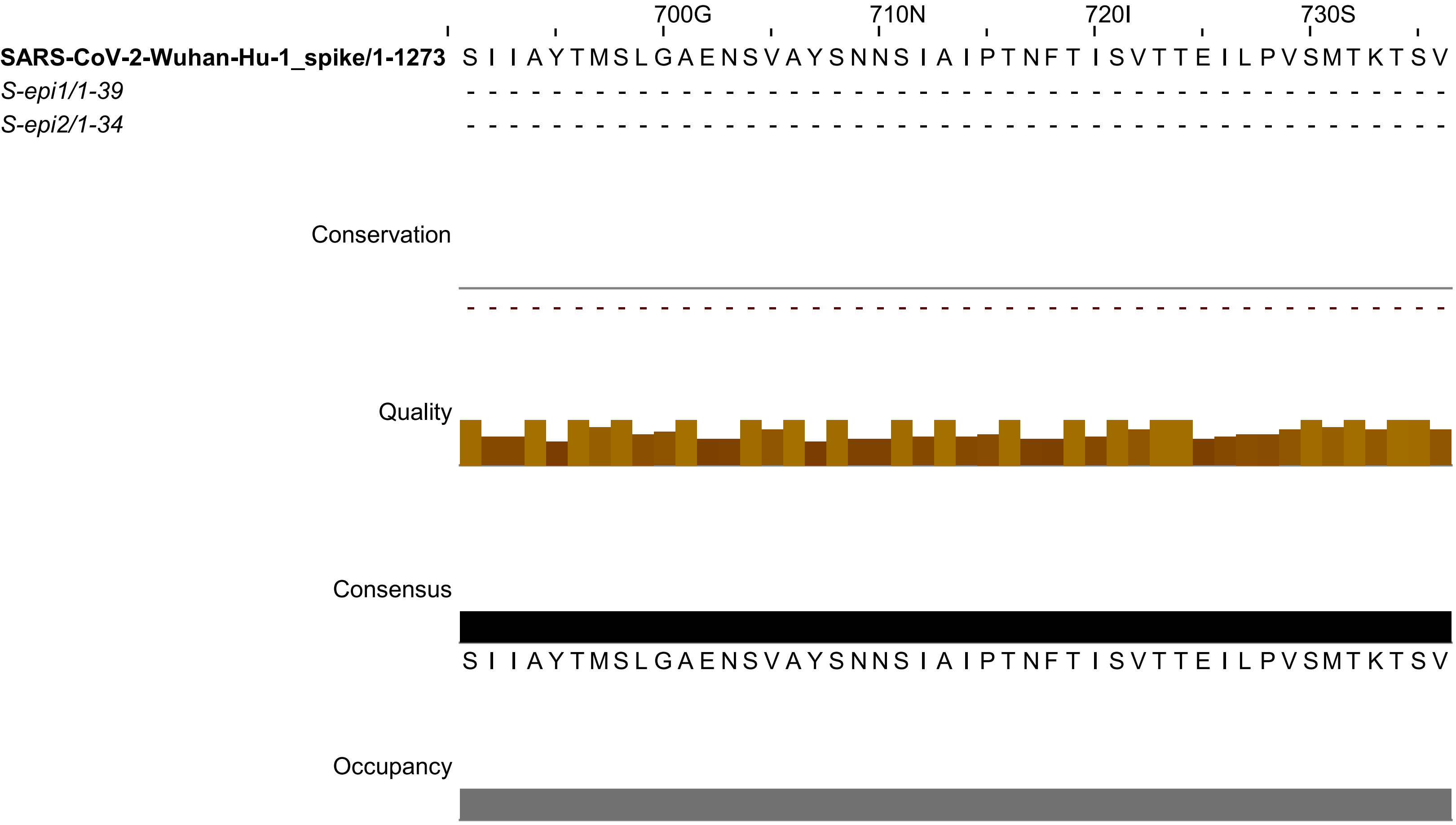
700G
710N
720I
730S
SARS-CoV-2-Wuhan-Hu-1_spike/1-1273
S I I A Y T M S L G A E N S V A Y S N N S I A I P T N F T I S V T T E I L P V S M T K T S V
S-epi1/1-39
S-epi2/1-34
Conservation
Quality
Consensus
S I I A Y T M S L G A E N S V A Y S N N S I A I P T N F T I S V T T E I L P V S M T K T S V
Occupancy

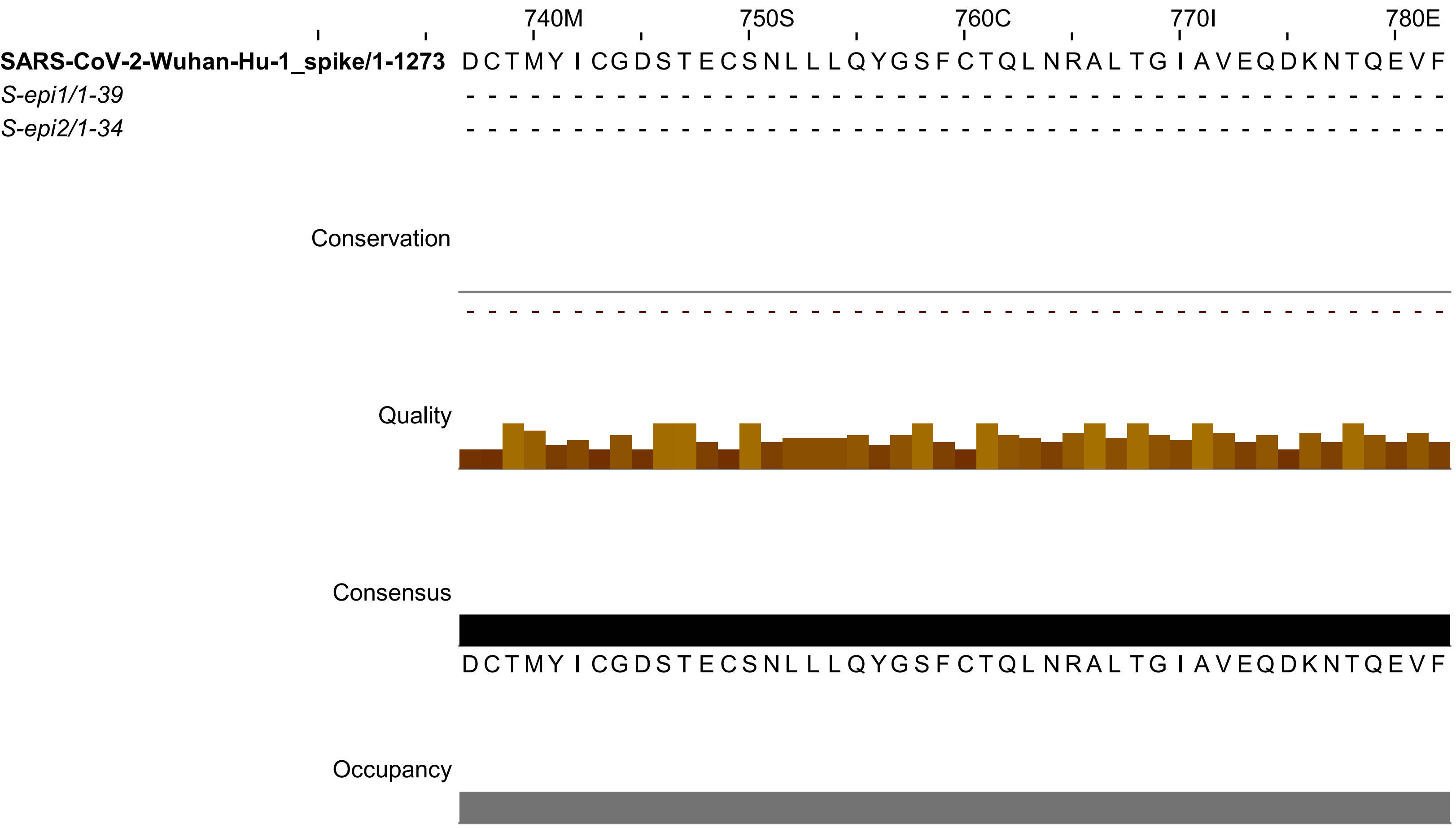
740M
750S
760C
770I
780E
SARS-CoV-2-Wuhan-Hu-1_spike/1-1273
D C T M Y I C G D S T E C S N L L L Q Y G S F C T Q L N R A L T G I A V E Q D K N T Q E V F
S-epi1/1-39
S-epi2/1-34
Conservation
Quality
Consensus
D C T M Y I C G D S T E C S N L L L Q Y G S F C T Q L N R A L T G I A V E Q D K N T Q E V F
Occupancy

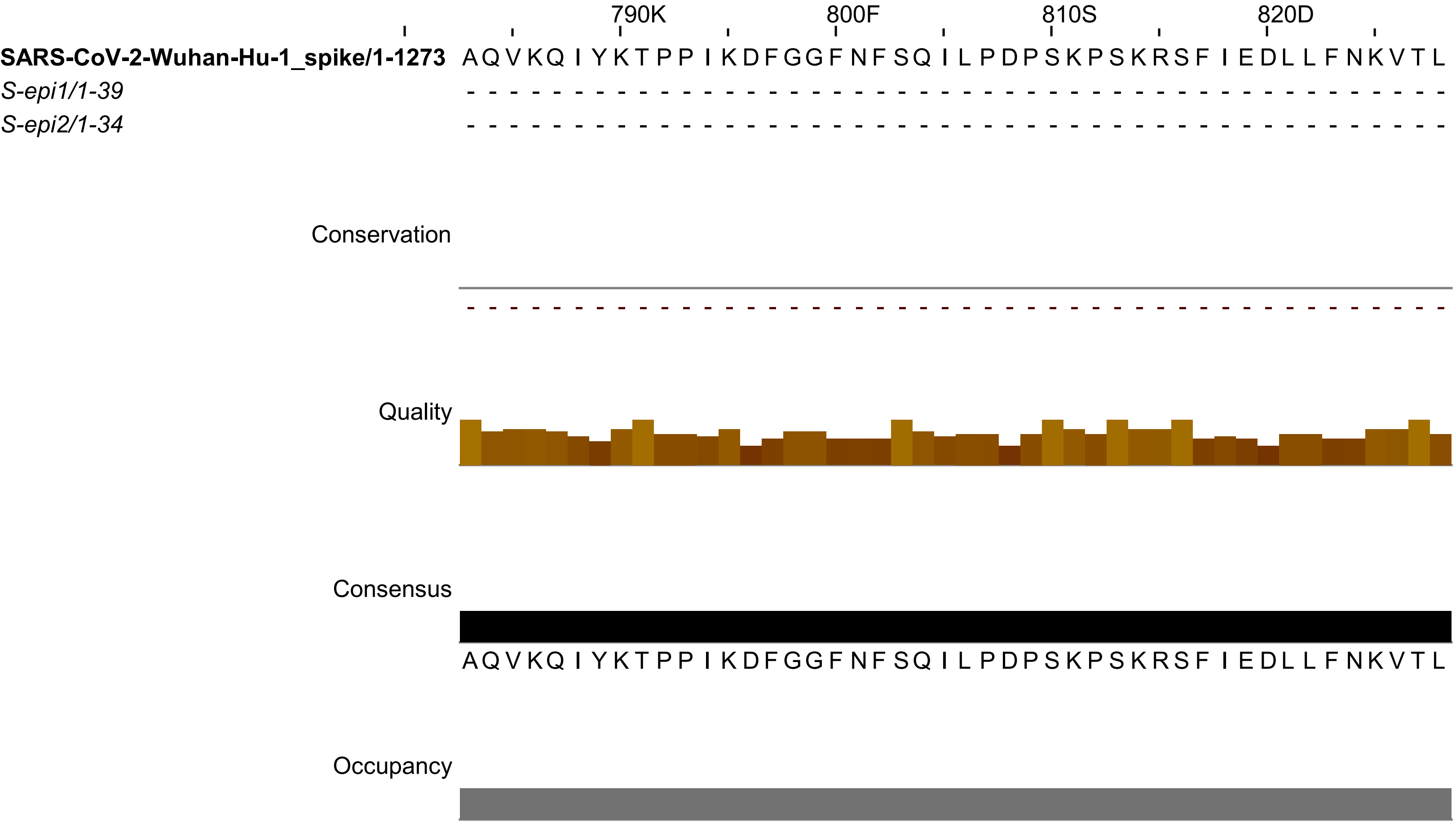
790K
800F
810S
820D
SARS-CoV-2-Wuhan-Hu-1_spike/1-1273 AQVKQIYKTPPIKDFGGFNFSQILPDPSKPSKRSFIEDLLFNKVTL
S-epi1/1-39
S-epi2/1-34
Conservation
Quality
Consensus
AQVKQIYKTPPIKDFGGFNFSQILPDPSKPSKRSFIEDLLFNKVTL
Occupancy

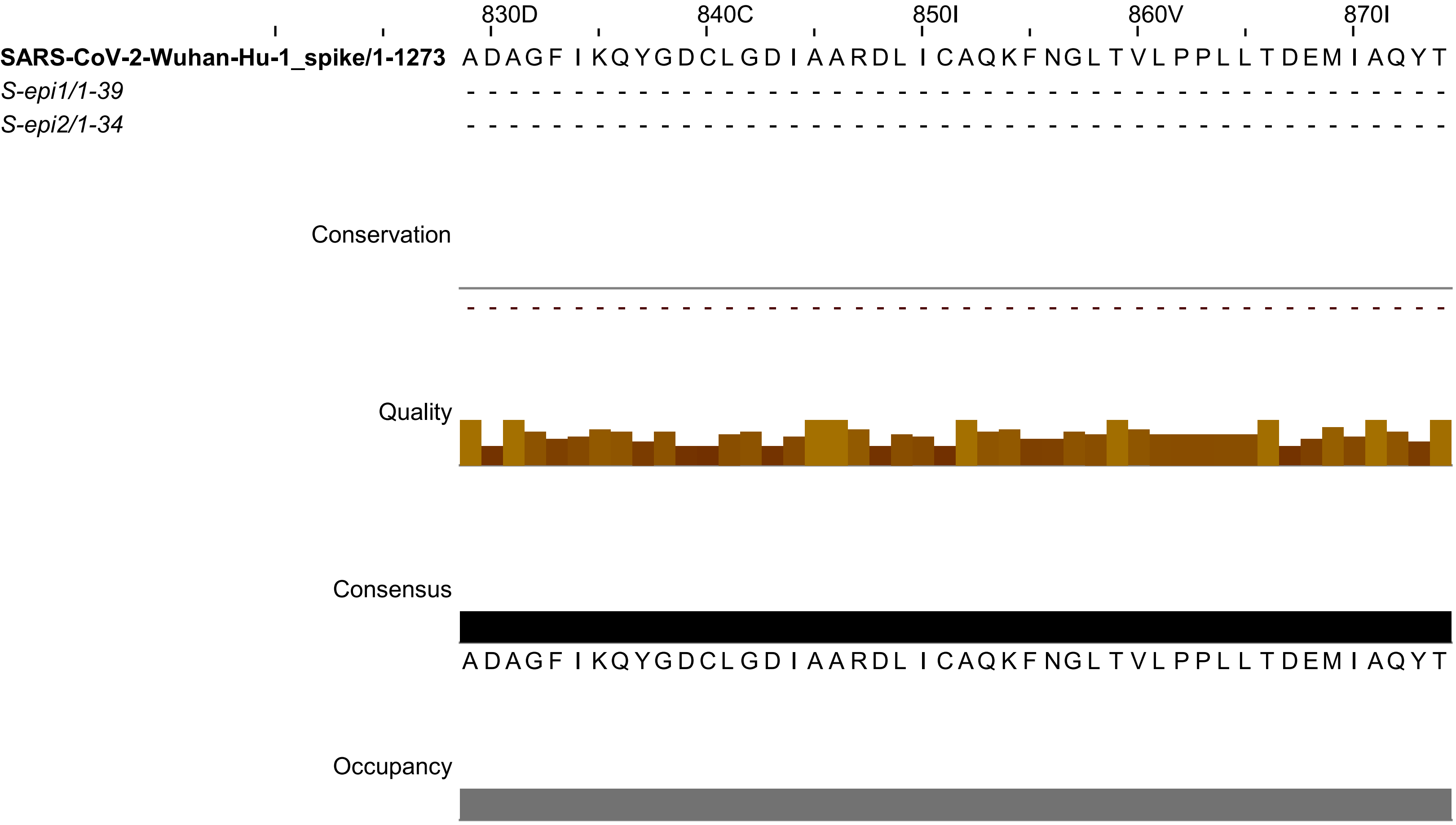
830D
840C
850I
860V
870I
SARS-CoV-2-Wuhan-Hu-1_spike/1-1273
ADAGFIKQYGDCLGDIAARDLICAQKFNGLTVLPPLLTDEMIAQYT
S-epi1/1-39
S-epi2/1-34
Conservation
Quality
Consensus
ADAGFIKQYGDCLGDIAARDLICAQKFNGLTVLPPLLTDEMIAQYT
Occupancy

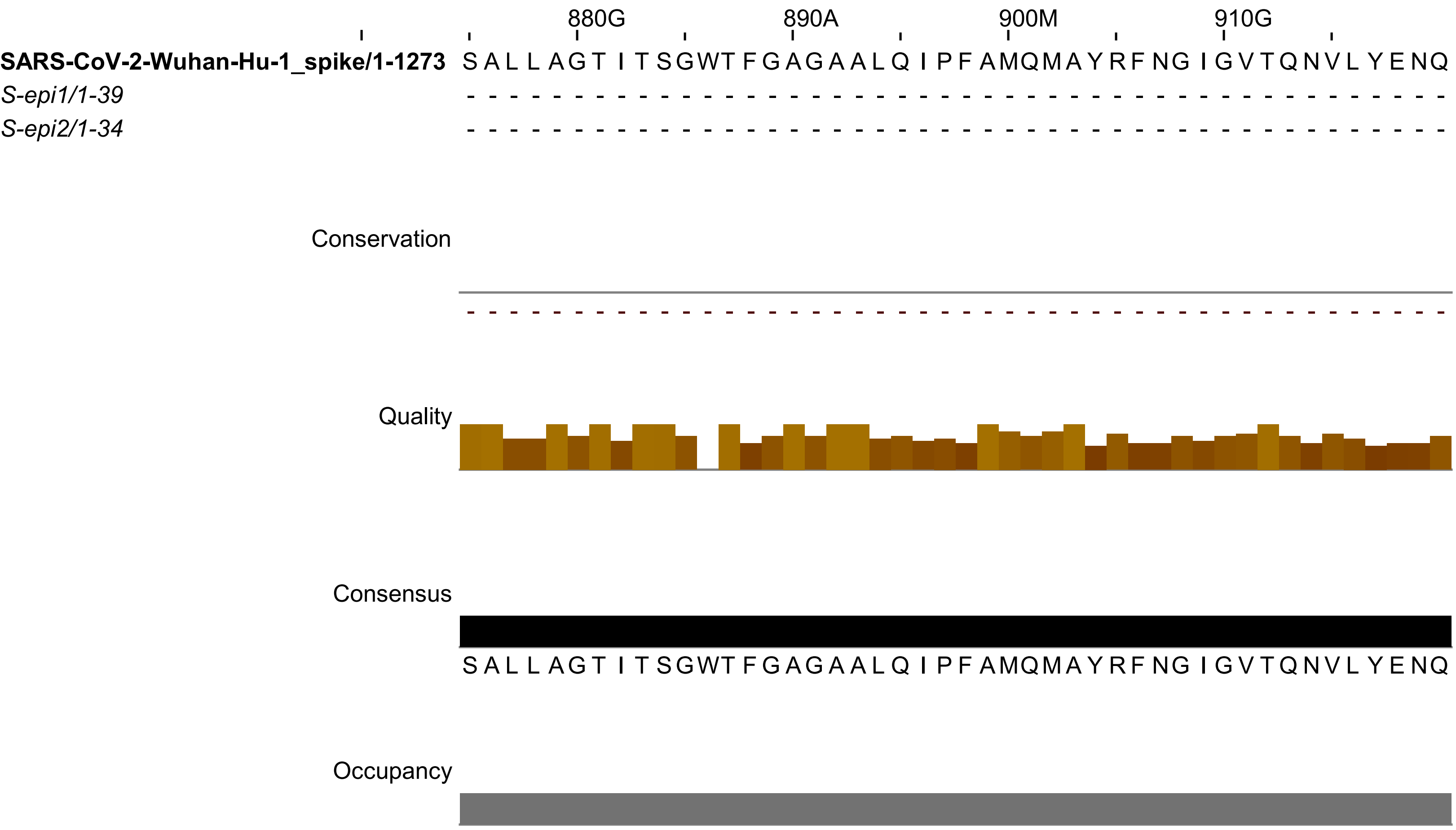
880G
890A
900M
910G
SARS-CoV-2-Wuhan-Hu-1_spike/1-1273
SALLAGTITSGWTFGAGAALQIPFAMQMAYRFNGIGVTQNVLYENQ
S-epi1/1-39
S-epi2/1-34
Conservation
Quality
Consensus
SALLAGTITSGWTFGAGAALQIPFAMQMAYRFNGIGVTQNVLYENQ
Occupancy

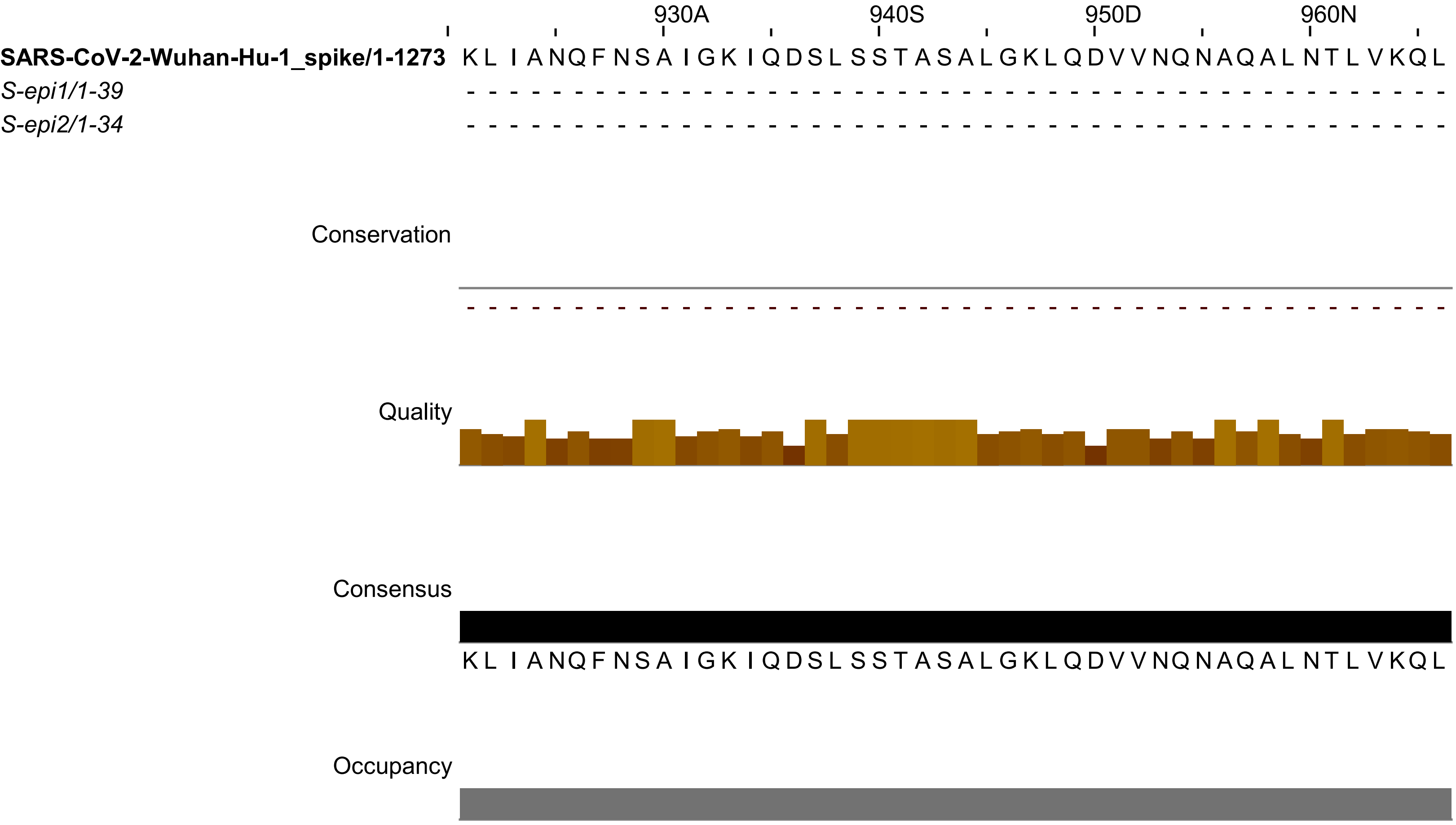
930A
940S
950D
960N
SARS-CoV-2-Wuhan-Hu-1_spike/1-1273 KLIANQFNSAIGKIQDSLSSTASALGKLQDVVNQNAQALNTLVKQL
S-epi1/1-39
S-epi2/1-34
Conservation
Quality
Consensus
KLIANQFNSAIGKIQDSLSSTASALGKLQDVVNQNAQALNTLVKQL
Occupancy

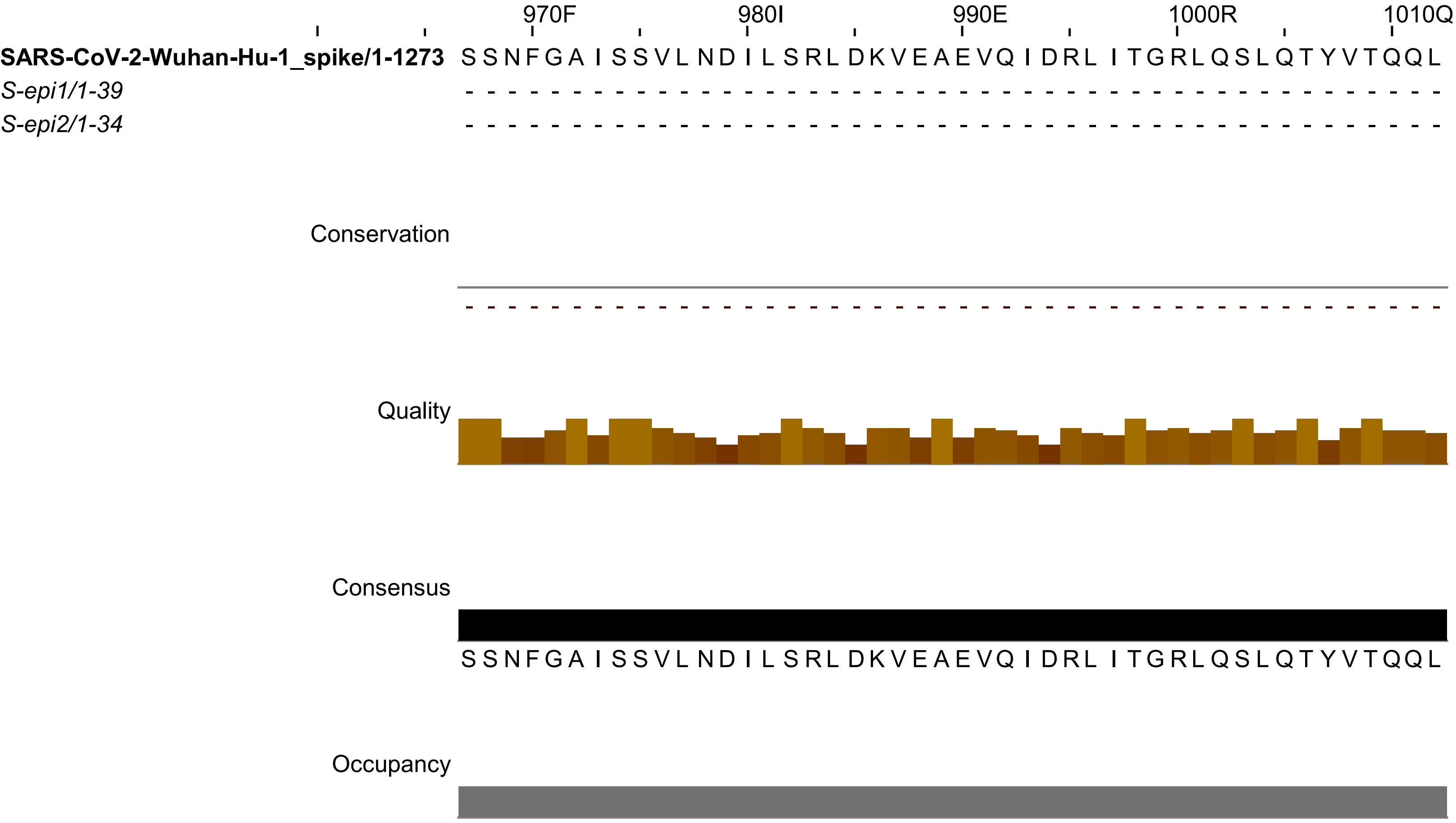
970F
980I
990E
1000R
1010Q
SARS-CoV-2-Wuhan-Hu-1_spike/1-1273
SSNFGAISSVLNDILSRLDKVEAEVQIDRLITGRLQSLQTYVTQQL
S-epi1/1-39
S-epi2/1-34
Conservation
Quality
Consensus
SSNFGAISSVLNDILSRLDKVEAEVQIDRLITGRLQSLQTYVTQQL
Occupancy

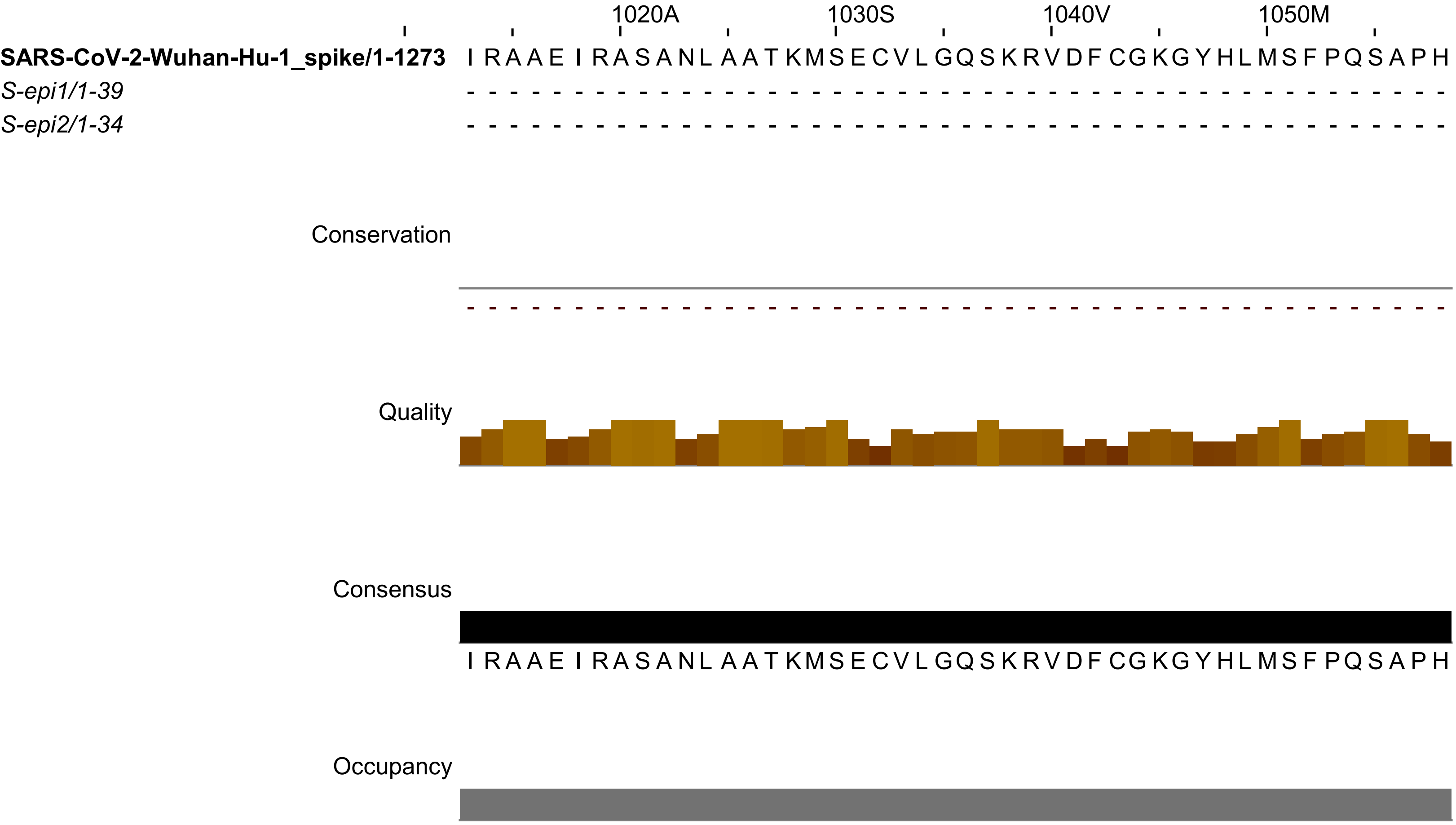
1020A
1030S
1040V
1050M
SARS-CoV-2-Wuhan-Hu-1_spike/1-1273
I R A A E I R A S A N L A A T K M S E C V L G Q S K R V D F C G K G Y H L M S F P Q S A P H
S-epi1/1-39
S-epi2/1-34
Conservation
Quality
Consensus
I R A A E I R A S A N L A A T K M S E C V L G Q S K R V D F C G K G Y H L M S F P Q S A P H
Occupancy

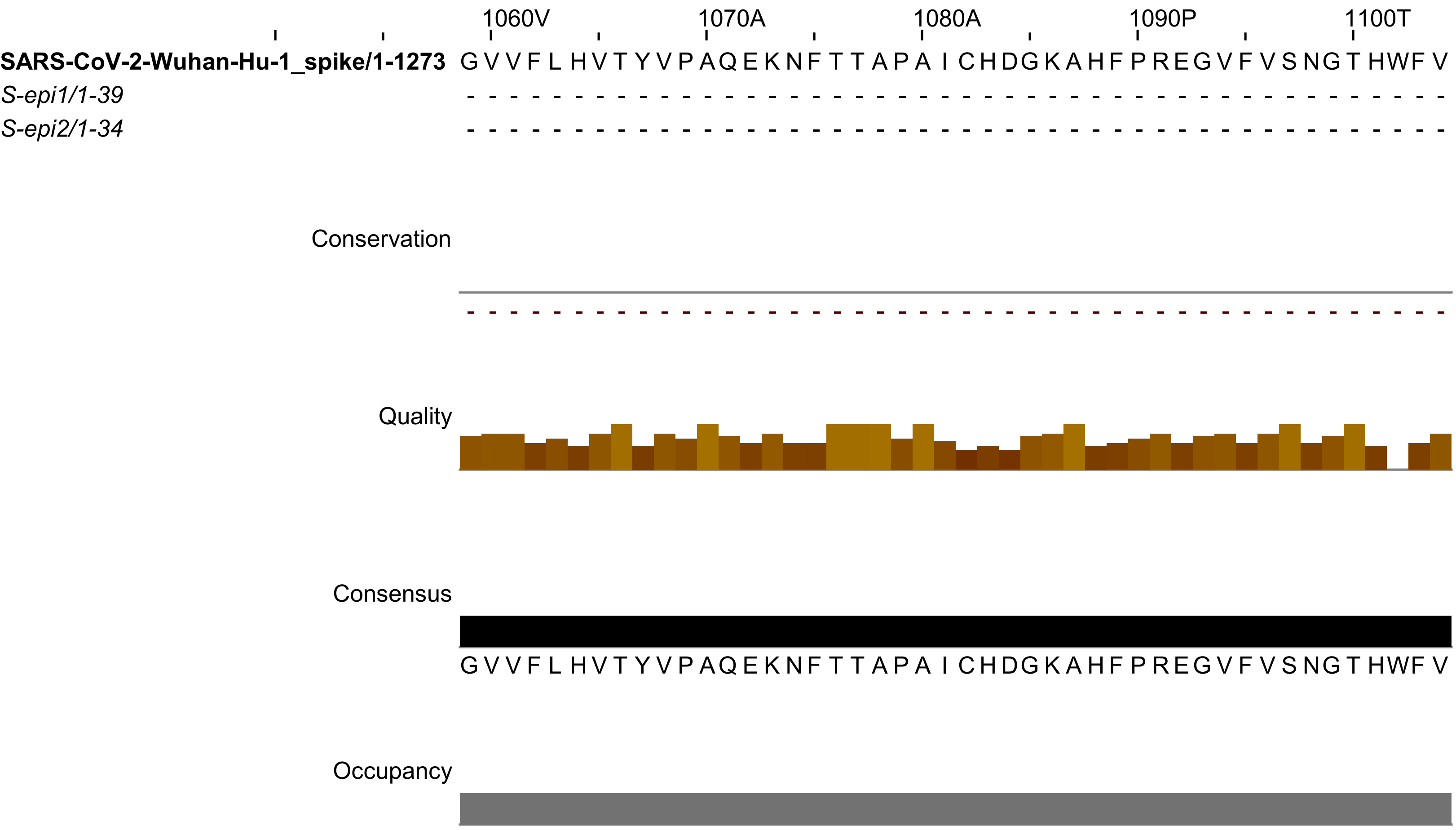
1060V
1070A
1080A
1090P
1100T
SARS-CoV-2-Wuhan-Hu-1_spike/1-1273
G V V F L H V T Y V P A Q E K N F T T A P A I C H D G K A H F P R E G V F V S N G T H W F V
S-epi1/1-39
S-epi2/1-34
Conservation
Quality
Consensus
G V V F L H V T Y V P A Q E K N F T T A P A I C H D G K A H F P R E G V F V S N G T H W F V
Occupancy

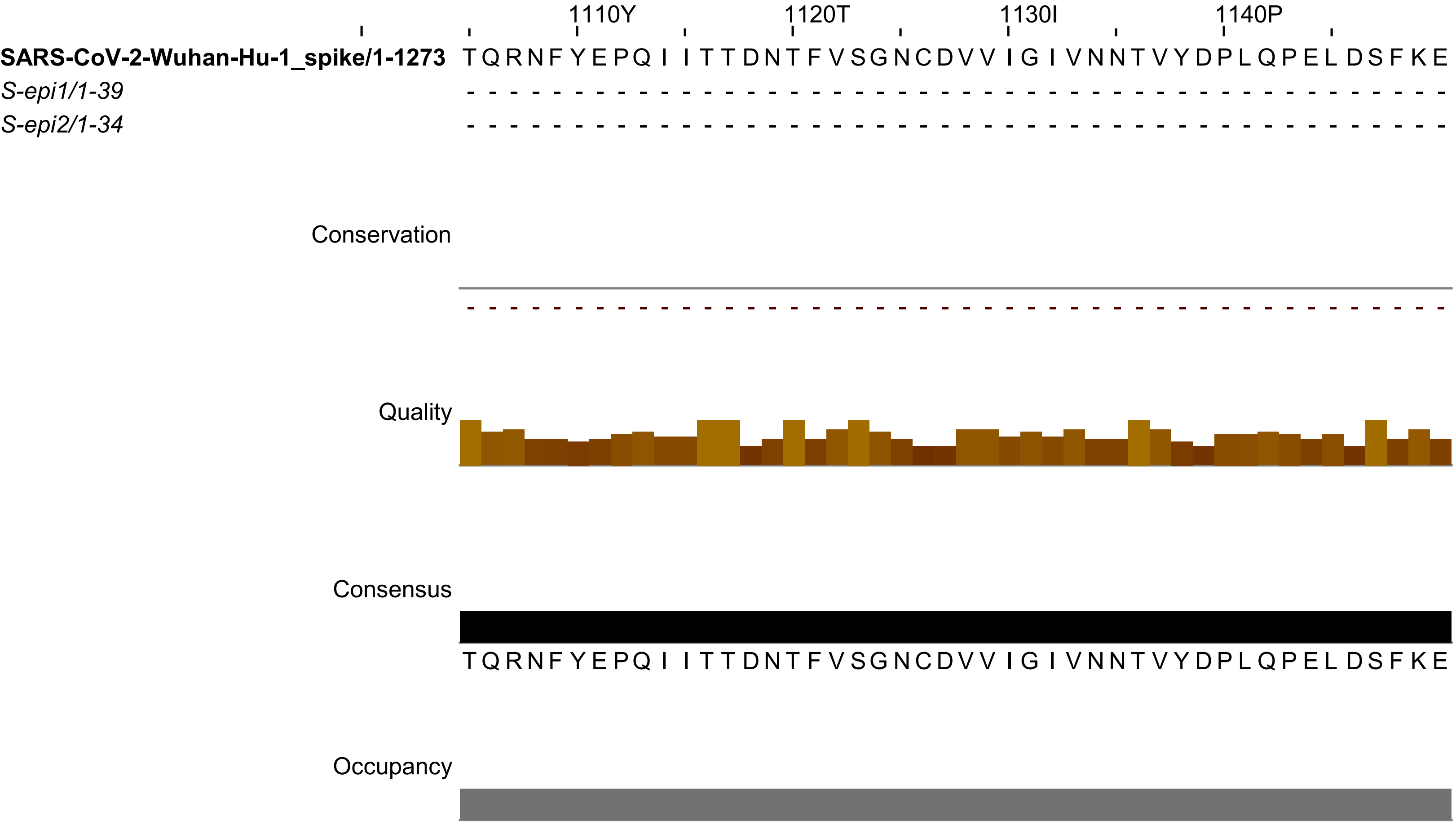
1110Y
1120T
1130I
1140P
SARS-CoV-2-Wuhan-Hu-1_spike/1-1273
TQRNFYEPQIITTDNTFVSGNCDVVIGIVNNTVYDPLQPELDSFKE
S-epi1/1-39
S-epi2/1-34
Conservation
Quality
Consensus
TQRNFYEPQIITTDNTFVSGNCDVVIGIVNNTVYDPLQPELDSFKE
Occupancy

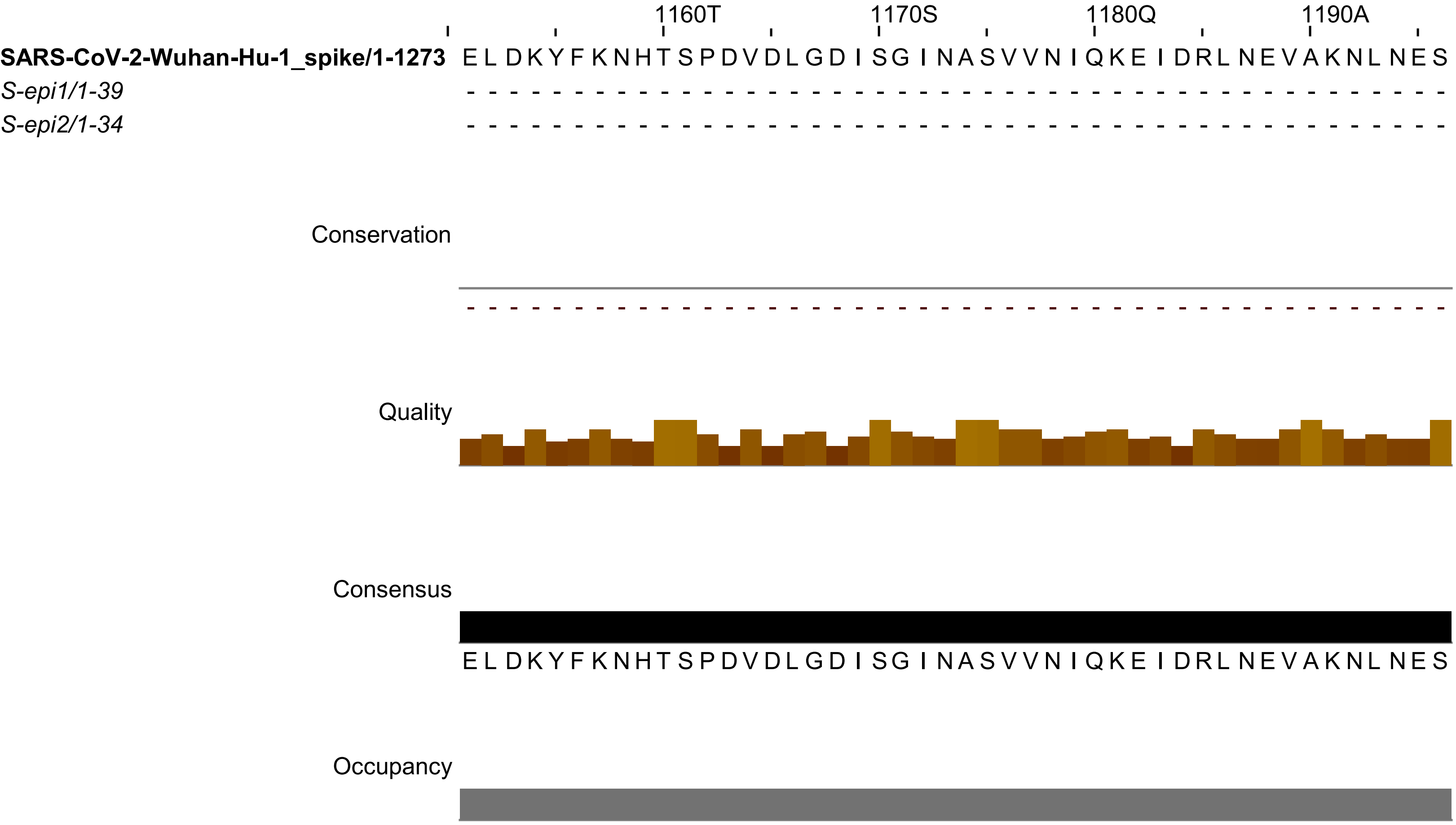
1160T
1170S
1180Q
1190A
SARS-CoV-2-Wuhan-Hu-1_spike/1-1273
E L D K Y F K N H T S P D V D L G D I S G I N A S V V N I Q K E I D R L N E V A K N L N E S
S-epi1/1-39
S-epi2/1-34
Conservation
Quality
Consensus
E L D K Y F K N H T S P D V D L G D I S G I N A S V V N I Q K E I D R L N E V A K N L N E S
Occupancy

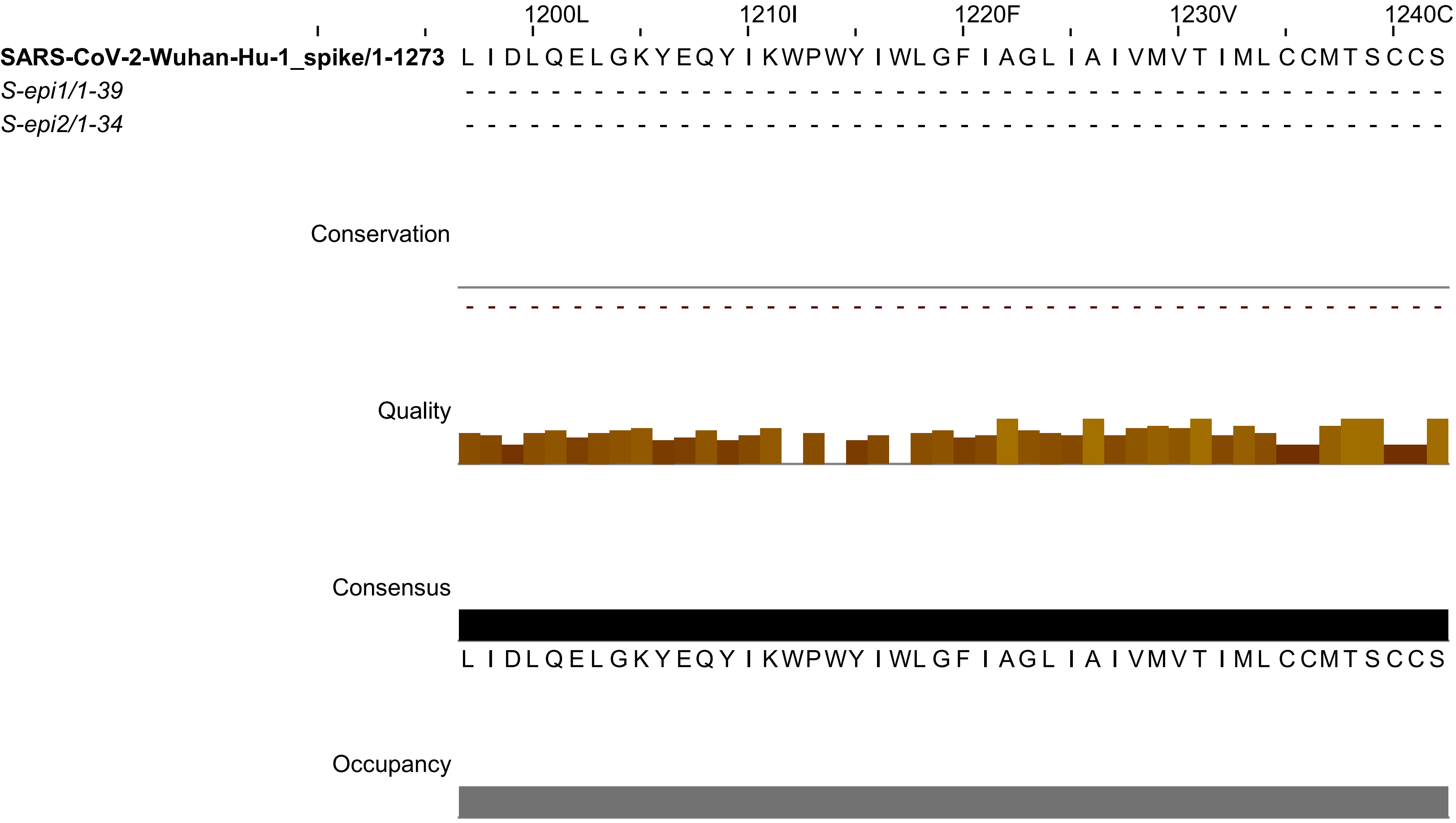
1200L
1210I
1220F
1230V
1240C
SARS-CoV-2-Wuhan-Hu-1_spike/1-1273
L I D L Q E L G K Y E Q Y I K W P W Y I W L G F I A G L I A I V M V T I M L C C M T S C C S
S-epi1/1-39
S-epi2/1-34
Conservation
Quality
Consensus
L I D L Q E L G K Y E Q Y I K W P W Y I W L G F I A G L I A I V M V T I M L C C M T S C C S
Occupancy

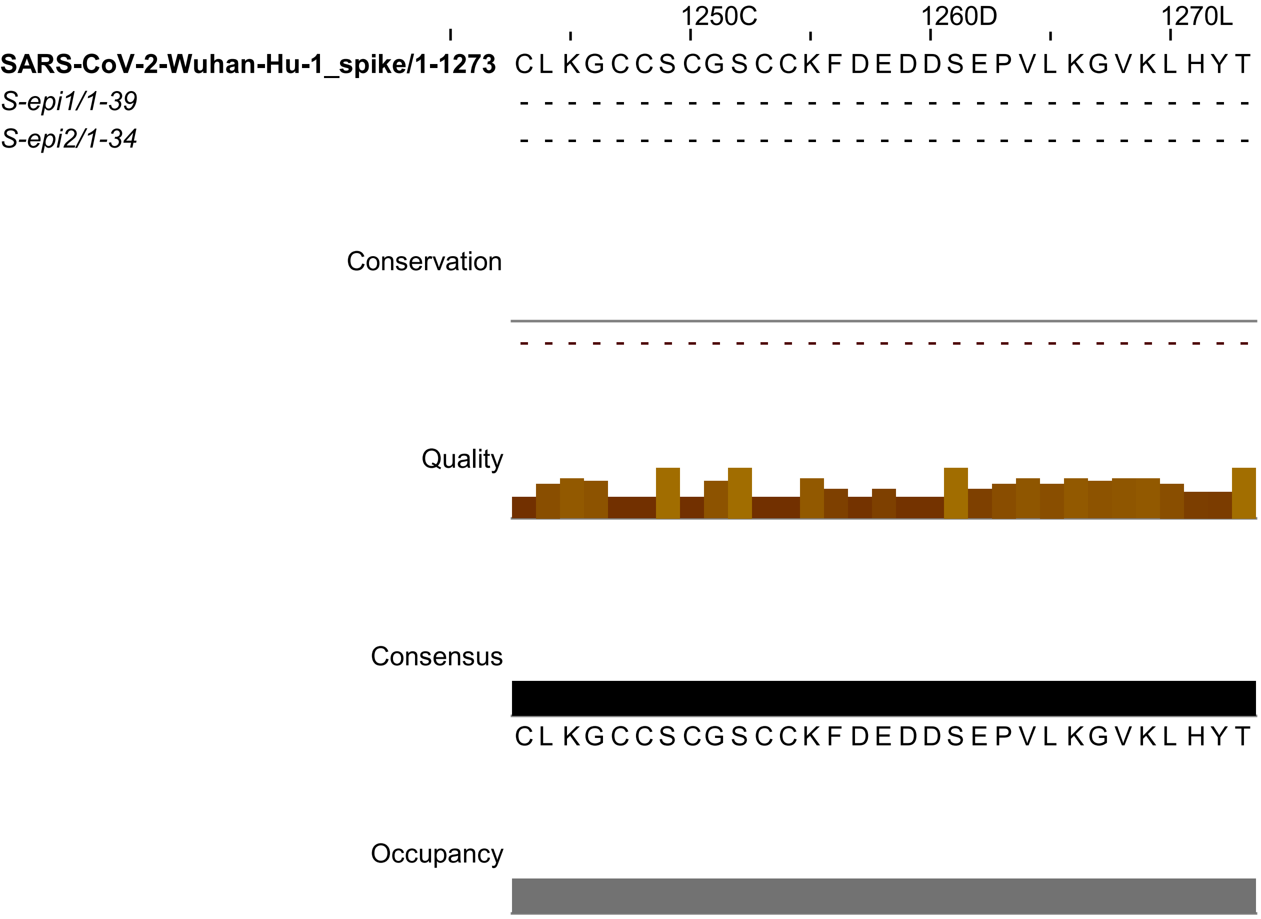
1250C
1260D
1270L
SARS-CoV-2-Wuhan-Hu-1_spike/1-1273 CLKGCCSCGSCCKFDEDDSEPVLKGVKLHYT
S-epi1/1-39
S-epi2/1-34
Conservation
Quality
Consensus
CLKGCCSCGSCCKFDEDDSEPVLKGVKLHYT
Occupancy
