# Supplementary material for: An Immunoinformatic Approach for Identifying and Designing Conserved Multi-Epitope Vaccines for Coronaviruses
Source: Biomedicines. 2024 Nov 5;12(11):2530. doi: 10.3390/biomedicines12112530 (PMC11592158; doi:10.3390/biomedicines12112530)
Supplement: Supplementary file 1 [file biomedicines-12-02530-s001.zip › Supplementary Figure S5. epi1+pruav1 alignment.pdf]

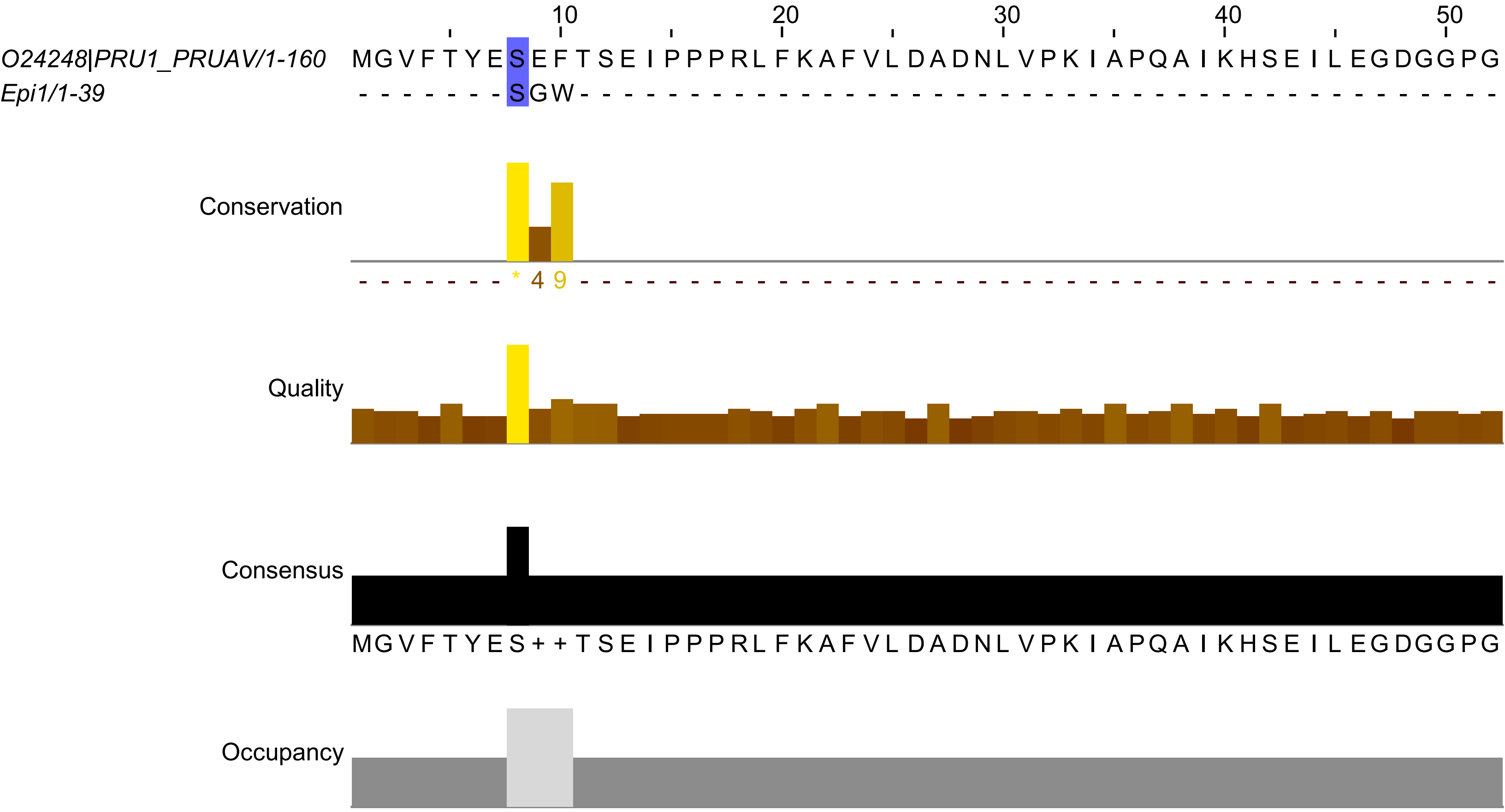
10
20
30
40
50
O24248|PRU1_PRUAV/1-160
MGVFTYESEFTSEIPPPRLFKAFVLDADNLVPKIAPQAIKHSEILEGDGGPG
Epi1/1-39
--------SGW---
Conservation
--------*49---
Quality
Consensus
MGVFTYES++TSEIPPPRLFKAFVLDADNLVPKIAPQAIKHSEILEGDGGPG
Occupancy

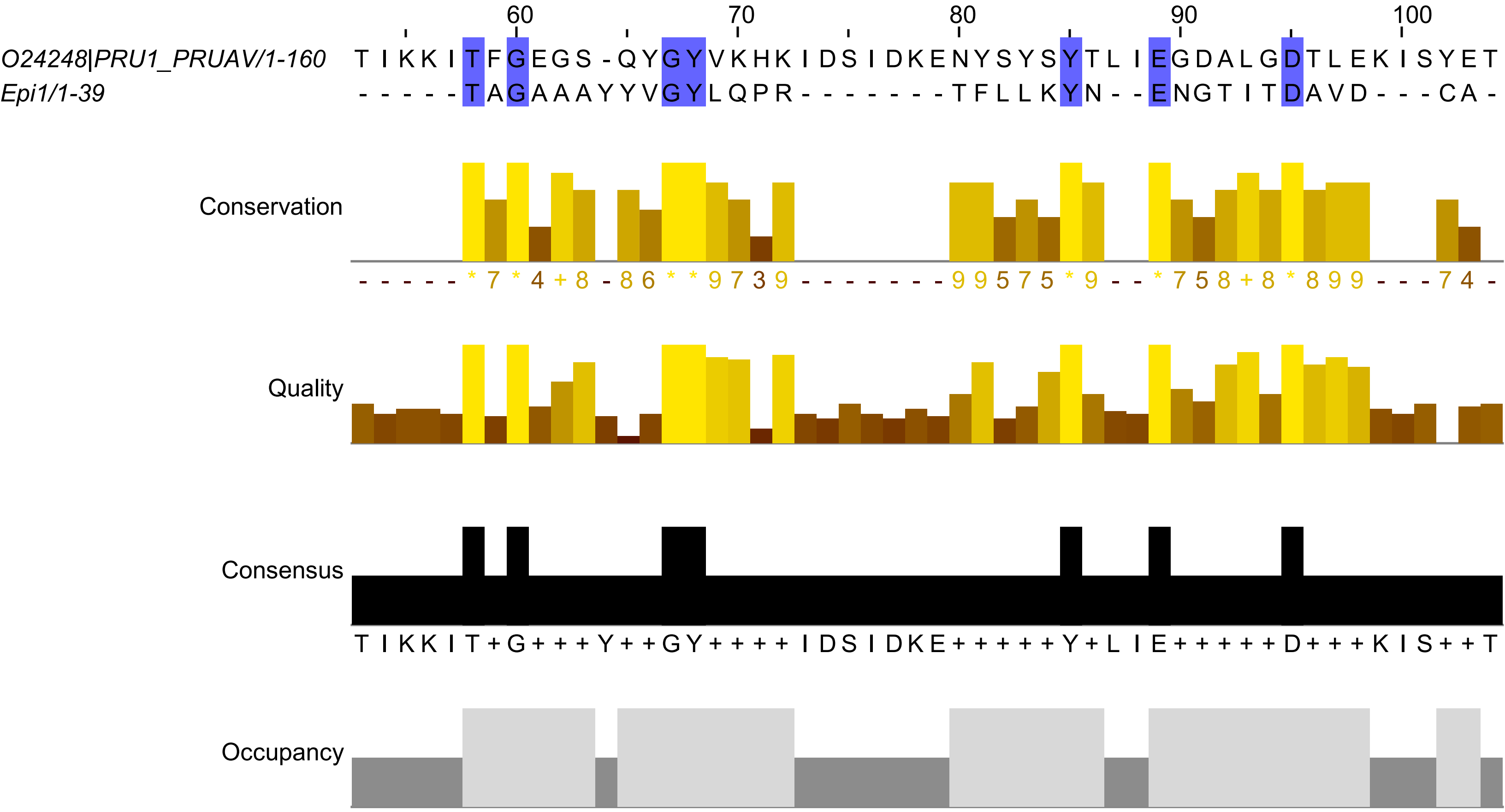

60 70 80 90 100
O24248|PRU1_PRUAV/1-160 TIKKITFGEGS-QYGYVKHKIDSIDKENYSYSYTLIEGDALGDTLEKISYET
Epi1/1-39 -----TAGAAAYYVGYLQPR-------TFLLKYN--ENGTITDAVD---CA-
Conservation
- - - - - * 7 * 4 + 8 - 8 6 * * 9 7 3 9 - - - - - - - 9 9 5 7 5 * 9 - - * 7 5 8 + 8 * 8 9 9 - - - 7 4 -
Quality
Consensus
TIKKIT+G+++Y++GY++++IDSIDKE+++++Y+LIE+++++D+++KIS++T
Occupancy

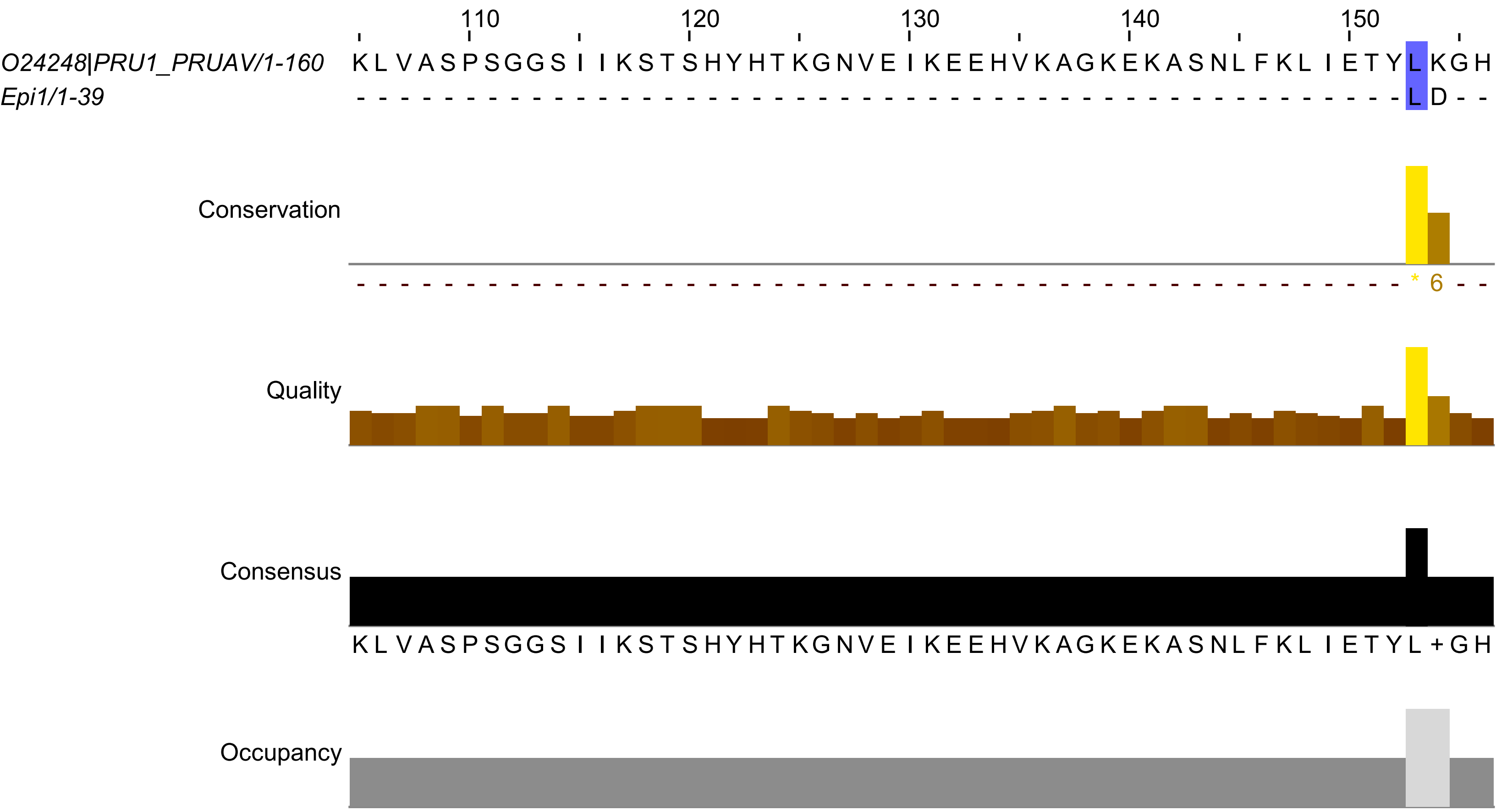
110 120 130 140 150
O24248|PRU1_PRUAV/1-160 KLVASPSGGSIIKSTSHYHTKGNVEIKEEHVKAGKEKASNLFKLIETYLKGH
Epi1/1-39 --LD--
Conservation
* 6
Quality
Consensus
KLVASPSGGSIIKSTSHYHTKGNVEIKEEHVKAGKEKASNLFKLIETYL+GH
Occupancy

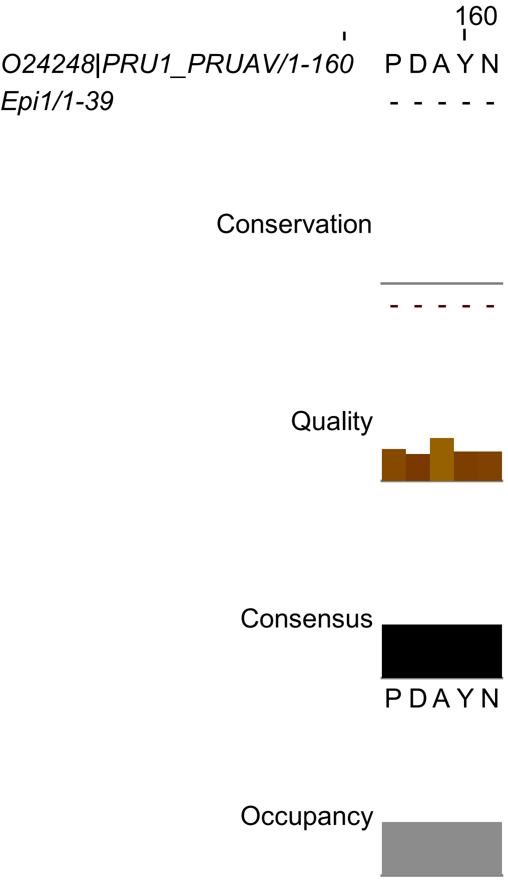
160
O24248|PRU1_PRUAV/1-160 P D A Y N
Epi1/1-39 - - - - -
Conservation
- - - - -
Quality
Consensus
P D A Y N
Occupancy
