# Supplementary material for: An Immunoinformatic Approach for Identifying and Designing Conserved Multi-Epitope Vaccines for Coronaviruses
Source: Biomedicines. 2024 Nov 5;12(11):2530. doi: 10.3390/biomedicines12112530 (PMC11592158; doi:10.3390/biomedicines12112530)
Supplement: Supplementary file 1 [file biomedicines-12-02530-s001.zip › Supplementary Table S1.pdf]

Supplementary Table S1.0 Final selected HTL epitopes.

Peptide	Number of matched HLA class-II alleles (out of 16)	Number of matched coronavirus strains	HLA class-II allele	Location in S glycoprotein	Assigned Name
WYIWLGFIA GLIAIV	15	23	HLA-DQA1*05:01/DQB1*03:01;HLA-DQA1*01:01/DQB1*05:01;HLA-DRB1*09:01;HLA-DPA1*03:01/DPB1*04:02;HLA-DRB1*01:01;HLA-DQA1*05:01/DQB1*02:01;HLA-DPA1*02:01/DPB1*01:01;HLA-DRB1*07:01;HLA-DPA1*01:03/DPB1*02:01;HLA-DQA1*04:01/DQB1*04:02;HLA-DPA1*01/DPB1*04:01;HLA-DRB3*01:01;HLA-DPA1*02:01/DPB1*05:01;HLA-DQA1*03:01/DQB1*03:02;HLA-DRB4*01:01	1214-1228	HTL 1

PWYIWLGFI AGLIAI	14	23	HLA-DQA1*01:01/DQB1*05:01;HLA-DPA1*01:03/DPB1*02:01;HLA-DQA1*05:01/DQB1*03:01;HLA-DRB1*01:01;HLA-DQA1*05:01/DQB1*02:01;HLA-DPA1*02:01/DPB1*01:01;HLA-DPA1*03:01/DPB1*04:02;HLA-DRB1*07:01;HLA-DPA1*01/DPB1*04:01;HLA-DRB1*09:01;HLA-DPA1*02:01/DPB1*05:01;HLA-DQA1*04:01/DQB1*04:02;HLA-DQA1*03:01/DQB1*03:02;HLA-DRB4*01:01	1213-1227	HTL 2
WLGFIAGLI AIVMVT	13	24	HLA-DQA1*05:01/DQB1*03:01;HLA-DPA1*03:01/DPB1*04:02;HLA-DRB1*09:01;HLA-DRB1*01:01;HLA-DQA1*04:01/DQB1*04:02;HLA-DQA1*01:01/DQB1*05:01;HLA-DQA1*01:02/DQB1*06:02;HLA-DQA1*05:01/DQB1*02:01;HLA-DPA1*01:03/DPB1*02:01;HLA-DPA1*02:01/DPB1*01:01;HLA-DQA1*03:01/DQB1*03:02;HLA-DRB3*01:01;HLA-DRB1*07:01	1217-1231	HTL 3

YIWLGFIAG LIAIVM	13	23	HLA-DQA1*05:01/DQB1*03:01;HLA-DQA1*01:01/DQB1*05:01;HLA-DRB1*01:01;HLA-DRB1*09:01;HLA-DPA1*03:01/DPB1*04:02;HLA-DQA1*05:01/DQB1*02:01;HLA-DQA1*01:02/DQB1*06:02;HLA-DRB1*07:01;HLA-DQA1*04:01/DQB1*04:02;HLA-DPA1*01:03/DPB1*02:01;HLA-DPA1*02:01/DPB1*01:01;HLA-DRB3*01:01;HLA-DQA1*03:01/DQB1*03:02	1215-1229	HTL 4
IWLGFIAGLI AIVMV	13	23	HLA-DQA1*05:01/DQB1*03:01;HLA-DRB1*01:01;HLA-DRB1*09:01;HLA-DPA1*03:01/DPB1*04:02;HLA-DQA1*01:01/DQB1*05:01;HLA-DQA1*04:01/DQB1*04:02;HLA-DQA1*01:02/DQB1*06:02;HLA-DQA1*05:01/DQB1*02:01;HLA-DPA1*01:03/DPB1*02:01;HLA-DPA1*02:01/DPB1*01:01;HLA-DRB1*07:01;HLA-DRB3*01:01;HLA-DQA1*03:01/DQB1*03:02	1216-1230	HTL 5

LGFIAGLIAI VMVTI	12	24	HLA-DQA1*05:01/DQB1*03:01;HLA-DPA1*03:01/DPB1*04:02;HLA-DQA1*01:02/DQB1*06:02;HLA-DRB1*01:01;HLA-DQA1*05:01/DQB1*02:01;HLA-DRB1*09:01;HLA-DQA1*04:01/DQB1*04:02;HLA-DPA1*02:01/DPB1*01:01;HLA-DQA1*01:01/DQB1*05:01;HLA-DPA1*01:03/DPB1*02:01;HLA-DQA1*03:01/DQB1*03:02;HLA-DRB3*01:01	1218-1232	HTL 6
WPWYIWLG FIAGLIA	12	23	HLA-DQA1*01:01/DQB1*05:01;HLA-DPA1*01:03/DPB1*02:01;HLA-DQA1*05:01/DQB1*02:01;HLA-DRB1*01:01;HLA-DQA1*05:01/DQB1*03:01;HLA-DPA1*02:01/DPB1*01:01;HLA-DPA1*03:01/DPB1*04:02;HLA-DRB1*07:01;HLA-DPA1*01/DPB1*04:01;HLA-DRB1*09:01;HLA-DPA1*02:01/DPB1*05:01;HLA-DRB4*01:01	1212-1226	HTL 7
GVVFLHVTY VPAQEK	11	24	HLA-DPA1*01/DPB1*04:01;HLA-DPA1*01:03/DPB1*02:01;HLA-DRB1*01:01;HLA-DQA1*05:01/DQB1*02:01;HLA-DQA1*04:01/DQB1*04:02;HLA-DQA1*03:01/DQB1*03:02;HLA-DRB1*07:01;HLA-DPA1*03:01/DPB1*04:02;HLA-DPA1*02:01/DPB1*01:01;HLA-DQA1*01:02/DQB1*06:02;HLA-DRB4*01:01	1059-1073	HTL 8

VVFLHVTYV PAQEKN	11	24	HLA-DPA1*01/DPB1*04:01;HLA-DPA1*01:03/DPB1*02:01;HLA-DQA1*04:01/DQB1*04:02;HLA-DQA1*03:01/DQB1*03:02;HLA-DQA1*05:01/DQB1*02:01;HLA-DRB1*01:01;HLA-DRB1*07:01;HLA-DQA1*01:02/DQB1*06:02;HLA-DPA1*02:01/DPB1*01:01;HLA-DPA1*03:01/DPB1*04:02;HLA-DRB4*01:01	1060-1074	HTL 9
GFIAGLIAIV MVTIM	11	23	HLA-DQA1*05:01/DQB1*03:01;HLA-DPA1*03:01/DPB1*04:02;HLA-DQA1*01:02/DQB1*06:02;HLA-DRB1*01:01;HLA-DQA1*05:01/DQB1*02:01;HLA-DQA1*04:01/DQB1*04:02;HLA-DRB1*09:01;HLA-DPA1*02:01/DPB1*01:01;HLA-DPA1*01:03/DPB1*02:01;HLA-DRB4*01:01;HLA-DQA1*03:01/DQB1*03:02	1219-1233	HTL 10
ADYSVLYNF APFFAF	11	13	HLA-DRB3*01:01;HLA-DPA1*01:03/DPB1*02:01;HLA-DQA1*01:01/DQB1*05:01;HLA-DPA1*02:01/DPB1*05:01;HLA-DPA1*02:01/DPB1*01:01;HLA-DQA1*04:01/DQB1*04:02;HLA-DQA1*05:01/DQB1*02:01;HLA-DQA1*03:01/DQB1*03:02;HLA-DPA1*01/DPB1*04:01;HLA-DRB1*01:01;HLA-DRB1*09:01	363-377	HTL 11

FIAGLIAIVM VTIML	10	24	HLA-DQA1*05:01/DQB1*03:01;HLA-DPA1*03:01/DPB1*04:02;HLA-DRB4*01:01;HLA-DQA1*01:02/DQB1*06:02;HLA-DQA1*05:01/DQB1*02:01;HLA-DQA1*04:01/DQB1*04:02;HLA-DRB1*01:01;HLA-DPA1*02:01/DPB1*01:01;HLA-DPA1*01/DPB1*04:01;HLA-DRB1*09:01	1220-1234	HTL 12
DLCFTNVYA DSFVIR	10	24	HLA-DQA1*01:01/DQB1*05:01;HLA-DPA1*01:03/DPB1*02:01;HLA-DQA1*05:01/DQB1*02:01;HLA-DPA1*01/DPB1*04:01;HLA-DRB3*01:01;HLA-DPA1*03:01/DPB1*04:02;HLA-DRB1*07:01;HLA-DPA1*02:01/DPB1*01:01;HLA-DQA1*01:02/DQB1*06:02;HLA-DPA1*02:01/DPB1*05:01	389-403	HTL 13
QPYRVVVLS FELLHA	10	24	HLA-DPA1*03:01/DPB1*04:02;HLA-DPA1*02:01/DPB1*01:01;HLA-DPA1*01:03/DPB1*02:01;HLA-DPA1*01/DPB1*04:01;HLA-DPA1*02:01/DPB1*05:01;HLA-DRB4*01:01;HLA-DQA1*01:01/DQB1*05:01;HLA-DRB1*07:01;HLA-DQA1*05:01/DQB1*02:01;HLA-DQA1*03:01/DQB1*03:02	506-520	HTL 14

RVVVLSFEL LHAPAT	10	24	HLA-DRB1*01:01;HLA-DPA1*02:01/DPB1*01:01;HLA-DPA1*03:01/DPB1*04:02;HLA-DPA1*01:03/DPB1*02:01;HLA-DPA1*01/DPB1*04:01;HLA-DPA1*02:01/DPB1*05:01;HLA-DQA1*01:01/DQB1*05:01;HLA-DRB4*01:01;HLA-DQA1*05:01/DQB1*02:01;HLA-DRB1*09:01	509-523	HTL 15
VVVLSFELL HAPATV	10	24	HLA-DRB1*01:01;HLA-DPA1*02:01/DPB1*01:01;HLA-DPA1*03:01/DPB1*04:02;HLA-DPA1*01:03/DPB1*02:01;HLA-DPA1*01/DPB1*04:01;HLA-DPA1*02:01/DPB1*05:01;HLA-DQA1*01:01/DQB1*05:01;HLA-DRB1*09:01;HLA-DQA1*05:01/DQB1*02:01;HLA-DRB4*01:01	510-524	HTL 16
MIAQYTSAL LAGTIT	10	24	HLA-DQA1*01:02/DQB1*06:02;HLA-DQA1*05:01/DQB1*03:01;HLA-DRB1*01:01;HLA-DPA1*03:01/DPB1*04:02;HLA-DRB1*09:01;HLA-DPA1*01/DPB1*04:01;HLA-DRB1*07:01;HLA-DPA1*02:01/DPB1*01:01;HLA-DPA1*02:01/DPB1*05:01;HLA-DPA1*01:03/DPB1*02:01	869-883	HTL 17

KWPWYIWL GFIAGLI	10	23	HLA-DQA1*01:01/DQB1*05:01;HLA-DPA1*01:03/DPB1*02:01;HLA-DQA1*05:01/DQB1*02:01;HLA-DPA1*02:01/DPB1*01:01;HLA-DPA1*03:01/DPB1*04:02;HLA-DRB1*01:01;HLA-DRB1*07:01;HLA-DPA1*01/DPB1*04:01;HLA-DQA1*05:01/DQB1*03:01;HLA-DPA1*02:01/DPB1*05:01	1211-1225	HTL 18
PTNFTISVTT EILPV	10	23	HLA-DRB1*07:01;HLA-DRB1*09:01;HLA-DQA1*03:01/DQB1*03:02;HLA-DQA1*05:01/DQB1*02:01;HLA-DPA1*02:01/DPB1*01:01;HLA-DPA1*01/DPB1*04:01;HLA-DPA1*03:01/DPB1*04:02;HLA-DRB3*01:01;HLA-DQA1*04:01/DQB1*04:02;HLA-DPA1*01:03/DPB1*02:01	715-729	HTL 19
LCFTNVYAD SFVIRG	9	24	HLA-DQA1*01:01/DQB1*05:01;HLA-DPA1*01:03/DPB1*02:01;HLA-DQA1*05:01/DQB1*02:01;HLA-DPA1*03:01/DPB1*04:02;HLA-DPA1*01/DPB1*04:01;HLA-DRB3*01:01;HLA-DRB1*07:01;HLA-DPA1*02:01/DPB1*01:01;HLA-DQA1*03:01/DQB1*03:02	390-404	HTL 20

STQDLFLPFF SNVTW	9	24	HLA-DPA1*01:03/DPB1*02:01;HLA-DPA1*02:01/DPB1*01:01;HLA-DPA1*03:01/DPB1*04:02;HLA-DPA1*01/DPB1*04:01;HLA-DQA1*01:01/DQB1*05:01;HLA-DPA1*02:01/DPB1*05:01;HLA-DRB1*01:01;HLA-DQA1*05:01/DQB1*02:01;HLA-DRB1*07:01	50-64	HTL 21
PYRVVVLSF ELLHAP	9	24	HLA-DPA1*03:01/DPB1*04:02;HLA-DPA1*02:01/DPB1*01:01;HLA-DPA1*01:03/DPB1*02:01;HLA-DPA1*01/DPB1*04:01;HLA-DPA1*02:01/DPB1*05:01;HLA-DRB4*01:01;HLA-DQA1*01:01/DQB1*05:01;HLA-DRB1*07:01;HLA-DQA1*05:01/DQB1*02:01	507-521	HTL 22
YRVVVLSFE LLHAPA	9	24	HLA-DPA1*03:01/DPB1*04:02;HLA-DPA1*02:01/DPB1*01:01;HLA-DPA1*01:03/DPB1*02:01;HLA-DPA1*01/DPB1*04:01;HLA-DPA1*02:01/DPB1*05:01;HLA-DRB4*01:01;HLA-DQA1*01:01/DQB1*05:01;HLA-DQA1*05:01/DQB1*02:01;HLA-DQA1*03:01/DQB1*03:02	508-522	HTL 23

FVFLVLLPL VSSQCV	9	23	HLA-DRB1*01:01;HLA-DPA1*03:01/DPB1*04:02;HLA-DPA1*02:01/DPB1*01:01;HLA-DPA1*01/DPB1*04:01;HLA-DPA1*02:01/DPB1*05:01;HLA-DPA1*01:03/DPB1*02:01;HLA-DQA1*01:01/DQB1*05:01;HLA-DRB4*01:01;HLA-DQA1*05:01/DQB1*02:01	Feb-16	HTL 24
GHQPYRVV VLSFELL	9	19	HLA-DPA1*02:01/DPB1*01:01;HLA-DPA1*01:03/DPB1*02:01;HLA-DPA1*01/DPB1*04:01;HLA-DPA1*02:01/DPB1*05:01;HLA-DRB4*01:01;HLA-DPA1*03:01/DPB1*04:02;HLA-DRB1*07:01;HLA-DQA1*05:01/DQB1*02:01;HLA-DQA1*03:01/DQB1*03:02	504-518	HTL 25
IRAAEIRASA NLAAT	8	24	HLA-DQA1*01:02/DQB1*06:02;HLA-DQA1*05:01/DQB1*03:01;HLA-DRB1*09:01;HLA-DRB4*01:01;HLA-DRB1*07:01;HLA-DQA1*03:01/DQB1*03:02;HLA-DQA1*04:01/DQB1*04:02;HLA-DRB1*01:01	1013-1027	HTL 26

HGVVFLHVT YVPAQE	8	24	HLA-DPA1*01:03/DPB1*02:01;HLA-DPA1*01/DPB1*04:01;HLA-DRB1*01:01;HLA-DQA1*05:01/DQB1*02:01;HLA-DRB1*07:01;HLA-DPA1*03:01/DPB1*04:02;HLA-DPA1*02:01/DPB1*01:01;HLA-DQA1*03:01/DQB1*03:02	1058-1072	HTL 27
IAGLIAIVMV TIMLC	8	24	HLA-DQA1*01:02/DQB1*06:02;HLA-DPA1*03:01/DPB1*04:02;HLA-DRB4*01:01;HLA-DPA1*02:01/DPB1*01:01;HLA-DQA1*05:01/DQB1*03:01;HLA-DRB1*01:01;HLA-DPA1*01/DPB1*04:01;HLA-DQA1*04:01/DQB1*04:02	1221-1235	HTL 28
CTFEYVSQP FLMDLE	8	24	HLA-DPA1*01:03/DPB1*02:01;HLA-DPA1*02:01/DPB1*01:01;HLA-DPA1*03:01/DPB1*04:02;HLA-DQA1*05:01/DQB1*02:01;HLA-DRB1*09:01;HLA-DRB1*07:01;HLA-DQA1*01:01/DQB1*05:01;HLA-DRB1*01:01	166-180	HTL 29
AAYYVGYL QPRTFLL	8	24	HLA-DRB1*01:01;HLA-DPA1*02:01/DPB1*01:01;HLA-DPA1*01:03/DPB1*02:01;HLA-DPA1*02:01/DPB1*05:01;HLA-DRB1*07:01;HLA-DPA1*01/DPB1*04:01;HLA-DPA1*03:01/DPB1*04:02;HLA-DRB1*09:01	263-277	HTL 30

AYYVGYLQ PRTFLLK	8	24	HLA-DPA1*01:03/DPB1*02:01;HLA-DRB1*01:01;HLA-DPA1*02:01/DPB1*05:01;HLA-DPA1*02:01/DPB1*01:01;HLA-DPA1*03:01/DPB1*04:02;HLA-DPA1*01/DPB1*04:01;HLA-DRB1*07:01;HLA-DRB1*09:01	264-278	HTL 31
KGIYQTSNF RVQPTE	8	24	HLA-DPA1*01/DPB1*04:01;HLA-DPA1*02:01/DPB1*01:01;HLA-DQA1*01:02/DQB1*06:02;HLA-DRB1*07:01;HLA-DPA1*02:01/DPB1*05:01;HLA-DPA1*01:03/DPB1*02:01;HLA-DQA1*01:01/DQB1*05:01;HLA-DRB1*01:01	310-324	HTL 32
IYQTSNFRV QPTESI	8	24	HLA-DRB3*01:01;HLA-DRB1*07:01;HLA-DRB4*01:01;HLA-DQA1*01:02/DQB1*06:02;HLA-DPA1*03:01/DPB1*04:02;HLA-DPA1*01/DPB1*04:01;HLA-DQA1*01:01/DQB1*05:01;HLA-DPA1*02:01/DPB1*01:01	312-326	HTL 33

MFVFLVLLP LVSSQC	8	23	HLA-DRB1*01:01;HLA-DPA1*03:01/DPB1*04:02;HLA-DPA1*02:01/DPB1*01:01;HLA-DPA1*01/DPB1*04:01;HLA-DPA1*02:01/DPB1*05:01;HLA-DPA1*01:03/DPB1*02:01;HLA-DQA1*05:01/DQB1*02:01;HLA-DQA1*01:01/DQB1*05:01	Jan-15	HTL 34
GINITRFQTL LALHR	8	23	HLA-DRB4*01:01;HLA-DPA1*03:01/DPB1*04:02;HLA-DPA1*02:01/DPB1*05:01;HLA-DPA1*01/DPB1*04:01;HLA-DPA1*01:03/DPB1*02:01;HLA-DPA1*02:01/DPB1*01:01;HLA-DRB1*01:01;HLA-DRB1*07:01	232-246	HTL 35
SQSIIAYTMS LGAEN	8	23	HLA-DRB1*09:01;HLA-DQA1*01:02/DQB1*06:02;HLA-DQA1*03:01/DQB1*03:02;HLA-DQA1*05:01/DQB1*02:01;HLA-DRB1*07:01;HLA-DPA1*03:01/DPB1*04:02;HLA-DQA1*04:01/DQB1*04:02;HLA-DRB1*01:01	689-703	HTL 36

QSIIAYTMSL GAENS	8	22	HLA-DRB1*09:01;HLA-DQA1*01:02/DQB1*06:02;HLA-DRB1*07:01;HLA-DQA1*03:01/DQB1*03:02;HLA-DPA1*03:01/DPB1*04:02;HLA-DRB1*01:01;HLA-DQA1*04:01/DQB1*04:02;HLA-DQA1*05:01/DQB1*02:01	690-704	HTL 37
TQLKRALTG IAVEQD	8	17	HLA-DQA1*03:01/DQB1*03:02;HLA-DQA1*04:01/DQB1*04:02;HLA-DQA1*01:02/DQB1*06:02;HLA-DRB1*01:01;HLA-DRB1*09:01;HLA-DQA1*05:01/DQB1*02:01;HLA-DRB4*01:01;HLA-DQA1*05:01/DQB1*03:01	761-775	HTL 38
QLKRALTGI AVEQDK	8	17	HLA-DQA1*03:01/DQB1*03:02;HLA-DQA1*04:01/DQB1*04:02;HLA-DQA1*01:02/DQB1*06:02;HLA-DRB1*01:01;HLA-DRB1*09:01;HLA-DQA1*05:01/DQB1*02:01;HLA-DRB4*01:01;HLA-DQA1*05:01/DQB1*03:01	762-776	HTL 39

LKRALTGIA VEQDKN	8	17	HLA-DQA1*04:01/DQB1*04:02;HLA-DQA1*03:01/DQB1*03:02;HLA-DQA1*01:02/DQB1*06:02;HLA-DRB1*01:01;HLA-DQA1*05:01/DQB1*02:01;HLA-DRB4*01:01;HLA-DQA1*05:01/DQB1*03:01;HLA-DRB1*09:01	763-777	HTL 40
WTAGAAAY YVGYLQP	7	24	HLA-DQA1*05:01/DQB1*03:01;HLA-DPA1*01:03/DPB1*02:01;HLA-DQA1*01:02/DQB1*06:02;HLA-DQA1*04:01/DQB1*04:02;HLA-DQA1*01:01/DQB1*05:01;HLA-DRB1*09:01;HLA-DPA1*01/DPB1*04:01	258-272	HTL 41
AAAYYVGY LQPRTFL	7	24	HLA-DPA1*01:03/DPB1*02:01;HLA-DPA1*02:01/DPB1*01:01;HLA-DPA1*01/DPB1*04:01;HLA-DPA1*02:01/DPB1*05:01;HLA-DQA1*01:01/DQB1*05:01;HLA-DRB1*01:01;HLA-DRB1*09:01	262-276	HTL 42
YVGYLQPRT FLLKYN	7	24	HLA-DPA1*02:01/DPB1*05:01;HLA-DPA1*01:03/DPB1*02:01;HLA-DRB1*01:01;HLA-DPA1*01/DPB1*04:01;HLA-DPA1*02:01/DPB1*01:01;HLA-DPA1*03:01/DPB1*04:02;HLA-DRB1*07:01	266-280	HTL 43

NDLCFTNVY ADSFVI	7	24	HLA-DRB1*07:01;HLA-DRB3*01:01;HLA-DPA1*01:03/DPB1*02:01;HLA-DQA1*01:01/DQB1*05:01;HLA-DQA1*05:01/DQB1*02:01;HLA-DQA1*04:01/DQB1*04:02;HLA-DQA1*03:01/DQB1*03:02	388-402	HTL 44
VVLSFELLH APATVC	7	24	HLA-DRB1*01:01;HLA-DRB1*09:01;HLA-DPA1*03:01/DPB1*04:02;HLA-DQA1*01:01/DQB1*05:01;HLA-DPA1*02:01/DPB1*01:01;HLA-DRB1*07:01;HLA-DPA1*01/DPB1*04:01	511-525	HTL 45
IAQYTSALL AGTITS	7	24	HLA-DQA1*01:02/DQB1*06:02;HLA-DQA1*05:01/DQB1*03:01;HLA-DRB1*01:01;HLA-DPA1*03:01/DPB1*04:02;HLA-DRB1*09:01;HLA-DPA1*02:01/DPB1*05:01;HLA-DRB1*07:01	870-884	HTL 46

YIKWPWYI WLGFIAG	7	23	HLA-DPA1*01:03/DPB1*02:01;HLA-DPA1*02:01/DPB1*01:01;HLA-DQA1*01:01/DQB1*05:01;HLA-DPA1*02:01/DPB1*05:01;HLA-DPA1*01/DPB1*04:01;HLA-DPA1*03:01/DPB1*04:02;HLA-DQA1*05:01/DQB1*02:01	1209-1223	HTL 47
IKWPWYIWL GFIAGL	7	23	HLA-DPA1*01:03/DPB1*02:01;HLA-DPA1*02:01/DPB1*01:01;HLA-DQA1*01:01/DQB1*05:01;HLA-DPA1*02:01/DPB1*05:01;HLA-DPA1*03:01/DPB1*04:02;HLA-DPA1*01/DPB1*04:01;HLA-DQA1*05:01/DQB1*02:01	1210-1224	HTL 48
ASQSIIAYTM SLGAE	7	23	HLA-DQA1*01:02/DQB1*06:02;HLA-DRB1*07:01;HLA-DQA1*03:01/DQB1*03:02;HLA-DQA1*04:01/DQB1*04:02;HLA-DPA1*03:01/DPB1*04:02;HLA-DQA1*05:01/DQB1*03:01;HLA-DRB1*01:01	688-702	HTL 49
SGWTAGAA AYYVGYL	7	20	HLA-DQA1*05:01/DQB1*03:01;HLA-DRB1*09:01;HLA-DQA1*04:01/DQB1*04:02;HLA-DRB1*01:01;HLA-DQA1*01:02/DQB1*06:02;HLA-DQA1*01:01/DQB1*05:01;HLA-DQA1*05:01/DQB1*02:01	256-270	HTL 50

VGHQPYRV VVLSFEL	7	19	HLA-DPA1*01:03/DPB1*02:01;HLA-DRB1*07:01;HLA-DPA1*02:01/DPB1*05:01;HLA-DQA1*03:01/DQB1*03:02;HLA-DQA1*05:01/DQB1*02:01;HLA-DPA1*02:01/DPB1*01:01;HLA-DPA1*01/DPB1*04:01	503-517	HTL 51
CTQLKRALT GIAVEQ	7	17	HLA-DQA1*04:01/DQB1*04:02;HLA-DRB1*09:01;HLA-DRB1*01:01;HLA-DQA1*03:01/DQB1*03:02;HLA-DQA1*01:02/DQB1*06:02;HLA-DRB4*01:01;HLA-DQA1*05:01/DQB1*03:01	760-774	HTL 52
