# Supplementary material for: An Immunoinformatic Approach for Identifying and Designing Conserved Multi-Epitope Vaccines for Coronaviruses
Source: Biomedicines. 2024 Nov 5;12(11):2530. doi: 10.3390/biomedicines12112530 (PMC11592158; doi:10.3390/biomedicines12112530)
Supplement: Supplementary file 1 [file biomedicines-12-02530-s001.zip › Supplementary Table S2.pdf]

Supplementary Table S2. PRODIGY analysis of the docked complexes

Docked Complexes	Gibb's Free Energy (kcal/mol)	K_d (M) at 25° C	Number of Interfacial Contacts (ICs) per property						Non- Interacting Surface (NIS) per property	
			ICs charged-charged	ICs charged-polar	ICs charged-apolar	ICs polar-polar	ICs polar-apolar	ICs apolar-apolar	NIS charged (%)	NIS apolar (%)
S1-TLR2	-14.8	$1.50x10^{-11}$	8	9	32	1	20	22	27.23	30.66
S2-TLR2	-13.0	$2.80x10^{-10}$	1	7	18	3	23	33	27.17	30.44
S1-TLR4	-17.1	$2.80x10^{-13}$	1	8	39	11	37	39	25.15	29.94
S2-TLR4	-18.8	$1.70x10^{-14}$	8	8	39	5	36	30	24.58	30.12
